# Increased intron retention is a post‐transcriptional signature associated with progressive aging and Alzheimer’s disease

**DOI:** 10.1111/acel.12928

**Published:** 2019-03-13

**Authors:** Swarnaseetha Adusumalli, Zhen‐Kai Ngian, Wei‐Qi Lin, Touati Benoukraf, Chin‐Tong Ong

**Affiliations:** ^1^ Temasek Life Sciences Laboratory National University of Singapore Singapore; ^2^ Department of Biological Sciences National University of Singapore Singapore; ^3^ Cancer Science Institute of Singapore National University of Singapore Singapore; ^4^ Discipline of Genetics, Faculty of Medicine Memorial University of Newfoundland St. John’s Newfoundland and Labrador Canada

**Keywords:** aging, Alzheimer’s disease, Drosophila, human, Intron retention, mouse

## Abstract

Intron retention (IR) by alternative splicing is a conserved regulatory mechanism that can affect gene expression and protein function during adult development and age‐onset diseases. However, it remains unclear whether IR undergoes spatial or temporal changes during different stages of aging or neurodegeneration like Alzheimer's disease (AD). By profiling the transcriptome of *Drosophila* head cells at different ages, we observed a significant increase in IR events for many genes during aging. Differential IR affects distinct biological functions at different ages and occurs at several AD‐associated genes in older adults. The increased nucleosome occupancy at the differentially retained introns in young animals suggests that it may regulate the level of IR during aging. Notably, an increase in the number of IR events was also observed in healthy older mouse and human brain tissues, as well as in the cerebellum and frontal cortex from independent AD cohorts. Genes with differential IR shared many common features, including shorter intron length, no perturbation in their mRNA level, and enrichment for biological functions that are associated with mRNA processing and proteostasis. The differentially retained introns identified in AD frontal cortex have higher GC content, with many of their mRNA transcripts showing an altered level of protein expression compared to control samples. Taken together, our results suggest that an increased IR is an conserved signature that is associated with aging. By affecting pathways involved in mRNA and protein homeostasis, changes of IR pattern during aging may regulate the transition from healthy to pathological state in late‐onset sporadic AD.

## 1 INTRODUCTION

Alternative splicing is a regulatory mechanism that generates multiple mRNA transcripts from a single gene which allows significant expansion in the proteome diversity (Baralle & Giudice, 2017). While this process is essential for many biological processes such as neurogenesis (Furlanis & Scheiffele, 2018), alteration in the splicing patterns is also prevalent during aging (Deschênes & Chabot, 2017; Stilling et al., 2014) and may contribute to many age‐onset diseases like Alzheimer's disease (AD) (Bai et al., 2013; Tollervey et al., 2011).

Intron retention (IR) occurs when a specific intron remains unspliced in the mature polyadenylated mRNA. As an IR may trigger nonsense‐mediated decay (NMD) of mRNA or introduce mutation in the translated protein, it has been widely considered as an aberrant splicing event that is associated with various diseases (Wong, Au, Ritchie, & Rasko, 2016). For instance, dysregulated IR is one of the drivers of transcriptome diversity in cancer (Dvinge & Bradley, 2015) and can lead to inactivation of different tumor‐suppressor genes (Jung et al., 2015). IR in endoglin and *EAAT2* gene also leads to cellular senescence (Blanco & Bernabeu, 2011) and amyotrophic lateral sclerosis, respectively (Lin et al., 1998). Interestingly, dietary restriction in worms could reduce aberrant IR caused by defective splicing during aging (Heintz et al., 2017), suggesting that IR at specific genes can be used as disease biomarkers or targets for therapeutic intervention.

Accumulated evidence indicated that IR may also play an important regulatory role during normal development, including translational inhibition in response to hypoxic stress (Brady et al., 2017), regulation of mRNA expression patterns during hematopoiesis (Cho et al., 2014; Wong et al., 2013) and neurogenesis (Braunschweig et al., 2014; Buckley et al., 2011). Therefore, defining age‐associated changes to IR may allow a far better understanding into how IR may regulate the transition from healthy to the pathological state during aging.

To this end, we analyzed the in‐house RNA‐sequencing (RNA‐seq) data of aging male *Drosophila* heads and observed a global increase in the level of IR as the animals aged. Interestingly, IR affects functionally distinct groups of genes at different stages of an adult lifespan. Consistent with the role of chromatin structure in regulating RNA splicing (Spies, Nielsen, Padgett, & Burge, 2009), we found that nucleosome positioning within a subset of introns in young flies correlated with their differential retention in older animals. Further analyses of transcriptome from mouse and human brain tissues (Lister et al., 2013; Mazin et al., 2014; Stilling et al., 2014) suggest that the global increase in IR during aging may be evolutionarily conserved. The differentially retained introns identified from different species share several similar characteristics, including shorter length when compared to spliced introns and not susceptible to NMD. Notably, several differential IR genes identified from aging *Drosophila* and human brain tissues are linked to AD‐related pathways, postulating that the pattern of IR may undergo further changes during AD progression. To test this possibility, we analyzed AD datasets from the cerebellum (syn8612213) and frontal cortex (Bai et al., 2013), and observed a global increase in the level of IR in AD brain tissues when compared to the control samples. These differentially retained introns have a shorter length and higher GC content compared to the spliced introns. Differential IR genes are enriched for functions associated with RNA processing and protein homeostasis, with more than a hundred of them having an altered level of protein expression in AD frontal cortex. Taken together, our results suggest that a global increase in IR may be a transcriptional signature of aging that is conserved across species and differential IR at specific genes may contribute to the etiology of late‐onset sporadic AD.

## 2 RESULTS

### 2.1 Age‐dependent IR in Drosophila

**Figure 1:**
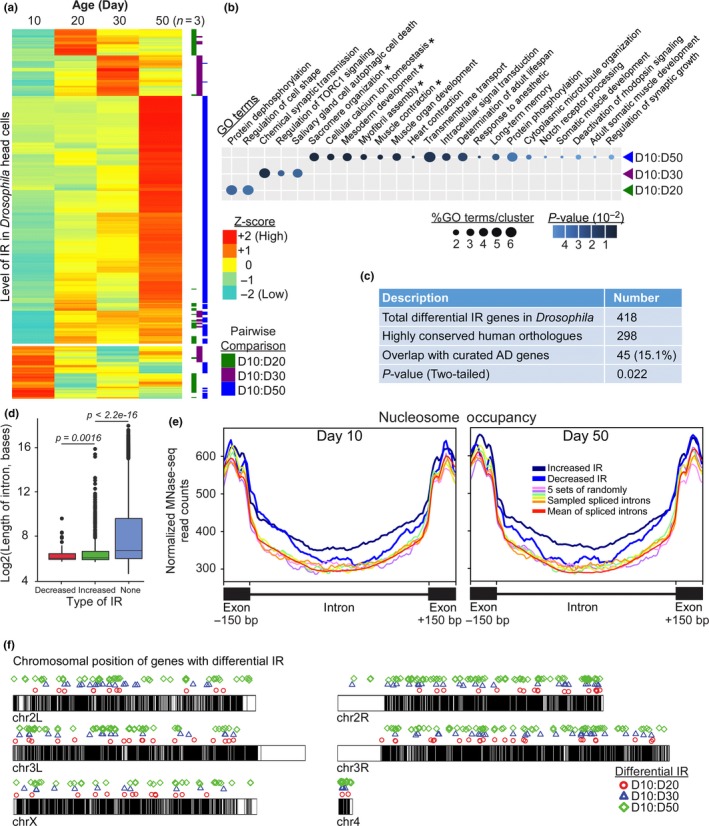
Differential intron retentions (IR) mark different stages of aging in *Drosophila*. (a) Expression heatmap of the differentially retained introns across various ages as represented by *Z*‐score. Pairwise comparison of the two age‐groups is highlighted by the color bar on the right. (b) Gene ontology analysis of the genes with differential IR in aging fly heads (*p*‐value <0.05, Fisher's exact test; *p‐value <0.05, Fisher's exact test with Benjamini–Hochberg correction). (c) The overlap between highly conserved human orthologues of fly differential IR genes and curated AD genes from DisGeNET. The *p*‐value was determined by two‐tailed chi‐square test with Yates’ continuity correction. (d) Boxplot for the length distribution of the various types of introns where D10 flies were used as the reference to determine the differential decreased or increased IR during aging. “None” refers to non‐retained, spliced introns (*p < *0.05, Welch's *t *test). (e) Normalized MNase‐seq read counts across different types of introns in Day 10 and 50 fly heads showed that nucleosome occupancy over differentially retained introns was significantly different from the spliced introns (*p* < 0.05, Wilcoxon rank sum test). The increased or decreased IR referred to the differentially retained introns at Day 50 with respect to Day 10. Five sets of spliced introns with similar expression level were randomly picked as control. (f) Ideogram displaying the genome‐wide distribution of the differential IR genes in fly

To study IR events during aging, we performed RNA‐seq of poly (A) + mRNAs isolated from the male *Drosophila* heads at four distinct time‐points (Supporting Information Figure S1). In *W^1118^* strain, day (D) 10 represents young adulthood. Significant changes in gene expression patterns between D20 and D30 suggest that this period may encompass the onset of aging (Zhan et al., 2007). Finally, D50 represents old adulthood. Three biological replicates were used for each of the time‐point, and IR events were computed using IRFinder algorithm, which quantifies the proportion of retained introns in mature mRNA transcripts as IR ratio (Middleton et al., 2017). The differential IR ratio calculated through pairwise comparison of two age‐groups was then scaled to generate global expression heatmap (Figure 1a). Genes were then clustered based on their IR patterns (Figure 1a). While there are a subset of genes with higher retained introns in young adults (D10, 20.8%), the majority of the increased IR events occurs at D50 (68.7%) (Figure 1a). Pairwise comparison between D10 and D20 yielded 88 differential IR events in 87 genes (*p < *0.05, DESeq2). There are 108 differential IR events in 105 genes between D10 and D30, which further increases to 368 IRs in 287 genes between D10 and D50 (Supporting Information Table S1). The increased IR events suggest a gradual decline in the global‐ and locus‐specific splicing efficiency as the animals aged.

We next asked whether the genes affected by differential IR are enriched for specific gene ontology (GO) categories. Notably, differential IR genes identified from different pairwise comparison are represented by distinct biological functions (*p*‐value <0.05, Fisher's exact test) (Figure 1b). Genes with differential retained introns at D20 are mostly involved in protein dephosphorylation and regulation of cell shape, whereas genes in TORC1 signaling pathway are affected by IR at D30. In D50 adult, the genes with differential IR are associated with other biological processes that include various aspects of muscular functions (*p*‐value <0.05, Fisher's exact test with Benjamini–Hochberg correction), memory, longevity, and protein phosphorylation (Figure 1b). Analyses with KEGG and PANTHER pathway databases revealed that some of the differential *Drosophila* IR genes may be associated with Alzheimer's disease–amyloid secretase pathways (Supporting Information Figure S2a). Similarly, ~15% of the highly conserved human orthologous counterparts of *Drosophila* differential IR genes overlapped with the curated AD genes (*p*‐value <0.05, two‐tailed chi‐square test with Yates’ correction) (Figure 1c; Supporting Information Table S1).

To better understand the mechanisms that may regulate age‐dependent changes in IR, we examined the genomic features that are associated with the retained introns in *Drosophila*. Consistent with the results in granulocytes differentiation and tumor progression (Dvinge & Bradley, 2015; Schmitz et al., 2017; Wong et al., 2013), the length of differentially retained introns in aging *Drosophila* was significantly shorter than the non‐retained introns (*p *= 5.263e−10 for decreased IR, Welch's *t *test) (Figure 1d). Given that RNA splicing can be regulated by chromatin states (Brown, Stoilov, & Xing, 2012; Spies et al., 2009), we examined the relationship between nucleosome occupancy and the differentially retained introns by MNase‐sequencing (MNase‐seq) in D10 and D50 male flies. Interestingly, in D10 animals, the level of normalized MNase‐seq read counts over the introns that become differentially retained at D50 is significantly higher than the randomly selected non‐retained introns (increased IR/non‐IR, *p* = 1.73e−06 and decreased IR/non‐IR, *p* = 5.685e−05, Wilcoxon rank sum test) (Figure 1e). However, the level of nucleosome occupancy is not significantly different between introns that have increased or decreased retention (*p* = 0.54). Similar nucleosome occupancy pattern is also observed in D50 animals (increased IR/non‐IR, *p* = 9.82e−08 and decreased IR/non‐IR, *p* = 5.24e−06). This suggests that stable nucleosome positioning within the introns of selected genes in young flies may contribute to their differential retention at older age. Finally, we did not observe any preference for the genomic location of genes that underwent age‐dependent differential IR (Figure 1f).

**Figure 2:**
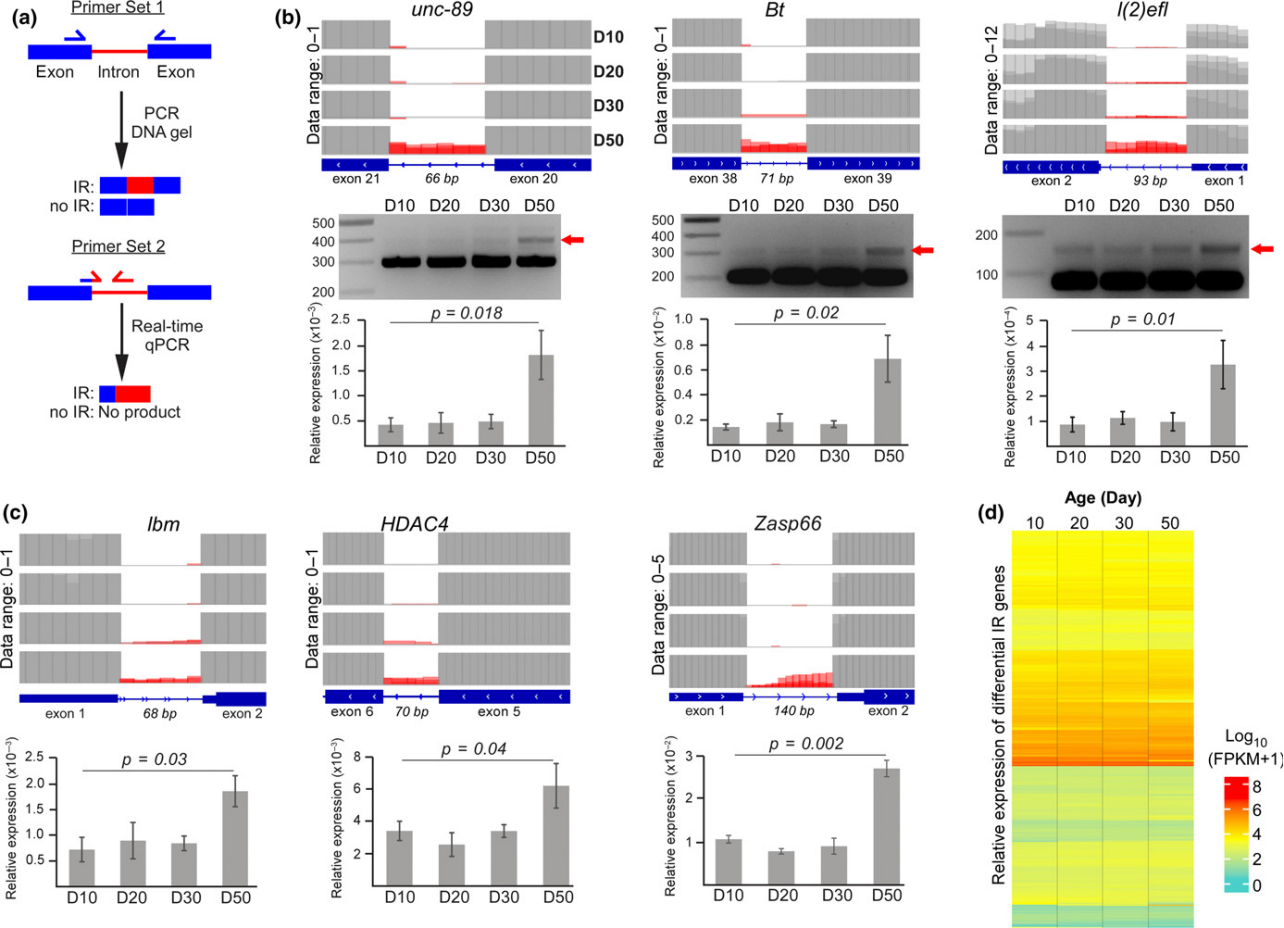
Experimental validation of IR at specific *Drosophila* genes. (a) Two different primer sets were designed to validate differential IR. (b, c) Top panel: The expression level of retained intron (red) and flanking exons (gray) on Integrative Genome Browser where the respective data range is indicated. Middle panel: Visualization of retained introns of three specific genes with DNA gel electrophoresis. Bottom panel: Real‐time quantitative PCR of differential retained introns using three biological replicates of fly heads for each time‐point (*p < *0.05, paired *t *test). (d) Relative expression of differential IR genes across various age‐groups as represented by Log_10 _(FPKM + 1) values

These differential IR events identified using IRFinder was validated via two approaches. The presence of both the retained and spliced introns was first visualized by designing a primer pair (set 1) that bound to the flanking exons (Figure 2a, top). Second, the level of differential IR was quantified with the real‐time PCR using primer pair (set 2) that recognizes only the exon–intron junction and the retained intron (Figure 2a, bottom). On the Integrated Genome View software, the level of the retained intron (red) is lower than the saturated exon signal (gray), indicating the low abundance of mRNA transcripts with retained intron (Figure 2b,c, top panel). The fully spliced exon–exon product was represented by a thick and fast migrating band on agarose gel (Figure 2b, middle panel). Consistent with increased IR during aging, the slower migrating band, which corresponds to mRNA species with retained intron, showed stronger intensity in D50 animals (Figure 2b). This result was further corroborated by real‐time PCR quantification where there is a significant difference in the level of retained introns between D10 and D50 animals (*p* < 0.05, *t *test, *n* = 3) (Figure 2b,c, bottom panel).

As differential IR of developmental genes can trigger NMD during differentiation (Braunschweig et al., 2014; Middleton et al., 2017; Wong et al., 2013), we asked whether mRNA from age‐dependent IR genes may be subjected to degradation. Unlike developmental genes, we did not observe any drastic change in their mRNA expression level in the aging flies (Figure 2d; Supporting Information Table S2). While this may be partly due to their low abundance, the overall unaltered expression suggests that these mRNA transcripts may be localized to the nucleus and are protected from NMD (Braunschweig et al., 2014; Mauger, Lemoine, & Scheiffele, 2016; Pimentel et al., 2016). Taken together, we show that there is an increase in the number of IR events as the flies aged, which unexpectedly has little effect on their mRNA expression.

To test whether IR affects protein translation, we screened through a collection of protein trap lines and found a line where green fluorescent protein (GFP) has been integrated into *HDAC4* gene upstream of its IR site (Supporting Information Figure S2b). Although the retained intron is in‐frame with the upstream exon, it introduces a premature termination codon that causes protein truncation (Supporting Information Figure S2c). When the nuclear lysates isolated from D10 and D50 heads were probed with anti‐GFP antibody, we observed an increase in the level of truncated HDAC4‐GFP protein in the older animals (Supporting Information Figure S2d). This indicated that age‐dependent increased IR at *HDAC4* gene can lead to the translation of premature terminated gene product in the older animals.

### 2.2 Age‐dependent changes in IR are highly conserved across species

**Figure 3:**
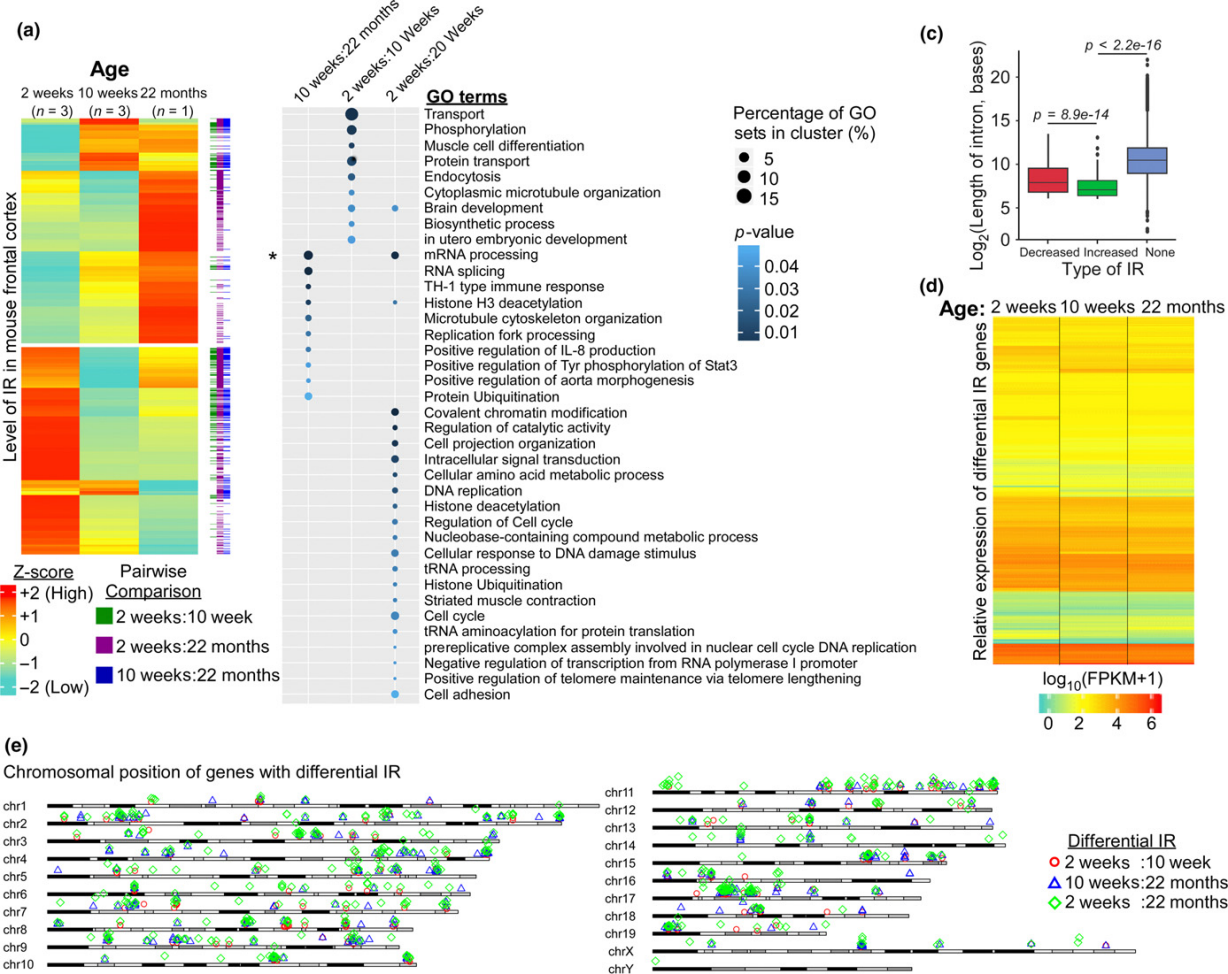
Intron retentions across three age‐groups in mouse frontal cortex. (a) Expression heatmap of differentially retained introns across three age‐groups (2, 10 weeks, and 22 months) in mouse frontal cortex. (b) Gene ontology analysis of the differential IR genes determined through pairwise comparison between two distinct time‐points (*p*‐value <0.05, Fisher's exact test; *p‐value <0.05, Fisher's exact test with Benjamini–Hochberg correction). (c) Boxplot for the length distribution of the differentially retained and spliced introns. The expression level of intron at 2 weeks was used as the reference to determine the decreased or increased IR during aging. “None” refers to spliced introns (*p*‐value <0.05, Welch's *t *test). (d) Relative expression of IR genes between different ages of mouse frontal cortex as represented by Log_10_ (FPKM + 1) values. (e) Ideogram displaying the distribution of the differential IR genes across the mouse genome

To address whether increased IR during aging is conserved, we analyzed the transcriptome from the mouse frontal cortex (Figure 3; Lister et al., 2013) and hippocampus (Supporting Information Figure S3) (Stilling et al., 2014). We observed highly dynamic differential IR pattern in the mouse's frontal cortex that were isolated from three different ages (2, 10 weeks, and 22 months) (Figure 3a). Consistent with the increased IR observed in aging *Drosophila*, there are 224 increased and 45 decreased IR events in 22‐month frontal cortex when compared to 10‐week‐old mouse (Supporting Information Table S3). Interestingly, compared to older mouse, there are a large number of increased IR events in 2‐week‐old frontal cortex (145 and 272 increased IR events compared to 10 weeks and 22 months, respectively). This observation can be explained by the roles of IR in regulating postnatal neuronal development and maturation (Buckley et al., 2011; Mauger et al., 2016). Similar to *Drosophila* (Figure 1b), GO analysis of differential IR genes revealed the enrichment of distinct biological functions at different age‐groups (*p*‐value <0.05, Fisher's exact test) (Figure 3b). For instance, genes with differential IR between 2‐ and 10‐week‐old mice are involved in protein transport, endocytosis, and brain development. On the other hand, differential IR genes between 10‐week and 22‐month‐old mice are involved in mRNA processing (*p*‐value <0.05, Fisher's exact test with Benjamini–Hochberg correction). These results suggest that differential IR may play distinct roles across adult lifespan: regulating brain development during the postnatal stage while contributing to the physiological changes during the aging. Consistent with our previous results, retained introns are consistently shorter than non‐retained introns (Figure 3c), they also do not exhibit significant changes to their level of mRNA expression (Figure 3d) and occur at genes that are well distributed across the genome (Figure 3e).

Similar to *Drosophila* and mouse frontal cortex, there is also an increase in the level of IR in older (24 and 29 months) hippocampus when compared to young mice (3 months) (Supporting Information Figure S3a and Table S4). Among the 98 differentially retained introns determined by IRFinder, 14 and 84 introns exhibited a higher level of retention in young and old hippocampus, respectively (Supporting Information Figure S3b). GO analysis of the differential IR genes revealed representation of genes involved in different aspects of RNA processing and metabolism of biomolecules (*p*‐value <0.05, Fisher's exact test) (Supporting Information Figure S3c & Table S4). This suggests a feedback mechanism whereby genes involved in pre‐mRNA processing may be regulated by IR during aging. Similar to our analysis and other studies, the length of differentially retained introns is significantly shorter than the non‐retained intron (Supporting Information Figure S3d). Moreover, these differential IR genes have unperturbed mRNA expression (Supporting Information Figure S3e) and are evenly distributed across the genome (Supporting Information Figure S3f). Similar to *Drosophila* (Figure 1c), the human orthologous counterparts of mouse differential IR genes have significant overlap with AD‐curated genes (*p*‐value <0.05, two‐tailed chi‐square test with Yates’ correction) (Supporting Information Figure S3g & Table S4).

Given that IR affects many orthologous or functionally related genes in granulocytes isolated from different species (Schmitz et al., 2017), we asked whether age‐dependent differential IR occurred at the conserved genes in *Drosophila* and mouse. Indeed, there are 45 conserved genes that have undergone differential IR in *Drosophila*, mouse hippocampus, and frontal cortex, although the position of retained introns is highly variable among the species (Supporting Information Table S5). Further studies using the same cell types from the brain will shed light if conserved pathways are indeed regulated by IR during aging across different species.

**Figure 4:**
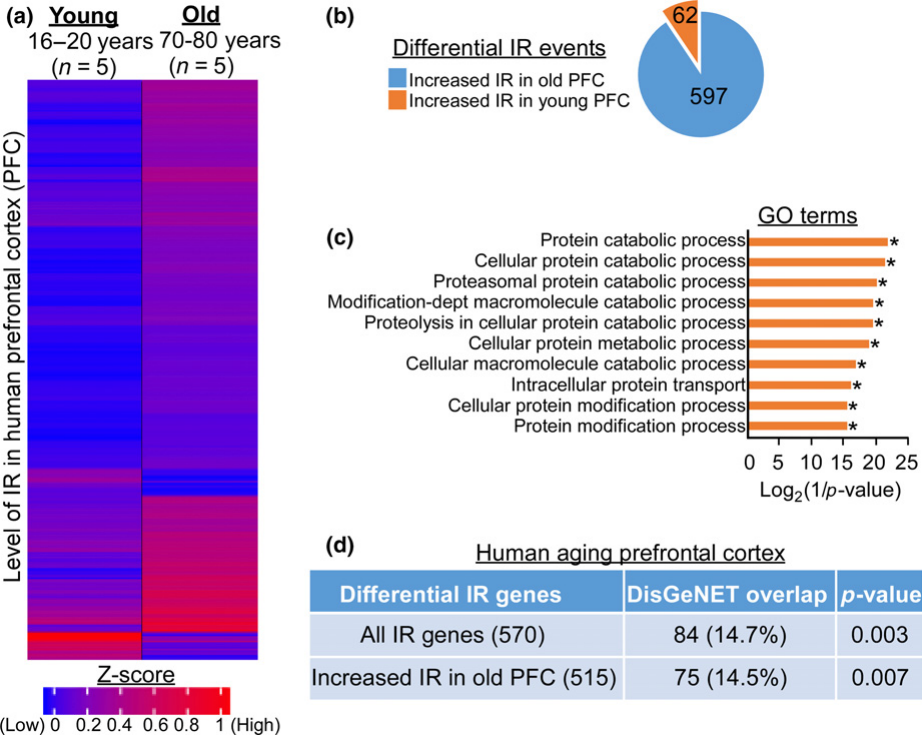
Genes with increased IR in old prefrontal cortex are linked to AD pathway. (a) Expression heatmap of differentially retained introns in young and old human prefrontal cortex (PFC). (b) Pie diagram showing the number of differentially increased IR events in young and old human PFC. (c) Gene ontology analysis of the genes with differential IR between young and old human PFC (*p‐value <0.05, Fisher's exact test with Benjamini–Hochberg correction). (d) The overlap of differential IR genes in aging PFC with curated AD genes from DisGeNET. The *p*‐value was determined by two‐tailed chi‐square test with Yates’ continuity correction

To address whether increased IR during aging is observed in human, we analyzed the transcriptome of human prefrontal cortex (PFC) isolated from two age‐groups (Mazin et al., 2014). We observed age‐dependent changes in IR patterns (Figure 4a), with 597 increased and 62 decreased IR events in older PFC when compared to young PFC (Figure 4b; Supporting Information Table S6). GO analysis revealed overrepresentation of differential IR genes that are involved in different aspects of proteostasis (*p‐*value <0.05, Fisher's exact test with Benjamini–Hochberg correction) (Figure 4c) with several of them linked to AD–amyloid secretase pathway (Supporting Information Figure S4a). Moreover, about 15% of the age‐dependent differential IR genes are overrepresented in curated AD gene‐list (*p*‐value <0.05, two‐tailed chi‐square test with Yates’ correction) (Figure 4d; Supporting Information Table S6). Consistent with the characteristics observed in fly and mouse, retained introns in human have shorter length than the spliced introns (Supporting Information Figure S4b); differential IR has no significant effect on the gene expression (Supporting Information Figure S4c) and occurs at genes that are well distributed across the genome (Supporting Information Figure S4d). Taken together, these results suggest that the increase in IR during aging is likely to be conserved across different species. It is also likely that differential IR during aging may contribute to the transition from healthy to prodromal AD state in the brain through regulating mRNA and protein homeostasis.

### 2.3 Increased IR in Alzheimer's disease

**Figure 5:**
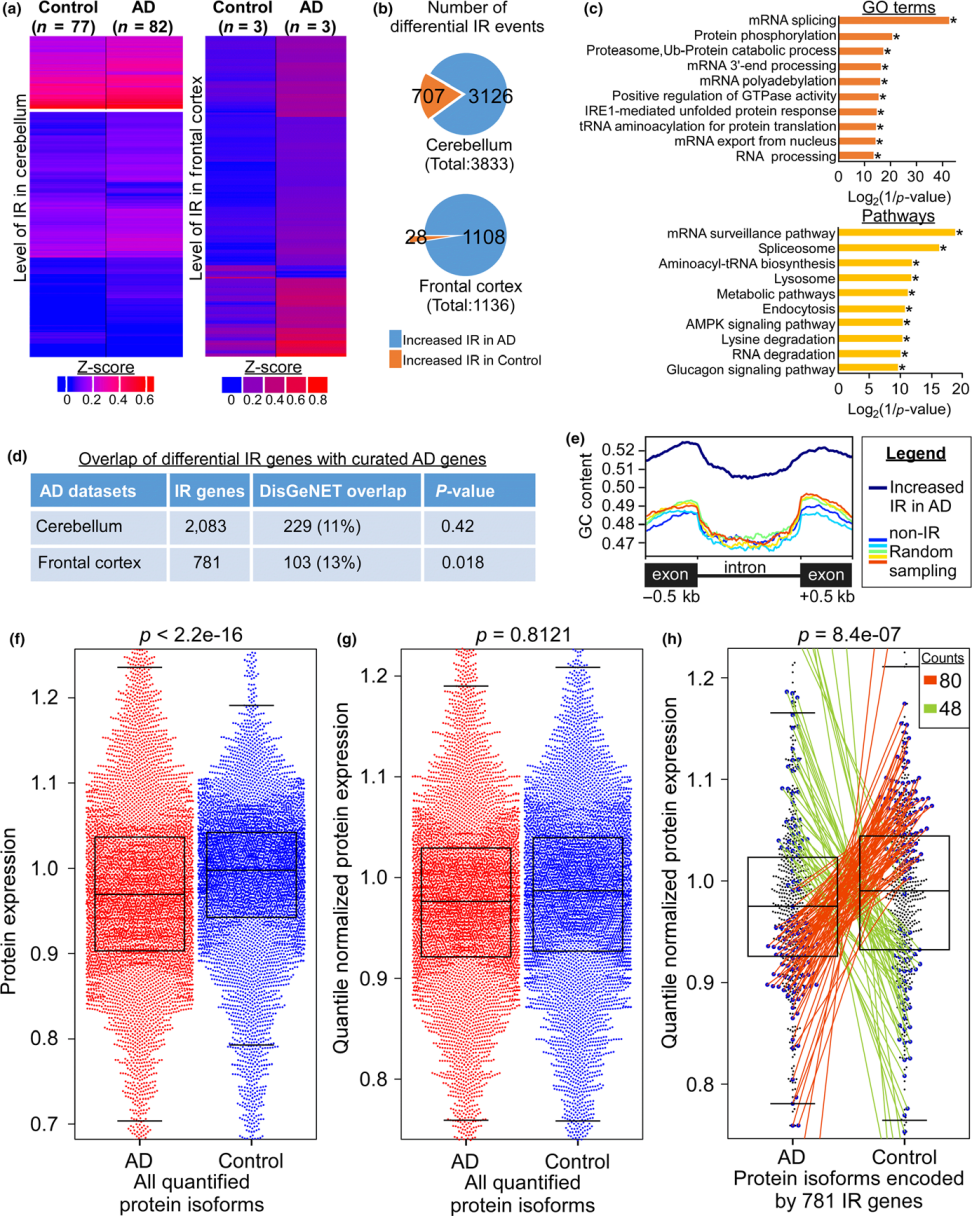
Increased number of IR events is observed in human AD brain tissues. (a) Expression heatmap of the differentially retained introns in the cerebellum (left, Mayo Clinic) and frontal cortex (right, University of Kentucky) from age‐matched control and AD subjects. (b) Pie diagrams indicating the number of increased IR events in cerebellum (top) and frontal cortex (bottom) of age‐matched control (orange) and AD patients (blue). (c) Gene ontology (*p‐value <0.05, Fisher's exact test with Benjamini–Hochberg correction) and pathway enrichment (*p‐value <0.05, hypergeometric test with FDR) analyses of the differential IR genes between control and AD cerebellum. (d) The overlap of differential IR genes identified in diseased brain tissues with curated AD genes from DisGeNET. The *p*‐value was determined by two‐tailed chi‐square test with Yates’ continuity correction. (e) GC content of the flanking exons and the differentially retained introns in AD frontal cortex. Five sets of non‐retained introns from genes with similar expression level were randomly picked as control. (f–g) Beeswarm boxplots illustrating the raw (f) and quantile normalized (g) expression of all 10,100 quantified protein isoforms in AD (red) and control (blue) frontal cortex. *p*‐value was calculated by Wilcoxon rank sum test with continuity correction. (h) The quantile normalized protein expression pattern of 781 differential IR genes is significantly different between AD and control frontal cortex (*p*‐value* *= 8.4e−07, Wilcoxon rank sum test with continuity correction). Blue dots represent genes whose protein level is significantly different between AD and control frontal cortex (*p*‐value <0.05, LIMMA *t *test). Among which, 73 (orange line) and 41 (green line) differential IR genes showed reduced and elevated protein level in AD frontal cortex, respectively

As aging is the major risk factor for sporadic AD, there is a likelihood that the increased IR events observed during aging may contribute to the etiology of AD and further changes in IR pattern may occur during disease manifestation. To test this possibility, we analyzed the cerebellum transcriptomes from Mayo Clinic Alzheimer's Disease Genetics Studies (https://www.synapse.org/#!Synapse:syn5049298). The downloaded datasets have 77 age‐match control subjects and 82 AD patients. There are altogether 3,833 differential IR events in 2083 genes between control and AD cerebellum (Figure 5a,b). Of which, there are 3,126 increased IR events (82%) at 1754 genes in AD cerebellum (Figure 5b; Supporting Information Table S7). GO and pathways analyses of these differential IR genes revealed representation of genes that are involved in regulating mRNA and protein homeostasis (*p‐*value <0.05, Fisher's exact test with Benjamini–Hochberg correction) (Figure 5c). To better understand how differential IR events may contribute to AD pathology, we further analyzed frontal cortex datasets where the transcriptome and global quantitative proteome are available (Bai et al., 2013; Ping et al., 2018). There were 1,136 differential IR events at 781 genes between the control and AD frontal cortex from University of Kentucky dataset (Figure 5a,b; Supporting Information Table S8). These differential IR genes are also highly enriched for biological functions that are associated with mRNA export and splicing (*p‐*value <0.05, Fisher's exact test with Benjamini–Hochberg correction) (Supporting Information Figure S5a). Similarly, there is an increase in IR events in AD frontal cortex from Emory University study where differential IR genes are enriched for mRNA export and metabolism of amino acids (Supporting Information Figure S5b and Table S9). Moreover, about 13% of these differential IR genes identified in the frontal cortex are overrepresented in the curated AD gene‐list from the DisGeNET database (*p* = 0.018, two‐tailed chi‐square with Yates’ correction) (Figure 5d; Supporting Information Table S8). Consistent with our other analyses, the length of differentially retained introns in AD frontal cortex is significantly shorter than non‐retained introns (Supporting Information Figure S5c,d, top panel). Also, differential IR has no significant effect on the level of mRNA expression (Supporting Information Figure S5c,d, bottom) and affects genes that are well distributed across the genome (Supporting Information Figure S5e). Similar to recent observation of higher CpG density at retained introns (Wong et al., 2017), we also observed elevated normalized GC content at retained introns when compared to spliced (non‐IR) introns (*p*‐value <0.05, Welch's *t* test) (Figure 5e). This suggests that changes in the level of DNA methylation during AD progression may contribute to differential IR.

As retained introns within mRNA transcript may introduce mutations or premature termination during translation, we aimed to determine whether the level of protein expression might be affected by differential IR. To this end, we examined a recent proteomic dataset generated from 10 control and AD frontal cortex (Ping et al., 2018). Beeswarm boxplot generated for all the 10,100 quantifiable protein isoforms showed that the overall distribution and the median expression levels of proteins are significantly lower in AD when compared to the control frontal cortex (*p* < 2.2e−16, Wilcoxon rank sum test with continuity correction) (Figure 5f and Supporting Information Figure S6a). As such, this dataset was subjected to quantile normalization to reduce technical variations and introduce similar statistical distribution in protein expression profiles between control and AD samples. We observed similar distribution in their expression pattern upon normalization (*p* = 0.8121) (Figure 5g and Supporting Information Figure S6b). Surprisingly, in spite of this drastic normalization, the overall protein expression pattern of the 781 differential IR genes remains significantly different between AD and control tissues (*p‐*value = 8.4e−07, Wilcoxon rank sum test with continuity correction) (Figure 5h). Moreover, 80 protein isoforms encoded by 73 differential IR genes have significant reduction in their expression (orange line, Figure 5h and Supporting Information Figure S6c), whereas 41 IR genes show increased protein level (48 protein isoforms, green line, Figure 5h and Supporting Information Figure S6c) in AD tissues when compared to control frontal cortex (*p* < 0.05, LIMMA *t *test) (Supporting Information Table S10). This suggests that differential IR at a subset of genes may contribute to the altered proteome observed in AD frontal cortex.

## 3 DISCUSSION

IR has been demonstrated to play important regulatory roles in differentiation and cellular plasticity by affecting the stability, nucleocytoplasmic transport, and translation of mRNA transcripts (Braunschweig et al., 2014; Buckley et al., 2011; Mauger et al., 2016; Wong et al., 2013). In line with the report in worms (Heintz et al., 2017), our study revealed a commonality in which there is an increase in IR events during aging in *Drosophila* heads, mouse, and human brain tissues. As dysregulated IR has been implicated in various human diseases (Wong et al., 2016), we extended our analyses to different AD cohorts and observed an increased number of retained introns in the diseased brain tissues. Taken together, our results suggest that increased IR may be a conserved post‐transcriptional signature of aging in different species and is likely to be associated with AD pathology.

Interestingly, genes with distinct biological functions are affected by differential IR at different stages of adult lifespan. This suggests that IR may have distinct roles in the regulating animal development and the process of aging. For instance, in mouse frontal cortex, there are an increased number of retained introns in the young 2‐week‐old mice which are subsequently lost in the older animals. Many of these genes were enriched for biological functions specific to brain development, cell cycles, and epigenetic regulation. Given the considerable level of postnatal brain development and maturation during this age, this result would be consistent with the reported roles of IR in regulating neurogenesis (Braunschweig et al., 2014; Buckley et al., 2011; Mauger et al., 2016). On the other hand, in both older and diseased mammalian brain tissues, genes with an increased level of retained introns are involved in regulating the homeostasis of mRNA and protein molecules. This would suggest that the altered IR patterns may contribute to the loss of proteostasis, which is a major hallmark of aging and AD pathology. The potential regulatory impact of IR on mRNA processing may also act as a feedback mechanism to further elevate the level of IR in older animals or AD brain. In the older day 50 fly heads, increased IR affected genes are involved in long‐term memory, determination of adult lifespan, and different aspects of muscle regulation. Given the tissue complexity of fly head and brain tissues, future studies would necessitate the purification of specific cell populations to better understand the regulatory roles of IR in aging.

In differentiating granulocytes and neurons, nascent mRNA transcripts of IR genes are not downregulated by NMD (Mauger et al., 2016; Wong et al., 2013). Instead, IR may regulate physiological functions through affecting the level of protein expression. Similar to other reports, the differentially retained introns identified in our aging and AD cohorts have shorter length when compared to the spliced introns and exert no impact on the expression level of their mRNA transcripts. Hence, it is likely that the physiological consequence of differential IR is manifested through affecting the integrity or expression level of the proteins. Consistent with this notion, retained intron at *HDAC4* gene introduces premature termination codon which leads to truncated protein in D50 flies (Supporting Information Figure S2). HDAC4, which deacetylates histones and nuclear factors, has been implicated in long‐term memory in flies (Schwartz, Truglio, Scott, & Fitzsimons, 2016). Further study would be necessary to establish whether the truncated HDAC4 protein may impact the memory of older flies. More importantly, analysis of quantitative proteome data revealed significant changes in the protein expression levels of more than one hundred differential IR genes between control and AD frontal cortex (Figure 5h and Supporting Information Figure S6c). However, the definitive causative effect of IR on protein expression in the context of aging and AD progression would require detailed molecular characterization of individual gene of interest in the specific cell populations.

What are the possible mechanisms that may regulate age‐ or AD‐dependent changes in IR patterns? Nucleosomes can act as potent barriers that inhibit RNA polymerase II elongation, which in turn promotes IR by allowing the recruitment of splicing repressive factors at exon–intron junction (Braunschweig et al., 2014). Interestingly, introns that are differentially retained during aging have higher level of nucleosome occupancy than the spliced introns in the younger flies (Figure 1e). This result suggests that factors involved in regulating nucleosome deposition at the introns in younger flies may determine the level of IR in older animals. Consistent with other studies (Braunschweig et al., 2014; Wong et al., 2017), the retained introns have a higher GC content than the spliced introns in AD tissues, suggesting that changes in the level of DNA methylation may affect the prevalence of IR in diseased brain tissues. Further studies to identify the mechanisms that led to changes in IR during aging and how it may promote the transition into the pathological state observed in AD would provide the framework for intervention against late‐onset AD.

## 4 EXPERIMENTAL PROCEDURES

A complete description of Materials and Methods can be found in Appendix S1.

### 4.1 RNA‐sequencing of male *Drosophila* heads

Total RNA was isolated from 100 male fly heads at the age of 10, 20, 30, and 50 days using RNAzol^®^RT (Sigma). Triplicates were prepared from each age‐group. RNA‐seq was conducted at Beijing Genomics Institute (BGI, Hong Kong) according to the company's protocol. RNA quality was assessed with Agilent 2100 bioanalyzer. Following DNase I treatment, poly (A) + enriched mRNA was fragmented and used as template for cDNA library construction. Short DNA fragments were then subjected to end‐repair, A‐tailing, adaptor ligation, size selection and sequenced on Illumina HiSeq4000. The Gene Expression Omnibus (GEO) accession ID of fly data is GSE110349.

### 4.2 Mammalian RNA‐seq datasets

The aging mouse and human brain transcriptomes (Lister et al., 2013; Mazin et al., 2014; Stilling et al., 2014) were downloaded from the NCBI's Sequence Read Archive (SRA) and GEO. The accession numbers are GSE47966 (mouse frontal cortex), GSE61915 (mouse hippocampus), and SRP005169 (human prefrontal cortex). The transcriptomes of AD cohorts were obtained from DDBJ SRA (SRS373308, SRS373257) (Bai et al., 2013) and AMP‐AD knowledge portal (syn8612213) (https://www.synapse.org/#!Synapse:syn5049298). Only poly (A) + enriched RNA‐sequencing datasets were analyzed as they were preferred in many other IR studies. Detailed descriptions of all datasets, including the tissue types, demographics of animals, and sequencing information, are summarized in Supporting Information Figure S1.

### 4.3 Data processing and identification of differential IR events

FastQC (https://www.bioinformatics.babraham.ac.uk/projects/fastqc/) was used to assess the quality of RNA‐seq data. Adaptor sequences were identified by AdaptorDetect and removed using trim functional module within the IRFinder pipeline (Middleton et al., 2017). Sequences from different species were aligned to *Drosophila *(BDGP6.84), mouse (GRCm38.87), or human (GRCh38.87) reference genome accordingly using STAR v2.5 (Dobin et al., 2013) with the following arguments: ‐‐outSAMtype BAM SortedByCoordinate ‐‐outFilterMultimapNmax 1 ‐‐outSAMstrandField intronMotif ‐‐outSAMunmapped None. The sequencing statistics from *Drosophila *dataset are summarized in Supporting Information Table [Link], [Link].

IRFinder algorithm was used to detect and quantify IR events in all known introns (Middleton et al., 2017). IRFinder estimates the level of IR by calculating the ratio of intronic abundance to the sum of intronic and exonic splice abundance. The calculated metric is specified as IR ratio (Middleton et al., 2017; Schmitz et al., 2017; Wong et al., 2017) or percent IR (PIR) in other studies (Braunschweig et al., 2014; Jacob & Smith, 2017; Mauger et al., 2016). IR ratio ranges from 0 to 1 and measures the proportion of total RNA transcripts that retained the given intron. Introns retained in at least 10% of the transcripts (IR ratio >0.1 in at least one sample group) with a minimal splicing sequencing depth of greater than five reads were used for further analysis. Differential IR events were calculated through pairwise comparison of different age‐groups within fly, mouse, and human datasets, and between AD patients and control subjects. DESeq2 or Bayesian statistic was applied to identify changes in IR between different samples (Audic & Claverie, 1997; Love, Huber, & Anders, 2014). In accordance with another study (Wong et al., 2017), significance of differential IR event was considered with a normal *p*‐value <0.05. A heatmap of *Z*‐scores of IR ratios for the differentially retained introns was plotted using the complex heatmap function in R Bioconductor. (Gu, Eils, & Schlesner, 2016).

### 4.4 Mouse orthologues of Drosophila IR genes

DIOPT‐DRSC Integrative Orthologue Prediction Tool (Hu et al., 2011) was used to identify highly conserved human orthologous counterparts of the differential IR genes identified in *Drosophila* and mouse; as well as the conserved *Drosophila* and mouse genes that have age‐dependent differential IR events.

### 4.5 Quantification of gene expression

The relative expression level of genes with differential IR events was determined by Cufflinks and specified as fragments per kilobase per million (FPKM) (Trapnell et al., 2012).

### 4.6 Characterization of genetic features of differentially retained introns

To analyze the length and GC content of the differentially retained introns, equal numbers of randomly picked spliced introns of similar expression were used as references. The boxplot for the intron length was generated using ggplot with the statistical significance determined by Welch's two‐sample *t *test function from R. To measure the GC content, equally sized 500 base pairs windows were defined across the human reference genome (GRCh38.87) using the BEDtools makewindows function (Quinlan & Hall, 2010). The nucleotide base frequencies across all windows were then calculated with BEDtools nuc. The guanine and cytosine nucleotide frequency profiles for flanking exons and the introns differentially retained between AD and control frontal cortex were plotted using deepTools (Ramírez, Dündar, Diehl, Grüning, & Manke, 2014) with spliced introns as reference. The genomic distribution of differential IR genes was illustrated by karyotype plot using the R karyoploteR library (Gel & Serra, 2017).

### 4.7 Characterization of epigenetic features associated with differentially retained introns

#### 4.7.1 Processing of Drosophila MNase‐seq data

The raw MNase‐seq reads were mapped to *Drosophila* reference genome (BDGP6.84) using STAR v2.5 (Dobin et al., 2013) with the arguments: ‐‐alignEndsType EndToEnd ‐‐alignIntronMax 1 ‐‐outFilterMultimapNmax 1. PCR duplicates were removed using Picard (MarkDuplicates) (http://picard.sourceforge.net/). Biological replicates were pooled, and average coverage over the intronic regions was calculated using ComputeMatrix from Deeptools (Ramírez et al., 2014). Wilcoxon rank sum test with continuity correction was used to determine the statistical significance of the difference in MNase‐seq signals between the retained and spliced introns.

### 4.8 Processing of published human proteomic data

The AD proteomic dataset generated by tandem mass tag (TMT) isobaric labeling and synchronous precursor selection‐based MS3 (SPS‐MS3) mass spectrometry was downloaded from the Synapse portal (syn10239444) (Ping et al., 2018). In this study, 10 control and 10 AD postmortem frontal cortex were analyzed in five batches to generate a list of 10,100 protein groups (represented by 9,028 genes). Protein groups represent the different protein isoforms encoded by the same gene. Beeswarm boxplots were generated using the R beeswarm package to graphically denote the expression of individual protein group in control and AD tissues. To reduce systemic bias caused by technical variations and introduce similar distribution pattern in protein expression between control and AD samples, the dataset was rescaled with quantile normalization using quantile function in R. Nonparametric Wilcoxon test was used to determine whether the overall protein expression pattern is significantly different between control and AD tissues. LIMMA from the R Bioconductor package was next used to identify differential IR genes whose normalized protein expression is significantly different (*p*‐value <0.05, LIMMA *t *test) between control and AD frontal cortex.

### 4.9 Gene ontology, pathway analysis, and disease association study

Gene ontology analysis was performed on the differential IR genes using the Database for Annotation, Visualization and Integrated Discovery (Huang, Sherman, & Lempicki, 2009). The significantly enriched biological processes were determined by Fisher's exact test (*p < *0.05, Fisher's exact/EASE score) and then plotted using dotplot from DOSE‐ClusterProfiler (R Bioconductor package) (Yu, Wang, Han, & He, 2012). Multiple testing correction was determined by Benjamini–Hochberg procedure (*p*‐value <0.05), and all significant terms were denoted by *. The KEGG and PANTHER databases from WebGESTALT were used to perform pathway enrichment analysis of the differential IR genes (Wang, Duncan, Shi, & Zhang, 2013). Pathways with *p*‐value <0.05 (using hypergeometric test) were considered significant. Association with AD was determined by overlapping genes with differential IR (from aging and AD datasets) with the AD‐curated gene‐list from DisGeNET v5.0 (Piñero et al., 2017). *p*‐value was calculated by two‐tailed chi‐square test with Yates’ continuity correction.

## CONFLICT OF INTEREST

None declared.

## AUTHORS’ CONTRIBUTIONS

S.A., T.B., and O.C.T. conceived and designed this study. S.A. contributed to data collection, processing, and analyses. S.A., T.B., and O.C.T. interpreted the results. Z.K.N. contributed to experimental validation of IR, Western analysis, and MNase‐seq*. *W.Q.L. processed the RNA samples from *Drosophila*. S.A. and O.C.T. wrote the manuscript. S.A., T.B., and O.C.T. have reviewed and approved the final version of the manuscript.
