# Supplementary material for: Increased intron retention is a post‐transcriptional signature associated with progressive aging and Alzheimer’s disease
Source: Aging Cell. 2019 Mar 13;18(3):e12928. doi: 10.1111/acel.12928 (PMC6516162; doi:10.1111/acel.12928)
Supplement: Supplementary file 1 [file ACEL-18-e12928-s001.docx]

**Appendix S1**

**Increased intron retention is a post-transcriptional signature associated with progressive aging and Alzheimer’s disease**

Swarnaseetha Adusumalli, Zhen-Kai Ngian, Wei-Qi Lin, Touati Benoukraf, Chin-Tong Ong

**Supplementary Figures**

**
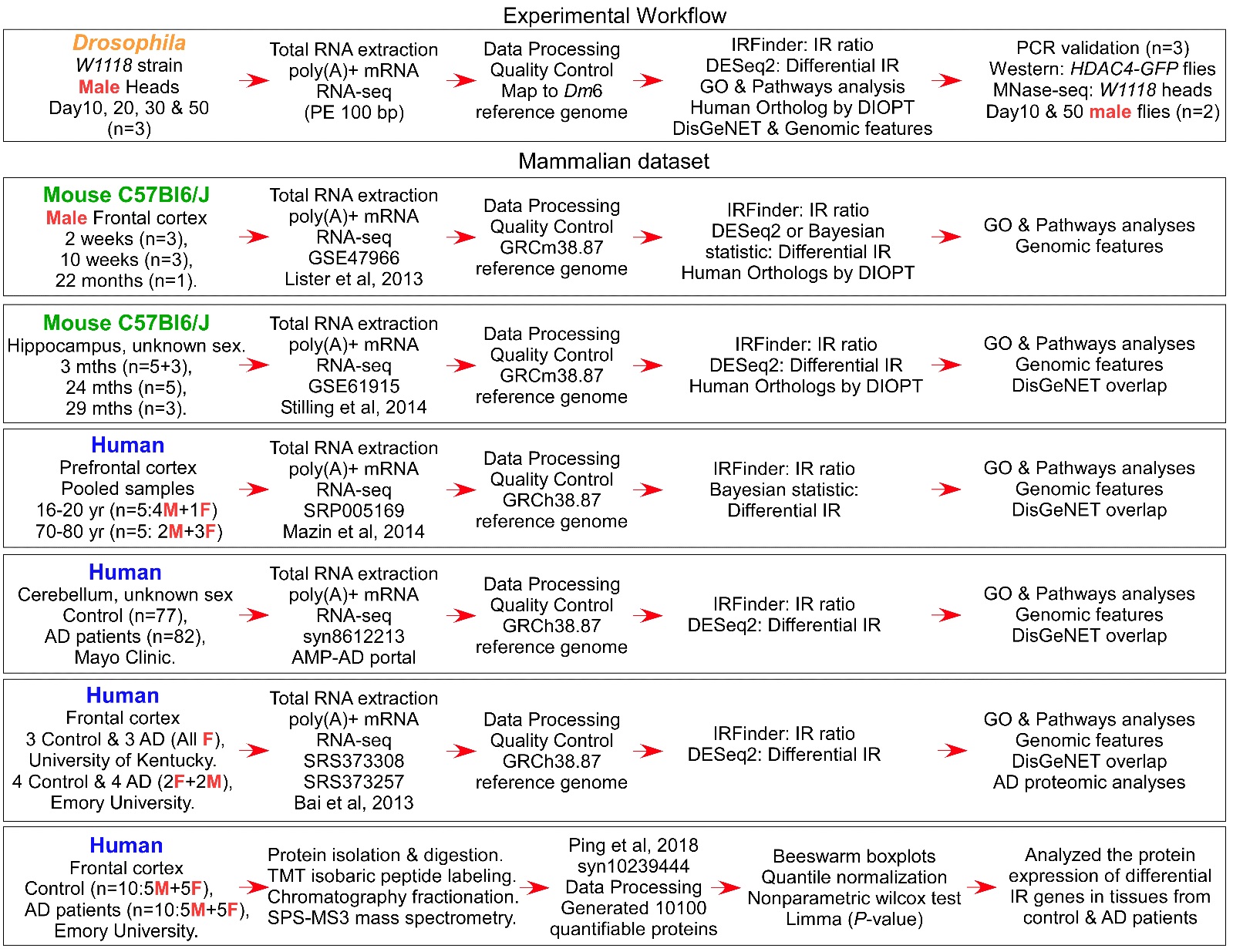
**

**Figure S1 Workflow of *Drosophila* experiment and dataset analyzed in this study**

**
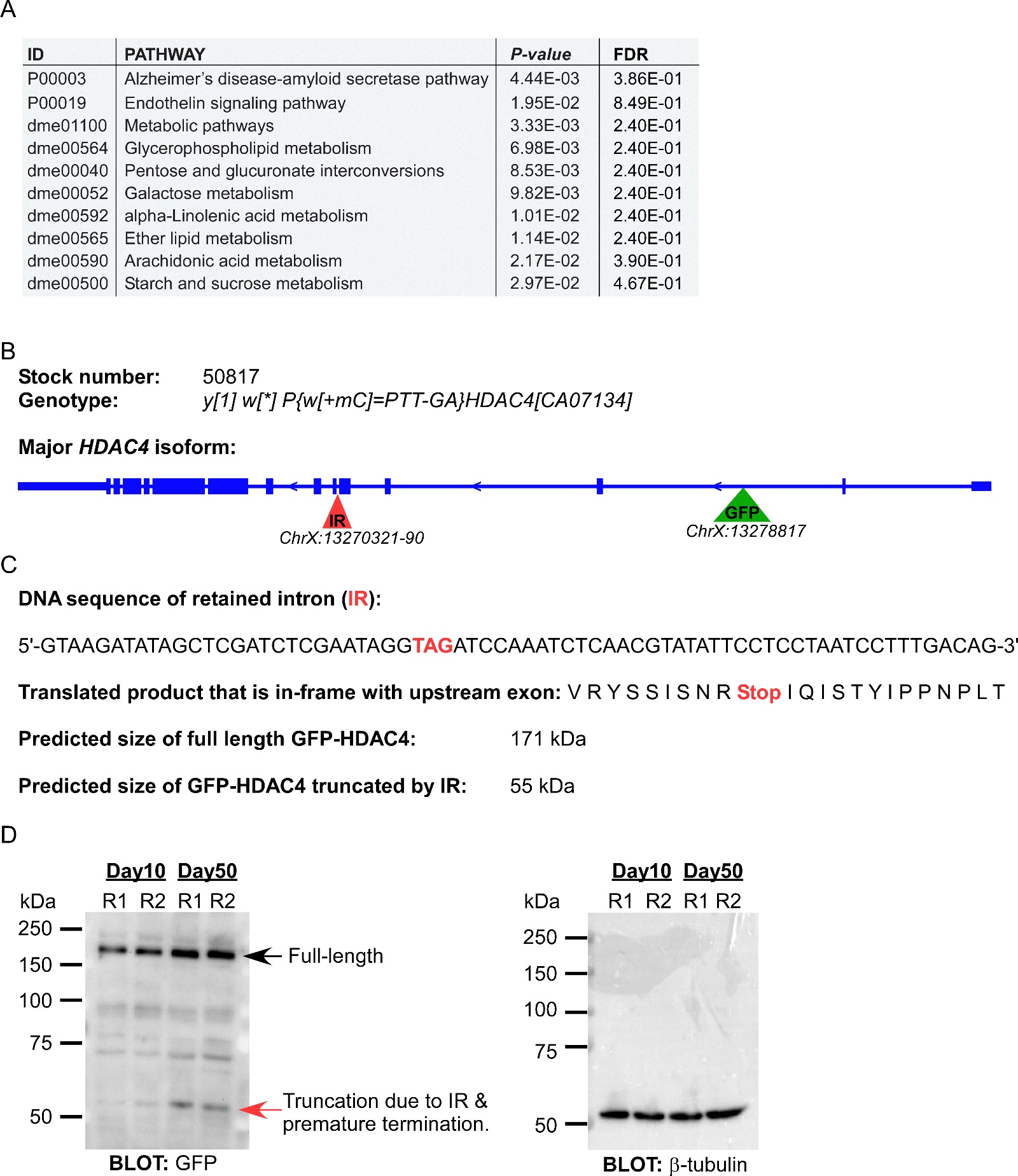
**

**Figure S2 IR at *HDAC4* gene leads to premature termination during translation**

**A.** Pathway enrichment analysis of fly differential IR genes (*p* < 0.05, hypergeometric test). **B.** Organization of the major isoform of *HDAC4* gene showing the position of retained intron (red) and the integration site of GFP transgene (green). **C.** The retained intron introduces premature termination codon that leads to the translation of truncated GFP-HDAC4 protein. **D.** Western blot of total head lysates isolated from two biological replicates (R1 and R2) of Day10 and Day 50 animals. Left panel: Western membrane probed with anti-GFP antibody. Right panel: Membrane probed with anti-β-tubulin antibody as loading control.

**
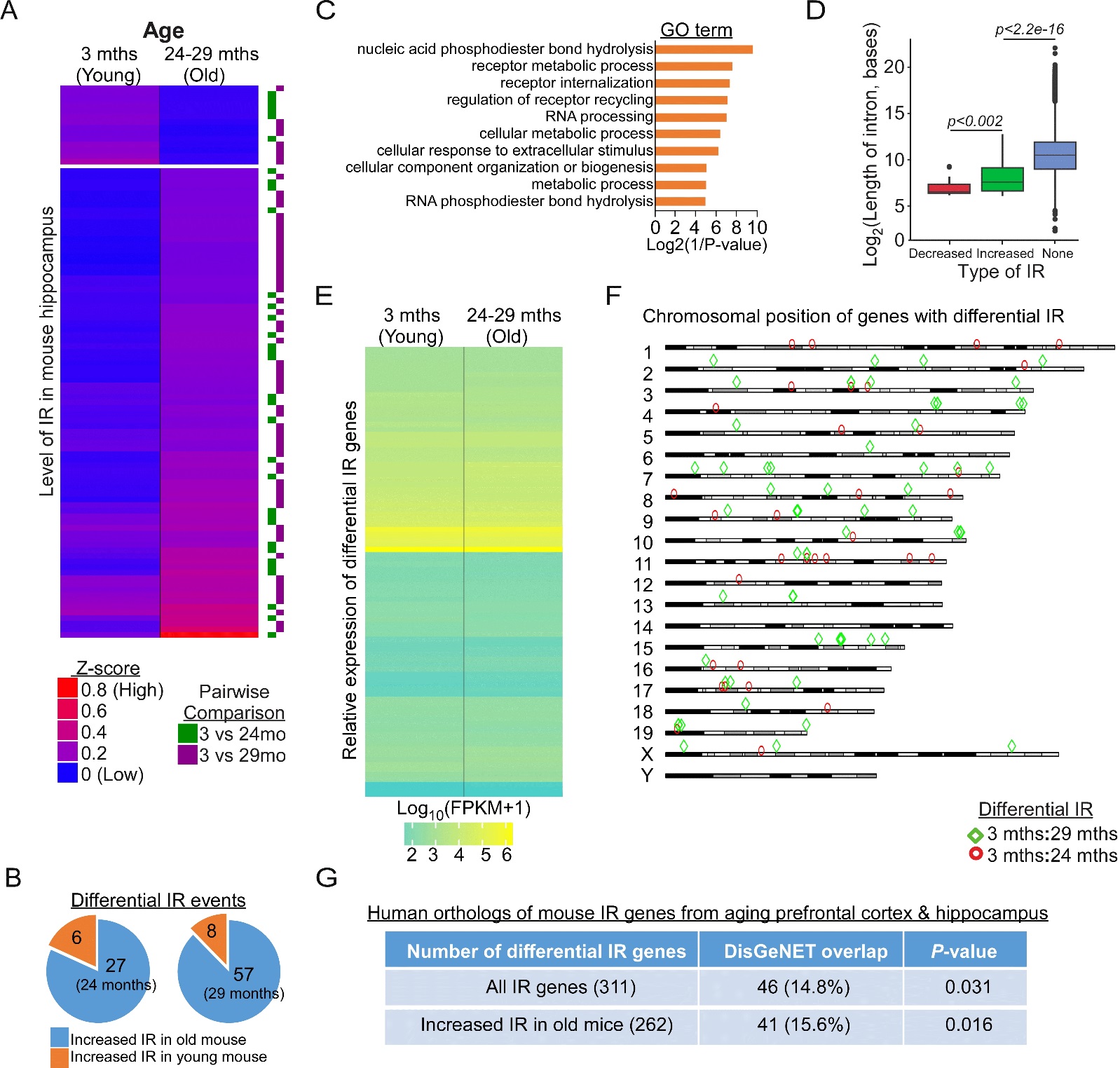
**

**Figure S3 Differential IR in aging mouse hippocampus**

**A.** Expression heatmap of differentially retained introns between young (3 months, n=5+3) and old (24 months, n=5. 29 months, n=3) mouse hippocampus. **B.** Pie diagrams showing the number of increased IR events between young (3 months) and old (24 and 29 months) mouse hippocampus. **C.** Gene ontology analysis of the genes with differential IR in aging mouse hippocampus (*p* < 0.05, Fisher exact test). **D.** Boxplot showing the length distribution of different types of introns with *p*-value calculated by Welch’s *t*-test. The expression level of intron at 3 months was used as the reference for calculating decreased or increased IR. “None” refers to non-retained intron. **E**. Relative expression of genes with differential IR between young and aged mouse hippocampus as represented by Log_10_ (FPKM+1) values. **F.** Ideogram displaying the distribution of the differential IR genes across the mouse genome. **G.** The overlap between human orthologs of mouse differential IR genes identified in aging brain tissues and curated AD genes from DisGeNET. The *p*-value was determined by two-tailed Chi-square test with Yates’ continuity correction.

**
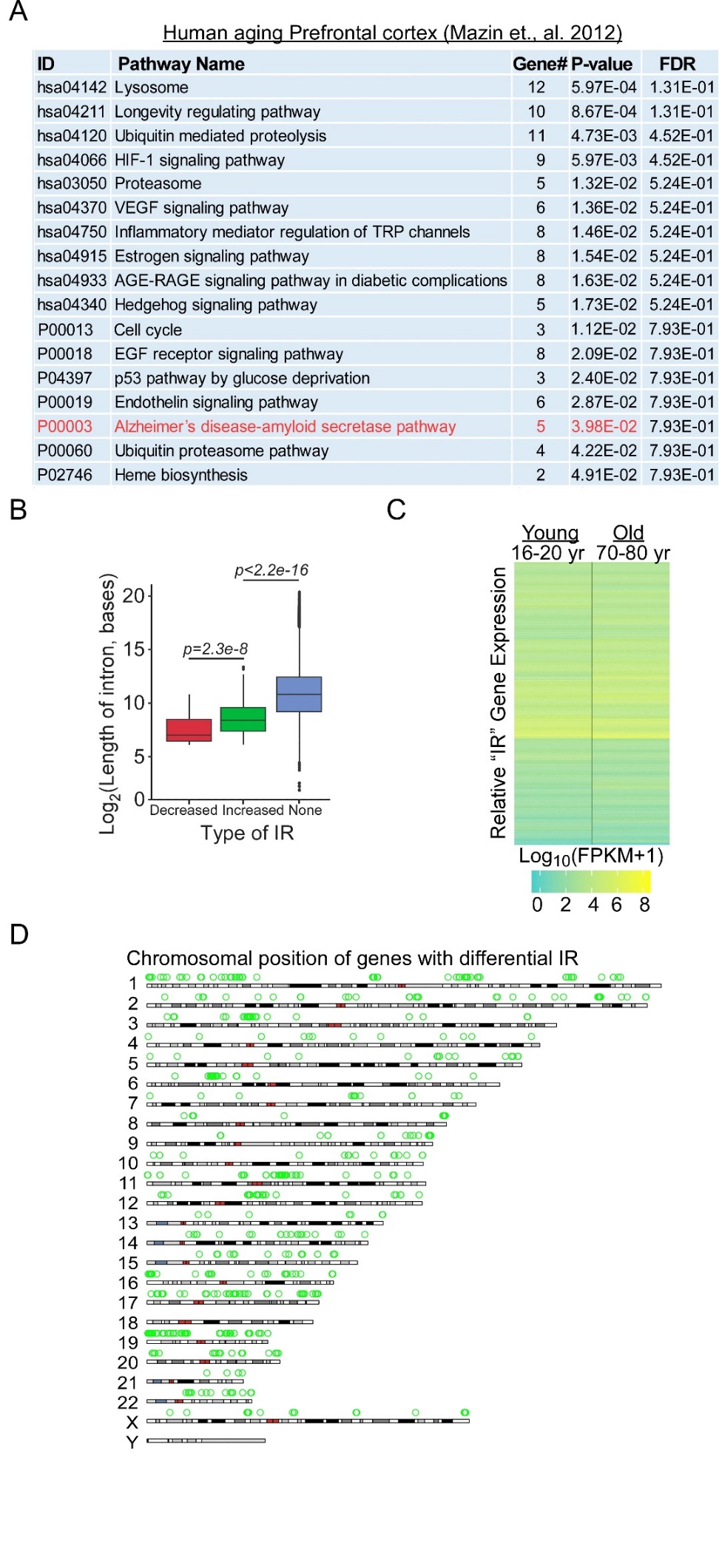
**

**Figure S4** **Characterization of differential IR genes in aging human prefrontal cortex**

**A.** Pathways enrichment analysis of genes that have increased IR in old PFC was performed with WebGestalt using KEGG and PANTHER databases. **B.** Boxplot showing the length distribution of different types of introns with *p*-value calculated by Welch’s *t*-test. The expression level of intron in young (16-20yr) prefrontal cortex was used as the reference for calculating decreased or increased IR. “None” refers to non-retained intron. **C.** Relative expression of IR genes between young and old human prefrontal cortex as represented by Log_10_ (FPKM+1) values. **D.** Ideogram displaying the distribution of the genes with age-dependent differential IR (green circle) across the human genome.

**
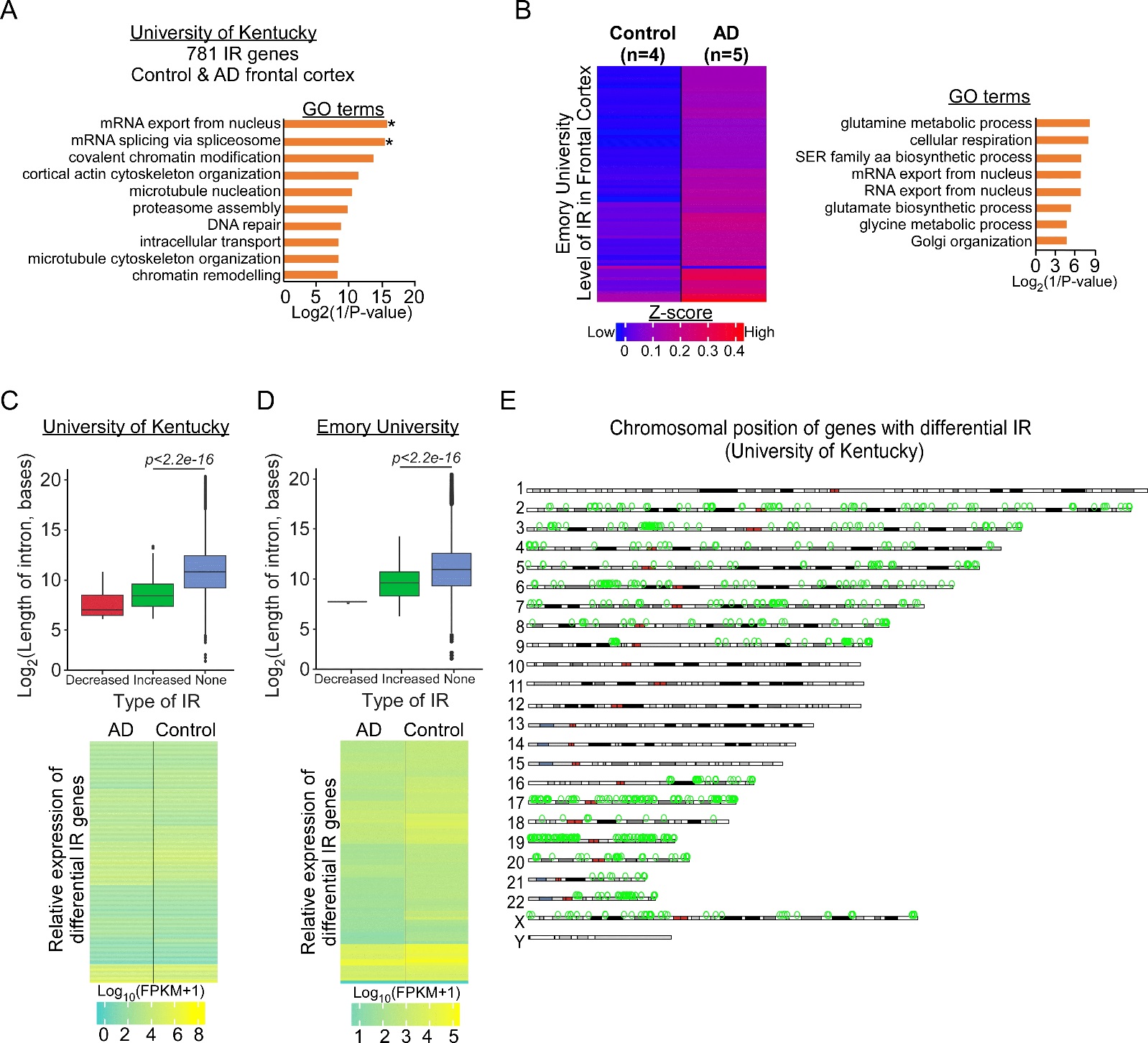
**

**Figure S5 Characterization of differential IR genes between control and AD subjects**

**A.** Gene ontology analysis of the genes with differential IR in aging mouse hippocampus (*p* < 0.05, Fisher exact test. *: *p*-value < 0.05, Fisher exact test with Benjamini-Hochberg correction). **B.** Expression heatmap of differentially retained introns in the frontal cortex from aged-matched control and AD patients (Emory University dataset). **C.** Gene ontology analysis of the differential IR genes from Emory University dataset (*p* < 0.05, Fisher exact text). **D-E.** Top panel: Boxplots showing the length distribution of introns with either significant decrease or increase in the level of IR in AD tissues versus the non-retained introns from University of Kentucky and Emory University cohorts. Bottom panel: Relative expression of differential IR genes between age-matched control and AD patients as represented by Log_10_ (FPKM+1) values from the two dataset. **F.** Ideogram displaying the distribution of the AD-related differential IR genes (green circle) across human genome (University of Kentucky).

**
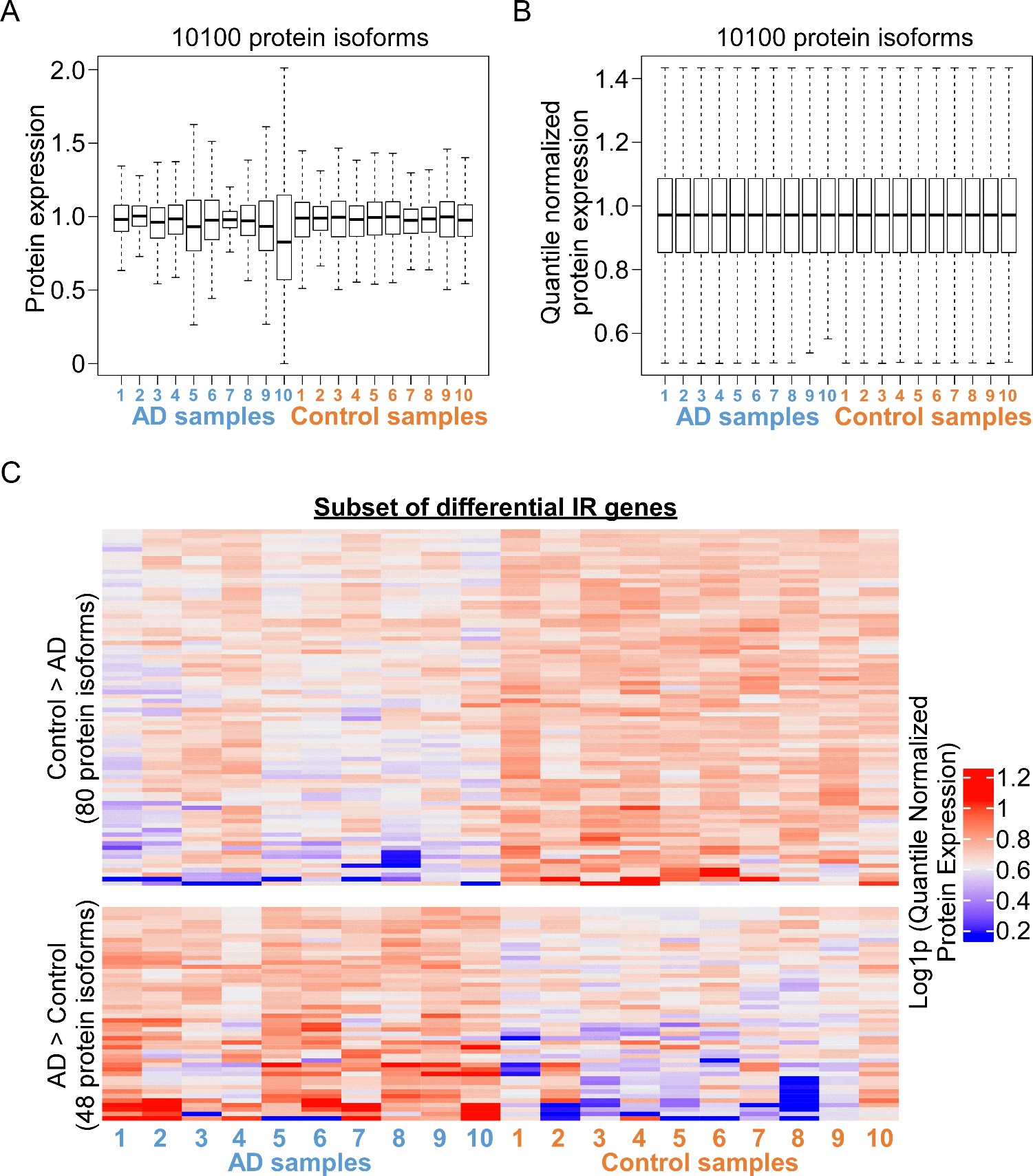
**

**Figure S6 Proteomic analysis of frontal cortex of age-matched control and AD subjects**

**A-B.** Boxplots illustrating the raw **(A)** and quantile normalized **(B)** expression of all 10100 quantified protein isoforms from individual AD (light blue) and control (orange) frontal cortex. **C.** A subset of differential IR genes whose protein expression is significantly different between AD and control samples (*p* < 0.05, Limma t-test). Each row represents the expression from unique protein isoform. Relative protein expression across 10 AD and control samples was represented by Log1p (Quantile normalized protein expression) values. There are 80 protein isoforms (encoded by 73 IR genes) whose expression is lower in AD samples and 48 protein isoforms (encoded by 41 IR genes) whose expression is lower in control samples.

**Supplementary Experimental Procedures**

**Fly husbandry**

*Drosophila melanogaster* strain *W^1118^* were cultured in standard fly media (6% cornmeal, 5% dextrose, 2.4% brewer’s yeast, 0.8% agarose and 0.3% NIPAGIN) at 25^◦^C with 12-hour light/dark cycle. Twenty male flies were housed in one vial (diameter of 2.5 cm and height of 9.5 cm) and the food was changed every two days until the desired age. To minimize the effect of circadian rhythm, fly heads were harvested from 2 to 4 pm. Protein trap line 50817 (Bloomington *Drosophila* stock center) contained green fluorescent protein (GFP) inserted in *HDAC4* gene and was used to evaluate the changes in the translation of HDAC4 protein during aging.

**Western analysis**

Male heads harvested from Day 10 and Day 50 protein trap line 50817 were lysed directly in 1 x Laemmli buffer and boiled for 10mins. Protein lysate was resolved in 8% SDS-PAGE gel and subjected to standard western transfer protocol. The transferred PVDF membrane was blocked in 5% milk (PBSTween 0.1%) and then probed with anti-GFP (ab290, Abcam, 1:6000) and β-tubulin (E7, DSHB, 1:3000) antibodies.

**Characterization of epigenetic features associated with differentially retained introns**

*Micrococcal nuclease (MNase) preparation*

50 mg of frozen D10 or D50 flies heads were transferred to Dounce homogenizer (Sigma, D9938) containing 5 ml of ice-cold A1 cell lysis buffer [60mM KCL, 15mM NaCl, 4mM MgCl2, 15mM HEPES pH7.6, 0.5% NP-40, 0.5mM DTT, protease inhibitor (Leupeptin, Pepstatin A, Aprotinin, 0.5mM PMSF, 1X Roche proteinase inhibitor cocktail)] and mechanically disrupted 30 strokes using pestle A. The lysate was incubated 15 min on ice and filtered through mira cloth to remove tissue debris. Filtrate was centrifuged at 4°C at 4000g for 5 min to remove cytoplasmic supernatant. The nuclear pellet was subjected to three additional rounds of A1 washes (3 ml of buffer/wash). After the final wash, nuclear pellet was resuspended in 3.3 ml of RIPA buffer [10mM Tris pH 8, 1mM EDTA, 1% Triton, 140mM NaCl, 0.1% SDS, 0.1% sodium deoxycholate, 0.5mM DTT, protease inhibitor (same as above)] and dounced with 30 strokes using pestle B (Sigma, D8938). 3.3 ul of 3M CaCl2 was then added to the sample and 50 ul was aliquoted as uncut nuclear input fraction. The remaining fraction was incubated with 15ul of MNase (Worthington Biochemical, 25U/ul) and incubated at 37°C for 15 min, with agitation every 5 min. 90 ul STOP buffer (0.5M EDTA:0.5M EGTA in 1:2 ratio) was added for every 1.5 ml of reaction to inhibit MNase activity. Sample was kept on ice and sonicated (Ultrasonic Processor, Vibra-Cell) for 5 x 10 seconds with a power output of 6 to release the chromatin from nuclear matrix. Samples were then centrifuged at maximum speed and the resultant supernatant was collected as chromatin extract. DNA was purified and used for library construction using standard Illumina protocol.

**Experimental validation and quantification of IR events in aging male fly heads**

The retained intron was visualized on Integrative Genomic Viewer and the genomic coordinates were determined using decorated region view in Flybase. DNA sequences was extracted from UCSC genome browser and used as template for primer design with Primer3 software. Complementary DNA (cDNA) was synthesized from total RNA using high capacity cDNA reverse transcription kit (Applied Biosystems) and quantified with Qubit^TM^ dsDNA HS Assay (Invitrogen). Equal amount of cDNA (50-100 ng) from each time-point was used for PCR.

Primer set 1, which hybridizes the flanking exons, was used in end-point PCR on C1000 Touch^TM^ Thermal Cycler (Biorad) to illustrate both retained and spliced transcripts (Fig.2A). The annealing temperature (50^◦^C to 60^◦^C) and amplification cycle (35 to 40 cycles) were optimized individually for each gene. The PCR amplicons were resolved on a 2.5% agarose gel.

Primer set 2, which recognizes the exon-intron junction and the retained intron, can only amplify transcripts that have undergone IR (Fig.2A). Three biological samples (n=3) were used for each age group. Real-time quantitative PCR for each biological sample was conducted in triplicates using 7900HT Fast Real-Time PCR System (ABI). The relative expression level of retained intron was calculated by 2^-ΔCt method where *β-Actin* expression was used as the internal control. Therefore, ΔCt= Ct value of *test retained intron* minus Ct value of *β-Actin*. The statistical significance of the difference between D10 and D50 was calculated by paired *t*-test of the relative expression level of retained introns of three biological replicates. Primer sequences are listed in Table S11.

**Additional Acknowledgment**

T.B. is supported by the RNA Biology Center at the Cancer Science Institute of Singapore, NUS, as part of funding under the Singapore Ministry of Education’s AcRF Tier 3 grants, grant number MOE2014-T3-1-006, the National Research Foundation and the Singapore Ministry of Education under its Centre of Excellence initiative. We thanked Mathanapriya Naidu for copy editing the manuscript.

Study data (syn8612213) were provided by the following sources: The Mayo Clinic Alzheimer’s Disease Genetic Studies, led by Dr. Nilufer Taner and Dr. Steven G. Younkin, Mayo Clinic, Jacksonville, FL using samples from the Mayo Clinic Study of Aging, the Mayo Clinic Alzheimer’s Disease Research Center, and the Mayo Clinic Brain Bank. Data collection was supported through funding by NIA grants P50 AG016574, R01 AG032990, U01 AG046139, R01 AG018023, U01 AG006576, U01 AG006786, R01 AG025711, R01 AG017216, R01 AG003949, NINDS grant R01 NS080820, CurePSP Foundation, and support from Mayo Foundation. Study data includes samples collected through the Sun Health Research Institute Brain and Body Donation Program of Sun City, Arizona. The Brain and Body Donation Program is supported by the National Institute of Neurological Disorders and Stroke (U24 NS072026 National Brain and Tissue Resource for Parkinson’s Disease and Related Disorders), the National Institute on Aging (P30 AG19610 Arizona Alzheimer’s Disease Core Center), the Arizona Department of Health Services (contract 211002, Arizona Alzheimer’s Research Center), the Arizona Biomedical Research Commission (contracts 4001, 0011, 05-901 and 1001 to the Arizona Parkinson's Disease Consortium) and the Michael J. Fox Foundation for Parkinson’s Research.

**Conflict of interest**

The authors have no conflict of interest to declare.
