# Supplementary material for: Increased intron retention is a post‐transcriptional signature associated with progressive aging and Alzheimer’s disease
Source: Aging Cell. 2019 Mar 13;18(3):e12928. doi: 10.1111/acel.12928 (PMC6516162; doi:10.1111/acel.12928)
Supplement: Supplementary file 2 [file ACEL-18-e12928-s002.pdf]

Table S1: DAVID functional annotation chart of differential IR genes identified in the head tissues during aging of male *Drosophila*

GO ID	GOTERM_BP_DIRECT	Count	%	*P* -value	Fold Enrichment	Benjamini value	Time Point
GO:0006470	protein dephosphorylation	4	4.598	0.026034496	6.170594837	0.999829848	D10_vs._D20
GO:0008360	regulation of cell shape	4	4.598	0.030280902	5.817989418	0.993642907	
GO:0007268	chemical synaptic transmission	5	4.762	0.002054225	9.154179154	0.504015851	D10_vs._D30
GO:1903432	regulation of TORC1 signaling	2	1.905	0.024356156	80.55677656	0.985066276	
GO:0035071	salivary gland cell autophagic cell death	4	3.810	0.025961211	6.19667512	0.949707797	
GO:0045214	sarcomere organization	10	3.484	9.63E-09	15.14600551	6.50E-06	D10_vs._D50
GO:0006874	cellular calcium ion homeostasis	6	2.091	1.22E-05	18.17520661	0.004100704	
GO:0007498	mesoderm development	9	3.136	7.28E-05	6.389721074	0.016236766	
GO:0030239	myofibril assembly	5	1.742	2.54E-04	15.14600551	0.041958111	
GO:0006936	muscle contraction	4	1.394	3.41E-04	25.96458087	0.044994846	
GO:0007517	muscle organ development	8	2.787	0.002246202	4.379567858	0.223518112	
GO:0060047	heart contraction	3	1.045	0.004579433	27.26280992	0.357637458	
GO:0055085	transmembrane transport	15	5.226	0.004745326	2.350242234	0.330577506	
GO:0035556	intracellular signal transduction	8	2.787	0.006684952	3.599050814	0.395319322	
GO:0008340	determination of adult lifespan	10	3.484	0.012544767	2.672824502	0.573493602	
GO:0072347	response to anesthetic	3	1.045	0.015558164	15.14600551	0.617952437	
GO:0007616	long-term memory	6	2.091	0.020665531	3.786501377	0.691062224	
GO:0006468	protein phosphorylation	12	4.181	0.032489639	2.049835332	0.820031749	
GO:0031122	cytoplasmic microtubule organization	4	1.394	0.034990849	5.507638367	0.820446437	
GO:0007220	Notch receptor processing	3	1.045	0.041634931	9.087603306	0.852465389	
GO:0016059	deactivation of rhodopsin mediated signaling	3	1.045	0.041634931	9.087603306	0.852465389	
GO:0007525	somatic muscle development	4	1.394	0.046779008	4.912218003	0.867495719	
GO:0007527	adult somatic muscle development	3	1.045	0.046909357	8.519628099	0.851575622	
GO:0008582	regulation of synaptic growth	4	1.394	0.049994815	4.782949108	0.853875147	

Table S1: Highly conserved human orthologs of fly differential IR genes

S/No	Fly GeneID	Fly Symbol	HGNCID	Human Symbol	DIOPT Score	Weighted Score	Rank
1	FBgn0000228	Bsg25D	14906	NIN	7	6.83	high
2	FBgn0000316	cin	15465	GPHN	14	13.74	high
3	FBgn0000319	Chc	2092	CLTC	14	13.8	high
4	FBgn0000464	Lar	9668	PTPRD	14	13.8	high
5	FBgn0000482	dor	15972	VPS18	15	14.75	high
6	FBgn0000667	Actn	164	ACTN2	14	13.75	high
7	FBgn0001202	hook	23576	HOOK3	15	14.75	high
8	FBgn0002431	hyd	16806	UBR5	15	14.75	high
9	FBgn0002566	lt	12713	VPS41	13	12.79	high
10	FBgn0002643	mam	13632	MAML1	2	2	high
11	FBgn0002774	mle	2750	DHX9	15	14.75	high
12	FBgn0002891	mus205	9968	REV3L	12	11.73	high
13	FBgn0003011	ort	4329	***GLRB***	5	4.84	high
14	FBgn0003016	osp	30321	MPRIP	9	8.88	high
15	FBgn0003068	per	8847	PER3	6	6.01	high
16	FBgn0003071	Pfk	8877	PFKM	14	13.82	high
17	FBgn0003141	pr	9689	PTS	14	13.85	high
18	FBgn0003218	rdgB	21044	PITPNM2	14	13.8	high
19	FBgn0003656	sws	24768	PNPLA7	14	13.8	high
20	FBgn0003744	trc	17847	STK38	14	13.8	high
21	FBgn0003748	Treh	12266	TREH	14	13.8	high
22	FBgn0004028	wupA	11946	TNNI2	10	9.96	high
23	FBgn0004103	Pp1-87B	9281	PPP1CA	13	12.75	high
24	FBgn0004169	up	11949	TNNT2	9	8.77	high
25	FBgn0004369	Ptp99A	9671	PTPRG	9	8.93	high
26	FBgn0004507	GlyP	9726	PYGM	15	14.75	high
27	FBgn0004598	Fur2	8747	PCSK5	10	9.76	high
28	FBgn0004698	Xpc	12816	XPC	11	10.69	high
29	FBgn0004795	retn	3031	ARID3A	11	10.83	high
30	FBgn0004797	mdy	2843	DGAT1	13	12.77	high
31	FBgn0004914	Hnf4	5026	HNF4G	13	12.74	high
32	FBgn0004919	gol	23138	RNF150	12	11.73	high
33	FBgn0005666	bt	12403	TTN	9	8.86	high
34	FBgn0010051	Itp-r83A	6180	ITPR1	15	14.75	high
35	FBgn0010235	Klc	6387	KLC1	13	12.87	high
36	FBgn0010278	Ssrp	11327	SSRP1	14	13.72	high
37	FBgn0010434	cora	3379	EPB41L2	8	7.87	high
	FBgn0010434	cora	3378	*EPB41L1*	8	7.78	high
	FBgn0010434	cora	3380	*EPB41L3*	8	7.77	high
38	FBgn0010438	mtSSB	11317	SSBP1	15	14.75	high
39	FBgn0010551	Phb2	30306	PHB2	14	13.8	high
40	FBgn0010750	atms	25459	PAF1	14	13.8	high
41	FBgn0010905	Spn	14946	PPP1R9A	9	8.78	high
42	FBgn0011206	bol	14273	BOLL	11	10.78	high
43	FBgn0011227	ox	30863	UQCR10	11	10.89	high
44	FBgn0011286	RyR	10484	RYR2	14	13.8	high
	FBgn0011286	RyR	10483	*RYR1*	14	13.75	high

S/No	Fly GeneID	Fly Symbol	HGNCID	Human Symbol	DIOPT Score	Weighted Score	Rank
45	FBgn0011296	l(2)efl	2389	CRYAB	12	11.95	high
46	FBgn0011455	ND-SGDH	7700	NDUFB5	13	12.73	high
47	FBgn0011745	Arp1	167	ACTR1A	14	13.8	high
48	FBgn0013762	Cdk5	1774	CDK5	14	13.8	high
49	FBgn0013972	Gycalpha99B	4684	GUCY1A2	13	12.8	high
50	FBgn0014863	Mlp84B	2472	CSRP3	10	9.68	high
51	FBgn0014906	Hydr2	18717	ABHD2	14	13.8	high
52	FBgn0015034	Cyp4e1	2644	CYP4B1	4	3.81	high
	FBgn0015034	Cyp4e1	13265	*CYP4F11*	4	3.81	high
	FBgn0015034	Cyp4e1	26820	*CYP4F22*	4	3.81	high
	FBgn0015034	Cyp4e1	18857	*CYP4F12*	4	3.81	high
	FBgn0015513	mbc	2987	*DOCK1*	12	11.86	high
53	FBgn0015513	mbc	2988	DOCK2	12	11.79	high
54	FBgn0015791	Rab14	16524	RAB14	13	12.8	high
55	FBgn0016696	Pitslre	1729	CDK11B	10	9.88	high
56	FBgn0016756	Usp47	20076	USP47	13	12.8	high
57	FBgn0017578	Max	6913	MAX	13	12.8	high
58	FBgn0019947	Psn	9509	PSEN2	13	12.79	high
59	FBgn0020370	TppII	12016	TPP2	15	14.75	high
60	FBgn0020647	KrT95D	23794	PACS2	13	12.79	high
61	FBgn0022029	l(2)k01209	15938	UCKL1	13	12.82	high
62	FBgn0022359	Sodh-2	11184	SORD	15	14.75	high
63	FBgn0023172	RhoGEF2	14193	ARHGEF12	10	9.89	high
	FBgn0023172	RhoGEF2	14580	*ARHGEF11*	10	9.89	high
64	FBgn0023213	eIF4G	3298	EIF4G3	11	10.74	high
65	FBgn0023458	Rbcn-3A	2938	DMXL2	11	10.93	high
66	FBgn0023549	Mct1	23094	SLC16A12	7	6.89	high
67	FBgn0024689	fws	14857	COG5	13	12.8	high
68	FBgn0024811	Crk	2362	CRK	14	13.8	high
69	FBgn0025592	Gk1	4289	GK	12	11.79	high
	FBgn0025592	Gk1	4291	*GK2*	12	11.79	high
70	FBgn0025684	MFS18	16192	SLC17A9	15	14.75	high
71	FBgn0025687	LKRSDH	17366	AASS	15	14.75	high
72	FBgn0026086	Adar	226	ADARB1	13	12.9	high
73	FBgn0026439	Eaat1	10941	SLC1A3	10	9.78	high
74	FBgn0026666	MagR	28660	ISCA1	14	13.8	high
75	FBgn0027529	tapas	30831	TDRD7	8	7.83	high
76	FBgn0027565	CG5498	28439	CHMP7	12	11.67	high
77	FBgn0027596	Kank	19309	KANK1	11	10.79	high
78	FBgn0027657	glob1	16505	CYGB	7	6.74	high
79	FBgn0027779	VhaSFD	18303	ATP6V1H	15	14.75	high
80	FBgn0027865	Tsp96F	1701	CD81	7	6.87	high
81	FBgn0027948	msps	28959	CKAP5	12	11.94	high
82	FBgn0028411	Nxt1	15913	NXT1	14	13.8	high
83	FBgn0028425	JhI-21	11063	SLC7A5	10	9.79	high
84	FBgn0028427	Ilk	6040	ILK	12	11.78	high
85	FBgn0028429	I-2	9288	PPP1R2	12	11.74	high
86	FBgn0028552	gammaSnap	7642	NAPG	14	13.8	high

S/No	Fly GeneID	Fly Symbol	HGNCID	Human Symbol	DIOPT Score	Weighted Score	Rank
87	FBgn0028646	aralar1	10982	SLC25A12	15	14.75	high
88	FBgn0028663	VhaM9.7-b	863	ATP6V0E1	11	10.84	high
89	FBgn0028670	Vha100-2	866	ATP6V0A4	9	8.79	high
90	FBgn0028703	Nhe3	11079	SLC9A6	14	13.8	high
91	FBgn0028704	Nckx30C	10976	SLC24A2	12	11.85	high
92	FBgn0029088	disp	19711	DISP1	15	14.75	high
93	FBgn0029174	FKBP59	3720	FKBP4	13	12.76	high
94	FBgn0029820	CG16721	25655	TCP11L1	13	12.79	high
95	FBgn0030013	GIIIspla2	17934	PLA2G3	9	8.78	high
96	FBgn0030081	CG7246	18279	UTP6	14	13.8	high
97	FBgn0030228	BTBD9	21228	BTBD9	14	13.8	high
98	FBgn0030286	CG1657	23375	GAPVD1	13	12.85	high
99	FBgn0030528	CG11095	8055	NUDT8	13	12.79	high
100	FBgn0030555	Fbxl4	13601	FBXL4	14	13.8	high
101	FBgn0030670	Pis	1769	CDIPT	15	14.75	high
102	FBgn0030685	Graf	17073	ARHGAP26	14	13.74	high
103	FBgn0030745	CG4239	28462	TMEM38A	14	13.75	high
104	FBgn0030791	CG9132	24539	NECAP1	13	12.84	high
	FBgn0030791	CG9132	25528	*NECAP2*	13	12.8	high
105	FBgn0030887	CG6867	29514	GLDN	5	5.09	high
106	FBgn0030897	Frq1	3953	NCS1	15	14.75	high
107	FBgn0030954	CCKLR-17D3	1571	CCKBR	10	9.82	high
	FBgn0030954	CCKLR-17D3	1570	*CCKAR*	10	9.77	high
108	FBgn0031021	ND-18	7711	NDUFS4	14	13.75	high
109	FBgn0031030	Tao	29259	TAOK1	13	12.87	high
	FBgn0031030	Tao	18133	*TAOK3*	13	12.72	high
110	FBgn0031057	Ubqn	12508	UBQLN1	14	13.8	high
111	FBgn0031081	Nep3	3146	ECE1	12	11.83	high
112	FBgn0031092	CG9577	3149	ECH1	15	14.75	high
113	FBgn0031304	CG4552	25622	TBC1D23	14	13.74	high
114	FBgn0031322	CG5001	14887	DNAJB5	13	12.87	high
	FBgn0031322	CG5001	5270	*DNAJB1*	13	12.8	high
115	FBgn0031456	Trn-SR	17103	TNPO3	12	11.78	high
116	FBgn0031604	Elp3	20696	ELP3	14	13.8	high
117	FBgn0031662	CG3792	7207	MPDU1	14	13.8	high
118	FBgn0031814	retm	29032	SEC14L5	14	13.72	high
119	FBgn0031869	CG18304	21494	SOGA3	4	3.82	high
120	FBgn0031952	cdc14	1718	CDC14A	12	11.79	high
	FBgn0031952	cdc14	1719	*CDC14B*	12	11.74	high
121	FBgn0032036	CG13384	19660	SLC36A4	14	13.8	high
	FBgn0032036	CG13384	18761	*SLC36A1*	14	13.75	high
122	FBgn0032129	jp	14203	JPH3	11	10.79	high
123	FBgn0032156	CG13124	24030	MIF4GD	5	4.95	high
	FBgn0032156	CG13124	23925	*CTIF*	5	4.88	high
124	FBgn0032196	CG5708	6644	LMO4	8	7.86	high
125	FBgn0032297	CG17124	9057	PPP1R14B	8	7.85	high
126	FBgn0032381	Mal-B1	11025	SLC3A1	12	11.76	high
127	FBgn0032455	Pih1D1	26075	PIH1D1	4	3.93	high

S/No	Fly GeneID	Fly Symbol	HGNCID	Human Symbol	DIOPT Score	Weighted Score	Rank
128	FBgn0032470	CG5142	26425	TTC30B	13	12.9	high
129	FBgn0032744	Ttc19	26006	TTC19	13	12.77	high
130	FBgn0032797	Hasp	2334	CR1	3	3.06	high
	FBgn0032797	Hasp	4883	*CFH*	3	2.94	high
131	FBgn0032859	Arpc2	705	ARPC2	14	13.8	high
132	FBgn0032988	Tif-IA	30346	RRN3	14	13.8	high
133	FBgn0033000	CG14464	26798	ARL14EP	9	8.83	high
134	FBgn0033015	d4	9964	DPF2	12	11.8	high
135	FBgn0033194	Vps13	1908	VPS13A	14	13.8	high
136	FBgn0033205	CG2064	17964	RDH11	9	8.73	high
137	FBgn0033247	Nup44A	30379	SEH1L	14	13.8	high
138	FBgn0033266	Socs44A	19383	SOCS1	7	6.75	high
139	FBgn0033382	Hydr1	18718	ABHD3	15	14.75	high
140	FBgn0033639	CG9003	24679	FBXL20	12	11.78	high
141	FBgn0033672	rho-7	18253	PARL	12	11.81	high
142	FBgn0033734	CG8520	16411	AFG1L	15	14.75	high
143	FBgn0033809	CG4630	8495	SLC22A14	4	4.04	high
	FBgn0033809	CG4630	10967	*SLC22A3*	4	3.96	high
144	FBgn0033844	bbc	24289	CEPT1	13	12.85	high
145	FBgn0034002	CG8079	24684	AGGF1	12	11.8	high
146	FBgn0034067	CG8399	27622	FRRS1	15	14.75	high
147	FBgn0034225	veil	8021	NT5E	13	12.67	high
148	FBgn0034275	CG5002	14471	SLC26A11	14	13.75	high
149	FBgn0034308	CG10915	25330	CTTNBP2NL	11	10.76	high
150	FBgn0034417	CG15117	4696	GUSB	15	14.75	high
151	FBgn0034418	CG15118	27880	ANKRD13D	14	13.8	high
152	FBgn0034420	CG10737	1266	C2CD2	4	3.94	high
153	FBgn0034451	TBCB	1989	TBCB	11	10.79	high
154	FBgn0034598	CG4266	20959	SCAF8	11	10.69	high
	FBgn0034598	CG4266	19304	*SCAF4*	11	10.69	high
155	FBgn0034605	CG15661	12530	UGT1A1	6	5.99	high
156	FBgn0034655	CG10307	28228	LRRC39	2	2.11	high
	FBgn0034655	CG10307	37215	*LRRC10B*	2	2.08	high
157	FBgn0034660	lox2	6666	LOXL2	11	10.78	high
	FBgn0034660	lox2	13869	*LOXL3*	11	10.73	high
158	FBgn0034876	wmd	30796	STRAP	13	12.7	high
159	FBgn0034897	Sesn	23060	SESN3	14	13.8	high
160	FBgn0034943	Fmo-1	3773	FMO5	9	8.76	high
161	FBgn0034951	CG3860	15761	OSBPL2	13	12.79	high
162	FBgn0034958	CG3907	29051	EFCAB14	4	3.86	high
163	FBgn0035026	Fcp1	2498	CTDP1	11	10.83	high
164	FBgn0035050	ST6Gal	10860	ST6GAL1	9	8.83	high
	FBgn0035050	ST6Gal	10861	*ST6GAL2*	9	8.78	high
165	FBgn0035060	Eps-15	24634	EPS15L1	13	12.84	high
166	FBgn0035064	TyrRS-m	24249	YARS2	15	14.75	high
167	FBgn0035146	CG13893	18655	SEC14L3	12	11.7	high
168	FBgn0035147	Gale	4116	GALE	14	13.8	high
169	FBgn0035205	CG2469	16850	CTR9	12	11.81	high

S/No	Fly GeneID	Fly Symbol	HGNCID	Human Symbol	DIOPT Score	Weighted Score	Rank
170	FBgn0035227	Iml1	18423	DEPDC5	13	12.84	high
171	FBgn0035232	CG12099	10055	RNF10	14	13.75	high
172	FBgn0035252	CG7970	9716	PXMP2	7	7.05	high
173	FBgn0035287	CG13937	15993	CHST8	5	4.95	high
174	FBgn0035388	CG2162	23512	R3HCC1L	8	7.71	high
175	FBgn0035416	gry	25751	TRAPPC11	12	11.78	high
176	FBgn0035445	CG12014	5389	IDS	15	14.75	high
177	FBgn0035526	CG1316	24468	RBM45	13	12.7	high
178	FBgn0035528	CG15012	30590	TMEM50A	14	13.8	high
179	FBgn0035649	CG10483	12827	XPR1	15	14.75	high
180	FBgn0035827	Srp9	11304	SRP9	13	12.8	high
181	FBgn0035978	UGP	12527	UGP2	12	11.73	high
182	FBgn0035986	CG4022	25210	PIH1D2	4	4.09	high
183	FBgn0035989	CG3967	21186	ATAT1	10	9.74	high
184	FBgn0035993	Nf-YA	7804	NFYA	10	9.88	high
185	FBgn0036133	CG7638	28483	TMEM161B	14	13.8	high
186	FBgn0036141	wls	30238	WLS	14	13.8	high
187	FBgn0036286	CG10616	24299	ODR4	9	8.83	high
188	FBgn0036369	CG10089	16077	DUSP22	11	10.69	high
189	FBgn0036515	AIMP2	20609	AIMP2	13	12.8	high
190	FBgn0036534	DCP2	24452	DCP2	10	9.74	high
191	FBgn0036545	GXIVsPLA2	18554	PLA2G12A	13	12.74	high
192	FBgn0036569	IleRS-m	29685	IARS2	13	12.82	high
193	FBgn0037084	Syx6	11441	STX6	11	10.88	high
194	FBgn0037098	Wnk	14540	WNK1	9	8.95	high
	FBgn0037098	Wnk	14543	*WNK3*	9	8.95	high
195	FBgn0037313	CG1161	18823	TMEM9	14	13.8	high
196	FBgn0037339	Pi4KIIalpha	30031	PI4K2A	11	10.89	high
197	FBgn0037344	CG2926	24351	PHRF1	11	10.88	high
198	FBgn0037382	Hpr1	19070	THOC1	13	12.79	high
199	FBgn0037513	pyd3	16297	UPB1	15	14.75	high
200	FBgn0037530	EMC1	28957	EMC1	13	12.79	high
201	FBgn0037574	Coq2	25223	COQ2	14	13.8	high
202	FBgn0037655	CG11984	20589	KCMF1	11	10.81	high
203	FBgn0037709	CG8199	986	BCKDHA	12	11.89	high
204	FBgn0037756	CG8507	6701	LRPAP1	14	13.85	high
205	FBgn0037892	mRpL40	14491	MRPL40	14	13.8	high
206	FBgn0037913	fabp	9117	PMP2	11	10.76	high
	FBgn0037913	fabp	3562	*FABP7*	11	10.73	high
	FBgn0037950	HisCl1	4329	***GLRB***	5	4.85	high
207	FBgn0038181	CG9297	11295	SRL	10	9.79	high
208	FBgn0038224	ATPsynE	846	ATP5ME	12	11.82	high
209	FBgn0038302	CG4210	23160	SAT2	14	13.85	high
210	FBgn0038564	CG7785	14297	SPRYD7	14	13.8	high
211	FBgn0038693	unc79	19966	UNC79	14	13.85	high
212	FBgn0038925	Cchl	4837	HCCS	13	12.8	high
213	FBgn0039135	CG13603	20137	TMEM179	14	13.8	high
214	FBgn0039141	spas	11233	SPAST	15	14.75	high

S/No	Fly GeneID	Fly Symbol	HGNCID	Human Symbol	DIOPT Score	Weighted Score	Rank
215	FBgn0039153	GatB	8849	GATB	13	12.79	high
216	FBgn0039215	CG6695	17731	CLASRP	13	12.8	high
217	FBgn0039229	Saf-B	10520	SAFB	11	10.94	high
	FBgn0039229	Saf-B	20709	*SLTM*	11	10.83	high
218	FBgn0039241	CG11089	794	ATIC	14	13.74	high
219	FBgn0039258	beta4GalT7	930	B4GALT7	14	13.74	high
220	FBgn0039259	CG11781	28430	EMC6	13	12.79	high
221	FBgn0039357	CG4743	20661	SLC25A26	15	14.75	high
222	FBgn0039562	Gp93	12028	HSP90B1	14	13.82	high
223	FBgn0039732	CG15525	28332	CCDC12	12	11.89	high
224	FBgn0039890	CG2316	66	ABCD2	15	14.75	high
225	FBgn0039902	Zip102B	20182	SLC39A9	14	13.74	high
226	FBgn0039907	lgs	23688	BCL9L	2	2.05	high
227	FBgn0039908	Asator	19140	TTBK1	8	7.87	high
	FBgn0039908	Asator	19141	*TTBK2*	8	7.87	high
228	FBgn0039916	Ekar	4579	GRIK1	8	7.88	high
229	FBgn0039969	Fis1	21689	FIS1	12	11.89	high
230	FBgn0039994	conu	21035	ARHGAP18	10	9.88	high
231	FBgn0040230	dbo	25056	KLHL20	14	13.85	high
232	FBgn0040337	CG3021	25481	TRMU	14	13.74	high
233	FBgn0040394	CG16903	20569	CCNL1	12	11.79	high
234	FBgn0041205	key	5961	IKBKG	4	3.96	high
235	FBgn0041210	HDAC4	14063	HDAC4	10	9.99	high
	FBgn0041210	HDAC4	14068	*HDAC5*	10	9.99	high
236	FBgn0041775	tral	24489	LSM14A	12	11.8	high
237	FBgn0042135	CG18812	18010	GDAP2	14	13.8	high
238	FBgn0043070	MESK2	14462	NDRG3	13	12.84	high
239	FBgn0044048	Ilp5	6081	INS	3	3.01	high
240	FBgn0046247	CG5938	1935	CHIC2	14	13.8	high
241	FBgn0050021	metro	26542	MPP7	13	12.89	high
242	FBgn0050051	Tmem18	25257	TMEM18	12	11.79	high
243	FBgn0050109	CG30109	26937	TRIAP1	14	13.75	high
244	FBgn0050438	CG30438	12555	UGT8	10	9.84	high
245	FBgn0050491	CG30491	19978	RDH13	9	8.8	high
246	FBgn0051472	sgll	30260	PNPO	14	13.7	high
247	FBgn0051619	nolo	14632	ADAMTSL1	7	6.76	high
248	FBgn0052068	Adi1	30576	ADI1	14	13.8	high
249	FBgn0052085	CG32085	14150	FBXL16	10	9.75	high
250	FBgn0053051	CG33051	28466	POLR3GL	8	8	high
	FBgn0053051	CG33051	30075	*POLR3G*	8	7.87	high
251	FBgn0053519	Unc-89	16901	SPEG	6	5.72	high
252	FBgn0053774	CG33774	32483	OST4	6	5.92	high
253	FBgn0058196	Maf1	24966	MAF1	12	11.78	high
254	FBgn0062449	CG13197	3066	DUSP11	14	13.75	high
255	FBgn0085377	CG34348	26510	TMEM68	15	14.75	high
256	FBgn0085446	CG34417	32394	SMTNL1	6	5.97	high
	FBgn0085446	CG34417	11126	*SMTN*	6	5.97	high
257	FBgn0086656	shrb	16171	CHMP4B	14	13.8	high

S/No	Fly GeneID	Fly Symbol	HGNCID	Human Symbol	DIOPT Score	Weighted Score	Rank
258	FBgn0086757	cbs	4424	GOLGA1	11	10.89	high
259	FBgn0086785	Vps36	20312	VPS36	14	13.8	high
260	FBgn0086906	sls	19194	HMCN1	3	2.71	high
261	FBgn0250789	alpha-Spec	11273	SPTAN1	15	14.75	high
262	FBgn0250819	CG33521	28142	LIMD2	2	1.95	high
263	FBgn0250851	CG33981	24540	ANAPC13	12	11.79	high
264	FBgn0250906	Pgk	8896	PGK1	14	13.8	high
265	FBgn0259214	PMCA	816	ATP2B3	14	13.8	high
266	FBgn0259234	Camta	18806	CAMTA1	11	10.79	high
267	FBgn0259678	sqa	29826	MYLK3	5	4.94	high
268	FBgn0259979	CG17337	24437	CNDP2	15	14.75	high
269	FBgn0259991	CG42488	28211	TMEM141	7	6.93	high
270	FBgn0260439	Pp2A-29B	9302	PPP2R1A	15	14.75	high
271	FBgn0260467	CG7071	18085	TMEM199	11	10.86	high
272	FBgn0260639	gammaTub23C	12419	TUBG2	14	13.8	high
	FBgn0260639	gammaTub23C	12417	*TUBG1*	14	13.75	high
273	FBgn0260935	Vps15	8982	PIK3R4	15	14.75	high
274	FBgn0260970	Ubr3	30467	UBR3	13	12.9	high
275	FBgn0261436	DhpD	4212	GDA	15	14.75	high
276	FBgn0261451	trol	5273	HSPG2	9	8.72	high
277	FBgn0261532	cdm	16853	IPO13	14	13.8	high
278	FBgn0261565	Lmpt	3703	FHL2	4	3.98	high
279	FBgn0261625	GLS	4331	GLS	14	13.8	high
280	FBgn0261674	CG42709	32443	LRTM2	2	1.92	high
	FBgn0261674	CG42709	14965	*LRRC3*	2	1.87	high
	FBgn0261674	CG42709	28105	*LRRC3B*	2	1.87	high
281	FBgn0261794	kcc	10913	SLC12A4	15	14.75	high
282	FBgn0262169	magu	20323	SMOC2	10	9.78	high
283	FBgn0262579	Ect4	17074	SARM1	7	6.87	high
284	FBgn0263006	SERCA	811	ATP2A1	14	13.82	high
285	FBgn0263396	sqd	5034	HNRNPAB	13	12.65	high
286	FBgn0263593	Lpin	14451	LPIN3	14	13.75	high
287	FBgn0263980	CG43729	28423	STAC3	5	4.96	high
288	FBgn0264324	spg	2989	DOCK3	14	13.75	high
289	FBgn0264491	how	21100	QKI	13	12.74	high
290	FBgn0264493	rdx	11254	SPOP	10	9.74	high
291	FBgn0264607	CaMKII	1462	CAMK2D	15	14.75	high
292	FBgn0264695	Mhc	7577	MYH7	14	13.8	high
293	FBgn0265045	Strn-Mlck	7590	MYLK	6	5.83	high
294	FBgn0265767	zyd	10977	SLC24A3	7	7.09	high
295	FBgn0265998	Doa	2069	CLK2	11	10.69	high
296	FBgn0266346	CngB	2151	CNGB1	12	11.74	high
297	FBgn0266917	CG16941	10765	SF3A1	14	13.8	high
298	FBgn0267398	Yeti	1873	CFDP1	11	10.83	high

Note: 1) Only "high" ranking human orthologs in DIOPT were analyzed.
2) "**Orange**" boxed fly genes have multiple human orthologs that were labelled as **red**.
3) "**Red**" human orthologs were not counted to prevent over-representation.
4) **GLRB** was counted once. There are **298** highly conserved human orthologs of fly IR genes.

Table S1: Highly conserved human orthologs of fly IR genes that overlapped with curated AD genes

ADARB1	CFDP1	CRYAB	FHL2	HOOK3	KLC1	PLA2G3	RYR2	TTBK1
ADI1	CLK2	DGAT1	FIS1	HSPG2	LMO4	PPP1CA	SLC1A3	UBQLN1
AIMP2	COG5	DOCK2	FKBP4	ILK	LRPAP1	PSEN2	SLC9A6	UBR5
ATAT1	CR1	DUSP22	GPHN	INS	PER3	PTS	SOCS1	WNK1
CDK5	CRK	ECE1	HDAC4	ITPR1	PHB2	RBM45	SPAST	XPR1

	IR	non-IR	total
**AD**	45	2241	2286
**non-AD**	253	18541	18794
**total**	298	20782	21080

Chi square + Yates correction

2-tail = 0.022

1-tail = 0.011

Table S1: Pathway analysis of differential IR genes in aging *Drosophila*

ID	Pathway Name	Gene #	*P* -value	FDR
dme01100	Metabolic pathways	50	3.33E-03	2.40E-01
dme00564	Glycerophospholipid metabolism	7	6.98E-03	2.40E-01
dme00040	Pentose and glucuronate interconversions	6	0.00853	2.40E-01
dme00052	Galactose metabolism	5	9.82E-03	2.40E-01
dme00592	alpha-Linolenic acid metabolism	3	1.01E-02	2.40E-01
dme00565	Ether lipid metabolism	4	1.14E-02	2.40E-01
dme00590	Arachidonic acid metabolism	3	2.17E-02	3.90E-01
dme00500	Starch and sucrose metabolism	4	2.97E-02	4.67E-01
dme00983	Drug metabolism - other enzymes	5	4.70E-02	6.58E-01
P00003	Alzheimer disease-amyloid secretase pathway	4	4.44E-03	3.86E-01
P00019	Endothelin signaling pathway	5	1.95E-02	8.49E-01

Table S1: Fly Differential retained introns (D10 versus D20)

S/N	Gene	ENSEMBL ID	Chr	Intron Start	Intron End	D10 IR ratio	D20 IR ratio	p-value
1	Adar	FBgn0026086	X	1780244	1780330	0.13303718	0.044459717	0.030233393
2	AGO2	FBgn0087035	3L	15560116	15560207	0.141795	0.079338741	0.027288527
3	alpha-Spec	FBgn0250789	3L	1779310	1779384	0.04091218	0.100731213	0.01668034
4	aralar1	FBgn0028646	3R	30446503	30446563	0.08432606	0.176623504	0.009595397
5	Arp1	FBgn0011745	3R	12693155	12693214	0.04760963	0.12957997	0.046817528
6	Arpc2	FBgn0032859	2L	20422411	20422468	0.24370048	0.084283702	0.042156663
7	beta4GalT7	FBgn0039258	3R	25023466	25023527	0.04566581	0.257639098	0.044992848
8	BTBD9	FBgn0030228	X	10769293	10769347	0.03936022	0.219085844	0.039562815
9	Camta	FBgn0259234	2R	9471756	9471818	0.21389755	0.071168205	0.032876165
10	cbs	FBgn0086757	2R	13453030	13453088	0.10093505	0.301990489	0.031237141
11	CCHa1	FBgn0038199	3R	14093308	14096595	0.25413881	0.477188845	0.049557694
12	CCKLR-17D3	FBgn0030954	X	18766169	18766231	0.18105649	0.460043977	0.033357099
13	cdc14	FBgn0031952	2L	7804915	7805059	0.15034926	0.028211774	0.037577293
14	cdm	FBgn0261532	3R	18180625	18180684	0.1048585	0.20199857	0.021846648
15	CG10089	FBgn0036369	3L	13466888	13466943	0.24945662	0.422887547	0.027216531
16	CG10184	FBgn0039094	3R	23603762	23603827	0.19673342	0.297847355	0.04884591
17	CG10307	FBgn0034655	2R	21689868	21689926	0.15626255	0.427127034	0.013225944
18	CG10352	FBgn0030348	X	11876199	11876255	0.2561428	0.114654343	0.047124311
19	CG10483	FBgn0035649	3L	5918165	5918359	0.095217	0.262804507	0.047834388
20	CG10616	FBgn0036286	3L	12471929	12472021	0.04468189	0.252862536	0.036304012
21	CG11095	FBgn0030528	X	13812045	13812111	0.1422769	0.512125488	0.021060803
22	CG11147	FBgn0031734	2L	5734493	5734557	0.12693743	0.019686052	0.014129928
23	CG11474	FBgn0034688	2R	22073067	22073211	0.11705576	0.33665991	0.011252011
24	CG11781	FBgn0039259	3R	25024743	25024799	0.07492884	0.222628135	0.032804631
25	CG11984	FBgn0037655	3R	9011252	9011362	0.11219586	0.04279358	0.046358227
26	CG12090	FBgn0035227	3L	1523969	1524026	0.05243554	0.114823009	0.04487556
27	CG1316	FBgn0035526	3L	4284990	4285052	0.20050619	0.105309354	0.018462065
28	CG15117	FBgn0034417	2R	19123232	19123294	0.27134399	0.117526308	0.044642728
29	CG15118	FBgn0034418	2R	19135625	19135684	0.12263056	0.02226711	0.02934284
30	CG1572	FBgn0030309	X	11556111	11556183	0.05168231	0.114022614	0.04094168
31	CG16941	FBgn0266917	3R	17001806	17001863	0.12856138	0.321129076	0.030513812
32	CG2926	FBgn0037344	3R	5580045	5580114	0.20369057	0.079854142	0.017735738
33	CG31109	FBgn0051109	3R	25053974	25054041	0.18267068	0.414337549	0.027672872
34	CG32068	FBgn0052068	3L	10663568	10663649	0.06769361	0.1448302	0.021089188
35	CG33051	FBgn0053051	3L	17426856	17426925	0.05443066	0.285104797	0.04827475
36	CG33981	FBgn0250851	2R	17775874	17775933	0.0550132	0.156372554	0.029091784
37	CG3792	FBgn0031662	2L	4977685	4977747	0.06788382	0.201853854	0.027871881
38	CG3907	FBgn0034958	2R	24021541	24021789	0.06217053	0.142804885	0.033241722
39	CG4210	FBgn0038302	3R	15214365	15214425	0.04723628	0.243152285	0.049596383
40	CG42593	FBgn0260970	X	7950784	7950862	0.18296248	0.061939935	0.039314101
41	CG44153	FBgn0265002	2L	10068355	10068635	0.13173071	0.30000679	0.049668662
42	CG5282	FBgn0036986	3L	20424844	20424897	0.05961522	0.185994645	0.01770263
43	CG7246	FBgn0030081	X	9079174	9079245	0.07089384	0.394409973	0.048804269
44	CG7785	FBgn0038564	3R	18021819	18022105	0.08710862	0.248238584	0.045648303

S/N	Gene	ENSEMBL ID	Chr	Intron Start	Intron End	D10 IR ratio	D20 IR ratio	p-value
45	CG8079	FBgn0034002	2R	15223159	15223220	0.17818025	0.035301389	0.048500253
46	CG8121	FBgn0037680	3R	9336252	9336482	0.29704992	0.169329154	0.042859235
47	CG9312	FBgn0038179	3R	13766021	13766105	0.16722499	0.26657758	0.02990564
48	CG9717	FBgn0039789	3R	30585220	30585288	0.20481567	0.040303338	0.049962146
49	cin	FBgn0000316	X	256641	256761	0.09852993	0.250056975	0.022929331
50	DhpD	FBgn0261436	3R	4228421	4228479	0.07083374	0.162928737	0.043604659
51	Dip-B	FBgn0000454	3R	13784259	13784316	0.07712572	0.162625239	0.018507764
52	dmpd	FBgn0033486	2R	10119727	10119791	0.06967947	0.18766964	0.04457774
53	Eaat1	FBgn0026439	2L	9339803	9339865	0.08459772	0.133792397	0.043826448
54	eIF4G	FBgn0023213	4	916059	916120	0.10298416	0.020744211	0.040872205
55	Elp3	FBgn0031604	2L	4445136	4445192	0.11196398	0.33016084	0.043909522
56	Eps-15	FBgn0035060	2R	24774505	24774576	0.02396657	0.11427426	0.040636304
57	Eps-15	FBgn0035060	2R	24774813	24774907	0.13808731	0.027211245	0.048459584
58	Fur2	FBgn0004598	X	16379847	16379921	0.14264043	0.049646091	0.037224784
59	GlyP	FBgn0004507	2L	2136644	2136730	0.07290588	0.11561538	0.048497649
60	gol	FBgn0004919	2R	25075338	25075396	0.12558695	0.203906804	0.048819786
61	Graf	FBgn0030685	X	15753836	15753908	0.06448328	0.200170631	0.020038024
62	GstT4	FBgn0030484	X	13408791	13408862	0.13299239	0.211164439	0.040742351
63	Gyk	FBgn0025592	3L	202429	202488	0.05275712	0.175748429	0.01629959
64	HisCl1	FBgn0037950	3R	11787948	11788021	0.38320832	0.173624371	0.027724767
65	Ilk	FBgn0028427	3L	21217732	21217790	0.1591719	0.070027607	0.042599871
66	ird1	FBgn0260935	3R	9243046	9243101	0.0435478	0.273034916	0.027976194
67	Itp-r83A	FBgn0010051	3R	5537325	5537385	0.16025403	0.027952577	0.028580332
68	key	FBgn0041205	2R	24786543	24786601	0.14779026	0.264155353	0.020955349
69	l(2)k01209	FBgn0022029	2R	17424759	17424818	0.11997783	0.237410348	0.049267579
70	l(2)SH0834	FBgn0267365	2L	10311784	10311898	0.07308742	0.194896563	0.046047749
71	Lar	FBgn0000464	2L	19714989	19715760	0.17652888	0.08273375	0.048889254
72	LKR	FBgn0025687	2L	7791876	7791938	0.22428617	0.041940983	0.043403915
73	Max	FBgn0017578	3L	19263524	19263839	0.17074661	0.066974794	0.03209057
74	mbc	FBgn0015513	3R	23800188	23800250	0.14137423	0.028383109	0.04560538
75	mdy	FBgn0004797	2L	16826422	16826482	0.03179694	0.17298234	0.034041673
76	mtSSB	FBgn0010438	3R	16205148	16205215	0.2497372	0.044000356	0.044461975
77	mus205	FBgn0002891	2R	7794417	7794474	0.45671004	0.077176517	0.044005735
78	Obp69a	FBgn0011279	3L	12396739	12396792	0.06869193	0.151106424	0.038382469
79	Phb2	FBgn0010551	2R	18820838	18821040	0.12578243	0.216225994	0.048767845
80	Pitslre	FBgn0016696	3L	20776053	20776173	0.05655866	0.120212901	0.022971356
81	PMCA	FBgn0259214	4	349424	349480	0.05088741	0.102787091	0.033715117
82	Rab14	FBgn0015791	2L	14357937	14358005	0.04241647	0.102440027	0.019719483
83	Rbcn-3A	FBgn0023458	X	6241664	6241727	0.04839372	0.138655738	0.028246891
84	rdgB	FBgn0003218	X	13773753	13773805	0.12186564	0.024073467	0.041817318
85	spg	FBgn0264324	3R	28865867	28865928	0.1526615	0.053763802	0.049080009
86	ST6Gal	FBgn0035050	2R	24692260	24692322	0.25981245	0.114629413	0.048694469
87	UGP	FBgn0035978	3L	9352543	9352601	0.04382223	0.124540896	0.006774187
88	wls	FBgn0036141	3L	11167608	11167818	0.22424949	0.590092304	0.030294598

Table S1: Fly Differential retained introns (D10 versus D30)

S/N	Gene	ENSEMBL ID	Chr	Intron Start	Intron End	D10 IR ratio	D30 IR ratio	p-value
1	Aats-tyr-m	FBgn0035064	2R	24782840	24782892	0.16052025	0.374624297	0.04924425
2	Ack-like	FBgn0263998	2R	13143317	13143374	0.36006659	0.064646149	0.042734749
3	alpha-Est3	FBgn0015571	3R	7539501	7539562	0.17802641	0.383239723	0.012729911
4	aralar1	FBgn0028646	3R	30446503	30446563	0.08432606	0.173849063	0.01196308
5	betaTub97EF	FBgn0003890	3R	27967201	27967268	0.03239882	0.170619595	0.043590625
6	Bsg25D	FBgn0000228	2L	5272928	5272985	0.16512923	0.032549972	0.045181829
7	BTBD9	FBgn0030228	X	10769293	10769347	0.03936022	0.216424387	0.041486422
8	CCHa1	FBgn0038199	3R	14093308	14096595	0.25413881	0.520936249	0.024780517
9	cdm	FBgn0261532	3R	18180625	18180684	0.1048585	0.221402374	0.009286375
10	CG10184	FBgn0039094	3R	23603113	23603353	0.17807342	0.283695354	0.046876374
11	CG10184	FBgn0039094	3R	23603762	23603827	0.19673342	0.336286836	0.011540714
12	CG10249	FBgn0027596	2R	14903564	14903630	0.1224762	0.286590396	0.019262101
13	CG10307	FBgn0034655	2R	21689868	21689926	0.15626255	0.370696878	0.036220475
14	CG10508	FBgn0037060	3L	21214097	21214150	0.05062172	0.149224425	0.027371187
15	CG10616	FBgn0036286	3L	12471929	12472021	0.04468189	0.235716625	0.04483969
16	CG11089	FBgn0039241	3R	24880746	24880805	0.06122286	0.105375869	0.044455642
17	CG11474	FBgn0034688	2R	22073067	22073211	0.11705576	0.288932869	0.032652956
18	CG12014	FBgn0035445	3L	3379571	3379622	0.05552598	0.307115958	0.040656208
19	CG12090	FBgn0035227	3L	1523969	1524026	0.05243554	0.11646585	0.04114061
20	CG12099	FBgn0035232	3L	1556217	1556271	0.08359188	0.165509075	0.023462609
21	CG1316	FBgn0035526	3L	4284990	4285052	0.20050619	0.091441864	0.010800201
22	CG13305	FBgn0035921	3L	8645084	8645167	0.14650963	0.479594803	0.040349614
23	CG13841	FBgn0040588	3R	22767581	22767647	0.02662796	0.131082773	0.045669844
24	CG13893	FBgn0035146	3L	600664	600726	0.06129878	0.198970477	0.018447048
25	CG15117	FBgn0034417	2R	19123232	19123294	0.27134399	0.100375891	0.049242199
26	CG15237	FBgn0033104	2R	6809356	6809423	0.03296612	0.181247274	0.040339188
27	CG16941	FBgn0266917	3R	17002028	17002150	0.14410321	0.330815424	0.042477269
28	CG17005	FBgn0032109	2L	9328992	9329078	0.12632745	0.337379839	0.038742473
29	CG17124	FBgn0032297	2L	10746078	10755903	0.1560831	0.253580258	0.041937337
30	CG17324	FBgn0027074	2L	18822171	18822256	0.52174761	0.157508889	0.029392206
31	CG18812	FBgn0042135	2R	7728957	7729015	0.05887158	0.149268926	0.02477588
32	CG2064	FBgn0033205	2R	7666016	7666145	0.14305637	0.262911275	0.044526603
33	CG3021	FBgn0040337	X	1027546	1027634	0.11824255	0.345884687	0.037381565
34	CG30427	FBgn0043792	2R	24929718	24929771	0.05637281	0.175577541	0.023626364
35	CG31321	FBgn0051321	3R	14313607	14313818	0.13144364	0.273946931	0.024788405
36	CG33774	FBgn0053774	2R	9400068	9400256	0.03451959	0.186213917	0.039429206
37	CG34348	FBgn0085377	X	11343912	11343975	0.48822886	0.2733877	0.025607401
38	CG3726	FBgn0029824	X	5938960	5939038	0.23693862	0.079446111	0.033324342
39	CG3792	FBgn0031662	2L	4977685	4977747	0.06788382	0.186848252	0.044444473
40	CG3967	FBgn0035989	3L	9418999	9419094	0.04799105	0.136502058	0.04126456
41	CG4022	FBgn0035986	3L	9409432	9409519	0.37982354	0.065713754	0.044974554
42	CG42235	FBgn0250757	3R	25918367	25918431	0.1804433	0.535020852	0.043639276
43	CG4266	FBgn0034598	2R	21163695	21163753	0.03174291	0.157321949	0.047152859
44	CG5414	FBgn0036569	3L	16102730	16102785	0.06976341	0.37674924	0.046804724

S/N	Gene	ENSEMBL ID	Chr	Intron Start	Intron End	D10 IR ratio	D30 IR ratio	p-value
45	CG5463	FBgn0039153	3R	24128622	24128685	0.34825544	0.064900724	0.047127603
46	CG5498	FBgn0027565	3L	20383500	20383552	0.21326873	0.038723587	0.036782869
47	CG6329	FBgn0033872	2R	13830718	13830777	0.0746546	0.160949125	0.006364093
48	CG6695	FBgn0039215	3R	24657410	24657515	0.26622137	0.132163065	0.035915529
49	CG6765	FBgn0035903	3L	8516450	8516512	0.03811098	0.200945415	0.04410035
50	CG7227	FBgn0031970	2L	7995691	7995755	0.40037065	0.061160555	0.030758097
51	CG8507	FBgn0037756	3R	9792757	9792812	0.15654885	0.028880965	0.034981176
52	CG9003	FBgn0033639	2R	11644416	11644484	0.21885466	0.094646442	0.04685764
53	CG9935	FBgn0039916	4	640476	640532	0.16611977	0.054653922	0.027181023
54	Chc	FBgn0000319	X	15828882	15828951	0.10237573	0.035242501	0.024303775
55	conu	FBgn0039994	2R	4756588	4756654	0.10415274	0.190040609	0.039804401
56	d4	FBgn0033015	2R	5294712	5294796	0.07901433	0.183629224	0.01974937
57	Dip-B	FBgn0000454	3R	13784259	13784316	0.07712572	0.156402924	0.027659605
58	Doa	FBgn0265998	3R	28921549	28921614	0.09549405	0.167184635	0.045147385
59	dpr2	FBgn0261871	2L	10918018	10918113	0.20102544	0.444682628	0.049916213
60	Eaat1	FBgn0026439	2L	9339803	9339865	0.08459772	0.13678896	0.039720251
61	Fis1	FBgn0039969	2R	5604411	5604467	0.13023662	0.196565086	0.048234977
62	FKBP59	FBgn0029174	2L	9888985	9889047	0.11087244	0.200333276	0.022925258
63	Frq1	FBgn0030897	X	18167656	18167784	0.08916794	0.209238036	0.047502709
64	fws	FBgn0024689	2L	17480242	17480295	0.22642491	0.043001305	0.047666781
65	GIIIspla2	FBgn0030013	X	8158386	8158564	0.1581922	0.031712882	0.045597794
66	Gycalpha99B	FBgn0013972	3R	29685347	29685409	0.12329102	0.248221779	0.018128788
67	hk	FBgn0001202	2L	19031762	19031820	0.07394305	0.165498711	0.037332987
68	HmgD	FBgn0004362	2R	21715617	21715691	0.02422297	0.115802161	0.04940876
69	Hnf4	FBgn0004914	2L	8688857	8688920	0.13078479	0.026424004	0.044582512
70	hyd	FBgn0002431	3R	9715454	9715520	0.14877585	0.028851657	0.03899367
71	Hydr2	FBgn0014906	2L	3167973	3168034	0.0623914	0.137739102	0.042573572
72	Ilp5	FBgn0044048	3L	9823639	9823710	0.15037317	0.312212479	0.040212618
73	Itp-r83A	FBgn0010051	3R	5537325	5537385	0.16025403	0.056319028	0.036524451
74	Jhl-21	FBgn0028425	2L	12053924	12053981	0.23868337	0.395503012	0.031016422
75	kcc	FBgn0261794	2R	23924549	23924615	0.12038658	0.180591018	0.048434883
76	lgs	FBgn0039907	4	438918	438974	0.17741653	0.078051577	0.04639621
77	Lmpt	FBgn0261565	3L	16902068	16902128	0.14523922	0.04990354	0.027390312
78	lox2	FBgn0034660	2R	21790217	21790291	0.06482785	0.410685734	0.035776887
79	lva	FBgn0029688	X	3955972	3956048	0.11871376	0.217233754	0.038331002
80	magu	FBgn0262169	2R	10062191	10062249	0.03890903	0.220415814	0.036819188
81	Mal-B1	FBgn0032381	2L	11848827	11848898	0.09472803	0.295237536	0.026919444
82	Mct1	FBgn0023549	X	2204437	2204508	0.63205593	0.271538471	3.12E-05
83	Mct1	FBgn0023549	X	2204709	2204764	0.65955011	0.409298187	0.013877425
84	mRpL40	FBgn0037892	3R	11408326	11408385	0.07979379	0.232710201	0.044737137
85	Nhe3	FBgn0028703	2L	6680114	6680174	0.0421294	0.11293729	0.037724762
86	Nup44A	FBgn0033247	2R	7990867	7990927	0.08006676	0.216006791	0.049138622
87	Obp19b	FBgn0031110	X	20416928	20416993	0.11149987	0.221453488	0.022167001
88	ort	FBgn0003011	3R	19661662	19661720	0.09838617	0.311985274	0.032915506
89	pio	FBgn0020521	2R	24596339	24596395	0.02098333	0.107699484	0.037228054

S/N	Gene	ENSEMBL ID	Chr	Intron Start	Intron End	D10 IR ratio	D30 IR ratio	p-value
90	PMCA	FBgn0259214	4	349146	349212	0.0353167	0.10634628	0.025467201
91	PMCA	FBgn0259214	4	349424	349480	0.05088741	0.10124541	0.043754597
92	Pp1alpha-96/	FBgn0003134	3R	24519881	24519939	0.1133618	0.179250388	0.041221105
93	Pp2A-29B	FBgn0260439	2L	8367249	8367311	0.05036876	0.116526098	0.042429452
94	Psn	FBgn0019947	3L	20434032	20434086	0.13171681	0.26903433	0.030474908
95	pst	FBgn0035770	3L	7359389	7359467	0.04659692	0.119657262	0.02236663
96	pyd3	FBgn0037513	3R	7748193	7748253	0.0307932	0.155122219	0.048632184
97	retm	FBgn0031814	2L	6451736	6451796	0.05295437	0.135548747	0.021521156
98	rho-7	FBgn0033672	2R	11988296	11988361	0.14628946	0.313703695	0.009612543
99	santa-maria	FBgn0025697	2L	7446935	7446995	0.0359254	0.108148676	0.026512408
100	Sesn	FBgn0034897	2R	23732503	23732561	0.15173644	0.2433744	0.043471443
101	SLC5A11	FBgn0031998	2L	8200960	8201016	0.24400713	0.370829158	0.042849618
102	Snmp1	FBgn0260004	3R	21167957	21168017	0.09640713	0.220220533	0.031459151
103	SPE	FBgn0039102	3R	23687630	23687690	0.110775	0.061569791	0.04615578
104	sqd	FBgn0263396	3R	13642427	13642511	0.02040962	0.102027685	0.036671686
105	TppII	FBgn0020370	2R	13155210	13155268	0.06239299	0.173166054	0.04395116
106	Vha100-2	FBgn0028670	3R	18391196	18391258	0.11937242	0.044209231	0.038057639
107	yellow-f2	FBgn0038105	3R	12994319	12994427	0.1355182	0.242618032	0.036616614
108	Yeti	FBgn0267398	2R	1344381	1344450	0.12356058	0.02330414	0.033664844

Table S1: Fly Differential retained introns (D10 versus D50)

S/N	Gene	ENSEMBL ID	Chr	Intron Start	Intron End	D10 IR ratio	D50 IR ratio	p-value
1	Actn	FBgn0000667	X	2027462	2027520	0.07356137	0.203647836	0.003434916
2	alpha-Est3	FBgn0015571	3R	7539501	7539562	0.17802641	0.36738894	0.017053908
3	Ank2	FBgn0261788	3L	7696724	7696779	0.16189892	0.321167207	0.016868164
4	Ank2	FBgn0261788	3L	7696878	7696937	0.18135997	0.354038108	0.031460538
5	Arp1	FBgn0011745	3R	12693155	12693214	0.04760963	0.133298315	0.042009599
6	Asator	FBgn0039908	4	477102	477291	0.11242887	0.216976501	0.028746096
7	atms	FBgn0010750	3R	4775779	4775837	0.0423678	0.23218123	0.042938881
8	Bacc	FBgn0031453	2L	2756170	2756244	0.08741066	0.154484737	0.001792729
9	bbc	FBgn0033844	2R	13491593	13491654	0.08519753	0.213434815	0.002549378
10	bol	FBgn0011206	3L	9123776	9125166	0.37121122	0.732764147	0.041645447
11	bt	FBgn0005666	4	767690	767761	0.02628194	0.193874954	0.034638189
12	bt	FBgn0005666	4	772459	774016	0.16172372	0.271460353	0.025339501
13	bt	FBgn0005666	4	774117	774184	0.02218852	0.124320576	0.049364197
14	bt	FBgn0005666	4	774679	774914	0.0541167	0.117791095	0.011106947
15	Ca-P60A	FBgn0263006	2R	23929431	23929499	0.04132889	0.114110695	0.004725682
16	Ca-P60A	FBgn0263006	2R	23929617	23929681	0.04000226	0.119827376	0.001246911
17	Ca-P60A	FBgn0263006	2R	23932329	23932405	0.03813459	0.125382383	0.000387719
18	Ca-P60A	FBgn0263006	2R	23932488	23932573	0.03005445	0.102050153	0.003010243
19	CaMKII	FBgn0264607	4	1050609	1053497	0.1589038	0.267553221	0.025841388
20	Camta	FBgn0259234	2R	9471756	9471818	0.21389755	0.07184825	0.034466746
21	cbs	FBgn0086757	2R	13453030	13453088	0.10093505	0.28486845	0.0437035
22	CCHa1	FBgn0038199	3R	14093037	14093136	0.29259071	0.580867805	0.04053792
23	CCHa1	FBgn0038199	3R	14093308	14096595	0.25413881	0.557998282	0.01523667

S/N	Gene	ENSEMBL ID	Chr	Intron Start	Intron End	D10 IR ratio	D50 IR ratio	p-value
24	Cchl	FBgn0038925	3R	22027066	22027159	0.08344646	0.185281382	0.028862461
25	Cdk5	FBgn0013762	2R	15571070	15571134	0.06442026	0.188120547	0.040551739
26	cdm	FBgn0261532	3R	18180625	18180684	0.1048585	0.210237501	0.016430989
27	CecC	FBgn0000279	3R	30216675	30216744	0.36015091	0.089859376	0.005400261
28	CG10186	FBgn0032797	2L	19510730	19510786	0.05543601	0.101724404	0.042227742
29	CG10186	FBgn0032797	2L	19514105	19514167	0.08161395	0.16145808	0.026356568
30	CG10616	FBgn0036286	3L	12471929	12472021	0.04468189	0.248658344	0.038739817
31	CG10680	FBgn0032836	2L	19965010	19965062	0.20096753	0.138183687	0.049866939
32	CG10737	FBgn0034420	2R	19176347	19176415	0.07168292	0.185565786	0.003323545
33	CG10915	FBgn0034308	2R	18162138	18162206	0.05805937	0.189459722	0.020082638
34	CG10962	FBgn0030073	X	8973380	8973654	0.15994231	0.373782612	0.037369011
35	CG11550	FBgn0039864	3R	31679440	31679522	0.10412607	0.202449451	0.018476096
36	CG11560	FBgn0036249	3L	12120588	12120654	0.11326395	0.328679542	0.042012305
37	CG1161	FBgn0037313	3R	5355253	5355326	0.06227774	0.152937222	0.036811908
38	CG11893	FBgn0039316	3R	25290992	25291055	0.34889817	0.197622626	0.031102502
39	CG12090	FBgn0035227	3L	1523969	1524026	0.05243554	0.116332388	0.039772602
40	CG12090	FBgn0035227	3L	1531522	1531579	0.10271733	0.289062237	0.002183637
41	CG12203	FBgn0031021	X	19492997	19493056	0.06828278	0.142458447	0.042489182
42	CG12304	FBgn0036515	3L	15584868	15584922	0.10383018	0.242947264	0.04522613
43	CG12730	FBgn0029771	X	5517498	5517571	0.13278534	0.303055587	0.011289955
44	CG13124	FBgn0032156	2L	9904758	9904826	0.08948312	0.16048376	0.047116359
45	CG1316	FBgn0035526	3L	4284990	4285052	0.20050619	0.081206188	0.005399708
46	CG13197	FBgn0062449	2R	11694581	11694654	0.16692675	0.301429879	0.045216577
47	CG13384	FBgn0032036	2L	8387831	8387890	0.05512787	0.167710484	0.021793036
48	CG13603	FBgn0039135	3R	23986099	23986165	0.02281933	0.112239242	0.043313781
49	CG13795	FBgn0031937	2L	7725473	7725534	0.14791886	0.302354386	0.028672654
50	CG13833	FBgn0039040	3R	23033729	23033796	0.06033837	0.18616486	0.00222526
51	CG13893	FBgn0035146	3L	600664	600726	0.06129878	0.169502036	0.048303515
52	CG13937	FBgn0035287	3L	1877662	1877760	0.2167863	0.371438888	0.012402556
53	CG14141	FBgn0036146	3L	11201642	11201695	0.2456744	0.451515353	0.04987297
54	CG14274	FBgn0032023	2L	8338488	8338579	0.15691532	0.281125544	0.047084808
55	CG14464	FBgn0033000	2R	4829493	4829546	0.09244719	0.216268419	0.003661938
56	CG15012	FBgn0035528	3L	4291623	4291686	0.02399388	0.117424168	0.042863152
57	CG15118	FBgn0034418	2R	19135625	19135684	0.12263056	0.02512163	0.045228589
58	CG15525	FBgn0039732	3R	29997375	29997437	0.04029388	0.2355626	0.037322954
59	CG15628	FBgn0031632	2L	4827100	4827172	0.0546593	0.148325385	0.046915201
60	CG15661	FBgn0034605	2R	21215908	21215970	0.09547773	0.184365155	0.029930789
61	CG1657	FBgn0030286	X	11333318	11333387	0.05858796	0.171214757	0.027985354
62	CG16721	FBgn0029820	X	5881870	5881934	0.04590496	0.145414603	0.02376045
63	CG1674	FBgn0039897	4	236475	237268	0.0313073	0.170214214	0.03344402
64	CG1674	FBgn0039897	4	240398	240644	0.10190006	0.198118953	0.032650846
65	CG16903	FBgn0040394	X	2078774	2078831	0.12347494	0.269613653	0.032642237
66	CG16941	FBgn0266917	3R	17002028	17002150	0.14410321	0.325376238	0.047560249
67	CG17337	FBgn0259979	2R	5698008	5698455	0.14633678	0.292700592	0.012162423
68	CG18304	FBgn0031869	2L	6938148	6938202	0.06740934	0.364955603	0.04581189

S/N	Gene	ENSEMBL ID	Chr	Intron Start	Intron End	D10 IR ratio	D50 IR ratio	p-value
69	CG18304	FBgn0031869	2L	6939715	6940610	0.0890988	0.550429931	0.037983012
70	CG18467	FBgn0034218	2R	17440875	17441486	0.17358504	0.367538074	0.023839505
71	CG2162	FBgn0035388	3L	3036661	3036728	0.09441834	0.241377875	0.01845474
72	CG2316	FBgn0039890	4	185916	185977	0.12713559	0.209649248	0.039726981
73	CG2469	FBgn0035205	3L	1309793	1309856	0.03110766	0.172499988	0.036088707
74	CG2926	FBgn0037344	3R	5580045	5580114	0.20369057	0.08694973	0.031398643
75	CG2943	FBgn0037530	3R	7903889	7903953	0.14942943	0.066948934	0.044190932
76	CG30051	FBgn0050051	2R	12436256	12436320	0.09701507	0.306978052	0.028634121
77	CG30109	FBgn0050109	2R	17670328	17670386	0.03995317	0.246211739	0.026652213
78	CG30438	FBgn0050438	2R	5548775	5548827	0.04461782	0.266741488	0.031648158
79	CG30491	FBgn0050491	2R	7656100	7656155	0.11929374	0.308041521	0.019630834
80	CG30503	FBgn0050503	2R	7445517	7445581	0.14722238	0.38942825	0.003082494
81	CG31103	FBgn0051103	3R	25219884	25219945	0.20308499	0.081632719	0.022936485
82	CG31109	FBgn0051109	3R	25053974	25054041	0.18267068	0.429610928	0.023777181
83	CG31191	FBgn0051191	3R	20911912	20911970	0.62850615	0.289145117	0.005631098
84	CG31191	FBgn0051191	3R	20912077	20912141	0.42213347	0.203957336	0.043307603
85	CG31619	FBgn0051619	2L	21713308	21716324	0.05026234	0.159948833	0.026480044
86	CG32085	FBgn0052085	3L	11668312	11668431	0.37152979	0.573042889	0.027167503
87	CG32523	FBgn0052523	X	21218157	21218224	0.07659113	0.153838114	0.032143132
88	CG33110	FBgn0053110	3R	22572747	22574361	0.20961284	0.315524729	0.040818492
89	CG3321	FBgn0038224	3R	14327183	14327252	0.21361737	0.408151102	0.026897405
90	CG33521	FBgn0250819	4	1188349	1189053	0.11630221	0.246410415	0.023307229
91	CG33521	FBgn0250819	4	1190008	1190066	0.19909418	0.401179606	0.005838013
92	CG33521	FBgn0250819	4	1192162	1192224	0.20949517	0.468952136	0.009345518
93	CG33774	FBgn0053774	2R	9400068	9400256	0.03451959	0.211480439	0.026300376
94	CG34355	FBgn0085384	3R	23849129	23849187	0.05692964	0.171097586	0.026519582
95	CG34417	FBgn0085446	X	6576957	6577036	0.04431777	0.142672342	0.012978893
96	CG34417	FBgn0085446	X	6585785	6585847	0.04519831	0.122665973	0.033140697
97	CG34423	FBgn0085452	2R	23358767	23358832	0.22202909	0.542015644	0.029121029
98	CG3860	FBgn0034951	2R	23992574	23992634	0.11257206	0.217965813	0.035156622
99	CG40006	FBgn0058006	2L	22718975	22755856	0.03014132	0.158162382	0.044055371
100	CG4210	FBgn0038302	3R	15214365	15214425	0.04723628	0.268916245	0.036237567
101	CG42268	FBgn0259163	3L	9750299	9750891	0.0577187	0.142837564	0.035033837
102	CG42361	FBgn0259707	2R	24608261	24608318	0.27627318	0.413934895	0.045752717
103	CG4239	FBgn0030745	X	16387619	16387680	0.02986964	0.154506241	0.040048386
104	CG42488	FBgn0259991	3R	25484302	25484360	0.03859834	0.206758653	0.043837737
105	CG42596	FBgn0260995	2R	3079082	3121990	0.06447215	0.159083876	0.026700491
106	CG42596	FBgn0260995	2R	3122084	3182391	0.05203759	0.145916291	0.044309931
107	CG42708	FBgn0261625	2R	12672587	12672663	0.03022383	0.161383911	0.03941761
108	CG42709	FBgn0261674	3L	12795324	12795457	0.08504768	0.23244966	0.045947297
109	CG43078	FBgn0262508	3L	8583766	8585970	0.19658166	0.339259823	0.031307798
110	CG43673	FBgn0263748	X	15779191	15779379	0.09227224	0.2861936	0.009542935
111	CG43729	FBgn0263980	2R	15416838	15419350	0.24602609	0.659025317	0.009863773
112	CG43897	FBgn0264489	3L	9832866	9832927	0.04774418	0.16840546	0.000124405
113	CG44085	FBgn0264894	2L	13261177	13261500	0.1493469	0.391458628	0.008009526

S/N	Gene	ENSEMBL ID	Chr	Intron Start	Intron End	D10 IR ratio	D50 IR ratio	p-value
114	CG44085	FBgn0264894	2L	13274376	13274642	0.13981764	0.337792526	0.000546336
115	CG44153	FBgn0265002	2L	10068355	10068635	0.13173071	0.313813503	0.038539983
116	CG45076	FBgn0266446	3R	10770432	10771037	0.15151441	0.302831133	0.002840782
117	CG45076	FBgn0266446	3R	10771467	10771859	0.09541629	0.178348347	0.040320159
118	CG45076	FBgn0266446	3R	10771972	10772112	0.09621057	0.174390766	0.048581884
119	CG4552	FBgn0031304	2L	1131181	1131263	0.02684616	0.132346437	0.045246414
120	CG4630	FBgn0033809	2R	13215966	13216022	0.10140078	0.345464419	0.022726314
121	CG4743	FBgn0039357	3R	25680008	25680071	0.04756603	0.263873322	0.040059789
122	CG5001	FBgn0031322	2L	1191534	1191598	0.03823124	0.250813778	0.021825494
123	CG5001	FBgn0031322	2L	1192198	1192255	0.14005952	0.45247949	0.02199076
124	CG5002	FBgn0034275	2R	17752644	17752706	0.15662527	0.385144082	0.009531032
125	CG5142	FBgn0032470	2L	13108282	13108415	0.28335279	0.045256925	0.02902984
126	CG5612	FBgn0039529	3R	27624173	27624230	0.08148688	0.251323639	0.023790544
127	CG5708	FBgn0032196	2L	10224892	10224957	0.03492553	0.181017293	0.045836783
128	CG5938	FBgn0046247	3R	27276503	27276569	0.04980035	0.135683271	0.040821284
129	CG6083	FBgn0036183	3L	11625552	11625616	0.08533519	0.350837543	0.008721668
130	CG6329	FBgn0033872	2R	13830718	13830777	0.0746546	0.139414577	0.029253017
131	CG6356	FBgn0039178	3R	24286946	24287003	0.18501866	0.332325937	0.035572452
132	CG6656	FBgn0038912	3R	21877400	21877466	0.07452418	0.151694965	0.049470678
133	CG6765	FBgn0035903	3L	8516450	8516512	0.03811098	0.22722396	0.030138837
134	CG6867	FBgn0030887	X	18025119	18025180	0.04133277	0.22809096	0.040475937
135	CG7071	FBgn0260467	3R	22363287	22363353	0.11759976	0.266590173	0.04988098
136	CG7607	FBgn0036145	3L	11200198	11200252	0.14744534	0.274911641	0.024270644
137	CG7627	FBgn0032026	2L	8356974	8357033	0.08863391	0.20701467	0.045843823
138	CG7638	FBgn0036133	3L	11110800	11110861	0.03370223	0.200506591	0.02872531
139	CG7720	FBgn0038652	3R	18792120	18795010	0.25166213	0.486378626	0.020996953
140	CG7781	FBgn0032021	2L	8322702	8322773	0.07052131	0.138240294	0.015452876
141	CG7785	FBgn0038564	3R	18021819	18022105	0.08710862	0.265134021	0.035768617
142	CG7970	FBgn0035252	3L	1653254	1653316	0.05147491	0.168103619	0.016982757
143	CG8199	FBgn0037709	3R	9556538	9556604	0.04252742	0.251632743	0.031471676
144	CG8388	FBgn0034062	2R	16009716	16009772	0.13980768	0.496288188	0.01859887
145	CG8399	FBgn0034067	2R	16023687	16023746	0.03396494	0.166945161	0.049152198
146	CG8520	FBgn0033734	2R	12424108	12424165	0.08846793	0.224749369	0.022971195
147	CG8920	FBgn0027529	2R	20325537	20325595	0.10569101	0.198130366	0.031170851
148	CG9003	FBgn0033639	2R	11644179	11644238	0.08894507	0.21023998	0.039578778
149	CG9132	FBgn0030791	X	16798684	16798749	0.05445797	0.147586204	0.041098837
150	CG9297	FBgn0038181	3R	13775169	13775227	0.01946499	0.12852651	0.01521315
151	CG9449	FBgn0036875	3L	19494918	19496550	0.13308484	0.24948537	0.025384935
152	CG9577	FBgn0031092	X	20167119	20167190	0.08402026	0.209386242	0.027313545
153	chp	FBgn0267435	3R	31206196	31206255	0.07197651	0.119712384	0.047795544
154	cin	FBgn0000316	X	256641	256761	0.09852993	0.2329973	0.041765758
155	CngB	FBgn0266346	2R	21693659	21693717	0.04797216	0.256305847	0.044768102
156	Coq2	FBgn0037574	3R	8316413	8316472	0.06015485	0.355595926	0.043508674
157	cora	FBgn0010434	2R	19236208	19236266	0.02153344	0.104758999	0.044590143
158	Cpr60D	FBgn0050163	2R	24663232	24663290	0.11073616	0.360340264	0.007488612

S/N	Gene	ENSEMBL ID	Chr	Intron Start	Intron End	D10 IR ratio	D50 IR ratio	p-value
159	Crk	FBgn0024811	4	210486	210557	0.12683032	0.221296218	0.008871043
160	Cyp28c1	FBgn0030339	X	11839797	11839857	0.04757424	0.272237211	0.048953643
161	Cyp28c1	FBgn0030339	X	11840203	11840261	0.14749052	0.428345253	0.03028423
162	Cyp4e1	FBgn0015034	2R	8447662	8447932	0.11688366	0.407197247	0.025943441
163	d4	FBgn0033015	2R	5294712	5294796	0.07901433	0.171885715	0.032313056
164	dbo	FBgn0040230	3L	15829037	15829185	0.12592822	0.248482195	0.048202182
165	dbr	FBgn0067779	2L	70549	70606	0.04251734	0.239466636	0.035627418
166	DCP2	FBgn0036534	3L	15822011	15822070	0.06764255	0.192023229	0.040318913
167	disp	FBgn0029088	3R	5852694	5852766	0.04603508	0.245978257	0.046409
168	dor	FBgn0000482	X	1667825	1667887	0.23825346	0.466575369	0.048608124
169	dpr16	FBgn0037295	3R	5165834	5166079	0.3613917	0.124358019	0.041732124
170	dpr17	FBgn0051361	3R	12110687	12110831	0.3225024	0.103917268	0.025790548
171	dpr19	FBgn0032233	2L	10378221	10378287	0.0324333	0.163753924	0.049362059
172	Eaat1	FBgn0026439	2L	9339803	9339865	0.08459772	0.138456238	0.034996574
173	Eaat1	FBgn0026439	2L	9340488	9340543	0.05851135	0.121427034	0.009408326
174	Ect4	FBgn0262579	3L	8072304	8072368	0.15058697	0.447252778	0.042052637
175	Eps-15	FBgn0035060	2R	24772037	24772097	0.05076326	0.152975329	0.02328958
176	Eps-15	FBgn0035060	2R	24774505	24774576	0.02396657	0.135889608	0.027983328
177	fabp	FBgn0037913	3R	11560792	11560851	0.0375333	0.101000669	0.048446554
178	Fbxl4	FBgn0030555	X	14236714	14236845	0.1357816	0.045971479	0.037511405
179	Fcp1	FBgn0035026	2R	24568602	24568659	0.08135175	0.247631255	0.03082896
180	Fis1	FBgn0039969	2R	5604411	5604467	0.13023662	0.202607436	0.032960606
181	Fmo-1	FBgn0034943	2R	23963717	23963774	0.15860498	0.457730109	0.044724574
182	Fur2	FBgn0004598	X	16379847	16379921	0.14264043	0.028367664	0.043964399
183	Gale	FBgn0035147	3L	650523	650583	0.02114886	0.103598755	0.04059019
184	gammaSnap1	FBgn0028552	2R	24048450	24048638	0.07741346	0.144761699	0.037002799
185	gammaSnap1	FBgn0028552	2R	24048706	24048768	0.01903334	0.102622707	0.029869368
186	gammaTub23	FBgn0260639	2L	2973107	2973165	0.13084469	0.293682348	0.035126177
187	GILT1	FBgn0038149	3R	13399432	13399509	0.04428084	0.102735484	0.041087057
188	glob1	FBgn0027657	3R	15898015	15901009	0.28347201	0.452850778	0.025976798
189	GlyP	FBgn0004507	2L	2136644	2136730	0.07290588	0.117122949	0.044562248
190	gol	FBgn0004919	2R	25077306	25077369	0.03869437	0.11516461	0.037092766
191	Gp93	FBgn0039562	3R	27948901	27948983	0.07760425	0.159146412	0.033732961
192	grass	FBgn0039494	3R	27158117	27158191	0.18608501	0.335188315	0.030019039
193	grk	FBgn0001137	2L	8433272	8433414	0.20340598	0.42844073	0.028770701
194	gry	FBgn0035416	3L	3200458	3200516	0.28330102	0.082534323	0.018660357
195	GXIVsPLA2	FBgn0036545	3L	15955547	15955610	0.10762895	0.230857393	0.038583523
196	Gycalpha99B	FBgn0013972	3R	29683938	29683998	0.06869843	0.166325628	0.026513182
197	HDAC4	FBgn0041210	X	13270320	13270390	0.07407569	0.218608978	0.025755242
198	how	FBgn0264491	3R	22042355	22042906	0.18918112	0.344300135	0.021948437
199	Hpr1	FBgn0037382	3R	5819644	5819698	0.10382415	0.316039038	0.030942568
200	Hydr1	FBgn0033382	2R	9133770	9133841	0.05250782	0.146119163	0.039503141
201	I-2	FBgn0028429	3L	9835980	9836048	0.02938058	0.178941341	0.022519432
202	inaC	FBgn0004784	2R	16900512	16900575	0.06877622	0.110238557	0.024561229
203	jp	FBgn0032129	2L	9553939	9553999	0.05216978	0.195848943	0.007780928

S/N	Gene	ENSEMBL ID	Chr	Intron Start	Intron End	D10 IR ratio	D50 IR ratio	p-value
204	kcc	FBgn0261794	2R	23922856	23922921	0.05363286	0.105967769	0.021800875
205	Klc	FBgn0010235	3L	12747941	12748055	0.12685288	0.042224478	0.01972242
206	KrT95D	FBgn0020647	3R	24000117	24000179	0.08179248	0.176519161	0.034273563
207	l(1)G0136	FBgn0026666	X	15734606	15735098	0.09004508	0.180548539	0.024964212
208	l(2)01289	FBgn0010482	2R	6722254	6722307	0.09775955	0.334410669	0.013562888
209	l(2)01289	FBgn0010482	2R	6722464	6722527	0.04819589	0.333000442	0.019650641
210	l(2)01289	FBgn0010482	2R	6722694	6722751	0.04564874	0.261672466	0.03607453
211	l(2)01289	FBgn0010482	2R	6725639	6725703	0.0518885	0.404149732	0.014542201
212	l(2)01289	FBgn0010482	2R	6732184	6732237	0.04602203	0.270211164	0.03392349
213	l(2)01289	FBgn0010482	2R	6732882	6732942	0.04711507	0.265590909	0.039119863
214	l(2)01289	FBgn0010482	2R	6733257	6733627	0.20145128	0.416340124	0.03896836
215	l(2)01289	FBgn0010482	2R	6733942	6734013	0.11167666	0.415213632	0.009939
216	l(2)01289	FBgn0010482	2R	6734194	6734258	0.0542462	0.423148671	0.015886541
217	l(2)01289	FBgn0010482	2R	6734392	6734454	0.1024774	0.398066333	0.011443267
218	l(2)efl	FBgn0011296	2R	23684932	23684994	0.08938535	0.195780469	0.010550867
219	l(2)efl	FBgn0011296	2R	23685382	23685475	0.18692919	0.436364443	0.000381797
220	l(2)k01209	FBgn0022029	2R	17424759	17424818	0.11997783	0.2411774	0.044288788
221	l(3)neo18	FBgn0011455	3L	12137944	12138015	0.06620056	0.118904483	0.046272954
222	Lar	FBgn0000464	2L	19714989	19715760	0.17652888	0.073337068	0.038106381
223	lbm	FBgn0016032	2R	7049965	7050033	0.04188411	0.28179423	0.022195972
224	Lin29	FBgn0262636	4	377669	377757	0.07318248	0.194875303	0.013075514
225	Lpin	FBgn0263593	2R	8141820	8141890	0.04303606	0.172620372	0.000731889
226	Lpin	FBgn0263593	2R	8142028	8142098	0.01805704	0.137408536	0.007256546
227	lt	FBgn0002566	2L	22924925	22924981	0.19258857	0.332964305	0.049256628
228	lt	FBgn0002566	2L	22925498	22925565	0.09464086	0.283511983	0.000878763
229	lt	FBgn0002566	2L	22925919	22927437	0.10191852	0.27961071	0.003563099
230	Maf1	FBgn0058196	2R	1516468	1516519	0.11001495	0.169956029	0.045011492
231	mam	FBgn0002643	2R	14057196	14057261	0.07281163	0.209433817	0.047395235
232	Mct1	FBgn0023549	X	2204437	2204508	0.63205593	0.33231668	0.001010317
233	Mct1	FBgn0023549	X	2204709	2204764	0.65955011	0.361961552	0.002529478
234	mdy	FBgn0004797	2L	16826422	16826482	0.03179694	0.159596931	0.044855162
235	MESK2	FBgn0043070	2R	21502479	21510866	0.21576984	0.350434345	0.049861573
236	metro	FBgn0050021	2R	11227543	11227662	0.07494945	0.176938569	0.034742872
237	Mf	FBgn0038294	3R	15176293	15176363	0.02500384	0.163890637	1.64E-07
238	Mf	FBgn0038294	3R	15179180	15180207	0.04566425	0.143153733	0.000339925
239	Mf	FBgn0038294	3R	15179180	15180292	0.34949452	0.881485405	0.003190682
240	MFS18	FBgn0025684	2L	4447851	4447905	0.03825177	0.217348676	0.035510352
241	Mhc	FBgn0264695	2L	16771141	16773276	0.0389302	0.101844322	0.033619786
242	Mhc	FBgn0264695	2L	16773380	16774449	0.04162254	0.118835529	0.032333804
243	Mhc	FBgn0264695	2L	16774857	16775682	0.07877384	0.129923847	0.003488538
244	Mhc	FBgn0264695	2L	16774857	16776262	0.23062441	0.399003368	0.001461533
245	Mhc	FBgn0264695	2L	16777439	16778389	0.1559591	0.283627716	0.040970535
246	Mhc	FBgn0264695	2L	16778507	16780549	0.17138294	0.316955733	0.04626364
247	Mhc	FBgn0264695	2L	16783661	16783832	0.27938196	0.466970129	0.013995493
248	Mhc	FBgn0264695	2L	16783911	16784665	0.32909147	0.544893095	0.00752769

S/N	Gene	ENSEMBL ID	Chr	Intron Start	Intron End	D10 IR ratio	D50 IR ratio	p-value
249	Mlc1	FBgn0002772	3R	27657955	27658066	0.08892471	0.158675381	0.034971805
250	mle	FBgn0002774	2R	5978677	5979318	0.2230266	0.374551568	0.044454907
251	Mlp60A	FBgn0259209	2R	24075069	24075198	0.04624766	0.218456475	2.16E-05
252	Mlp60A	FBgn0259209	2R	24075337	24075448	0.05653519	0.145275695	0.001429842
253	Mlp84B	FBgn0014863	3R	7115838	7116651	0.09505862	0.342947699	0.005007385
254	Mlp84B	FBgn0014863	3R	7115838	7117650	0.05589973	0.196587547	0.013032291
255	msps	FBgn0027948	3R	16059684	16059738	0.13551507	0.213351705	0.040838947
256	mus210	FBgn0004698	2R	15317147	15317208	0.04104094	0.255401918	0.026064388
257	mxt	FBgn0031637	2L	4845158	4845253	0.04434539	0.147192754	0.01443916
258	Nckx30C	FBgn0028704	2L	9744226	9746433	0.28586797	0.697281357	0.044726484
259	Nep3	FBgn0031081	X	19963341	19963954	0.30328332	0.607737109	0.035641854
260	Nf-YA	FBgn0035993	3L	9441966	9442123	0.2993124	0.603974144	0.044724284
261	Nxt1	FBgn0028411	2R	23811881	23811938	0.11676443	0.362160026	0.033703162
262	Obp49a	FBgn0050052	2R	12690548	12690609	0.07589035	0.135851757	0.026133208
263	Obp56a	FBgn0034468	2R	19697829	19698068	0.40294571	0.191441233	0.040986025
264	Obp69a	FBgn0011279	3L	12396739	12396792	0.06869193	0.229391051	0.001150639
265	osp	FBgn0003016	2L	14601798	14601850	0.14362376	0.295216665	0.0404713
266	ox	FBgn0011227	2R	12757231	12757297	0.09493417	0.184764084	0.023488625
267	per	FBgn0003068	X	2689439	2689503	0.06722034	0.197428197	0.049729725
268	Pfk	FBgn0003071	2R	10116657	10116736	0.05945912	0.118827838	0.029234105
269	Pgk	FBgn0250906	2L	2748255	2748319	0.09600898	0.163233741	0.0179765
270	Pi4KIIalpha	FBgn0037339	3R	5558301	5558366	0.06295319	0.244269653	0.00462743
271	Pi4KIIalpha	FBgn0037339	3R	5563345	5563541	0.0824442	0.290432622	0.016429503
272	Pif1A	FBgn0261015	3R	8798046	8799461	0.04589422	0.100342111	0.034383251
273	Pih1D1	FBgn0032455	2L	12713375	12713442	0.10380343	0.336075071	0.029245637
274	pio	FBgn0020521	2R	24596339	24596395	0.02098333	0.105469555	0.039720293
275	Pis	FBgn0030670	X	15703318	15703569	0.05964516	0.113867249	0.039333932
276	Pitslre	FBgn0016696	3L	20776053	20776173	0.05655866	0.112269307	0.041399709
277	PMCA	FBgn0259214	4	349146	349212	0.0353167	0.167608774	0.001155784
278	PMCA	FBgn0259214	4	349424	349480	0.05088741	0.157615103	0.000447124
279	PMCA	FBgn0259214	4	350043	350602	0.07448222	0.154720855	0.004744562
280	PMCA	FBgn0259214	4	350690	350764	0.08406378	0.173639834	0.005668832
281	PMCA	FBgn0259214	4	351209	351585	0.10750808	0.189441525	0.032106568
282	PMCA	FBgn0259214	4	351827	351883	0.06550979	0.178652244	0.00063655
283	PMCA	FBgn0259214	4	352126	352180	0.11549552	0.200802045	0.02504045
284	Pp1-87B	FBgn0004103	3R	12424516	12424610	0.10804227	0.173618787	0.011822315
285	pr	FBgn0003141	2L	20074128	20074190	0.06159106	0.179598969	0.007646751
286	Prm	FBgn0003149	3L	8744331	8744962	0.03035268	0.183633512	0.022581778
287	prt	FBgn0043005	3R	23698618	23698681	0.19806039	0.466033837	0.016164741
288	Psn	FBgn0019947	3L	20434032	20434086	0.13171681	0.258112579	0.042162369
289	Ptp99A	FBgn0004369	3R	29483072	29483129	0.06136418	0.205358727	0.020229039
290	rdx	FBgn0264493	3R	13970629	13970951	0.1852765	0.079577326	0.022725957
291	retm	FBgn0031814	2L	6451736	6451796	0.05295437	0.141049597	0.015997596
292	retn	FBgn0004795	2R	23642405	23642483	0.03734788	0.246052018	0.022332594
293	retn	FBgn0004795	2R	23651084	23651434	0.04357067	0.22935416	0.047115952

S/N	Gene	ENSEMBL ID	Chr	Intron Start	Intron End	D10 IR ratio	D50 IR ratio	p-value
294	Rh2	FBgn0003248	3R	18899576	18899672	0.08692538	0.22723908	0.001813572
295	RhoGEF2	FBgn0023172	2R	17036901	17036970	0.08727545	0.259021155	0.003585029
296	RyR	FBgn0011286	2R	8874862	8874934	0.11252663	0.282814867	0.020243335
297	Saf-B	FBgn0039229	3R	24818350	24818410	0.0410571	0.115703818	0.0421363
298	Saf-B	FBgn0039229	3R	24818700	24818802	0.0425262	0.128327382	0.027515894
299	sgll	FBgn0051472	3R	7810789	7810856	0.07503652	0.164544403	0.047954601
300	shrb	FBgn0086656	2R	9143000	9143058	0.06182194	0.131189501	0.038194858
301	SLC5A11	FBgn0031998	2L	8200960	8201016	0.24400713	0.446146156	0.003027342
302	SLC5A11	FBgn0031998	2L	8202409	8202467	0.27997975	0.546940385	0.002121614
303	sls	FBgn0086906	3L	2041078	2041138	0.02916626	0.168594922	0.04161827
304	sls	FBgn0086906	3L	2042015	2042090	0.03001299	0.269833062	0.027047689
305	sls	FBgn0086906	3L	2043106	2043240	0.02820647	0.142320471	0.005610893
306	sls	FBgn0086906	3L	2046164	2046281	0.05916172	0.187585273	0.044426704
307	sls	FBgn0086906	3L	2092559	2092620	0.04374469	0.238608887	0.023657229
308	Socs44A	FBgn0033266	2R	8128261	8128323	0.07962514	0.236671579	0.03456548
309	Sodh-2	FBgn0022359	3R	10877647	10877701	0.24877121	0.417197724	0.039619655
310	spas	FBgn0039141	3R	24039765	24039910	0.16541657	0.47987013	0.044765119
311	Spn	FBgn0010905	3L	2508573	2508638	0.1234814	0.24840084	0.019730358
312	sqa	FBgn0259678	2R	9719748	9719818	0.03531369	0.209888547	0.032569114
313	Srp9	FBgn0035827	3L	7936615	7936680	0.02656919	0.139075799	0.037507119
314	Ssk	FBgn0036945	3L	20189188	20189319	0.04788702	0.320152125	0.025382867
315	Ssrp	FBgn0010278	2R	23808732	23808809	0.07435949	0.25825882	0.023426249
316	Strn-Mlck	FBgn0265045	2R	15961188	15961312	0.0994861	0.321419962	0.026498153
317	sws	FBgn0003656	X	7957798	7957865	0.05862516	0.167377219	0.032284572
318	Syx6	FBgn0037084	2R	11220621	11220927	0.16755578	0.286121979	0.018308166
319	Tao	FBgn0031030	X	19572613	19572677	0.02373319	0.113873431	0.047407306
320	TBCB	FBgn0034451	2R	19486104	19486168	0.06066945	0.188408874	0.02831579
321	Tif-IA	FBgn0032988	2L	22250314	22250371	0.15560849	0.286506354	0.039446672
322	TppII	FBgn0020370	2R	13155210	13155268	0.06239299	0.182960099	0.032846578
323	tral	FBgn0041775	3L	12519070	12519143	0.03903789	0.125586256	0.017199478
324	trc	FBgn0003744	3L	19832876	19832933	0.06799255	0.156512607	0.025854684
325	Treh	FBgn0003748	2R	21085387	21085447	0.06125242	0.111823244	0.047607585
326	Trn-SR	FBgn0031456	2L	2760146	2760216	0.05322306	0.334893945	0.03121522
327	trol	FBgn0261451	X	2486242	2486926	0.18223672	0.380010422	0.045691563
328	Tsp42Eh	FBgn0033129	2R	7033718	7033778	0.227929	0.64086602	0.010701507
329	Tsp42Ek	FBgn0033133	2R	7043876	7043945	0.14471087	0.308923813	0.04213608
330	Tsp42En	FBgn0033135	2R	7053236	7053300	0.20312214	0.039076934	0.049641658
331	Tsp96F	FBgn0027865	3R	25875874	25875944	0.1248792	0.254722555	0.039869199
332	Ttc19	FBgn0032744	2L	19044317	19044369	0.15315593	0.322309412	0.027597872
333	tyf	FBgn0026083	X	4117895	4117963	0.23240779	0.474474264	0.019385178
334	Ubp64E	FBgn0016756	3L	5760609	5760674	0.04313067	0.11743143	0.036682572
335	Ubqn	FBgn0031057	X	19685910	19685989	0.04547143	0.125120032	0.035520522
336	UGP	FBgn0035978	3L	9352827	9352881	0.05569547	0.101573466	0.043609789
337	UGP	FBgn0035978	3L	9353228	9353512	0.32015278	0.537171261	0.016099603
338	Unc-89	FBgn0053519	2R	23997709	23997771	0.03416323	0.231581214	0.018489644

S/N	Gene	ENSEMBL ID	Chr	Intron Start	Intron End	D10 IR ratio	D50 IR ratio	p-value
339	Unc-89	FBgn0053519	2R	24000543	24000596	0.07882685	0.23741082	0.027938741
340	Unc-89	FBgn0053519	2R	24001950	24002008	0.03819949	0.283080833	0.015791255
341	Unc-89	FBgn0053519	2R	24002201	24002260	0.04118451	0.252007126	0.028843939
342	Unc-89	FBgn0053519	2R	24003890	24003956	0.03810387	0.343635842	0.00716289
343	Unc-89	FBgn0053519	2R	24006282	24006393	0.04649851	0.265303462	0.040065582
344	Unc-89	FBgn0053519	2R	24007766	24007833	0.04160715	0.256779774	0.029341336
345	Unc-89	FBgn0053519	2R	24012659	24012723	0.09717407	0.317932458	0.043097263
346	unc79	FBgn0038693	3R	19231918	19231975	0.07537936	0.142845578	0.038971599
347	up	FBgn0004169	X	13595242	13595319	0.03515195	0.180905071	3.33E-06
348	veil	FBgn0034225	2R	17480588	17480650	0.03992469	0.236399526	0.033735463
349	VhaM9.7-b	FBgn0028663	3L	21540082	21540152	0.08441238	0.166330451	0.01204874
350	VhaSFD	FBgn0027779	2L	16727488	16727629	0.04458292	0.102251415	0.034879647
351	Vps13	FBgn0033194	2R	7579410	7579475	0.03037083	0.153871354	0.040885189
352	Vps36	FBgn0086785	3L	13512081	13512139	0.11751109	0.322036924	0.04927689
353	wmd	FBgn0034876	2R	23545263	23545325	0.09524114	0.225822983	0.033801472
354	Wnk	FBgn0037098	3L	21544268	21544325	0.09055956	0.256404286	0.008086136
355	wupA	FBgn0004028	X	18106931	18109469	0.23226732	0.348787315	0.015588618
356	wupA	FBgn0004028	X	18106931	18111937	0.38507512	0.652952277	0.002576178
357	wupA	FBgn0004028	X	18109570	18113257	0.17441947	0.304857315	0.004680056
358	wupA	FBgn0004028	X	18112038	18113257	0.3714164	0.668489342	0.002364673
359	yuri	FBgn0045842	2L	15263664	15263769	0.38102595	0.174630083	0.017689166
360	Zasp66	FBgn0035917	3L	8626510	8626960	0.0806112	0.284457161	0.001191451
361	Zasp66	FBgn0035917	3L	8630376	8630516	0.11531603	0.361352786	0.022341198
362	Zasp66	FBgn0035917	3L	8635081	8635680	0.15360273	0.228022838	0.027499061
363	Zasp66	FBgn0035917	3L	8635752	8637438	0.2109349	0.309327234	0.020708319
364	Zasp66	FBgn0035917	3L	8637598	8638156	0.14918192	0.212774881	0.045055201
365	Zasp66	FBgn0035917	3L	8638195	8638266	0.08872298	0.159107576	0.000734858
366	Zip102B	FBgn0039902	4	313298	313356	0.12597672	0.025525686	0.043043106
367	zyd	FBgn0265767	X	23023431	23030189	0.20981114	0.428412668	0.03244298
368	zyd	FBgn0265767	X	23028597	23030189	0.38097802	0.633434857	0.042649676
