# Supplementary material for: Increased intron retention is a post‐transcriptional signature associated with progressive aging and Alzheimer’s disease
Source: Aging Cell. 2019 Mar 13;18(3):e12928. doi: 10.1111/acel.12928 (PMC6516162; doi:10.1111/acel.12928)
Supplement: Supplementary file 3 [file ACEL-18-e12928-s003.pdf]

TableS2: Mean FPKM values of differential IR genes across different ages in fly

geneID	GeneSym	Coord	D10_mean	D20_mean	D30_mean	D50_mean
FBgn0000228	Bsg25D	2L:5271727-5278548	16.85	20.17	20.53	19.96
FBgn0000279	CecC	3R:30216483-30216938	2.66	15.85	26.14	102.33
FBgn0000316	cin	X:255673-258469	20.73	21.06	22.21	19.61
FBgn0000319	Chc	X:15827985-15835490	89.82	93.59	100.35	98.94
FBgn0000454	Dip-B	3R:13782287-13785676	60.01	51.64	57.11	59.09
FBgn0000464	Lar	2L:19586622-19732069	50.27	55.80	54.96	53.33
FBgn0000482	dor	X:1665051-1668451	8.05	7.61	8.57	7.59
FBgn0000667	Actn	X:2023821-2042311	33.34	37.39	38.45	39.74
FBgn0001137	grk	2L:8431085-8433598	13.62	16.44	18.77	19.97
FBgn0001202	hk	2L:19030843-19033498	36.50	37.08	39.15	34.33
FBgn0002431	hyd	3R:9714622-9725615	29.38	35.96	36.17	38.15
FBgn0002566	lt	2L:22924794-22943135	37.09	44.87	40.30	41.08
FBgn0002643	mam	2R:13991202-14060356	18.61	21.53	20.84	23.13
FBgn0002772	Mlc1	3R:27657077-27660177	97.82	111.94	134.36	150.46
FBgn0002774	mle	2R:5974856-5980778	40.50	44.51	50.29	50.70
FBgn0002891	mus205	2R:7793624-7801044	2.43	2.84	3.21	3.81
FBgn0003011	ort	3R:19659747-19663672	9.60	9.99	9.21	9.56
FBgn0003016	osp	2L:14599195-14689340	13.79	16.59	15.28	14.51
FBgn0003068	per	X:2685579-2692780	13.39	11.62	11.28	13.01
FBgn0003071	Pfk	2R:10109739-10117457	88.16	81.43	84.63	76.35
FBgn0003134	Pp1alpha-96A	3R:24518269-24522331	83.77	86.25	78.17	92.83
FBgn0003141	pr	2L:20073718-20075458	42.67	38.24	37.01	33.09
FBgn0003149	Prm	3L:8732857-8745310	34.72	38.76	49.86	61.47
FBgn0003218	rdgB	X:13762751-13779488	48.55	57.09	54.15	62.30
FBgn0003248	Rh2	3R:18899422-18901019	276.88	205.10	166.62	45.71
FBgn0003656	sws	X:7956819-7968236	34.39	38.43	39.47	36.66
FBgn0003744	trc	3L:19830863-19834304	36.75	39.03	39.23	34.64
FBgn0003748	Treh	2R:21074017-21088945	159.71	154.94	133.51	110.54
FBgn0003890	betaTub97EF	3R:27967007-27988384	33.24	34.51	35.01	34.93
FBgn0004028	wupA	X:18105567-18116922	104.19	122.65	143.36	143.50
FBgn0004103	Pp1-87B	3R:12423620-12425917	102.18	108.59	113.08	108.75
FBgn0004169	up	X:13593473-13602578	84.25	90.97	116.78	121.65
FBgn0004362	HmgD	2R:21701457-21716727	32.03	36.56	42.13	40.95
FBgn0004369	Ptp99A	3R:29377645-29487131	27.43	32.00	31.95	33.66
FBgn0004507	GlyP	2L:2130812-2137296	138.37	129.15	130.27	127.08
FBgn0004598	Fur2	X:16373085-16381782	39.54	45.80	46.19	45.57
FBgn0004698	mus210	2R:15311509-15318239	30.82	34.14	32.00	31.90
FBgn0004784	inaC	2R:16898534-16901575	359.82	333.08	359.21	306.41
FBgn0004795	retn	2R:23632609-23654820	15.13	15.95	18.95	16.34
FBgn0004797	mdy	2L:16816659-16826964	37.44	38.27	36.89	36.12
FBgn0004914	Hnf4	2L:8687225-8709611	30.29	32.14	33.90	34.02
FBgn0004919	gol	2R:25072626-25084347	68.98	67.68	60.80	53.20
FBgn0005666	bt	4:724399-776474	19.97	23.82	29.27	32.80
FBgn0010051	Itp-r83A	3R:5517116-5539411	21.81	24.87	25.93	25.52

geneID	GeneSym	Coord	D10_mean	D20_mean	D30_mean	D50_mean
FBgn0010235	Klc	3L:12746615-12750056	40.69	43.85	46.64	46.82
FBgn0010278	Ssrp	2R:23806360-23809004	7.89	8.38	9.79	11.74
FBgn0010434	cora	2R:19229062-19243751	54.79	63.06	65.20	66.19
FBgn0010438	mtSSB	3R:16204193-16205250	9.11	12.39	12.76	13.52
FBgn0010482	l(2)01289	2R:6721100-6740672	12.81	15.32	16.14	17.18
FBgn0010551	Phb2	2R:18819466-18822250	64.85	68.44	75.80	82.70
FBgn0010750	atms	3R:4750034-4777490	12.65	12.20	12.60	11.49
FBgn0010905	Spn	3L:2505244-2554292	23.17	26.86	26.18	24.49
FBgn0011206	bol	3L:9097628-9134405	42.54	40.92	36.80	46.94
FBgn0011227	ox	2R:12757132-12757529	302.99	254.96	219.92	222.62
FBgn0011279	Obp69a	3L:12396170-12396957	93.59	74.78	64.86	48.78
FBgn0011286	RyR	2R:8860363-8888097	17.93	17.53	19.40	18.26
FBgn0011296	l(2)efl	2R:23684650-23685543	52.29	72.36	108.39	132.59
FBgn0011455	l(3)neo18	3L:12137495-12138584	142.69	133.71	146.81	139.31
FBgn0011745	Arp1	3R:12691742-12694232	50.15	48.49	50.95	49.25
FBgn0013762	Cdk5	2R:15569527-15571417	19.35	18.93	19.60	18.65
FBgn0013972	Gycalpha99B	3R:29682502-29685758	50.11	37.61	39.49	27.27
FBgn0014863	Mlp84B	3R:7112849-7117732	46.89	52.80	66.35	67.45
FBgn0014906	Hydr2	2L:3163644-3169462	53.38	60.51	65.55	60.58
FBgn0015034	Cyp4e1	2R:8447450-8449745	5.70	4.60	4.08	3.33
FBgn0015513	mbc	3R:23781764-23801634	21.88	25.91	27.16	32.30
FBgn0015571	alpha-Est3	3R:7538971-7541631	16.19	15.13	17.07	23.13
FBgn0015791	Rab14	2L:14355144-14358764	97.46	97.05	103.19	93.47
FBgn0016032	lbm	2R:7049237-7051964	15.77	16.73	18.19	17.31
FBgn0016696	Pitslre	3L:20771527-20776931	85.85	96.11	99.86	96.32
FBgn0016756	Ubp64E	3L:5752972-5761886	39.68	48.18	53.46	54.58
FBgn0017578	Max	3L:19262231-19264184	16.57	19.68	19.94	19.83
FBgn0019947	Psn	3L:20432467-20435156	24.17	25.58	27.33	26.58
FBgn0020370	TppII	2R:13152884-13159141	20.32	20.90	22.12	21.53
FBgn0020521	pio	2R:24581244-24597862	51.21	53.84	54.24	52.66
FBgn0020647	KrT95D	3R:23997537-24031827	35.77	40.18	43.16	39.77
FBgn0022029	l(2)k01209	2R:17420406-17425679	18.17	19.29	21.47	21.74
FBgn0022359	Sodh-2	3R:10876383-10878257	29.49	27.98	20.80	19.36
FBgn0023172	RhoGEF2	2R:17027189-17044653	33.46	41.82	43.46	48.70
FBgn0023213	eIF4G	4:915296-930715	62.59	80.84	75.25	92.52
FBgn0023458	Rbcn-3A	X:6240744-6255487	36.67	39.19	42.69	39.91
FBgn0023549	Mct1	X:2189187-2205373	14.59	18.69	19.22	20.71
FBgn0024689	fws	2L:17478441-17481029	16.24	16.01	17.77	15.52
FBgn0024811	Crk	4:210223-213165	116.67	138.88	102.32	98.80
FBgn0025592	Gyk	3L:202097-205844	42.83	41.28	37.81	37.61
FBgn0025684	MFS18	2L:4446669-4448635	17.16	15.51	18.21	15.65
FBgn0025687	LKR	2L:7791174-7795959	12.51	13.34	14.07	18.81
FBgn0025697	santa-maria	2L:7445354-7451244	73.82	77.40	77.80	84.98
FBgn0026083	tyf	X:4117019-4130016	17.19	20.59	20.34	21.64
FBgn0026086	Adar	X:1773724-1788065	58.18	62.91	55.17	56.58

geneID	GeneSym	Coord	D10_mean	D20_mean	D30_mean	D50_mean
FBgn0026439	Eaat1	2L:9333566-9341925	162.65	157.53	128.84	119.68
FBgn0026666	l(1)G0136	X:15734269-15735780	34.33	33.50	34.30	28.46
FBgn0027074	CG17324	2L:18819343-18822573	3.47	5.17	5.80	6.39
FBgn0027529	CG8920	2R:20319179-20325973	57.13	66.18	63.19	59.27
FBgn0027565	CG5498	3L:20382404-20384109	16.42	16.97	18.11	18.32
FBgn0027596	CG10249	2R:14901474-14928425	32.67	33.34	33.29	31.91
FBgn0027657	glob1	3R:15896925-15904521	124.70	134.50	127.48	212.73
FBgn0027779	VhaSFD	2L:16722882-16727826	119.97	118.60	125.59	124.93
FBgn0027865	Tsp96F	3R:25872899-25881427	28.85	34.23	31.15	35.83
FBgn0027948	msps	3R:16050800-16060675	39.56	42.60	43.66	39.37
FBgn0028411	Nxt1	2R:23811320-23811974	10.05	10.09	12.61	14.18
FBgn0028425	JhI-21	2L:12051754-12056042	17.08	17.93	20.97	24.27
FBgn0028427	Ilk	3L:21216900-21219240	32.46	34.50	37.62	38.17
FBgn0028429	I-2	3L:9834366-9836805	19.68	20.95	23.11	24.22
FBgn0028552	gammaSnap1	2R:24047144-24048903	67.20	61.63	61.48	53.38
FBgn0028646	aralar1	3R:30445634-30455867	55.80	58.54	61.39	78.80
FBgn0028663	VhaM9.7-b	3L:21539861-21541215	94.44	89.05	104.18	110.45
FBgn0028670	Vha100-2	3R:18390322-18399169	60.86	57.11	65.18	59.75
FBgn0028703	Nhe3	2L:6677834-6684364	84.41	93.09	85.65	77.90
FBgn0028704	Nckx30C	2L:9711511-9746495	74.46	73.67	66.10	74.25
FBgn0029088	disp	3R:5849696-5856311	13.73	14.65	16.01	15.22
FBgn0029174	FKBP59	2L:9888004-9890119	36.78	38.47	38.22	34.13
FBgn0029688	lva	X:3955542-3965327	24.24	29.49	32.85	33.51
FBgn0029771	CG12730	X:5516607-5518771	25.78	27.03	28.61	25.88
FBgn0029820	CG16721	X:5879868-5883391	42.21	38.27	36.68	31.73
FBgn0029824	CG3726	X:5917096-5941852	11.85	12.61	14.07	13.58
FBgn0030013	GIIIspla2	X:8157627-8159348	24.04	29.74	26.11	29.82
FBgn0030073	CG10962	X:8972907-8974255	14.85	18.88	14.54	15.27
FBgn0030081	CG7246	X:9078822-9081057	3.44	3.79	4.79	5.00
FBgn0030228	BTBD9	X:10768536-10772154	12.04	12.92	14.10	14.05
FBgn0030286	CG1657	X:11325384-11334071	28.39	29.14	32.72	30.67
FBgn0030309	CG1572	X:11555049-11558654	56.00	69.19	85.94	121.46
FBgn0030339	Cyp28c1	X:11839607-11841503	7.50	5.84	5.41	3.92
FBgn0030348	CG10352	X:11875305-11877168	12.34	10.69	10.15	9.15
FBgn0030484	GstT4	X:13404748-13409323	56.68	53.42	59.83	63.37
FBgn0030528	CG11095	X:13811683-13812870	4.77	5.37	5.69	4.93
FBgn0030555	Fbxl4	X:14234412-14237923	34.69	39.66	45.96	40.48
FBgn0030670	Pis	X:15701450-15703770	257.03	237.35	232.47	193.07
FBgn0030685	Graf	X:15751673-15760733	22.74	26.43	25.06	26.54
FBgn0030745	CG4239	X:16385277-16388702	24.69	24.26	25.01	21.64
FBgn0030791	CG9132	X:16796901-16800710	48.10	52.30	51.09	48.61
FBgn0030887	CG6867	X:18023869-18028261	13.10	14.18	15.06	12.34
FBgn0030897	Frq1	X:18155368-18174744	27.69	26.04	21.73	24.52
FBgn0030954	CCKLR-17D3	X:18764482-18774027	4.90	4.96	4.52	3.72
FBgn0031021	CG12203	X:19492420-19493337	69.92	72.91	74.93	64.13

geneID	GeneSym	Coord	D10_mean	D20_mean	D30_mean	D50_mean
FBgn0031030	Tao	X:19569720-19579800	54.28	70.29	68.33	72.56
FBgn0031057	Ubqn	X:19683807-19687658	47.77	52.58	57.05	56.04
FBgn0031081	Nep3	X:19961296-19969323	22.67	23.58	23.43	19.04
FBgn0031092	CG9577	X:20166672-20168080	21.52	19.54	19.34	17.35
FBgn0031110	Obp19b	X:20416732-20417812	32.44	27.25	21.58	13.23
FBgn0031304	CG4552	2L:1129315-1132411	29.30	31.31	36.29	32.49
FBgn0031322	CG5001	2L:1185756-1196807	14.67	14.77	17.45	17.00
FBgn0031453	Bacc	2L:2752444-2757975	285.44	322.45	305.90	318.66
FBgn0031456	Trn-SR	2L:2759923-2763889	5.89	5.71	6.21	6.37
FBgn0031604	Elp3	2L:4444731-4446765	8.65	9.83	11.85	11.48
FBgn0031632	CG15628	2L:4821773-4828945	31.75	37.40	33.55	29.44
FBgn0031637	mxt	2L:4841481-4847295	52.37	59.55	67.45	64.67
FBgn0031662	CG3792	2L:4977311-4978567	30.76	32.34	33.80	32.59
FBgn0031734	CG11147	2L:5733674-5741244	94.06	99.05	84.21	78.80
FBgn0031814	retm	2L:6448463-6455929	75.93	71.59	64.73	63.36
FBgn0031869	CG18304	2L:6932544-6942814	3.02	3.56	3.89	3.98
FBgn0031937	CG13795	2L:7723044-7726298	33.45	35.27	33.73	43.87
FBgn0031952	cdc14	2L:7801667-7810703	43.30	49.33	50.39	43.80
FBgn0031970	CG7227	2L:7994387-7998151	1.46	1.89	4.19	2.57
FBgn0031998	SLC5A11	2L:8200464-8205132	27.59	28.36	23.14	16.54
FBgn0032021	CG7781	2L:8321279-8325409	108.77	102.12	91.89	78.03
FBgn0032023	CG14274	2L:8338286-8342056	25.94	24.64	22.21	18.74
FBgn0032026	CG7627	2L:8355677-8362469	17.69	18.42	19.38	19.56
FBgn0032036	CG13384	2L:8384454-8388804	37.83	37.66	37.61	33.73
FBgn0032109	CG17005	2L:9327743-9330332	18.54	9.85	3.31	1.92
FBgn0032129	jp	2L:9547719-9566750	34.17	39.70	39.95	39.31
FBgn0032156	CG13124	2L:9903278-9908633	84.65	99.67	102.92	109.03
FBgn0032196	CG5708	2L:10224456-10226808	38.59	39.34	36.49	34.35
FBgn0032233	dpr19	2L:10376167-10379905	26.74	25.71	24.63	18.19
FBgn0032297	CG17124	2L:10744074-10756325	57.56	55.27	45.30	43.31
FBgn0032381	Mal-B1	2L:11847225-11849169	10.51	10.24	8.56	8.97
FBgn0032455	Pih1D1	2L:12713275-12721843	28.19	30.63	31.10	28.77
FBgn0032470	CG5142	2L:13106806-13109468	5.37	8.52	14.38	12.26
FBgn0032744	Ttc19	2L:19043534-19045148	13.98	14.54	15.65	13.79
FBgn0032797	CG10186	2L:19508952-19517204	232.46	196.64	192.09	136.34
FBgn0032836	CG10680	2L:19964135-19965702	155.96	170.93	199.61	247.27
FBgn0032859	Arpc2	2L:20417056-20423027	19.76	22.95	24.86	24.77
FBgn0032988	Tif-IA	2L:22249875-22258260	31.04	36.52	35.28	33.92
FBgn0033000	CG14464	2R:4829245-4830587	64.04	59.78	57.48	59.35
FBgn0033015	d4	2R:5259003-5295965	31.82	35.03	36.12	33.49
FBgn0033104	CG15237	2R:6809043-6809766	13.97	13.84	14.08	13.10
FBgn0033129	Tsp42Eh	2R:7033561-7034920	6.20	7.75	9.70	11.46
FBgn0033133	Tsp42Ek	2R:7042655-7044710	13.63	14.61	12.84	15.16
FBgn0033135	Tsp42En	2R:7052746-7054399	8.23	11.07	16.70	18.77
FBgn0033194	Vps13	2R:7566808-7580675	17.76	20.95	22.96	26.51

geneID	GeneSym	Coord	D10_mean	D20_mean	D30_mean	D50_mean
FBgn0033205	CG2064	2R:7665827-7667343	22.19	23.63	26.79	30.87
FBgn0033247	Nup44A	2R:7988461-7991283	18.86	19.74	18.73	20.56
FBgn0033266	Socs44A	2R:8127574-8129476	15.19	14.08	16.00	16.37
FBgn0033382	Hydr1	2R:9132479-9135453	35.50	33.55	35.87	30.35
FBgn0033486	dmpd	2R:10117693-10121451	21.22	22.45	22.92	19.61
FBgn0033639	CG9003	2R:11641476-11651616	25.49	29.16	26.67	25.35
FBgn0033672	rho-7	2R:11986512-11988787	18.26	17.88	20.23	18.24
FBgn0033734	CG8520	2R:12422930-12425459	24.58	25.68	27.23	23.32
FBgn0033809	CG4630	2R:13215658-13219247	12.11	10.93	11.34	8.75
FBgn0033844	bbc	2R:13486633-13494862	61.04	64.85	69.25	66.67
FBgn0033872	CG6329	2R:13826370-13833503	162.51	145.70	115.25	114.68
FBgn0034002	CG8079	2R:15218378-15224795	16.10	19.40	21.03	20.21
FBgn0034062	CG8388	2R:16009400-16011438	3.80	3.94	4.49	4.62
FBgn0034067	CG8399	2R:16017736-16024627	32.04	33.75	37.12	37.66
FBgn0034218	CG18467	2R:17440296-17442483	11.91	10.16	10.41	8.26
FBgn0034225	veil	2R:17478157-17482118	13.73	12.61	13.42	10.19
FBgn0034275	CG5002	2R:17750292-17753214	10.76	13.12	16.27	23.16
FBgn0034308	CG10915	2R:18160719-18164746	22.93	25.59	24.42	21.96
FBgn0034417	CG15117	2R:19122230-19129300	9.55	11.38	12.11	16.80
FBgn0034418	CG15118	2R:19133726-19137169	56.77	58.64	61.12	53.55
FBgn0034420	CG10737	2R:19164142-19178223	70.24	80.52	87.94	82.38
FBgn0034451	TBCB	2R:19485493-19486837	17.07	17.95	18.71	17.43
FBgn0034468	Obp56a	2R:19697722-19698508	4.40	11.39	12.69	20.72
FBgn0034598	CG4266	2R:21161712-21168659	18.26	21.68	22.66	21.87
FBgn0034605	CG15661	2R:21215434-21217779	46.46	50.42	56.21	64.33
FBgn0034655	CG10307	2R:21688772-21690253	9.40	9.90	8.59	7.15
FBgn0034660	lox2	2R:21789622-21791580	6.10	4.43	3.12	3.29
FBgn0034688	CG11474	2R:22071233-22073443	20.55	18.65	18.97	18.43
FBgn0034876	wmd	2R:23544822-23548151	15.51	17.79	19.04	18.32
FBgn0034897	Sesn	2R:23713899-23734846	44.90	53.59	58.18	65.08
FBgn0034943	Fmo-1	2R:23962955-23964641	4.07	4.16	4.69	5.60
FBgn0034951	CG3860	2R:23992137-23995055	34.27	35.10	36.57	32.03
FBgn0034958	CG3907	2R:24019897-24022298	47.56	47.00	47.29	44.22
FBgn0035026	Fcp1	2R:24567986-24571821	11.11	11.04	10.95	8.78
FBgn0035050	ST6Gal	2R:24691421-24694386	9.38	12.29	13.13	12.09
FBgn0035060	Eps-15	2R:24768313-24775265	48.19	46.57	48.61	41.55
FBgn0035064	Aats-tyr-m	2R:24781725-24783500	6.44	6.21	6.46	6.44
FBgn0035146	CG13893	3L:599093-604008	19.29	19.47	17.13	13.49
FBgn0035147	Gale	3L:648554-651311	55.97	66.05	65.22	73.95
FBgn0035205	CG2469	3L:1307223-1311805	29.17	29.51	31.69	28.15
FBgn0035227	CG12090	3L:1523413-1534034	56.09	61.12	70.22	79.08
FBgn0035232	CG12099	3L:1555362-1558484	52.83	54.67	53.29	55.75
FBgn0035252	CG7970	3L:1652749-1654238	38.47	38.00	38.36	35.27
FBgn0035287	CG13937	3L:1876550-1883041	26.94	27.97	24.38	20.08
FBgn0035388	CG2162	3L:3035195-3040469	18.61	20.85	20.02	18.92

geneID	GeneSym	Coord	D10_mean	D20_mean	D30_mean	D50_mean
FBgn0035416	gry	3L:3199089-3211348	12.97	13.69	14.44	14.47
FBgn0035445	CG12014	3L:3378267-3380083	7.11	7.22	9.21	9.58
FBgn0035526	CG1316	3L:4283850-4286865	38.84	44.91	42.79	41.35
FBgn0035528	CG15012	3L:4291207-4292246	87.34	82.57	90.36	83.68
FBgn0035649	CG10483	3L:5917904-5921316	14.32	12.15	11.77	9.33
FBgn0035770	pst	3L:7357274-7360263	94.38	92.95	99.98	143.83
FBgn0035827	Srp9	3L:7936372-7937258	26.12	27.57	27.08	26.91
FBgn0035903	CG6765	3L:8512766-8518738	11.45	12.77	13.57	11.59
FBgn0035917	Zasp66	3L:8624370-8638813	92.02	95.76	106.43	106.11
FBgn0035921	CG13305	3L:8644915-8646822	4.91	2.96	3.02	2.26
FBgn0035978	UGP	3L:9350866-9358935	125.42	133.49	142.25	171.14
FBgn0035986	CG4022	3L:9406178-9410832	6.04	7.47	7.25	6.98
FBgn0035989	CG3967	3L:9413595-9426758	37.19	36.89	36.00	32.43
FBgn0035993	Nf-YA	3L:9440055-9442267	7.05	8.05	8.88	8.66
FBgn0036133	CG7638	3L:11109689-11112117	22.35	21.12	23.04	18.96
FBgn0036141	wls	3L:11166619-11169625	16.70	16.92	17.67	15.60
FBgn0036145	CG7607	3L:11199619-11200587	70.88	52.37	47.06	24.04
FBgn0036146	CG14141	3L:11201350-11202158	32.15	27.39	22.01	12.33
FBgn0036183	CG6083	3L:11624527-11626125	7.66	8.17	7.39	5.87
FBgn0036249	CG11560	3L:12120018-12121185	6.22	6.91	8.06	7.46
FBgn0036286	CG10616	3L:12471727-12474428	14.57	16.33	17.89	17.17
FBgn0036369	CG10089	3L:13465551-13474787	18.10	19.20	19.07	15.88
FBgn0036515	CG12304	3L:15583637-15585347	23.62	23.56	26.37	29.54
FBgn0036534	DCP2	3L:15818733-15826423	31.07	36.53	34.04	36.52
FBgn0036545	GXIVsPLA2	3L:15955095-15956465	17.71	17.14	21.17	20.90
FBgn0036569	CG5414	3L:16100279-16103482	4.08	5.04	4.63	4.73
FBgn0036875	CG9449	3L:19492052-19497723	33.13	31.13	35.91	32.40
FBgn0036945	Ssk	3L:20188521-20189779	12.79	9.66	10.42	10.03
FBgn0036986	CG5282	3L:20424423-20426071	24.05	21.37	21.91	17.79
FBgn0037060	CG10508	3L:21210214-21216051	27.63	28.76	27.47	23.32
FBgn0037084	Syx6	2R:11219538-11222480	45.69	46.10	48.36	44.94
FBgn0037098	Wnk	3L:21543849-21554928	23.31	27.09	28.01	30.06
FBgn0037295	dpr16	3R:5150907-5170121	5.53	6.06	6.34	5.90
FBgn0037313	CG1161	3R:5355091-5356748	76.82	70.49	72.94	67.25
FBgn0037339	Pi4KIIalpha	3R:5557662-5563652	25.25	28.87	29.13	30.01
FBgn0037344	CG2926	3R:5579300-5588380	16.17	18.78	19.48	19.66
FBgn0037382	Hpr1	3R:5818793-5821440	10.81	9.76	11.22	9.91
FBgn0037513	pyd3	3R:7747912-7750079	29.87	25.14	25.23	24.17
FBgn0037530	CG2943	3R:7903029-7907363	43.60	44.60	47.61	46.81
FBgn0037574	Coq2	3R:8314783-8316543	6.11	7.07	7.50	6.85
FBgn0037655	CG11984	3R:9010281-9014909	50.25	52.53	56.72	52.73
FBgn0037680	CG8121	3R:9335169-9337459	20.52	22.76	23.72	24.22
FBgn0037709	CG8199	3R:9555784-9557776	13.38	13.84	15.16	16.79
FBgn0037756	CG8507	3R:9791482-9793098	30.43	35.94	36.22	40.42
FBgn0037892	mRpL40	3R:11407917-11408756	13.72	12.72	14.72	13.80

geneID	GeneSym	Coord	D10_mean	D20_mean	D30_mean	D50_mean
FBgn0037913	fabp	3R:11564057-11567054	511.83	441.16	482.46	455.13
FBgn0037950	HisCl1	3R:11787175-11789393	11.57	12.18	13.45	11.62
FBgn0038105	yellow-f2	3R:12993394-12995147	31.51	30.69	28.33	29.71
FBgn0038149	GILT1	3R:13398525-13401862	81.78	82.75	96.09	98.56
FBgn0038179	CG9312	3R:13765755-13767103	37.58	33.54	30.45	34.93
FBgn0038181	CG9297	3R:13771494-13779317	51.74	48.12	44.67	33.67
FBgn0038199	CCHa1	3R:14092503-14096835	4.78	5.43	6.00	4.64
FBgn0038224	CG3321	3R:14326946-14327735	241.60	209.96	226.79	200.72
FBgn0038294	Mf	3R:15170624-15181510	247.26	284.89	317.52	306.18
FBgn0038302	CG4210	3R:15213973-15214845	10.61	10.98	11.95	14.18
FBgn0038564	CG7785	3R:18021599-18023689	13.68	14.09	14.17	12.67
FBgn0038652	CG7720	3R:18759235-18795169	21.26	25.78	27.09	21.91
FBgn0038693	unc79	3R:19230763-19246130	62.82	61.22	66.37	57.50
FBgn0038912	CG6656	3R:21875292-21877788	50.27	58.49	61.15	49.46
FBgn0038925	Cchl	3R:22025974-22027480	51.84	47.08	53.61	46.68
FBgn0039040	CG13833	3R:23033283-23035563	78.12	54.63	42.93	30.31
FBgn0039094	CG10184	3R:23602506-23605495	65.29	83.53	59.23	54.78
FBgn0039102	SPE	3R:23686254-23688147	135.02	128.43	144.34	172.54
FBgn0039135	CG13603	3R:23985212-23986569	62.88	56.54	62.07	56.47
FBgn0039141	spas	3R:24038214-24042982	13.13	12.78	13.50	13.42
FBgn0039153	CG5463	3R:24128048-24130255	7.19	7.04	7.14	6.30
FBgn0039178	CG6356	3R:24283954-24288617	39.09	38.40	31.20	26.91
FBgn0039215	CG6695	3R:24655159-24659379	9.62	12.06	13.44	12.46
FBgn0039229	Saf-B	3R:24817463-24822350	48.55	54.22	52.15	45.42
FBgn0039241	CG11089	3R:24877044-24881478	107.20	147.25	138.04	248.83
FBgn0039258	beta4GalT7	3R:25023255-25024482	6.66	5.31	6.71	5.76
FBgn0039259	CG11781	3R:25024472-25025017	24.31	21.58	22.21	21.39
FBgn0039316	CG11893	3R:25289888-25291366	26.90	35.27	38.51	62.96
FBgn0039357	CG4743	3R:25679241-25680550	10.34	8.72	9.81	8.98
FBgn0039494	grass	3R:27157947-27159781	24.19	25.51	28.44	37.17
FBgn0039529	CG5612	3R:27623358-27626011	21.21	20.92	22.48	23.67
FBgn0039562	Gp93	3R:27948710-27952151	45.85	55.04	65.69	70.42
FBgn0039732	CG15525	3R:29996960-29997676	9.24	8.68	8.05	7.04
FBgn0039789	CG9717	3R:30584019-30587302	17.59	15.67	22.25	17.21
FBgn0039864	CG11550	3R:31678146-31679880	68.91	59.70	54.70	48.52
FBgn0039890	CG2316	4:184075-193829	104.01	115.07	99.81	95.71
FBgn0039897	CG1674	4:230729-245903	40.62	49.45	42.87	38.90
FBgn0039902	Zip102B	4:310929-314087	43.87	50.31	45.77	45.39
FBgn0039907	lgs	4:436956-443911	27.19	31.69	31.23	30.31
FBgn0039908	Asator	4:469408-488994	81.75	92.41	82.61	78.10
FBgn0039916	CG9935	4:636130-646556	34.55	45.12	39.79	35.29
FBgn0039969	Fis1	2R:5603586-5604834	140.19	144.41	127.06	127.64
FBgn0039994	conu	2R:4744347-4758048	45.23	52.21	46.58	49.97
FBgn0040230	dbo	3L:15826887-15832004	26.01	29.86	28.55	27.26
FBgn0040337	CG3021	X:1026790-1028290	7.56	7.31	7.94	8.37

geneID	GeneSym	Coord	D10_mean	D20_mean	D30_mean	D50_mean
FBgn0040394	CG16903	X:2078342-2080405	14.92	15.20	17.52	14.78
FBgn0040588	CG13841	3R:22766731-22767699	39.18	44.19	57.99	63.94
FBgn0041205	key	2R:24785513-24787411	21.47	24.06	25.13	23.64
FBgn0041210	HDAC4	X:13262686-13285632	21.01	25.84	25.58	28.22
FBgn0041775	tral	3L:12515058-12520954	48.15	58.97	57.27	58.85
FBgn0042135	CG18812	2R:7716806-7735622	153.26	173.86	173.24	175.45
FBgn0043005	prt	3R:23696371-23699027	8.51	6.90	9.06	6.98
FBgn0043070	MESK2	2R:21502096-21517241	137.05	140.79	137.11	139.26
FBgn0043792	CG30427	2R:24928746-24937477	36.07	34.91	28.36	25.45
FBgn0044048	Ilp5	3L:9823348-9823897	27.58	22.84	13.49	4.55
FBgn0045842	yuri	2L:15257266-15264692	10.90	14.02	14.46	18.79
FBgn0046247	CG5938	3R:27274793-27277418	44.23	42.76	42.00	38.02
FBgn0050021	metro	2R:11226457-11232516	30.80	28.67	30.17	22.60
FBgn0050051	CG30051	2R:12436119-12436990	13.62	13.54	12.18	11.98
FBgn0050052	Obp49a	2R:12690058-12690955	131.75	151.35	143.31	122.31
FBgn0050109	CG30109	2R:17670081-17670748	11.35	9.89	11.89	13.80
FBgn0050163	Cpr60D	2R:24662681-24663406	15.92	13.20	10.39	7.45
FBgn0050438	CG30438	2R:5496673-5549543	17.51	18.66	13.32	10.70
FBgn0050491	CG30491	2R:7655071-7656770	9.78	10.45	12.11	9.56
FBgn0050503	CG30503	2R:7443823-7444627	89.47	76.60	70.08	71.84
FBgn0051103	CG31103	3R:25219317-25221444	48.87	46.56	44.45	47.02
FBgn0051109	CG31109	3R:25053368-25054653	10.21	10.25	11.02	9.43
FBgn0051191	CG31191	3R:20909212-20945530	7.61	9.30	9.28	8.84
FBgn0051321	CG31321	3R:14310361-14314412	18.41	19.70	18.01	15.27
FBgn0051361	dpr17	3R:12094875-12111420	8.39	9.93	9.83	9.25
FBgn0051472	sgll	3R:7810531-7811912	35.51	33.91	34.63	36.04
FBgn0051619	CG31619	2L:21684105-21729051	35.13	37.46	34.32	30.54
FBgn0052068	CG32068	3L:10663375-10664435	77.61	72.30	73.19	84.06
FBgn0052085	CG32085	3L:11650516-11680344	17.11	19.28	19.54	17.83
FBgn0052523	CG32523	X:21217877-21218835	83.74	73.24	67.05	28.59
FBgn0053051	CG33051	3L:17426285-17427130	5.27	6.80	5.31	6.85
FBgn0053110	CG33110	3R:22570187-22580392	44.80	46.99	35.82	45.14
FBgn0053519	Unc-89	2R:23995542-24014254	14.01	15.78	18.09	15.82
FBgn0053774	CG33774	2R:9399752-9400329	53.67	56.63	51.50	45.68
FBgn0058006	CG40006	2L:22622416-22756349	44.80	41.00	29.39	27.54
FBgn0058196	Maf1	2R:1512809-1527095	113.91	122.01	107.67	95.71
FBgn0062449	CG13197	2R:11691284-11697785	14.60	17.78	16.80	16.41
FBgn0067779	dbr	2L:66481-71390	19.41	22.83	23.99	23.69
FBgn0085377	CG34348	X:11339872-11344734	10.26	10.55	11.13	10.28
FBgn0085384	CG34355	3R:23803858-23860013	23.90	27.45	25.57	24.10
FBgn0085446	CG34417	X:6540770-6589173	44.02	52.48	53.23	62.99
FBgn0085452	CG34423	2R:23358611-23359204	16.52	22.40	28.28	59.22
FBgn0086656	shrb	2R:9142145-9143914	63.90	68.96	73.36	81.62
FBgn0086757	cbs	2R:13450585-13453393	10.43	11.60	11.81	11.41
FBgn0086785	Vps36	3L:13511645-13513112	8.29	9.21	9.03	9.48

geneID	GeneSym	Coord	D10_mean	D20_mean	D30_mean	D50_mean
FBgn0086906	sls	3L:2039680-2115611	12.47	14.05	17.93	21.66
FBgn0087035	AGO2	3L:15554112-15561042	55.33	60.49	62.76	67.01
FBgn0250757	CG42235	3R:25907021-25921046	22.33	21.60	21.93	16.57
FBgn0250789	alpha-Spec	3L:1778484-1795328	85.44	93.47	100.86	95.86
FBgn0250819	CG33521	4:1185767-1192985	24.31	26.88	21.60	21.58
FBgn0250851	CG33981	2R:17773963-17781930	3.76	4.29	4.15	4.44
FBgn0250906	Pgk	2L:2746879-2753068	111.37	101.38	104.83	99.65
FBgn0259163	CG42268	3L:9734011-9760395	72.69	79.03	81.82	78.32
FBgn0259209	Mlp60A	2R:24074601-24078223	132.57	170.75	204.75	179.03
FBgn0259214	PMCA	4:328192-358917	149.94	169.42	147.33	149.22
FBgn0259234	Camta	2R:9446045-9480076	23.14	26.55	26.19	28.10
FBgn0259678	sqa	2R:9716347-9748121	13.74	13.64	14.97	15.53
FBgn0259707	CG42361	2R:24607672-24608788	36.50	36.22	40.00	30.50
FBgn0259979	CG17337	2R:5697871-5700212	34.76	40.61	38.46	37.02
FBgn0259991	CG42488	3R:25483097-25485481	10.85	10.31	11.43	10.02
FBgn0260004	Snmp1	3R:21165166-21168205	33.24	36.97	37.20	32.77
FBgn0260439	Pp2A-29B	2L:8366037-8370090	82.17	85.03	86.87	83.10
FBgn0260467	CG7071	3R:22362619-22364580	17.42	16.75	19.19	17.49
FBgn0260639	gammaTub23C	2L:2972890-2974858	13.81	14.02	15.48	13.71
FBgn0260935	ird1	3R:9240756-9245869	16.04	14.77	16.32	13.61
FBgn0260970	CG42593	X:7935895-7953883	18.96	21.57	22.33	21.87
FBgn0260995	CG42596	2R:3066498-3191011	59.64	64.24	58.01	49.68
FBgn0261015	Pif1A	3R:8774285-8801176	140.49	168.15	181.15	191.51
FBgn0261436	DhpD	3R:4227386-4229238	29.79	28.78	29.01	32.52
FBgn0261451	trol	X:2470459-2545410	28.88	27.14	25.04	22.02
FBgn0261532	cdm	3R:18179274-18183479	42.02	39.50	40.76	35.20
FBgn0261565	Lmpt	3L:16865294-16922487	117.77	136.67	134.13	126.06
FBgn0261625	CG42708	2R:12670482-12679593	17.79	18.53	23.00	15.75
FBgn0261674	CG42709	3L:12790191-12796084	11.18	10.78	11.00	9.97
FBgn0261788	Ank2	3L:7655388-7718395	82.75	92.82	102.39	92.40
FBgn0261794	kcc	2R:23907653-23925066	165.55	150.43	142.55	121.81
FBgn0261871	dpr2	2L:10917014-10964153	6.14	5.73	5.93	4.49
FBgn0262169	magu	2R:10052540-10063712	16.25	16.91	16.82	18.65
FBgn0262508	CG43078	3L:8576807-8599617	57.87	67.35	75.89	71.30
FBgn0262579	Ect4	3L:8063873-8108897	120.51	136.24	146.83	165.13
FBgn0262636	Lin29	4:375639-395605	23.10	25.62	26.61	24.86
FBgn0263006	Ca-P60A	2R:23925076-23935884	192.26	162.82	148.80	132.97
FBgn0263396	sqd	3R:13634861-13646304	104.32	130.00	125.84	147.45
FBgn0263593	Lpin	2R:8136985-8156654	76.11	86.58	93.03	87.31
FBgn0263748	CG43673	X:15779145-15780214	37.35	12.36	23.17	6.42
FBgn0263980	CG43729	2R:15401095-15471884	46.95	52.46	53.06	49.48
FBgn0263998	Ack-like	2R:13142833-13152677	14.21	15.99	17.60	17.44
FBgn0264324	spg	3R:28845269-28870439	17.47	20.62	20.02	20.70
FBgn0264489	CG43897	3L:9813846-9834345	123.71	147.98	152.08	156.63
FBgn0264491	how	3R:22042190-22079321	23.68	29.14	27.83	35.83

geneID	GeneSym	Coord	D10_mean	D20_mean	D30_mean	D50_mean
FBgn0264493	rdx	3R:13967060-14032057	34.44	40.55	36.79	40.60
FBgn0264607	CaMKII	4:1035287-1053703	186.94	189.52	155.91	159.58
FBgn0264695	Mhc	2L:16766565-16788766	177.87	186.11	196.53	180.80
FBgn0264894	CG44085	2L:13261007-13284043	28.27	38.07	44.17	60.06
FBgn0265002	CG44153	2L:10064120-10180256	6.32	7.04	7.47	6.92
FBgn0265045	Strn-Mlck	2R:15946932-15989498	55.30	55.26	56.49	51.61
FBgn0265767	zyd	X:23023316-23035550	40.35	42.50	43.28	42.82
FBgn0265998	Doa	3R:28888072-28922772	71.17	81.12	83.02	85.82
FBgn0266346	CngB	2R:21692690-21697152	13.28	12.33	12.81	9.92
FBgn0266446	CG45076	3R:10768000-10777374	54.99	73.08	93.89	116.89
FBgn0266917	CG16941	3R:16999383-17002355	9.40	10.71	11.87	11.47
FBgn0267365	l(2)SH0834	2L:10311049-10312099	31.33	28.60	29.73	26.07
FBgn0267398	Yeti	2R:1343402-1345119	76.07	90.10	79.40	81.82
FBgn0267435	chp	3R:31204179-31210730	342.51	363.48	388.75	359.23
