# Supplementary material for: Increased intron retention is a post‐transcriptional signature associated with progressive aging and Alzheimer’s disease
Source: Aging Cell. 2019 Mar 13;18(3):e12928. doi: 10.1111/acel.12928 (PMC6516162; doi:10.1111/acel.12928)
Supplement: Supplementary file 4 [file ACEL-18-e12928-s004.pdf]

Table S3: David functional annotation chart of differential IR genes identified in different ages of mouse frontal cortex (Lister et al., 2013)

S/N	GOTERMS_BP_DIRECT	Count	%	p-value	Benjamini value	TimePoint
1	GO:0006810~transport	28	15.642	0.003019695	0.920204161	2w_10w
2	GO:0016310~phosphorylation	13	7.263	0.006201975	0.925763397	2w_10w
3	GO:0042692~muscle cell differentiation	3	1.676	0.009012379	0.919768179	2w_10w
4	GO:0015031~protein transport	12	6.704	0.012586296	0.929152844	2w_10w
5	GO:0006897~endocytosis	6	3.352	0.019418467	0.962322997	2w_10w
6	GO:0031122~cytoplasmic microtubule organization	3	1.676	0.037585096	0.995193606	2w_10w
7	GO:0007420~brain development	6	3.352	0.037673399	0.989808716	2w_10w
8	GO:0009058~biosynthetic process	3	1.676	0.039515259	0.98520026	2w_10w
9	GO:0001701~in utero embryonic development	7	3.911	0.043050226	0.983219155	2w_10w
10	GO:0006397~mRNA processing	15	6.494	1.55E-05	0.016140402	10w_22mo
11	GO:0008380~RNA splicing	11	4.762	3.77E-04	0.179356091	10w_22mo
12	GO:0042088~T-helper 1 type immune response	3	1.299	0.00420465	0.770837862	10w_22mo
13	GO:0070932~histone H3 deacetylation	3	1.299	0.008847589	0.902763153	10w_22mo
14	GO:0000226~microtubule cytoskeleton organization	5	2.165	0.022281969	0.991152544	10w_22mo
15	GO:0031297~replication fork processing	3	1.299	0.028904225	0.994070961	10w_22mo
16	GO:0032757~positive regulation of interleukin-8 production	3	1.299	3.12E-02	0.991337435	10w_22mo
17	GO:0042517~positive regulation of tyrosine phosphorylation of Stat3 protein	3	1.299	4.36E-02	0.997118388	10w_22mo
18	GO:1903849~positive regulation of aorta morphogenesis	2	0.866	4.37E-02	0.994549203	10w_22mo
19	GO:0016567~protein ubiquitination	9	3.896	4.94E-02	0.995103624	10w_22mo
20	GO:0016569~covalent chromatin modification	17	3.820	1.66E-04	0.232992643	2w_22mo
21	GO:0006397~mRNA processing	17	3.820	1.33E-03	0.654784067	2w_22mo
22	GO:0050790~regulation of catalytic activity	5	1.124	0.001971144	0.650871119	2w_22mo
23	GO:0030030~cell projection organization	10	2.247	0.004616253	0.842884865	2w_22mo
24	GO:0035556~intracellular signal transduction	17	3.820	0.010340328	0.964068154	2w_22mo
25	GO:0006520~cellular amino acid metabolic process	4	0.899	0.013339346	0.972154869	2w_22mo
26	GO:0006260~DNA replication	8	1.798	0.015032276	0.96863415	2w_22mo
27	GO:0016575~histone deacetylation	4	0.899	0.022304364	0.989016637	2w_22mo
28	GO:0070932~histone H3 deacetylation	3	0.674	0.029340412	0.994978992	2w_22mo
29	GO:0051726~regulation of cell cycle	7	1.573	0.03056182	0.993030219	2w_22mo

S/N	GOTERMS_BP_DIRECT	Count	%	p-value	Benjamini value	TimePoint
30	GO:0006139~nucleobase-containing compound metabolic process	4	0.899	0.031332195	0.990248501	2w_22mo
31	GO:0006974~cellular response to DNA damage stimulus	16	3.596	0.032035291	0.986980234	2w_22mo
32	GO:0008033~tRNA processing	6	1.348	0.033076668	0.984074348	2w_22mo
33	GO:0016574~histone ubiquitination	3	0.674	0.033763779	0.980265057	2w_22mo
34	GO:0006941~striated muscle contraction	3	0.674	0.033763779	0.980265057	2w_22mo
35	GO:0007049~cell cycle	21	4.719	0.034967236	0.977553279	2w_22mo
36	GO:0007420~brain development	10	2.247	0.038952714	0.981186237	2w_22mo
37	GO:0006418~tRNA aminoacylation for protein translation	4	0.899	0.039175746	0.976746404	2w_22mo
38	GO:0006267~pre-replicative complex assembly involved in nuclear cell cycle DNA replicati	2	0.449	0.041481952	0.976854283	2w_22mo
39	GO:0016479~negative regulation of transcription from RNA polymerase I promoter	2	0.449	0.041481952	0.976854283	2w_22mo
40	GO:1904358~positive regulation of telomere maintenance via telomere lengthening	2	0.449	0.041481952	0.976854283	2w_22mo
41	GO:0007155~cell adhesion	17	3.820	0.049200543	0.985715769	2w_22mo

Table S3: Human orthologs of the differential mouse IR genes identified between 10 weeks (young) & 22 months (old) frontal cortex (Lister et al., 2013)

S/N	Mouse Gene ID	MGI ID	Mouse Symbol	Species	Human Gene ID	HGNC ID	Human Symbol	DIOPT Score	Weighted Score	Rank	Best Score
1	18132	107471	Notch4	Human	4855	7884	NOTCH4	14	14.29	high	Yes
2	70511	1917761	Eef2kmt	Human	196483	32221	EEF2KMT	15	15.24	high	Yes
3	751865	3802945	Sap25	Human	100316904	41908	SAP25	11	11.5	high	Yes
4	76265	1923515	Tsen54	Human	283989	27561	TSEN54	15	15.24	high	Yes
5	27390	1351603	Mmel1	Human	79258	14668	MMEL1	11	11.37	high	Yes
6	55981	1891825	Pigb	Human	9488	8959	PIGB	13	13.28	high	Yes
7	243538	2141635	Cfap100	Human	348807	26842	CFAP100	15	15.3	high	Yes
8	18846	107683	Plxna3	Human	55558	9101	PLXNA3	14	14.3	high	Yes
9	269400	2139369	Rtel1	Human	51750	15888	RTEL1	13	13.33	high	Yes
10	52150	1891291	Kcnk6	Human	9424	6281	KCNK6	16	16.25	high	Yes
11	109652	87913	Acy1	Human	95	177	ACY1	13	13.28	high	Yes
12	60534	1926471	Fancg	Human	2189	3588	FANCG	14	14.29	high	Yes
13	353287	2672935	Clec18a	Human	497190	33849	CLEC18B	15	15.3	high	Yes
14	245865	2444120	Spag4	Human	6676	11214	SPAG4	14	14.27	high	Yes
15	433809	2684989	Rnf207	Human	388591	32947	RNF207	10	10.45	high	Yes
16	433466	3845785	Jmjd7	Human	100137047	34397	JMJD7	7	7.66	high	Yes
17	27279	1351484	Tnfrsf12a	Human	51330	18152	TNFRSF12A	13	13.26	high	Yes
18	27404	1351668	Abca8b	Human	10351	38	ABCA8	15	15.22	high	Yes
19	18627	1195265	Per2	Human	8864	8846	PER2	14	14.27	high	Yes
20	70052	1917302	Prpf4	Human	9128	17349	PRPF4	15	15.24	high	Yes
21	100088	1913989	Rcc1	Human	1104	1913	RCC1	15	15.24	high	Yes
22	223646	2442664	Naprt	Human	93100	30450	NAPRT	14	14.29	high	Yes
23	30877	1353651	Gnl3	Human	26354	29931	GNL3	16	16.25	high	Yes
24	14885	1338799	Gtf2h4	Human	2968	4658	GTF2H4	14	14.29	high	Yes
25	67728	1914978	Dph2	Human	1802	3004	DPH2	14	14.29	high	Yes
26	78308	1925558	Gpr108	Human	56927	17829	GPR108	14	14.37	high	Yes
27	234686	2679008	Fhod1	Human	29109	17905	FHOD1	13	13.29	high	Yes
28	229600	2385885	BC028528	Human	79630	26258	C1orf54	14	14.29	high	Yes
29	668940	3710243	Myh7b	Human	57644	15906	MYH7B	13	13.39	high	Yes
30	22379	109569	Fmnl3	Human	91010	23698	FMNL3	14	14.29	high	Yes

S/N	Mouse Gene ID	MGI ID	Mouse Symbol	Species	Human Gene ID	HGNC ID	Human Symbol	DIOPT Score	Weighted Score	Rank	Best Score
31	72151	1919401	Rfc5	Human	5985	9973	RFC5	15	15.3	high	Yes
32	208146	2447762	Yeats2	Human	55689	25489	YEATS2	15	15.3	high	Yes
33	238023	3605542	Hexdc	Human	284004	26307	HEXDC	12	12.32	high	Yes
34	22130	105044	Ttf1	Human	7270	12397	TTF1	12	12.28	high	Yes
35	74322	1921572	Cxxc1	Human	30827	24343	CXXC1	15	15.24	high	Yes
36	69215	1916465	Sat2	Human	112483	23160	SAT2	15	15.35	high	Yes
37	104831	2144837	Ptpn23	Human	25930	14406	PTPN23	15	15.24	high	Yes
38	68241	1915491	Mcrip2	Human	84331	14142	MCRIP2	13	13.27	high	Yes
39	78797	1926047	Ndor1	Human	27158	29838	NDOR1	15	15.24	high	Yes
40	19649	1343102	Robo3	Human	64221	13433	ROBO3	14	14.2	high	Yes
41	223690	2444209	Ankrd54	Human	129138	25185	ANKRD54	13	13.41	high	Yes
42	16882	109152	Lig3	Human	3980	6600	LIG3	14	14.23	high	Yes
43	11512	87917	Adcy6	Human	112	237	ADCY6	16	16.25	high	Yes
44	110094	97577	Phka2	Human	5256	8926	PHKA2	14	14.29	high	Yes
45	52563	1098815	Cdc23	Human	8697	1724	CDC23	15	15.3	high	Yes
46	226432	1918944	Ipo9	Human	55705	19425	IPO9	15	15.24	high	Yes
47	106618	1921267	Wdr90	Human	197335	26960	WDR90	13	13.28	high	Yes
48	12667	1313268	Chrd	Human	8646	1949	CHRD	14	14.21	high	Yes
49	16801	1353510	Arhgef1	Human	9138	681	ARHGEF1	13	13.3	high	Yes
50	100126824	3818630	Sco2	Human	9997	10604	SCO2	14	14.4	high	Yes
51	97884	2145517	B3galnt2	Human	148789	28596	B3GALNT2	15	15.24	high	Yes
52	107650	1334433	Pi4kb	Human	5298	8984	PI4KB	14	14.23	high	Yes
53	69440	1916690	Dennd6b	Human	414918	32690	DENND6B	14	14.29	high	Yes
54	225870	2385695	Rin1	Human	9610	18749	RIN1	16	16.25	high	Yes
55	233405	2446237	Vps33b	Human	26276	12712	VPS33B	15	15.3	high	Yes
56	71957	1919207	Ints11	Human	54973	26052	INTS11	13	13.36	high	Yes
57	11781	1337063	Ap4m1	Human	9179	574	AP4M1	14	14.27	high	Yes
58	17350	101938	Mlh1	Human	4292	7127	MLH1	14	14.29	high	Yes
59	68556	1915806	Uckl1	Human	54963	15938	UCKL1	14	14.37	high	Yes
60	68718	1915968	Rnf166	Human	115992	28856	RNF166	15	15.25	high	Yes
61	230734	2387201	Yrdc	Human	79693	28905	YRDC	15	15.24	high	Yes

S/N	Mouse Gene ID	MGI ID	Mouse Symbol	Species	Human Gene ID	HGNC ID	Human Symbol	DIOPT Score	Weighted Score	Rank	Best Score
62	72699	1919949	Lime1	Human	54923	26016	LIME1	13	13.39	high	Yes
63	231600	2444898	Chfr	Human	55743	20455	CHFR	13	13.26	high	Yes
64	100609	2140844	Nsun5	Human	55695	16385	NSUN5	15	15.24	high	Yes
65	13527	106039	Dtna	Human	1837	3057	DTNA	14	14.14	high	Yes
66	237926	3039628	Rsad1	Human	55316	25634	RSAD1	15	15.24	high	Yes
67	17761	1328328	Map7	Human	9053	6869	MAP7	14	14.29	high	Yes
68	67862	1915112	2310033P09Rik	Human	79169	19032	C1orf35	14	14.29	high	Yes
69	16179	107420	Irak1	Human	3654	6112	IRAK1	14	14.37	high	Yes
70	71947	1919197	Tmem94	Human	9772	28983	TMEM94	14	14.31	high	Yes
71	269951	96414	Idh2	Human	3418	5383	IDH2	15	15.3	high	Yes
72	16068	1333800	Il18bp	Human	10068	5987	IL18BP	14	14.29	high	Yes
73	80880	1098615	Kank3	Human	256949	24796	KANK3	15	15.3	high	Yes
74	73389	894659	Hbp1	Human	26959	23200	HBP1	14	14.29	high	Yes
75	72569	1919819	Bbs5	Human	129880	970	BBS5	15	15.32	high	Yes
76	105446	1917903	Gmpr2	Human	51292	4377	GMPR2	16	16.25	high	Yes
77	16897	102682	Llgl1	Human	3996	6628	LLGL1	15	15.24	high	Yes
78	22122	98857	Tsta3	Human	7264	12390	TSTA3	14	14.28	high	Yes
79	218442	2444223	Serinc5	Human	256987	18825	SERINC5	14	14.29	high	Yes
80	71177	1918427	Ints13	Human	55726	20174	INTS13	14	14.29	high	Yes
81	69080	1916330	Gmppa	Human	29926	22923	GMPPA	15	15.24	high	Yes
82	50782	1354739	Rgs11	Human	8786	9993	RGS11	15	15.3	high	Yes
83	382073	2685960	Ccdc84	Human	338657	30460	CCDC84	14	14.29	high	Yes
84	67392	1914642	4833420G17Rik	Human	375444	24738	C5orf34	14	14.29	high	Yes
85	228410	1351825	Cstf3	Human	1479	2485	CSTF3	14	14.29	high	Yes
86	18432	106181	Mybbp1a	Human	10514	7546	MYBBP1A	14	14.29	high	Yes
87	24051	1346523	Sgcb	Human	6443	10806	SGCB	14	14.29	high	Yes
88	12747	107403	Clk1	Human	1195	2068	CLK1	14	14.37	high	Yes
89	16978	1342770	Lrrfip1	Human	9208	6702	LRRFIP1	13	13.29	high	Yes
90	228961	2448523	Npepl1	Human	79716	16244	NPEPL1	14	14.29	high	Yes
91	17294	96968	Mest	Human	4232	7028	MEST	14	14.29	high	Yes
92	112403	1890444	Dxo	Human	1797	2992	DXO	14	14.29	high	Yes

S/N	Mouse Gene ID	MGI ID	Mouse Symbol	Species	Human Gene ID	HGNC ID	Human Symbol	DIOPT Score	Weighted Score	Rank	Best Score
93	66921	1914171	Prpf38b	Human	55119	25512	PRPF38B	14	14.29	high	Yes
94	72199	1919449	Mms19	Human	64210	13824	MMS19	14	14.21	high	Yes
95	269615	2443078	Plch2	Human	9651	29037	PLCH2	14	14.3	high	Yes
96	66627	1913877	Ogfod2	Human	79676	25823	OGFOD2	14	14.21	high	Yes
97	217588	1918320	Mbip	Human	51562	20427	MBIP	15	15.3	high	Yes
98	69315	1916565	1700001L19Rik	Human	134121	27028	C5orf49	14	14.29	high	Yes
99	74549	1921799	Mau2	Human	23383	29140	MAU2	13	13.33	high	Yes
100	224619	3042141	Traf7	Human	84231	20456	TRAF7	14	14.29	high	Yes
101	67367	1914617	Paxbp1	Human	94104	13579	PAXBP1	14	14.29	high	Yes
102	16173	107936	Il18	Human	3606	5986	IL18	11	11.15	high	Yes
103	71382	1918632	Pex1	Human	5189	8850	PEX1	14	14.29	high	Yes
104	320267	2443699	Fubp3	Human	8939	4005	FUBP3	16	16.25	high	Yes
105	50793	1354944	Orc3	Human	23595	8489	ORC3	14	14.29	high	Yes
106	60507	1931441	Qtrt1	Human	81890	23797	QTRT1	14	14.29	high	Yes
107	21856	1343262	Timm44	Human	10469	17316	TIMM44	15	15.24	high	Yes
108	231003	2678948	Klhl17	Human	339451	24023	KLHL17	13	13.47	high	Yes
109	225392	1918044	Rell2	Human	285613	26902	RELL2	14	14.29	high	Yes
110	94178	1890498	Mcoln1	Human	57192	13356	MCOLN1	14	14.29	high	Yes
111	67420	1914670	Far1	Human	84188	26222	FAR1	15	15.3	high	Yes
112	17537	108519	Meis3	Human	56917	29537	MEIS3	14	14.4	high	Yes
113	74011	1921261	Slc25a27	Human	9481	21065	SLC25A27	15	15.3	high	Yes
114	216148	106180	Shc2	Human	25759	29869	SHC2	13	13.39	high	Yes
115	66264	1913514	Ccdc28b	Human	79140	28163	CCDC28B	13	13.39	high	Yes
116	93843	1347357	Pnck	Human	139728	13415	PNCK	15	15.24	high	Yes
117	67414	1914664	Mfn1	Human	55669	18262	MFN1	14	14.29	high	Yes
118	71514	1918764	Sfpq	Human	6421	10774	SFPQ	14	14.3	high	Yes
119	109229	1924483	Fam118b	Human	79607	26110	FAM118B	14	14.27	high	Yes
120	20383	98285	Srsf3	Human	6428	10785	SRSF3	14	14.4	high	Yes
121	665113	1916264	Tnik	Human	23043	30765	TNIK	14	14.4	high	Yes
122	14430	95638	Galt	Human	2592	4135	GALT	15	15.3	high	Yes
123	13198	109247	Ddit3	Human	1649	2726	DDIT3	14	14.29	high	Yes

S/N	Mouse Gene ID	MGI ID	Mouse Symbol	Species	Human Gene ID	HGNC ID	Human Symbol	DIOPT Score	Weighted Score	Rank	Best Score
124	330671	2652891	B4galnt4	Human	338707	26315	B4GALNT4	15	15.3	high	Yes
125	245877	2384297	Map7d1	Human	55700	25514	MAP7D1	15	15.3	high	Yes
126	83766	1933548	Actl6b	Human	51412	160	ACTL6B	16	16.25	high	Yes
127	15499	96238	Hsf1	Human	3297	5224	HSF1	15	15.3	high	Yes
128	69207	1916457	Srsf11	Human	9295	10782	SRSF11	15	15.3	high	Yes
129	14979	95911	H2-Ke6	Human	7923	3554	HSD17B8	15	15.3	high	Yes
130	17827	103025	Mtx1	Human	4580	7504	MTX1	14	14.29	high	Yes
131	108155	1339639	Ogt	Human	8473	8127	OGT	14	14.21	high	Yes
132	50755	1354699	Fbxo18	Human	84893	13620	FBXO18	15	15.24	high	Yes
133	84505	1934229	Setdb1	Human	9869	10761	SETDB1	15	15.24	high	Yes
134	30840	1354705	Fbxl6	Human	26233	13603	FBXL6	15	15.24	high	Yes
135	227656	2684957	Rexo4	Human	57109	12820	REXO4	16	16.25	high	Yes
136	56389	1928483	Stx5a	Human	6811	11440	STX5	13	13.28	high	Yes
137	66593	1913843	Diablo	Human	56616	21528	DIABLO	13	13.26	high	Yes
138	12531	99701	Cdc25b	Human	994	1726	CDC25B	15	15.24	high	Yes
139	68554	1915804	Cebpzos	Human	100505876	49288	CEBPZOS	13	13.29	high	Yes
140	245857	2683546	Ssh3	Human	54961	30581	SSH3	16	16.25	high	Yes
141	228356	1915079	1110051M20Rik	Human	79096	28720	C11orf49	14	14.29	high	Yes
142	53861	1858211	Zranb2	Human	9406	13058	ZRANB2	12	12.33	high	Yes
143	225027	1926232	Srsf7	Human	6432	10789	SRSF7	15	15.3	high	Yes
144	434437	3646700	Amt	Human	275	473	AMT	15	15.24	high	Yes
145	320816	2444796	Ankrd16	Human	54522	23471	ANKRD16	13	13.39	high	Yes
146	83486	1933204	Rbm5	Human	10181	9902	RBM5	16	16.25	high	Yes
147	21357	103027	Tarbp2	Human	6895	11569	TARBP2	14	14.27	high	Yes
148	11488	1098667	Adam11	Human	4185	189	ADAM11	15	15.24	high	Yes
149	18803	97615	Plcg1	Human	5335	9065	PLCG1	15	15.24	high	Yes
150	76900	1924150	Ssbp4	Human	170463	15676	SSBP4	15	15.3	high	Yes
151	110157	97847	Raf1	Human	5894	9829	RAF1	13	13.26	high	Yes
152	70999	1918249	Naa40	Human	79829	25845	NAA40	15	15.24	high	Yes
153	70430	1917680	Tbce	Human	6905	11582	TBCE	15	15.24	high	Yes
154	68183	1915433	Bcas2	Human	10286	975	BCAS2	14	14.29	high	Yes

S/N	Mouse Gene ID	MGI ID	Mouse Symbol	Species	Human Gene ID	HGNC ID	Human Symbol	DIOPT Score	Weighted Score	Rank	Best Score
155	225861	2444704	Snx32	Human	254122	26423	SNX32	15	15.3	high	Yes
156	24068	1344414	Sra1	Human	10011	11281	SRA1	12	12.35	high	Yes
157	68278	1915528	Ddx39	Human	10212	17821	DDX39A	13	13.29	high	Yes
158	66625	1913875	Pnisr	Human	25957	21222	PNISR	14	14.29	high	Yes
159	230101	2654325	Gba2	Human	57704	18986	GBA2	15	15.24	high	Yes
160	225160	1919668	Thoc1	Human	9984	19070	THOC1	14	14.29	high	Yes
161	234407	1924348	Colgalt1	Human	79709	26182	COLGALT1	15	15.3	high	Yes
162	83768	1933213	Dpp7	Human	29952	14892	DPP7	15	15.24	high	Yes
163	14073	109609	Faah	Human	2166	3553	FAAH	14	14.34	high	Yes
164	654795	1916876	Sdr39u1	Human	56948	20275	SDR39U1	14	14.29	high	Yes
165	19082	108411	Prkag1	Human	5571	9385	PRKAG1	15	15.2	high	Yes
166	51885	1196293	Tubgcp4	Human	27229	16691	TUBGCP4	15	15.3	high	Yes
167	216190	2384914	Appl2	Human	55198	18242	APPL2	13	13.26	high	Yes
168	319278	2441788	A230050P20Rik	Human	55337	25649	C19orf66	14	14.29	high	Yes
169	108062	1343054	Cstf2	Human	1478	2484	CSTF2	15	15.3	high	Yes
170	67399	1914649	Pdlim7	Human	9260	22958	PDLIM7	15	15.24	high	Yes
171	23821	1346542	Bace1	Human	23621	933	BACE1	15	15.24	high	Yes
172	54160	1858683	Copg2	Human	26958	2237	COPG2	11	11.53	high	Yes
173	66826	109626	Taz	Human	6901	11577	TAZ	14	14.34	high	Yes
174	78670	1925920	Plekhj1	Human	55111	18211	PLEKHJ1	13	13.36	high	Yes
175	15369	109373	Hmox2	Human	3163	5014	HMOX2	15	15.24	high	Yes
176	22221	104889	Ubp1	Human	7342	12507	UBP1	15	15.3	high	Yes
177	64085	1929897	Clstn2	Human	64084	17448	CLSTN2	16	16.25	high	Yes
178	68597	1915847	Ccdc167	Human	154467	21239	CCDC167	13	13.39	high	Yes
179	227632	1924627	Kcnt1	Human	57582	18865	KCNT1	16	16.25	high	Yes
180	214951	2384891	Rhbdl1	Human	9028	10007	RHBDL1	15	15.3	high	Yes
181	381199	2147713	Tmem151a	Human	256472	28497	TMEM151A	14	14.29	high	Yes
182	209011	2385849	Sirt7	Human	51547	14935	SIRT7	14	14.21	high	Yes
183	107999	1306825	Gtpbp6	Human	8225	30189	GTPBP6	13	13.33	high	Yes
184	227700	2385131	Sh3glb2	Human	56904	10834	SH3GLB2	13	13.19	high	Yes
185	207777	2450877	Tspoap1	Human	9256	16831	TSPOAP1	15	15.3	high	Yes

S/N	Mouse Gene ID	MGI ID	Mouse Symbol	Species	Human Gene ID	HGNC ID	Human Symbol	DIOPT Score	Weighted Score	Rank	Best Score
186	214917	2384888	Fam173a	Human	65990	14152	FAM173A	15	15.3	high	Yes
187	78784	1926034	Celf3	Human	11189	11967	CELF3	14	14.29	high	Yes
188	15388	104816	Hnrnpl	Human	3191	5045	HNRNPL	14	14.29	high	Yes
189	224019	107238	Tmem191c	Human	645426	33601	TMEM191C	6	6.11	high	Yes
190	83965	1933830	Enpp5	Human	59084	13717	ENPP5	13	13.33	high	Yes
191	104444	1888981	Rexo2	Human	25996	17851	REXO2	15	15.24	high	Yes
192	14828	95835	Hspa5	Human	3309	5238	HSPA5	16	16.25	high	Yes
193	20266	98247	Scn1b	Human	6324	10586	SCN1B	15	15.24	high	Yes
194	15461	96224	Hras	Human	3265	5173	HRAS	14	14.45	high	Yes
195	80904	2135752	Dtx3	Human	196403	24457	DTX3	15	15.3	high	Yes
196	11836	88065	Araf	Human	369	646	ARAF	15	15.3	high	Yes
197	67789	1915039	Dalrd3	Human	55152	25536	DALRD3	15	15.24	high	Yes
198	107831	1933736	Adgrb1	Human	575	943	ADGRB1	15	15.3	high	Yes
199	268482	96687	Krt12	Human	3859	6414	KRT12	15	15.3	high	Yes
200	67881	1915131	Mdp1	Human	145553	28781	MDP1	14	14.29	high	Yes
201	68165	1915415	Fdx1l	Human	112812	30546	FDX1L	15	15.3	high	Yes
202	225341	2385067	Lims2	Human	55679	16084	LIMS2	16	16.25	high	Yes
203	68475	1915725	Ssna1	Human	8636	11321	SSNA1	14	14.29	high	Yes
204	14814	95823	Grin2d	Human	2906	4588	GRIN2D	15	15.24	high	Yes
205	68379	1920234	Ciz1	Human	25792	16744	CIZ1	15	15.24	high	Yes
206	76485	1923735	Glt8d1	Human	55830	24870	GLT8D1	15	15.3	high	Yes
207	192169	1913679	Ufsp2	Human	55325	25640	UFSP2	12	12.1	high	Yes
208	68347	1915597	Mettl26	Human	84326	14141	METTL26	13	13.39	high	Yes
209	109331	1925927	Rnf20	Human	56254	10062	RNF20	14	14.29	high	Yes
210	268933	2446285	Wdr24	Human	84219	20852	WDR24	13	13.29	high	Yes
211	20535	109351	Slc4a2	Human	6522	11028	SLC4A2	16	16.25	high	Yes
212	108077	1099835	Skiv2l	Human	6499	10898	SKIV2L	14	14.27	high	Yes
213	224650	2446180	Anks1	Human	23294	20961	ANKS1A	14	14.29	high	Yes
214	84585	2148796	Rnf123	Human	63891	21148	RNF123	15	15.24	high	Yes
215	18438	1338859	P2rx4	Human	5025	8535	P2RX4	16	16.25	high	Yes
216	243914	2180197	Lgi4	Human	163175	18712	LGI4	15	15.3	high	Yes

S/N	Mouse Gene ID	MGI ID	Mouse Symbol	Species	Human Gene ID	HGNC ID	Human Symbol	DIOPT Score	Weighted Score	Rank	Best Score
217	20401	104603	Sh3bp1	Human	23616	10824	SH3BP1	12	12.35	high	Yes
218	27403	1351646	Abca7	Human	10347	37	ABCA7	15	15.3	high	Yes
219	59032	1930009	Ppp2r3c	Human	55012	17485	PPP2R3C	13	13.16	high	Yes
220	140571	2154240	Plxnb3	Human	5365	9105	PLXNB3	13	13.39	high	Yes
221	16450	1098270	Jag2	Human	3714	6189	JAG2	14	14.29	high	Yes
222	75410	109565	Kmt2b	Human	9757	15840	KMT2B	16	16.25	high	Yes
223	15208	104876	Hes5	Human	388585	19764	HES5	15	15.24	high	Yes
224	74126	1921376	Syvn1	Human	84447	20738	SYVN1	12	12.18	high	Yes
225	11845	99435	Arf6	Human	382	659	ARF6	16	16.25	high	Yes
226	234839	3603204	Piezo1	Human	9780	28993	PIEZO1	12	12.46	high	Yes
227	17534	107818	Mrc2	Human	9902	16875	MRC2	13	13.39	high	Yes
228	106512	2146785	Gpsm3	Human	63940	13945	GPSM3	14	14.29	high	Yes
229	50498	1354171	Ebi3	Human	10148	3129	EBI3	15	15.24	high	Yes
230	13846	104757	Ephb4	Human	2050	3395	EPHB4	15	15.3	high	Yes
231	226250	2147658	Afap1l2	Human	84632	25901	AFAP1L2	14	14.21	high	Yes

Table S3: Differential retained introns between 2 weeks & 10 weeks mouse frontal cortex (Lister et al, 2013)

S/N	Chr	Start	End	Gene	Ensembl ID	IR ratio 2W	IR ratio 10W	*p* -value
1	1	135405972	135406516	Ipo9	ENSMUSG00000041879	0.103676	0.0455369	0.0138955
2	1	36701901	36702058	Actr1b	ENSMUSG00000037351	0.109256	0.0599466	0.0018512
3	1	74563623	74564376	Plcd4	ENSMUSG00000026173	0.140487	0.0101506	0.028191
4	1	74565367	74565746	Plcd4	ENSMUSG00000026173	0.190556	0.0440167	0.0444591
5	1	75170459	75170538	Zfand2b	ENSMUSG00000026197	0.138799	0.0284613	0.00582513
6	1	75198865	75199063	Ankzf1	ENSMUSG00000026199	0.324683	0.147112	0.0360421
7	1	75384745	75384827	Speg	ENSMUSG00000026207	0.14324	0.494066	0.000209856
8	1	75441827	75441984	Gmppa	ENSMUSG00000033021	0.0867148	0.195162	0.0218421
9	10	127185172	127185286	Arhgef25	ENSMUSG00000019467	0.138129	0.0645002	0.00741354
10	10	128124262	128124334	Baz2a	ENSMUSG00000040054	0.151713	0.0150502	0.0328574
11	10	128315657	128315775	Pan2	ENSMUSG00000005682	0.0251781	0.218274	0.0272492
12	10	80004850	80004953	Abca7	ENSMUSG00000035722	0.170213	0.016147	0.0492963
13	10	80393018	80393094	Mbd3	ENSMUSG00000035478	0.181425	0.0952059	0.000973992
14	10	80703756	80703852	Izumo4	ENSMUSG00000055862	0.266731	0.130612	0.0217406
15	10	81262156	81262447	Matk	ENSMUSG00000004933	0.100135	0.0555539	0.0220574
16	10	81580667	81580894	Tle2	ENSMUSG00000034771	0.214447	0.0410822	0.0389531
17	11	100348859	100349236	Hap1	ENSMUSG00000006930	0.600634	0.776508	0.0348457
18	11	101183582	101183851	Cntnap1	ENSMUSG00000017167	0.11418	0.0316603	0.000187818
19	11	115255426	115255685	Grin2c	ENSMUSG00000020734	0.146571	0.0320412	0.0300785
20	11	115788390	115788662	Tmem94	ENSMUSG00000020747	0.170254	0.0444956	0.0352388
21	11	115893329	115893494	Recql5	ENSMUSG00000020752	0.211514	0.0231092	0.0213726
22	11	120008444	120009274	Aatk	ENSMUSG00000025375	0.126907	0.241595	0.00039582
23	11	120612083	120612462	Pcyt2	ENSMUSG00000025137	0.237042	0.104075	0.000564553
24	11	120788596	120788669	Gps1	ENSMUSG00000025156	0.174881	0.114598	0.00258482
25	11	121221161	121221235	Hexdc	ENSMUSG00000039307	0.176117	0.0595745	0.0274639
26	11	121221337	121221500	Hexdc	ENSMUSG00000039307	0.183711	0.304175	0.0371619
27	11	5707409	5707486	Mrps24	ENSMUSG00000020477	0.25406	0.162847	0.0383016
28	11	59209051	59209127	2310033P09Rik	ENSMUSG00000020441	0.135135	0.0224981	0.0339436
29	11	61450902	61450977	Rnf112	ENSMUSG00000010086	0.392641	0.318414	0.0114913
30	11	62318030	62319365	Ncor1	ENSMUSG00000018501	0.0695276	0.123352	0.0437627
31	11	69330226	69330396	Kcnab3	ENSMUSG00000018470	0.13455	0.0224996	0.00167641
32	11	70010770	70010848	Acadvl	ENSMUSG00000018574	0.0294238	0.103818	0.0274464
33	11	75647276	75647401	Inpp5k	ENSMUSG00000006127	0.0521253	0.162584	0.0307642
34	11	78161448	78161517	Traf4	ENSMUSG00000017386	0.0188679	0.125	0.0412001
35	11	78470851	78471370	Slc46a1	ENSMUSG00000020829	0.621037	0.315145	0.0121228
36	11	87589818	87589955	Sep-04	ENSMUSG00000020486	0.136907	0.0891177	0.0389162
37	11	87777235	87777766	Bzrap1	ENSMUSG00000034156	0.389259	0.256118	0.00817042
38	11	98716525	98717708	Med24	ENSMUSG00000017210	0.139687	0.0671352	0.0280788
39	11	99418135	99418273	Krt12	ENSMUSG00000020912	0.686452	0.516022	0.0437716
40	12	109034351	109035327	Begain	ENSMUSG00000040867	0.118713	0.0658253	0.00989419
41	12	112912720	112913164	Jag2	ENSMUSG00000002799	0.122763	0.0305179	0.037627
42	12	31327821	31329109	Lamb1	ENSMUSG00000002900	0.14778	0.461928	0.0110325
43	12	56340297	56340384	Mbip	ENSMUSG00000021028	0.152344	0.433694	0.00633853
44	12	69372223	69372323	Arf6	ENSMUSG00000044147	0.0909091	0.310034	0.0272719
45	12	80169016	80170182	Actn1	ENSMUSG00000015143	0.120554	0.0410996	0.00320106
46	13	19370367	19370479	Stard3nl	ENSMUSG00000003062	0.0693026	0.143885	0.00773779
47	13	21448484	21449273	Zscan26	ENSMUSG00000022228	0.384601	0.224713	0.0436382

S/N	Chr	Start	End	Gene	Ensembl ID	IR ratio 2W	IR ratio 10W	*p* -value
48	13	49203789	49203928	Card19	ENSMUSG00000037960	0.430788	0.164756	0.0428843
49	13	55507123	55507329	Pdlim7	ENSMUSG00000021493	0.387267	0.244018	0.0129957
50	13	55507366	55507487	Pdlim7	ENSMUSG00000021493	0.349804	0.228908	0.0252138
51	13	55531684	55531831	Ddx41	ENSMUSG00000021494	0.10292	0.0344828	0.0218174
52	13	55535804	55535885	Ddx41	ENSMUSG00000021494	0.145781	0.0306122	0.00527032
53	13	92844867	92845205	Mtx3	ENSMUSG00000021704	0.225238	0.0482529	0.0306794
54	15	102500922	102501100	Map3k12	ENSMUSG00000023050	0.174924	0.10103	0.0191782
55	15	74582987	74584377	Adgrb1	ENSMUSG00000034730	0.0652778	0.157383	1.2739E-07
56	15	75925662	75925792	Tsta3	ENSMUSG00000022570	0.17639	0.0811304	0.0319573
57	15	76537006	76537089	Fbxl6	ENSMUSG00000022559	0.147727	0.0416667	0.018448
58	15	76537197	76537288	Fbxl6	ENSMUSG00000022559	0.0377179	0.117007	0.0385786
59	15	76593859	76593937	Adck5	ENSMUSG00000022550	0.626667	0.26087	0.0358645
60	15	89373602	89373682	Sco2	ENSMUSG00000091780	0.112768	0.03125	0.0397963
61	15	89584007	89584278	Rabl2	ENSMUSG00000022621	0.395215	0.215098	0.0132688
62	15	98595042	98595126	Adcy6	ENSMUSG00000022994	0.146552	0.0433213	0.0310691
63	16	17276887	17276961	Tmem191c	ENSMUSG00000055692	0.224604	0.147541	0.0134029
64	16	20709315	20709569	Clcn2	ENSMUSG00000022843	0.173834	0.0382553	0.000669554
65	16	20734401	20734486	Chrd	ENSMUSG00000006958	0.209534	0.0540541	0.0433971
66	16	20734586	20734685	Chrd	ENSMUSG00000006958	0.0239264	0.138603	0.0304049
67	16	20736143	20736263	Chrd	ENSMUSG00000006958	0.190869	0.0694334	0.0471994
68	16	32616898	32617096	Tfrc	ENSMUSG00000022797	0.12	0.0465277	0.0185625
69	16	5011692	5012240	Rogdi	ENSMUSG00000022540	0.142681	0.0724463	0.00361119
70	16	5198623	5198730	Nagpa	ENSMUSG00000023143	0.144737	0.00939542	0.0341145
71	17	15521793	15521915	Pdcd2	ENSMUSG00000014771	0.182658	0.0285714	0.00495297
72	17	24529970	24530054	Rab26	ENSMUSG00000079657	0.111804	0.0381813	0.00814461
73	17	24890460	24890567	Spsb3	ENSMUSG00000024160	0.190426	0.0977918	0.00711228
74	17	24890935	24891010	Spsb3	ENSMUSG00000024160	0.149454	0.0841864	0.018174
75	17	25235111	25235181	Gnptg	ENSMUSG00000035521	0.166635	0.105758	0.0360887
76	17	25827532	25827652	Wdr24	ENSMUSG00000025737	0.172083	0.0625512	0.026275
77	17	25835893	25835968	Rhbdl1	ENSMUSG00000025735	0.0900859	0.194456	0.000745908
78	17	25840751	25840835	Rhot2	ENSMUSG00000025733	0.138024	0.054657	0.00158414
79	17	25868543	25868624	Fam195a	ENSMUSG00000025732	0.488665	0.0975104	0.0185286
80	17	25876562	25876660	Mettl26	ENSMUSG00000025731	0.280374	0.164381	0.0310218
81	17	26203512	26203577	Rgs11	ENSMUSG00000024186	0.677785	0.361111	0.0416643
82	17	26934535	26934630	Phf1	ENSMUSG00000024193	0.143955	0.043863	0.0289048
83	17	32316667	32317066	Akap8	ENSMUSG00000024045	0.236354	0.655198	0.010978
84	17	37011517	37012062	Mog	ENSMUSG00000076439	0.133681	0.0160183	0.00342837
85	17	44082920	44085204	Enpp5	ENSMUSG00000023960	0.638339	0.421266	7.93525E-05
86	17	56768628	56768805	Dus3l	ENSMUSG00000007603	0.528766	0.400237	0.043818
87	17	80204387	80205261	Srsf7	ENSMUSG00000024097	0.229582	0.14186	0.0225319
88	18	32429303	32429699	Bin1	ENSMUSG00000024381	0.101317	0.146861	0.0440452
89	18	34651462	34651546	Cdc23	ENSMUSG00000024370	0.167508	0.0515222	0.0339197
90	18	36787627	36787898	Hars2	ENSMUSG00000019143	0.401368	0.177975	0.0383683
91	18	37958389	37958511	Rell2	ENSMUSG00000044024	0.114653	0.0561689	0.00316136
92	18	37958571	37958758	Rell2	ENSMUSG00000044024	0.116543	0.0754282	0.030889
93	18	38258186	38258269	0610009O20Rik	ENSMUSG00000024442	0.127989	0.00403101	0.00911186
94	18	61738756	61739123	Afap1l1	ENSMUSG00000033032	0.114856	0.0089173	0.0139939
95	18	74219238	74219382	Cxxc1	ENSMUSG00000024560	0.1	0.213039	0.0196597

S/N	Chr	Start	End	Gene	Ensembl ID	IR ratio 2W	IR ratio 10W	*p* -value
96	19	29584213	29584466	Ric1	ENSMUSG00000038658	0.136364	0.0149181	0.0480064
97	19	36970038	36972897	Btaf1	ENSMUSG00000040565	0.00298221	0.447136	0.000171702
98	19	43665828	43666914	Slc25a28	ENSMUSG00000040414	0.459284	0.247403	0.00985645
99	19	5051254	5051353	Rin1	ENSMUSG00000024883	0.230769	0.00924176	0.0021926
100	19	5495982	5496057	Snx32	ENSMUSG00000056185	0.10255	0.0342904	0.00418044
101	19	5498301	5498395	Snx32	ENSMUSG00000056185	0.17134	0.106953	0.0476063
102	19	8743135	8743352	Stx5a	ENSMUSG00000010110	0.102144	0.0420223	0.0264319
103	19	8871756	8871858	Ubxn1	ENSMUSG00000071655	0.149864	0.0883154	0.0359977
104	2	112640286	112640612	Ryr3	ENSMUSG00000057378	0.0322581	0.290203	0.0292136
105	2	119752156	119752230	Ltk	ENSMUSG00000027297	0.514472	0.236816	0.00683572
106	2	121196242	121196734	Tubgcp4	ENSMUSG00000027263	0.507753	0.244023	0.020808
107	2	164834208	164834319	Ctsa	ENSMUSG00000017760	0.0430152	0.108713	0.00784084
108	2	164893615	164893700	Zfp335	ENSMUSG00000039834	0.241489	0.395754	0.031448
109	2	181339094	181339162	Rtel1	ENSMUSG00000038685	0.346939	0.0333333	0.0254804
110	2	181382544	181382615	Lime1	ENSMUSG00000090077	0.16574	0.0487805	0.0126838
111	2	20808024	20808709	Etl4	ENSMUSG00000036617	0.126541	0.279269	0.00308322
112	2	25353516	25353607	Dpp7	ENSMUSG00000026958	0.365836	0.197706	0.042136
113	2	25354822	25354892	Dpp7	ENSMUSG00000026958	0.341865	0.0294957	0.0272401
114	2	25909306	25909593	Kcnt1	ENSMUSG00000058740	0.192787	0.0980598	0.0200227
115	2	26591728	26592117	Egfl7	ENSMUSG00000026921	0.192698	0.0985656	0.0267453
116	2	30347104	30348554	Sh3glb2	ENSMUSG00000026860	0.144545	0.101399	0.0173885
117	2	30348617	30349225	Sh3glb2	ENSMUSG00000026860	0.150472	0.0912719	0.00542029
118	2	32377362	32377508	Ciz1	ENSMUSG00000039205	0.145863	0.312266	0.00604352
119	2	32377630	32377963	Ciz1	ENSMUSG00000039205	0.131824	0.374056	8.73801E-05
120	2	5866241	5866353	Nudt5	ENSMUSG00000025817	0.139	0.0111089	0.0181256
121	2	69654112	69654245	Bbs5	ENSMUSG00000063145	0.179678	0.0390625	0.0350721
122	2	69654373	69655498	Bbs5	ENSMUSG00000063145	0.207745	0.0575585	0.016264
123	3	10346026	10346109	Zfand1	ENSMUSG00000039795	0.112296	0.0204717	0.0161365
124	3	108075359	108075530	Ampd2	ENSMUSG00000027889	0.137764	0.0541077	0.0205616
125	3	108079326	108079586	Ampd2	ENSMUSG00000027889	0.274611	0.123459	0.0196358
126	3	116128808	116129303	Vcam1	ENSMUSG00000027962	0.11468	0.00443232	0.0116561
127	3	35837589	35839048	Atp11b	ENSMUSG00000037400	0.701022	0.418338	0.0101762
128	3	80688714	80689102	Gria2	ENSMUSG00000033981	0.254224	0.188426	0.00244411
129	3	89210183	89210265	Mtx1	ENSMUSG00000064068	0.172066	0.303328	0.01356
130	3	89343583	89343672	Adam15	ENSMUSG00000028041	0.309963	0.187342	0.0279879
131	3	94955385	94955745	Rfx5	ENSMUSG00000005774	0.304369	0.0891365	0.033203
132	3	96707300	96707557	Nudt17	ENSMUSG00000028100	0.219243	0.0451915	0.0431203
133	4	109062332	109062464	Osbpl9	ENSMUSG00000028559	0.36932	0.241812	0.0176989
134	4	117130817	117131061	Plk3	ENSMUSG00000028680	0.0444444	0.180042	0.0498074
135	4	141465640	141465721	Zbtb17	ENSMUSG00000006215	0.160434	0.0380835	0.0207579
136	4	154961049	154961135	Hes5	ENSMUSG00000048001	0.119928	0.298246	0.0129777
137	4	155854659	155854730	Dvl1	ENSMUSG00000029071	0.256009	0.148439	0.00572899
138	4	155855025	155855103	Dvl1	ENSMUSG00000029071	0.10324	0.039604	0.00777573
139	4	43569285	43569488	Gba2	ENSMUSG00000028467	0.109859	0.0499484	0.0276359
140	5	108653219	108653635	Dgkq	ENSMUSG00000004815	0.208755	0.0816799	0.0454035
141	5	110104168	110104267	Gtpbp6	ENSMUSG00000033434	0.0832274	0.332787	1.35676E-05
142	5	110104658	110104889	Gtpbp6	ENSMUSG00000033434	0.0960578	0.189857	0.00372204
143	5	110105098	110105198	Gtpbp6	ENSMUSG00000033434	0.101284	0.272589	0.000104511

S/N	Chr	Start	End	Gene	Ensembl ID	IR ratio 2W	IR ratio 10W	*p* -value
144	5	118056438	118056536	Tesc	ENSMUSG00000029359	0.349206	0.22902	0.0128326
145	5	124000774	124000991	Hip1r	ENSMUSG00000000915	0.120585	0.0331328	0.00685577
146	5	137520800	137520884	Gigyf1	ENSMUSG00000029714	0.413203	0.0666667	0.0282369
147	5	24441261	24441335	Fastk	ENSMUSG00000028959	0.121038	0.0492099	0.0157037
148	5	24593118	24593224	Smarcd3	ENSMUSG00000028949	0.0715394	0.116272	0.0272798
149	5	30930636	30930715	Khk	ENSMUSG00000029162	0.25	0.0479798	0.0295263
150	5	74597410	74597775	Lnx1	ENSMUSG00000029228	0.359438	0.517787	0.0152329
151	6	106779041	106779185	Trnt1	ENSMUSG00000013736	0.290684	0.469536	0.00534542
152	6	113194303	113195214	Lhfpl4	ENSMUSG00000042873	0.0431655	0.16206	0.0251824
153	6	124702322	124702411	Lpcat3	ENSMUSG00000004270	0.00041485	0.110512	0.00587187
154	6	30742220	30742527	Mest	ENSMUSG00000051855	0.0417715	0.115133	0.0282217
155	6	30742605	30742715	Mest	ENSMUSG00000051855	0.0437436	0.149425	0.00798307
156	6	30742852	30743013	Mest	ENSMUSG00000051855	0.0149825	0.118349	0.00191984
157	6	30744932	30745081	Mest	ENSMUSG00000051855	0.056582	0.151064	0.0096256
158	6	30746325	30747051	Mest	ENSMUSG00000051855	0.0725477	0.22368	0.0060604
159	6	30748987	30749405	Copg2	ENSMUSG00000025607	0.192642	0.0832169	0.00215607
160	6	30749899	30750537	Copg2	ENSMUSG00000025607	0.299038	0.145063	0.0027501
161	6	85512943	85513264	Egr4	ENSMUSG00000071341	0.0353649	0.117543	0.0371271
162	7	105740867	105740959	Ilk	ENSMUSG00000030890	0.0608129	0.119696	0.0136622
163	7	126444429	126444522	Rabep2	ENSMUSG00000030727	0.111675	0.0119048	0.0413648
164	7	141064555	141064640	B4galnt4	ENSMUSG00000055629	0.138224	0.0666295	0.0456837
165	7	141491673	141491773	Tspan4	ENSMUSG00000025511	0.018641	0.103339	0.0155036
166	7	27325803	27326486	Ltbp4	ENSMUSG00000040488	0.10277	0.0127281	0.0344198
167	7	34118999	34119138	Wtip	ENSMUSG00000036459	0.415011	0.0952381	0.0263377
168	7	44842720	44842815	Tbc1d17	ENSMUSG00000038520	0.106628	0.0109389	0.00578796
169	7	45634854	45634966	Rasip1	ENSMUSG00000044562	0.34715	0.692205	0.0368241
170	7	45636581	45636719	Rasip1	ENSMUSG00000044562	0.472081	0.783269	0.0111116
171	7	45834481	45834638	Grin2d	ENSMUSG00000002771	0.425973	0.795902	0.0020361
172	7	80891909	80892975	Wdr73	ENSMUSG00000025722	0.319578	0.182444	0.0387605
173	7	90444377	90445424	Crebzf	ENSMUSG00000051451	0.229963	0.387672	0.0293143
174	8	105356751	105356970	Slc9a5	ENSMUSG00000014786	0.482721	0.233753	0.0144635
175	8	105697945	105698030	Carmil2	ENSMUSG00000050357	0.159249	0.0779487	0.0251364
176	8	105704021	105704112	Enkd1	ENSMUSG00000013155	0.00211193	0.16	0.0125587
177	8	105704249	105704350	Enkd1	ENSMUSG00000013155	0.0130781	0.25	0.0046312
178	8	105937381	105937493	Psmb10	ENSMUSG00000031897	0.140173	0.0648956	0.0199868
179	8	122482526	122482648	Piezo1	ENSMUSG00000014444	0.0198366	0.1693	0.0474209
180	8	123227017	123227422	Cdk10	ENSMUSG00000033862	0.176755	0.0725742	0.00618131
181	8	123230327	123230593	Cdk10	ENSMUSG00000033862	0.185179	0.0969331	0.0227315
182	8	25735111	25735744	Ddhd2	ENSMUSG00000061313	0.14301	0.0591961	0.0445232
183	8	25741354	25741600	Ddhd2	ENSMUSG00000061313	0.110768	0.0259512	0.0473574
184	8	70186776	70188299	Slc25a42	ENSMUSG00000002346	0.171454	0.0428758	0.0308023
185	8	70188949	70189410	Slc25a42	ENSMUSG00000002346	0.268024	0.121274	0.0449073
186	8	70598617	70598701	Ssbp4	ENSMUSG00000070003	0.43918	0.288993	0.00036309
187	8	83725346	83725864	Adgre5	ENSMUSG00000002885	0.0102123	0.117177	0.0396201
188	8	84098040	84098198	Dcaf15	ENSMUSG00000037103	0.105977	0.0212766	0.0475055
189	8	84134987	84135061	Cc2d1a	ENSMUSG00000036686	0.00952381	0.108407	0.00167772
190	8	84835674	84835754	Rad23a	ENSMUSG00000003813	0.109234	0.19978	0.0352085
191	8	85064496	85064580	Fbxw9	ENSMUSG00000008167	0.20373	0.490196	0.00644022

S/N	Chr	Start	End	Gene	Ensembl ID	IR ratio 2W	IR ratio 10W	*p* -value
192	8	85066169	85066272	Fbxw9	ENSMUSG00000008167	0.0364794	0.133333	0.0151772
193	8	85079837	85080119	Wdr83	ENSMUSG00000005150	0.55836	0.35979	0.0251254
194	8	85492689	85492904	Gpt2	ENSMUSG00000031700	0.0808982	0.250323	0.0268353
195	8	91106983	91107057	Rbl2	ENSMUSG00000031666	0.131403	0.0397727	0.0457597
196	9	106435014	106435095	Acy1	ENSMUSG00000023262	0.25	0.0155297	0.0142472
197	9	107590422	107590504	Ifrd2	ENSMUSG00000010048	0.586614	0.10683	0.00655897
198	9	108299890	108300533	Amt	ENSMUSG00000032607	0.0307377	0.171361	0.0356239
199	9	21073318	21073388	Fdx1l	ENSMUSG00000079677	0.52381	0.309353	0.00833777
200	9	21590028	21590110	Yipf2	ENSMUSG00000032182	0.242991	0.0611961	0.0137388
201	9	44298411	44298872	Hinfp	ENSMUSG00000032119	0.181818	0.0176355	0.0446779
202	9	45859059	45859278	Bace1	ENSMUSG00000032086	0.164452	0.253967	0.0244596
203	9	59713940	59714176	Gramd2	ENSMUSG00000074259	0.0237581	0.372837	0.0107422
204	9	64307348	64307429	Dis3l	ENSMUSG00000032396	0.0208333	0.13151	0.0276094
205	9	97445713	97454582	Clstn2	ENSMUSG00000032452	0.57461	0.409153	0.0345341
206	X	134080437	134081190	Cstf2	ENSMUSG00000031256	0.364152	0.240265	0.0444715
207	X	160529015	160529914	Phka2	ENSMUSG00000031295	0.268109	0.0721455	0.0259692
208	X	73657343	73657437	Pnck	ENSMUSG00000002012	0.148925	0.042708	0.000162521
209	X	73657513	73657719	Pnck	ENSMUSG00000002012	0.126315	0.0473795	0.00557259

Table S3: Differential retained introns between 10 weeks & 22 months mouse frontal cortex (Lister et al, 2013)

S/N	Chr	Start	End	Gene	Ensembl ID	IR ratio 10w	IR ratio 22m	*p* -value
1	1	135405972	135406516	Ipo9	ENSMUSG00000041879	0.0354338	0.102966	1.32044E-05
2	1	58414684	58417012	Clk1	ENSMUSG00000026034	0.0569629	0.122465	0.012689
3	1	75442031	75442184	Gmppa	ENSMUSG00000033021	0.100045	0.218837	0.00314071
4	1	91076991	91078390	Lrrfip1	ENSMUSG00000026305	0.0498244	0.106709	0.0462956
5	1	91424486	91427738	Per2	ENSMUSG00000055866	0.0242656	0.121902	0.018144
6	10	127191039	127191335	Dtx3	ENSMUSG00000040415	0.189415	0.161327	0.0486148
7	10	127295478	127295722	Ddit3	ENSMUSG00000025408	0.0971555	0.174634	0.00819299
8	10	20261983	20266735	Map7	ENSMUSG00000019996	0.0408969	0.10135	0.0148992
9	10	79621187	79622254	Shc2	ENSMUSG00000020312	0.0942331	0.175018	0.0385415
10	10	80011842	80011914	Abca7	ENSMUSG00000035722	0.144304	0.058689	0.0368013
11	10	80797839	80797909	Plekhj1	ENSMUSG00000035278	0.258642	0.358105	0.0448766
12	10	83611492	83612711	Appl2	ENSMUSG00000020263	0.155212	0.230299	0.0246411
13	11	102777648	102778130	Adam11	ENSMUSG00000020926	0.0749961	0.117354	0.0387834
14	11	105347473	105347598	Mrc2	ENSMUSG00000020695	0.157895	0.017321	0.0452675
15	11	109945858	109946244	Abca8b	ENSMUSG00000020620	0.0320013	0.177181	0.015338
16	11	115788390	115788662	Tmem94	ENSMUSG00000020747	0.0436384	0.105741	0.0367392
17	11	115820223	115820378	Tsen54	ENSMUSG00000020781	0.0070687	0.156727	0.020483
18	11	120620504	120620646	Sirt7	ENSMUSG00000025138	0.471492	0.617077	0.0470011
19	11	121221161	121221235	Hexdc	ENSMUSG00000039307	0.0687797	0.231965	0.000310447
20	11	59209051	59209127	2310033P09Rik	ENSMUSG00000020441	0.0555165	0.136802	0.0456866
21	11	59209200	59209307	2310033P09Rik	ENSMUSG00000020441	0.151263	0.263039	0.0483324
22	11	60708697	60708788	Llgl1	ENSMUSG00000020536	0.060339	0.139268	0.0187479
23	11	69622790	69622859	Sat2	ENSMUSG00000069835	0.0443213	0.141008	0.0205772
24	11	72444977	72445175	Mybbp1a	ENSMUSG00000040463	0.127399	0.275061	0.0207959
25	11	82795786	82796027	Lig3	ENSMUSG00000020697	0.0489522	0.14381	0.0369191
26	11	87777235	87777766	Bzrap1	ENSMUSG00000034156	0.250619	0.323847	0.011719
27	11	94542984	94543293	Rsad1	ENSMUSG00000039096	0.0688726	0.170717	0.0311955

S/N	Chr	Start	End	Gene	Ensembl ID	IR ratio 10w	IR ratio 22m	*p* -value
28	11	99418135	99418273	Krt12	ENSMUSG00000020912	0.520969	0.40939	0.0184528
29	12	112912614	112912692	Jag2	ENSMUSG00000002799	0.109579	0.0422535	0.0320678
30	12	31934441	31936971	Hbp1	ENSMUSG00000002996	0.082885	0.194994	0.028233
31	12	55302795	55302914	Ppp2r3c	ENSMUSG00000021022	0.241737	0.0971338	0.0443258
32	12	56337455	56337763	Mbip	ENSMUSG00000021028	0.149168	0.3046	0.0331206
33	12	56337829	56340200	Mbip	ENSMUSG00000021028	0.289227	0.483462	0.0278681
34	12	69372223	69372323	Arf6	ENSMUSG00000044147	0.276087	0.0670423	0.00111077
35	13	119473956	119474832	4833420G17Rik	ENSMUSG00000062822	0.0712543	0.15506	0.0394664
36	13	13995783	13996892	B3galnt2	ENSMUSG00000039242	0.0827267	0.230846	0.0305328
37	13	14009731	14010541	Tbce	ENSMUSG00000039233	0.269088	0.418655	0.0112468
38	13	55507123	55507329	Pdlim7	ENSMUSG00000021493	0.22163	0.31427	0.0075148
39	13	68611277	68612829	1700001L19Rik	ENSMUSG00000021534	0.0670931	0.135608	0.0221557
40	13	92685238	92688618	Serinc5	ENSMUSG00000021703	0.0468479	0.107125	0.0369929
41	14	31010409	31010863	Glt8d1	ENSMUSG00000021916	0.427369	0.261126	0.0135582
42	14	31011030	31011120	Glt8d1	ENSMUSG00000021916	0.2443	0.115348	0.00480167
43	14	31013565	31013652	Gnl3	ENSMUSG00000042354	0.0302645	0.131095	0.00400137
44	14	31014227	31014589	Gnl3	ENSMUSG00000042354	0.0763586	0.145106	0.042444
45	14	55660098	55660167	Mdp1	ENSMUSG00000002329	0.549027	0.414195	0.00612163
46	14	55676836	55676927	Gmpr2	ENSMUSG00000002326	0.0727156	0.169867	0.011209
47	14	55898012	55898242	Sdr39u1	ENSMUSG00000022223	0.216431	0.323038	0.00142162
48	15	102523050	102523184	Tarbp2	ENSMUSG00000023051	0.2482	0.391437	0.0387076
49	15	74582987	74584377	Adgrb1	ENSMUSG00000034730	0.161734	0.127258	0.00514972
50	15	75892753	75893104	Naprt	ENSMUSG00000022574	0.0524496	0.234637	0.0366442
51	15	75928543	75928891	Tsta3	ENSMUSG00000022570	0.0918639	0.21193	0.0129173
52	15	76497829	76497951	Hsf1	ENSMUSG00000022556	0.103399	0.182222	0.0244921
53	15	76537786	76537916	Fbxl6	ENSMUSG00000022559	0.227537	0.388483	0.00902392
54	15	78908058	78908352	Sh3bp1	ENSMUSG00000022436	0.176579	0.0725981	0.0357607
55	15	79055416	79055494	Ankrd54	ENSMUSG00000033055	0.0430237	0.127935	0.0290813
56	15	89186427	89186753	Dennd6b	ENSMUSG00000015377	0.165665	0.253103	0.0286276
57	15	89186819	89186955	Dennd6b	ENSMUSG00000015377	0.0483271	0.132576	0.0021883
58	15	89187050	89187147	Dennd6b	ENSMUSG00000015377	0.524551	0.651426	0.0421526
59	15	89373602	89373682	Sco2	ENSMUSG00000091780	0.0576923	0.161211	0.00654877
60	15	98595042	98595126	Adcy6	ENSMUSG00000022994	0.0517241	0.151694	0.00916438
61	15	98815740	98815875	Prkag1	ENSMUSG00000067713	0.109145	0.162716	0.0250282
62	15	99319017	99319130	Fmnl3	ENSMUSG00000023008	0.0363636	0.14	0.0360215
63	16	17276887	17276961	Tmem191c	ENSMUSG00000055692	0.167027	0.211619	0.0408693
64	16	20207793	20208418	Yeats2	ENSMUSG00000041215	0.0479403	0.165932	0.0328076
65	16	20740056	20741189	Chrd	ENSMUSG00000006958	0.0494878	0.140698	0.0458235
66	16	4765889	4765977	Hmox2	ENSMUSG00000004070	0.152443	0.207844	0.018294
67	16	5248830	5248911	Eef2kmt	ENSMUSG00000022544	0.00452284	0.111111	0.0447856
68	16	91030556	91034097	Paxbp1	ENSMUSG00000022974	0.18189	0.366463	0.00987089
69	16	91034287	91034799	Paxbp1	ENSMUSG00000022974	0.217837	0.368217	0.0314626
70	17	23676273	23676518	Tnfrsf12a	ENSMUSG00000023905	0.0192308	0.118639	0.0486961
71	17	24510075	24510248	Traf7	ENSMUSG00000052752	0.110476	0.222716	0.00306887
72	17	24510698	24511314	Traf7	ENSMUSG00000052752	0.0553983	0.104023	0.0497962
73	17	25791676	25792052	Fam173a	ENSMUSG00000057411	0.245348	0.316182	0.039434
74	17	25827356	25827427	Wdr24	ENSMUSG00000025737	0.25	0.143687	0.0322953
75	17	25835056	25835136	Rhbdl1	ENSMUSG00000025735	0.248463	0.329807	0.0126218

S/N	Chr	Start	End	Gene	Ensembl ID	IR ratio 10w	IR ratio 22m	*p* -value
76	17	25835893	25835968	Rhbdl1	ENSMUSG00000025735	0.192263	0.137251	0.0327887
77	17	25860817	25860906	Wdr90	ENSMUSG00000073434	0.235294	0.676118	0.022845
78	17	25868543	25868624	Fam195a	ENSMUSG00000025732	0.108499	0.341444	0.0123513
79	17	25876122	25876219	Mettl26	ENSMUSG00000025731	0.163035	0.0963257	0.0222997
80	17	26204383	26204486	Rgs11	ENSMUSG00000024186	0.0614587	0.134103	0.046628
81	17	26208079	26208163	Rgs11	ENSMUSG00000024186	0.279214	0.110012	0.00287925
82	17	28059391	28060561	Anks1	ENSMUSG00000024219	0.45036	0.232729	0.00557743
83	17	29038624	29040774	Srsf3	ENSMUSG00000071172	0.0656293	0.119295	0.00619764
84	17	29705323	29705488	Ccdc167	ENSMUSG00000024018	0.368547	0.496752	0.0295369
85	17	33818444	33818525	Kank3	ENSMUSG00000042099	0.166231	0.325581	0.0416096
86	17	33822375	33822674	Kank3	ENSMUSG00000042099	0.135685	0.32053	0.00246406
87	17	34026935	34027015	H2-Ke6	ENSMUSG00000073422	0.101977	0.179039	0.012406
88	17	34587214	34587373	Notch4	ENSMUSG00000015468	0.00534045	0.153846	0.0400386
89	17	34590563	34590741	Gpsm3	ENSMUSG00000034786	0.349282	0.0312595	0.0348664
90	17	34838683	34838812	Dxo	ENSMUSG00000040482	0.0769788	0.163796	0.0334087
91	17	34845912	34846026	Skiv2l	ENSMUSG00000040356	0.133183	0.0700148	0.0247605
92	17	35670031	35670189	Gtf2h4	ENSMUSG00000001524	0.0244558	0.101167	0.00946961
93	17	43664265	43666899	Slc25a27	ENSMUSG00000023912	0.107143	0.199212	0.0493686
94	17	44082920	44085204	Enpp5	ENSMUSG00000023960	0.426723	0.535236	0.000437992
95	17	55956339	55956621	Ebi3	ENSMUSG00000003206	0.209222	0.0136433	0.023195
96	17	57236239	57236373	Gpr108	ENSMUSG00000005823	0.0298113	0.120956	0.00747775
97	17	78919843	78919976	Cebpzos	ENSMUSG00000062691	0.191602	0.312288	0.00118435
98	17	80204387	80205261	Srsf7	ENSMUSG00000024097	0.168262	0.268779	0.00363155
99	18	23651648	23653328	Dtna	ENSMUSG00000024302	0.0428532	0.106245	0.0119471
100	18	31944205	31944435	Lims2	ENSMUSG00000024395	0.749306	0.543726	0.0257295
101	18	34651462	34651546	Cdc23	ENSMUSG00000024370	0.0487555	0.142103	0.0232603
102	18	36676622	36676864	Sra1	ENSMUSG00000006050	0.157735	0.244442	0.0313041
103	18	37958571	37958758	Rell2	ENSMUSG00000044024	0.078823	0.149104	6.31258E-06
104	18	74217489	74217760	Cxxc1	ENSMUSG00000024560	0.102564	0.174269	0.038635
105	18	74218655	74218731	Cxxc1	ENSMUSG00000024560	0.0328559	0.104985	0.0124431
106	18	9986355	9986552	Thoc1	ENSMUSG00000024287	0.318698	0.488854	0.0358276
107	19	41945815	41946916	Mms19	ENSMUSG00000025159	0.061441	0.129127	0.0267315
108	19	4264108	4264313	Ssh3	ENSMUSG00000034616	0.157157	0.253889	0.0398144
109	19	5051254	5051353	Rin1	ENSMUSG00000024883	0.0498202	0.13465	0.0178944
110	19	5083101	5085297	Tmem151a	ENSMUSG00000061451	0.106332	0.140641	0.0356497
111	19	5495648	5495896	Snx32	ENSMUSG00000056185	0.106383	0.165299	0.00359424
112	19	56916242	56916323	Afap1l2	ENSMUSG00000025083	0.157895	0.00040225	0.0153264
113	19	6052479	6052547	Syvn1	ENSMUSG00000024807	0.104583	0.0361871	0.00897098
114	19	7230106	7230187	Naa40	ENSMUSG00000024764	0.366812	0.570752	0.0208097
115	19	8743135	8743352	Stx5a	ENSMUSG00000010110	0.0643366	0.106791	0.0434056
116	19	8748711	8748806	Stx5a	ENSMUSG00000010110	0.090065	0.151845	0.0228501
117	2	104608972	104609090	Cstf3	ENSMUSG00000027176	0.121281	0.0723459	0.0416301
118	2	104664163	104664296	Cstf3	ENSMUSG00000027176	0.119078	0.222608	0.045534
119	2	104664357	104664716	Cstf3	ENSMUSG00000027176	0.104446	0.226053	0.016188
120	2	11748489	11748936	Fbxo18	ENSMUSG00000058594	0.0687604	0.119351	0.0249836
121	2	11781519	11783640	Ankrd16	ENSMUSG00000047909	0.282948	0.448728	0.0443028
122	2	120031651	120031840	Jmjd7	ENSMUSG00000098789	0.0526316	0.333942	0.0320964
123	2	121196242	121196734	Tubgcp4	ENSMUSG00000027263	0.310943	0.462561	0.0406439

S/N	Chr	Start	End	Gene	Ensembl ID	IR ratio 10w	IR ratio 22m	*p* -value
124	2	131193687	131194114	Cdc25b	ENSMUSG00000027330	0.249471	0.413071	0.0465857
125	2	155634000	155634079	Myh7b	ENSMUSG00000074652	0.0625	0.242537	0.0343359
126	2	156067337	156067454	Spag4	ENSMUSG00000038180	0.0294785	0.285714	0.0452661
127	2	160761648	160761738	Plcg1	ENSMUSG00000016933	0.169567	0.263111	0.00618646
128	2	160761923	160762093	Plcg1	ENSMUSG00000016933	0.210065	0.328608	0.00716687
129	2	174120667	174120947	Npepl1	ENSMUSG00000039263	0.132071	0.282836	0.0199203
130	2	181339094	181339162	Rtel1	ENSMUSG00000038685	0.0176152	0.24838	0.0043026
131	2	181355552	181355650	Rtel1	ENSMUSG00000038685	0.0540541	0.211268	0.0295373
132	2	181382544	181382615	Lime1	ENSMUSG00000090077	0.0473738	0.121362	0.00975047
133	2	181572486	181573073	Uckl1	ENSMUSG00000089917	0.0487549	0.126785	0.030002
134	2	25250003	25250095	Ndor1	ENSMUSG00000006471	0.0673317	0.209302	0.0302919
135	2	25272128	25272297	Ssna1	ENSMUSG00000026966	0.233109	0.168061	0.0190732
136	2	25353516	25353607	Dpp7	ENSMUSG00000026958	0.213225	0.324488	0.0450563
137	2	25888858	25890848	Kcnt1	ENSMUSG00000058740	0.290166	0.388407	0.045883
138	2	26958567	26960223	Rexo4	ENSMUSG00000052406	0.0662172	0.112757	0.0498851
139	2	29081077	29083532	Ttf1	ENSMUSG00000026803	0.0753392	0.243899	0.0409334
140	2	30348617	30349225	Sh3glb2	ENSMUSG00000026860	0.0860872	0.112079	0.0364859
141	2	31612558	31612836	Fubp3	ENSMUSG00000026843	0.094735	0.184673	0.0334148
142	2	32377362	32377508	Ciz1	ENSMUSG00000039205	0.337588	0.227017	0.0117975
143	2	34772146	34772228	Hspa5	ENSMUSG00000026864	0.442941	0.541755	0.00422339
144	2	69654373	69655498	Bbs5	ENSMUSG00000063145	0.0704518	0.165594	0.0309826
145	2	91278957	91279189	1110051M20Rik	ENSMUSG00000040591	0.0706786	0.113536	0.0101833
146	3	103171865	103171947	Bcas2	ENSMUSG00000005687	0.11066	0.172047	0.0176657
147	3	108905334	108907786	Prpf38b	ENSMUSG00000027881	0.104318	0.219383	0.0105215
148	3	157543244	157544963	Zranb2	ENSMUSG00000028180	0.06263	0.100332	0.00835433
149	3	158022950	158026724	Srsf11	ENSMUSG00000055436	0.163825	0.287866	0.00046872
150	3	28666228	28667898	Tnik	ENSMUSG00000027692	0.201803	0.365468	0.000746233
151	3	32561586	32563011	Mfn1	ENSMUSG00000027668	0.0782794	0.144052	0.0193875
152	3	89209421	89209504	Mtx1	ENSMUSG00000064068	0.155902	0.242816	0.0155782
153	3	89210448	89210583	Mtx1	ENSMUSG00000064068	0.113366	0.197769	0.00407339
154	3	94488279	94488526	Celf3	ENSMUSG00000028137	0.161222	0.204769	0.044957
155	3	95004754	95005758	Pi4kb	ENSMUSG00000038861	0.0392392	0.109311	0.00227005
156	3	95337265	95338345	Setdb1	ENSMUSG00000015697	0.20453	0.353381	0.0386391
157	3	95884237	95884969	BC028528	ENSMUSG00000038543	0.0670797	0.26379	0.0497826
158	4	115998615	115998820	Faah	ENSMUSG00000034171	0.0679385	0.102896	0.0426941
159	4	117889931	117890017	Dph2	ENSMUSG00000028540	0.025974	0.106977	0.0380243
160	4	124854016	124854474	Yrdc	ENSMUSG00000028889	0.0389425	0.100458	0.00405623
161	4	126237059	126237155	Map7d1	ENSMUSG00000028849	0.23681	0.418678	1.06268E-05
162	4	127027518	127029826	Sfpq	ENSMUSG00000028820	0.100324	0.183996	0.000314767
163	4	129621150	129622599	Ccdc28b	ENSMUSG00000028795	0.0771903	0.143134	0.0326075
164	4	132337910	132338050	Rcc1	ENSMUSG00000028896	0.0230415	0.104167	0.0453582
165	4	152312256	152312389	Rnf207	ENSMUSG00000058498	0.0246244	0.189982	0.0382876
166	4	154895037	154895125	Mmel1	ENSMUSG00000058183	0.0100825	0.210526	0.042081
167	4	154961301	154961391	Hes5	ENSMUSG00000048001	0.247624	0.0886076	0.00260692
168	4	154991382	154992532	Plch2	ENSMUSG00000029055	0.0593559	0.121761	0.0283538
169	4	155885703	155886091	Cpsf3l	ENSMUSG00000029034	0.0774657	0.170133	0.0085987
170	4	155887521	155887604	Cpsf3l	ENSMUSG00000029034	0.0470588	0.122708	0.01265
171	4	155887962	155888047	Cpsf3l	ENSMUSG00000029034	0.0420379	0.112567	0.0282364

S/N	Chr	Start	End	Gene	Ensembl ID	IR ratio 10w	IR ratio 22m	*p* -value
172	4	156231173	156231546	Klhl17	ENSMUSG00000078484	0.0785543	0.149358	0.0355644
173	4	21869615	21870351	Pnisr	ENSMUSG00000028248	0.232757	0.357813	0.00360129
174	4	21874672	21874912	Pnisr	ENSMUSG00000028248	0.333758	0.249882	0.0441916
175	4	34571018	34571728	Orc3	ENSMUSG00000040044	0.129057	0.207413	0.0419427
176	4	34572572	34575031	Orc3	ENSMUSG00000040044	0.0948811	0.180451	0.013548
177	4	34575173	34576351	Orc3	ENSMUSG00000040044	0.139345	0.27071	0.00276343
178	4	41758238	41758502	Galt	ENSMUSG00000036073	0.0826508	0.148696	0.00115845
179	4	43008913	43009147	Fancg	ENSMUSG00000028453	0.0152644	0.165808	0.0419613
180	4	43570190	43570345	Gba2	ENSMUSG00000028467	0.144143	0.221371	0.0131248
181	4	49632211	49632364	Rnf20	ENSMUSG00000028309	0.628463	0.361279	0.00220086
182	4	62422300	62422414	Prpf4	ENSMUSG00000066148	0.0241287	0.117076	0.0233368
183	5	110104658	110104889	Gtpbp6	ENSMUSG00000033434	0.191568	0.249788	0.038344
184	5	110151686	110152335	Chfr	ENSMUSG00000014668	0.0533562	0.132915	0.0366753
185	5	110152490	110153117	Chfr	ENSMUSG00000014668	0.103934	0.20389	0.0270699
186	5	110162781	110164005	Chfr	ENSMUSG00000014668	0.135531	0.26419	0.0346051
187	5	117386858	117387868	Rfc5	ENSMUSG00000029363	0.0429168	0.149238	0.0469538
188	5	122718384	122718500	P2rx4	ENSMUSG00000029470	0.161715	0.0670661	0.0337599
189	5	123523381	123524070	Diablo	ENSMUSG00000029433	0.263558	0.442132	0.02677
190	5	124114645	124114714	Ogfod2	ENSMUSG00000023707	0.0835851	0.171429	0.00990262
191	5	135375485	135375554	Nsun5	ENSMUSG00000000916	0.0961538	0.239062	0.0372863
192	5	137372217	137372384	Ephb4	ENSMUSG00000029710	0.136986	0.00119284	0.0202855
193	5	137554140	137554512	Actl6b	ENSMUSG00000029712	0.165677	0.292902	0.0120717
194	5	137642013	137642095	Sap25	ENSMUSG00000079165	0.00689655	0.154013	0.0424491
195	5	138176118	138176209	Ap4m1	ENSMUSG00000019518	0.0539895	0.141308	0.0409362
196	5	138177973	138178068	Ap4m1	ENSMUSG00000019518	0.236711	0.477312	0.0154204
197	5	24438949	24439055	Slc4a2	ENSMUSG00000028962	0.293536	0.158273	0.019301
198	5	24439145	24439231	Slc4a2	ENSMUSG00000028962	0.226989	0.113373	0.0347675
199	5	3630112	3630188	Pex1	ENSMUSG00000005907	0.132623	0.259971	0.0493686
200	5	73639514	73639788	Sgcb	ENSMUSG00000029156	0.0662103	0.142939	0.00100101
201	6	115619916	115620208	Raf1	ENSMUSG00000000441	0.0796051	0.124054	0.0381742
202	6	146557622	146560125	Asun	ENSMUSG00000040250	0.0744611	0.165704	0.0413477
203	6	30742220	30742527	Mest	ENSMUSG00000051855	0.112053	0.192142	0.0163027
204	6	30742852	30743013	Mest	ENSMUSG00000051855	0.113816	0.215848	0.00867979
205	6	30743072	30744353	Mest	ENSMUSG00000051855	0.0806437	0.164431	0.00631061
206	6	30744932	30745081	Mest	ENSMUSG00000051855	0.154068	0.220596	0.0310226
207	6	30745183	30745829	Mest	ENSMUSG00000051855	0.130768	0.22873	0.0127286
208	6	30745906	30746261	Mest	ENSMUSG00000051855	0.109836	0.234043	0.00852586
209	6	30749899	30750537	Copg2	ENSMUSG00000025607	0.155564	0.216316	0.0207997
210	6	90405443	90406017	Cfap100	ENSMUSG00000048794	0.0182768	0.307659	0.0301408
211	7	102015835	102016042	Il18bp	ENSMUSG00000070427	0.205416	0.489086	0.0439968
212	7	113553813	113561375	Far1	ENSMUSG00000030759	0.0705356	0.132557	0.0231456
213	7	141064555	141064640	B4galnt4	ENSMUSG00000055629	0.057652	0.102015	0.031045
214	7	141191194	141191341	Hras	ENSMUSG00000025499	0.415696	0.361964	0.00382473
215	7	16178795	16178950	Meis3	ENSMUSG00000041420	0.0801782	0.149247	0.0283313
216	7	24912155	24912309	Arhgef1	ENSMUSG00000040940	0.0638298	0.180489	0.00868527
217	7	28815352	28818460	Hnrnpl	ENSMUSG00000015165	0.137149	0.174008	0.0464375
218	7	29225259	29225471	Kcnk6	ENSMUSG00000046410	0.0146341	0.189189	0.0471441
219	7	30583755	30583916	Kmt2b	ENSMUSG00000006307	0.119769	0.0447855	0.0476407

S/N	Chr	Start	End	Gene	Ensembl ID	IR ratio 10w	IR ratio 22m	*p* -value
220	7	31063175	31063284	Lgi4	ENSMUSG00000036560	0.117014	0.0484528	0.0180917
221	7	31117136	31117223	Scn1b	ENSMUSG00000019194	0.26152	0.241833	0.0456812
222	7	45834481	45834638	Grin2d	ENSMUSG00000002771	0.749083	0.528992	0.0166591
223	7	80098272	80098770	Idh2	ENSMUSG00000030541	0.047237	0.112902	0.00771467
224	7	80285630	80285833	Vps33b	ENSMUSG00000030534	0.0522627	0.140894	0.0249311
225	8	105331290	105331369	Fhod1	ENSMUSG00000014778	0.083004	0.335878	0.0331519
226	8	111075255	111075415	Clec18a	ENSMUSG00000033633	0.022536	0.23506	0.0385715
227	8	122470356	122475862	Rnf166	ENSMUSG00000014470	0.0629849	0.163278	0.0247238
228	8	122482991	122483056	Piezo1	ENSMUSG00000014444	0.421053	0.0952381	0.0223149
229	8	3512865	3514782	Mcoln1	ENSMUSG00000004567	0.096264	0.141622	0.0193072
230	8	3514913	3515013	Mcoln1	ENSMUSG00000004567	0.0646507	0.122085	0.00939481
231	8	4265276	4266555	Timm44	ENSMUSG00000002949	0.100053	0.19025	0.00194102
232	8	45980492	45980572	Ufsp2	ENSMUSG00000031634	0.10873	0.0657114	0.0287156
233	8	70028774	70029207	Mau2	ENSMUSG00000031858	0.0724925	0.146169	0.00597943
234	8	70597768	70598012	Ssbp4	ENSMUSG00000070003	0.11617	0.181528	0.00212209
235	8	70598617	70598701	Ssbp4	ENSMUSG00000070003	0.256667	0.323542	0.0116868
236	8	71621896	71622702	Colgalt1	ENSMUSG00000034807	0.306827	0.46713	0.0105412
237	8	83721852	83722229	Ddx39	ENSMUSG00000005481	0.280432	0.434325	0.0313095
238	9	106435704	106435783	Acy1	ENSMUSG00000023262	0.0108485	0.129744	0.0182602
239	9	107756992	107759775	Rbm5	ENSMUSG00000032580	0.226561	0.328174	0.0250692
240	9	107759849	107760321	Rbm5	ENSMUSG00000032580	0.260048	0.410895	0.00147141
241	9	108051960	108052034	Rnf123	ENSMUSG00000041528	0.104179	0.0505939	0.0154605
242	9	108299458	108299744	Amt	ENSMUSG00000032607	0.195924	0.312825	0.0442893
243	9	108571864	108571940	Dalrd3	ENSMUSG00000019039	0.299889	0.237276	0.032269
244	9	110391681	110391766	Ptpn23	ENSMUSG00000036057	0.0416667	0.13121	0.0258719
245	9	111228626	111229839	Mlh1	ENSMUSG00000032498	0.122426	0.319258	0.0254184
246	9	113956068	113956706	Ubp1	ENSMUSG00000009741	0.254861	0.345675	0.0423795
247	9	20869653	20871196	A230050P20Rik	ENSMUSG00000038884	0.140588	0.208159	0.0122813
248	9	21073318	21073388	Fdx1l	ENSMUSG00000079677	0.389951	0.287785	0.0441914
249	9	21419548	21419624	Qtrt1	ENSMUSG00000002825	0.155779	0.29771	0.0131774
250	9	35217747	35217928	Fam118b	ENSMUSG00000050471	0.101313	0.184701	0.0378107
251	9	37417502	37417690	Robo3	ENSMUSG00000032128	0.0453155	0.137036	0.0155276
252	9	44412483	44412916	Ccdc84	ENSMUSG00000043923	0.394337	0.663705	0.0427166
253	9	44413201	44413442	Ccdc84	ENSMUSG00000043923	0.21067	0.458703	0.028044
254	9	45859059	45859278	Bace1	ENSMUSG00000032086	0.215225	0.281131	0.0353713
255	9	45859450	45860053	Bace1	ENSMUSG00000032086	0.152369	0.215571	0.0215636
256	9	48474513	48475119	Rexo2	ENSMUSG00000032026	0.212042	0.263468	0.0275391
257	9	50576705	50577760	Il18	ENSMUSG00000039217	0.0948961	0.186775	0.015786
258	9	73036821	73038622	Pigb	ENSMUSG00000079469	0.0174105	0.331638	0.000988998
259	9	97445713	97454582	Clstn2	ENSMUSG00000032452	0.409536	0.552184	0.00970771
260	X	101652208	101655623	Ogt	ENSMUSG00000034160	0.159896	0.278715	0.000135383
261	X	134080437	134081190	Cstf2	ENSMUSG00000031256	0.23875	0.340659	0.0191541
262	X	160529015	160529914	Phka2	ENSMUSG00000031295	0.0840734	0.245388	0.0227003
263	X	20851812	20851929	Araf	ENSMUSG00000001127	0.143426	0.119053	0.0340804
264	X	73657343	73657437	Pnck	ENSMUSG00000002012	0.0631703	0.116503	0.00685457
265	X	73767881	73767957	Plxnb3	ENSMUSG00000031385	0.273282	0.10828	0.0427998
266	X	74021963	74022184	Irak1	ENSMUSG00000031392	0.0552234	0.133958	0.00852865
267	X	74288714	74289104	Taz	ENSMUSG00000009995	0.205946	0.285422	0.0468243

S/N	Chr	Start	End	Gene	Ensembl ID	IR ratio 10w	IR ratio 22m	*p* -value
268	X	74333846	74334224	Plxna3	ENSMUSG00000031398	0.096603	0.308842	0.0355854
269	X	74339625	74339774	Plxna3	ENSMUSG00000031398	0.0106762	0.153143	0.0119493

Table S3: Differential retained introns between 2 weeks & 22 months mouse frontal cortex (Lister et al, 2013)

S/N	Chr	Start	End	Gene	GeneID	IR ratio 2w	IR ratio 22m	*p* -value
1	1	36701901	36702058	Actr1b	ENSMUSG00000037351	0.109256	0.0763732	0.0045921
2	1	37437478	37438681	Unc50	ENSMUSG00000026111	0.0697683	0.119267	0.0206279
3	1	38053718	38053839	Rev1	ENSMUSG00000026082	0.00349127	0.109848	0.000338569
4	1	75441827	75441984	Gmppa	ENSMUSG00000033021	0.0867148	0.206151	0.00054853
5	1	75442031	75442184	Gmppa	ENSMUSG00000033021	0.12125	0.218837	0.0221419
6	1	75507518	75509333	Inha	ENSMUSG00000032968	0.286056	0.424783	0.0221359
7	1	91424486	91427738	Per2	ENSMUSG00000055866	0.0106805	0.121902	0.0401769
8	10	127185172	127185286	Arhgef25	ENSMUSG00000019467	0.138129	0.0756809	0.0021906
9	10	127191039	127191335	Dtx3	ENSMUSG00000040415	0.219939	0.161327	0.000698579
10	10	127296633	127296716	Mars	ENSMUSG00000040354	0.271848	0.124412	0.000143834
11	10	127520171	127520337	Shmt2	ENSMUSG00000025403	0.228544	0.0690432	0.00503148
12	10	128124262	128124334	Baz2a	ENSMUSG00000040054	0.151713	0.0352113	0.00545483
13	10	128315657	128315775	Pan2	ENSMUSG00000005682	0.0127496	0.15547	0.0368281
14	10	128318342	128318944	Pan2	ENSMUSG00000005682	0.058225	0.167519	0.026786
15	10	20261983	20266735	Map7	ENSMUSG00000019996	0.0326198	0.10135	0.0235527
16	10	41488097	41488176	Smpd2	ENSMUSG00000019822	0.213419	0.0958904	0.028975
17	10	61342949	61343040	Pald1	ENSMUSG00000020092	0.0103586	0.121302	0.039791
18	10	75509833	75510031	Gucd1	ENSMUSG00000033416	0.0738162	0.193901	0.0383744
19	10	78589745	78589901	Syde1	ENSMUSG00000032714	0.2	0.04	0.0354938
20	10	79621187	79622254	Shc2	ENSMUSG00000020312	0.0853572	0.175018	0.0138356
21	10	79737509	79737599	Polrmt	ENSMUSG00000020329	0.167931	0.0900099	0.044938
22	10	79918917	79919426	Kiss1r	ENSMUSG00000035773	0.146991	0.0138867	0.0439158
23	10	80004850	80004953	Abca7	ENSMUSG00000035722	0.170213	0.0401969	0.0483639
24	10	80027727	80027820	Hmha1	ENSMUSG00000035697	0.25	0.00683761	0.0209931
25	10	80170764	80170981	Cirbp	ENSMUSG00000045193	0.111684	0.0766527	0.0329606
26	10	80347625	80348355	Adamtsl5	ENSMUSG00000043822	1	0.0424935	0.000714369
27	10	80393018	80393094	Mbd3	ENSMUSG00000035478	0.181425	0.110048	0.00022641
28	10	80862362	80862698	Sppl2b	ENSMUSG00000035206	0.0814148	0.141977	0.0356677
29	10	80866660	80867399	Sppl2b	ENSMUSG00000035206	0.155439	0.233768	0.0451916
30	10	81394150	81394775	Smim24	ENSMUSG00000078439	0.00268197	0.173222	0.0214335
31	10	83611066	83611142	Appl2	ENSMUSG00000020263	0.123007	0.197917	0.0407415
32	11	101092591	101092723	Psmc3ip	ENSMUSG00000019303	0.00860421	0.18734	0.0106971
33	11	101183582	101183851	Cntnap1	ENSMUSG00000017167	0.11418	0.0426867	7.1262E-06
34	11	101253807	101254030	Vps25	ENSMUSG00000078656	0.133089	0.0845826	0.0354254
35	11	103339276	103339454	Arhgap27	ENSMUSG00000034255	0.2	0.0416792	0.0371508
36	11	106378963	106380666	Icam2	ENSMUSG00000001029	0.00746509	0.134467	0.0195152
37	11	113657149	113657374	Cog1	ENSMUSG00000018661	0.136556	0.0388287	0.000816862
38	11	115476027	115476121	Armc7	ENSMUSG00000057219	0.24758	0.0952381	0.0228743
39	11	115588003	115588311	Gga3	ENSMUSG00000020740	0.219314	0.143829	0.0389379
40	11	115815238	115815326	Tsen54	ENSMUSG00000020781	0.333333	0.0789474	0.0119843
41	11	116138621	116138710	Mrpl38	ENSMUSG00000020775	0.18056	0.091653	0.0102708
42	11	119040159	119040347	Cbx8	ENSMUSG00000025578	0.101773	0.0165054	0.0271207
43	11	120008444	120009274	Aatk	ENSMUSG00000025375	0.126907	0.238871	4.05385E-07

S/N	Chr	Start	End	Gene	GeneID	IR ratio 2w	IR ratio 22m	*p* -value
44	11	120067038	120067129	Cep131	ENSMUSG00000039781	0.128903	0.0400979	0.0185829
45	11	120374651	120375416	Faap100	ENSMUSG00000025384	0.0329787	0.117747	0.019974
46	11	120612083	120612462	Pcyt2	ENSMUSG00000025137	0.237042	0.152039	0.00381146
47	11	120620504	120620646	Sirt7	ENSMUSG00000025138	0.416085	0.617077	0.00791719
48	11	120642962	120643158	Pycr1	ENSMUSG00000025140	0.00089526	0.22449	0.021883
49	11	120717902	120718036	Lrrc45	ENSMUSG00000025145	0.429569	0.579568	0.0286039
50	11	120730523	120730617	Cbr2	ENSMUSG00000025150	0.2	0.0147884	0.0278265
51	11	120788596	120788669	Gps1	ENSMUSG00000025156	0.174881	0.13752	0.00974505
52	11	121221337	121221500	Hexdc	ENSMUSG00000039307	0.183711	0.269601	0.0265704
53	11	32214109	32214181	Rhbdf1	ENSMUSG00000020282	0.00564301	0.158031	0.00207516
54	11	3524726	3524882	Smtn	ENSMUSG00000020439	0.0529248	0.20464	0.0476005
55	11	4139858	4139934	Rnf215	ENSMUSG00000003581	0.149798	0.252369	0.0325701
56	11	5078952	5079484	Ewsr1	ENSMUSG00000009079	0.135854	0.101626	0.0470098
57	11	5116924	5128869	Emid1	ENSMUSG00000034164	0.0120965	0.254937	0.00109609
58	11	5801839	5802998	Pgam2	ENSMUSG00000020475	0.497174	0.17977	0.000745823
59	11	5803179	5803274	Pgam2	ENSMUSG00000020475	0.181287	0.0580737	0.0331675
60	11	60194032	60197788	Rai1	ENSMUSG00000062115	0.118468	0.0660214	0.0403005
61	11	61450902	61450977	Rnf112	ENSMUSG00000010086	0.392641	0.313002	0.000239393
62	11	62318030	62319365	Ncor1	ENSMUSG00000018501	0.0695276	0.140209	0.000176442
63	11	62321217	62321391	Ncor1	ENSMUSG00000018501	0.15603	0.253421	0.00154208
64	11	62321445	62325486	Ncor1	ENSMUSG00000018501	0.0808219	0.159335	0.0014402
65	11	69330226	69330396	Kcnab3	ENSMUSG00000018470	0.13455	0.0313885	0.000337177
66	11	69557492	69559691	Efnb3	ENSMUSG00000003934	0.0607748	0.117593	0.0146443
67	11	69915954	69916032	Gps2	ENSMUSG00000023170	0.171975	0.0933621	0.0236838
68	11	70216051	70216136	Slc16a11	ENSMUSG00000040938	0.426684	0.283177	0.0435037
69	11	74905316	74905890	Tsr1	ENSMUSG00000038335	0.694505	0.842672	0.00935962
70	11	75524137	75525369	Scarf1	ENSMUSG00000038188	0.0206371	0.406277	0.00869774
71	11	78034136	78034274	Dhrs13	ENSMUSG00000020834	0.00396558	0.102538	0.0127362
72	11	78315108	78315187	Spag5	ENSMUSG00000002055	0.0119332	0.246753	0.00592054
73	11	78320672	78321042	Spag5	ENSMUSG00000002055	0.222462	0.0243902	0.01356
74	11	78470851	78471370	Slc46a1	ENSMUSG00000020829	0.621037	0.383057	0.00710527
75	11	78540998	78541360	Ift20	ENSMUSG00000001105	0.0663142	0.103064	0.030185
76	11	82795452	82795708	Lig3	ENSMUSG00000020697	0.0667773	0.16088	0.0295672
77	11	83766160	83767077	Heatr6	ENSMUSG00000000976	0.315094	0.0596792	0.0414359
78	11	84821468	84821664	Dhrs11	ENSMUSG00000034449	0.00166134	0.121951	0.01381
79	11	87589818	87589955	Sep-04	ENSMUSG00000020486	0.136907	0.0744922	0.000120331
80	11	9010417	9010496	Hus1	ENSMUSG00000020413	0.0005571	0.230681	0.00678369
81	11	9010624	9011058	Hus1	ENSMUSG00000020413	0.777487	0.43682	0.0370291
82	11	94542984	94543293	Rsad1	ENSMUSG00000039096	0.0208375	0.170717	0.000996405
83	11	98716525	98717708	Med24	ENSMUSG00000017210	0.139687	0.0774493	0.00585547
84	11	98717765	98717909	Med24	ENSMUSG00000017210	0.15413	0.105727	0.0360045
85	11	99418135	99418273	Krt12	ENSMUSG00000020912	0.686452	0.40939	8.13396E-06
86	12	105809662	105811036	Papola	ENSMUSG00000021111	0.139732	0.275206	0.00177108
87	12	109034351	109035327	Begain	ENSMUSG00000040867	0.118713	0.0726782	0.00280969
88	12	112123039	112123289	Aspg	ENSMUSG00000037686	1	0.0604491	0.0037
89	12	112912614	112912692	Jag2	ENSMUSG00000002799	0.184674	0.0422535	0.000462491
90	12	112976982	112977058	Btbd6	ENSMUSG00000002803	0.262579	0.188101	0.0449921
91	12	31327821	31329109	Lamb1	ENSMUSG00000002900	0.14778	0.415156	0.00085673

S/N	Chr	Start	End	Gene	GeneID	IR ratio 2w	IR ratio 22m	*p* -value
92	12	31329269	31329356	Lamb1	ENSMUSG00000002900	0.149264	0.30855	0.0144684
93	12	56337455	56337763	Mbip	ENSMUSG00000021028	0.138877	0.3046	0.0396311
94	12	56340297	56340384	Mbip	ENSMUSG00000021028	0.152344	0.313867	0.0135343
95	12	79076596	79076705	Plekhh1	ENSMUSG00000060716	0.145833	0.0185827	0.0305633
96	12	79147765	79149868	Arg2	ENSMUSG00000021125	0.0335957	0.507725	0.000332086
97	12	79151057	79151921	Arg2	ENSMUSG00000021125	0.0114861	0.29541	0.00195313
98	12	80169016	80170182	Actn1	ENSMUSG00000015143	0.120554	0.0754926	0.0243624
99	12	80637610	80640956	Erh	ENSMUSG00000021131	0.0444051	0.177123	0.0347977
100	13	111686354	111687827	Mier3	ENSMUSG00000032727	1	0.0448493	0.0168411
101	13	13995783	13996892	B3galnt2	ENSMUSG00000039242	0.0711133	0.230846	0.018448
102	13	13998181	13998362	Tbce	ENSMUSG00000039233	0.142631	0.246956	0.00499213
103	13	19370367	19370479	Stard3nl	ENSMUSG00000003062	0.0693026	0.144018	0.00047148
104	13	24831929	24832009	Tdp2	ENSMUSG00000035958	0.122257	0.0385802	0.0462465
105	13	49203789	49203928	Card19	ENSMUSG00000037960	0.430788	0.199348	0.0215617
106	13	49464786	49465678	Cenpp	ENSMUSG00000021391	0.0684211	0.384654	0.0377654
107	13	51682154	51682443	Secisbp2	ENSMUSG00000035139	0.199717	0.031116	0.00794535
108	13	55320553	55320744	Rab24	ENSMUSG00000034789	0.192615	0.0940407	0.000014226
109	13	55507366	55507487	Pdlim7	ENSMUSG00000021493	0.349804	0.176755	2.54378E-05
110	13	55531684	55531831	Ddx41	ENSMUSG00000021494	0.10292	0.0497653	0.03221
111	13	55534488	55535288	Ddx41	ENSMUSG00000021494	0.214244	0.139055	0.0377811
112	13	55535619	55535729	Ddx41	ENSMUSG00000021494	0.121821	0.0326481	0.000751857
113	13	55535804	55535885	Ddx41	ENSMUSG00000021494	0.145781	0.0638596	0.0124204
114	13	55536406	55536480	Ddx41	ENSMUSG00000021494	0.102302	0.00156006	0.00446124
115	13	68611277	68612829	1700001L19Rik	ENSMUSG00000021534	0.0505791	0.135608	0.00121368
116	13	9689248	9689451	Zmynd11	ENSMUSG00000021156	0.106198	0.0653012	0.00396144
117	13	98272852	98273377	Ankra2	ENSMUSG00000021661	0.167307	0.241199	0.0349511
118	14	119029042	119032193	Uggt2	ENSMUSG00000042104	0.00806684	0.101973	0.0399226
119	14	123972231	123973284	Itgbl1	ENSMUSG00000032925	0.332148	0.751498	0.00607043
120	14	14095601	14095699	Atxn7	ENSMUSG00000021738	0.186441	0.0404569	0.0144217
121	14	20719672	20719812	Zswim8	ENSMUSG00000021819	0.209608	0.103261	0.000703047
122	14	30917787	30918274	Itih3	ENSMUSG00000006522	0.428388	0.149066	0.00484725
123	14	31011030	31011120	Glt8d1	ENSMUSG00000021916	0.257927	0.115348	0.00357608
124	14	31260547	31261045	Dnah1	ENSMUSG00000019027	0.551487	0.128639	0.0380918
125	14	31261280	31261488	Dnah1	ENSMUSG00000019027	0.683794	0.209798	0.0293727
126	14	31265055	31265262	Dnah1	ENSMUSG00000019027	1	0.0728606	0.00782966
127	14	31266030	31266314	Dnah1	ENSMUSG00000019027	0.356725	0.0480179	0.0371817
128	14	45371091	45372384	Styx	ENSMUSG00000053205	0.0134248	0.136565	0.0433735
129	14	52296581	52296663	Mettl3	ENSMUSG00000022160	0.0951276	0.250669	0.00691652
130	14	55494893	55495403	Carmil3	ENSMUSG00000022211	0.0390273	0.113664	0.0385136
131	14	55898012	55898242	Sdr39u1	ENSMUSG00000022223	0.194312	0.323038	0.000119211
132	14	70193459	70193560	Sorbs3	ENSMUSG00000022091	0.125	0.00361627	0.00209715
133	14	70547780	70547939	Reep4	ENSMUSG00000033589	0.04	0.261884	0.00713849
134	15	102109433	102109512	Tns2	ENSMUSG00000037003	0.125	0.0254201	0.0354938
135	15	102178832	102178960	Csad	ENSMUSG00000023044	0.138584	0.0537634	0.0467009
136	15	31606188	31608095	Fam173b	ENSMUSG00000039065	0.00948115	0.13165	0.0260123
137	15	74582987	74584377	Adgrb1	ENSMUSG00000034730	0.0652778	0.127258	6.44849E-08
138	15	75893220	75893305	Naprt	ENSMUSG00000022574	0.0180033	0.234568	0.03125
139	15	75893847	75894016	Naprt	ENSMUSG00000022574	0.0290076	0.283898	0.046656

S/N	Chr	Start	End	Gene	GeneID	IR ratio 2w	IR ratio 22m	*p* -value
140	15	75925662	75925792	Tsta3	ENSMUSG00000022570	0.17639	0.0953637	0.023717
141	15	75928543	75928891	Tsta3	ENSMUSG00000022570	0.103691	0.21193	0.0241373
142	15	76049301	76049456	Scrib	ENSMUSG00000022568	0.220856	0.0776337	0.00684386
143	15	76537006	76537089	Fbxl6	ENSMUSG00000022559	0.147727	0.0737941	0.0168749
144	15	76596349	76596415	Cpsf1	ENSMUSG00000034022	0.221355	0.125428	0.00983943
145	15	79531914	79535956	Ddx17	ENSMUSG00000055065	0.330406	0.468473	3.23462E-05
146	15	82374056	82374297	Cyp2d22	ENSMUSG00000061740	0.00954014	0.105584	0.0201396
147	15	98122263	98122629	Pfkm	ENSMUSG00000033065	0.103203	0.0678482	0.001132
148	15	98815740	98815875	Prkag1	ENSMUSG00000067713	0.0838323	0.162716	0.00441148
149	15	99305193	99305407	Prpf40b	ENSMUSG00000023007	0.0490756	0.110229	0.046436
150	16	10419685	10420255	Nubp1	ENSMUSG00000022503	0.130792	0.334748	0.000271877
151	16	17277039	17277193	Tmem191c	ENSMUSG00000055692	0.267496	0.182971	0.000448298
152	16	17803600	17803736	Scarf2	ENSMUSG00000012017	0.432727	0.0714286	0.0316375
153	16	18795226	18795824	Cdc45	ENSMUSG00000000028	0.248886	0.0106215	0.0403814
154	16	20642373	20642472	Ece2	ENSMUSG00000022842	0.156535	0.0251497	0.0167468
155	16	20643733	20643852	Ece2	ENSMUSG00000022842	0.133891	0.0361446	0.0247733
156	16	20707632	20707842	Clcn2	ENSMUSG00000022843	0.214373	0.146644	0.0288249
157	16	20709315	20709569	Clcn2	ENSMUSG00000022843	0.173834	0.0867793	0.0109322
158	16	20734586	20734685	Chrd	ENSMUSG00000006958	0.0239264	0.145215	0.00251995
159	16	43869097	43871620	Qtrtd1	ENSMUSG00000022704	0.0727249	0.206897	0.0422901
160	16	44370019	44370237	Spice1	ENSMUSG00000043065	0.218605	0.0318332	0.01774
161	16	44745493	44745998	Gtpbp8	ENSMUSG00000022668	0.117165	0.291242	0.00423777
162	16	5011692	5012240	Rogdi	ENSMUSG00000022540	0.142681	0.0971483	0.00764367
163	16	5198936	5199615	Nagpa	ENSMUSG00000023143	0.22523	0.0701312	0.0334782
164	17	17836744	17837508	Spaca6	ENSMUSG00000080316	0.381962	0.213899	0.0443193
165	17	17838243	17838315	Spaca6	ENSMUSG00000080316	0.792	0.338983	0.000513051
166	17	23735372	23735562	Pkmyt1	ENSMUSG00000023908	0.249289	0.541693	0.0407565
167	17	24206947	24207207	Tbc1d24	ENSMUSG00000036473	0.279496	0.484631	0.0386534
168	17	24207443	24207565	Tbc1d24	ENSMUSG00000036473	0.169032	0.333333	0.0196413
169	17	24499620	24500696	Caskin1	ENSMUSG00000033597	0.0680769	0.105053	0.0465506
170	17	24510075	24510248	Traf7	ENSMUSG00000052752	0.113301	0.222716	0.0014668
171	17	24529970	24530054	Rab26	ENSMUSG00000079657	0.111804	0.0509915	0.00733021
172	17	24530709	24530785	Rab26	ENSMUSG00000079657	0.11194	0.0500146	0.00572801
173	17	24680798	24680880	Zfp598	ENSMUSG00000041130	0.117117	0.0390461	0.0160265
174	17	24890460	24890567	Spsb3	ENSMUSG00000024160	0.190426	0.0634987	3.02708E-07
175	17	24890755	24890832	Spsb3	ENSMUSG00000024160	0.115624	0.071699	0.0137131
176	17	24890935	24891010	Spsb3	ENSMUSG00000024160	0.149454	0.0794913	0.0011871
177	17	24896758	24896851	Nme3	ENSMUSG00000073435	0.174012	0.0894245	0.00683699
178	17	25235111	25235181	Gnptg	ENSMUSG00000035521	0.166635	0.102804	0.00272232
179	17	25239842	25239913	Gnptg	ENSMUSG00000035521	0.400518	0.260628	0.00146771
180	17	25384614	25384694	Cacna1h	ENSMUSG00000024112	0.0294118	0.12	0.0244888
181	17	25784178	25784259	Haghl	ENSMUSG00000061046	0.128884	0.0719352	0.000425192
182	17	25791486	25791571	Fam173a	ENSMUSG00000057411	0.270111	0.193685	0.00959367
183	17	25827356	25827427	Wdr24	ENSMUSG00000025737	0.251222	0.143687	0.0246337
184	17	25827532	25827652	Wdr24	ENSMUSG00000025737	0.172083	0.0845443	0.0253368
185	17	25835056	25835136	Rhbdl1	ENSMUSG00000025735	0.271001	0.329807	0.0332649
186	17	25835893	25835968	Rhbdl1	ENSMUSG00000025735	0.0900859	0.137251	0.0127614
187	17	25840751	25840835	Rhot2	ENSMUSG00000025733	0.138024	0.0545079	5.77492E-06

S/N	Chr	Start	End	Gene	GeneID	IR ratio 2w	IR ratio 22m	*p* -value
188	17	25855234	25855300	Wdr90	ENSMUSG00000073434	0.64532	0.239437	0.0275292
189	17	25860817	25860906	Wdr90	ENSMUSG00000073434	0.4	0.676118	0.0402478
190	17	25876122	25876219	Mettl26	ENSMUSG00000025731	0.198856	0.0963257	0.00141035
191	17	26204927	26205107	Rgs11	ENSMUSG00000024186	0.0907571	0.2033	0.00932871
192	17	26934535	26934630	Phf1	ENSMUSG00000024193	0.143955	0.0689468	0.0348186
193	17	26935818	26935918	Phf1	ENSMUSG00000024193	0.135231	0.0455331	0.0254059
194	17	27113925	27114079	Itpr3	ENSMUSG00000042644	0.333333	0.0127971	0.0197754
195	17	27114505	27114846	Itpr3	ENSMUSG00000042644	1	0.0223371	0.00346021
196	17	27199438	27201636	Lemd2	ENSMUSG00000044857	0.143878	0.28422	0.0343915
197	17	28059391	28060561	Anks1	ENSMUSG00000024219	0.393189	0.232729	0.0170213
198	17	28599869	28600242	Srpk1	ENSMUSG00000004865	0.241767	0.163743	0.025876
199	17	28984200	28988177	Stk38	ENSMUSG00000024006	0.0894461	0.210083	0.0363033
200	17	32047731	32048552	Rrp1b	ENSMUSG00000058392	0.0546139	0.248824	0.0196616
201	17	33821914	33822005	Kank3	ENSMUSG00000042099	0.0289193	0.125	0.00473884
202	17	34024418	34024509	Ring1	ENSMUSG00000024325	0.102785	0.0120956	0.00375802
203	17	34026935	34027015	H2-Ke6	ENSMUSG00000073422	0.105354	0.179039	0.0268354
204	17	34183744	34184281	Psmb9	ENSMUSG00000096727	0.5	0.108199	0.0285273
205	17	34590563	34590741	Gpsm3	ENSMUSG00000034786	0.262473	0.0312595	0.0466521
206	17	34838683	34838812	Dxo	ENSMUSG00000040482	0.0230394	0.163796	0.000364139
207	17	34838907	34838986	Dxo	ENSMUSG00000040482	0.0649949	0.196143	0.00254366
208	17	34845912	34846026	Skiv2l	ENSMUSG00000040356	0.131665	0.0700148	0.0266344
209	17	34846173	34846521	Skiv2l	ENSMUSG00000040356	0.243657	0.138644	0.00566198
210	17	35004893	35004976	Vars	ENSMUSG00000007029	0.118222	0.0428816	0.0139219
211	17	35266726	35266865	H2-D1	ENSMUSG00000073411	0.123555	0.0852957	0.0478097
212	17	35660127	35660213	Vars2	ENSMUSG00000038838	0.00317965	0.110178	0.0026439
213	17	36257524	36257605	Rpp21	ENSMUSG00000024446	0.125966	0.0646733	0.0151634
214	17	37011517	37012062	Mog	ENSMUSG00000076439	0.133681	0.0426449	0.0158501
215	17	44082920	44085204	Enpp5	ENSMUSG00000023960	0.638339	0.535236	0.00691089
216	17	45540089	45541132	Tcte1	ENSMUSG00000023949	0.654886	0.0928595	0.00114849
217	17	57236740	57236870	Gpr108	ENSMUSG00000005823	0.147059	0.0570506	0.0356838
218	17	57237497	57237850	Gpr108	ENSMUSG00000005823	0.167986	0.079646	0.0341066
219	17	78919843	78919976	Cebpzos	ENSMUSG00000062691	0.210672	0.312288	0.00652581
220	17	78920630	78920721	Cebpz	ENSMUSG00000024081	0.0634146	0.178423	0.00291118
221	18	31944205	31944435	Lims2	ENSMUSG00000024395	0.939037	0.543726	5.63941E-05
222	18	35660353	35660443	Spata24	ENSMUSG00000024352	0.146667	0.0503226	0.0436112
223	18	36677256	36677444	Sra1	ENSMUSG00000006050	0.196279	0.0972956	0.0219617
224	18	36762757	36762848	Wdr55	ENSMUSG00000042660	0.13	0.0316602	0.00989165
225	18	37958389	37958511	Rell2	ENSMUSG00000044024	0.114653	0.0624228	0.00010692
226	18	37958571	37958758	Rell2	ENSMUSG00000044024	0.116543	0.149104	0.0355918
227	18	37963037	37963127	Fchsd1	ENSMUSG00000038524	0.0068605	0.125	0.0224532
228	18	38258186	38258269	0610009O20Rik	ENSMUSG00000024442	0.127989	0.0434783	0.0115629
229	18	67227719	67227803	Mppe1	ENSMUSG00000062526	0.00852619	0.145265	0.00396796
230	19	10215810	10216060	Myrf	ENSMUSG00000036098	0.127273	0.0395869	0.0473999
231	19	23975653	23977961	Fam189a2	ENSMUSG00000071604	0.245473	0.0947219	0.013631
232	19	36970038	36972897	Btaf1	ENSMUSG00000040565	0.00149333	0.698316	2.67467E-13
233	19	43665828	43666914	Slc25a28	ENSMUSG00000040414	0.459284	0.289126	0.00441674
234	19	45008375	45009279	Twnk	ENSMUSG00000025209	0.0901787	0.224294	0.0271748
235	19	47129782	47129947	Pdcd11	ENSMUSG00000025047	0.0322581	0.122606	0.0367382

S/N	Chr	Start	End	Gene	GeneID	IR ratio 2w	IR ratio 22m	*p* -value
236	19	5049032	5049406	Brms1	ENSMUSG00000080268	0.0556805	0.118184	0.0439848
237	19	5083101	5085297	Tmem151a	ENSMUSG00000061451	0.0974928	0.140641	0.0104837
238	19	5495648	5495896	Snx32	ENSMUSG00000056185	0.0800645	0.165299	4.98036E-05
239	19	5495982	5496057	Snx32	ENSMUSG00000056185	0.10255	0.0356397	6.79553E-05
240	19	5661111	5661595	Sipa1	ENSMUSG00000056917	0.0640394	0.291309	0.0452675
241	19	5665247	5665328	Pcnx3	ENSMUSG00000054874	0.171429	0.063354	0.0126564
242	19	5903772	5904239	Dpf2	ENSMUSG00000024826	0.114943	0.0602332	0.0236006
243	19	6052479	6052547	Syvn1	ENSMUSG00000024807	0.108312	0.0361871	0.00950683
244	19	6063446	6063526	Tm7sf2	ENSMUSG00000024799	0.0629556	0.138103	0.0236773
245	19	6063607	6063821	Tm7sf2	ENSMUSG00000024799	0.0865611	0.161869	0.0248951
246	19	6063990	6064078	Tm7sf2	ENSMUSG00000024799	0.0588235	0.172414	0.00223608
247	19	61226654	61226805	Csf2ra	ENSMUSG00000059326	0.234629	0.624506	0.00460643
248	19	6954299	6954380	Plcb3	ENSMUSG00000024960	0.100443	0.0178739	0.0167862
249	19	8748711	8748806	Stx5a	ENSMUSG00000010110	0.0885841	0.151845	0.0228501
250	19	8936459	8936618	Eml3	ENSMUSG00000071647	0.828812	0.480114	0.0287457
251	2	104664163	104664296	Cstf3	ENSMUSG00000027176	0.0925926	0.222608	0.00748372
252	2	112640673	112641010	Ryr3	ENSMUSG00000057378	0.134497	0.368089	0.0300732
253	2	112667896	112668044	Ryr3	ENSMUSG00000057378	0.0160428	0.284672	0.0171794
254	2	118759256	118759507	A430105I19Rik	ENSMUSG00000045838	0.388013	0.00910426	0.0123457
255	2	118869782	118871377	Ivd	ENSMUSG00000027332	0.0700607	0.141898	0.0258204
256	2	118871547	118872884	Ivd	ENSMUSG00000027332	0.058413	0.12832	0.0210216
257	2	119752156	119752230	Ltk	ENSMUSG00000027297	0.514472	0.291805	0.00372799
258	2	120030542	120030815	Gm28042	ENSMUSG00000033852	0.0125156	0.125	0.0390184
259	2	120031651	120031840	Jmjd7	ENSMUSG00000098789	0.0411559	0.333942	0.0060636
260	2	120494941	120495235	Capn3	ENSMUSG00000079110	0.474654	0.0181951	0.00228366
261	2	120723017	120723191	Cdan1	ENSMUSG00000027284	0.486381	0.229469	0.0102928
262	2	127248084	127248491	Tmem127	ENSMUSG00000034850	0.273508	0.369305	0.0357375
263	2	129206486	129207609	Slc20a1	ENSMUSG00000027397	0.14735	0.0993598	0.0253303
264	2	131040475	131041015	Gfra4	ENSMUSG00000027316	0.25774	0.36078	0.00924879
265	2	14045255	14055730	Hacd1	ENSMUSG00000063275	0.0156996	0.108595	0.00821052
266	2	151632254	151632455	Snph	ENSMUSG00000027457	0.67089	0.49498	0.000395662
267	2	155578428	155578711	Gss	ENSMUSG00000027610	0.0181956	0.125801	0.00469005
268	2	155629680	155630257	Myh7b	ENSMUSG00000074652	0.0208462	0.363806	0.0100788
269	2	155630384	155630474	Myh7b	ENSMUSG00000074652	0.0349819	0.37094	0.0276413
270	2	155632752	155632847	Myh7b	ENSMUSG00000074652	0.1	0.0196488	0.0389531
271	2	160761648	160761738	Plcg1	ENSMUSG00000016933	0.134144	0.263111	0.000242421
272	2	162967085	162967251	L3mbtl1	ENSMUSG00000035576	0.138155	0.292944	0.0444874
273	2	163454228	163454324	Gdap1l1	ENSMUSG00000017943	0.242343	0.312449	0.0483635
274	2	164795179	164795651	Acot8	ENSMUSG00000017307	0.16523	0.346519	0.00800928
275	2	164892606	164892686	Zfp335	ENSMUSG00000039834	0.297211	0.399818	0.040021
276	2	164892952	164893245	Zfp335	ENSMUSG00000039834	0.246949	0.396325	0.00459987
277	2	164893615	164893700	Zfp335	ENSMUSG00000039834	0.241489	0.395366	0.00350002
278	2	180582955	180583400	Mrgbp	ENSMUSG00000027569	0.141995	0.0460653	0.0210177
279	2	181230920	181231007	Helz2	ENSMUSG00000027580	1	0.0555556	0.0037
280	2	181355552	181355650	Rtel1	ENSMUSG00000038685	0.0555556	0.211268	0.0327148
281	2	181382703	181382798	Lime1	ENSMUSG00000090077	0.245491	0.154897	0.0476501
282	2	181597343	181597427	Samd10	ENSMUSG00000038605	0.145647	0.0290864	0.00395617
283	2	181686974	181687613	Tcea2	ENSMUSG00000059540	0.0575221	0.103509	0.0220924

S/N	Chr	Start	End	Gene	GeneID	IR ratio 2w	IR ratio 22m	*p* -value
284	2	20808024	20808709	Etl4	ENSMUSG00000036617	0.126541	0.278534	9.65723E-06
285	2	22935639	22939822	Pdss1	ENSMUSG00000026784	0.031469	0.135824	0.0440421
286	2	25272128	25272297	Ssna1	ENSMUSG00000026966	0.23136	0.168061	0.0164035
287	2	25353217	25353359	Dpp7	ENSMUSG00000026958	0.469809	0.308542	0.0465288
288	2	25354822	25354892	Dpp7	ENSMUSG00000026958	0.341865	0.116883	0.00351877
289	2	25460074	25460166	BC029214	ENSMUSG00000047617	0.327083	0.23708	0.0234506
290	2	25909306	25909593	Kcnt1	ENSMUSG00000058740	0.192787	0.125185	0.0201156
291	2	26591728	26592117	Egfl7	ENSMUSG00000026921	0.192698	0.128562	0.0320964
292	2	26958567	26960223	Rexo4	ENSMUSG00000052406	0.061109	0.112757	0.0359658
293	2	29923639	29925939	Odf2	ENSMUSG00000026790	0.292921	0.416712	0.0415071
294	2	30177721	30177919	Spout1	ENSMUSG00000039660	0.498822	0.37059	0.0292184
295	2	30347104	30348554	Sh3glb2	ENSMUSG00000026860	0.144545	0.106167	0.0044211
296	2	30348617	30349225	Sh3glb2	ENSMUSG00000026860	0.150472	0.112079	0.0139645
297	2	32377362	32377508	Ciz1	ENSMUSG00000039205	0.145863	0.227017	0.0235147
298	2	32377630	32377963	Ciz1	ENSMUSG00000039205	0.131824	0.224872	0.00709611
299	2	32571295	32572296	Dpm2	ENSMUSG00000026810	0.0467979	0.110565	0.00015591
300	2	5866241	5866353	Nudt5	ENSMUSG00000025817	0.139	0.0568032	0.0411308
301	2	5893221	5895256	Sec61a2	ENSMUSG00000025816	0.0398986	0.132855	0.0107525
302	2	84737107	84737419	Ypel4	ENSMUSG00000034059	0.265293	0.178343	0.0433392
303	2	84769910	84770092	Serping1	ENSMUSG00000023224	0.230769	0.0227273	0.0128847
304	2	91278957	91279189	1110051M20Rik	ENSMUSG00000040591	0.0773647	0.113536	0.0333368
305	3	10346026	10346109	Zfand1	ENSMUSG00000039795	0.112296	0.0366331	0.0114786
306	3	108075359	108075530	Ampd2	ENSMUSG00000027889	0.137764	0.0832229	0.0398802
307	3	116006935	116007134	Slc30a7	ENSMUSG00000054414	0.00139043	0.110469	0.0272492
308	3	133005432	133005559	Gstcd	ENSMUSG00000028018	0.20339	0.00566572	0.03125
309	3	135329098	135329767	Slc9b2	ENSMUSG00000037994	0.0164179	0.228904	0.0291306
310	3	135329917	135330582	Slc9b2	ENSMUSG00000037994	0.017149	0.230432	0.0431513
311	3	158022950	158026724	Srsf11	ENSMUSG00000055436	0.19528	0.287866	0.010319
312	3	28665839	28666088	Tnik	ENSMUSG00000027692	0.120048	0.205844	0.00593307
313	3	28666228	28667898	Tnik	ENSMUSG00000027692	0.213258	0.365468	0.00206115
314	3	33813200	33814189	Ccdc39	ENSMUSG00000027676	0.252577	0.461451	0.0134036
315	3	33814529	33814654	Ccdc39	ENSMUSG00000027676	0.485895	0.709653	0.0362019
316	3	33814795	33815411	Ccdc39	ENSMUSG00000027676	0.160099	0.447837	0.00496076
317	3	33815518	33815911	Ccdc39	ENSMUSG00000027676	0.13175	0.324222	0.01512
318	3	53473567	53474687	Proser1	ENSMUSG00000049504	0.0341748	0.120665	0.0321988
319	3	80688714	80689102	Gria2	ENSMUSG00000033981	0.254224	0.199949	0.000379335
320	3	87997356	87997744	Bcan	ENSMUSG00000004892	0.140156	0.230083	0.00010266
321	3	88023640	88023764	Hapln2	ENSMUSG00000004894	1	0.210944	0.0103338
322	3	88633042	88633257	Arhgef2	ENSMUSG00000028059	0.171433	0.118479	0.0329789
323	3	89151080	89151424	Hcn3	ENSMUSG00000028051	0.172505	0.337429	0.0133317
324	3	89210183	89210265	Mtx1	ENSMUSG00000064068	0.172066	0.27444	0.00598049
325	3	89219995	89220151	Thbs3	ENSMUSG00000028047	0.00567537	0.244094	0.00518126
326	3	89340472	89340862	Adam15	ENSMUSG00000028041	0.100548	0.0482119	0.00802517
327	3	89343583	89343672	Adam15	ENSMUSG00000028041	0.309963	0.175274	0.000919492
328	3	90259492	90259582	Crtc2	ENSMUSG00000027936	0.151096	0.030575	0.00334467
329	3	94488279	94488526	Celf3	ENSMUSG00000028137	0.159408	0.204769	0.0159998
330	3	94955385	94955745	Rfx5	ENSMUSG00000005774	0.304369	0.134062	0.0327039
331	3	95167476	95167623	Sema6c	ENSMUSG00000038777	0.065534	0.153846	0.0289183

S/N	Chr	Start	End	Gene	GeneID	IR ratio 2w	IR ratio 22m	*p* -value
332	3	95321060	95321189	Cers2	ENSMUSG00000015714	0.116279	0.0426735	0.0113633
333	3	95884237	95884969	BC028528	ENSMUSG00000038543	0.0646473	0.26379	0.0207419
334	4	108620073	108621511	Cc2d1b	ENSMUSG00000028582	0.215399	0.0815466	0.0468838
335	4	109066999	109068362	Osbpl9	ENSMUSG00000028559	0.0435194	0.110112	0.040748
336	4	117002322	117002509	Hectd3	ENSMUSG00000046861	0.0557019	0.117716	0.0181433
337	4	117889931	117890017	Dph2	ENSMUSG00000028540	0.00731452	0.106977	0.0129407
338	4	118372247	118372329	Szt2	ENSMUSG00000033253	0.142857	0.0172414	0.0116653
339	4	118373555	118373743	Szt2	ENSMUSG00000033253	0.335385	0.174556	0.0399015
340	4	118376127	118376332	Szt2	ENSMUSG00000033253	0.0196834	0.107274	0.0209153
341	4	119232469	119232582	AU022252	ENSMUSG00000078584	0.418482	0.666851	0.0430626
342	4	121049715	121050075	Col9a2	ENSMUSG00000028626	0.0911452	0.234609	0.0485029
343	4	121050129	121050354	Col9a2	ENSMUSG00000028626	0.038674	0.181818	0.0398646
344	4	126034339	126034421	Csf3r	ENSMUSG00000028859	1	0.153846	0.00332381
345	4	126317904	126317983	Adprhl2	ENSMUSG00000042558	0.22043	0.112245	0.0137377
346	4	127027518	127029826	Sfpq	ENSMUSG00000028820	0.117922	0.183996	0.00251136
347	4	129620876	129620983	Ccdc28b	ENSMUSG00000028795	0.015748	0.102091	1.6851E-06
348	4	129621150	129622599	Ccdc28b	ENSMUSG00000028795	0.0434838	0.143134	0.00011389
349	4	136995712	136996024	Zbtb40	ENSMUSG00000060862	0.166667	0.0171598	0.043965
350	4	141010721	141012825	Mfap2	ENSMUSG00000060572	0.00907402	0.139729	0.04
351	4	141013859	141014055	Mfap2	ENSMUSG00000060572	0.0157508	0.151982	0.00472389
352	4	141019312	141019707	Crocc	ENSMUSG00000040860	0.21875	0.128681	0.0298578
353	4	141465842	141465976	Zbtb17	ENSMUSG00000006215	0.105071	0.0481013	0.0339766
354	4	154961049	154961135	Hes5	ENSMUSG00000048001	0.119928	0.222703	0.0234928
355	4	154961301	154961391	Hes5	ENSMUSG00000048001	0.18239	0.0886076	0.0328291
356	4	155429298	155429874	Cfap74	ENSMUSG00000078490	1	0.265629	0.0222277
357	4	155855025	155855103	Dvl1	ENSMUSG00000029071	0.10324	0.0565659	0.0158948
358	4	155887521	155887604	Cpsf3l	ENSMUSG00000029034	0.0527199	0.122708	0.0166757
359	4	155887767	155887854	Cpsf3l	ENSMUSG00000029034	0.0990099	0.16491	0.0427607
360	4	155890902	155891051	Pusl1	ENSMUSG00000051557	0.0797634	0.196349	0.0026417
361	4	156124001	156124091	9430015G10Rik	ENSMUSG00000059939	0.666667	0.370548	0.0234949
362	4	156173130	156173542	Agrn	ENSMUSG00000041936	0.198262	0.267638	0.0307628
363	4	21874672	21874912	Pnisr	ENSMUSG00000028248	0.385564	0.249882	0.00583748
364	4	34572572	34575031	Orc3	ENSMUSG00000040044	0.108812	0.180451	0.0398372
365	4	41758238	41758502	Galt	ENSMUSG00000036073	0.103015	0.148696	0.0184732
366	4	43426365	43426447	Rusc2	ENSMUSG00000035969	0.280069	0.220183	0.0343195
367	4	43567467	43567724	Gba2	ENSMUSG00000028467	0.10344	0.160145	0.0274891
368	4	43570190	43570345	Gba2	ENSMUSG00000028467	0.112558	0.221371	0.00029159
369	4	49632211	49632364	Rnf20	ENSMUSG00000028309	0.65878	0.361279	0.000364546
370	4	56812388	56812510	Ctnnal1	ENSMUSG00000038816	0.144981	0.00789177	0.019073
371	5	100768455	100770397	Helq	ENSMUSG00000035266	0.152392	0.415189	0.038364
372	5	106636323	106636416	Zfp644	ENSMUSG00000049606	0.623386	0.406931	0.00161222
373	5	108638490	108638666	Tmem175	ENSMUSG00000013495	0.0406742	0.102728	0.024391
374	5	108649925	108650200	Dgkq	ENSMUSG00000004815	0.300164	0.142496	0.00730554
375	5	110104168	110104267	Gtpbp6	ENSMUSG00000033434	0.0832274	0.407853	9.49866E-13
376	5	110104420	110104509	Gtpbp6	ENSMUSG00000033434	0.125828	0.201777	0.00746241
377	5	110104658	110104889	Gtpbp6	ENSMUSG00000033434	0.0960578	0.249788	8.05427E-09
378	5	110105098	110105198	Gtpbp6	ENSMUSG00000033434	0.101284	0.308086	2.1479E-09
379	5	110106783	110106869	Gtpbp6	ENSMUSG00000033434	0.813387	0.625924	0.0125302

S/N	Chr	Start	End	Gene	GeneID	IR ratio 2w	IR ratio 22m	*p* -value
380	5	110151686	110152335	Chfr	ENSMUSG00000014668	0.047183	0.132915	0.00615468
381	5	110152490	110153117	Chfr	ENSMUSG00000014668	0.0737293	0.20389	0.000631924
382	5	110162781	110164005	Chfr	ENSMUSG00000014668	0.0949454	0.26419	0.0011907
383	5	117092856	117092945	Suds3	ENSMUSG00000066900	0.882185	0.760802	0.0255628
384	5	117382860	117383394	Rfc5	ENSMUSG00000029363	0.0443686	0.167768	0.00862138
385	5	117384604	117385387	Rfc5	ENSMUSG00000029363	0.0432049	0.170093	0.0053557
386	5	117387933	117388916	Rfc5	ENSMUSG00000029363	0.0270607	0.221309	0.0037908
387	5	118056438	118056536	Tesc	ENSMUSG00000029359	0.349206	0.230409	0.00152597
388	5	121850546	121851167	Fam109a	ENSMUSG00000044134	0.0458358	0.134517	0.04913
389	5	123523381	123524070	Diablo	ENSMUSG00000029433	0.280537	0.442132	0.0295748
390	5	124000774	124000991	Hip1r	ENSMUSG00000000915	0.120585	0.0529407	0.00332755
391	5	124114192	124114387	Ogfod2	ENSMUSG00000023707	0.106492	0.170507	0.0439598
392	5	124114645	124114714	Ogfod2	ENSMUSG00000023707	0.098689	0.171429	0.0265047
393	5	137313708	137313959	Trip6	ENSMUSG00000023348	0.691574	0.238469	0.0243083
394	5	137518612	137518877	Gigyf1	ENSMUSG00000029714	0.423661	0.256037	0.0413677
395	5	137520800	137520884	Gigyf1	ENSMUSG00000029714	0.413203	0.107632	0.00119788
396	5	138166078	138166682	Mcm7	ENSMUSG00000029730	0.0587011	0.205882	0.031464
397	5	138167439	138167550	Mcm7	ENSMUSG00000029730	0.00605602	0.127295	0.00597424
398	5	138167634	138167840	Mcm7	ENSMUSG00000029730	0.0860873	0.213603	0.0375644
399	5	138168482	138168809	Mcm7	ENSMUSG00000029730	0.047619	0.168262	0.0487721
400	5	138169473	138169559	Mcm7	ENSMUSG00000029730	0.052094	0.199148	0.0089173
401	5	139338375	139342562	Cox19	ENSMUSG00000045438	0.0457663	0.117476	0.00291322
402	5	21270614	21271216	Gsap	ENSMUSG00000039934	0.541867	0.179074	0.0265943
403	5	24439398	24439736	Slc4a2	ENSMUSG00000028962	0.0672599	0.223529	0.0137646
404	5	24441261	24441335	Fastk	ENSMUSG00000028959	0.121038	0.0517276	0.0014495
405	5	24592804	24593016	Smarcd3	ENSMUSG00000028949	0.0945964	0.136067	0.018852
406	5	24593118	24593224	Smarcd3	ENSMUSG00000028949	0.0715394	0.101451	0.0262335
407	5	29224284	29225042	Rnf32	ENSMUSG00000029130	0.00667576	0.124901	0.015625
408	5	30930636	30930715	Khk	ENSMUSG00000029162	0.25	0.0445476	0.00177752
409	5	37311742	37313428	Evc	ENSMUSG00000029122	0.0339994	0.381142	0.0363284
410	5	45532835	45534152	Fam184b	ENSMUSG00000015879	0.0268319	0.203932	0.00912662
411	5	73639514	73639788	Sgcb	ENSMUSG00000029156	0.0510555	0.142939	1.46275E-05
412	5	74597410	74597775	Lnx1	ENSMUSG00000029228	0.359438	0.494787	0.00314611
413	5	77095022	77095150	Hopx	ENSMUSG00000059325	0.110395	0.0627127	0.0495521
414	6	106779041	106779185	Trnt1	ENSMUSG00000013736	0.290684	0.409149	0.00467858
415	6	113283242	113283349	Cpne9	ENSMUSG00000030270	0.408701	0.265811	0.019676
416	6	113321865	113321967	Brpf1	ENSMUSG00000001632	0.32635	0.157661	0.0159676
417	6	113482378	113482496	Il17rc	ENSMUSG00000030281	1	0.169811	0.0104064
418	6	115619916	115620208	Raf1	ENSMUSG00000000441	0.0612331	0.124054	0.00242238
419	6	120518537	120521193	Cecr5	ENSMUSG00000058979	0.139468	0.258945	0.0297146
420	6	125047587	125047792	Ing4	ENSMUSG00000030330	0.0608998	0.138787	0.00253939
421	6	134922083	134924242	Cdkn1b	ENSMUSG00000003031	0.250856	0.383518	0.0148802
422	6	142353598	142354624	Pyroxd1	ENSMUSG00000041671	0.0222596	0.122172	0.0136252
423	6	30742220	30742527	Mest	ENSMUSG00000051855	0.0417715	0.192142	1.06138E-06
424	6	30742605	30742715	Mest	ENSMUSG00000051855	0.0437436	0.204695	1.70273E-06
425	6	30742852	30743013	Mest	ENSMUSG00000051855	0.0149825	0.215848	3.93117E-10
426	6	30743072	30744353	Mest	ENSMUSG00000051855	0.0159155	0.164431	7.39671E-09
427	6	30744932	30745081	Mest	ENSMUSG00000051855	0.056582	0.220596	2.42081E-08

S/N	Chr	Start	End	Gene	GeneID	IR ratio 2w	IR ratio 22m	*p* -value
428	6	30745183	30745829	Mest	ENSMUSG00000051855	0.0380606	0.22873	3.84936E-08
429	6	30745906	30746261	Mest	ENSMUSG00000051855	0.0448853	0.234043	1.28222E-05
430	6	30746325	30747051	Mest	ENSMUSG00000051855	0.0725477	0.26228	1.38304E-06
431	6	30748987	30749405	Copg2	ENSMUSG00000025607	0.192642	0.116349	0.00301435
432	6	30749899	30750537	Copg2	ENSMUSG00000025607	0.299038	0.216316	0.0193629
433	6	59209181	59209338	Tigd2	ENSMUSG00000049232	0.151079	0.0170684	0.0393983
434	6	71596892	71597216	Kdm3a	ENSMUSG00000053470	0.133237	0.0563107	0.0428366
435	6	83054832	83054910	Aup1	ENSMUSG00000068328	0.150496	0.0854083	0.0166557
436	6	83079745	83079921	Pcgf1	ENSMUSG00000069678	0.0392465	0.126362	0.0393647
437	6	83103532	83103611	Ccdc142	ENSMUSG00000107499	0.289796	0.0205047	0.0131306
438	6	86420411	86424344	Tia1	ENSMUSG00000071337	0.0848557	0.2044	0.00134965
439	6	91254621	91256234	Fbln2	ENSMUSG00000064080	0.0211285	0.34787	0.03078
440	6	91266084	91266314	Fbln2	ENSMUSG00000064080	0.0145577	0.215287	0.0144583
441	7	101823646	101823892	Inppl1	ENSMUSG00000032737	0.165775	0.0599078	0.0480665
442	7	105740342	105740512	Ilk	ENSMUSG00000030890	0.0801105	0.119088	0.0188204
443	7	105740608	105740770	Ilk	ENSMUSG00000030890	0.0730947	0.106825	0.0294656
444	7	105740867	105740959	Ilk	ENSMUSG00000030890	0.0608129	0.111253	0.00249298
445	7	105741043	105741147	Ilk	ENSMUSG00000030890	0.0670615	0.107844	0.012599
446	7	105741233	105741373	Ilk	ENSMUSG00000030890	0.0805637	0.12679	0.00899933
447	7	109027115	109027752	Tub	ENSMUSG00000031028	0.0897246	0.135178	0.0227102
448	7	118156735	118156859	Smg1	ENSMUSG00000030655	0.219163	0.124943	0.037447
449	7	126372567	126372744	Spns1	ENSMUSG00000030741	0.085499	0.14635	0.0194711
450	7	126575258	126575343	Cln3	ENSMUSG00000030720	0.205882	0.0578609	0.0127406
451	7	127016282	127016442	Gm42742	ENSMUSG00000107068	0.100793	0.0572669	0.0281832
452	7	127375502	127375642	Zfp747	ENSMUSG00000054381	0.333333	0.037037	0.0123046
453	7	139920825	139920901	Kndc1	ENSMUSG00000066129	0.114236	0.0351617	0.00693523
454	7	140101188	140101331	Fuom	ENSMUSG00000025466	0.0471849	0.145992	0.00180495
455	7	141071114	141071207	B4galnt4	ENSMUSG00000055629	0.119788	0.170032	0.0370146
456	7	141464842	141464938	Cracr2b	ENSMUSG00000048200	0.43083	0.142857	0.0409362
457	7	141491905	141491979	Tspan4	ENSMUSG00000025511	0.0473557	0.121622	0.0134277
458	7	16892356	16892449	Gng8	ENSMUSG00000063594	0.666667	0.0769231	0.013884
459	7	19811408	19811494	Bcl3	ENSMUSG00000053175	1	0.0909091	0.0128847
460	7	24912155	24912309	Arhgef1	ENSMUSG00000040940	0.0729108	0.180489	0.00887352
461	7	27580691	27581350	2310022A10Rik	ENSMUSG00000049643	0.323262	0.488201	0.01941
462	7	28288196	28288300	Selenov	ENSMUSG00000046750	0.634615	0.181818	0.0399248
463	7	28401617	28401823	Samd4b	ENSMUSG00000109336	0.221053	0.372933	0.00297217
464	7	28815352	28818460	Hnrnpl	ENSMUSG00000015165	0.123554	0.174008	0.00523262
465	7	30526500	30527141	Arhgap33	ENSMUSG00000036882	0.04088	0.101455	2.45551E-05
466	7	31063175	31063284	Lgi4	ENSMUSG00000036560	0.177945	0.0484528	0.00317862
467	7	31117136	31117223	Scn1b	ENSMUSG00000019194	0.272901	0.241833	0.0364253
468	7	44898700	44898775	Fuz	ENSMUSG00000011658	0.145119	0.00328587	0.00195313
469	7	44899238	44899311	Fuz	ENSMUSG00000011658	0.43038	0.146816	0.000936249
470	7	4507423	4507521	Tnnt1	ENSMUSG00000064179	0.25	0.0333333	0.0314301
471	7	45134516	45135121	Flt3l	ENSMUSG00000089989	1	0.317963	0.0413641
472	7	45634854	45634966	Rasip1	ENSMUSG00000044562	0.34715	0.636552	0.0116502
473	7	45636581	45636719	Rasip1	ENSMUSG00000044562	0.472081	0.675801	0.0128424
474	7	67733553	67734459	Synm	ENSMUSG00000030554	0.368449	0.21962	0.00865749
475	7	81871490	81873731	Tm6sf1	ENSMUSG00000038623	0.0964864	0.193098	0.0371084

S/N	Chr	Start	End	Gene	GeneID	IR ratio 2w	IR ratio 22m	*p* -value
476	7	90444377	90445424	Crebzf	ENSMUSG00000051451	0.229963	0.484089	0.000019279
477	8	105269996	105270546	Hsf4	ENSMUSG00000033249	0.583815	0.0959368	0.00278678
478	8	105331290	105331369	Fhod1	ENSMUSG00000014778	0.0301205	0.335878	0.0496319
479	8	105697945	105698030	Carmil2	ENSMUSG00000050357	0.159249	0.0694789	0.000665504
480	8	105704249	105704350	Enkd1	ENSMUSG00000013155	0.00658212	0.178508	0.00401235
481	8	123226419	123226945	Cdk10	ENSMUSG00000033862	0.115219	0.0720141	0.0378907
482	8	123227017	123227422	Cdk10	ENSMUSG00000033862	0.176755	0.0864241	0.00166403
483	8	123228900	123229111	Cdk10	ENSMUSG00000033862	0.121255	0.0534813	0.00274455
484	8	123230327	123230593	Cdk10	ENSMUSG00000033862	0.185179	0.0725087	4.08655E-05
485	8	25734886	25735008	Ddhd2	ENSMUSG00000061313	0.155525	0.0918694	0.0343861
486	8	25735111	25735744	Ddhd2	ENSMUSG00000061313	0.14301	0.0694191	0.0223225
487	8	3512865	3514782	Mcoln1	ENSMUSG00000004567	0.0873884	0.141622	0.0077044
488	8	3514913	3515013	Mcoln1	ENSMUSG00000004567	0.0721649	0.122085	0.0329062
489	8	4325288	4327756	Ccl25	ENSMUSG00000023235	0.00993303	0.138862	0.018102
490	8	45980492	45980572	Ufsp2	ENSMUSG00000031634	0.124786	0.0657114	0.0065417
491	8	56294712	56294938	Hpgd	ENSMUSG00000031613	0.112008	0.010838	0.0354656
492	8	69797836	69797940	Atp13a1	ENSMUSG00000031862	0.0330789	0.1	0.0181691
493	8	69798513	69798583	Atp13a1	ENSMUSG00000031862	0.146245	0.265882	0.0127532
494	8	70028774	70029207	Mau2	ENSMUSG00000031858	0.0856197	0.146169	0.011892
495	8	70186776	70188299	Slc25a42	ENSMUSG00000002346	0.171454	0.0738569	0.0346936
496	8	70598617	70598701	Ssbp4	ENSMUSG00000070003	0.43918	0.323542	0.000299656
497	8	71367369	71367643	Use1	ENSMUSG00000002395	0.211919	0.122692	0.00072353
498	8	71484881	71484999	Ano8	ENSMUSG00000034863	0.114306	0.033437	0.000554912
499	8	71621896	71622702	Colgalt1	ENSMUSG00000034807	0.294572	0.46713	0.00174362
500	8	72462490	72462800	Cherp	ENSMUSG00000052488	0.125475	0.048022	0.00311793
501	8	83721852	83722229	Ddx39	ENSMUSG00000005481	0.250651	0.434325	0.0102926
502	8	84868086	84868223	Farsa	ENSMUSG00000003808	0.125964	0.19781	0.0471848
503	8	84998137	84998203	Hook2	ENSMUSG00000052566	0.217391	0.0769231	0.0460821
504	8	85064496	85064580	Fbxw9	ENSMUSG00000008167	0.20373	0.426949	0.00149773
505	8	85066169	85066272	Fbxw9	ENSMUSG00000008167	0.0364794	0.10673	0.00539121
506	8	85075937	85076023	Wdr83	ENSMUSG00000005150	0.155935	0.0667854	0.00961844
507	8	91106983	91107057	Rbl2	ENSMUSG00000031666	0.131403	0.0405904	0.01712
508	9	106435014	106435095	Acy1	ENSMUSG00000023262	0.25	0.0587875	0.0164911
509	9	107980959	107981203	Uba7	ENSMUSG00000032596	0.5	0.0321349	0.0130267
510	9	108299458	108299744	Amt	ENSMUSG00000032607	0.133166	0.312825	0.00481138
511	9	108563691	108563912	Impdh2	ENSMUSG00000062867	0.617117	0.173298	0.0116099
512	9	108571864	108571940	Dalrd3	ENSMUSG00000019039	0.299703	0.237276	0.0441115
513	9	110389163	110389240	Ptpn23	ENSMUSG00000036057	0.107299	0.0522876	0.0484045
514	9	110626630	110626732	Nbeal2	ENSMUSG00000056724	0.00705687	0.190852	0.0171251
515	9	121784240	121784587	Hhatl	ENSMUSG00000032523	0.289474	0.100144	0.0168081
516	9	15317593	15317673	Cep295	ENSMUSG00000046111	0.303483	0.114286	0.0448295
517	9	21073181	21073263	Fdx1l	ENSMUSG00000079677	0.400705	0.267041	0.00315754
518	9	21073318	21073388	Fdx1l	ENSMUSG00000079677	0.52381	0.287785	0.000173913
519	9	21419548	21419624	Qtrt1	ENSMUSG00000002825	0.165354	0.29771	0.0202229
520	9	21590028	21590110	Yipf2	ENSMUSG00000032182	0.242991	0.112172	0.0408275
521	9	21590277	21590355	Yipf2	ENSMUSG00000032182	0.129032	0.0397436	0.014899
522	9	21592218	21592527	Yipf2	ENSMUSG00000032182	0.246244	0.140578	0.0308936
523	9	21960611	21960689	Epor	ENSMUSG00000006235	0.666667	0.261582	0.0366967

S/N	Chr	Start	End	Gene	GeneID	IR ratio 2w	IR ratio 22m	*p* -value
524	9	21990244	21990396	Ccdc151	ENSMUSG00000039632	0.142857	0.00957015	0.014817
525	9	35217747	35217928	Fam118b	ENSMUSG00000050471	0.0649228	0.184701	0.00784987
526	9	35223742	35227163	Fam118b	ENSMUSG00000050471	0.0341554	0.111438	0.014414
527	9	37419808	37420016	Robo3	ENSMUSG00000032128	0.476071	0.794989	0.0103304
528	9	45859059	45859278	Bace1	ENSMUSG00000032086	0.164452	0.281131	9.15946E-05
529	9	45859450	45860053	Bace1	ENSMUSG00000032086	0.112143	0.215571	5.57885E-05
530	9	46273367	46273477	Zpr1	ENSMUSG00000032078	0.214554	0.119731	0.0246673
531	9	48474513	48475119	Rexo2	ENSMUSG00000032026	0.16675	0.263468	5.54657E-05
532	9	49009233	49010242	Usp28	ENSMUSG00000032267	0.160142	0.334762	0.0420599
533	9	53453435	53453531	Atm	ENSMUSG00000034218	0.2	0.03125	0.0343136
534	9	58025606	58026084	Cyp11a1	ENSMUSG00000032323	0.0328453	0.174986	0.0297651
535	9	58026282	58026693	Cyp11a1	ENSMUSG00000032323	0.00914257	0.124207	0.0174262
536	9	59716027	59716207	Gramd2	ENSMUSG00000074259	0.00750626	0.130188	0.00877009
537	9	64307348	64307429	Dis3l	ENSMUSG00000032396	0.0208333	0.130705	0.00169318
538	9	73018083	73021285	Pigb	ENSMUSG00000079469	0.119085	0.373697	0.0182653
539	9	73036821	73038622	Pigb	ENSMUSG00000079469	0.00129657	0.331638	2.74541E-05
540	9	89911592	89915491	Rasgrf1	ENSMUSG00000032356	0.480234	0.635238	0.0349653
541	X	100733739	100733898	Gdpd2	ENSMUSG00000019359	0.00223214	0.150155	0.0310437
542	X	101652208	101655623	Ogt	ENSMUSG00000034160	0.188242	0.278715	0.00629067
543	X	13071733	13072214	Usp9x	ENSMUSG00000031010	0.263595	0.106542	0.010189
544	X	134072474	134072677	Cstf2	ENSMUSG00000031256	0.0871191	0.14799	0.0304232
545	X	150645521	150645693	Tro	ENSMUSG00000025272	0.201954	0.141126	0.0322044
546	X	151088111	151088602	Fgd1	ENSMUSG00000025265	0.604844	0.748943	0.0360752
547	X	20641256	20642743	Rbm10	ENSMUSG00000031060	0.182981	0.103512	0.0431743
548	X	20851812	20851929	Araf	ENSMUSG00000001127	0.167951	0.119053	0.000201398
549	X	73657513	73657719	Pnck	ENSMUSG00000002012	0.126315	0.0365922	1.0231E-06
550	X	74021963	74022184	Irak1	ENSMUSG00000031392	0.07239	0.133958	0.048915
551	X	74226334	74226860	Flna	ENSMUSG00000031328	0.669013	0.416095	0.00126527
552	X	74227335	74227490	Flna	ENSMUSG00000031328	0.475847	0.33134	0.0366958
553	X	74336422	74336491	Plxna3	ENSMUSG00000031398	0.125	0.0108481	0.0216065
554	Y	927891	927969	Kdm5d	ENSMUSG00000056673	0.133333	0.0303676	0.0328535

Explanation

Start: The genomic coordinates of the start of the retained intron.

End: The genomic coordinates of the end of the retained intron.

Gene: The gene that contains the differential retained intron.
