# Supplementary material for: Increased intron retention is a post‐transcriptional signature associated with progressive aging and Alzheimer’s disease
Source: Aging Cell. 2019 Mar 13;18(3):e12928. doi: 10.1111/acel.12928 (PMC6516162; doi:10.1111/acel.12928)
Supplement: Supplementary file 5 [file ACEL-18-e12928-s005.pdf]

Table S4: DAVID functional annotation chart of differential IR genes between Young & Old mouse hippocampus (Stilling et al, 2014)

GOTERM_BP_ALL	Count	%	*P-Value*	Fold Enrichment	Benjamini value
GO:0090305~nucleic acid phosphodiester bond hydrolysis	5	5.494505	0.001280429	10.47381835	0.896990726
GO:0043112~receptor metabolic process	5	5.494505	0.005170874	7.119458225	0.98993272
GO:0031623~receptor internalization	4	4.395604	0.00615356	10.62709374	0.9740101
GO:0001919~regulation of receptor recycling	3	3.296703	0.007113804	23.34165232	0.957838492
GO:0006396~RNA processing	10	10.98901	0.007703361	2.84778329	0.935670323
GO:0044237~cellular metabolic process	56	61.53846	0.011832331	1.245650767	0.970379475
GO:0031668~cellular response to extracellular stimulus	5	5.494505	0.013213039	5.419289097	0.96564064
GO:0071840~cellular component organization or biogenesis	37	40.65934	0.030106569	1.335207984	0.998862239
GO:0008152~metabolic process	59	64.83516	0.030806789	1.181385099	0.997904288
GO:0090501~RNA phosphodiester bond hydrolysis	3	3.296703	0.032233021	10.54139137	0.997009527
GO:0016071~mRNA metabolic process	7	7.692308	0.032702594	2.888234757	0.995309431
GO:0043170~macromolecule metabolic process	50	54.94505	0.034464801	1.225835143	0.9943996
GO:0033572~transferrin transport	2	2.197802	0.035715342	54.46385542	0.993007619
GO:0071496~cellular response to external stimulus	5	5.494505	0.036798591	3.94665619	0.991355793
GO:0042254~ribosome biogenesis	5	5.494505	0.037630151	3.91826298	0.989286908
GO:0022613~ribonucleoprotein complex biogenesis	6	6.593407	0.040653931	3.142145505	0.989965137
GO:0009987~cellular process	79	86.81319	0.042056723	1.07077573	0.988708476
GO:0023051~regulation of signaling	20	21.97802	0.044232943	1.551676793	0.988423265
GO:0051304~chromosome separation	3	3.296703	0.044471217	8.831976555	0.985698146
GO:0031123~RNA 3'-end processing	3	3.296703	0.045558113	8.714216867	0.984012528
GO:0035690~cellular response to drug	3	3.296703	0.047761179	8.487873572	0.98398477
GO:0090304~nucleic acid metabolic process	30	32.96703	0.049280596	1.360179532	0.983008883

Table S4: Human orthologs of the differential mouse IR genes identified in aging hippocampus (Stilling et al., 2014)

S/N	Mus Gene ID	MGI ID	Mouse Symbol	Species	Human GeneID	HGNC ID	Human Symbol	DIOPT Score	Weighted Score	Rank	Best Score
1	81898	1932339	Sf3b1	Human	23451	10768	SF3B1	14	14.29	high	Yes
2	75619	1922869	Fastkd2	Human	22868	29160	FASTKD2	15	15.3	high	Yes
3	226432	1918944	Ipo9	Human	55705	19425	IPO9	15	15.24	high	Yes
4	66155	1913405	Ufc1	Human	51506	26941	UFC1	15	15.24	high	Yes
5	103425	1926081	Ncln	Human	56926	26923	NCLN	15	15.3	high	Yes
6	59013	1891925	Hnrnph1	Human	3187	5041	HNRNPH1	13	13.38	high	Yes
7	22671	106611	Rnf112	Human	7732	12968	RNF112	12	12.43	high	Yes
8	68626	1890496	Elac2	Human	60528	14198	ELAC2	15	15.24	high	Yes
9	56310	1891751	Gps2	Human	2874	4550	GPS2	14	14.29	high	Yes
10	72149	1919399	Strada	Human	92335	30172	STRADA	16	16.25	high	Yes
11	170472	2156841	Recql5	Human	9400	9950	RECQL5	15	15.24	high	Yes
12	73389	894659	Hbp1	Human	26959	23200	HBP1	14	14.29	high	Yes
13	107522	1101356	Ece2	Human	9718	13275	ECE2	14	14.31	high	Yes
14	22042	98822	Tfrc	Human	7037	11763	TFRC	16	16.25	high	Yes
15	224619	3042141	Traf7	Human	84231	20456	TRAF7	14	14.29	high	Yes
16	268933	2446285	Wdr24	Human	84219	20852	WDR24	13	13.29	high	Yes
17	67676	1914926	Rpp21	Human	79897	21300	RPP21	14	14.29	high	Yes
18	269033	1922045	4930503L19Rik	Human	162681	13796	C18orf54	12	12.21	high	Yes
19	107392	2388804	Brms1	Human	25855	17262	BRMS1	15	15.3	high	Yes
20	170791	2157953	Rbm39	Human	9584	15923	RBM39	14	14.29	high	Yes
21	69639	1916889	Exosc8	Human	11340	17035	EXOSC8	15	15.22	high	Yes
22	14800	95809	Gria2	Human	2891	4572	GRIA2	15	15.3	high	Yes
23	229503	2387197	Rrnad1	Human	51093	24273	RRNAD1	14	14.29	high	Yes
24	66625	1913875	Pnisr	Human	25957	21222	PNISR	14	14.29	high	Yes
25	69940	2445020	Exoc1	Human	55763	30380	EXOC1	14	14.29	high	Yes
26	69663	1916913	Ddx51	Human	317781	20082	DDX51	14	14.28	high	Yes
27	233895	2384565	Prr14	Human	78994	28458	PRR14	14	14.29	high	Yes
28	69697	1916947	Camsap3	Human	57662	29307	CAMSAP3	15	15.3	high	Yes
29	212139	2384831	Cc2d1a	Human	54862	30237	CC2D1A	15	15.3	high	Yes
30	11459	87902	Acta1	Human	58	129	ACTA1	15	15.3	high	Yes
31	13430	109547	Dnm2	Human	1785	2974	DNM2	15	15.24	high	Yes

S/N	Mus Gene ID	MGI ID	Mouse Symbol	Species	Human GeneID	HGNC ID	Human Symbol	DIOPT Score	Weighted Score	Rank	Best Score
32	104444	1888981	Rexo2	Human	25996	17851	REXO2	15	15.24	high	Yes
33	53623	95810	Gria3	Human	2892	4573	GRIA3	15	15.24	high	Yes
34	216136	1351911	Ilvbl	Human	10994	6041	ILVBL	14	14.21	high	Yes
35	80904	2135752	Dtx3	Human	196403	24457	DTX3	15	15.3	high	Yes
36	216456	2143539	Gls2	Human	27165	29570	GLS2	16	16.25	high	Yes
37	23943	1344426	Esyt1	Human	23344	29534	ESYT1	14	14.4	high	Yes
38	14799	95808	Gria1	Human	2890	4571	GRIA1	15	15.3	high	Yes
39	14756	106604	Gpld1	Human	2822	4459	GPLD1	15	15.24	high	Yes
40	19336	105065	Rab24	Human	53917	9765	RAB24	15	15.3	high	Yes
41	67399	1914649	Pdlim7	Human	9260	22958	PDLIM7	15	15.24	high	Yes
42	239510	2444412	Phf20l1	Human	51105	24280	PHF20L1	15	15.3	high	Yes
43	223646	2442664	Naprt	Human	93100	30450	NAPRT	14	14.29	high	Yes
44	66656	1913906	Eef1d	Human	1936	3211	EEF1D	14	14.29	high	Yes
45	13350	1333825	Dgat1	Human	8694	2843	DGAT1	14	14.31	high	Yes
46	268822	2679274	Adck5	Human	203054	21738	ADCK5	16	16.25	high	Yes
47	94230	2679722	Cpsf1	Human	29894	2324	CPSF1	14	14.31	high	Yes
48	12651	1328313	Chkb	Human	1120	1938	CHKB	14	14.31	high	Yes
49	54003	1858510	Nell2	Human	4753	7751	NELL2	14	14.29	high	Yes
50	72168	1919418	Aifm3	Human	150209	26398	AIFM3	15	15.24	high	Yes
51	50817	1355075	Capn15	Human	6650	11182	CAPN15	15	15.3	high	Yes
52	224656	2687278	Zfp523	Human	7629	13149	ZNF76	12	12.29	high	Yes
53	16549	1336214	Khsrp	Human	8570	6316	KHSRP	12	12.35	high	Yes
54	277250	1923356	Kdm3b	Human	51780	1337	KDM3B	14	14.27	high	Yes
55	71711	1918961	Mus81	Human	80198	29814	MUS81	14	14.29	high	Yes
56	20469	107576	Sipa1	Human	6494	10885	SIPA1	15	15.3	high	Yes
57	56613	1930076	Rps6ka4	Human	8986	10433	RPS6KA4	15	15.29	high	Yes
58	12982	1339754	Csf2ra	Human	1438	2435	CSF2RA	13	13.26	high	Yes
59	71435	1918685	Arhgap21	Human	57584	23725	ARHGAP21	14	14.29	high	Yes
60	68427	1915677	Slc39a13	Human	91252	20859	SLC39A13	15	15.24	high	Yes
61	99010	2138993	Lpcat4	Human	254531	30059	LPCAT4	16	16.25	high	Yes
62	67145	1914395	Tomm34	Human	10953	15746	TOMM34	14	14.29	high	Yes
63	241915	2181434	Phc3	Human	80012	15682	PHC3	16	16.25	high	Yes

S/N	Mus Gene ID	MGI ID	Mouse Symbol	Species	Human GeneID	HGNC ID	Human Symbol	DIOPT Score	Weighted Score	Rank	Best Score
64	360213	2673000	Trim46	Human	80128	19019	TRIM46	14	14.4	high	Yes
65	170822	2159711	Usp33	Human	23032	20059	USP33	13	13.39	high	Yes
66	76608	1923858	Hectd3	Human	79654	26117	HECTD3	13	13.33	high	Yes
67	19268	102695	Ptprf	Human	5792	9670	PTPRF	15	15.3	high	Yes
68	68180	1915430	Hyi	Human	81888	26948	HYI	11	11.37	high	Yes
69	59002	1891749	Wrap73	Human	49856	12759	WRAP73	14	14.29	high	Yes
70	12537	88353	Cdk11b	Human	984	1729	CDK11B	13	13.39	high	Yes
71	68796	1916046	Tmem214	Human	54867	25983	TMEM214	13	13.26	high	Yes
72	15932	96418	Idua	Human	3425	5391	IDUA	15	15.24	high	Yes
73	24100	1345190	Tpra1	Human	131601	30413	TPRA1	13	13.26	high	Yes
74	232875	3643810	Zscan18	Human	65982	21037	ZSCAN18	8	8.42	high	Yes
75	330485	3607779	Tmem145	Human	284339	26912	TMEM145	13	13.29	high	Yes
76	243961	3613677	Shank1	Human	50944	15474	SHANK1	13	13.39	high	Yes
77	381983	3039582	Lmtk3	Human	114783	19295	LMTK3	16	16.25	high	Yes
78	320878	2444947	Mical2	Human	9645	24693	MICAL2	16	16.25	high	Yes
79	17188	1338823	Maz	Human	4150	6914	MAZ	13	13.24	high	Yes
80	330671	2652891	B4galnt4	Human	338707	26315	B4GALNT4	15	15.3	high	Yes
81	234214	1924574	Sorbs2	Human	8470	24098	SORBS2	12	12.38	high	Yes
82	76900	1924150	Ssbp4	Human	170463	15676	SSBP4	15	15.3	high	Yes
83	70796	1918046	Zdhhc1	Human	29800	17916	ZDHHC1	15	15.24	high	Yes
84	78658	2142989	Ncapd3	Human	23310	28952	NCAPD3	13	13.26	high	Yes
85	73744	1920994	Man2c1	Human	4123	6827	MAN2C1	14	14.31	high	Yes
86	71742	1918992	Ulk3	Human	25989	19703	ULK3	16	16.25	high	Yes
87	109785	97566	Pgm3	Human	5238	8907	PGM3	15	15.24	high	Yes
88	56032	1914482	Nprl2	Human	10641	24969	NPRL2	15	15.3	high	Yes
89	15185	1333752	Hdac6	Human	10013	14064	HDAC6	16	16.25	high	Yes
90	72554	1919804	Utp14a	Human	10813	10665	UTP14A	15	15.24	high	Yes
91	56191	1928994	Tro	Human	7216	12326	TRO	14	14.29	high	Yes

Table S4: List of differential IR genes from Hippocampus that overlapped with curated AD genes							
**RNF112**	**ECE2**	**GRIA2**	**ACTA1**	**GRIA3**	**DGAT1**	**IDUA**	**MAZ**
**ELAC2**	**TFRC**	**RRNAD1**	**HDAC6**	**GRIA1**	**KHSRP**	**SHANK1**	DNM2

16 genes

Table S4: List of differential IR genes from PFC (10w:22m) that overlapped with curated AD genes							
**NOTCH4**	**FANCG**	**IL18BP**	**IL18**	**SFPQ**	**HSF1**	**RAF1**	**FAAH**
**MMEL1**	**PER2**	**CLK1**	**MCOLN1**	**SRSF3**	**OGT**	**SRA1**	**BACE1**
**PLXNA3**	**PI4KB**	**DXO**	**SLC25A27**	**DDIT3**	**CDC25B**	**DDX39A**	**HMOX2**
**CCDC167**	**HSPA5**	JAG2	CIZ1	ABCA7	SYVN1		

30 genes

Note: **41 genes with increased IR in old brain tissues.**

Table S4: Differential IR between young (3 months) and old (24 or 29 months) mouse hippocampus (Stilling et al, 2014)

S/N	Gene Symbol	Ensembl ID	Position of intron	pvalue	IR ratio Older	IR ratio Younger	Timepoint
1	Sf3b1	ENSMUSG00000025982	1: 55014562- 55016375:-	0.0249357	0.237500034	0.110619515	3Mvs24M
2	Fastkd2	ENSMUSG00000025962	1: 63735571- 63735844:+	0.0267099	0.166666838	0.02420269	3Mvs24M
3	Ipo9	ENSMUSG00000041879	1:135405972-135406516:-	0.0255961	0.123188449	0.040462477	3Mvs24M
4	Ufc1	ENSMUSG00000062963	1:171294827-171294920:-	0.0277119	0.026385263	0.137931127	3Mvs24M
5	Ncln	ENSMUSG00000020238	10: 81488355- 81488427:-	0.0324384	0.148148335	0.021897673	3Mvs24M
6	Hnrnph1	ENSMUSG00000007850	11: 50379964- 50381458:+	0.0010159	0.105468775	0.027700857	3Mvs24M
7	Rnf112	ENSMUSG00000010086	11: 61453496- 61453618:-	0.0379092	0.134831518	0.050925967	3Mvs24M
8	Elac2	ENSMUSG00000020549	11: 64998379- 64998507:+	0.0126291	0.190476393	0.020437829	3Mvs24M
9	Gps2	ENSMUSG00000023170	11: 69914861- 69914932:+	0.0303227	0.156862853	0.027659923	3Mvs24M
10	Strada	ENSMUSG00000069631	11:106171077-106171155:-	0.0363153	0.121212286	0.019160464	3Mvs24M
11	Recql5	ENSMUSG00000020752	11:115893719-115893946:-	0.0283485	0.300000261	0.034300858	3Mvs24M
12	Hbp1	ENSMUSG00000002996	12: 31937281- 31937617:-	0.043932	0.239130529	0.05660391	3Mvs24M
13	Ece2	ENSMUSG00000022842	16: 20643733- 20643852:+	0.0335012	0.142857327	0.021388425	3Mvs24M
14	Tfrc	ENSMUSG00000022797	16: 32617199- 32618217:+	0.0131239	0.163636446	0.032786954	3Mvs24M
15	Traf7	ENSMUSG00000052752	17: 24510075- 24510248:-	0.0360271	0.023254811	0.116883214	3Mvs24M
16	Wdr24	ENSMUSG00000025737	17: 25827356- 25827427:+	0.0297336	0.393939486	0.100000144	3Mvs24M
17	Rpp21	ENSMUSG00000024446	17: 36257524- 36257605:-	0.0355231	0.024202679	0.125000108	3Mvs24M
18	4930503L19Rik	ENSMUSG00000044906	18: 70467485- 70467758:-	0.0466842	0.777777728	0.113712949	3Mvs24M
19	Brms1	ENSMUSG00000080268	19: 5046729- 5046814:+	0.0493787	0.024856807	0.122807149	3Mvs24M
20	Rbm39	ENSMUSG00000027620	2:156177385-156179176:-	0.0351591	0.194594627	0.094861701	3Mvs24M
21	Exosc8	ENSMUSG00000027752	3: 54732026- 54732111:-	0.0473622	0.150943499	0.031489752	3Mvs24M
22	Gria2	ENSMUSG00000033981	3: 80707258- 80707698:-	0.0192212	0.116696608	0.059639413	3Mvs24M
23	Rrnad1	ENSMUSG00000004896	3: 87926874- 87927054:-	0.0256669	0.333333534	0.044259159	3Mvs24M
24	Pnisr	ENSMUSG00000028248	4: 21869615- 21870351:+	0.0394247	0.400000039	0.196721382	3Mvs24M
25	Exoc1	ENSMUSG00000036435	5: 76560406- 76561291:+	0.0248665	0.113636486	0.017818628	3Mvs24M
26	Ddx51	ENSMUSG00000029504	5:110655163-110655306:+	0.019128	0.272727556	0.027050068	3Mvs24M
27	Prr14	ENSMUSG00000030822	7:127476293-127476383:+	0.0451641	0.024070767	0.112676153	3Mvs24M
28	Camsap3	ENSMUSG00000044433	8: 3603501- 3603605:+	0.0318003	0.115384816	0.015856938	3Mvs24M
29	Cc2d1a	ENSMUSG00000036686	8: 84134987- 84135061:-	0.0272877	0.109091012	0.018541004	3Mvs24M
30	Acta1	ENSMUSG00000031972	8:123892735-123892832:-	0.0445125	0.178571619	0.029667813	3Mvs24M
31	Dnm2	ENSMUSG00000033335	9: 21505715- 21506332:+	0.0354845	0.016721858	0.105263328	3Mvs24M

S/N	Gene Symbol	Ensembl ID	Position of intron	pvalue	IR ratio Older	IR ratio Younger	Timepoint
32	Rexo2	ENSMUSG00000032026	9: 48473140- 48474401:-	0.0428861	0.221374082	0.107954591	3Mvs24M
33	Gria3	ENSMUSG00000001986	X: 41654253- 41654809:+	0.0285232	0.28571437	0.070175564	3Mvs24M
34	Ilvbl	ENSMUSG00000032763	10: 78583983- 78584069:+	0.039439	0.181818271	0.032787022	3Mvs.29M
35	Dtx3	ENSMUSG00000040415	10:127193338-127193729:-	0.0441704	0.260000091	0.074626984	3Mvs.29M
36	Gls2	ENSMUSG00000044005	10:128199758-128200172:+	0.0418904	0.125000234	0.013138648	3Mvs.29M
37	Esyt1	ENSMUSG00000025366	10:128511786-128511868:-	0.0168914	0.020437879	0.250000307	3Mvs.29M
38	Gria1	ENSMUSG00000020524	11: 57310722- 57317665:+	0.0481342	0.134770909	0.076543236	3Mvs.29M
39	Rnf112	ENSMUSG00000010086	11: 61450705- 61450827:-	0.0267848	0.24584105	0.153078231	3Mvs.29M
40	Rnf112	ENSMUSG00000010086	11: 61450902- 61450977:-	0.0010192	0.318333347	0.163027685	3Mvs.29M
41	Gpld1	ENSMUSG00000021340	13: 24986918- 24987008:+	0.0430063	0.152173983	0.029411888	3Mvs.29M
42	Rab24	ENSMUSG00000034789	13: 55320553- 55320744:-	0.0445593	0.182509529	0.101639377	3Mvs.29M
43	Pdlim7	ENSMUSG00000021493	13: 55507123- 55507329:-	0.0439568	0.29310348	0.152439081	3Mvs.29M
44	Phf20l1	ENSMUSG00000072501	15: 66597447- 66597676:+	0.0350712	0.500000198	0.053740143	3Mvs.29M
45	Naprt	ENSMUSG00000022574	15: 75892047- 75892341:-	0.0130326	0.011996152	0.151515366	3Mvs.29M
46	Eef1d	ENSMUSG00000055762	15: 75896919- 75897225:-	0.0330538	0.140000119	0.017619736	3Mvs.29M
47	Dgat1	ENSMUSG00000022555	15: 76503198- 76503273:-	0.0390963	0.15555568	0.021074579	3Mvs.29M
48	Dgat1	ENSMUSG00000022555	15: 76503315- 76503389:-	0.022355	0.211538572	0.024427349	3Mvs.29M
49	Adck5	ENSMUSG00000022550	15: 76594284- 76594363:+	0.0373724	0.393939553	0.051181069	3Mvs.29M
50	Adck5	ENSMUSG00000022550	15: 76595266- 76595336:+	0.0275873	0.125000186	0.012262738	3Mvs.29M
51	Cpsf1	ENSMUSG00000034022	15: 76601027- 76601105:-	0.0156293	0.109756174	0.011682654	3Mvs.29M
52	Chkb	ENSMUSG00000022617	15: 89427427- 89427560:-	0.0051706	0.102941268	0.007260833	3Mvs.29M
53	Nell2	ENSMUSG00000022454	15: 95527886- 95528475:-	0.0482404	0.368589767	0.221052683	3Mvs.29M
54	Aifm3	ENSMUSG00000022763	16: 17500534- 17500746:+	0.0492623	0.112069021	0.032786956	3Mvs.29M
55	Capn15	ENSMUSG00000037326	17: 25962894- 25962961:-	0.004812	0.127272835	0.008489588	3Mvs.29M
56	Zfp523	ENSMUSG00000024220	17: 28201440- 28202114:+	0.0452972	0.298387132	0.150684989	3Mvs.29M
57	Khsrp	ENSMUSG00000007670	17: 57023593- 57023826:-	0.0104204	0.102272799	0.009596468	3Mvs.29M
58	Kdm3b	ENSMUSG00000038773	18: 34828585- 34828938:+	0.0052939	0.101695018	0.006897792	3Mvs.29M
59	Mus81	ENSMUSG00000024906	19: 5483558- 5483634:-	0.0329039	0.164383648	0.022868155	3Mvs.29M
60	Sipa1	ENSMUSG00000056917	19: 5652566- 5652654:-	0.0376516	0.011496313	0.100000188	3Mvs.29M
61	Rps6ka4	ENSMUSG00000024952	19: 6840405- 6840492:-	0.0089539	0.28358216	0.055555664	3Mvs.29M
62	Csf2ra	ENSMUSG00000059326	19: 61225214- 61225323:-	0.0376799	0.021074576	0.17241401	3Mvs.29M
63	Arhgap21	ENSMUSG00000036591	2: 20855395- 20855580:-	0.0485084	0.130612271	0.064102602	3Mvs.29M

S/N	Gene Symbol	Ensembl ID	Position of intron	pvalue	IR ratio Older	IR ratio Younger	Timepoint
64	Slc39a13	ENSMUSG00000002105	2: 91065700- 91066012:-	0.0230237	0.15789489	0.016230085	3Mvs.29M
65	Lpcat4	ENSMUSG00000027134	2:112241593-112241924:+	0.0372186	0.104761964	0.02797209	3Mvs.29M
66	Tomm34	ENSMUSG00000018322	2:164066669-164070466:-	0.0181865	0.100000128	0.009352937	3Mvs.29M
67	Phc3	ENSMUSG00000037652	3: 30916717- 30922196:-	0.0415333	0.111111325	0.011996157	3Mvs.29M
68	Gria2	ENSMUSG00000033981	3: 80689351- 80691319:-	0.0491888	0.128323712	0.082251102	3Mvs.29M
69	Gria2	ENSMUSG00000033981	3: 80690518- 80692284:-	0.0216942	0.295194524	0.178137684	3Mvs.29M
70	Gria2	ENSMUSG00000033981	3: 80707258- 80707698:-	0.0193985	0.116831693	0.072992715	3Mvs.29M
71	Trim46	ENSMUSG00000042766	3: 89236600- 89237616:-	0.0467425	0.288288331	0.13445385	3Mvs.29M
72	Usp33	ENSMUSG00000025437	3:152374789-152374890:+	0.026463	0.112903276	0.033898356	3Mvs.29M
73	Hectd3	ENSMUSG00000046861	4:117002322-117002509:+	0.049759	0.127659638	0.032608786	3Mvs.29M
74	Ptprf	ENSMUSG00000033295	4:118226377-118226453:-	0.0428318	0.016230082	0.137931276	3Mvs.29M
75	Hyi	ENSMUSG00000006395	4:118362260-118362362:+	0.0484404	0.138889058	0.018394094	3Mvs.29M
76	Wrap73	ENSMUSG00000029029	4:154152424-154152560:+	0.0475031	0.023255912	0.120000099	3Mvs.29M
77	Cdk11b	ENSMUSG00000029062	4:155624941-155625530:+	0.0204975	0.010218945	0.104166825	3Mvs.29M
78	Tmem214	ENSMUSG00000038828	5: 30871570- 30871720:+	0.0418275	0.139240577	0.037735929	3Mvs.29M
79	Idua	ENSMUSG00000033540	5:108683153-108683266:+	0.0357995	0.193548579	0.022072912	3Mvs.29M
80	Tpra1	ENSMUSG00000002871	6: 88910401- 88910547:+	0.0166821	0.195402371	0.028169135	3Mvs.29M
81	Zscan18	ENSMUSG00000070822	7: 12772115- 12773440:-	0.0231426	0.114285882	0.010820063	3Mvs.29M
82	Tmem145	ENSMUSG00000043843	7: 25308841- 25308978:+	0.0316912	0.214285845	0.026870082	3Mvs.29M
83	Shank1	ENSMUSG00000038738	7: 44343738- 44344297:+	0.0216206	0.146341597	0.015138087	3Mvs.29M
84	Lmtk3	ENSMUSG00000062044	7: 45786584- 45786809:+	0.0270336	0.126182987	0.059649154	3Mvs.29M
85	Mical2	ENSMUSG00000038244	7:112335156-112335328:+	0.0017135	0.149350665	0.066543458	3Mvs.29M
86	Maz	ENSMUSG00000030678	7:127023168-127023481:-	0.0155436	0.208333539	0.016721909	3Mvs.29M
87	B4galnt4	ENSMUSG00000055629	7:141064555-141064640:+	0.0357148	0.113095277	0.038216615	3Mvs.29M
88	Sorbs2	ENSMUSG00000031626	8: 45736202- 45741486:+	0.0488724	0.103448478	0.012262736	3Mvs.29M
89	Ssbp4	ENSMUSG00000070003	8: 70597768- 70598012:-	0.031874	0.190635473	0.103896137	3Mvs.29M
90	Zdhhc1	ENSMUSG00000039199	8:105476529-105476613:-	0.0379135	0.117647136	0.02083343	3Mvs.29M
91	Ncapd3	ENSMUSG00000035024	9: 27052665- 27055471:+	0.0483277	0.136363882	0.015328419	3Mvs.29M
92	Man2c1	ENSMUSG00000032295	9: 57138969- 57139068:+	0.0206811	0.104166795	0.010411757	3Mvs.29M
93	Ulk3	ENSMUSG00000032308	9: 57594212- 57594283:+	0.026052	0.014521658	0.16000028	3Mvs.29M
94	Pgm3	ENSMUSG00000056131	9: 86558508- 86559414:-	0.0278058	0.121212296	0.012262736	3Mvs.29M
95	Nprl2	ENSMUSG00000010057	9:107544634-107544746:+	0.0189579	0.143884936	0.042857202	3Mvs.29M

S/N	Gene Symbol	Ensembl ID	Position of intron	pvalue	IR ratio Older	IR ratio Younger	Timepoint
96	Hdac6	ENSMUSG00000031161	X: 7939532- 7939605:-	0.0368865	0.113207659	0.014927833	3Mvs.29M
97	Utp14a	ENSMUSG00000063785	X: 48272919- 48273021:+	0.0474654	0.222222456	0.026214726	3Mvs.29M
98	Tro	ENSMUSG00000025272	X:150651632-150652283:-	0.0451677	0.150684972	0.063953538	3Mvs.29M
