# Supplementary material for: Increased intron retention is a post‐transcriptional signature associated with progressive aging and Alzheimer’s disease
Source: Aging Cell. 2019 Mar 13;18(3):e12928. doi: 10.1111/acel.12928 (PMC6516162; doi:10.1111/acel.12928)
Supplement: Supplementary file 6 [file ACEL-18-e12928-s006.pdf]

Table S5: List of conserved fly and mouse genes that underwent age-dependent IR

S/N	Fly Gene	Total # Introns	# of IR	MouseSymbol	Total # Introns	# of IR
1	Actn	20	1	Actn1	22	2
2	Arp1	5	1	Actr1b	14	2
3	CG10089	9	1	Ssh3	14	1
4	CG10249	18	1	Kank3	12	3
5	CG10962	1	1	Dhrs11	6	1
6	CG11147	11	1	Abca8b	39	1
7	CG2064	4	1	Dhrs13	5	1
8	CG30427	16	1	Far1	17	1
9	CG30491	5	1	Dhrs13	5	1
10	CG31321	7	1	Slc46a1	4	2
11	CG34417	35	2	Smtn	33	1
12	CG4210	3	2	Sat2	6	1
13	CG42708	13	1	Gls2	22	1
14	CG43897	25	1	Pdlim7	17	5
15	CG8388	3	1	Zfp335	27	4
16	chp	16	1	Scrib	41	1
17	d4	7	2	Dpf2	14	1
18	Dip-B	5	2	Npepl1	11	1
19	Doa	30	1	Clk1	12	1
20	gol	8	2	Rnf215	10	1
21	HDAC4	22	1	Hdac6	30	1
22	hk	20	1	Hook2	23	1
23	Hpr1	4	1	Thoc1	20	1
24	Ilk	3	1	Ilk	13	6
25	Itp-r83A	21	2	Itpr3	57	2
26	l(2)k01209	8	2	Uckl1	20	1
27	Lar	30	2	Ptprf	38	1
28	lbm	5	1	Tspan4	11	2
29	Mct1	9	4	Slc16a11	11	1
30	mdy	12	1	Dgat1	17	1
31	Mhc	46	8	Myh7b	41	4
32	Nep3	10	1	Ece2	24	3
33	per	8	1	Per2	23	2
34	Pfk	17	1	Pfkm	23	1
35	Pitslre	6	2	Cdk11b	24	1
36	Prm	11	1	Myh7b	41	4
37	RhoGEF2	27	1	Arhgef1	43	2
38	RyR	39	1	Ryr3	110	3
39	sls	52	5	Speg	52	1
40	trc	5	1	Stk38	20	1
41	Tsp42En	4	1	Tspan4	11	2
42	Tsp96F	4	1	Tspan4	11	2
43	Unc-89	42	8	Speg	52	1
44	up	21	1	Tnnt1	22	1
45	Zasp66	15	6	Pdlim7	17	5

Total # Introns: Total number of introns in the gene

# of IR: Number of introns that are differentially retained in aging.
