# Supplementary material for: Increased intron retention is a post‐transcriptional signature associated with progressive aging and Alzheimer’s disease
Source: Aging Cell. 2019 Mar 13;18(3):e12928. doi: 10.1111/acel.12928 (PMC6516162; doi:10.1111/acel.12928)
Supplement: Supplementary file 7 [file ACEL-18-e12928-s007.pdf]

**Table S6: DAVID functional annotation chart of differential IR genes between Young & Old human prefrontal cortex**

| GOTERM_BP_5 | Count | % | *P-Value* | Fold Enrichment | Benjamini value |
|---|---|---|---|---|---|
| GO:0030163~protein catabolic process | 55 | 9.734513 | 2.76E-07 | 2.111769159 | 7.93E-04 |
| GO:0044257~cellular protein catabolic process | 49 | 8.672566 | 3.63E-07 | 2.214529479 | 5.23E-04 |
| GO:0010498~proteasomal protein catabolic process | 34 | 6.017699 | 8.37E-07 | 2.627111337 | 8.02E-04 |
| GO:0043632~modification-dependent macromolecule catabolic process | 43 | 7.610619 | 1.31E-06 | 2.257971052 | 9.45E-04 |
| GO:0051603~proteolysis involved in cellular protein catabolic process | 46 | 8.141593 | 1.35E-06 | 2.180203687 | 7.77E-04 |
| GO:0044267~cellular protein metabolic process | 207 | 36.63717 | 2.07E-06 | 1.315463714 | 9.92E-04 |
| GO:0044265~cellular macromolecule catabolic process | 58 | 10.26549 | 8.00E-06 | 1.850473131 | 0.003284399 |
| GO:0006886~intracellular protein transport | 59 | 10.44248 | 1.46E-05 | 1.801177228 | 0.005255264 |
| GO:0006464~cellular protein modification process | 164 | 29.02655 | 2.12E-05 | 1.337557718 | 0.006747364 |
| GO:0036211~protein modification process | 164 | 29.02655 | 2.12E-05 | 1.337557718 | 0.006747364 |
| GO:0042176~regulation of protein catabolic process | 29 | 5.132743 | 2.46E-05 | 2.440624022 | 0.007050565 |
| GO:0036503~ER associated protein degradation (ERAD) pathway | 13 | 2.300885 | 3.65E-05 | 4.400075541 | 0.009497119 |
| GO:1903362~regulation of cellular protein catabolic process | 22 | 3.893805 | 6.13E-05 | 2.729314403 | 0.014594634 |
| GO:0048666~neuron development | 54 | 9.557522 | 8.65E-05 | 1.742493048 | 0.018959648 |
| GO:0015031~protein transport | 88 | 15.57522 | 1.73E-04 | 1.473242828 | 0.034915746 |
| GO:0007399~nervous system development | 99 | 17.52212 | 2.23E-04 | 1.420627013 | 0.041803736 |
| GO:0043087~regulation of GTPase activity | 41 | 7.256637 | 2.27E-04 | 1.84761048 | 0.04003216 |
| GO:0031175~neuron projection development | 46 | 8.141593 | 2.80E-04 | 1.753236013 | 0.046345931 |

| Table S6: List of differential IR genes that overlapped with curated AD genes | | | |
|---|---|---|---|
| ***ABCA2*** | ***DLG3*** | ***MOG*** | ***STIP1*** |
| ***ABCG4*** | ***DNMT1*** | ***MTG1*** | ***STUB1*** |
| ***ADAM10*** | ***EIF4A1*** | ***NAE1*** | ***SV2A*** |
| ***AGRN*** | ***ETS2*** | ***NPEPPS*** | ***SYVN1*** |
| ***ALS2*** | ***FAAH*** | ***OGT*** | ***TAOK2*** |
| ***APBB3*** | ***FLOT1*** | ***OLR1*** | ***TF*** |
| ***APEH*** | ***GABBR1*** | ***OXA1L*** | ***TNFAIP1*** |
| ***APEX1*** | ***GRIA2*** | ***PFKFB3*** | ***TRPC4AP*** |
| ***ARRB2*** | ***GRK2*** | ***PIK3CD*** | ***TSC2*** |
| ***ARSA*** | ***HARS*** | ***PITRM1*** | ***TTBK1*** |
| ***ATN1*** | ***HGS*** | ***PLK2*** | ***XBP1*** |
| ***BIN1*** | ***HNRNPA1*** | ***POLDIP2*** | ***XPNPEP1*** |
| ***BRD2*** | ***HNRNPA2B1*** | ***PRRT2*** | ACADVL |
| ***BRSK1*** | ***HSPD1*** | ***PTGDS*** | CCL2 |
| ***C1R*** | ***KAT5*** | ***PTPN5*** | CFB |
| ***CDK18*** | ***L1CAM*** | ***PTPRA*** | CPT1B |
| ***CDK5R1*** | ***MAPK8IP1*** | ***RELA*** | CYP27A1 |
| ***CIB1*** | ***MARK2*** | ***RYR3*** | EPOR |
| ***CTTN*** | ***MCOLN1*** | ***SEL1L*** | FES |
| ***DAPK1*** | ***MED23*** | ***SMAD3*** | LRP2 |
| ***DGAT1*** | ***MLX*** | ***SMPD1*** | NR1H3 |

Note: *75 Genes with increased IR in old prefrontal cortex*

Table S6: Differential IR between young (16-20 yrs) & old (70-80 yrs) human prefrontal cortex

| S/N | Chr | Start | End | Gene | IR ratio-Young | IR ratio-Old | *p*- value |
|---|---|---|---|---|---|---|---|
| 1 | 1 | 1042162 | 1043238 | AGRN | 0.103469 | 0.350257 | 0.00980044 |
| 2 | 1 | 1045876 | 1045963 | AGRN | 0.0227273 | 0.162869 | 0.0218293 |
| 3 | 1 | 1046088 | 1046159 | AGRN | 0.109015 | 0.262936 | 0.0271723 |
| 4 | 1 | 109261264 | 109261512 | CELSR2 | 0.0117384 | 0.113846 | 0.0354233 |
| 5 | 1 | 109627316 | 109627428 | AMPD2 | 0.0396341 | 0.227586 | 0.00340395 |
| 6 | 1 | 109628806 | 109629108 | AMPD2 | 0.0442933 | 0.148883 | 0.0352942 |
| 7 | 1 | 109715042 | 109715129 | GSTM5 | 0.166667 | 0.0110717 | 0.0239891 |
| 8 | 1 | 110019626 | 110020730 | AHCYL1 | 0.0406795 | 0.100795 | 0.0485468 |
| 9 | 1 | 110047869 | 110049111 | STRIP1 | 0.0575922 | 0.238208 | 0.0295332 |
| 10 | 1 | 110049238 | 110049459 | STRIP1 | 0.0291453 | 0.177809 | 0.00574505 |
| 11 | 1 | 111477775 | 111477982 | C1orf162 | 0.822072 | 0.333829 | 0.0462912 |
| 12 | 1 | 111777273 | 111780224 | KCND3 | 0.0313666 | 0.281549 | 0.0443213 |
| 13 | 1 | 1255487 | 1256044 | UBE2J2 | 0.131599 | 0.486618 | 0.0206896 |
| 14 | 1 | 1298679 | 1299344 | ACAP3 | 0.145403 | 0.267091 | 0.0247057 |
| 15 | 1 | 1299356 | 1299830 | ACAP3 | 0.223546 | 0.383698 | 0.0290321 |
| 16 | 1 | 1300068 | 1300157 | ACAP3 | 0.0577547 | 0.147601 | 0.0112225 |
| 17 | 1 | 1309851 | 1310633 | PUSL1 | 0.0263282 | 0.220126 | 0.037037 |
| 18 | 1 | 1311924 | 1312017 | CPSF3L | 0.184994 | 0.0196078 | 0.0111699 |
| 19 | 1 | 1312368 | 1312439 | CPSF3L | 0.0551583 | 0.21018 | 0.0206662 |
| 20 | 1 | 1315438 | 1315519 | CPSF3L | 0.313609 | 0.628523 | 0.0347054 |
| 21 | 1 | 145836574 | 145836814 | POLR3C | 0.0135811 | 0.124567 | 0.0433735 |
| 22 | 1 | 149907833 | 149908041 | SV2A | 0.0802056 | 0.208041 | 0.00231907 |
| 23 | 1 | 151139701 | 151139975 | SEMA6C | 0.0178187 | 0.198074 | 0.0323934 |
| 24 | 1 | 151400588 | 151400763 | PSMB4 | 0.101645 | 0.261223 | 6.33E-07 |
| 25 | 1 | 151400845 | 151401238 | PSMB4 | 0.0780136 | 0.139568 | 0.00340709 |
| 26 | 1 | 151401355 | 151401541 | PSMB4 | 0.0543869 | 0.126004 | 0.00290245 |
| 27 | 1 | 153778355 | 153778462 | SLC27A3 | 1 | 0.270703 | 0.0104698 |
| 28 | 1 | 153932777 | 153932860 | DENND4B | 0.0452163 | 0.162162 | 0.0352313 |
| 29 | 1 | 154585344 | 154585752 | ADAR | 0.106019 | 0.205495 | 0.0398742 |
| 30 | 1 | 154588258 | 154588550 | ADAR | 0.0994075 | 0.279472 | 0.0216584 |
| 31 | 1 | 154948005 | 154948132 | PBXIP1 | 0.374525 | 0.0389484 | 0.004672 |
| 32 | 1 | 155197213 | 155197462 | THBS3 | 0.173765 | 0.682488 | 0.00861217 |
| 33 | 1 | 155202401 | 155202811 | THBS3 | 0.141602 | 0.609146 | 0.0390067 |
| 34 | 1 | 156743455 | 156743651 | HDGF | 0.0372716 | 0.164405 | 0.014462 |
| 35 | 1 | 15943519 | 15943598 | ZBTB17 | 0.0159396 | 0.246238 | 0.0264749 |
| 36 | 1 | 160084880 | 160087485 | KCNJ9 | 0.0979441 | 0.257011 | 0.0158451 |
| 37 | 1 | 160092695 | 160092923 | IGSF8 | 0.399262 | 0.524194 | 0.00773123 |
| 38 | 1 | 160093331 | 160093709 | IGSF8 | 0.102999 | 0.201268 | 0.0065214 |
| 39 | 1 | 160332557 | 160333602 | COPA | 0.0413205 | 0.154294 | 0.0455789 |
| 40 | 1 | 161213921 | 161214155 | NDUFS2 | 0.109081 | 0.448156 | 5.17E-16 |
| 41 | 1 | 161228308 | 161228427 | TOMM40L | 0.0713672 | 0.318182 | 0.0359365 |
| 42 | 1 | 1669736 | 1670097 | SLC35E2B | 0.264047 | 0.817924 | 0.00433631 |
| 43 | 1 | 1670151 | 1671508 | SLC35E2B | 0.055903 | 0.258244 | 0.0195313 |
| 44 | 1 | 16993835 | 16995975 | ATP13A2 | 0.055705 | 0.151086 | 0.0299411 |
| 45 | 1 | 19243707 | 19243949 | EMC1 | 0.0159067 | 0.27553 | 0.0158535 |
| 46 | 1 | 202909124 | 202911053 | KLHL12 | 0.0154889 | 0.12658 | 0.0470893 |
| 47 | 1 | 203048714 | 203048917 | PPFIA4 | 0.0670761 | 0.292839 | 0.0493233 |
| 48 | 1 | 204222779 | 204223461 | PLEKHA6 | 0.0558065 | 0.232955 | 0.0431982 |

| S/N | Chr | Start | End | Gene | IR ratio-Young | IR ratio-Old | *p*- value |
|---|---|---|---|---|---|---|---|
| 49 | 1 | 205526837 | 205527793 | CDK18 | 0.285232 | 0.467489 | 0.0384412 |
| 50 | 1 | 205798815 | 205798956 | SLC41A1 | 0.0786424 | 0.311092 | 0.0472987 |
| 51 | 1 | 206585295 | 206586825 | RASSF5 | 0.0336936 | 0.280899 | 0.0406498 |
| 52 | 1 | 220210489 | 220210800 | RAB3GAP2 | 0.00800377 | 0.218515 | 0.0480841 |
| 53 | 1 | 225842474 | 225844497 | EPHX1 | 0.204173 | 0.367898 | 0.02411 |
| 54 | 1 | 225922383 | 225923700 | PYCR2 | 0.166833 | 0.365821 | 0.023657 |
| 55 | 1 | 228102382 | 228102477 | C1orf35 | 0.178571 | 0.565217 | 0.00672337 |
| 56 | 1 | 229325411 | 229326737 | CCSAP | 0.00744445 | 0.122073 | 0.00872685 |
| 57 | 1 | 2495570 | 2496606 | PLCH2 | 0.00919823 | 0.128158 | 0.0123102 |
| 58 | 1 | 26032485 | 26033228 | EXTL1 | 0.122329 | 0.246932 | 0.0486331 |
| 59 | 1 | 26474667 | 26475112 | HMGN2 | 0.0169492 | 0.107509 | 0.0277198 |
| 60 | 1 | 32160703 | 32161946 | KPNA6 | 0.0114648 | 0.246471 | 0.00411523 |
| 61 | 1 | 32163313 | 32166104 | KPNA6 | 0.0195155 | 0.185468 | 0.041477 |
| 62 | 1 | 36089115 | 36091243 | ADPRHL2 | 0.0408793 | 0.356991 | 0.0314301 |
| 63 | 1 | 37806976 | 37807100 | YRDC | 0.0271084 | 0.199367 | 0.0415973 |
| 64 | 1 | 37865888 | 37866458 | INPP5B | 0.11067 | 0.00663767 | 0.0495712 |
| 65 | 1 | 40413102 | 40414158 | SMAP2 | 0.0550492 | 0.286808 | 0.00201849 |
| 66 | 1 | 41008714 | 41008793 | CTPS1 | 0.047619 | 0.269521 | 0.04096 |
| 67 | 1 | 42747412 | 42747722 | P3H1 | 0.631902 | 0.162968 | 0.048769 |
| 68 | 1 | 43617911 | 43618629 | PTPRF | 0.0409926 | 0.200724 | 0.022671 |
| 69 | 1 | 44649957 | 44650703 | RNF220 | 0.027153 | 0.21673 | 1.24E-08 |
| 70 | 1 | 45008701 | 45009143 | HECTD3 | 0.0122279 | 0.2327 | 0.0138658 |
| 71 | 1 | 46193388 | 46193563 | POMGNT1 | 0.193223 | 0.575245 | 4.06E-06 |
| 72 | 1 | 46410497 | 46410813 | FAAH | 0.139518 | 0.316277 | 0.0317964 |
| 73 | 1 | 53218495 | 53218866 | C1orf123 | 0.302376 | 0.497641 | 0.0450563 |
| 74 | 1 | 6588442 | 6588755 | ZBTB48 | 0.0465702 | 0.339724 | 0.0394336 |
| 75 | 1 | 8014396 | 8015307 | ERRFI1 | 0.132853 | 0.484378 | 0.0413018 |
| 76 | 1 | 9724092 | 9724275 | PIK3CD | 0.0625 | 0.386167 | 0.0443488 |
| 77 | 10 | 101031534 | 101032313 | SFXN3 | 0.0801616 | 0.416541 | 0.030136 |
| 78 | 10 | 109875599 | 109877789 | XPNPEP1 | 0.106383 | 0.446363 | 0.00552924 |
| 79 | 10 | 119576750 | 119577079 | TIAL1 | 0.238137 | 0.665065 | 0.00053094 |
| 80 | 10 | 119823902 | 119826630 | INPP5F | 0.0283699 | 0.114852 | 0.0269716 |
| 81 | 10 | 119933167 | 119933685 | SEC23IP | 0.0431507 | 0.368421 | 0.0305176 |
| 82 | 10 | 121959410 | 121959500 | NSMCE4A | 0.23506 | 0.66879 | 0.0304508 |
| 83 | 10 | 125796188 | 125798064 | UROS | 0.0463324 | 0.142162 | 0.0498199 |
| 84 | 10 | 133401590 | 133402148 | MTG1 | 0.202899 | 0.506075 | 0.0199328 |
| 85 | 10 | 133557156 | 133557863 | SYCE1 | 0.0357143 | 0.258307 | 0.0365798 |
| 86 | 10 | 17603744 | 17603929 | HACD1 | 0.0214352 | 0.419223 | 0.0184237 |
| 87 | 10 | 3147737 | 3147986 | PITRM1 | 0.0498296 | 0.215959 | 0.00796147 |
| 88 | 10 | 6220865 | 6221380 | PFKFB3 | 0.0115658 | 0.158175 | 0.0151575 |
| 89 | 10 | 6226365 | 6228195 | PFKFB3 | 0.0408455 | 0.175899 | 0.0340407 |
| 90 | 10 | 73782441 | 73783073 | CHCHD1 | 0.095125 | 0.273708 | 0.0287628 |
| 91 | 10 | 73789539 | 73789716 | ZSWIM8 | 0.0271857 | 0.148927 | 0.0229286 |
| 92 | 10 | 95693476 | 95693643 | TCTN3 | 0.0141287 | 0.209806 | 0.0474272 |
| 93 | 10 | 97466159 | 97466503 | MMS19 | 0.056338 | 0.152435 | 0.0432819 |
| 94 | 10 | 97753182 | 97757264 | ZFYVE27 | 0.0987424 | 0.30595 | 0.0463323 |
| 95 | 11 | 111720003 | 111720477 | SIK2 | 0.0163703 | 0.2 | 0.0455539 |
| 96 | 11 | 119097063 | 119097141 | DPAGT1 | 0.112903 | 0.245261 | 0.0284504 |
| 97 | 11 | 119154575 | 119154769 | ABCG4 | 0.0286576 | 0.285714 | 0.037037 |

| S/N | Chr | Start | End | Gene | IR ratio-Young | IR ratio-Old | *p*- value |
|---|---|---|---|---|---|---|---|
| 98 | 11 | 119677219 | 119677554 | NECTIN1 | 0.00886431 | 0.127249 | 0.0118588 |
| 99 | 11 | 126261484 | 126262119 | FAM118B | 0.0121871 | 0.143723 | 0.0337359 |
| 100 | 11 | 18729027 | 18729452 | PTPN5 | 0.14047 | 0.248781 | 0.0208762 |
| 101 | 11 | 251626 | 251819 | PSMD13 | 0.0628124 | 0.172707 | 0.0187791 |
| 102 | 11 | 373116 | 373190 | B4GALNT4 | 0.0777202 | 0.210394 | 0.00770858 |
| 103 | 11 | 45870532 | 45870841 | CRY2 | 0.0766797 | 0.216194 | 0.0360484 |
| 104 | 11 | 45900452 | 45901979 | MAPK8IP1 | 0.0401422 | 0.141676 | 0.00788726 |
| 105 | 11 | 46656273 | 46657094 | ATG13 | 0.00819371 | 0.200278 | 0.00231655 |
| 106 | 11 | 47173482 | 47173758 | ARFGAP2 | 0.0693667 | 0.256966 | 0.0231804 |
| 107 | 11 | 47261449 | 47261546 | NR1H3 | 0.608209 | 0.128631 | 0.0308023 |
| 108 | 11 | 488647 | 489399 | PTDSS2 | 0.245635 | 0.506845 | 0.0233232 |
| 109 | 11 | 57646405 | 57646750 | YPEL4 | 0.164318 | 0.324389 | 0.0257765 |
| 110 | 11 | 57704106 | 57704301 | MED19 | 0.047619 | 0.473684 | 0.00738188 |
| 111 | 11 | 57808292 | 57808389 | CTNND1 | 0.0463215 | 0.198207 | 0.0141924 |
| 112 | 11 | 61314210 | 61314307 | DDB1 | 0.0654381 | 0.211066 | 0.0103397 |
| 113 | 11 | 61876455 | 61876865 | FADS3 | 0.35528 | 0.693669 | 0.00042387 |
| 114 | 11 | 6270337 | 6270645 | CCKBR | 0.00751494 | 0.139623 | 0.0169189 |
| 115 | 11 | 63900110 | 63900558 | MARK2 | 0.0682353 | 0.279557 | 0.0285631 |
| 116 | 11 | 6394041 | 6394197 | SMPD1 | 0.173913 | 0.371126 | 0.0336964 |
| 117 | 11 | 64195813 | 64197270 | STIP1 | 0.0208333 | 0.151391 | 0.0208312 |
| 118 | 11 | 64224341 | 64224542 | TRPT1 | 0.0574774 | 0.290789 | 0.0176464 |
| 119 | 11 | 64770408 | 64773429 | SF1 | 0.263081 | 0.47035 | 0.0445102 |
| 120 | 11 | 64911275 | 64911841 | ATG2A | 0.100402 | 0.508772 | 0.0429114 |
| 121 | 11 | 64931489 | 64931558 | PPP2R5B | 0.119872 | 0.256111 | 0.00293459 |
| 122 | 11 | 65132351 | 65132731 | SYVN1 | 0.0757759 | 0.206572 | 0.0137804 |
| 123 | 11 | 65649672 | 65649756 | SIPA1 | 0.333333 | 0.00237906 | 0.0225 |
| 124 | 11 | 65655000 | 65655687 | RELA | 0.0713878 | 0.394714 | 0.00569665 |
| 125 | 11 | 65662088 | 65662178 | RELA | 0.248042 | 0.0416667 | 0.0452675 |
| 126 | 11 | 65716807 | 65716888 | KAT5 | 0.015873 | 0.124461 | 0.0125913 |
| 127 | 11 | 65865145 | 65865219 | MUS81 | 0.106383 | 0.0116861 | 0.0239621 |
| 128 | 11 | 66564146 | 66564557 | CTSF | 0.219957 | 0.338363 | 0.0240724 |
| 129 | 11 | 66845562 | 66845859 | RCE1 | 0.02 | 0.13181 | 0.0215189 |
| 130 | 11 | 67280783 | 67281092 | GRK2 | 0.0282686 | 0.118262 | 0.0112303 |
| 131 | 11 | 67352019 | 67352690 | AP003419.11 | 0.0296836 | 0.135341 | 0.0286471 |
| 132 | 11 | 67420840 | 67420938 | CARNS1 | 0.165701 | 0.0420543 | 0.0114452 |
| 133 | 11 | 67608478 | 67608551 | NDUFV1 | 0.0540795 | 0.12619 | 0.0263759 |
| 134 | 11 | 70407591 | 70409830 | CTTN | 0.22293 | 0.469369 | 0.0221787 |
| 135 | 11 | 72004099 | 72004224 | NUMA1 | 0.115347 | 0.0270701 | 0.0218669 |
| 136 | 11 | 72579608 | 72580576 | PDE2A | 0.0473734 | 0.105793 | 0.0100132 |
| 137 | 11 | 72589240 | 72589750 | PDE2A | 0.0444437 | 0.275428 | 1.33E-05 |
| 138 | 11 | 74251018 | 74251647 | PPME1 | 0.0143916 | 0.111576 | 0.00085667 |
| 139 | 11 | 96387613 | 96389843 | CCDC82 | 0.0200524 | 0.27896 | 0.0174851 |
| 140 | 12 | 10160021 | 10160346 | OLR1 | 0.0277778 | 0.418099 | 0.0150062 |
| 141 | 12 | 119710620 | 119712177 | CIT | 0.260368 | 0.479688 | 0.0382366 |
| 142 | 12 | 120659908 | 120660195 | CABP1 | 0.0972624 | 0.238673 | 1.06E-05 |
| 143 | 12 | 122855903 | 122855979 | HIP1R | 0.0381679 | 0.146493 | 0.0342597 |
| 144 | 12 | 122976767 | 122976870 | OGFOD2 | 0.25 | 0.0151286 | 0.0480841 |
| 145 | 12 | 122986829 | 122987280 | PITPNM2 | 0.107063 | 0.321839 | 0.0424224 |
| 146 | 12 | 131838327 | 131838611 | MMP17 | 0.0108303 | 0.161677 | 0.00849266 |

| S/N | Chr | Start | End | Gene | IR ratio-Young | IR ratio-Old | *p*- value |
|---|---|---|---|---|---|---|---|
| 147 | 12 | 48768716 | 48768936 | ADCY6 | 0.0300231 | 0.192982 | 0.0461635 |
| 148 | 12 | 49003595 | 49003756 | PRKAG1 | 0.0292807 | 0.24255 | 0.00297042 |
| 149 | 12 | 49558970 | 49559213 | MCRS1 | 0.0125375 | 0.135177 | 0.0248424 |
| 150 | 12 | 49559301 | 49559452 | MCRS1 | 0.128946 | 0.335347 | 0.0190753 |
| 151 | 12 | 49801967 | 49802957 | NCKAP5L | 0.175544 | 0.655773 | 0.0294483 |
| 152 | 12 | 50077363 | 50077999 | ASIC1 | 0.0160967 | 0.120338 | 0.0346266 |
| 153 | 12 | 53039343 | 53039629 | EIF4B | 0.157395 | 0.371443 | 0.0194862 |
| 154 | 12 | 53171441 | 53171881 | CSAD | 0.755769 | 0.368683 | 0.042459 |
| 155 | 12 | 53712121 | 53713099 | CALCOCO1 | 0.31182 | 0.509082 | 0.00985679 |
| 156 | 12 | 54284317 | 54284548 | HNRNPA1 | 0.0660529 | 0.14529 | 0.0122497 |
| 157 | 12 | 55938052 | 55938510 | DGKA | 0.0192466 | 0.239079 | 0.0123457 |
| 158 | 12 | 56138272 | 56138403 | ESYT1 | 0.0693257 | 0.262465 | 0.0405476 |
| 159 | 12 | 56157735 | 56157881 | MYL6B | 0.045614 | 0.123193 | 0.0420562 |
| 160 | 12 | 56349059 | 56349162 | STAT2 | 0.0657382 | 0.196208 | 0.0368484 |
| 161 | 12 | 57516337 | 57516434 | MARS | 0.022538 | 0.100488 | 0.0329887 |
| 162 | 12 | 57614400 | 57614515 | ARHGEF25 | 0.0632077 | 0.170721 | 0.00418668 |
| 163 | 12 | 62565380 | 62566013 | MON2 | 0.012052 | 0.195144 | 0.0445645 |
| 164 | 12 | 62566031 | 62566321 | MON2 | 0.00895274 | 0.325809 | 0.0121315 |
| 165 | 12 | 6937561 | 6937844 | ATN1 | 0.0653667 | 0.207143 | 0.0497985 |
| 166 | 12 | 7088986 | 7089292 | C1R | 0.242902 | 0.667999 | 0.041639 |
| 167 | 12 | 8092784 | 8092871 | NECAP1 | 0.0273973 | 0.18495 | 0.00318629 |
| 168 | 12 | 96280885 | 96282508 | CDK17 | 0.0288744 | 0.119857 | 0.0366442 |
| 169 | 13 | 113996739 | 113999584 | RASA3 | 0.0800405 | 0.298538 | 0.0465691 |
| 170 | 13 | 114243989 | 114244889 | CDC16 | 0.142646 | 0.432995 | 0.0498839 |
| 171 | 13 | 24940785 | 24941986 | TPTE2P1 | 0.500151 | 0.0833333 | 0.04096 |
| 172 | 13 | 29517790 | 29519446 | SLC7A1 | 0.0136932 | 0.150215 | 0.0423875 |
| 173 | 13 | 49522107 | 49523174 | PHF11 | 0.0452021 | 0.309689 | 0.0482828 |
| 174 | 13 | 98000645 | 98002466 | IPO5 | 0.0344335 | 0.184732 | 0.0207958 |
| 175 | 13 | 98796213 | 98797114 | DOCK9 | 0.119241 | 0.269812 | 0.0210544 |
| 176 | 14 | 105479219 | 105479435 | CRIP2 | 0.0176296 | 0.16946 | 1.95E-06 |
| 177 | 14 | 20456101 | 20456667 | APEX1 | 0.0610367 | 0.206952 | 0.0385724 |
| 178 | 14 | 22770278 | 22770460 | OXA1L | 0.187473 | 0.396298 | 0.0120998 |
| 179 | 14 | 24074125 | 24074290 | CPNE6 | 0.138568 | 0.25994 | 0.0298114 |
| 180 | 14 | 24318568 | 24318653 | ADCY4 | 0.5 | 0.152518 | 0.0357836 |
| 181 | 14 | 35096633 | 35096708 | PPP2R3C | 0.0176322 | 0.153846 | 0.0380204 |
| 182 | 14 | 49779675 | 49779747 | KLHDC2 | 0.0757199 | 0.339243 | 3.03E-06 |
| 183 | 14 | 49785004 | 49785091 | NEMF | 0.0619597 | 0.4 | 0.00509854 |
| 184 | 14 | 50982965 | 50983379 | TRIM9 | 0.0789434 | 0.23913 | 0.00612416 |
| 185 | 14 | 64749696 | 64749980 | SPTB | 0.138483 | 0.554269 | 0.00714071 |
| 186 | 14 | 64951151 | 64951602 | RAB15 | 0.0880428 | 0.222733 | 0.00791807 |
| 187 | 14 | 67573887 | 67574241 | PLEKHH1 | 0.00454733 | 0.116396 | 0.0450632 |
| 188 | 14 | 69703592 | 69704603 | SUSD6 | 0.143946 | 0.741207 | 0.00828126 |
| 189 | 14 | 72954262 | 72954385 | DCAF4 | 0.0150555 | 0.4125 | 0.0459184 |
| 190 | 14 | 73111155 | 73111527 | RBM25 | 0.196459 | 0.432323 | 0.00651935 |
| 191 | 14 | 74290499 | 74292286 | ABCD4 | 0.256237 | 0.608162 | 0.0167131 |
| 192 | 14 | 75003153 | 75003274 | EIF2B2 | 0.0273823 | 0.364706 | 0.0167962 |
| 193 | 14 | 77435393 | 77435843 | VIPAS39 | 0.0977918 | 0.371802 | 0.0491881 |
| 194 | 14 | 81487942 | 81489251 | SEL1L | 0.00816652 | 0.121282 | 0.0470893 |
| 195 | 14 | 92704361 | 92704639 | LGMN | 0.0212072 | 0.149925 | 0.0168823 |

| S/N | Chr | Start | End | Gene | IR ratio-Young | IR ratio-Old | *p*- value |
|---|---|---|---|---|---|---|---|
| 196 | 14 | 92714451 | 92716135 | LGMN | 0.0310131 | 0.199802 | 0.0453427 |
| 197 | 14 | 94128675 | 94129261 | IFI27L2 | 0.047619 | 0.213393 | 0.0498229 |
| 198 | 15 | 25409207 | 25411907 | UBE3A | 0.0433799 | 0.502436 | 0.0382658 |
| 199 | 15 | 33826752 | 33827198 | RYR3 | 0.00456621 | 0.317035 | 0.0368729 |
| 200 | 15 | 34359745 | 34360110 | LPCAT4 | 0.224376 | 0.453735 | 0.00016834 |
| 201 | 15 | 40290672 | 40290760 | PLCB2 | 0.62704 | 0.235912 | 0.0276981 |
| 202 | 15 | 41842276 | 41842553 | JMJD7-PLA2G | 0.186787 | 0.439487 | 0.0281639 |
| 203 | 15 | 42547529 | 42548105 | LRRC57 | 0.0197248 | 0.25 | 0.0330579 |
| 204 | 15 | 58610517 | 58610998 | ADAM10 | 0.0182542 | 0.18554 | 0.0377654 |
| 205 | 15 | 67170604 | 67181240 | SMAD3 | 0.0169668 | 0.179396 | 0.0170029 |
| 206 | 15 | 68711706 | 68713924 | CORO2B | 0.0479403 | 0.199891 | 0.00661981 |
| 207 | 15 | 68718810 | 68719143 | CORO2B | 0.0479515 | 0.192747 | 0.00135666 |
| 208 | 15 | 74842195 | 74842279 | ULK3 | 0.0515654 | 0.138831 | 0.0340259 |
| 209 | 15 | 74852186 | 74854020 | SCAMP2 | 0.5 | 0.0434108 | 0.0342936 |
| 210 | 15 | 74904712 | 74905153 | FAM219B | 0.188004 | 0.391359 | 0.00665995 |
| 211 | 15 | 75339344 | 75339701 | COMMD4 | 0.144875 | 0.372883 | 0.0165718 |
| 212 | 15 | 82679800 | 82680174 | AP3B2 | 0.0750424 | 0.280331 | 0.00392569 |
| 213 | 15 | 82688831 | 82689157 | AP3B2 | 0.121841 | 0.283621 | 0.0212861 |
| 214 | 15 | 90231507 | 90232218 | CIB1 | 0.0584431 | 0.226359 | 0.0166545 |
| 215 | 15 | 90877024 | 90877134 | FURIN | 0.0150094 | 0.207921 | 0.0213726 |
| 216 | 15 | 90889443 | 90889516 | FES | 0.608696 | 0.0833333 | 0.0272 |
| 217 | 15 | 91006729 | 91006949 | VPS33B | 0.0324927 | 0.309735 | 0.0298959 |
| 218 | 16 | 1344525 | 1344600 | BAIAP3 | 0.120627 | 0.258824 | 0.034 |
| 219 | 16 | 1352306 | 1361742 | GNPTG | 0.0793783 | 0.163903 | 0.0361467 |
| 220 | 16 | 1459187 | 1460417 | CLCN7 | 0.191424 | 0.418559 | 0.0274248 |
| 221 | 16 | 1743476 | 1747028 | MAPK8IP3 | 0.171447 | 0.308402 | 0.0358139 |
| 222 | 16 | 2085322 | 2086192 | TSC2 | 0.059289 | 0.181664 | 0.0344448 |
| 223 | 16 | 2235143 | 2235215 | E4F1 | 0.125234 | 0.0120879 | 0.031117 |
| 224 | 16 | 2304150 | 2308449 | ABCA3 | 0.0178774 | 0.173038 | 0.039007 |
| 225 | 16 | 23478501 | 23478882 | GGA2 | 0.0415805 | 0.246714 | 0.0210432 |
| 226 | 16 | 2499420 | 2499834 | TBC1D24 | 0.0545273 | 0.250463 | 0.0256461 |
| 227 | 16 | 2528718 | 2528993 | AMDHD2 | 0.106248 | 0.46589 | 0.00934416 |
| 228 | 16 | 29813933 | 29814332 | PRRT2 | 0.0542638 | 0.177511 | 8.15E-05 |
| 229 | 16 | 29814465 | 29814627 | PRRT2 | 0.118116 | 0.333208 | 1.37E-05 |
| 230 | 16 | 29983664 | 29985212 | TAOK2 | 0.0731124 | 0.214755 | 0.0420859 |
| 231 | 16 | 29993370 | 29993458 | HIRIP3 | 0.0191926 | 0.212735 | 0.0238689 |
| 232 | 16 | 30083567 | 30083654 | PPP4C | 0.000301932 | 0.143786 | 0.00603772 |
| 233 | 16 | 30108264 | 30108372 | GDPD3 | 0.25 | 0.0013587 | 0.0138408 |
| 234 | 16 | 30186945 | 30187038 | CORO1A | 0.121563 | 0.23187 | 0.0358346 |
| 235 | 16 | 31034325 | 31034461 | STX4 | 0.188029 | 0.444976 | 0.0406926 |
| 236 | 16 | 31493048 | 31493466 | C16orf58 | 0.302293 | 0.575165 | 0.0152321 |
| 237 | 16 | 31493524 | 31493602 | C16orf58 | 0.289676 | 0.552 | 0.0134679 |
| 238 | 16 | 4802453 | 4802526 | ROGDI | 0.00889193 | 0.225735 | 0.00039846 |
| 239 | 16 | 50312191 | 50312889 | ADCY7 | 0.18198 | 0.756828 | 0.0375977 |
| 240 | 16 | 57121760 | 57123413 | CPNE2 | 0.0353624 | 0.295661 | 0.0007623 |
| 241 | 16 | 57249852 | 57250410 | ARL2BP | 0.00469199 | 0.114916 | 0.0276806 |
| 242 | 16 | 58509352 | 58511421 | NDRG4 | 0.0364897 | 0.114479 | 4.16E-06 |
| 243 | 16 | 66809075 | 66810373 | NAE1 | 0.0482287 | 0.219907 | 0.0349131 |
| 244 | 16 | 66823600 | 66824854 | NAE1 | 0.0317813 | 0.176062 | 0.0421774 |

| S/N | Chr | Start | End | Gene | IR ratio-Young | IR ratio-Old | *p*- value |
|---|---|---|---|---|---|---|---|
| 245 | 16 | 66824885 | 66826522 | NAE1 | 0.027118 | 0.300308 | 0.0216392 |
| 246 | 16 | 67000779 | 67000856 | CES4A | 0.0731707 | 0.325 | 0.00331327 |
| 247 | 16 | 67003360 | 67003514 | CES4A | 0.253961 | 0.14845 | 0.0285298 |
| 248 | 16 | 67659419 | 67659536 | ACD | 0.300813 | 0.691656 | 0.0170339 |
| 249 | 16 | 677388 | 677458 | RHBDL1 | 0.119857 | 0.60625 | 3.46E-05 |
| 250 | 16 | 677699 | 677780 | RHBDL1 | 0.371574 | 0.623842 | 0.030201 |
| 251 | 16 | 67881533 | 67881667 | EDC4 | 0.130435 | 0.381261 | 0.0436993 |
| 252 | 16 | 67883731 | 67883955 | EDC4 | 0.111516 | 0.358964 | 0.00831929 |
| 253 | 16 | 681880 | 682019 | STUB1 | 0.199451 | 0.295521 | 0.0281748 |
| 254 | 16 | 70481487 | 70481763 | COG4 | 0.0156986 | 0.222147 | 0.013189 |
| 255 | 16 | 72108382 | 72108472 | DHX38 | 0.0353144 | 0.193425 | 0.0158628 |
| 256 | 16 | 74472411 | 74474545 | GLG1 | 0.0150394 | 0.152747 | 0.022438 |
| 257 | 16 | 89693326 | 89693397 | CDK10 | 0.479167 | 0.74975 | 0.0304855 |
| 258 | 16 | 89693467 | 89694172 | CDK10 | 0.48796 | 0.870853 | 0.00221459 |
| 259 | 16 | 89962125 | 89963362 | DEF8 | 0.166101 | 0.40434 | 0.0222475 |
| 260 | 16 | 89963443 | 89964169 | DEF8 | 0.181696 | 0.409797 | 0.0263163 |
| 261 | 16 | 90037399 | 90037761 | AC133919.1 | 0.0504559 | 0.309002 | 0.0262237 |
| 262 | 17 | 16002025 | 16002792 | TTC19 | 0.0294741 | 0.212111 | 0.00626937 |
| 263 | 17 | 18245660 | 18245743 | FLII | 0.176181 | 0.364671 | 0.0170824 |
| 264 | 17 | 2041181 | 2041480 | DPH1 | 0.185395 | 0.355932 | 0.0487633 |
| 265 | 17 | 28342442 | 28344363 | TNFAIP1 | 0.043993 | 0.225369 | 0.00899708 |
| 266 | 17 | 28353316 | 28353694 | POLDIP2 | 0.0147033 | 0.134284 | 0.00105175 |
| 267 | 17 | 28396026 | 28396156 | SARM1 | 0.112754 | 0.42671 | 0.0369132 |
| 268 | 17 | 29082438 | 29086437 | MYO18A | 0.0676064 | 0.310694 | 0.00364757 |
| 269 | 17 | 32487162 | 32487475 | CDK5R1 | 0.00934143 | 0.333333 | 0.0311419 |
| 270 | 17 | 34256339 | 34256721 | CCL2 | 1 | 0.110477 | 0.00975059 |
| 271 | 17 | 38498510 | 38498931 | ARHGAP23 | 0.0547422 | 0.280053 | 0.0388679 |
| 272 | 17 | 39658026 | 39658404 | STARD3 | 0.12439 | 0.33504 | 0.00066248 |
| 273 | 17 | 39989929 | 39990093 | PSMD3 | 0.103035 | 0.224354 | 0.0159379 |
| 274 | 17 | 40035349 | 40035721 | MED24 | 0.00976944 | 0.111382 | 0.0159246 |
| 275 | 17 | 40167948 | 40168202 | CASC3 | 0.142232 | 0.439359 | 0.00030035 |
| 276 | 17 | 41976770 | 41977260 | DNAJC7 | 0.245454 | 0.474033 | 8.47E-05 |
| 277 | 17 | 42569303 | 42569506 | MLX | 0.1 | 0.395022 | 0.0327148 |
| 278 | 17 | 42582806 | 42583497 | FAM134C | 0.00745885 | 0.253807 | 0.041477 |
| 279 | 17 | 42684229 | 42684990 | CNTNAP1 | 0.0722453 | 0.27349 | 0.00350455 |
| 280 | 17 | 42685420 | 42685956 | CNTNAP1 | 0.0384598 | 0.185399 | 0.0107278 |
| 281 | 17 | 42691511 | 42691805 | CNTNAP1 | 0.0559006 | 0.134388 | 0.0123657 |
| 282 | 17 | 43202712 | 43203680 | NBR1 | 0.0369339 | 0.156779 | 0.0357907 |
| 283 | 17 | 44853635 | 44854268 | EFTUD2 | 0.101802 | 0.283778 | 0.0457991 |
| 284 | 17 | 45103876 | 45104858 | NMT1 | 0.0379043 | 0.208266 | 0.00265777 |
| 285 | 17 | 46830918 | 46831447 | WNT3 | 0.0932755 | 0.408769 | 0.04096 |
| 286 | 17 | 4717276 | 4717684 | ARRB2 | 0.0512129 | 0.273553 | 0.0257892 |
| 287 | 17 | 4718023 | 4718260 | ARRB2 | 0.14455 | 0.267918 | 0.0477769 |
| 288 | 17 | 4718345 | 4718611 | ARRB2 | 0.0935832 | 0.280834 | 0.0015978 |
| 289 | 17 | 47619164 | 47619736 | NPEPPS | 0.107594 | 0.298461 | 0.0389428 |
| 290 | 17 | 4886198 | 4886450 | MINK1 | 0.0433085 | 0.337227 | 4.29E-05 |
| 291 | 17 | 48910880 | 48912833 | UBE2Z | 0.0495626 | 0.194499 | 0.0282903 |
| 292 | 17 | 4894093 | 4894173 | MINK1 | 0.0583756 | 0.205047 | 0.011458 |
| 293 | 17 | 4894311 | 4894524 | MINK1 | 0.0964621 | 0.284682 | 0.00205163 |

| S/N | Chr | Start | End | Gene | IR ratio-Young | IR ratio-Old | *p*- value |
|---|---|---|---|---|---|---|---|
| 294 | 17 | 4938975 | 4939059 | SLC25A11 | 0.0326296 | 0.146205 | 0.00921303 |
| 295 | 17 | 4969337 | 4969499 | CAMTA2 | 0.0136986 | 0.158117 | 0.00256176 |
| 296 | 17 | 5377305 | 5378176 | RABEP1 | 0.151067 | 0.765836 | 0.0303646 |
| 297 | 17 | 58005600 | 58005800 | SRSF1 | 0.222307 | 0.553398 | 0.00109955 |
| 298 | 17 | 58310751 | 58310835 | TSPOAP1 | 0.147952 | 0.397129 | 0.00964405 |
| 299 | 17 | 63824411 | 63824636 | FTSJ3 | 0.00830258 | 0.536 | 0.0104166 |
| 300 | 17 | 7222302 | 7222666 | ACADVL | 0.720588 | 0.522695 | 0.00270742 |
| 301 | 17 | 74249080 | 74249343 | TTYH2 | 0.107501 | 0.282441 | 0.0352238 |
| 302 | 17 | 74958478 | 74958672 | HID1 | 0.262401 | 0.467506 | 0.0315542 |
| 303 | 17 | 75492789 | 75492928 | TMEM94 | 0.268629 | 0.0605613 | 0.0123927 |
| 304 | 17 | 75703859 | 75704755 | SAP30BP | 0.0138622 | 0.100621 | 0.0466295 |
| 305 | 17 | 7578244 | 7578341 | EIF4A1 | 0.0615779 | 0.137766 | 0.00558139 |
| 306 | 17 | 76391613 | 76391755 | UBE2O | 0.0281414 | 0.179558 | 0.0162568 |
| 307 | 17 | 7909338 | 7910427 | CHD3 | 0.162439 | 0.317517 | 0.0183912 |
| 308 | 17 | 80358479 | 80360060 | RNF213 | 0.640752 | 0.118868 | 0.023195 |
| 309 | 17 | 81690234 | 81690673 | HGS | 0.0270708 | 0.18887 | 0.00452703 |
| 310 | 17 | 81690742 | 81691446 | HGS | 0.0278871 | 0.240718 | 0.00255648 |
| 311 | 17 | 81694853 | 81694923 | HGS | 0.0128946 | 0.207485 | 0.00117896 |
| 312 | 17 | 82478918 | 82481081 | NARF | 0.0361515 | 0.162749 | 0.0274273 |
| 313 | 17 | 82716896 | 82718905 | FN3KRP | 0.093244 | 0.314015 | 0.0356175 |
| 314 | 17 | 82907821 | 82909284 | TBCD | 0.0494846 | 0.194114 | 0.0303228 |
| 315 | 19 | 10110866 | 10110944 | PPAN-P2RY11 | 0.117647 | 0.0101892 | 0.0473209 |
| 316 | 19 | 1011195 | 1011366 | TMEM259 | 0.146558 | 0.241905 | 0.0301279 |
| 317 | 19 | 10149968 | 10151397 | DNMT1 | 0.0367102 | 0.262953 | 0.042875 |
| 318 | 19 | 10258522 | 10258608 | MRPL4 | 0.199524 | 0.460091 | 0.00972702 |
| 319 | 19 | 10395343 | 10395434 | CDC37 | 0.192624 | 0.318257 | 0.00127335 |
| 320 | 19 | 11378595 | 11378690 | EPOR | 0.569672 | 0.10596 | 0.0081368 |
| 321 | 19 | 12679543 | 12679622 | DHPS | 0.0163934 | 0.130419 | 0.00963671 |
| 322 | 19 | 12701672 | 12701751 | TNPO2 | 0.0395452 | 0.159 | 0.0164134 |
| 323 | 19 | 12948813 | 12949080 | RAD23A | 0.112725 | 0.359652 | 0.012882 |
| 324 | 19 | 1366381 | 1367159 | MUM1 | 0.010342 | 0.183821 | 0.00702556 |
| 325 | 19 | 1367220 | 1369264 | MUM1 | 0.0181021 | 0.175977 | 0.0408947 |
| 326 | 19 | 1383942 | 1387810 | NDUFS7 | 0.0249706 | 0.172888 | 0.00587342 |
| 327 | 19 | 14159773 | 14160111 | ADGRL1 | 0.0084507 | 0.102804 | 0.0244705 |
| 328 | 19 | 1422396 | 1425877 | DAZAP1 | 0.193028 | 0.54566 | 0.0070872 |
| 329 | 19 | 1425960 | 1428841 | DAZAP1 | 0.16042 | 0.545123 | 0.00510745 |
| 330 | 19 | 16519715 | 16520148 | CHERP | 0.134128 | 0.342871 | 0.0406016 |
| 331 | 19 | 17210380 | 17210714 | MYO9B | 0.140141 | 0.481291 | 0.0247353 |
| 332 | 19 | 18122751 | 18123216 | MAST3 | 0.0372493 | 0.138519 | 0.0158381 |
| 333 | 19 | 18436877 | 18436972 | ISYNA1 | 0.179229 | 0.588235 | 0.0286865 |
| 334 | 19 | 18618272 | 18618374 | TMEM59L | 0.0713909 | 0.153652 | 1.20E-05 |
| 335 | 19 | 19055928 | 19057415 | ARMC6 | 0.0956362 | 0.369094 | 0.0352068 |
| 336 | 19 | 19656916 | 19656993 | ATP13A1 | 0.177986 | 0.591793 | 0.0369191 |
| 337 | 19 | 1978511 | 1978601 | CSNK1G2 | 0.030303 | 0.201597 | 0.0288346 |
| 338 | 19 | 2111332 | 2111678 | AP3D1 | 0.0321168 | 0.162774 | 0.0213329 |
| 339 | 19 | 2225452 | 2226182 | DOT1L | 0.0591925 | 0.636134 | 0.0357628 |
| 340 | 19 | 2476630 | 2477028 | GADD45B | 0.0617064 | 0.300757 | 0.0132812 |
| 341 | 19 | 2757782 | 2759256 | SGTA | 0.0278746 | 0.191682 | 0.00172645 |
| 342 | 19 | 35126775 | 35126852 | LGI4 | 0.0434783 | 0.221902 | 0.0456794 |

| S/N | Chr | Start | End | Gene | IR ratio-Young | IR ratio-Old | *p*- value |
|---|---|---|---|---|---|---|---|
| 343 | 19 | 35131555 | 35131788 | LGI4 | 0.00623886 | 0.117975 | 0.001117 |
| 344 | 19 | 35752999 | 35753086 | LIN37 | 0.271812 | 0.0344828 | 0.0177904 |
| 345 | 19 | 38217591 | 38217797 | DPF1 | 0.0457934 | 0.167389 | 0.0401406 |
| 346 | 19 | 38307521 | 38307596 | YIF1B | 0.0292135 | 0.163622 | 0.00747199 |
| 347 | 19 | 38711361 | 38714468 | ACTN4 | 0.0533577 | 0.152489 | 0.0135612 |
| 348 | 19 | 38918008 | 38918093 | CTC-360G5.8 | 0.120567 | 0.477528 | 0.00785794 |
| 349 | 19 | 39980146 | 39980285 | PSMC4 | 0.0772255 | 0.253521 | 0.00567267 |
| 350 | 19 | 40238973 | 40240044 | AKT2 | 0.295467 | 0.631763 | 0.00363026 |
| 351 | 19 | 40554646 | 40556083 | SPTBN4 | 0.136247 | 0.376928 | 0.0137471 |
| 352 | 19 | 40578212 | 40578461 | SHKBP1 | 0.158606 | 0.484054 | 0.0413018 |
| 353 | 19 | 42294735 | 42294823 | CIC | 0.0238095 | 0.144957 | 0.0288843 |
| 354 | 19 | 42316561 | 42316661 | TMEM145 | 0.0446001 | 0.155505 | 0.0154448 |
| 355 | 19 | 4310596 | 4311841 | FSD1 | 0.202776 | 0.456294 | 0.0110802 |
| 356 | 19 | 436431 | 436629 | SHC2 | 0.294686 | 0.46963 | 0.0231478 |
| 357 | 19 | 49114967 | 49115259 | LIN7B | 0.013296 | 0.124947 | 0.0479763 |
| 358 | 19 | 49447908 | 49448000 | PIH1D1 | 0.0448296 | 0.198953 | 0.0124926 |
| 359 | 19 | 49686726 | 49688161 | PRMT1 | 0.070527 | 0.190075 | 0.00739252 |
| 360 | 19 | 49802141 | 49802533 | AP2A1 | 0.0576923 | 0.184932 | 0.0202666 |
| 361 | 19 | 49855077 | 49856974 | PTOV1 | 0.15409 | 0.34205 | 0.00017129 |
| 362 | 19 | 49857130 | 49857692 | PTOV1 | 0.0607453 | 0.133223 | 0.0160595 |
| 363 | 19 | 49861365 | 49861448 | PNKP | 0.283967 | 0.0778032 | 0.0324206 |
| 364 | 19 | 5239063 | 5240198 | AC005338.1 | 0.0114945 | 0.150792 | 0.0250667 |
| 365 | 19 | 54143749 | 54144005 | CNOT3 | 0.0113301 | 0.161017 | 0.0280078 |
| 366 | 19 | 54436171 | 54436332 | TTYH1 | 0.0594311 | 0.120105 | 0.0112937 |
| 367 | 19 | 55304110 | 55304550 | BRSK1 | 0.0536585 | 0.211297 | 0.00096891 |
| 368 | 19 | 58548776 | 58548861 | TRIM28 | 0.14606 | 0.295855 | 0.0449977 |
| 369 | 19 | 58549650 | 58549736 | TRIM28 | 0.0315977 | 0.142497 | 0.009126 |
| 370 | 19 | 614016 | 615794 | HCN2 | 0.076804 | 0.201993 | 0.0183536 |
| 371 | 19 | 7527628 | 7527863 | MCOLN1 | 0.129958 | 0.427313 | 0.0274578 |
| 372 | 19 | 7627851 | 7628149 | XAB2 | 0.0180702 | 0.260474 | 0.0133701 |
| 373 | 19 | 8255879 | 8256235 | CERS4 | 0.024 | 0.253689 | 0.0002724 |
| 374 | 19 | 877180 | 879936 | MED16 | 0.158986 | 0.557341 | 0.0134091 |
| 375 | 19 | 990364 | 990851 | WDR18 | 0.183761 | 0.503779 | 0.0424213 |
| 376 | 2 | 101269136 | 101269215 | CNOT11 | 0.00986547 | 0.235659 | 0.037656 |
| 377 | 2 | 127050912 | 127051153 | BIN1 | 0.0719368 | 0.256254 | 1.84E-05 |
| 378 | 2 | 127052362 | 127053421 | BIN1 | 0.173368 | 0.472114 | 0.00010386 |
| 379 | 2 | 127289501 | 127289688 | ERCC3 | 0.112546 | 0.312743 | 0.0338333 |
| 380 | 2 | 127289824 | 127290223 | ERCC3 | 0.272356 | 0.596689 | 0.00957386 |
| 381 | 2 | 130372483 | 130372872 | PTPN18 | 0.280339 | 0.0534521 | 0.0321042 |
| 382 | 2 | 131041462 | 131041814 | ARHGEF4 | 0.209145 | 0.561997 | 7.12E-06 |
| 383 | 2 | 131045446 | 131046037 | ARHGEF4 | 0.0749096 | 0.363588 | 2.23E-08 |
| 384 | 2 | 131518708 | 131519049 | AC093838.4 | 0.180025 | 0.542326 | 0.0281253 |
| 385 | 2 | 169139371 | 169139542 | LRP2 | 0.2 | 0.0154912 | 0.0204082 |
| 386 | 2 | 169806892 | 169806970 | SSB | 0.0205761 | 0.266667 | 0.0370225 |
| 387 | 2 | 177216188 | 177216502 | HNRNPA3 | 0.0288049 | 0.10835 | 0.0212076 |
| 388 | 2 | 186504214 | 186505450 | ZC3H15 | 0.0124438 | 0.121286 | 0.0296539 |
| 389 | 2 | 197494250 | 197494656 | HSPD1 | 0.0345953 | 0.136492 | 0.0348361 |
| 390 | 2 | 201704218 | 201704453 | ALS2 | 0.0136986 | 0.147811 | 0.0203634 |
| 391 | 2 | 218340141 | 218340727 | PNKD | 0.093288 | 0.35322 | 0.0360215 |

| S/N | Chr | Start | End | Gene | IR ratio-Young | IR ratio-Old | *p*- value |
|---|---|---|---|---|---|---|---|
| 392 | 2 | 218812749 | 218812923 | CYP27A1 | 0.126273 | 0.0322151 | 0.0441813 |
| 393 | 2 | 219208348 | 219208425 | ZFAND2B | 0.0873239 | 0.223136 | 0.0452394 |
| 394 | 2 | 219233943 | 219234132 | ANKZF1 | 0.358524 | 0.119742 | 0.0223437 |
| 395 | 2 | 219299384 | 219299699 | PTPRN | 0.0521176 | 0.1393 | 0.00545905 |
| 396 | 2 | 219556292 | 219556453 | OBSL1 | 0.025641 | 0.147937 | 0.0315528 |
| 397 | 2 | 219612058 | 219613127 | STK11IP | 0.0613958 | 0.283305 | 0.0351563 |
| 398 | 2 | 219633009 | 219633273 | SLC4A3 | 0.104906 | 0.0110901 | 0.0455296 |
| 399 | 2 | 219633457 | 219633879 | SLC4A3 | 0.0867003 | 0.278295 | 0.0355535 |
| 400 | 2 | 229771632 | 229774096 | TRIP12 | 0.0125412 | 0.118373 | 0.0479921 |
| 401 | 2 | 232566981 | 232567077 | EIF4E2 | 0.00370714 | 0.138756 | 0.0341145 |
| 402 | 2 | 241499414 | 241500172 | STK25 | 0.227577 | 0.374602 | 0.0183588 |
| 403 | 2 | 241671405 | 241672190 | ATG4B | 0.306306 | 0.600371 | 0.00338231 |
| 404 | 2 | 25136598 | 25137340 | EFR3B | 0.0723926 | 0.318568 | 0.0439158 |
| 405 | 2 | 27039837 | 27040029 | TMEM214 | 0.0333333 | 0.330317 | 0.00685871 |
| 406 | 2 | 27200579 | 27200997 | SLC5A6 | 0.0321721 | 0.200585 | 0.0310968 |
| 407 | 2 | 27434762 | 27435132 | NRBP1 | 0.0458588 | 0.183475 | 0.0141701 |
| 408 | 2 | 38296590 | 38296685 | ATL2 | 0.223301 | 0.593023 | 0.0386147 |
| 409 | 2 | 53865573 | 53866084 | PSME4 | 0.0106865 | 0.224338 | 0.00189416 |
| 410 | 2 | 61189069 | 61190270 | USP34 | 0.184555 | 0.359183 | 0.0281668 |
| 411 | 2 | 74365241 | 74365514 | DCTN1 | 0.0854701 | 0.220401 | 0.00121183 |
| 412 | 2 | 74368131 | 74368727 | DCTN1 | 0.0440996 | 0.108073 | 0.0194173 |
| 413 | 2 | 74425491 | 74425569 | WDR54 | 0.0327724 | 0.151515 | 0.0289261 |
| 414 | 2 | 74527838 | 74527939 | AUP1 | 0.0881971 | 0.173221 | 0.0469728 |
| 415 | 2 | 8682906 | 8683684 | ID2 | 0.103976 | 0.199739 | 0.0210218 |
| 416 | 2 | 96268658 | 96269267 | CIAO1 | 0.146109 | 0.298614 | 0.0362054 |
| 417 | 2 | 97656960 | 97657151 | ACTR1B | 0.0693663 | 0.537211 | 6.84E-11 |
| 418 | 2 | 97658643 | 97658878 | ACTR1B | 0.201339 | 0.476216 | 6.39E-06 |
| 419 | 20 | 2659793 | 2660029 | IDH3B | 0.136854 | 0.222334 | 0.0459573 |
| 420 | 20 | 2660365 | 2660456 | IDH3B | 0.083809 | 0.174421 | 0.025752 |
| 421 | 20 | 2864287 | 2864364 | PTPRA | 0.0121637 | 0.152174 | 0.0067 |
| 422 | 20 | 31945889 | 31946495 | PDRG1 | 0.0163664 | 0.152131 | 0.0381189 |
| 423 | 20 | 32703458 | 32704022 | COMMD7 | 0.108683 | 0.284392 | 0.0288964 |
| 424 | 20 | 33439026 | 33443310 | SNTA1 | 0.00958825 | 0.110745 | 0.0495948 |
| 425 | 20 | 34921139 | 34921329 | ACSS2 | 0.114912 | 0.44294 | 0.0115851 |
| 426 | 20 | 34989919 | 34990013 | MYH7B | 1 | 0.221374 | 0.0344048 |
| 427 | 20 | 35007640 | 35008663 | TRPC4AP | 0.0211771 | 0.10561 | 0.0360689 |
| 428 | 20 | 35631034 | 35631113 | CPNE1 | 0.0412075 | 0.136295 | 0.0369903 |
| 429 | 20 | 3673911 | 3674063 | ADAM33 | 0.0345188 | 0.356449 | 0.0254804 |
| 430 | 20 | 3934201 | 3934993 | RNF24 | 0.00875778 | 0.2 | 0.0342936 |
| 431 | 20 | 41163275 | 41163377 | PLCG1 | 0.115593 | 0.399297 | 0.00911302 |
| 432 | 20 | 41170269 | 41172192 | PLCG1 | 0.024869 | 0.160751 | 0.0173848 |
| 433 | 20 | 45897806 | 45898004 | CTSA | 0.181755 | 0.336216 | 0.00914175 |
| 434 | 20 | 46035042 | 46035403 | SLC12A5 | 0.0191835 | 0.100654 | 0.0330331 |
| 435 | 20 | 46356637 | 46357622 | SLC35C2 | 0.0695113 | 0.382995 | 0.00108543 |
| 436 | 20 | 59037280 | 59038465 | PRELID3B | 0.0243353 | 0.218552 | 0.0270336 |
| 437 | 20 | 5941139 | 5941242 | TRMT6 | 0.00738916 | 0.107394 | 0.0489691 |
| 438 | 20 | 62333243 | 62333374 | LAMA5 | 0.620155 | 0.0938834 | 0.019073 |
| 439 | 20 | 62941620 | 62942986 | GID8 | 0.0460506 | 0.249008 | 0.0365176 |
| 440 | 20 | 63678346 | 63679848 | RTEL1 | 0.0148202 | 0.1555 | 0.0242565 |

| S/N | Chr | Start | End | Gene | IR ratio-Young | IR ratio-Old | *p*- value |
|---|---|---|---|---|---|---|---|
| 441 | 21 | 29043599 | 29046666 | USP16 | 0.0913205 | 0.750561 | 0.015625 |
| 442 | 21 | 38814981 | 38817007 | ETS2 | 0.0171748 | 0.101047 | 0.0171092 |
| 443 | 21 | 44314021 | 44316243 | PFKL | 0.0706194 | 0.238111 | 0.00644794 |
| 444 | 21 | 44324917 | 44325152 | PFKL | 0.0389439 | 0.131401 | 0.01497 |
| 445 | 21 | 44325264 | 44325960 | PFKL | 0.0444989 | 0.161992 | 0.00828163 |
| 446 | 21 | 46195756 | 46196201 | LSS | 0.0559676 | 0.288939 | 0.0105901 |
| 447 | 22 | 18910272 | 18910887 | DGCR6 | 0.0748364 | 0.35905 | 0.00868347 |
| 448 | 22 | 20113007 | 20113117 | TRMT2A | 0.0196078 | 0.164867 | 0.0422608 |
| 449 | 22 | 20139892 | 20140114 | ZDHHC8 | 0.173935 | 0.365207 | 0.0451989 |
| 450 | 22 | 20141012 | 20141216 | ZDHHC8 | 0.0688202 | 0.277778 | 0.0357009 |
| 451 | 22 | 20400758 | 20401276 | ZNF74 | 0.0404533 | 1 | 0.0145773 |
| 452 | 22 | 21025898 | 21026010 | P2RX6 | 0.439065 | 0.125 | 0.0403612 |
| 453 | 22 | 21703882 | 21710627 | YPEL1 | 0.0158577 | 0.107056 | 0.0449492 |
| 454 | 22 | 26465551 | 26466225 | HPS4 | 0.237692 | 0.60445 | 0.0374027 |
| 455 | 22 | 28796192 | 28797076 | XBP1 | 0.0207283 | 0.21875 | 0.0480841 |
| 456 | 22 | 30887618 | 30888222 | OSBP2 | 0.00865969 | 0.333333 | 0.0351562 |
| 457 | 22 | 38073823 | 38074306 | PICK1 | 0.372905 | 0.719118 | 0.0042115 |
| 458 | 22 | 40423685 | 40424205 | MKL1 | 0.0265406 | 0.261872 | 0.0452675 |
| 459 | 22 | 41224801 | 41224966 | L3MBTL2 | 0.0179514 | 0.262397 | 0.00646659 |
| 460 | 22 | 41225071 | 41225793 | L3MBTL2 | 0.0122163 | 0.10846 | 0.0250221 |
| 461 | 22 | 41584058 | 41584280 | PMM1 | 0.060241 | 0.150765 | 0.0336884 |
| 462 | 22 | 46355588 | 46355989 | TRMU | 0.128642 | 0.468418 | 0.00312764 |
| 463 | 22 | 46356072 | 46356841 | TRMU | 0.136984 | 0.466395 | 0.0278038 |
| 464 | 22 | 50245827 | 50245909 | HDAC10 | 0.0544413 | 0.23951 | 0.0419856 |
| 465 | 22 | 50265660 | 50266225 | MAPK11 | 0.361165 | 0.604772 | 0.022102 |
| 466 | 22 | 50266305 | 50266539 | MAPK11 | 0.0996999 | 0.201044 | 0.0393909 |
| 467 | 22 | 50441026 | 50443865 | PPP6R2 | 0.213731 | 0.772065 | 0.00031746 |
| 468 | 22 | 50459392 | 50459469 | SBF1 | 0.0882579 | 0.220365 | 0.0394526 |
| 469 | 22 | 50459666 | 50459951 | SBF1 | 0.0854998 | 0.294031 | 0.00442783 |
| 470 | 22 | 50574419 | 50574492 | CPT1B | 0.262295 | 0.0512821 | 0.0276847 |
| 471 | 22 | 50581553 | 50581748 | CHKB | 0.460575 | 0.759475 | 0.0296398 |
| 472 | 22 | 50626760 | 50626833 | ARSA | 0.397942 | 0.728328 | 0.0180606 |
| 473 | 3 | 127017892 | 127018293 | PLXNA1 | 0.00958445 | 0.146739 | 0.0146878 |
| 474 | 3 | 127029536 | 127029873 | PLXNA1 | 0.0771822 | 0.237263 | 0.0432643 |
| 475 | 3 | 12908525 | 12909272 | IQSEC1 | 0.031669 | 0.101432 | 0.0404641 |
| 476 | 3 | 13323432 | 13325794 | NUP210 | 0.046963 | 0.530409 | 0.0233236 |
| 477 | 3 | 133777238 | 133778585 | TF | 0.191044 | 0.279945 | 0.00923342 |
| 478 | 3 | 184143142 | 184143441 | EIF2B5 | 0.34013 | 0.693637 | 0.00172216 |
| 479 | 3 | 184164931 | 184165112 | DVL3 | 0.168675 | 0.404005 | 0.0477703 |
| 480 | 3 | 184381624 | 184381715 | CHRD | 0.025641 | 0.147601 | 0.0368061 |
| 481 | 3 | 37065942 | 37066223 | LRRFIP2 | 0.182208 | 0.471871 | 0.0382875 |
| 482 | 3 | 38251471 | 38252327 | OXSR1 | 0.0281447 | 0.527778 | 0.0109739 |
| 483 | 3 | 38252392 | 38252816 | OXSR1 | 0.0192605 | 0.247166 | 0.0116219 |
| 484 | 3 | 46672201 | 46673338 | ALS2CL | 0.327613 | 0.0491093 | 0.0429688 |
| 485 | 3 | 47406750 | 47407129 | PTPN23 | 0.15373 | 0.522627 | 0.0369903 |
| 486 | 3 | 48412009 | 48412237 | PLXNB1 | 0.0597377 | 0.225404 | 0.0411379 |
| 487 | 3 | 48628038 | 48628433 | SLC26A6 | 0.676856 | 0.211568 | 0.0489273 |
| 488 | 3 | 48678969 | 48679054 | NCKIPSD | 0.0659971 | 0.177598 | 0.0188111 |
| 489 | 3 | 49014116 | 49014209 | WDR6 | 0.508993 | 0.722672 | 0.0256911 |

| S/N | Chr | Start | End | Gene | IR ratio-Young | IR ratio-Old | *p*- value |
|---|---|---|---|---|---|---|---|
| 490 | 3 | 49015707 | 49015803 | DALRD3 | 0.719098 | 0.934471 | 0.00168462 |
| 491 | 3 | 49024404 | 49024494 | IMPDH2 | 0.0245195 | 0.135854 | 0.0248231 |
| 492 | 3 | 49098692 | 49098884 | QARS | 0.189452 | 0.476935 | 0.0193145 |
| 493 | 3 | 49114093 | 49114173 | USP19 | 0.0222222 | 0.150289 | 0.0244869 |
| 494 | 3 | 49116607 | 49116726 | USP19 | 0.105012 | 0.414062 | 0.012253 |
| 495 | 3 | 49123373 | 49123446 | LAMB2 | 0.463068 | 0.129032 | 0.0294076 |
| 496 | 3 | 49125169 | 49125252 | LAMB2 | 0.409524 | 0.0183374 | 0.0459184 |
| 497 | 3 | 49129324 | 49129603 | LAMB2 | 0.141732 | 0.642388 | 0.0355124 |
| 498 | 3 | 49674413 | 49674488 | APEH | 0.010084 | 0.333333 | 0.018595 |
| 499 | 3 | 49679644 | 49680540 | APEH | 0.0368879 | 0.127678 | 0.0340371 |
| 500 | 3 | 49860852 | 49860942 | CAMKV | 0.117664 | 0.27572 | 0.00208331 |
| 501 | 3 | 49861577 | 49861790 | CAMKV | 0.104386 | 0.178053 | 0.0346145 |
| 502 | 3 | 50364806 | 50364887 | CACNA2D2 | 0.0733083 | 0.379679 | 0.0243861 |
| 503 | 3 | 50366338 | 50366577 | CACNA2D2 | 0.0675134 | 0.349237 | 0.0423057 |
| 504 | 3 | 50366625 | 50366830 | CACNA2D2 | 0.142857 | 0.548088 | 0.0437436 |
| 505 | 3 | 50367902 | 50368137 | CACNA2D2 | 0.0454545 | 0.371166 | 0.0118542 |
| 506 | 3 | 51275206 | 51277607 | DOCK3 | 0.0411096 | 0.277327 | 0.0276502 |
| 507 | 3 | 51277754 | 51280105 | DOCK3 | 0.0042172 | 0.220819 | 0.00666389 |
| 508 | 3 | 52504876 | 52505002 | STAB1 | 1 | 0.146919 | 0.01585 |
| 509 | 3 | 53185700 | 53185926 | PRKCD | 0.0116929 | 0.111111 | 0.04 |
| 510 | 3 | 58285139 | 58285352 | ABHD6 | 0.0373324 | 0.170518 | 0.0345831 |
| 511 | 3 | 72378637 | 72379039 | RYBP | 0.0148003 | 0.139486 | 0.0366364 |
| 512 | 3 | 9842082 | 9842198 | RPUSD3 | 0.117647 | 0.323572 | 0.0408477 |
| 513 | 4 | 113237172 | 113237598 | ANK2 | 0.00725468 | 0.166483 | 0.0285279 |
| 514 | 4 | 143540495 | 143543508 | SMARCA5 | 0.0279587 | 0.213772 | 0.0474272 |
| 515 | 4 | 153322816 | 153324077 | TRIM2 | 0.041438 | 0.183401 | 0.0418379 |
| 516 | 4 | 157335877 | 157336376 | GRIA2 | 0.0594571 | 0.163966 | 0.00630074 |
| 517 | 4 | 186197130 | 186197532 | CYP4V2 | 1 | 0.250216 | 0.0242801 |
| 518 | 4 | 25392421 | 25393804 | ANAPC4 | 0.607303 | 0.131275 | 0.048769 |
| 519 | 4 | 55417247 | 55417802 | TMEM165 | 0.173585 | 0.428921 | 0.0492603 |
| 520 | 4 | 55424643 | 55425375 | TMEM165 | 0.0306261 | 0.143668 | 0.033854 |
| 521 | 4 | 679033 | 679913 | MYL5 | 0.0145648 | 0.211211 | 0.0354672 |
| 522 | 4 | 76100824 | 76100989 | ART3 | 0.1936 | 0.0166945 | 0.0410021 |
| 523 | 4 | 78595437 | 78595793 | ANXA3 | 1 | 0.144737 | 0.013545 |
| 524 | 4 | 80196601 | 80196962 | PRDM8 | 0.0416382 | 0.337639 | 0.0476895 |
| 525 | 4 | 99949386 | 99949662 | H2AFZ | 0.0352253 | 0.130029 | 0.000175 |
| 526 | 5 | 126488892 | 126492914 | GRAMD3 | 0.142847 | 0.510126 | 0.0355402 |
| 527 | 5 | 140561701 | 140561843 | APBB3 | 0.460402 | 0.713886 | 0.0181584 |
| 528 | 5 | 140563915 | 140564196 | APBB3 | 0.157736 | 0.423398 | 0.00795338 |
| 529 | 5 | 140677753 | 140677907 | HARS | 0.0498076 | 0.135142 | 0.010759 |
| 530 | 5 | 141529273 | 141529602 | DIAPH1 | 0.122449 | 0.509195 | 0.0457558 |
| 531 | 5 | 141639649 | 141639919 | RELL2 | 0.0936819 | 0.246698 | 0.0244595 |
| 532 | 5 | 141640295 | 141640411 | RELL2 | 0.0833333 | 0.227166 | 0.0294641 |
| 533 | 5 | 141643088 | 141644217 | FCHSD1 | 0.432577 | 0.0761985 | 0.0182961 |
| 534 | 5 | 141645148 | 141645770 | FCHSD1 | 0.272189 | 0.00578739 | 0.04 |
| 535 | 5 | 146144349 | 146144471 | LARS | 0.0335037 | 0.170424 | 0.035327 |
| 536 | 5 | 14711310 | 14712873 | ANKH | 0.0785866 | 0.252237 | 0.0190471 |
| 537 | 5 | 1479675 | 1480941 | LPCAT1 | 0.01865 | 0.147601 | 0.0470893 |
| 538 | 5 | 149251419 | 149252200 | ABLIM3 | 0.0167108 | 0.21828 | 0.0197754 |

| S/N | Chr | Start | End | Gene | IR ratio-Young | IR ratio-Old | *p*- value |
|---|---|---|---|---|---|---|---|
| 539 | 5 | 173608292 | 173609237 | BOD1 | 0.0115962 | 0.132889 | 0.0252576 |
| 540 | 5 | 176529090 | 176529287 | RNF44 | 0.0528129 | 0.22292 | 0.0116532 |
| 541 | 5 | 177302178 | 177302396 | RAB24 | 0.357292 | 0.580514 | 0.00867762 |
| 542 | 5 | 179621397 | 179623036 | HNRNPH1 | 0.134593 | 0.236272 | 0.0477743 |
| 543 | 5 | 179798434 | 179798512 | MGAT4B | 0.00120919 | 0.275 | 0.00018153 |
| 544 | 5 | 58458171 | 58458398 | PLK2 | 0.0650906 | 0.290464 | 0.017866 |
| 545 | 5 | 73554986 | 73555487 | ANKRA2 | 0.102408 | 0.343564 | 0.0304773 |
| 546 | 6 | 131596163 | 131596517 | MED23 | 0.0165407 | 0.348442 | 0.0174851 |
| 547 | 6 | 13615624 | 13615711 | NOL7 | 0.226611 | 0.0518617 | 0.04096 |
| 548 | 6 | 136344238 | 136345855 | MAP7 | 0.059879 | 0.418167 | 0.00161432 |
| 549 | 6 | 158066635 | 158068646 | SYNJ2 | 0.0226939 | 0.265029 | 0.021883 |
| 550 | 6 | 29627646 | 29629086 | GABBR1 | 0.239251 | 0.645416 | 0.00236182 |
| 551 | 6 | 29629107 | 29630457 | GABBR1 | 0.187901 | 0.605283 | 0.00146753 |
| 552 | 6 | 29670721 | 29671171 | MOG | 0.294372 | 0.556659 | 0.00015603 |
| 553 | 6 | 30741333 | 30741613 | FLOT1 | 0.152431 | 0.295737 | 0.00112594 |
| 554 | 6 | 30913157 | 30913308 | GTF2H4 | 0.157792 | 0.337817 | 0.0270775 |
| 555 | 6 | 31143134 | 31143261 | CCHCR1 | 0.0909091 | 0.476031 | 0.0484541 |
| 556 | 6 | 31625871 | 31626019 | PRRC2A | 0.0357143 | 0.220418 | 0.027862 |
| 557 | 6 | 31640306 | 31640384 | BAG6 | 0.00273428 | 0.11074 | 0.00513528 |
| 558 | 6 | 31760216 | 31760689 | MSH5 | 1 | 0.218227 | 0.0327738 |
| 559 | 6 | 31950147 | 31950285 | CFB | 1 | 0.0290049 | 0.00801866 |
| 560 | 6 | 31963559 | 31963636 | SKIV2L | 0.117647 | 0.00509122 | 0.0349411 |
| 561 | 6 | 32191503 | 32191708 | GPSM3 | 0.501577 | 0.018621 | 0.0233236 |
| 562 | 6 | 32977570 | 32977756 | BRD2 | 0.0302741 | 0.116564 | 0.0128132 |
| 563 | 6 | 33205539 | 33205639 | HSD17B8 | 0.0714286 | 0.389831 | 0.0491881 |
| 564 | 6 | 33264497 | 33264781 | VPS52 | 0.275885 | 0.647558 | 0.00024348 |
| 565 | 6 | 33442494 | 33442888 | SYNGAP1 | 0.0144928 | 0.177486 | 0.00185307 |
| 566 | 6 | 34247492 | 34247680 | C6orf1 | 0.0282209 | 0.146322 | 0.0189242 |
| 567 | 6 | 36798494 | 36799966 | CPNE5 | 0.0232847 | 0.108196 | 0.0420121 |
| 568 | 6 | 43259706 | 43262788 | TTBK1 | 0.121894 | 0.410215 | 0.0403612 |
| 569 | 6 | 43503508 | 43503622 | TJAP1 | 0.133333 | 0.0277778 | 0.0389118 |
| 570 | 6 | 43521048 | 43521181 | POLR1C | 0.0555556 | 0.292369 | 0.048282 |
| 571 | 6 | 53092824 | 53093465 | FBXO9 | 0.0372668 | 0.238146 | 3.64E-06 |
| 572 | 7 | 100106003 | 100106240 | AP4M1 | 0.331865 | 0.746165 | 0.0330983 |
| 573 | 7 | 100110073 | 100110199 | TAF6 | 0.057903 | 0.167025 | 0.0326253 |
| 574 | 7 | 100222720 | 100223569 | GATS | 0.0305297 | 0.133469 | 0.00646973 |
| 575 | 7 | 100575304 | 100575704 | LRCH4 | 0.340659 | 0.588649 | 0.0498306 |
| 576 | 7 | 100683230 | 100683303 | GIGYF1 | 0.0251451 | 0.203204 | 0.0449795 |
| 577 | 7 | 100888383 | 100888473 | SRRT | 0.0415973 | 0.124698 | 0.0391285 |
| 578 | 7 | 103312131 | 103312206 | PMPCB | 0.158442 | 0.658791 | 0.00065252 |
| 579 | 7 | 128324694 | 128325817 | RBM28 | 0.118896 | 0.718911 | 0.00419021 |
| 580 | 7 | 128394599 | 128394888 | IMPDH1 | 0.0170592 | 0.271215 | 0.012419 |
| 581 | 7 | 1438220 | 1438288 | MICALL2 | 0.501299 | 0.186701 | 0.0132999 |
| 582 | 7 | 148799388 | 148800501 | CUL1 | 0.016497 | 0.102256 | 0.0217263 |
| 583 | 7 | 151077780 | 151077878 | FASTK | 0.0860174 | 0.191454 | 0.0368178 |
| 584 | 7 | 26190460 | 26190992 | HNRNPA2B1 | 0.176634 | 0.265721 | 0.0485664 |
| 585 | 7 | 44761593 | 44761694 | ZMIZ2 | 0.0990869 | 0.30975 | 0.00161163 |
| 586 | 7 | 99047882 | 99049562 | SMURF1 | 0.0114475 | 0.622901 | 0.00462963 |
| 587 | 8 | 143379078 | 143379314 | RHPN1 | 0.0265871 | 0.170914 | 0.0228147 |

| S/N | Chr | Start | End | Gene | IR ratio-Young | IR ratio-Old | *p*- value |
|---|---|---|---|---|---|---|---|
| 588 | 8 | 143380783 | 143381267 | RHPN1 | 0.111339 | 0.26097 | 0.0335394 |
| 589 | 8 | 143811345 | 143812265 | SCRIB | 0.0714286 | 0.265416 | 0.0495688 |
| 590 | 8 | 143835730 | 143835819 | NRBP2 | 0.235741 | 0.390635 | 0.0265539 |
| 591 | 8 | 143836030 | 143836126 | NRBP2 | 0.0493465 | 0.128251 | 0.018522 |
| 592 | 8 | 143992078 | 143992244 | GRINA | 0.0700014 | 0.2009 | 0.00034064 |
| 593 | 8 | 144083500 | 144083713 | GPAA1 | 0.226373 | 0.476135 | 0.0195036 |
| 594 | 8 | 144098788 | 144098865 | SHARPIN | 0.18429 | 0.0439093 | 0.0171364 |
| 595 | 8 | 144258914 | 144259239 | MROH1 | 0.109218 | 0.279271 | 0.0419167 |
| 596 | 8 | 144262317 | 144262395 | BOP1 | 0.047619 | 0.205429 | 0.0189228 |
| 597 | 8 | 144262503 | 144262587 | BOP1 | 0.1067 | 0.342756 | 0.010004 |
| 598 | 8 | 144263761 | 144263830 | BOP1 | 0.225067 | 0.492754 | 0.0482879 |
| 599 | 8 | 144264616 | 144264713 | BOP1 | 0.216125 | 0.686701 | 0.00988442 |
| 600 | 8 | 144317109 | 144317186 | DGAT1 | 0.176471 | 0.416427 | 0.0314536 |
| 601 | 8 | 144317588 | 144317670 | DGAT1 | 0.173989 | 0.44912 | 0.00572475 |
| 602 | 8 | 144317712 | 144317783 | DGAT1 | 0.175069 | 0.376155 | 0.0478616 |
| 603 | 8 | 144423922 | 144424040 | VPS28 | 0.106546 | 0.200191 | 0.0493686 |
| 604 | 8 | 144510903 | 144510990 | MFSD3 | 0.0587045 | 0.265548 | 0.0219033 |
| 605 | 8 | 18025658 | 18027636 | PCM1 | 0.0231027 | 0.262963 | 0.0181763 |
| 606 | 8 | 22101530 | 22101707 | FAM160B2 | 0.0566544 | 0.308169 | 0.00269837 |
| 607 | 8 | 22154213 | 22154559 | LGI3 | 0.0777171 | 0.251813 | 0.00045757 |
| 608 | 8 | 22614503 | 22614837 | CCAR2 | 0.0466412 | 0.149033 | 0.0304356 |
| 609 | 8 | 22615001 | 22615424 | CCAR2 | 0.13437 | 0.371683 | 0.0384064 |
| 610 | 8 | 66444420 | 66444593 | ADHFE1 | 0.333333 | 0.0151057 | 0.04 |
| 611 | 9 | 113290588 | 113290699 | PRPF4 | 0.05 | 0.485356 | 0.0115623 |
| 612 | 9 | 113390676 | 113390797 | ALAD | 0.224138 | 0.588832 | 0.00933304 |
| 613 | 9 | 125354841 | 125355643 | GAPVD1 | 0.0350289 | 0.541787 | 0.0204082 |
| 614 | 9 | 127495418 | 127495963 | LRSAM1 | 0.0843271 | 0.374818 | 0.0351563 |
| 615 | 9 | 129005776 | 129006049 | NUP188 | 0.11699 | 0.398635 | 0.0406971 |
| 616 | 9 | 131511467 | 131512040 | POMT1 | 0.103701 | 0.322239 | 0.014138 |
| 617 | 9 | 136375800 | 136376348 | SNAPC4 | 0.0218182 | 0.170458 | 0.0490416 |
| 618 | 9 | 136433279 | 136434036 | INPP5E | 0.0953906 | 0.576247 | 0.00979386 |
| 619 | 9 | 136463132 | 136463462 | SEC16A | 0.125 | 1 | 0.00228161 |
| 620 | 9 | 136669721 | 136669913 | EGFL7 | 0.0175439 | 0.173052 | 0.00588262 |
| 621 | 9 | 136979299 | 136979945 | PTGDS | 0.204421 | 0.446221 | 1.39E-21 |
| 622 | 9 | 137017692 | 137017786 | ABCA2 | 0.0483654 | 0.120419 | 0.00765908 |
| 623 | 9 | 137021610 | 137021890 | ABCA2 | 0.0757528 | 0.244034 | 0.00271777 |
| 624 | 9 | 137040598 | 137040670 | NPDC1 | 0.0401477 | 0.136029 | 0.00014257 |
| 625 | 9 | 137185087 | 137186223 | ANAPC2 | 0.110586 | 0.394823 | 0.0178751 |
| 626 | 9 | 137428141 | 137428881 | NOXA1 | 0.113432 | 0.326538 | 0.0351578 |
| 627 | 9 | 137429016 | 137429275 | NOXA1 | 0.269175 | 0.59761 | 0.0256162 |
| 628 | 9 | 137455634 | 137456410 | NSMF | 0.0656293 | 0.136022 | 0.0100035 |
| 629 | 9 | 35555701 | 35555951 | RUSC2 | 0.0238095 | 0.185455 | 0.0147037 |
| 630 | 9 | 35740362 | 35740525 | GBA2 | 0.193457 | 0.546763 | 0.00181751 |
| 631 | 9 | 35750989 | 35751265 | RGP1 | 0.0113821 | 0.25 | 0.04 |
| 632 | 9 | 35751412 | 35751626 | RGP1 | 0.10219 | 0.615475 | 0.00828126 |
| 633 | 9 | 35793075 | 35793897 | NPR2 | 1 | 0.0571296 | 0.0196759 |
| 634 | 9 | 83974589 | 83975461 | HNRNPK | 0.0484699 | 0.144336 | 0.00077647 |
| 635 | 9 | 87639698 | 87639788 | DAPK1 | 0.133333 | 0.549296 | 0.0348361 |
| 636 | 9 | 87697204 | 87698655 | DAPK1 | 0.0243993 | 0.12216 | 0.0447191 |

| S/N | Chr | Start | End | Gene | IR ratio-Young | IR ratio-Old | *p*- value |
|---|---|---|---|---|---|---|---|
| 637 | X | 101254924 | 101255183 | DRP2 | 0.00387926 | 0.123125 | 0.0225713 |
| 638 | X | 101623893 | 101624157 | ARMCX3 | 0.00325785 | 0.115409 | 0.00841653 |
| 639 | X | 11120993 | 11121611 | HCCS | 0.0106827 | 0.2192 | 0.0170045 |
| 640 | X | 130139871 | 130140532 | AIFM1 | 0.0173014 | 0.235053 | 0.049556 |
| 641 | X | 153671190 | 153671284 | PNCK | 0.153377 | 0.246984 | 0.0498538 |
| 642 | X | 153671672 | 153671879 | PNCK | 0.157999 | 0.260668 | 0.0336027 |
| 643 | X | 153688836 | 153690374 | SLC6A8 | 0.0391877 | 0.159051 | 0.00932989 |
| 644 | X | 153790292 | 153790563 | IDH3G | 0.010166 | 0.121212 | 0.0127185 |
| 645 | X | 153798136 | 153798328 | SSR4 | 0.0201581 | 0.116111 | 0.0101307 |
| 646 | X | 153871179 | 153872151 | L1CAM | 0.0486063 | 0.161098 | 0.0401197 |
| 647 | X | 153932115 | 153932315 | NAA10 | 0.221291 | 0.441909 | 0.0018078 |
| 648 | X | 154380019 | 154380233 | EMD | 0.196907 | 0.399008 | 0.027291 |
| 649 | X | 154412214 | 154413206 | TAZ | 0.12402 | 0.43812 | 0.0347925 |
| 650 | X | 19354583 | 19355348 | PDHA1 | 0.117999 | 0.315769 | 0.00150976 |
| 651 | X | 48478168 | 48478448 | FTSJ1 | 0.029525 | 0.272539 | 0.0316764 |
| 652 | X | 48598037 | 48598716 | WDR13 | 0.131508 | 0.265087 | 0.0321983 |
| 653 | X | 48923746 | 48923850 | OTUD5 | 0.0219934 | 0.103041 | 0.0391659 |
| 654 | X | 49033540 | 49033725 | TFE3 | 0.0146396 | 0.146293 | 0.0243741 |
| 655 | X | 49282063 | 49285833 | PPP1R3F | 0.0385169 | 0.16988 | 0.0330987 |
| 656 | X | 54922291 | 54922577 | TRO | 0.410709 | 0.148206 | 0.0154452 |
| 657 | X | 70500989 | 70502162 | DLG3 | 0.0331263 | 0.166629 | 0.0323792 |
| 658 | X | 71298816 | 71299941 | NONO | 0.040637 | 0.121772 | 0.0479643 |
| 659 | X | 71544635 | 71547906 | OGT | 0.454747 | 0.756643 | 0.0136301 |

**Explanation**

**Start:** The genomic coordinates of the start of the retained intron.
**End:** The genomic coordinates of the end of the retained intron.
**Gene:** The gene that contains the differential retained intron.
