# Supplementary material for: Increased intron retention is a post‐transcriptional signature associated with progressive aging and Alzheimer’s disease
Source: Aging Cell. 2019 Mar 13;18(3):e12928. doi: 10.1111/acel.12928 (PMC6516162; doi:10.1111/acel.12928)
Supplement: Supplementary file 8 [file ACEL-18-e12928-s008.pdf]

Table S7: DAVID functional annotation chart of differential IR genes between Control & AD patients Cerebellum (Mayo Clinic: syn8612213)

S/N	GOTERM_BP_DIRECT	Count	%	*P-value*	Benjamini value
1	GO:0000398~mRNA splicing, via spliceosome	65	3.1538	1.66096E-13	7.39E-10
2	GO:0006468~protein phosphorylation	86	4.1727	5.99395E-07	0.001332467
3	GO:0043161~proteasome-mediated ubiquitin-dependent protein catabolic process	45	2.1834	6.96139E-06	0.010270669
4	GO:0031124~mRNA 3'-end processing	18	0.8734	1.26778E-05	0.014001968
5	GO:0006378~mRNA polyadenylation	13	0.6308	1.65229E-05	0.014594659
6	GO:0043547~positive regulation of GTPase activity	95	4.6094	2.27231E-05	0.016708234
7	GO:0036498~IRE1-mediated unfolded protein response	19	0.9219	3.79626E-05	0.02383964
8	GO:0006418~tRNA aminoacylation for protein translation	15	0.7278	5.08464E-05	0.027881578
9	GO:0006406~mRNA export from nucleus	26	1.2615	5.80989E-05	0.028312513
10	GO:0006396~RNA processing	25	1.2130	9.66562E-05	0.042092865
11	GO:0006369~termination of RNA polymerase II transcription	19	0.9219	0.000123673	0.048792798
12	GO:0006281~DNA repair	46	2.2319	0.00013551	0.049002296

Table S7: Pathway analysis of differential IR genes between Control & AD patients Cerebellum (Mayo Clinic: syn8612213)

S/N	Pathway name	Gene #	ID	*P-value*	FDR
1	mRNA surveillance pathway - Homo sapiens (human)	27	hsa03015	2.22E-06	6.74E-04
2	Spliceosome - Homo sapiens (human)	33	hsa03040	1.31E-05	1.99E-03
3	Aminoacyl-tRNA biosynthesis - Homo sapiens (human)	14	hsa00970	2.80E-04	2.18E-02
4	Lysosome - Homo sapiens (human)	28	hsa04142	2.87E-04	2.18E-02
5	Metabolic pathways - Homo sapiens (human)	182	hsa01100	4.26E-04	2.58E-02
6	Endocytosis - Homo sapiens (human)	48	hsa04144	5.91E-04	2.99E-02
7	AMPK signaling pathway - Homo sapiens (human)	27	hsa04152	7.69E-04	2.99E-02
8	Lysine degradation - Homo sapiens (human)	16	hsa00310	7.90E-04	2.99E-02
9	RNA degradation - Homo sapiens (human)	19	hsa03018	9.48E-04	3.19E-02
10	Glucagon signaling pathway - Homo sapiens (human)	23	hsa04922	1.29E-03	3.56E-02

Table S7: Overlap of differential IR genes with curated AD genes (DisGeNET)

DVL1	WNK1	ELAC2	Sep-02	FLOT1	CCDC22
SPEN	NCAPD2	VTN	PTPRA	HLA-B	ZMYM3
CDC42	PTMS	PHF12	CDC25B	DDX39B	TAF1
FABP3	P3H3	RARA	PYGB	NELFE	FLNA
SFPQ	ENO2	COASY	HM13	DXO	MRE11
PABPC4	PHB2	NPEPPS	TRPC4AP	TNXB	CD68
AKR1A1	RAPGEF3	COG1	CD40	AGER	CAPN10
FAAH	AAAS	GGA3	RRP1	TAP2	ATAT1
NRDC	HNRNPA1	HGS	C21orf2	COL11A2	WDR45
CLK2	LRP1	FASN	PCNT	RXRB	IL18BP
LMNA	CDK4	CSNK1D	GGA1	PHF1	GAB2
NCSTN	ACACB	MBP	PLA2G6	PPARD	MED17
POU2F1	ATXN2	PTBP1	SGSM3	TFEB	PLD2
COG2	MAPKAPK5	ABCA7	ACO2	TTBK1	DLG4
AGT	PSMD9	SBNO2	SREBF2	GSTA4	ACADVL
BMS1	HSPH1	STK11	LMF2	HNRNPA2B1	BCS1L
UBE2D1	LAMP1	APC2	NCAPH2	NT5C3A	ABCB6
VPS26A	NDRG2	SPPL2B	CPT1B	MLXIPL	GPC1
HPS1	TGM1	APBA3	ARSA	ELN	RANBP9
NDUFB8	MTHFD1	MCOLN1	SHANK3	AKAP9	PGBD1
MGEA5	DLST	DNMT1	OGG1	ZCWPW1	GABBR1
ADD3	MOK	ZGLP1	RAF1	FIS1	ABCA2
MTG1	CKB	TYK2	CTNNB1	LRRC4	EHMT1
IRF7	AKT1	DNM2	PTH1R	GSTK1	HDAC6
BRSK2	JAG2	NFIX	SCAP	PAXIP1	GRK2
APBB1	EIF2AK4	CRTC1	APEH	BMP1	RPS6KB2
ILK	RMDN3	APLP1	PCBP4	PTK2B	CTTN
ABCC8	TYRO3	SIRT2	CPOX	FGFR1	APRT
PAX6	CSK	XRCC1	CD47	IKBKB	SPG7
NR1H3	CIB1	TOMM40	TF	POLB	ARRB2
MADD	SYNM	PPP1R15A	ECE2	RMDN1	PRKRA
NXF1	TSC2	BAX	IDUA	HSF1	CLK1
MARK2	CREBBP	RRAS	CRMP1	DGAT1	ALS2
SYVN1	NUBP1	PRPF31	HADH	SIGMAR1	ANXA6
CAPN1	EEF2K	TIA1	PPID	FANCG	SFXN1
RELA	TAOK2	HTRA2	CCNB1	SLC2A8	NQO2
KAT5	NAE1	BIN1	LMNB1	CIZ1	GOLGA2
PC	MPHOSPH6	UBXN4	APBB3	DNM1	PTPA
					PTGDS

Table S7: Differential IR between Control & AD patients Cerebellum (Mayo Clinic: syn8612213)

S/N	Gene	Ensembl ID	Position of retained intron	p-value	AD_IR_average	Cont_IR_average
1	NOC2L	ENSG00000188976	1:944800-945056:-	2.17E-06	0.069969558	0.044867947
2	NOC2L	ENSG00000188976	1:954082-955922:-	0.04010844	0.086704762	0.111062871
3	KLHL17	ENSG00000187961	1:964008-964106:+	0.011481	0.146047991	0.180556084
4	SCNN1D	ENSG00000162572	1:1282315-1283977:+	0.00149371	0.160404239	0.103515534
5	SCNN1D	ENSG00000162572	1:1287596-1287672:+	0.00258349	0.246711409	0.19542197
6	SCNN1D	ENSG00000162572	1:1287836-1287938:+	0.00209705	0.116815046	0.084468727
7	ACAP3	ENSG00000131584	1:1294816-1295446:-	0.04501617	0.105588785	0.082954675
8	ACAP3	ENSG00000131584	1:1298679-1299344:-	0.03690758	0.104151913	0.122266581
9	ACAP3	ENSG00000131584	1:1299356-1299830:-	0.03938376	0.20988317	0.180043603
10	ACAP3	ENSG00000131584	1:1299905-1299972:-	0.00026566	0.15843722	0.1044191
11	ACAP3	ENSG00000131584	1:1300068-1300157:-	0.00176799	0.232639284	0.178183627
12	ACAP3	ENSG00000131584	1:1300202-1300508:-	0.00285782	0.225713323	0.174801492
13	ACAP3	ENSG00000131584	1:1300692-1301987:-	0.03123215	0.153205523	0.132263097
14	ACAP3	ENSG00000131584	1:1304143-1307768:-	0.02702963	0.719096951	0.738183117
15	PUSL1	ENSG00000169972	1:1309603-1309680:+	0.03582142	0.28975947	0.236491645
16	PUSL1	ENSG00000169972	1:1309851-1310633:+	0.00123268	0.250095007	0.187164795
17	PUSL1	ENSG00000169972	1:1310688-1310908:+	0.00868759	0.171615662	0.128738203
18	PUSL1	ENSG00000169972	1:1311071-1311329:+	0.00072334	0.12360345	0.086717193
19	CPSF3L	ENSG00000127054	1:1314962-1315403:-	0.01780933	0.52038828	0.468730039
20	CPSF3L	ENSG00000127054	1:1315438-1315519:-	0.01786511	0.49116189	0.444574753
21	DVL1	ENSG00000107404	1:1341805-1342052:-	0.00063431	0.134292728	0.103785274
22	CCNL2	ENSG00000221978	1:1388743-1390229:-	2.04E-07	0.056647872	0.036431844
23	ATAD3B	ENSG00000160072	1:1482614-1485015:+	0.00885205	0.3469245	0.300333584
24	ATAD3B	ENSG00000160072	1:1489274-1490256:+	2.88E-09	0.265254517	0.364342909
25	ATAD3A	ENSG00000197785	1:1522899-1523510:+	0.00208806	0.099181278	0.068697683
26	ATAD3A	ENSG00000197785	1:1525291-1526460:+	0.01530906	0.101964327	0.074457598
27	ATAD3A	ENSG00000197785	1:1527862-1529222:+	0.0054325	0.424230317	0.372704286
28	ATAD3A	ENSG00000197785	1:1529331-1533925:+	0.00153584	0.157981193	0.120432513
29	MIB2	ENSG00000197530	1:1616614-1623388:+	0.03005089	0.196362671	0.204107129
30	MIB2	ENSG00000197530	1:1625428-1625545:+	0.00966094	0.100909707	0.078467595
31	MIB2	ENSG00000197530	1:1627444-1627672:+	0.00818602	0.353272585	0.291603052
32	MIB2	ENSG00000197530	1:1628179-1628272:+	0.04272041	0.297847622	0.248002155
33	NADK	ENSG00000008130	1:1753060-1753566:-	0.00068233	0.097680306	0.071495842
34	NADK	ENSG00000008130	1:1753649-1754050:-	0.00284198	0.233496666	0.207707955
35	SKI	ENSG00000157933	1:2229735-2302977:+	0.01829734	0.071858801	0.095672988
36	MORN1	ENSG00000116151	1:2324143-2336468:-	0.04238599	0.213608627	0.147236794
37	MORN1	ENSG00000116151	1:2358715-2372480:-	0.00407114	0.147778948	0.11801404
38	PLCH2	ENSG00000149527	1:2491335-2494855:+	0.03674837	0.08058638	0.099941323
39	PLCH2	ENSG00000149527	1:2499720-2502111:+	7.87E-05	0.06791918	0.090332781
40	PANK4	ENSG00000157881	1:2521315-2521717:-	0.00068428	0.068151192	0.108366879
41	WRAP73	ENSG00000116213	1:3636030-3636994:-	0.03000288	0.264397001	0.314598117
42	CEP104	ENSG00000116198	1:3823562-3825757:-	0.02874476	0.107949974	0.090823928
43	NPHP4	ENSG00000131697	1:5863405-5863889:-	0.02134309	0.391936256	0.345039026
44	PLEKHG5	ENSG00000171680	1:6467572-6467824:-	3.80E-08	0.322391027	0.155017314
45	PLEKHG5	ENSG00000171680	1:6468586-6469041:-	0.00166145	0.208878263	0.164146961
46	DNAJC11	ENSG00000007923	1:6637340-6637446:-	0.01903267	0.102867917	0.08931041
47	VAMP3	ENSG00000049245	1:7771385-7773441:+	0.00151749	0.348581024	0.270559026
48	RERE	ENSG00000142599	1:8364838-8365811:-	0.03009122	0.108948857	0.14860736
49	UBE4B	ENSG00000130939	1:10161286-10168135:+	0.02637975	0.13370675	0.118127161
50	PEX14	ENSG00000142655	1:10623121-10624339:+	0.02037952	0.11688526	0.10169699
51	PEX14	ENSG00000142655	1:10624437-10627271:+	0.00139447	0.105952776	0.082281742
52	PEX14	ENSG00000142655	1:10627363-10629530:+	4.96E-05	0.116527306	0.087897268
53	SRM	ENSG00000116649	1:11055084-11055780:-	0.00263127	0.112627623	0.086128553
54	EXOSC10	ENSG00000171824	1:11077443-11077600:-	0.00930762	0.663224927	0.630862519
55	FBXO6	ENSG00000116663	1:11668944-11671265:+	0.00107784	0.355789402	0.290669832
56	FBXO6	ENSG00000116663	1:11672023-11673276:+	0.00311907	0.390958207	0.328237799

S/N	Gene	Ensembl ID	Position of retained intron	p-value	AD_IR_average	Cont_IR_average
57	MAD2L2	ENSG00000116670	1:11680613-11681038:-	4.00E-06	0.194162444	0.123726573
58	KAZN	ENSG00000189337	1:15101774-15103358:+	0.01209683	0.205621499	0.172293018
59	KAZN	ENSG00000189337	1:15103460-15104022:+	0.0001857	0.173661455	0.123001209
60	PLEKHM2	ENSG00000116786	1:15717992-15718537:+	2.69E-06	0.103085715	0.066722697
61	PLEKHM2	ENSG00000116786	1:15728357-15728668:+	0.00011291	0.142015938	0.106132135
62	PLEKHM2	ENSG00000116786	1:15728733-15729101:+	0.00028672	0.119561062	0.089323848
63	SPEN	ENSG00000065526	1:15938006-15938717:+	0.02767209	0.150111082	0.130739382
64	ZBTB17	ENSG00000116809	1:15943895-15944299:-	0.03917124	0.166590259	0.190916214
65	NECAP2	ENSG00000157191	1:16455893-16458841:+	0.01285782	0.326966951	0.377973639
66	CROCCP3	ENSG00000080947	1:16484464-16485568:-	0.00996199	0.036428834	0.040590994
67	CROCCP2	ENSG00000215908	1:16631109-16631529:-	0.00014672	0.186972898	0.134202077
68	CROCC	ENSG00000058453	1:16961130-16965722:+	0.03629107	0.103469255	0.119284929
69	ATP13A2	ENSG00000159363	1:16986632-16986804:-	0.00575673	0.225253135	0.171424179
70	ATP13A2	ENSG00000159363	1:16992581-16993628:-	0.00140337	0.200908929	0.162681609
71	ATP13A2	ENSG00000159363	1:16993835-16995975:-	0.00088402	0.412125695	0.356833104
72	ATP13A2	ENSG00000159363	1:17002103-17002295:-	0.00734954	0.13087665	0.115528683
73	ARHGEF10	ENSG00000074964	1:17638689-17640201:+	0.00297857	0.118317	0.10337893
74	ARHGEF10	ENSG00000074964	1:17687747-17695157:+	0.01200489	0.109735623	0.089437831
75	ALDH4A1	ENSG00000159423	1:18874581-18875381:-	0.0003876	0.119691372	0.086397103
76	ALDH4A1	ENSG00000159423	1:18879373-18881699:-	0.00101406	0.105085966	0.070077994
77	ALDH4A1	ENSG00000159423	1:18881887-18883123:-	0.00145363	0.108093017	0.068298863
78	MRTO4	ENSG00000053372	1:19257145-19257453:+	0.00586063	0.131402812	0.103063932
79	CAPZB	ENSG00000077549	1:19339617-19342751:-	3.64E-06	0.104909554	0.098720839
80	CAPZB	ENSG00000077549	1:19342864-19344357:-	1.86E-06	0.082357502	0.073535557
81	CDC42	ENSG00000070831	1:22086866-22089942:+	0.04358545	0.051136663	0.042821842
82	LYPLA2	ENSG00000011009	1:23793930-23794062:+	0.01272738	0.19321068	0.155785783
83	GALE	ENSG00000117308	1:23796265-23796508:-	0.01504272	0.170193483	0.133913412
84	SRSF10	ENSG00000188529	1:23971439-23971572:-	0.00167681	0.067437189	0.048290258
85	TMEM57	ENSG00000204178	1:25491584-25498263:+	1.66E-06	0.212486017	0.148730973
86	LDLRAP1	ENSG00000157978	1:25543786-25553921:+	0.00017798	0.287626015	0.211408477
87	LDLRAP1	ENSG00000157978	1:25565207-25566847:+	0.02230337	0.44270201	0.377363909
88	MAN1C1	ENSG00000117643	1:25778324-25780939:+	1.34E-05	0.257042576	0.185975987
89	SELENON	ENSG00000162430	1:25809820-25811453:+	0.01972988	0.14157665	0.120103797
90	SELENON	ENSG00000162430	1:25813993-25814076:+	0.00588754	0.100016874	0.078483968
91	SELENON	ENSG00000162430	1:25814178-25815547:+	7.93E-06	0.15990623	0.115733135
92	CNKSR1	ENSG00000142675	1:26188503-26188597:+	0.01594751	0.131497004	0.100283958
93	UBXN11	ENSG00000158062	1:26284482-26285463:-	0.01448225	0.357254098	0.429556857
94	DHDDS	ENSG00000117682	1:26438284-26442730:+	0.0165611	0.158200002	0.20477464
95	RPS6KA1	ENSG00000117676	1:26554251-26554595:+	0.00050388	0.100146017	0.076468139
96	RPS6KA1	ENSG00000117676	1:26561134-26561504:+	0.00350463	0.10184327	0.083482645
97	PIGV	ENSG00000060642	1:26795234-26797562:+	0.00712333	0.187036929	0.225563234
98	SLC9A1	ENSG00000090020	1:27102130-27102384:-	0.00289418	0.103890888	0.075502341
99	SLC9A1	ENSG00000090020	1:27102558-27102672:-	0.03601171	0.131260365	0.110693349
100	SLC9A1	ENSG00000090020	1:27102743-27103222:-	0.01447308	0.152390861	0.125073491
101	SLC9A1	ENSG00000090020	1:27103312-27105884:-	0.03871468	0.175436985	0.149211661
102	TMEM222	ENSG00000186501	1:27322391-27330719:+	0.00012587	0.100689276	0.072748119
103	TRNAU1AF	ENSG00000180098	1:28571900-28577499:+	1.51E-11	0.189546909	0.132188104
104	PTPRU	ENSG00000060656	1:29317921-29320684:+	0.00047166	0.235404934	0.175487729
105	PTPRU	ENSG00000060656	1:29320825-29323370:+	6.67E-06	0.228657065	0.161426596
106	SNRNP40	ENSG00000060688	1:31261632-31267870:-	0.03032199	0.170107433	0.140035339
107	FABP3	ENSG00000121769	1:31365939-31367392:-	1.23E-13	0.428318691	0.244621488
108	FABP3	ENSG00000121769	1:31367494-31369384:-	8.22E-11	0.41810572	0.256843191
109	FABP3	ENSG00000121769	1:31369557-31372941:-	2.79E-07	0.266983239	0.183843109
110	COL16A1	ENSG00000084636	1:31654043-31654791:-	2.44E-06	0.106666985	0.061990451
111	ADGRB2	ENSG00000121753	1:31727605-31728024:-	9.15E-06	0.183396909	0.146679927
112	ADGRB2	ENSG00000121753	1:31728081-31728181:-	8.74E-07	0.060313899	0.041467035
113	ADGRB2	ENSG00000121753	1:31728277-31728597:-	0.01636001	0.04151748	0.030310965

S/N	Gene	Ensembl ID	Position of retained intron	p-value	AD_IR_average	Cont_IR_average
114	ADGRB2	ENSG00000121753	1:31736390-31736572:-	0.00021375	0.265373576	0.220363203
115	ADGRB2	ENSG00000121753	1:31736723-31737428:-	0.00106053	0.191068306	0.157827984
116	ADGRB2	ENSG00000121753	1:31740034-31740109:-	0.02050504	0.104740598	0.082544344
117	ADGRB2	ENSG00000121753	1:31740541-31741372:-	0.00206799	0.18097001	0.141490394
118	TMEM39B	ENSG00000121775	1:32094971-32100441:+	6.90E-05	0.180099544	0.1339788
119	TXLNA	ENSG00000084652	1:32192430-32192656:+	0.00189242	0.224070015	0.188834257
120	CCDC28B	ENSG00000160050	1:32204379-32204597:+	0.01288251	0.150076299	0.122055578
121	BSDC1	ENSG00000160058	1:32366654-32368446:-	4.88E-05	0.321840621	0.249378319
122	ZBTB8OS	ENSG00000176261	1:32627544-32631826:-	0.00583954	0.041302537	0.036204355
123	SFPQ	ENSG00000116560	1:35176471-35177318:-	1.56E-05	0.050615071	0.035133515
124	NCDN	ENSG00000020129	1:35563909-35565226:+	0.00375953	0.285040191	0.239268726
125	TRAPPC3	ENSG00000054116	1:36137322-36137795:-	5.06E-06	0.122278438	0.169886203
126	MAP7D1	ENSG00000116871	1:36179315-36179511:+	0.01954355	0.021440798	0.019287101
127	DNALI1	ENSG00000163879	1:37562245-37565025:+	2.46E-07	0.172394002	0.092209355
128	MACF1	ENSG00000127603	1:39284185-39284332:+	0.00040939	0.077188407	0.123687576
129	PABPC4	ENSG00000090621	1:39563741-39564422:-	0.00346166	0.18816325	0.182740596
130	TRIT1	ENSG00000043514	1:39844640-39847219:-	0.00763651	0.12701372	0.103355852
131	TRIT1	ENSG00000043514	1:39847297-39847547:-	0.00152653	0.140949655	0.101016477
132	PPIH	ENSG00000171960	1:42667440-42676583:+	0.030379	0.194646516	0.17652519
133	P3H1	ENSG00000117385	1:42747412-42747722:-	0.02509703	0.207923356	0.179745245
134	P3H1	ENSG00000117385	1:42747798-42748199:-	0.00042621	0.13638679	0.104208887
135	P3H1	ENSG00000117385	1:42748317-42750185:-	0.00031424	0.247314628	0.200459574
136	SZT2	ENSG00000198198	1:43442368-43442441:+	0.0089629	0.103359657	0.070159669
137	HYI	ENSG00000178922	1:43451544-43451647:-	1.42E-06	0.177740599	0.133456979
138	HYI	ENSG00000178922	1:43451717-43451797:-	2.50E-06	0.208794015	0.160827683
139	HYI	ENSG00000178922	1:43451847-43451934:-	2.74E-05	0.218993291	0.172923313
140	HYI	ENSG00000178922	1:43452013-43452204:-	0.00639861	0.292115305	0.253603595
141	HYI	ENSG00000178922	1:43453497-43453594:-	0.03030705	0.28082887	0.241627088
142	IPO13	ENSG00000117408	1:43958268-43958460:+	0.00183826	0.142973302	0.111698284
143	IPO13	ENSG00000117408	1:43958889-43960248:+	1.27E-05	0.183450518	0.127865208
144	IPO13	ENSG00000117408	1:43961262-43964268:+	0.00035603	0.107332402	0.081337544
145	IPO13	ENSG00000117408	1:43964321-43966574:+	0.03082101	0.140038206	0.122918752
146	IPO13	ENSG00000117408	1:43966782-43966929:+	0.00029678	0.109295639	0.083036313
147	CCDC24	ENSG00000159214	1:43993964-43995107:+	0.01877678	0.287123854	0.2461815
148	SLC6A9	ENSG00000196517	1:43997739-43997854:-	0.00036595	0.18767901	0.128221819
149	ERI3	ENSG00000117419	1:44352925-44354891:-	0.04080151	0.143471743	0.167286358
150	RNF220	ENSG00000187147	1:44649957-44650703:+	2.02E-05	0.177476449	0.119793813
151	HECTD3	ENSG00000126107	1:45003936-45004059:-	0.0181334	0.100943294	0.082392406
152	HECTD3	ENSG00000126107	1:45005883-45005996:-	0.00516052	0.164896098	0.214351856
153	MUTYH	ENSG00000132781	1:45330557-45331181:-	0.00212739	0.182940278	0.154582883
154	MUTYH	ENSG00000132781	1:45331849-45332022:-	0.00060597	0.504290488	0.433016039
155	MUTYH	ENSG00000132781	1:45332086-45332165:-	0.00193834	0.160938426	0.127645292
156	MUTYH	ENSG00000132781	1:45332310-45332390:-	0.00309262	0.346231634	0.297427597
157	MUTYH	ENSG00000132781	1:45332488-45332573:-	0.00484248	0.384167671	0.325434026
158	MUTYH	ENSG00000132781	1:45332687-45332762:-	0.01077193	0.301566073	0.25854891
159	AKR1A1	ENSG00000117448	1:45569229-45569890:+	1.78E-06	0.156680228	0.10099376
160	POMGNT1	ENSG00000085998	1:46196849-46196969:-	0.03406445	0.121489399	0.102558543
161	LRRC41	ENSG00000132128	1:46279257-46279491:-	0.00030204	0.261021178	0.21008826
162	LRRC41	ENSG00000132128	1:46279614-46280191:-	2.62E-06	0.15287784	0.106563896
163	LRRC41	ENSG00000132128	1:46280290-46280395:-	3.85E-07	0.155732907	0.097392524
164	LRRC41	ENSG00000132128	1:46280560-46281124:-	0.04933094	0.198795905	0.164889765
165	FAAH	ENSG00000117480	1:46409198-46410397:+	4.68E-06	0.384505634	0.312552597
166	MKNK1	ENSG00000079277	1:46561642-46562648:-	0.00018267	0.20602128	0.159156525
167	NRDC	ENSG00000078618	1:51789657-51790532:-	0.00016184	0.144806824	0.115184443
168	NRDC	ENSG00000078618	1:51814813-51816311:-	0.03021904	0.160461946	0.145780129
169	ZCCHC11	ENSG00000134744	1:52423913-52425348:-	1.09E-05	0.048483061	0.037045265
170	ECHDC2	ENSG00000121310	1:52896597-52897436:-	0.0029192	0.095015961	0.076079718

S/N	Gene	Ensembl ID	Position of retained intron	p-value	AD_IR_average	Cont_IR_average
171	ECHDC2	ENSG00000121310	1:52897484-52899173:-	5.55E-14	0.301336322	0.174944317
172	ECHDC2	ENSG00000121310	1:52904833-52905033:-	2.82E-05	0.576429341	0.500839091
173	C1orf123	ENSG00000162384	1:53220344-53220569:-	1.97E-06	0.372796829	0.294746468
174	MRPL37	ENSG00000116221	1:54216344-54218171:+	0.00339632	0.207923933	0.176668083
175	MROH7	ENSG00000184313	1:54706537-54709013:+	0.00131742	0.140224595	0.103219962
176	TM2D1	ENSG00000162604	1:61681350-61683416:-	1.17E-08	0.43428911	0.316010688
177	TM2D1	ENSG00000162604	1:61683546-61686791:-	0.00082716	0.031668031	0.01911106
178	ALG6	ENSG00000088035	1:63419440-63428732:+	0.02184927	0.139395083	0.105507047
179	ITGB3BP	ENSG00000142856	1:63441103-63446805:-	0.0084221	0.158511204	0.135659583
180	EFCAB7	ENSG00000203965	1:63568519-63571020:+	0.0001269	0.163052959	0.115913475
181	EFCAB7	ENSG00000203965	1:63571128-63572441:+	1.58E-06	0.232109593	0.168822992
182	SRSF11	ENSG00000116754	1:70221839-70228421:+	0.00831464	0.067450958	0.087647866
183	ACADM	ENSG00000117054	1:75761370-75762691:+	1.38E-06	0.23569723	0.14992654
184	FUBP1	ENSG00000162613	1:77944269-77945505:-	8.41E-05	0.853286817	0.785527857
185	FUBP1	ENSG00000162613	1:77946576-77947452:-	0.00014209	0.845316488	0.746363156
186	RNPC3	ENSG00000185946	1:103546342-103546976:+	0.00855926	0.092549499	0.066542374
187	CLCC1	ENSG00000121940	1:108934942-108937076:-	0.00052949	0.215569984	0.162496543
188	SARS	ENSG00000031698	1:109236548-109237243:+	0.01328319	0.079211766	0.093175726
189	PSRC1	ENSG00000134222	1:109281251-109281618:-	0.03078009	0.21849219	0.261372313
190	AMPD2	ENSG00000116337	1:109628277-109628363:+	0.02644057	0.129597568	0.112226795
191	GSTM4	ENSG00000168765	1:109656787-109657214:+	4.04E-07	0.139263549	0.095436636
192	GSTM4	ENSG00000168765	1:109657671-109657771:+	8.16E-07	0.125500634	0.087377683
193	GSTM4	ENSG00000168765	1:109659110-109661164:+	8.20E-05	0.082081978	0.068389054
194	AHCYL1	ENSG00000168710	1:110019626-110020730:+	0.01920178	0.100912844	0.075858324
195	AHCYL1	ENSG00000168710	1:110020851-110021673:+	0.04909099	0.129228883	0.116584575
196	STRIP1	ENSG00000143093	1:110045078-110046679:+	0.01688396	0.102875302	0.087344456
197	STRIP1	ENSG00000143093	1:110047869-110049111:+	0.00963216	0.240346434	0.213593365
198	STRIP1	ENSG00000143093	1:110049238-110049459:+	0.00856862	0.25748342	0.208773613
199	STRIP1	ENSG00000143093	1:110049560-110050342:+	0.00015838	0.169100372	0.131556822
200	PHTF1	ENSG00000116793	1:113700949-113704080:-	2.18E-06	0.134580054	0.090790696
201	AP4B1	ENSG00000134262	1:113896465-113897839:-	0.00387266	0.087429061	0.071160909
202	AP4B1	ENSG00000134262	1:113897943-113898717:-	0.00851608	0.132257922	0.104341528
203	SIKE1	ENSG00000052723	1:114779284-114780109:-	0.00791676	0.084253794	0.060157543
204	POLR3C	ENSG00000186141	1:145839991-145840115:+	0.00742892	0.100486751	0.083970061
205	NUDT17	ENSG00000186364	1:145847348-145847582:+	0.03236542	0.259554715	0.214927897
206	PEX11B	ENSG00000131779	1:145912566-145916816:-	0.00451101	0.070105722	0.10527107
207	POLR3GL	ENSG00000121851	1:145977851-145977982:+	0.02371665	0.144819663	0.171446617
208	SF3B4	ENSG00000143368	1:149924014-149925835:-	0.00700823	0.0954065	0.077623473
209	VPS45	ENSG00000136631	1:150077231-150077668:+	0.01952423	0.166305428	0.216466288
210	CIART	ENSG00000159208	1:150284708-150286429:+	0.04187598	0.100501207	0.080765239
211	PRPF3	ENSG00000117360	1:150346136-150346407:+	7.34E-05	0.380855744	0.312717909
212	PRPF3	ENSG00000117360	1:150346491-150349156:+	8.38E-10	0.228386832	0.160219036
213	TARS2	ENSG00000143374	1:150491511-150491597:+	0.00342863	0.149539771	0.201402613
214	TARS2	ENSG00000143374	1:150504733-150504905:+	0.00899215	0.104358342	0.128678781
215	SETDB1	ENSG00000143379	1:150963139-150963529:+	0.0217818	0.342909659	0.307981753
216	SETDB1	ENSG00000143379	1:150964083-150964246:+	0.00297905	0.118145524	0.093386065
217	CERS2	ENSG00000143418	1:150966862-150967073:-	0.00192857	0.148930133	0.123919378
218	CERS2	ENSG00000143418	1:150967202-150967391:-	0.00340889	0.195771171	0.164900506
219	CERS2	ENSG00000143418	1:150967484-150967663:-	0.04422473	0.283410239	0.26243813
220	CERS2	ENSG00000143418	1:150967714-150967819:-	0.00049485	0.107876194	0.082424027
221	CERS2	ENSG00000143418	1:150967877-150968082:-	0.00222804	0.092497172	0.07841533
222	FAM63A	ENSG00000143409	1:150997367-150997623:-	2.35E-05	0.459906585	0.370824377
223	FAM63A	ENSG00000143409	1:150997779-150998081:-	0.01025552	0.087929038	0.110720419
224	SEMA6C	ENSG00000143434	1:151138406-151138629:-	0.02565867	0.165965659	0.157312908
225	SEMA6C	ENSG00000143434	1:151138731-151139424:-	0.00119674	0.156235754	0.134363245
226	PSMB4	ENSG00000159377	1:151400588-151400763:+	0.03820446	0.19820894	0.171159252
227	PSMB4	ENSG00000159377	1:151401355-151401541:+	4.24E-06	0.20687279	0.148456299

S/N	Gene	Ensembl ID	Position of retained intron	p-value	AD_IR_average	Cont_IR_average
228	POGZ	ENSG00000143442	1:151424286-151424954:-	0.0179528	0.198603943	0.165466219
229	MRPL9	ENSG00000143436	1:151761552-151762104:-	0.00618755	0.10822347	0.133824949
230	SNAPIN	ENSG00000143553	1:153658886-153659137:+	0.04399106	0.218218091	0.203664781
231	ILF2	ENSG00000143621	1:153662795-153663018:-	0.0400226	0.116693055	0.135077905
232	INTS3	ENSG00000143624	1:153760390-153760826:+	0.01363584	0.178104856	0.151536321
233	INTS3	ENSG00000143624	1:153772440-153772638:+	2.34E-07	0.237784011	0.168411335
234	INTS3	ENSG00000143624	1:153772711-153772924:+	3.71E-06	0.089459471	0.061284771
235	AL513523.	ENSG00000279767	1:153772711-153772924:-	3.71E-06	0.089459471	0.061284771
236	DENND4B	ENSG00000198837	1:153939804-153940155:-	0.00210682	0.472876463	0.407797104
237	DENND4B	ENSG00000198837	1:153941048-153941230:-	0.00820881	0.439771573	0.381282104
238	CRTC2	ENSG00000160741	1:153949384-153951259:-	0.00014545	0.135533745	0.111714865
239	CRTC2	ENSG00000160741	1:153951666-153952017:-	0.00031628	0.243722823	0.206586662
240	CRTC2	ENSG00000160741	1:153953369-153953537:-	0.03916695	0.166630372	0.143066468
241	CRTC2	ENSG00000160741	1:153953606-153954254:-	0.01414095	0.134838056	0.114149734
242	CREB3L4	ENSG00000143578	1:153973716-153973871:+	8.77E-06	0.141062235	0.07231427
243	JTB	ENSG00000143543	1:153977013-153977169:-	0.03912753	0.08741741	0.106937222
244	TPM3	ENSG00000143549	1:154169383-154170399:-	0.00158141	0.021781906	0.020486224
245	C1orf43	ENSG00000143612	1:154212624-154214456:-	5.69E-05	0.01503615	0.01984215
246	UBAP2L	ENSG00000143569	1:154260029-154260891:+	0.00014125	0.159850987	0.122504905
247	ATP8B2	ENSG00000143515	1:154345545-154345799:+	0.02506876	0.199560439	0.237118286
248	SHC1	ENSG00000160691	1:154967797-154967979:-	0.00749491	0.180970556	0.139784671
249	FLAD1	ENSG00000160688	1:154988849-154989559:+	0.02004197	0.113785823	0.095773496
250	FLAD1	ENSG00000160688	1:154989707-154990158:+	0.00233088	0.099009715	0.075608869
251	MTX1	ENSG00000173171	1:155212219-155212384:+	0.00397109	0.264974694	0.216152327
252	FAM189B	ENSG00000160767	1:155250821-155251094:-	0.00323481	0.134398472	0.103592008
253	FAM189B	ENSG00000160767	1:155251164-155251501:-	0.00065565	0.279409122	0.229655642
254	FAM189B	ENSG00000160767	1:155251601-155251732:-	0.04178193	0.297041963	0.272200661
255	SCAMP3	ENSG00000116521	1:155256419-155256673:-	4.35E-06	0.274630939	0.201343768
256	CLK2	ENSG00000176444	1:155263400-155263949:-	0.01121081	0.15357344	0.138890713
257	CLK2	ENSG00000176444	1:155264300-155264467:-	0.02722905	0.192448745	0.166548826
258	CLK2	ENSG00000176444	1:155265954-155266728:-	0.01196334	0.216298909	0.184749418
259	RUSC1	ENSG00000160753	1:155325178-155325315:+	0.01924625	0.354277707	0.304746539
260	YY1AP1	ENSG00000163374	1:155660913-155661306:-	4.75E-05	0.142107163	0.189365004
261	DAP3	ENSG00000132676	1:155732033-155736945:+	0.00026516	0.151834804	0.117910464
262	GON4L	ENSG00000116580	1:155750733-155751766:-	6.28E-06	0.454221683	0.538331675
263	GON4L	ENSG00000116580	1:155752587-155753203:-	8.28E-05	0.066146152	0.104206224
264	GON4L	ENSG00000116580	1:155753414-155754374:-	0.00011708	0.066582528	0.108054331
265	KIAA0907	ENSG00000132680	1:155914320-155915872:-	0.03816359	0.286296488	0.336582221
266	KIAA0907	ENSG00000132680	1:155916737-155917498:-	8.20E-05	0.154516378	0.188542925
267	ARHGEF2	ENSG00000116584	1:155952235-155952627:-	0.00137854	0.099179667	0.082084865
268	LMNA	ENSG00000160789	1:156138757-156139079:+	0.04671165	0.230782051	0.205051371
269	PMF1	ENSG00000160783	1:156236483-156239547:+	0.0425026	0.053731485	0.052183561
270	PAQR6	ENSG00000160781	1:156244403-156244689:-	0.03872218	0.019164617	0.012628374
271	SMG5	ENSG00000198952	1:156250996-156251402:-	1.53E-10	0.388211988	0.285965338
272	C1orf61	ENSG00000125462	1:156407197-156407841:-	0.02772105	0.327715244	0.275451104
273	C1orf61	ENSG00000125462	1:156414753-156421554:-	0.01393783	0.047610156	0.03932137
274	HAPLN2	ENSG00000132702	1:156624467-156624600:+	0.00099998	0.231417907	0.174570523
275	HAPLN2	ENSG00000132702	1:156624783-156625100:+	0.00651344	0.295911056	0.229435384
276	ARHGEF11	ENSG00000132694	1:156946162-156946661:-	0.0001853	0.09403386	0.064448058
277	IGSF8	ENSG00000162729	1:160091608-160091807:-	1.47E-09	0.456467561	0.362573335
278	IGSF8	ENSG00000162729	1:160091938-160092281:-	8.54E-08	0.430076073	0.345161768
279	IGSF8	ENSG00000162729	1:160094171-160094868:-	0.04267364	0.301931902	0.330315468
280	IGSF8	ENSG00000162729	1:160095246-160098408:-	0.0110518	0.119151602	0.139752262
281	NCSTN	ENSG00000162736	1:160355757-160355862:+	0.00047912	0.131790938	0.100401292
282	NIT1	ENSG00000158793	1:161119612-161119818:+	0.03386272	0.127215577	0.101410996
283	USP21	ENSG00000143258	1:161162726-161162918:+	0.01199466	0.381074659	0.341163078
284	USP21	ENSG00000143258	1:161163074-161163554:+	2.01E-05	0.202232462	0.137396343

S/N	Gene	Ensembl ID	Position of retained intron	p-value	AD_IR_average	Cont_IR_average
285	USP21	ENSG00000143258	1:161163619-161163877:+	9.27E-05	0.346139573	0.266596423
286	USP21	ENSG00000143258	1:161164612-161164834:+	0.00090007	0.46744378	0.397422792
287	PPOX	ENSG00000143224	1:161170769-161170906:+	0.01564131	0.241437098	0.208024571
288	PPOX	ENSG00000143224	1:161170949-161171033:+	0.00184591	0.403888488	0.327216117
289	B4GALT3	ENSG00000158850	1:161172331-161173604:-	0.03727074	0.263466988	0.237625818
290	NDUFS2	ENSG00000158864	1:161213921-161214155:+	0.00094558	0.485455537	0.409476403
291	UCK2	ENSG00000143179	1:165896332-165903181:+	0.00326985	0.181712938	0.14002144
292	POU2F1	ENSG00000143190	1:167412304-167413025:+	0.01401267	0.101526037	0.082571381
293	C1orf112	ENSG00000000460	1:169851936-169852789:+	0.00141735	0.174778534	0.207773399
294	PRRC2C	ENSG00000117523	1:171587221-171587647:+	0.00849635	0.092772195	0.074473847
295	MRPS14	ENSG00000120333	1:175014851-175018417:-	2.52E-05	0.108709037	0.062435378
296	MRPS14	ENSG00000120333	1:175018576-175023363:-	0.00228404	0.139593388	0.08947883
297	TOR3A	ENSG00000186283	1:179094217-179094967:+	5.48E-07	0.103937741	0.061998866
298	IPO9	ENSG00000198700	1:201870858-201871160:+	0.00070876	0.061160319	0.096899777
299	IPO9	ENSG00000198700	1:201874372-201874831:+	2.79E-06	0.311343927	0.221568356
300	IPO9	ENSG00000198700	1:201874936-201875151:+	4.53E-06	0.19751435	0.137231949
301	IPO9	ENSG00000198700	1:201875228-201875943:+	3.95E-05	0.096738563	0.068548784
302	KDM5B	ENSG00000117139	1:202729173-202729706:-	0.00072057	0.148668491	0.182176469
303	CYB5R1	ENSG00000159348	1:202963727-202964611:-	0.00340574	0.125680729	0.093921551
304	CYB5R1	ENSG00000159348	1:202964695-202965370:-	0.02800146	0.12038749	0.093897062
305	PPFIA4	ENSG00000143847	1:203045987-203046247:+	2.84E-05	0.220473031	0.167091534
306	SOX13	ENSG00000143842	1:204123804-204124640:+	0.02016732	0.202900222	0.159171806
307	PLEKHA6	ENSG00000143850	1:204222779-204223461:-	5.18E-05	0.343897463	0.267853765
308	PLEKHA6	ENSG00000143850	1:204223585-204228082:-	9.20E-07	0.425285256	0.307350536
309	PLEKHA6	ENSG00000143850	1:204228228-204228727:-	3.99E-05	0.200835365	0.137289679
310	CNTN2	ENSG00000184144	1:205073236-205073655:+	0.01238112	0.157992556	0.130887018
311	EIF2D	ENSG00000143486	1:206591845-206593618:-	0.00015244	0.156834701	0.121890227
312	EIF2D	ENSG00000143486	1:206593793-206595717:-	0.00028054	0.266109671	0.221711661
313	EIF2D	ENSG00000143486	1:206602453-206602950:-	0.0017564	0.235469432	0.173327039
314	PLXNA2	ENSG00000076356	1:208038474-208038824:-	0.02294994	0.205900717	0.15493629
315	SMYD2	ENSG00000143499	1:214331070-214332017:+	0.00611365	0.214440141	0.163068717
316	SMYD2	ENSG00000143499	1:214332192-214334199:+	0.00435619	0.248258907	0.196662725
317	SMYD2	ENSG00000143499	1:214334308-214336703:+	2.03E-05	0.101250346	0.066612426
318	KCTD3	ENSG00000136636	1:215611921-215618885:+	0.01392113	0.073937343	0.086625652
319	EPRS	ENSG00000136628	1:219969122-219972068:-	0.02473918	0.09755036	0.073863143
320	TMEM63A	ENSG00000196187	1:225852769-225853628:-	4.15E-06	0.107096138	0.059272113
321	TMEM63A	ENSG00000196187	1:225853791-225855877:-	1.78E-06	0.138392552	0.069983071
322	PYCR2	ENSG00000143811	1:225921371-225921551:-	1.16E-05	0.106392327	0.072015092
323	PYCR2	ENSG00000143811	1:225921644-225921857:-	1.13E-05	0.129257332	0.086634921
324	PYCR2	ENSG00000143811	1:225922383-225923700:-	1.94E-05	0.296741159	0.226339364
325	COQ8A	ENSG00000163050	1:226982149-226982677:+	0.00083634	0.08839551	0.128389892
326	COQ8A	ENSG00000163050	1:226982763-226982893:+	0.03584356	0.126159512	0.113145057
327	COQ8A	ENSG00000163050	1:226983633-226983760:+	0.00052536	0.327074195	0.252802161
328	COQ8A	ENSG00000163050	1:226983854-226984093:+	0.00328634	0.29182104	0.232449242
329	COQ8A	ENSG00000163050	1:226984235-226984547:+	0.00201613	0.11581059	0.081199239
330	COG2	ENSG00000135775	1:230691564-230693291:+	0.00191763	0.139730751	0.11386963
331	AGT	ENSG00000135744	1:230704337-230705932:-	1.37E-05	0.135726102	0.09196057
332	TTC13	ENSG00000143643	1:230907019-230908711:-	0.00014245	0.106028594	0.077838116
333	C1orf131	ENSG00000143633	1:231224414-231225470:-	0.00658593	0.32676007	0.265930981
334	GNPAT	ENSG00000116906	1:231274062-231275220:+	0.00680841	0.140851248	0.116238705
335	TARBP1	ENSG00000059588	1:234398553-234401180:-	0.00627393	0.087401776	0.084543458
336	SDCCAG8	ENSG00000054282	1:243489140-243499755:+	2.43E-07	0.191726768	0.129427369
337	EFCAB2	ENSG00000203666	1:245083688-245087244:+	0.0007042	0.138183844	0.111234771
338	ZNF692	ENSG00000171163	1:248854051-248855379:-	0.00595236	0.089946866	0.077791203
339	PFKP	ENSG00000067057	10:3120044-3129818:+	2.50E-06	0.32346181	0.228182061
340	RBM17	ENSG00000134453	10:6113581-6114048:+	0.02975952	0.120250478	0.101436938
341	KIN	ENSG00000151657	10:7756142-7759889:-	2.93E-05	0.106765289	0.070717564

S/N	Gene	Ensembl ID	Position of retained intron	p-value	AD_IR_average	Cont_IR_average
342	SEC61A2	ENSG00000065665	10:12158105-12160929:+	0.04687754	0.127793945	0.103432706
343	NUDT5	ENSG00000165609	10:12170770-12170899:-	0.00019124	0.29123098	0.356467581
344	NUDT5	ENSG00000165609	10:12170908-12172764:-	0.00109507	0.101874884	0.122576605
345	CDC123	ENSG00000151465	10:12249718-12250310:+	2.14E-05	0.128483345	0.085881132
346	NMT2	ENSG00000152465	10:15109215-15109701:-	0.00301839	0.177534868	0.180192909
347	NMT2	ENSG00000152465	10:15132933-15133052:-	5.11E-07	0.088162757	0.174554186
348	FAM188A	ENSG00000148481	10:15782226-15786560:-	0.00492667	0.143511656	0.115071592
349	NEBL	ENSG00000078114	10:20814043-20815624:-	0.00353628	0.232438654	0.183362113
350	COMMD3-	ENSG00000269897	10:22317995-22318104:+	0.00095679	0.106680012	0.068970446
351	COMMD3-	ENSG00000269897	10:22318168-22318271:+	0.01317248	0.148669098	0.111780656
352	COMMD3-	ENSG00000269897	10:22318862-22318958:+	0.00851901	0.312909524	0.295830714
353	RAB18	ENSG00000099246	10:27533853-27533927:+	0.00053482	0.136487055	0.099716175
354	BMS1	ENSG00000165733	10:42816672-42817317:+	0.00069332	0.129136823	0.190786513
355	BMS1	ENSG00000165733	10:42820992-42822061:+	0.00851351	0.109038951	0.084820799
356	OGDHL	ENSG00000197444	10:49739839-49740709:-	0.0001852	0.201188345	0.144968091
357	OGDHL	ENSG00000197444	10:49740837-49742827:-	0.01390822	0.126421115	0.123509594
358	UBE2D1	ENSG00000072401	10:58364876-58367922:+	1.30E-07	0.12446652	0.061831603
359	UBE2D1	ENSG00000072401	10:58368016-58368719:+	6.69E-06	0.15744782	0.091677452
360	HNRNPH3	ENSG00000096746	10:68341309-68341584:+	0.01786367	0.083078924	0.099092609
361	RUFY2	ENSG00000204130	10:68355401-68363589:-	0.00147512	0.099066344	0.068494573
362	CCAR1	ENSG00000060339	10:68754827-68755369:+	0.04597967	0.079545135	0.0931193
363	CCAR1	ENSG00000060339	10:68757377-68761006:+	0.00843751	0.089801218	0.109695629
364	VPS26A	ENSG00000122958	10:69168631-69171155:+	0.01592595	0.154930517	0.197631523
365	TSPAN15	ENSG00000099282	10:69498396-69504437:+	0.0003454	0.252270173	0.206809519
366	TSPAN15	ENSG00000099282	10:69504485-69506123:+	3.91E-09	0.320025401	0.218189408
367	TSPAN15	ENSG00000099282	10:69506240-69506828:+	2.81E-08	0.303021926	0.200128309
368	H2AFY2	ENSG00000099284	10:70100297-70109032:+	0.00366526	0.137469426	0.105329394
369	ANAPC16	ENSG00000166295	10:72216138-72220053:+	0.02686247	0.09879957	0.094192231
370	DNAJB12	ENSG00000148719	10:72336696-72338201:-	1.89E-06	0.35863231	0.273625552
371	FAM149B1	ENSG00000138286	10:73234940-73235192:+	0.00500484	0.286865756	0.274704964
372	FAM149B1	ENSG00000138286	10:73239384-73240945:+	0.00236631	0.186497741	0.148595316
373	DNAJC9	ENSG00000213551	10:73246828-73247009:-	0.01663267	0.191634846	0.23938086
374	MRPS16	ENSG00000182180	10:73250991-73251762:-	9.44E-05	0.133390917	0.092351508
375	ZSWIM8	ENSG00000214655	10:73789824-73789955:+	0.00098613	0.133103373	0.183799094
376	ZSWIM8	ENSG00000214655	10:73794330-73794540:+	5.01E-06	0.040486332	0.062728451
377	ZSWIM8	ENSG00000214655	10:73797014-73797112:+	0.01269378	0.420887207	0.46755839
378	ZSWIM8	ENSG00000214655	10:73800170-73800295:+	7.65E-08	0.284049024	0.229288623
379	ZSWIM8	ENSG00000214655	10:73801195-73801315:+	3.81E-05	0.17919582	0.138216834
380	NDST2	ENSG00000166507	10:73802776-73802971:-	0.0306747	0.309667902	0.265858068
381	COMTD1	ENSG00000165644	10:75234743-75234937:-	0.01365304	0.294110744	0.248892225
382	ANXA11	ENSG00000122359	10:80155912-80157640:-	2.33E-06	0.103947768	0.072779723
383	RPP30	ENSG00000148688	10:90894891-90895453:+	0.03937296	0.230453694	0.188760522
384	EXOSC1	ENSG00000171311	10:97436551-97437190:-	4.27E-05	0.129850315	0.095550847
385	EXOSC1	ENSG00000171311	10:97437275-97437699:-	5.20E-06	0.132011701	0.092056501
386	EXOSC1	ENSG00000171311	10:97438703-97441170:-	0.00174761	0.126641473	0.102141825
387	ZDHHC16	ENSG00000171307	10:97451918-97452089:+	0.02150509	0.09344446	0.080032773
388	ZDHHC16	ENSG00000171307	10:97452284-97452414:+	0.00017115	0.114518111	0.082427236
389	ZDHHC16	ENSG00000171307	10:97453663-97453798:+	5.97E-07	0.181405582	0.127390065
390	ZDHHC16	ENSG00000171307	10:97453846-97454713:+	2.85E-07	0.213036037	0.155560569
391	ZDHHC16	ENSG00000171307	10:97455783-97455973:+	4.62E-07	0.225754817	0.16960121
392	MMS19	ENSG00000155229	10:97458719-97458799:-	7.30E-05	0.251710761	0.186629356
393	MMS19	ENSG00000155229	10:97459282-97459361:-	6.40E-05	0.161267268	0.129066917
394	MMS19	ENSG00000155229	10:97459741-97460045:-	0.01589619	0.139425763	0.12004007
395	MMS19	ENSG00000155229	10:97460232-97460694:-	0.04149278	0.14320585	0.127137773
396	MMS19	ENSG00000155229	10:97460751-97460906:-	0.00013739	0.115191317	0.086397292
397	MMS19	ENSG00000155229	10:97461007-97461495:-	0.02351975	0.201235506	0.170797838
398	MMS19	ENSG00000155229	10:97461896-97462016:-	0.00021198	0.095997117	0.068535284

S/N	Gene	Ensembl ID	Position of retained intron	p-value	AD_IR_average	Cont_IR_average
399	MMS19	ENSG00000155229	10:97465954-97466058:-	0.00352631	0.124267361	0.100263603
400	MMS19	ENSG00000155229	10:97466159-97466503:-	0.01046461	0.306899463	0.26153313
401	MMS19	ENSG00000155229	10:97466585-97466775:-	0.01747961	0.189243633	0.163791117
402	ZFYVE27	ENSG00000155256	10:97753182-97757264:+	0.00027207	0.219501879	0.167555761
403	ZFYVE27	ENSG00000155256	10:97757311-97757641:+	0.00072368	0.125653443	0.088760742
404	HPS1	ENSG00000107521	10:98425985-98427214:-	0.01415139	0.213814889	0.174600453
405	HPS1	ENSG00000107521	10:98434091-98435634:-	0.00452438	0.063549869	0.049986213
406	SLC25A28	ENSG00000155287	10:99613924-99620044:-	2.94E-05	0.217159156	0.153440852
407	CWF19L1	ENSG00000095485	10:100235764-100236849:-	0.0008483	0.071621345	0.053944817
408	SEC31B	ENSG00000075826	10:100489398-100489702:-	0.00011543	0.476408049	0.390099623
409	SEC31B	ENSG00000075826	10:100490883-100495384:-	0.01462765	0.094545556	0.106077687
410	NDUFB8	ENSG00000166136	10:100523929-100526398:-	7.48E-05	0.088072259	0.069141174
411	LZTS2	ENSG00000107816	10:101002946-101003506:+	0.0090947	0.08841805	0.071105083
412	PDZD7	ENSG00000186862	10:101019217-101020617:-	0.0495867	0.135532523	0.107204496
413	PDZD7	ENSG00000186862	10:101020678-101021797:-	0.00529897	0.171049652	0.121794904
414	POLL	ENSG00000166169	10:101583681-101584601:-	0.02246564	0.176667178	0.190895577
415	MGEA5	ENSG00000198408	10:101813606-101817823:-	0.01749394	0.100078915	0.080063094
416	KCNIP2	ENSG00000120049	10:101827993-101828150:-	0.00899285	0.217131378	0.191560055
417	PPRC1	ENSG00000148840	10:102148694-102148816:+	0.0373747	0.176748934	0.148632082
418	PPRC1	ENSG00000148840	10:102149329-102149925:+	0.00072742	0.096991328	0.071455793
419	GBF1	ENSG00000107862	10:102377140-102379283:+	0.00331428	0.086519488	0.07140856
420	GBF1	ENSG00000107862	10:102379651-102379852:+	0.01784536	0.140683293	0.123651829
421	FBXL15	ENSG00000107872	10:102421507-102421786:+	0.03822348	0.464419024	0.421445416
422	MFSD13A	ENSG00000138111	10:102470075-102470664:+	3.33E-06	0.377935171	0.296197075
423	MFSD13A	ENSG00000138111	10:102472950-102473569:+	0.0142706	0.175695449	0.143528555
424	MFSD13A	ENSG00000138111	10:102474012-102475721:+	0.02564477	0.226674549	0.202266956
425	BORCS7	ENSG00000166275	10:102862180-102862872:+	0.04357374	0.124144493	0.10673588
426	PDCD11	ENSG00000148843	10:103444068-103444516:+	0.01215215	0.129952551	0.100952339
427	ADD3	ENSG00000148700	10:110130486-110133325:+	0.03596179	0.088942837	0.070588411
428	BBIP1	ENSG00000214413	10:110918213-110919120:-	0.00992911	0.072100162	0.054487819
429	ZDHHC6	ENSG00000023041	10:112430907-112432239:-	0.00021419	0.097646001	0.072872867
430	ZDHHC6	ENSG00000023041	10:112433281-112434296:-	0.00203679	0.113667421	0.139389466
431	SFXN4	ENSG00000183605	10:119141319-119146235:-	0.00082831	0.100233211	0.073581606
432	WDR11	ENSG00000120008	10:120906021-120906775:+	0.04339755	0.479653927	0.431179532
433	NSMCE4A	ENSG00000107672	10:121957259-121959323:-	0.00181354	0.120661774	0.095981755
434	NSMCE4A	ENSG00000107672	10:121959595-121960357:-	0.01064195	0.257727268	0.21444943
435	EDRF1	ENSG00000107938	10:125752914-125753693:+	0.03336148	0.103480665	0.118182139
436	UROS	ENSG00000188690	10:125796188-125798064:-	0.01613659	0.104260474	0.083970726
437	DHX32	ENSG00000089876	10:125836855-125838205:-	0.00127962	0.109067921	0.083677934
438	DHX32	ENSG00000089876	10:125838387-125839000:-	0.03157549	0.111602462	0.090147508
439	DHX32	ENSG00000089876	10:125841934-125852292:-	0.01736751	0.148638446	0.11541501
440	DPYSL4	ENSG00000151640	10:132198971-132200355:+	0.01400222	0.085717916	0.11448256
441	DPYSL4	ENSG00000151640	10:132202825-132203761:+	0.00240388	0.282428915	0.22980997
442	KNDC1	ENSG00000171798	10:133160569-133167380:+	0.04057769	0.237369623	0.192514335
443	TUBGCP2	ENSG00000130640	10:133293769-133297951:-	0.02207446	0.141586273	0.192385026
444	ECHS1	ENSG00000127884	10:133366095-133366888:-	7.04E-10	0.119502511	0.074395772
445	ECHS1	ENSG00000127884	10:133366993-133368922:-	0.0063463	0.11087485	0.091284109
446	MTG1	ENSG00000148824	10:133399619-133401528:+	0.04254246	0.484755354	0.454529299
447	MTG1	ENSG00000148824	10:133401590-133402148:+	8.83E-05	0.493384585	0.430794091
448	SYCE1	ENSG00000171772	10:133555903-133555980:-	0.04111819	0.131298861	0.096539921
449	RIC8A	ENSG00000177963	11:212759-212836:+	0.02963792	0.115554673	0.136024743
450	PSMD13	ENSG00000185627	11:247448-248775:+	0.02873558	0.136814805	0.116819368
451	PSMD13	ENSG00000185627	11:248855-248931:+	0.00585909	0.14568699	0.174807066
452	B4GALNT4	ENSG00000182272	11:375773-375846:+	0.04058789	0.29435028	0.243565727
453	SIGIRR	ENSG00000185187	11:406993-407061:-	0.04990693	0.08905731	0.109104306
454	PTDSS2	ENSG00000174915	11:487073-487419:+	0.03874197	0.139846376	0.116050012
455	RNH1	ENSG00000023191	11:495053-497970:-	0.00030205	0.098659341	0.071575909

S/N	Gene	Ensembl ID	Position of retained intron	p-value	AD_IR_average	Cont_IR_average
456	HRAS	ENSG00000174775	11:532522-532630:-	0.04666357	0.194013473	0.164754418
457	PHRF1	ENSG00000070047	11:610347-610500:+	0.00523954	0.070210384	0.084402361
458	IRF7	ENSG00000185507	11:612800-612998:-	0.00699829	0.233195516	0.276026671
459	PDDC1	ENSG00000177225	11:772521-773521:-	0.00011371	0.112559757	0.093331249
460	PDDC1	ENSG00000177225	11:772521-774007:-	0.03222237	0.079014598	0.066209031
461	PIDD1	ENSG00000177595	11:802090-802194:-	0.00039355	0.180183889	0.129897579
462	PNPLA2	ENSG00000177666	11:821860-821957:+	0.01330416	0.104063457	0.061221758
463	PNPLA2	ENSG00000177666	11:822023-822396:+	0.00336589	0.120142179	0.079237029
464	TSPAN4	ENSG00000214063	11:866001-866561:+	0.02127354	0.273280037	0.226505857
465	AP2A2	ENSG00000183020	11:994245-1000431:+	0.04362284	0.080265427	0.100354748
466	BRSK2	ENSG00000174672	11:1445458-1445570:+	0.00012228	0.162567882	0.086255707
467	BRSK2	ENSG00000174672	11:1445907-1449775:+	2.20E-06	0.205796012	0.261242938
468	BRSK2	ENSG00000174672	11:1449836-1450586:+	0.02652777	0.159729821	0.189297749
469	BRSK2	ENSG00000174672	11:1451419-1454484:+	4.65E-05	0.363464579	0.272559892
470	BRSK2	ENSG00000174672	11:1454608-1456347:+	1.52E-06	0.104177134	0.060236426
471	BRSK2	ENSG00000174672	11:1456687-1456954:+	6.51E-06	0.103716878	0.070898243
472	BRSK2	ENSG00000174672	11:1456687-1459191:+	0.04296541	0.121157996	0.086703858
473	BRSK2	ENSG00000174672	11:1457049-1459191:+	2.77E-05	0.146405922	0.104781571
474	MOB2	ENSG00000182208	11:1471419-1480392:-	0.01248418	0.153269927	0.123842309
475	MOB2	ENSG00000182208	11:1480486-1480724:-	0.00111728	0.233835373	0.177652738
476	IFITM10	ENSG00000244242	11:1735429-1747666:-	0.00016991	0.107054216	0.069080683
477	NAP1L4	ENSG00000205531	11:2945646-2949226:-	0.00784145	0.092564194	0.077623321
478	NAP1L4	ENSG00000205531	11:2945646-2951258:-	0.02020373	0.083740265	0.067969501
479	NAP1L4	ENSG00000205531	11:2949264-2951258:-	0.0050828	0.072131004	0.056571841
480	NAP1L4	ENSG00000205531	11:2951315-2951779:-	1.36E-05	0.116233666	0.081521925
481	NAP1L4	ENSG00000205531	11:2951809-2954526:-	1.16E-07	0.118403582	0.075936255
482	CARS	ENSG00000110619	11:3002053-3002540:-	0.00545699	0.216408705	0.180241391
483	APBB1	ENSG00000166313	11:6401688-6402081:-	0.0005676	0.050258049	0.047792946
484	RRP8	ENSG00000132275	11:6601055-6601148:-	0.00398113	0.240173099	0.187498385
485	ILK	ENSG00000166333	11:6604360-6608045:+	9.75E-05	0.176338104	0.238622417
486	ILK	ENSG00000166333	11:6608211-6608393:+	0.00123121	0.200729461	0.24964113
487	ILK	ENSG00000166333	11:6608489-6608693:+	0.03272281	0.17709466	0.204285569
488	ILK	ENSG00000166333	11:6610278-6610461:+	0.0028777	0.103122494	0.08302876
489	TAF10	ENSG00000166337	11:6611452-6611663:-	0.00075395	0.167867832	0.135442661
490	MRPL17	ENSG00000158042	11:6682815-6683122:-	0.01254761	0.244687007	0.205619752
491	RIC3	ENSG00000166405	11:8126804-8137377:-	0.04605343	0.074955418	0.088862071
492	ST5	ENSG00000166444	11:8698974-8699212:-	1.69E-06	0.152994663	0.109083901
493	ST5	ENSG00000166444	11:8710914-8711121:-	0.02366556	0.134176248	0.114975666
494	ST5	ENSG00000166444	11:8712735-8713997:-	0.00766854	0.109315176	0.086594864
495	DENND5A	ENSG00000184014	11:9145815-9147029:-	0.00247183	0.090767938	0.065624764
496	MICAL2	ENSG00000133816	11:12241162-12242213:+	0.01313806	0.456930695	0.399310805
497	MICAL2	ENSG00000133816	11:12242772-12243986:+	2.50E-06	0.129430929	0.083160592
498	MICAL2	ENSG00000133816	11:12244112-12249183:+	0.00025686	0.077545937	0.061697118
499	MICAL2	ENSG00000133816	11:12244112-12255642:+	0.02988393	0.077932571	0.060820631
500	MICAL2	ENSG00000133816	11:12249246-12255642:+	3.61E-05	0.079540801	0.061278977
501	PSMA1	ENSG00000129084	11:14513887-14514402:-	0.01083473	0.104021691	0.128766666
502	CYP2R1	ENSG00000186104	11:14878297-14879113:-	1.33E-05	0.14445266	0.101096594
503	ABCC8	ENSG00000006071	11:17395930-17396915:-	0.00599043	0.454052805	0.391852623
504	SAAL1	ENSG00000166788	11:18081503-18083534:-	0.0017253	0.308002184	0.288025484
505	PAX6	ENSG00000007372	11:31800856-31801560:-	0.00145065	0.080580831	0.11560541
506	PAX6	ENSG00000007372	11:31801776-31801870:-	0.02537386	0.050860845	0.052019456
507	CSTF3	ENSG00000176102	11:33085773-33085894:-	0.02065677	0.095294624	0.072443066
508	CSTF3	ENSG00000176102	11:33141762-33141884:-	0.00012111	0.567517939	0.485031883
509	NAT10	ENSG00000135372	11:34134420-34134511:+	1.96E-08	0.1957237	0.104075786
510	TTC17	ENSG00000052841	11:43444209-43445930:+	0.00184027	0.148645421	0.138370716
511	CRY2	ENSG00000121671	11:45870532-45870841:+	0.0187936	0.197686787	0.179043394
512	PEX16	ENSG00000121680	11:45910962-45913818:-	7.01E-05	0.10609058	0.067594327

S/N	Gene	Ensembl ID	Position of retained intron	p-value	AD_IR_average	Cont_IR_average
513	AMBRA1	ENSG00000110497	11:46433628-46434848:-	0.00024873	0.099503398	0.072162601
514	ARHGAP1	ENSG00000175220	11:46680282-46680486:-	0.00320577	0.161985007	0.129539726
515	C11orf49	ENSG00000149179	11:47155261-47157022:+	1.17E-07	0.126056834	0.081844575
516	C11orf49	ENSG00000149179	11:47157218-47157812:+	8.50E-07	0.129936907	0.088158365
517	ARFGAP2	ENSG00000149182	11:47168251-47171425:-	0.0250253	0.081054748	0.10545811
518	ARFGAP2	ENSG00000149182	11:47171800-47172280:-	0.00246654	0.094998246	0.099099186
519	ARFGAP2	ENSG00000149182	11:47172333-47173425:-	0.00228001	0.240111156	0.200149581
520	ARFGAP2	ENSG00000149182	11:47175098-47175181:-	0.006994	0.133892018	0.109291221
521	ARFGAP2	ENSG00000149182	11:47175313-47175850:-	0.00712792	0.118314837	0.10501543
522	DDB2	ENSG00000134574	11:47238001-47238137:+	0.00024998	0.275203134	0.223026886
523	DDB2	ENSG00000134574	11:47238183-47238799:+	0.00017916	0.187672922	0.146457355
524	NR1H3	ENSG00000025434	11:47268026-47268260:+	0.03699046	0.14366199	0.118511236
525	MADD	ENSG00000110514	11:47309042-47309280:+	0.00915388	0.184126301	0.153856453
526	MADD	ENSG00000110514	11:47326807-47328657:+	0.02159706	0.187105512	0.178444382
527	SLC39A13	ENSG00000165915	11:47412039-47412345:+	0.02362045	0.120317939	0.14325006
528	KBTBD4	ENSG00000123444	11:47573790-47575592:-	0.00130641	0.123766483	0.107169226
529	FNBP4	ENSG00000109920	11:47724778-47731373:-	0.00604368	0.154866606	0.190313032
530	FNBP4	ENSG00000109920	11:47751290-47752915:-	0.00181567	0.091791938	0.148036621
531	VPS37C	ENSG00000167987	11:61132539-61133254:-	0.02896784	0.14643117	0.127342917
532	VPS37C	ENSG00000167987	11:61134207-61138736:-	0.01311079	0.127973691	0.104189871
533	CPSF7	ENSG00000149532	11:61415784-61416104:-	0.00203039	0.137648989	0.189146616
534	FADS1	ENSG00000149485	11:61802926-61803031:-	0.04342037	0.15233192	0.132033875
535	FADS3	ENSG00000221968	11:61876963-61877510:-	8.73E-08	0.557067415	0.449547844
536	TUT1	ENSG00000149016	11:62576749-62576906:-	0.00136118	0.132242654	0.093658444
537	TUT1	ENSG00000149016	11:62577017-62577181:-	0.00523423	0.117556213	0.084695061
538	MTA2	ENSG00000149480	11:62594040-62594258:-	0.00223089	0.09776691	0.070349172
539	EML3	ENSG00000149499	11:62602678-62602758:-	0.00848752	0.116103693	0.087909231
540	EML3	ENSG00000149499	11:62604041-62604112:-	0.00017443	0.261922857	0.184143853
541	EML3	ENSG00000149499	11:62604201-62605112:-	0.0222839	0.214938344	0.170289839
542	B3GAT3	ENSG00000149541	11:62620671-62621865:-	0.0004864	0.094736704	0.069085414
543	C11orf98	ENSG00000278615	11:62664973-62665130:-	0.00201591	0.234788585	0.198470379
544	UBXN1	ENSG00000162191	11:62676639-62676812:-	0.01299854	0.100181835	0.078984435
545	TMEM179	ENSG00000185475	11:62789426-62789600:+	1.81E-07	0.290604549	0.217416897
546	NXF1	ENSG00000162231	11:62803978-62805328:-	0.04861047	0.212424246	0.186940982
547	STX5	ENSG00000162236	11:62825339-62825422:-	0.00156394	0.096082028	0.071715875
548	C11orf84	ENSG00000168005	11:63819002-63826927:+	7.18E-07	0.271716501	0.213806203
549	MARK2	ENSG00000072518	11:63898280-63898607:+	0.00055508	0.180258687	0.140743053
550	MARK2	ENSG00000072518	11:63905043-63908876:+	0.00021712	0.115994349	0.127682423
551	NAA40	ENSG00000110583	11:63954049-63954337:+	0.04957023	0.177435639	0.147891897
552	NUDT22	ENSG00000149761	11:64227132-64227567:+	0.02615424	0.213571995	0.198380012
553	NUDT22	ENSG00000149761	11:64229571-64229849:+	3.00E-05	0.282002915	0.212246482
554	DNAJC4	ENSG00000110011	11:64231964-64232429:+	0.00201228	0.125184506	0.099166643
555	DNAJC4	ENSG00000110011	11:64232865-64233893:+	0.03466391	0.104136839	0.091670087
556	FKBP2	ENSG00000173486	11:64242213-64242383:+	0.00013233	0.09670292	0.079902812
557	PLCB3	ENSG00000149782	11:64259244-64260028:+	0.03538727	0.088003138	0.068755602
558	PLCB3	ENSG00000149782	11:64262808-64263497:+	9.83E-05	0.265395493	0.189098877
559	PLCB3	ENSG00000149782	11:64263795-64264020:+	2.70E-05	0.156856533	0.109134921
560	PLCB3	ENSG00000149782	11:64265502-64265885:+	0.04897262	0.077031836	0.085752794
561	PLCB3	ENSG00000149782	11:64266404-64266494:+	0.02346064	0.159259695	0.114942143
562	PLCB3	ENSG00000149782	11:64266552-64267184:+	0.00361872	0.153217871	0.110846599
563	GPR137	ENSG00000173264	11:64287014-64287720:+	0.043091	0.135220487	0.116773914
564	TRMT112	ENSG00000173113	11:64317365-64317448:-	0.0041691	0.123212527	0.096107208
565	CCDC88B	ENSG00000168071	11:64351255-64351475:+	0.00084295	0.758523695	0.656505558
566	CCDC88B	ENSG00000168071	11:64355400-64355559:+	0.02927671	0.13501809	0.118428758
567	RPS6KA4	ENSG00000162302	11:64360592-64361133:+	0.00079838	0.29582822	0.266573719
568	RPS6KA4	ENSG00000162302	11:64361241-64361468:+	0.00305529	0.100002612	0.072424039
569	RPS6KA4	ENSG00000162302	11:64368260-64368467:+	0.00660684	0.154207002	0.122737692

S/N	Gene	Ensembl ID	Position of retained intron	p-value	AD_IR_average	Cont_IR_average
570	RPS6KA4	ENSG00000162302	11:64368797-64369445:+	2.38E-05	0.212949651	0.134954696
571	RPS6KA4	ENSG00000162302	11:64369619-64369698:+	0.00034213	0.151517735	0.097728884
572	MAP4K2	ENSG00000168067	11:64797380-64797500:-	6.67E-05	0.281311134	0.214144457
573	MAP4K2	ENSG00000168067	11:64802121-64802418:-	0.00272447	0.130758144	0.101339302
574	PPP2R5B	ENSG00000068971	11:64928158-64928294:+	0.01308631	0.14498487	0.130129105
575	PPP2R5B	ENSG00000068971	11:64931489-64931558:+	7.23E-05	0.304268805	0.23651671
576	PPP2R5B	ENSG00000068971	11:64931868-64932764:+	7.27E-06	0.100076541	0.073496464
577	TM7SF2	ENSG00000149809	11:65113414-65113490:+	0.00533998	0.20326161	0.153845492
578	TM7SF2	ENSG00000149809	11:65114832-65114912:+	0.00043918	0.131184683	0.08683466
579	TM7SF2	ENSG00000149809	11:65115394-65115475:+	0.02179549	0.285626854	0.246439065
580	SYVN1	ENSG00000162298	11:65128714-65129728:-	0.00087558	0.290030085	0.248949364
581	SYVN1	ENSG00000162298	11:65132351-65132731:-	0.01024691	0.360144012	0.323452195
582	SYVN1	ENSG00000162298	11:65132780-65132921:-	0.02391819	0.116175563	0.098195821
583	CAPN1	ENSG00000014216	11:65187298-65187954:+	0.02416048	0.10521129	0.131675022
584	CAPN1	ENSG00000014216	11:65205721-65206462:+	0.02329515	0.153554255	0.129532181
585	CAPN1	ENSG00000014216	11:65210872-65211259:+	0.03254756	0.138005423	0.11319063
586	DPF2	ENSG00000133884	11:65344069-65344579:+	0.00014497	0.25922089	0.193686657
587	DPF2	ENSG00000133884	11:65344621-65345665:+	0.0013644	0.234452339	0.17953341
588	FRMD8	ENSG00000126391	11:65399859-65400723:+	0.03715757	0.224037329	0.195083339
589	FRMD8	ENSG00000126391	11:65405068-65411241:+	0.00217064	0.186812688	0.245242688
590	SCYL1	ENSG00000142186	11:65526350-65526782:+	0.0003232	0.200944043	0.161389112
591	SCYL1	ENSG00000142186	11:65526873-65526961:+	8.63E-05	0.097010988	0.069104715
592	SCYL1	ENSG00000142186	11:65538182-65538269:+	0.00249892	0.288632951	0.228930891
593	LTBP3	ENSG00000168056	11:65551224-65551401:-	8.18E-07	0.184770956	0.097154255
594	LTBP3	ENSG00000168056	11:65551474-65551547:-	2.41E-05	0.321254249	0.222822739
595	LTBP3	ENSG00000168056	11:65551564-65551971:-	6.91E-06	0.398474134	0.288397087
596	LTBP3	ENSG00000168056	11:65552406-65552859:-	0.00726299	0.078933666	0.062970394
597	MAP3K11	ENSG00000173327	11:65606081-65606690:-	1.36E-12	0.147815675	0.076192599
598	PCNX3	ENSG00000197136	11:65634356-65634537:+	0.04413471	0.093582112	0.077495323
599	SIPA1	ENSG00000213445	11:65649480-65649560:+	0.00118919	0.091862306	0.134799967
600	SIPA1	ENSG00000213445	11:65650192-65650400:+	0.04762615	0.095953854	0.113725321
601	RELA	ENSG00000173039	11:65655000-65655687:-	4.03E-10	0.506187444	0.328691181
602	RELA	ENSG00000173039	11:65658499-65658717:-	0.00027983	0.408851073	0.339512312
603	KAT5	ENSG00000172977	11:65713503-65713592:+	0.00107562	0.092656265	0.084915571
604	KAT5	ENSG00000172977	11:65718954-65719046:+	1.78E-06	0.437412878	0.361346701
605	SNX32	ENSG00000172803	11:65850855-65851054:+	0.02858525	0.241506645	0.195895319
606	MUS81	ENSG00000172732	11:65863205-65863409:+	0.00874503	0.100135532	0.082438696
607	MUS81	ENSG00000172732	11:65865323-65865810:+	0.01715528	0.143255093	0.122936713
608	EFEMP2	ENSG00000172638	11:65868629-65869856:-	0.01557361	0.162218578	0.135364244
609	EFEMP2	ENSG00000172638	11:65870658-65871156:-	0.00337133	0.146574229	0.108239819
610	EFEMP2	ENSG00000172638	11:65871363-65871969:-	1.16E-06	0.281839718	0.20133361
611	EFEMP2	ENSG00000172638	11:65872018-65872243:-	4.94E-11	0.13871835	0.075900549
612	FIBP	ENSG00000172500	11:65884656-65884934:-	0.00059826	0.249992305	0.191636334
613	FIBP	ENSG00000172500	11:65887726-65887933:-	1.83E-06	0.201410294	0.119746321
614	SF3B2	ENSG00000087365	11:66058405-66058829:+	0.02184804	0.382998171	0.364673122
615	KLC2	ENSG00000174996	11:66263036-66263659:+	1.29E-05	0.156224267	0.107432188
616	KLC2	ENSG00000174996	11:66266490-66266872:+	0.00452284	0.088536135	0.065837918
617	YIF1A	ENSG00000174851	11:66285536-66285700:-	0.0005074	0.293491178	0.226570427
618	YIF1A	ENSG00000174851	11:66285758-66287597:-	2.97E-08	0.363590905	0.253469203
619	YIF1A	ENSG00000174851	11:66287676-66287811:-	8.12E-12	0.329934489	0.183810961
620	YIF1A	ENSG00000174851	11:66287916-66288080:-	3.67E-08	0.295181155	0.190569965
621	YIF1A	ENSG00000174851	11:66288292-66288954:-	1.00E-07	0.359981037	0.243851352
622	BRMS1	ENSG00000174744	11:66341046-66341205:-	0.04130963	0.095541242	0.111129631
623	BRMS1	ENSG00000174744	11:66342241-66344971:-	0.01163918	0.398923915	0.354981284
624	SLC29A2	ENSG00000174669	11:66366021-66366125:-	0.00763083	0.403637476	0.348809481
625	SLC29A2	ENSG00000174669	11:66366564-66367463:-	9.93E-05	0.449983329	0.366330455
626	BBS1	ENSG00000174483	11:66510706-66511012:+	7.30E-07	0.204646316	0.127466362

S/N	Gene	Ensembl ID	Position of retained intron	p-value	AD_IR_average	Cont_IR_average
627	CTSF	ENSG00000174080	11:66564146-66564557:-	3.46E-08	0.426877354	0.317746455
628	CTSF	ENSG00000174080	11:66568082-66568273:-	9.05E-06	0.250620022	0.171274721
629	SPTBN2	ENSG00000173898	11:66688849-66689095:-	0.01634462	0.08211647	0.109084345
630	SPTBN2	ENSG00000173898	11:66721262-66721349:-	0.01488148	0.021893195	0.033034701
631	C11orf80	ENSG00000173715	11:66838445-66842848:+	6.89E-05	0.17447031	0.13874479
632	RCE1	ENSG00000173653	11:66843861-66843955:+	0.00321857	0.117491734	0.083621666
633	RCE1	ENSG00000173653	11:66844039-66844285:+	0.00900569	0.135831588	0.110168248
634	RCE1	ENSG00000173653	11:66845237-66845499:+	0.00152287	0.197100955	0.195094171
635	RCE1	ENSG00000173653	11:66845562-66845859:+	2.08E-05	0.218204244	0.204426013
636	PC	ENSG00000173599	11:66850464-66850673:-	0.01210423	0.090581112	0.114410895
637	LRFN4	ENSG00000173621	11:66859093-66859636:+	0.00210439	0.189902909	0.143225196
638	GRK2	ENSG00000173020	11:67282365-67282434:+	0.04996941	0.143175965	0.114127822
639	ANKRD13D	ENSG00000172932	11:67290213-67290321:+	0.0144467	0.103155357	0.122959716
640	ANKRD13D	ENSG00000172932	11:67292180-67299057:+	9.62E-14	0.513746951	0.360669727
641	ANKRD13D	ENSG00000172932	11:67299124-67299529:+	2.13E-09	0.627010122	0.536233052
642	ANKRD13D	ENSG00000172932	11:67299611-67299826:+	2.78E-05	0.077831683	0.057143725
643	ANKRD13D	ENSG00000172932	11:67301398-67301487:+	0.01199068	0.106181894	0.083873403
644	SSH3	ENSG00000172830	11:67307436-67307548:+	0.00278233	0.75323378	0.696704468
645	SSH3	ENSG00000172830	11:67307939-67308173:+	1.81E-05	0.16385136	0.11271451
646	SSH3	ENSG00000172830	11:67308302-67308411:+	5.85E-07	0.152882786	0.093091601
647	AP003419.	ENSG00000256514	11:67352019-67352690:-	0.04543501	0.141509333	0.109823071
648	RPS6KB2	ENSG00000175634	11:67433042-67433125:+	6.07E-05	0.168246205	0.141771269
649	ORAOV1	ENSG00000149716	11:69668012-69671748:-	0.0115083	0.104959074	0.121651809
650	ORAOV1	ENSG00000149716	11:69671819-69673215:-	0.00908491	0.131855567	0.15501373
651	ORAOV1	ENSG00000149716	11:69673321-69675189:-	0.00088513	0.095399526	0.123675187
652	CTTN	ENSG00000085733	11:70415717-70417012:+	0.03491377	0.185905035	0.157560729
653	CTTN	ENSG00000085733	11:70417123-70419745:+	0.00615071	0.154292527	0.126645978
654	NADSYN1	ENSG00000172890	11:71481404-71481922:+	0.00295814	0.088316072	0.069490074
655	IL18BP	ENSG00000137496	11:72000012-72000350:+	0.04711589	0.079654639	0.102357857
656	NUMA1	ENSG00000137497	11:72006263-72007188:-	0.00636121	0.122982939	0.1037366
657	ARAP1	ENSG00000186635	11:72693470-72693691:-	0.00166825	0.175168348	0.132010313
658	ARAP1	ENSG00000186635	11:72696654-72696982:-	5.06E-06	0.126343374	0.078691414
659	ARAP1	ENSG00000186635	11:72697486-72697597:-	0.0081017	0.211531518	0.161180797
660	ARAP1	ENSG00000186635	11:72697649-72697910:-	0.00176614	0.19528184	0.14263884
661	STARD10	ENSG00000214530	11:72755753-72757766:-	0.00108765	0.201143866	0.147400704
662	STARD10	ENSG00000214530	11:72755753-72758529:-	0.00056564	0.19433817	0.140208044
663	STARD10	ENSG00000214530	11:72757884-72758529:-	0.00011806	0.197903415	0.137237236
664	STARD10	ENSG00000214530	11:72758633-72759233:-	0.00345377	0.113159539	0.085492605
665	ATG16L2	ENSG00000168010	11:72825407-72826172:+	0.04679533	0.284958585	0.243163618
666	ATG16L2	ENSG00000168010	11:72826243-72826517:+	0.00284732	0.270883073	0.210234545
667	ATG16L2	ENSG00000168010	11:72827293-72828358:+	1.09E-06	0.26386997	0.19416513
668	ATG16L2	ENSG00000168010	11:72828508-72828728:+	0.00039792	0.227815422	0.174134461
669	ATG16L2	ENSG00000168010	11:72828984-72829302:+	0.03747749	0.158368665	0.138178549
670	FCHSD2	ENSG00000137478	11:72843328-72843448:-	0.00024921	0.124894892	0.187949052
671	ARHGEF17	ENSG00000110237	11:73362734-73363205:+	0.00154121	0.313582435	0.252595604
672	ARHGEF17	ENSG00000110237	11:73364239-73364451:+	0.00041005	0.171574546	0.113693419
673	ARHGEF17	ENSG00000110237	11:73364600-73365389:+	0.00760316	0.311265307	0.263051688
674	C2CD3	ENSG00000168014	11:74013525-74028286:-	3.76E-05	0.117515202	0.089252792
675	ARRB1	ENSG00000137486	11:75277448-75278608:-	2.52E-05	0.092534354	0.061485347
676	ARRB1	ENSG00000137486	11:75282021-75283286:-	4.13E-07	0.14841897	0.0842685
677	RPS3	ENSG00000149273	11:75404868-75421726:+	0.00239813	0.011922298	0.009315118
678	USP35	ENSG00000118369	11:78210744-78213645:+	3.47E-05	0.112534244	0.076932175
679	GAB2	ENSG00000033327	11:78219415-78220318:-	0.00149354	0.244847161	0.189138422
680	GAB2	ENSG00000033327	11:78220444-78221676:-	0.00277016	0.223425885	0.177711801
681	GAB2	ENSG00000033327	11:78221779-78222104:-	0.00640171	0.172266139	0.136027628
682	GAB2	ENSG00000033327	11:78222195-78223411:-	0.04754531	0.144184034	0.122228802
683	PCF11	ENSG00000165494	11:83182491-83183037:+	0.00294321	0.13157034	0.098643519

S/N	Gene	Ensembl ID	Position of retained intron	p-value	AD_IR_average	Cont_IR_average
684	TMEM126	ENSG00000171204	11:85635778-85636045:+	0.00019751	0.198204738	0.258518857
685	TMEM126,	ENSG00000171202	11:85648089-85650248:+	0.02289756	0.125973239	0.099658617
686	TMEM126,	ENSG00000171202	11:85650341-85654062:+	1.51E-05	0.100112966	0.069712788
687	TMEM126,	ENSG00000171202	11:85654256-85655593:+	4.64E-06	0.127417005	0.087211049
688	TMEM126,	ENSG00000171202	11:85655708-85656308:+	1.26E-05	0.133578413	0.097511261
689	CEP295	ENSG00000166004	11:93727637-93728680:+	0.00193582	0.403427195	0.348061353
690	CEP295	ENSG00000166004	11:93728821-93729433:+	1.58E-06	0.352257463	0.2800926
691	CEP295	ENSG00000166004	11:93729530-93729610:+	4.92E-09	0.37061139	0.283283339
692	CEP295	ENSG00000166004	11:93729969-93730048:+	1.58E-10	0.250302343	0.172621065
693	TAF1D	ENSG00000166012	11:93736304-93736693:-	0.01243485	0.175827299	0.12154731
694	MED17	ENSG00000042429	11:93797719-93801834:+	0.04398724	0.122147967	0.091680232
695	MRE11	ENSG00000020922	11:94420181-94429910:-	0.01776645	0.101153257	0.077519542
696	CWC15	ENSG00000150316	11:94971065-94971374:-	0.00022196	0.10917483	0.081656612
697	CWC15	ENSG00000150316	11:94972193-94973497:-	0.00023401	0.102788903	0.073453036
698	FAM76B	ENSG00000077458	11:95771650-95775921:-	4.34E-06	0.100488744	0.047356649
699	CCDC82	ENSG00000149231	11:96353714-96358992:-	2.61E-05	0.245217427	0.162858109
700	GRIA4	ENSG00000152578	11:105974444-105979574:+	0.00018882	0.133058002	0.087798174
701	USP28	ENSG00000048028	11:113803281-113803797:-	0.00130482	0.192299479	0.133615699
702	USP28	ENSG00000048028	11:113803877-113804672:-	0.00348189	0.152999654	0.102030818
703	ZBTB16	ENSG00000109906	11:114242337-114247197:+	0.00208721	0.182501557	0.133921387
704	REXO2	ENSG00000076043	11:114447879-114449845:+	0.00102939	0.085418819	0.068287595
705	BUD13	ENSG00000137656	11:116757950-116758268:-	2.27E-05	0.087739851	0.142446871
706	BUD13	ENSG00000137656	11:116758407-116759073:-	0.00192141	0.068242606	0.099617401
707	BUD13	ENSG00000137656	11:116759179-116760734:-	0.0169206	0.114242391	0.139965101
708	SIK3	ENSG00000160584	11:116845629-116846382:-	0.00032193	0.144307299	0.11125271
709	SIDT2	ENSG00000149577	11:117183878-117184073:+	0.00162332	0.101430541	0.073840547
710	SIDT2	ENSG00000149577	11:117186223-117186583:+	0.00112948	0.307600902	0.255391273
711	SIDT2	ENSG00000149577	11:117186636-117186989:+	0.0404111	0.212164207	0.186608391
712	SIDT2	ENSG00000149577	11:117187699-117188707:+	0.02612317	0.09541183	0.120272187
713	SIDT2	ENSG00000149577	11:117188826-117189168:+	0.01540167	0.149571199	0.121904736
714	SIDT2	ENSG00000149577	11:117192650-117192819:+	6.37E-05	0.273409976	0.196738456
715	SIDT2	ENSG00000149577	11:117192866-117193152:+	0.0019572	0.291529463	0.243470639
716	PCSK7	ENSG00000160613	11:117218568-117219056:-	0.02449963	0.140321421	0.120274136
717	CEP164	ENSG00000110274	11:117408032-117408889:+	0.00013759	0.194967488	0.140549117
718	IFT46	ENSG00000118096	11:118557045-118559784:-	0.04372601	0.067600118	0.073581579
719	PHLDB1	ENSG00000019144	11:118655690-118656682:+	0.0120066	0.097473246	0.103430681
720	CCDC84	ENSG00000186166	11:119010830-119011220:+	0.03683709	0.220081598	0.194233379
721	CCDC84	ENSG00000186166	11:119012003-119012148:+	6.22E-05	0.189803676	0.154057922
722	CCDC84	ENSG00000186166	11:119012254-119013231:+	1.98E-05	0.224169128	0.184282266
723	TRAPPC4	ENSG00000196655	11:119020253-119021759:+	0.00178242	0.122248915	0.101001369
724	HMBS	ENSG00000256269	11:119089131-119089216:+	0.01064275	0.13974061	0.098396841
725	C2CD2L	ENSG00000172375	11:119112874-119113610:+	0.0003677	0.133980912	0.09552293
726	MCAM	ENSG00000076706	11:119311185-119311279:-	0.00352894	0.091020779	0.142182448
727	ROBO3	ENSG00000154134	11:124878796-124879189:+	0.03496796	0.260857717	0.216725838
728	FOXRED1	ENSG00000110074	11:126273079-126273335:+	0.01989733	0.462961829	0.422736987
729	THYN1	ENSG00000151500	11:134248484-134248808:-	0.01279347	0.160889141	0.137540562
730	THYN1	ENSG00000151500	11:134248959-134249166:-	0.00722871	0.249463668	0.217658156
731	WNK1	ENSG00000060237	12:900670-907846:+	2.83E-05	0.148375163	0.106937647
732	ITFG2	ENSG00000111203	12:2821596-2821691:+	3.22E-05	0.3555625	0.274541413
733	ITFG2	ENSG00000111203	12:2821792-2822793:+	0.01435959	0.131500916	0.108060209
734	DYRK4	ENSG00000010219	12:4610284-4612542:+	0.01271468	0.141655834	0.11536691
735	NCAPD2	ENSG00000010292	12:6529958-6530690:+	8.60E-08	0.19200133	0.268429688
736	NCAPD2	ENSG00000010292	12:6530817-6530920:+	1.64E-06	0.106709409	0.161046865
737	IFFO1	ENSG00000010295	12:6548157-6548424:-	0.03375884	0.057717066	0.072956815
738	MLF2	ENSG00000089693	12:6748547-6748769:-	0.04378253	0.127144102	0.110530499
739	PTMS	ENSG00000159335	12:6769999-6770156:+	0.00584425	0.094122037	0.067426291
740	PTMS	ENSG00000159335	12:6770218-6770391:+	0.00805916	0.095299617	0.071022018

S/N	Gene	Ensembl ID	Position of retained intron	p-value	AD_IR_average	Cont_IR_average
741	GPR162	ENSG00000250510	12:6825673-6826195:+	0.00165618	0.219817605	0.169242416
742	P3H3	ENSG00000110811	12:6831914-6833591:+	0.02708526	0.210607549	0.176474244
743	P3H3	ENSG00000110811	12:6833809-6833924:+	0.00087203	0.400241841	0.45486961
744	P3H3	ENSG00000110811	12:6837086-6837422:+	0.03300732	0.466459246	0.411836186
745	ENO2	ENSG00000111674	12:6916729-6917037:+	0.00414437	0.158730383	0.145498018
746	PHB2	ENSG00000215021	12:6967779-6967891:-	7.07E-08	0.15315036	0.097054471
747	LPCAT3	ENSG00000111684	12:6976891-6977133:-	2.04E-07	0.137531	0.100461421
748	LPCAT3	ENSG00000111684	12:6977262-6977366:-	0.00011349	0.177221235	0.144549748
749	LPCAT3	ENSG00000111684	12:6977525-6977597:-	0.00151646	0.415143195	0.341454169
750	MAGOHB	ENSG00000111196	12:10606374-10607853:-	0.00472493	0.154036538	0.118455671
751	GOLT1B	ENSG00000111711	12:21512376-21515668:+	0.00539505	0.121047653	0.164083162
752	C2CD5	ENSG00000111731	12:22457161-22458483:-	0.0324014	0.093080313	0.076053257
753	FGFR1OP2	ENSG00000111790	12:26957743-26960514:+	0.01083939	0.053487601	0.050542256
754	KIAA1551	ENSG00000174718	12:31985957-31987238:+	0.00270772	0.457393756	0.379558468
755	KIAA1551	ENSG00000174718	12:31987322-31992377:+	0.00042039	0.182674198	0.142332018
756	YARS2	ENSG00000139131	12:32747363-32749936:-	0.00155147	0.145729529	0.116236413
757	RAPGEF3	ENSG00000079337	12:47748931-47749389:-	0.00535064	0.508762378	0.414401675
758	HDAC7	ENSG00000061273	12:47785471-47785751:-	0.00035341	0.407270915	0.476872792
759	PFKM	ENSG00000152556	12:48135038-48135290:+	7.26E-05	0.305405006	0.243390577
760	KANSL2	ENSG00000139620	12:48655060-48660365:-	0.00252296	0.078058341	0.09631091
761	ADCY6	ENSG00000174233	12:48768716-48768936:-	5.38E-06	0.082529356	0.123862197
762	ADCY6	ENSG00000174233	12:48772424-48772507:-	0.01195184	0.062588222	0.083866585
763	PRKAG1	ENSG00000181929	12:49003595-49003756:-	0.02420428	0.208344634	0.196951119
764	KMT2D	ENSG00000167548	12:49030768-49030892:-	0.04139707	0.0819046	0.099264293
765	LMBR1L	ENSG00000139636	12:49097739-49097943:-	7.06E-05	0.207434654	0.15865814
766	LMBR1L	ENSG00000139636	12:49098105-49100387:-	0.00201779	0.10044635	0.081086584
767	LMBR1L	ENSG00000139636	12:49100454-49100555:-	0.00321575	0.301832341	0.25080987
768	LMBR1L	ENSG00000139636	12:49101323-49101471:-	0.00011747	0.27550039	0.202248648
769	LMBR1L	ENSG00000139636	12:49101549-49102119:-	3.88E-05	0.332437939	0.259919417
770	LMBR1L	ENSG00000139636	12:49102196-49102292:-	5.17E-05	0.28869278	0.218431056
771	LMBR1L	ENSG00000139636	12:49102951-49103090:-	0.00012988	0.185294687	0.137352323
772	PRPF40B	ENSG00000110844	12:49631544-49631859:+	0.01328304	0.107468521	0.079768824
773	PRPF40B	ENSG00000110844	12:49635263-49635364:+	0.02027287	0.090365318	0.070723015
774	PRPF40B	ENSG00000110844	12:49636849-49637469:+	0.00426198	0.319629402	0.367866039
775	PRPF40B	ENSG00000110844	12:49642024-49642234:+	0.02742352	0.287137927	0.24549696
776	PRPF40B	ENSG00000110844	12:49643752-49643860:+	0.01509046	0.197269171	0.162851553
777	FMNL3	ENSG00000161791	12:49652212-49653225:-	0.01448281	0.11308447	0.142094717
778	NCKAP5L	ENSG00000167566	12:49801967-49802957:-	0.00150212	0.388354374	0.337814169
779	ASIC1	ENSG00000110881	12:50077363-50077999:+	0.00610781	0.217444198	0.175865935
780	ASIC1	ENSG00000110881	12:50078577-50078923:+	7.48E-05	0.269185829	0.206418132
781	ASIC1	ENSG00000110881	12:50078980-50079901:+	0.00017931	0.394602459	0.320993221
782	ASIC1	ENSG00000110881	12:50080589-50081101:+	0.00052902	0.181882504	0.136664638
783	ASIC1	ENSG00000110881	12:50081181-50081259:+	0.00136703	0.215698563	0.166251249
784	ASIC1	ENSG00000110881	12:50081364-50081544:+	0.00020083	0.140561856	0.102527206
785	SMARCD1	ENSG00000066117	12:50086878-50087362:+	0.0108944	0.167905777	0.147965716
786	CERS5	ENSG00000139624	12:50134702-50135731:-	3.37E-05	0.064790333	0.089502306
787	DIP2B	ENSG00000066084	12:50678876-50680671:+	0.04558436	0.09217724	0.14062701
788	POU6F1	ENSG00000184271	12:51196927-51197769:-	0.00648476	0.191826338	0.156882831
789	POU6F1	ENSG00000184271	12:51204368-51206788:-	0.00134632	0.086270989	0.126540708
790	NR4A1	ENSG00000123358	12:52057259-52057351:+	8.58E-07	0.069615991	0.037024187
791	NR4A1	ENSG00000123358	12:52057530-52058687:+	0.00021497	0.072825866	0.05262938
792	TNS2	ENSG00000111077	12:53055845-53057012:+	0.03550486	0.191105906	0.228947703
793	CSAD	ENSG00000139631	12:53172436-53172521:-	0.00073471	0.230905465	0.179324261
794	AAAS	ENSG00000094914	12:53307713-53307844:-	0.00036991	0.19100208	0.138209173
795	AAAS	ENSG00000094914	12:53307929-53308051:-	0.00087929	0.239419415	0.183974783
796	AAAS	ENSG00000094914	12:53314441-53314750:-	0.00630389	0.125828665	0.117935382
797	AAAS	ENSG00000094914	12:53314849-53315093:-	0.00147063	0.106056574	0.090375749

S/N	Gene	Ensembl ID	Position of retained intron	p-value	AD_IR_average	Cont_IR_average
798	AAAS	ENSG00000094914	12:53315426-53315726:-	0.00329043	0.139822552	0.107898187
799	PCBP2	ENSG00000197111	12:53467293-53468776:+	8.57E-06	0.056245155	0.075208001
800	MAP3K12	ENSG00000139625	12:53481280-53481940:-	0.00097435	0.113265839	0.085483271
801	MAP3K12	ENSG00000139625	12:53482369-53482564:-	0.00329996	0.189270088	0.234538108
802	TARBP2	ENSG00000139546	12:53502184-53503026:+	0.00061232	0.142976837	0.104643223
803	TARBP2	ENSG00000139546	12:53504815-53505134:+	0.00023194	0.199717334	0.151801481
804	TARBP2	ENSG00000139546	12:53505262-53505648:+	0.03650228	0.11178544	0.09405593
805	ATF7	ENSG00000170653	12:53517354-53523275:-	0.00022629	0.066365111	0.097792821
806	ATF7	ENSG00000170653	12:53534659-53537414:-	0.03192907	0.10061832	0.122359501
807	CALCOCO1	ENSG00000012822	12:53712121-53713099:-	0.0023635	0.270913	0.326095218
808	CALCOCO1	ENSG00000012822	12:53713900-53714132:-	4.69E-05	0.64740322	0.562059935
809	CALCOCO1	ENSG00000012822	12:53716047-53716259:-	0.00280672	0.084533476	0.109114838
810	HNRNPA1	ENSG00000135486	12:54284317-54284548:+	2.43E-05	0.097639123	0.057203888
811	RDH5	ENSG00000135437	12:55724049-55724321:+	0.00231857	0.439017268	0.370328364
812	ORMDL2	ENSG00000123353	12:55819173-55819341:+	6.38E-10	0.212605983	0.111168618
813	ORMDL2	ENSG00000123353	12:55819493-55820259:+	1.64E-12	0.197034189	0.095065895
814	RPL41	ENSG00000229117	12:56116659-56116774:+	0.04691913	0.333875122	0.290739597
815	MYL6	ENSG00000092841	12:56158711-56158901:+	0.0030842	0.023641264	0.026394233
816	MYL6	ENSG00000092841	12:56158711-56159974:+	5.49E-08	0.023055445	0.025285638
817	MYL6	ENSG00000092841	12:56160320-56160625:+	3.45E-05	0.087897776	0.076463304
818	SMARCC2	ENSG00000139613	12:56181101-56181481:-	0.00055045	0.136138546	0.109353975
819	NABP2	ENSG00000139579	12:56224441-56224833:+	6.86E-06	0.570169085	0.469806078
820	ANKRD52	ENSG00000139645	12:56244133-56244352:-	0.04641304	0.209848605	0.17708449
821	PAN2	ENSG00000135473	12:56318434-56319087:-	0.02822322	0.103808939	0.096889138
822	PAN2	ENSG00000135473	12:56319181-56319307:-	2.00E-06	0.68589022	0.599081714
823	PAN2	ENSG00000135473	12:56322482-56322614:-	0.03283468	0.103438222	0.124133727
824	STAT2	ENSG00000170581	12:56346955-56348528:-	0.00090669	0.079912005	0.123013142
825	STAT2	ENSG00000170581	12:56348623-56348751:-	0.03977498	0.10399478	0.130819173
826	STAT2	ENSG00000170581	12:56349059-56349162:-	0.03742384	0.166778328	0.206057881
827	BAZ2A	ENSG00000076108	12:56606733-56609735:-	0.03938487	0.087555444	0.118635095
828	PRIM1	ENSG00000198056	12:56731734-56734146:-	0.02620641	0.167779799	0.147939994
829	LRP1	ENSG00000123384	12:57162983-57165804:+	0.01884347	0.1050319	0.15884271
830	LRP1	ENSG00000123384	12:57203521-57204409:+	0.04366979	0.084808791	0.113278378
831	LRP1	ENSG00000123384	12:57205677-57206472:+	0.00825865	0.092178412	0.075863271
832	SHMT2	ENSG00000182199	12:57232575-57232703:+	0.00423287	0.095897145	0.073406834
833	SHMT2	ENSG00000182199	12:57232843-57233179:+	0.00014672	0.269385244	0.215409294
834	SHMT2	ENSG00000182199	12:57233345-57233562:+	0.01952099	0.092182754	0.074004106
835	R3HDM2	ENSG00000179912	12:57268457-57268921:-	0.00645852	0.156756916	0.128632879
836	MARS	ENSG00000166986	12:57512353-57512750:+	2.72E-05	0.37946161	0.292420312
837	MARS	ENSG00000166986	12:57515336-57515919:+	7.65E-06	0.09336345	0.064655683
838	MBD6	ENSG00000166987	12:57524092-57524279:+	1.00E-05	0.317081229	0.224112283
839	MBD6	ENSG00000166987	12:57526388-57526565:+	0.00424853	0.155335338	0.110545368
840	MBD6	ENSG00000166987	12:57527227-57527506:+	0.00605149	0.377365659	0.307542026
841	MBD6	ENSG00000166987	12:57527660-57527847:+	0.00232524	0.28928401	0.223860883
842	KIF5A	ENSG00000155980	12:57581568-57581869:+	0.00030857	0.097025761	0.071597876
843	KIF5A	ENSG00000155980	12:57582629-57583100:+	0.01599944	0.160389128	0.138351267
844	DTX3	ENSG00000178498	12:57608737-57609076:+	3.19E-11	0.290335367	0.16856077
845	ARHGEF25	ENSG00000240771	12:57614119-57614330:+	0.01020347	0.087591578	0.071293344
846	ARHGEF25	ENSG00000240771	12:57614400-57614515:+	5.21E-06	0.158592211	0.120856778
847	ARHGEF25	ENSG00000240771	12:57614605-57614688:+	1.12E-06	0.173519438	0.130918235
848	SLC26A10	ENSG00000135502	12:57624982-57625122:+	1.65E-08	0.773565805	0.669616299
849	OS9	ENSG00000135506	12:57696038-57696274:+	0.0062532	0.123074687	0.114994223
850	OS9	ENSG00000135506	12:57719182-57720405:+	0.0146415	0.063315712	0.059486126
851	OS9	ENSG00000135506	12:57720263-57720405:+	0.00157541	0.059855518	0.073287639
852	AGAP2	ENSG00000135439	12:57734679-57735368:-	0.00468412	0.216136755	0.167173105
853	AGAP2	ENSG00000135439	12:57735427-57737078:-	0.00751703	0.160210707	0.206050882
854	TSPAN31	ENSG00000135452	12:57745217-57745744:+	7.52E-05	0.125189302	0.090625793

S/N	Gene	Ensembl ID	Position of retained intron	p-value	AD_IR_average	Cont_IR_average
855	TSPAN31	ENSG00000135452	12:57745912-57746175:+	3.79E-05	0.117347732	0.077851113
856	TSPAN31	ENSG00000135452	12:57746720-57747017:+	1.61E-08	0.176706227	0.115347205
857	CDK4	ENSG00000135446	12:57748617-57749181:-	0.00241295	0.177196699	0.152203496
858	CDK4	ENSG00000135446	12:57749317-57749453:-	0.01256133	0.21494042	0.185803127
859	March9 ge	ENSG00000139266	12:57755885-57756928:+	0.00072632	0.254766866	0.191476361
860	AVIL	ENSG00000135407	12:57797995-57799794:-	0.04238526	0.237059415	0.195916005
861	TBK1	ENSG00000183735	12:64498039-64501329:+	0.00470976	0.107323224	0.081258396
862	MDM1	ENSG00000111554	12:68295366-68296922:-	2.82E-05	0.114026182	0.075562377
863	MDM1	ENSG00000111554	12:68296982-68302619:-	1.04E-08	0.082856644	0.038967754
864	CPSF6	ENSG00000111605	12:69259543-69260043:+	0.00922007	0.123082421	0.094824449
865	METTL25	ENSG00000127720	12:82476718-82477280:+	2.77E-09	0.39747121	0.272938346
866	METTL25	ENSG00000127720	12:82477352-82478931:+	0.00323446	0.309777416	0.252619704
867	NR2C1	ENSG00000120798	12:95025255-95028386:-	0.02696276	0.292917082	0.251346497
868	NR2C1	ENSG00000120798	12:95057643-95057730:-	0.00091783	0.128670868	0.092886503
869	LTA4H	ENSG00000111144	12:96003064-96003837:-	7.49E-06	0.206788061	0.153624131
870	LTA4H	ENSG00000111144	12:96003064-96006313:-	8.75E-06	0.061173118	0.04225346
871	SLC25A3	ENSG00000075415	12:98593740-98593974:+	0.00281336	0.350287506	0.261406208
872	UTP20	ENSG00000120800	12:101383669-101385582:+	0.00016956	0.499467171	0.418628416
873	UTP20	ENSG00000120800	12:101385728-101385967:+	1.49E-07	0.42907128	0.310674214
874	CRY1	ENSG00000008405	12:106992890-106992964:-	0.00521226	0.20751612	0.156262355
875	ACACB	ENSG00000076555	12:109259108-109260479:+	0.01565129	0.081727991	0.107568042
876	PPP1CC	ENSG00000186298	12:110722269-110722471:-	0.00119408	0.082442583	0.118763795
877	ATXN2	ENSG00000204842	12:111453845-111456028:-	0.00989741	0.321637805	0.282116416
878	MAPKAPK5	ENSG00000089022	12:111889000-111890039:+	0.01041081	0.081848981	0.067651851
879	TMEM116	ENSG00000198270	12:111936830-111937159:-	0.00013083	0.104951972	0.061570415
880	TRAFD1	ENSG00000135148	12:112152140-112152426:+	0.03568369	0.163653793	0.19051184
881	HECTD4	ENSG00000173064	12:112226758-112228088:-	0.00157392	0.078718818	0.146020292
882	TPCN1	ENSG00000186815	12:113280195-113284580:+	0.00167664	0.068353889	0.090905045
883	TPCN1	ENSG00000186815	12:113288234-113288757:+	3.41E-10	0.753888768	0.626315545
884	SLC8B1	ENSG00000089060	12:113299974-113304320:-	0.00033377	0.15851736	0.122973669
885	SLC8B1	ENSG00000089060	12:113321108-113321195:-	1.52E-05	0.13005634	0.068645815
886	TAOK3	ENSG00000135090	12:118152409-118160145:-	0.00042607	0.098877795	0.072780939
887	SIRT4	ENSG00000089163	12:120312750-120312883:+	0.00052562	0.090343189	0.123371918
888	DYNLL1	ENSG00000088986	12:120496216-120496401:+	0.03368533	0.028924858	0.020560072
889	RNF10	ENSG00000022840	12:120563070-120563331:+	0.00709061	0.018450866	0.027593975
890	POP5	ENSG00000167272	12:120579412-120579513:-	0.01150773	0.141800902	0.108057778
891	CABP1	ENSG00000157782	12:120659908-120660195:+	0.0296104	0.267987171	0.312849479
892	ACADS	ENSG00000122971	12:120738670-120738819:+	0.0004627	0.118341301	0.07385007
893	ACADS	ENSG00000122971	12:120738915-120739139:+	0.00081273	0.141475594	0.083467359
894	P2RX4	ENSG00000135124	12:121232676-121232996:+	0.00248175	0.212418057	0.171911199
895	ANAPC5	ENSG00000089053	12:121318424-121318500:-	0.00016716	0.13426059	0.092175957
896	TMEM120	ENSG00000188735	12:121752223-121761648:+	0.00653001	0.169934943	0.140312222
897	PSMD9	ENSG00000110801	12:121899845-121903005:+	0.01971399	0.083052868	0.071165757
898	MLXIP	ENSG00000175727	12:122137590-122138193:+	0.0031566	0.333256451	0.268591234
899	HIP1R	ENSG00000130787	12:122851297-122854042:+	0.00070512	0.112212404	0.148512419
900	HIP1R	ENSG00000130787	12:122860077-122860147:+	0.02607762	0.182110478	0.210730636
901	ABCB9	ENSG00000150967	12:122935431-122940110:-	6.23E-05	0.184841473	0.12802693
902	ARL6IP4	ENSG00000182196	12:122980812-122981128:+	0.0002135	0.200863238	0.146667496
903	C12orf65	ENSG00000130921	12:123253956-123256812:+	0.01484134	0.094572648	0.079873468
904	EIF2B1	ENSG00000111361	12:123621920-123622635:-	0.01174487	0.123570138	0.141909149
905	NCOR2	ENSG00000196498	12:124334617-124335134:-	0.01184464	0.167117584	0.129221945
906	NCOR2	ENSG00000196498	12:124335632-124336752:-	0.00022163	0.234875476	0.197888923
907	NCOR2	ENSG00000196498	12:124355571-124356641:-	1.72E-05	0.163595288	0.239362699
908	NCOR2	ENSG00000196498	12:124362297-124363678:-	0.04034812	0.040034624	0.045846736
909	SFSWAP	ENSG00000061936	12:131711447-131714070:+	0.00355042	0.253748057	0.304826896
910	SFSWAP	ENSG00000061936	12:131714240-131714821:+	1.33E-05	0.188535123	0.23407357
911	SFSWAP	ENSG00000061936	12:131753363-131754367:+	0.01563647	0.312336424	0.266629384

S/N	Gene	Ensembl ID	Position of retained intron	p-value	AD_IR_average	Cont_IR_average
912	ULK1	ENSG00000177169	12:131908817-131908897:+	0.01367532	0.111891013	0.079741097
913	ULK1	ENSG00000177169	12:131909237-131909774:+	0.04104792	0.078492694	0.089969666
914	ULK1	ENSG00000177169	12:131915421-131915890:+	0.0019152	0.462083768	0.384358701
915	PUS1	ENSG00000177192	12:131939275-131941291:+	0.01037042	0.165174067	0.14314357
916	EP400	ENSG00000183495	12:132067486-132069494:+	0.00541712	0.110352082	0.146016103
917	DDX51	ENSG00000185163	12:132142422-132142727:-	0.00013874	0.238309905	0.170164126
918	DDX51	ENSG00000185163	12:132142878-132143694:-	0.00098005	0.410912182	0.331678664
919	FBRSL1	ENSG00000112787	12:132574348-132574492:+	0.00123259	0.121111213	0.078434886
920	POLE	ENSG00000177084	12:132625770-132626116:-	6.53E-05	0.110799449	0.069553422
921	CHFR	ENSG00000072609	12:132847130-132848084:-	0.02369809	0.107614602	0.127883861
922	MPHOSPH	ENSG00000196199	13:19647291-19648421:+	6.84E-05	0.07372964	0.111647358
923	MPHOSPH	ENSG00000196199	13:19666579-19668376:+	0.02601787	0.350693829	0.403283805
924	MPHOSPH	ENSG00000196199	13:19670363-19670839:+	0.03962216	0.454031122	0.424293727
925	MPHOSPH	ENSG00000196199	13:19671289-19671833:+	0.00061334	0.249697882	0.196284044
926	PSPC1	ENSG00000121390	13:19705831-19709541:-	0.02824889	0.121731087	0.08827294
927	MICU2	ENSG00000165487	13:21495318-21496051:-	1.33E-09	0.235781273	0.160408321
928	CENPJ	ENSG00000151849	13:24884083-24884160:-	0.02372333	0.21455839	0.154502831
929	CENPJ	ENSG00000151849	13:24884463-24885275:-	0.00153686	0.363730293	0.300616108
930	CDK8	ENSG00000132964	13:26400550-26401268:+	0.00865002	0.094631624	0.066564197
931	CDK8	ENSG00000132964	13:26401347-26401465:+	0.01542246	0.071314973	0.054323916
932	HSPH1	ENSG00000120694	13:31137524-31138406:-	1.24E-07	0.053663794	0.041260792
933	HSPH1	ENSG00000120694	13:31141259-31143791:-	1.66E-08	0.034016302	0.022377593
934	HSPH1	ENSG00000120694	13:31143923-31145562:-	1.01E-06	0.042415037	0.039345152
935	N4BP2L2	ENSG00000244754	13:32521449-32522181:-	0.0413783	0.229691248	0.199802984
936	EXOSC8	ENSG00000120699	13:37002309-37002487:+	0.00925191	0.097246252	0.069630236
937	SUPT20H	ENSG00000102710	13:37009809-37010551:-	5.82E-06	0.152565009	0.121790143
938	SUPT20H	ENSG00000102710	13:37010655-37012191:-	2.07E-08	0.104403105	0.073244685
939	UFM1	ENSG00000120686	13:38358132-38359300:+	0.01470416	0.124349496	0.084022015
940	MRPS31	ENSG00000102738	13:40749281-40754018:-	1.17E-05	0.237227082	0.164630514
941	MRPS31	ENSG00000102738	13:40754092-40756872:-	5.34E-07	0.215585505	0.131384148
942	WBP4	ENSG00000120688	13:41062716-41065015:+	0.00736061	0.146544793	0.116407794
943	NAA16	ENSG00000172766	13:41374839-41375404:+	0.01594922	0.160043541	0.126590475
944	SETDB2	ENSG00000136169	13:49483563-49485629:+	0.0116929	0.109328404	0.085009647
945	PHF11	ENSG00000136147	13:49522107-49523174:+	0.01863168	0.094206021	0.069039771
946	TGDS	ENSG00000088451	13:94574852-94576313:-	0.0191247	0.104463907	0.082079165
947	DZIP1	ENSG00000134874	13:95594086-95599364:-	0.04273206	0.096140549	0.0813058
948	CLYBL	ENSG00000125246	13:99864911-99866239:+	0.0435442	0.168515751	0.129137419
949	NAXD	ENSG00000213995	13:110634595-110634671:+	0.02117332	0.470722329	0.416717597
950	NAXD	ENSG00000213995	13:110634776-110635467:+	1.43E-05	0.43960672	0.351182506
951	NAXD	ENSG00000213995	13:110635588-110637128:+	0.00506118	0.203378696	0.165108616
952	CARS2	ENSG00000134905	13:110642521-110644384:-	0.0285113	0.11183299	0.120247953
953	CARS2	ENSG00000134905	13:110651100-110663450:-	0.00834765	0.105591938	0.082222732
954	MCF2L	ENSG00000126217	13:113096653-113096773:+	0.01213349	0.121468654	0.145393338
955	PCID2	ENSG00000126226	13:113177976-113178173:-	0.04489842	0.627784451	0.642609688
956	PCID2	ENSG00000126226	13:113181230-113184345:-	0.00031762	0.171767884	0.130420958
957	PCID2	ENSG00000126226	13:113184487-113185484:-	0.0160925	0.118336589	0.09092167
958	LAMP1	ENSG00000185896	13:113321727-113322281:+	0.0200773	0.136362128	0.126690401
959	TMCO3	ENSG00000150403	13:113534240-113539356:+	0.00279049	0.17495842	0.136662401
960	GAS6	ENSG00000183087	13:113821018-113821957:-	0.00140035	0.204935027	0.152313147
961	GAS6	ENSG00000183087	13:113835635-113838068:-	0.00179707	0.071650391	0.095051173
962	GAS6	ENSG00000183087	13:113846589-113848025:-	0.02487771	0.365118634	0.323309701
963	CDC16	ENSG00000130177	13:114243348-114243855:+	5.03E-05	0.369550854	0.239264264
964	CDC16	ENSG00000130177	13:114243989-114244889:+	0.00210743	0.392973439	0.314221355
965	CDC16	ENSG00000130177	13:114259398-114261886:+	0.00243089	0.130401527	0.115279778
966	UPF3A	ENSG00000169062	13:114286400-114286518:+	0.00576262	0.186018989	0.1705279
967	UPF3A	ENSG00000169062	13:114291792-114298839:+	0.03448323	0.204285812	0.177688987
968	UPF3A	ENSG00000169062	13:114291792-114301730:+	0.00068035	0.099583356	0.079465343

S/N	Gene	Ensembl ID	Position of retained intron	p-value	AD_IR_average	Cont_IR_average
969	PARP2	ENSG00000129484	14:20357149-20357395:+	0.00085336	0.15672572	0.218351568
970	PARP2	ENSG00000129484	14:20357520-20357637:+	1.55E-05	0.366905293	0.478274273
971	OSGEP	ENSG00000092094	14:20448811-20448963:-	0.04428062	0.164142927	0.145619455
972	TMEM55B	ENSG00000165782	14:20458702-20459205:-	0.02022734	0.107631327	0.093378004
973	TMEM55B	ENSG00000165782	14:20459440-20459627:-	7.26E-05	0.128269665	0.097723296
974	TMEM55B	ENSG00000165782	14:20459733-20460191:-	6.07E-07	0.386688846	0.294086234
975	TMEM55B	ENSG00000165782	14:20460298-20460654:-	0.00076423	0.433868122	0.375956364
976	METTL17	ENSG00000165792	14:20990383-20990463:+	0.04491252	0.250165627	0.214797223
977	METTL17	ENSG00000165792	14:20992205-20992540:+	7.10E-06	0.109796009	0.06929261
978	METTL17	ENSG00000165792	14:20992622-20993117:+	1.29E-05	0.173052357	0.12098979
979	METTL17	ENSG00000165792	14:20993191-20993968:+	0.01477367	0.251208173	0.209262527
980	METTL17	ENSG00000165792	14:20994901-20995164:+	0.00093862	0.303234313	0.357824442
981	METTL17	ENSG00000165792	14:20995951-20996208:+	0.00077808	0.501741646	0.43315187
982	METTL17	ENSG00000165792	14:20996292-20996526:+	0.00074465	0.152284093	0.117730142
983	NDRG2	ENSG00000165795	14:21018239-21018762:-	0.00074082	0.016538718	0.01268688
984	ARHGEF40	ENSG00000165801	14:21076643-21076773:+	0.00015686	0.069721891	0.104910752
985	ARHGEF40	ENSG00000165801	14:21076890-21078176:+	0.01683462	0.098582123	0.118320839
986	ARHGEF40	ENSG00000165801	14:21085866-21087000:+	0.00092452	0.302745134	0.246317173
987	ARHGEF40	ENSG00000165801	14:21088098-21088829:+	9.87E-11	0.260515548	0.177264671
988	METTL3	ENSG00000165819	14:21499802-21500494:-	0.0036084	0.186102491	0.152586479
989	METTL3	ENSG00000165819	14:21500682-21500912:-	0.0170138	0.218790629	0.178371027
990	MRPL52	ENSG00000172590	14:22829940-22830049:+	1.31E-07	0.325208296	0.232215616
991	MRPL52	ENSG00000172590	14:22830254-22833417:+	7.30E-05	0.090559321	0.073940653
992	CDH24	ENSG00000139880	14:23048479-23049026:-	5.88E-10	0.226014302	0.139560732
993	CDH24	ENSG00000139880	14:23049275-23049626:-	0.00096908	0.155270345	0.109062068
994	CDH24	ENSG00000139880	14:23049738-23049821:-	0.01712233	0.136583113	0.104939858
995	BCL2L2-PA	ENSG00000258643	14:23324289-23325246:+	0.00118411	0.427414305	0.389258714
996	SLC22A17	ENSG00000092096	14:23347212-23347459:-	3.95E-05	0.056118526	0.041496625
997	SLC22A17	ENSG00000092096	14:23347731-23347890:-	0.00862134	0.198908206	0.176778429
998	SLC22A17	ENSG00000092096	14:23347996-23348160:-	0.00225798	0.202061165	0.170439757
999	EFS	ENSG00000100842	14:23357660-23358875:-	0.00242381	0.105166876	0.077759268
1000	AP1G2	ENSG00000213983	14:23562066-23562287:-	0.01655016	0.102716987	0.087147319
1001	AP1G2	ENSG00000213983	14:23562593-23563199:-	0.00078853	0.102275478	0.09808877
1002	AP1G2	ENSG00000213983	14:23563502-23563583:-	0.00066058	0.236893378	0.179150401
1003	AP1G2	ENSG00000213983	14:23564159-23564332:-	0.0102331	0.07860535	0.070816636
1004	AP1G2	ENSG00000213983	14:23564660-23565118:-	6.50E-05	0.153701779	0.100162957
1005	AP1G2	ENSG00000213983	14:23565701-23566063:-	0.0019118	0.215551561	0.173997351
1006	AP1G2	ENSG00000213983	14:23566419-23566561:-	1.70E-07	0.228031201	0.177706609
1007	AP1G2	ENSG00000213983	14:23566686-23567110:-	4.01E-07	0.20980855	0.164642619
1008	CARMIL3	ENSG00000186648	14:24060756-24060926:+	0.03719491	0.207403578	0.172646523
1009	CARMIL3	ENSG00000186648	14:24065273-24065621:+	0.00478078	0.069030963	0.085108358
1010	CARMIL3	ENSG00000186648	14:24069247-24069378:+	0.04341747	0.079891698	0.09229896
1011	CPNE6	ENSG00000100884	14:24075276-24075504:+	0.00029239	0.2290559	0.14844906
1012	CPNE6	ENSG00000100884	14:24075591-24075826:+	0.00046994	0.172684811	0.113575247
1013	CPNE6	ENSG00000100884	14:24076282-24076359:+	2.12E-05	0.297100371	0.180696283
1014	CPNE6	ENSG00000100884	14:24076414-24076505:+	1.60E-05	0.269075973	0.15987434
1015	CPNE6	ENSG00000100884	14:24076557-24076878:+	0.00029028	0.357439554	0.247239221
1016	DCAF11	ENSG00000100897	14:24123077-24123174:+	0.00390441	0.26737828	0.227224495
1017	PSME1	ENSG00000092010	14:24138560-24138735:+	4.59E-05	0.123658751	0.08991169
1018	PSME2	ENSG00000100911	14:24144468-24145057:-	4.53E-10	0.155868367	0.091413965
1019	PSME2	ENSG00000100911	14:24146240-24146533:-	6.88E-08	0.110578506	0.068632999
1020	RNF31	ENSG00000092098	14:24150448-24150597:+	0.0129576	0.131031915	0.171084804
1021	RNF31	ENSG00000092098	14:24150888-24151130:+	0.00026288	0.140146446	0.199006464
1022	RNF31	ENSG00000092098	14:24157638-24157897:+	0.0013487	0.199884773	0.156955549
1023	RNF31	ENSG00000092098	14:24158011-24158141:+	4.80E-05	0.308982841	0.243128766
1024	REC8	ENSG00000100918	14:24179726-24179799:+	0.00093715	0.10217608	0.077238445
1025	CHMP4A	ENSG00000254505	14:24210768-24211410:-	0.03237013	0.038948414	0.035823998

S/N	Gene	Ensembl ID	Position of retained intron	p-value	AD_IR_average	Cont_IR_average
1026	MDP1	ENSG00000213920	14:24214395-24214491:-	0.03855494	0.130597573	0.112690784
1027	MDP1	ENSG00000213920	14:24215662-24215736:-	8.14E-06	0.55301122	0.444962
1028	GMPR2	ENSG00000100938	14:24238405-24238588:+	0.00698188	0.137105315	0.169288816
1029	TINF2	ENSG00000092330	14:24241994-24242140:-	0.01149255	0.087824709	0.12415239
1030	TGM1	ENSG00000092295	14:24249541-24254151:-	0.00580268	0.126111216	0.105590339
1031	RABGGTA	ENSG00000100949	14:24266517-24266775:-	0.02594248	0.258533098	0.224488556
1032	RABGGTA	ENSG00000100949	14:24267958-24268109:-	0.04824423	0.117798172	0.095063191
1033	RABGGTA	ENSG00000100949	14:24268198-24268368:-	0.00046259	0.406811085	0.366014727
1034	RABGGTA	ENSG00000100949	14:24268619-24268724:-	0.01273224	0.076286576	0.106979009
1035	RABGGTA	ENSG00000100949	14:24268826-24268910:-	0.01023279	0.163565026	0.136822119
1036	DHRS1	ENSG00000157379	14:24291219-24291555:-	0.01111113	0.460835939	0.453755247
1037	DHRS1	ENSG00000157379	14:24291625-24292183:-	5.33E-05	0.346953354	0.29373441
1038	KHNYN	ENSG00000100441	14:24432610-24432721:+	0.02057362	0.090634277	0.109897703
1039	SDR39U1	ENSG00000100445	14:24442260-24442345:-	0.00094105	0.178376139	0.161050465
1040	PPP2R3C	ENSG00000092020	14:35096633-35096708:-	0.00082672	0.275776694	0.245729662
1041	PPP2R3C	ENSG00000092020	14:35096764-35099251:-	0.00307769	0.122103923	0.114755779
1042	MBIP	ENSG00000151332	14:36311725-36311958:-	0.01738166	0.102707171	0.088049561
1043	PRPF39	ENSG00000185246	14:45114614-45114856:+	0.00344918	0.112754613	0.088491781
1044	FKBP3	ENSG00000100442	14:45130800-45134348:-	0.00266049	0.388232878	0.322556065
1045	KLHDC2	ENSG00000165516	14:49779675-49779747:+	0.00449786	0.457600183	0.419771662
1046	KLHDC2	ENSG00000165516	14:49779806-49780212:+	0.00075599	0.076483306	0.10772161
1047	KLHDC2	ENSG00000165516	14:49782457-49782541:+	0.01066334	0.090699476	0.104340492
1048	KLHDC2	ENSG00000165516	14:49782594-49782829:+	0.00825886	0.09019391	0.102599979
1049	PSMA3	ENSG00000100567	14:58270485-58270933:+	0.00820939	0.134790128	0.107726029
1050	PSMA3	ENSG00000100567	14:58270998-58271850:+	0.00045035	0.129915648	0.099815166
1051	ARID4A	ENSG00000032219	14:58347117-58347646:+	0.00025106	0.088495212	0.165484424
1052	ARID4A	ENSG00000032219	14:58367029-58371885:+	0.00262017	0.113284955	0.078029438
1053	MTHFD1	ENSG00000100714	14:64442402-64444692:+	0.00027355	0.385801695	0.296478935
1054	MTHFD1	ENSG00000100714	14:64444734-64448216:+	0.00441986	0.380607146	0.321509468
1055	PLEKHG3	ENSG00000126822	14:64742455-64742981:+	0.00051846	0.331102885	0.264206096
1056	CHURC1-FI	ENSG00000125954	14:65053349-65054574:+	0.00099997	0.204175271	0.160056162
1057	CHURC1-FI	ENSG00000125954	14:65054689-65061180:+	2.05E-06	0.207864013	0.144662168
1058	PLEKHH1	ENSG00000054690	14:67579311-67579720:+	0.01403811	0.389424634	0.338442195
1059	COQ6	ENSG00000119723	14:73959078-73959161:+	0.00362414	0.24661457	0.192246558
1060	ABCD4	ENSG00000119688	14:74287886-74288206:-	0.00817519	0.298208439	0.254344138
1061	ABCD4	ENSG00000119688	14:74290499-74292286:-	5.37E-10	0.59937761	0.463063701
1062	ABCD4	ENSG00000119688	14:74295198-74295853:-	7.77E-05	0.22347527	0.160228853
1063	ABCD4	ENSG00000119688	14:74295979-74296332:-	0.00028099	0.264405741	0.193981495
1064	ABCD4	ENSG00000119688	14:74296449-74297929:-	2.56E-05	0.471818244	0.407585195
1065	ABCD4	ENSG00000119688	14:74298069-74299547:-	0.02206678	0.33167922	0.325272753
1066	DLST	ENSG00000119689	14:74889349-74889896:+	0.02523923	0.0989069	0.104188801
1067	DLST	ENSG00000119689	14:74889952-74891055:+	0.00570325	0.084054026	0.074856718
1068	DLST	ENSG00000119689	14:74891167-74892833:+	0.01097355	0.313682	0.27544074
1069	VASH1	ENSG00000071246	14:76763130-76769962:+	0.04311101	0.1672365	0.137534517
1070	TMEM63C	ENSG00000165548	14:77251898-77253304:+	0.000206	0.092093935	0.062749131
1071	POMT2	ENSG00000009830	14:77283873-77284949:-	4.00E-06	0.318584793	0.238806897
1072	PSMC1	ENSG00000100764	14:90270352-90272272:+	0.00025952	0.185663267	0.142235275
1073	C14orf159	ENSG00000133943	14:91215556-91224683:+	2.21E-09	0.170137959	0.109718178
1074	C14orf159	ENSG00000133943	14:91223795-91224683:+	0.00014974	0.210608643	0.161847792
1075	PPP4R3A	ENSG00000100796	14:91465419-91466260:-	0.00028081	0.022599237	0.018688965
1076	LGMN	ENSG00000100600	14:92704361-92704639:-	0.01374184	0.187756176	0.166649121
1077	LGMN	ENSG00000100600	14:92706653-92709671:-	0.00010319	0.085274933	0.056199927
1078	UNC79	ENSG00000133958	14:93691946-93694334:+	0.00017917	0.09727479	0.067325039
1079	UNC79	ENSG00000133958	14:93704666-93706703:+	0.00017986	0.117102567	0.0849498
1080	PAPOLA	ENSG00000090060	14:96532649-96534490:+	0.00061419	0.28084422	0.383749974
1081	EVL	ENSG00000196405	14:100137644-100137739:+	3.48E-09	0.198040148	0.151393496
1082	BEGAIN	ENSG00000183092	14:100540579-100543857:-	0.01701279	0.298351616	0.257848574

S/N	Gene	Ensembl ID	Position of retained intron	p-value	AD_IR_average	Cont_IR_average
1083	MOK	ENSG00000080823	14:102231821-102232534:-	0.03501439	0.073997627	0.068380173
1084	CDC42BPB	ENSG00000198752	14:102940324-102943890:-	0.02328924	0.189702224	0.170805256
1085	MARK3	ENSG00000075413	14:103467191-103468032:+	2.38E-09	0.062196467	0.152661616
1086	MARK3	ENSG00000075413	14:103491107-103491776:+	0.00325202	0.226303878	0.220926299
1087	MARK3	ENSG00000075413	14:103492034-103498501:+	0.00483064	0.039846251	0.031308218
1088	CKB	ENSG00000166165	14:103521434-103521817:-	0.00110156	0.102626721	0.074288486
1089	ZFYVE21	ENSG00000100711	14:103715979-103726791:+	0.0352399	0.096070514	0.075244356
1090	ZFYVE21	ENSG00000100711	14:103727914-103728907:+	0.00324721	0.147165009	0.116852997
1091	ZFYVE21	ENSG00000100711	14:103729182-103729792:+	0.00026599	0.288016378	0.23266766
1092	ZFYVE21	ENSG00000100711	14:103729182-103732619:+	0.03221083	0.106147979	0.092955066
1093	PPP1R13B	ENSG00000088808	14:103738812-103738885:-	0.00166564	0.097338083	0.072531564
1094	TMEM179	ENSG00000258986	14:104595243-104596989:-	0.00991686	0.139653998	0.110896944
1095	AKT1	ENSG00000142208	14:104770847-104772364:-	0.04895798	0.105073867	0.092847296
1096	CEP170B	ENSG00000099814	14:104876345-104877884:+	0.0016387	0.15091191	0.202009083
1097	JAG2	ENSG00000184916	14:105146724-105147325:-	0.00436358	0.281549896	0.213498635
1098	BRF1	ENSG00000185024	14:105211293-105212112:-	0.00336019	0.101133533	0.124369931
1099	BRF1	ENSG00000185024	14:105226290-105226633:-	0.01056314	0.083127698	0.104846079
1100	PACS2	ENSG00000179364	14:105382581-105382806:+	5.68E-07	0.093507628	0.04459614
1101	PACS2	ENSG00000179364	14:105382913-105383358:+	0.00033304	0.120002252	0.08148191
1102	PACS2	ENSG00000179364	14:105384987-105389960:+	0.00031016	0.397995366	0.367585065
1103	PACS2	ENSG00000179364	14:105390003-105391206:+	0.00683252	0.278038915	0.231026897
1104	PACS2	ENSG00000179364	14:105391249-105391630:+	0.00013124	0.364807146	0.300368701
1105	LPCAT4	ENSG00000176454	15:34359745-34360110:-	0.00092524	0.555724037	0.475000208
1106	LPCAT4	ENSG00000176454	15:34362321-34362572:-	1.20E-05	0.198896021	0.134627997
1107	LPCAT4	ENSG00000176454	15:34362655-34362781:-	2.86E-06	0.172493524	0.108941879
1108	LPCAT4	ENSG00000176454	15:34362836-34363421:-	0.00023367	0.406421671	0.323843688
1109	LPCAT4	ENSG00000176454	15:34363456-34363660:-	0.02400714	0.28726678	0.251048727
1110	EIF2AK4	ENSG00000128829	15:40032237-40032756:+	0.00996118	0.138018523	0.109528275
1111	EIF2AK4	ENSG00000128829	15:40034444-40035026:+	0.00040094	0.22902921	0.181820944
1112	IVD	ENSG00000128928	15:40415482-40416077:+	0.00318996	0.089499268	0.070379348
1113	IVD	ENSG00000128928	15:40416362-40418129:+	7.63E-05	0.152207477	0.107735927
1114	RMDN3	ENSG00000137824	15:40738576-40740132:-	0.00356427	0.17782717	0.153001113
1115	DNAJC17	ENSG00000104129	15:40774436-40775030:-	0.0411313	0.092487217	0.078297547
1116	DNAJC17	ENSG00000104129	15:40775108-40775552:-	0.00901939	0.101363356	0.080231968
1117	ZFYVE19	ENSG00000166140	15:40809240-40809407:+	0.00011678	0.258176116	0.20613897
1118	ZFYVE19	ENSG00000166140	15:40809970-40810070:+	0.04660403	0.102478371	0.086647955
1119	ZFYVE19	ENSG00000166140	15:40813417-40813712:+	0.00653829	0.119792788	0.101732157
1120	ZFYVE19	ENSG00000166140	15:40814070-40814147:+	1.56E-07	0.271225037	0.182189657
1121	RTF1	ENSG00000137815	15:41475819-41476445:+	0.00199002	0.108189956	0.083449983
1122	TYRO3	ENSG00000092445	15:41573815-41577885:+	0.0036399	0.192576967	0.161462247
1123	MAPKBP1	ENSG00000137802	15:41813781-41814549:+	0.04140698	0.08877371	0.114944174
1124	MAPKBP1	ENSG00000137802	15:41814739-41815258:+	0.02310054	0.064247322	0.087404464
1125	MAPKBP1	ENSG00000137802	15:41819379-41819594:+	0.00021303	0.435608378	0.330871662
1126	MAPKBP1	ENSG00000137802	15:41819650-41820831:+	0.02418824	0.068101439	0.084055001
1127	MAPKBP1	ENSG00000137802	15:41824061-41824483:+	0.00017709	0.426213415	0.484509273
1128	JMJD7-PLA	ENSG00000168970	15:41841273-41841516:+	3.42E-05	0.317403866	0.24565146
1129	JMJD7-PLA	ENSG00000168970	15:41841571-41841818:+	0.00230582	0.292290585	0.244499722
1130	JMJD7-PLA	ENSG00000168970	15:41842276-41842553:+	0.0112346	0.555587427	0.495776078
1131	JMJD7-PLA	ENSG00000168970	15:41843811-41844470:+	0.00166408	0.294270918	0.21867557
1132	CDAN1	ENSG00000140326	15:42725251-42725488:-	0.04905157	0.179415798	0.157519705
1133	CDAN1	ENSG00000140326	15:42728033-42728203:-	0.0023163	0.260967134	0.219603661
1134	CDAN1	ENSG00000140326	15:42728267-42728651:-	0.0026174	0.281868052	0.230018835
1135	CDAN1	ENSG00000140326	15:42735178-42735260:-	0.01657453	0.157312624	0.132473865
1136	TUBGCP4	ENSG00000137822	15:43404552-43405201:+	0.01036202	0.584462683	0.657645597
1137	TP53BP1	ENSG00000067369	15:43409096-43409646:-	0.00058338	0.525587232	0.592527519
1138	ELL3	ENSG00000128886	15:43774518-43774595:-	0.02265743	0.156227658	0.119642989
1139	SPG11	ENSG00000104133	15:44564698-44565853:-	0.0042861	0.122577445	0.098092912

S/N	Gene	Ensembl ID	Position of retained intron	p-value	AD_IR_average	Cont_IR_average
1140	SPG11	ENSG00000104133	15:44566009-44566216:-	0.0259589	0.165287479	0.142025222
1141	SPG11	ENSG00000104133	15:44573745-44574901:-	0.00882405	0.100958949	0.084325326
1142	GATM	ENSG00000171766	15:45362221-45363899:-	0.01136901	0.133737618	0.109557392
1143	SPATA5L1	ENSG00000171763	15:45415779-45417268:+	0.00103959	0.09609763	0.067607329
1144	SPATA5L1	ENSG00000171763	15:45418682-45421042:+	0.00095424	0.147996277	0.114468848
1145	CCPG1	ENSG00000260916	15:55356409-55359538:-	0.00018919	0.25221705	0.169774597
1146	VPS13C	ENSG00000129003	15:61867947-61868658:-	0.00011309	0.293783387	0.191201965
1147	TLN2	ENSG00000171914	15:62836073-62838855:+	1.07E-05	0.090034851	0.056138365
1148	TPM1	ENSG00000140416	15:63060939-63061197:+	0.00530192	0.186894572	0.146041418
1149	TPM1	ENSG00000140416	15:63062645-63064063:+	6.37E-05	0.129074763	0.107325287
1150	HERC1	ENSG00000103657	15:63632811-63633847:-	0.0012004	0.140870567	0.098343275
1151	SNX1	ENSG00000028528	15:64136932-64137567:+	0.00534277	0.113677551	0.093240549
1152	UBAP1L	ENSG00000246922	15:65093231-65094474:-	0.00011	0.284406512	0.220256875
1153	SNAPC5	ENSG00000174446	15:66495419-66497641:-	1.15E-05	0.06495248	0.043368201
1154	C15orf61	ENSG00000189227	15:67521594-67526417:+	0.00098794	0.25035343	0.299692651
1155	CORO2B	ENSG00000103647	15:68711706-68713924:+	0.000956	0.291152866	0.241857778
1156	CORO2B	ENSG00000103647	15:68718810-68719143:+	0.0174499	0.314560878	0.300107208
1157	PKM	ENSG00000067225	15:72200655-72202453:-	8.61E-07	0.169872321	0.130068197
1158	PKM	ENSG00000067225	15:72200655-72203021:-	0.00047923	0.161864591	0.120015245
1159	PARP6	ENSG00000137817	15:72250092-72250844:-	0.01217559	0.114504766	0.109714745
1160	PARP6	ENSG00000137817	15:72250954-72251206:-	0.01491551	0.076002573	0.070670807
1161	HEXA	ENSG00000213614	15:72344140-72345445:-	8.59E-10	0.335844246	0.232887138
1162	HEXA	ENSG00000213614	15:72346325-72346526:-	2.53E-10	0.07977564	0.045347814
1163	HEXA	ENSG00000213614	15:72346710-72347685:-	7.59E-05	0.139704361	0.106351955
1164	CLK3	ENSG00000179335	15:74622560-74624901:+	0.00021762	0.125668594	0.098673614
1165	CSK	ENSG00000103653	15:74798312-74798614:+	0.01050027	0.103808254	0.127360213
1166	CSK	ENSG00000103653	15:74802083-74802330:+	0.00049098	0.158172112	0.119259769
1167	ULK3	ENSG00000140474	15:74837798-74838151:-	0.00465479	0.242375195	0.208473448
1168	ULK3	ENSG00000140474	15:74838192-74838265:-	0.00094379	0.186446551	0.147198161
1169	ULK3	ENSG00000140474	15:74839050-74839267:-	0.00013977	0.111641843	0.075962875
1170	ULK3	ENSG00000140474	15:74839373-74839557:-	5.85E-05	0.179829494	0.121173601
1171	ULK3	ENSG00000140474	15:74839713-74840233:-	0.00831123	0.113424479	0.085216342
1172	SCAMP2	ENSG00000140497	15:74845593-74848599:-	0.00106486	0.174075633	0.129597449
1173	COMMD4	ENSG00000140365	15:75338685-75338984:+	0.01619989	0.165407459	0.132644849
1174	COMMD4	ENSG00000140365	15:75339104-75339263:+	0.04754197	0.307465537	0.262225506
1175	COMMD4	ENSG00000140365	15:75339344-75339701:+	0.02366513	0.469427256	0.404553857
1176	NEIL1	ENSG00000140398	15:75352387-75352601:+	0.02566314	0.095009701	0.117942181
1177	NEIL1	ENSG00000140398	15:75354818-75354963:+	0.00102467	0.179707959	0.147166292
1178	MAN2C1	ENSG00000140400	15:75358808-75359058:-	0.00059966	0.107759535	0.077577944
1179	RCN2	ENSG00000117906	15:76948552-76949069:+	0.03894047	0.212145354	0.184140987
1180	RASGRF1	ENSG00000058335	15:78973420-78980619:-	0.01555279	0.07372694	0.082807706
1181	AP3B2	ENSG00000103723	15:82661922-82662167:-	2.43E-07	0.14822296	0.09474597
1182	AP3B2	ENSG00000103723	15:82662252-82662693:-	3.29E-06	0.43960111	0.356833416
1183	AP3B2	ENSG00000103723	15:82662922-82663126:-	1.59E-06	0.23257811	0.169464756
1184	AP3B2	ENSG00000103723	15:82663233-82663559:-	0.00014105	0.234096382	0.18420881
1185	AP3B2	ENSG00000103723	15:82663620-82663800:-	4.36E-05	0.16412157	0.122837906
1186	AP3B2	ENSG00000103723	15:82678167-82679728:-	0.00570065	0.114792971	0.093827965
1187	ZNF592	ENSG00000166716	15:84798045-84798314:+	0.00057232	0.300871521	0.24726953
1188	ZNF592	ENSG00000166716	15:84798474-84798587:+	0.00892281	0.098668661	0.081653238
1189	SEMA4B	ENSG00000185033	15:90217602-90217766:+	0.0026072	0.143185709	0.109144983
1190	SEMA4B	ENSG00000185033	15:90221093-90221366:+	2.68E-05	0.095591385	0.066836419
1191	CIB1	ENSG00000185043	15:90231022-90231094:-	0.01193883	0.109119444	0.082531423
1192	FURIN	ENSG00000140564	15:90877024-90877134:+	0.0217677	0.166829645	0.133541049
1193	FURIN	ENSG00000140564	15:90877211-90877526:+	0.03210652	0.138813556	0.124385645
1194	FURIN	ENSG00000140564	15:90877615-90878131:+	0.00035433	0.141732505	0.10947151
1195	FURIN	ENSG00000140564	15:90879984-90880093:+	0.0369563	0.143280163	0.1209252
1196	MAN2A2	ENSG00000196547	15:90912279-90912541:+	0.01234447	0.269307659	0.332315844

S/N	Gene	Ensembl ID	Position of retained intron	p-value	AD_IR_average	Cont_IR_average
1197	MAN2A2	ENSG00000196547	15:90913406-90913613:+	0.03555035	0.123075491	0.150330012
1198	MAN2A2	ENSG00000196547	15:90916256-90918193:+	0.00031613	0.126260513	0.172717955
1199	UNC45A	ENSG00000140553	15:90940473-90942436:+	0.00100642	0.102531107	0.14363643
1200	UNC45A	ENSG00000140553	15:90948283-90948653:+	2.22E-07	0.411779524	0.312749266
1201	VPS33B	ENSG00000184056	15:91005119-91005379:-	2.66E-09	0.238449776	0.169273619
1202	VPS33B	ENSG00000184056	15:91005454-91005693:-	0.00203053	0.230877134	0.18465421
1203	VPS33B	ENSG00000184056	15:91005784-91005972:-	1.56E-05	0.117612422	0.078238309
1204	SYNM	ENSG00000182253	15:99131813-99132749:+	0.01029716	0.390226402	0.317272487
1205	LINS1	ENSG00000140471	15:100570117-100571893:-	0.01932814	0.124020711	0.093687399
1206	SNRPA1	ENSG00000131876	15:101285801-101286907:-	0.01954163	0.23013396	0.253602013
1207	TM2D3	ENSG00000184277	15:101645162-101646724:-	0.00060094	0.101895035	0.138814938
1208	RHBDF1	ENSG00000007384	16:58759-58973:-	0.00034972	0.181529085	0.115329676
1209	NPRL3	ENSG00000103148	16:88890-89712:-	1.56E-05	0.152191538	0.108719423
1210	NPRL3	ENSG00000103148	16:89902-92595:-	0.00385574	0.186358494	0.152750305
1211	NPRL3	ENSG00000103148	16:92725-93218:-	0.04875004	0.168571849	0.143005327
1212	NPRL3	ENSG00000103148	16:125073-130521:-	0.00488307	0.029416126	0.034272812
1213	RGS11	ENSG00000076344	16:271099-271201:-	0.00057931	0.135464555	0.094654742
1214	RGS11	ENSG00000076344	16:271315-271398:-	0.00896847	0.115638815	0.088233805
1215	RGS11	ENSG00000076344	16:271569-272862:-	0.03415696	0.293032524	0.258335597
1216	RGS11	ENSG00000076344	16:272931-273474:-	0.00283825	0.215289283	0.167598135
1217	RGS11	ENSG00000076344	16:275082-275282:-	1.34E-06	0.478365329	0.35081274
1218	PDIA2	ENSG00000185615	16:284593-284658:+	0.0006716	0.407863088	0.295846104
1219	PDIA2	ENSG00000185615	16:284792-284877:+	0.02733675	0.253337634	0.204892714
1220	PDIA2	ENSG00000185615	16:285015-285083:+	0.00016123	0.442707207	0.345622403
1221	PDIA2	ENSG00000185615	16:285200-285311:+	0.0039286	0.369919683	0.305040649
1222	AXIN1	ENSG00000103126	16:289607-293487:-	0.00035723	0.275877354	0.237241362
1223	AXIN1	ENSG00000103126	16:298251-304303:-	0.04327032	0.076558229	0.088172927
1224	MRPL28	ENSG00000086504	16:368635-369067:-	0.00081367	0.146863217	0.117532319
1225	TMEM8A	ENSG00000129925	16:374151-374220:-	1.00E-04	0.109273159	0.070303801
1226	TMEM8A	ENSG00000129925	16:376455-376543:-	9.57E-06	0.21015101	0.301658723
1227	TMEM8A	ENSG00000129925	16:376812-377036:-	0.0001438	0.190459393	0.124532945
1228	RAB11FIP3	ENSG00000090565	16:489000-496823:+	0.00768372	0.137402988	0.134768908
1229	RAB11FIP3	ENSG00000090565	16:496859-503003:+	0.0015967	0.149558622	0.189885458
1230	CAPN15	ENSG00000103326	16:551664-552050:+	4.75E-06	0.356892024	0.252843071
1231	PIGQ	ENSG00000007541	16:578505-578784:+	0.00133657	0.258697763	0.194485866
1232	PIGQ	ENSG00000007541	16:578938-579068:+	0.0062602	0.149699157	0.11400869
1233	PIGQ	ENSG00000007541	16:580263-580857:+	0.02010027	0.433079854	0.377641581
1234	METTL26	ENSG00000130731	16:634797-634888:-	0.00684247	0.076510339	0.058488065
1235	METTL26	ENSG00000130731	16:635774-636093:-	0.00029059	0.141103183	0.12075761
1236	MCRIP2	ENSG00000172366	16:642043-642119:+	1.16E-06	0.106012757	0.062487585
1237	MCRIP2	ENSG00000172366	16:642249-646471:+	1.52E-05	0.278585456	0.224946021
1238	MCRIP2	ENSG00000172366	16:646608-647416:+	0.00039322	0.277772926	0.230517649
1239	MCRIP2	ENSG00000172366	16:647544-647782:+	4.05E-05	0.27166278	0.206544197
1240	MCRIP2	ENSG00000172366	16:647884-648119:+	0.01227372	0.243006183	0.204004477
1241	WDR90	ENSG00000161996	16:666792-666904:+	0.01416932	0.139845932	0.120993278
1242	RHOT2	ENSG00000140983	16:668236-668352:+	0.00804935	0.105649606	0.079807913
1243	RHOT2	ENSG00000140983	16:670175-670248:+	0.00231101	0.138780611	0.100729505
1244	RHOT2	ENSG00000140983	16:670773-670891:+	0.00650986	0.512918524	0.452058883
1245	RHOT2	ENSG00000140983	16:672566-672702:+	0.04570144	0.254671838	0.212134797
1246	RHBDL1	ENSG00000103269	16:677388-677458:+	6.17E-07	0.649516256	0.495633039
1247	JMJD8	ENSG00000161999	16:682874-682952:-	0.0134723	0.174766146	0.138606805
1248	JMJD8	ENSG00000161999	16:683087-683166:-	0.03746538	0.19848096	0.165663157
1249	JMJD8	ENSG00000161999	16:683441-683529:-	0.00274443	0.453544159	0.392900117
1250	METRN	ENSG00000103260	16:715984-716932:+	3.82E-07	0.136599729	0.091203744
1251	FAM173A	ENSG00000103254	16:721436-721597:+	0.02539994	0.698584402	0.665452364
1252	FAM173A	ENSG00000103254	16:721941-722083:+	0.00036375	0.558653207	0.512937494
1253	FAM173A	ENSG00000103254	16:722134-722308:+	0.00176782	0.300407841	0.299140506

S/N	Gene	Ensembl ID	Position of retained intron	p-value	AD_IR_average	Cont_IR_average
1254	NARFL	ENSG00000103245	16:731000-731564:-	0.00875785	0.102139996	0.080528409
1255	RPUSD1	ENSG00000007376	16:786377-786826:-	0.00483112	0.214117465	0.176583561
1256	RPUSD1	ENSG00000007376	16:787179-787353:-	7.30E-09	0.244641129	0.181507503
1257	GNPTG	ENSG00000090581	16:1352306-1361742:+	0.02540089	0.204992035	0.185215261
1258	GNPTG	ENSG00000090581	16:1362320-1362451:+	2.24E-07	0.152523538	0.103362709
1259	CCDC154	ENSG00000197599	16:1434852-1435088:-	0.00804247	0.295526854	0.235741365
1260	CLCN7	ENSG00000103249	16:1449093-1449275:-	0.00800349	0.257461016	0.210439292
1261	CLCN7	ENSG00000103249	16:1449327-1450496:-	0.0012912	0.541511744	0.468672857
1262	TELO2	ENSG00000100726	16:1493605-1494245:+	0.00054763	0.06766872	0.092046244
1263	TELO2	ENSG00000100726	16:1507051-1507305:+	6.29E-07	0.36738454	0.243217606
1264	TELO2	ENSG00000100726	16:1507370-1507600:+	3.84E-08	0.273671473	0.179343199
1265	CRAMP1	ENSG00000007545	16:1641187-1652495:+	0.01202416	0.08379821	0.115835575
1266	CRAMP1	ENSG00000007545	16:1669165-1670663:+	0.00519432	0.178294301	0.226923822
1267	CRAMP1	ENSG00000007545	16:1670809-1673880:+	0.00340344	0.104062502	0.139430753
1268	MAPK8IP3	ENSG00000138834	16:1758159-1758977:+	0.01776556	0.077514627	0.097980661
1269	MAPK8IP3	ENSG00000138834	16:1758159-1759957:+	0.00070205	0.043276678	0.056675
1270	NME3	ENSG00000103024	16:1770993-1771069:-	0.01030766	0.198460099	0.161441945
1271	NME3	ENSG00000103024	16:1771410-1771488:-	3.84E-07	0.260650317	0.179500435
1272	SPSB3	ENSG00000162032	16:1778321-1778434:-	0.00358682	0.324392805	0.376372675
1273	NUBP2	ENSG00000095906	16:1786655-1787676:+	4.21E-05	0.258639451	0.251895597
1274	ZNF598	ENSG00000167962	16:2001191-2001582:-	0.00224665	0.081719426	0.101426268
1275	ZNF598	ENSG00000167962	16:2001700-2002205:-	0.01097403	0.212870039	0.254898403
1276	ZNF598	ENSG00000167962	16:2002423-2002520:-	0.00898814	0.094948739	0.120950158
1277	ZNF598	ENSG00000167962	16:2002732-2002990:-	0.00466103	0.08705972	0.117970252
1278	TSC2	ENSG00000103197	16:2054440-2055401:+	0.00133859	0.070021724	0.098827693
1279	TSC2	ENSG00000103197	16:2080377-2081594:+	0.00093917	0.071088517	0.094458249
1280	TSC2	ENSG00000103197	16:2081798-2083694:+	0.01492249	0.30205389	0.324099974
1281	TSC2	ENSG00000103197	16:2085026-2085229:+	0.00312491	0.260213372	0.187087371
1282	PKD1	ENSG00000008710	16:2114925-2115377:-	0.00648952	0.077015012	0.104985287
1283	RAB26	ENSG00000167964	16:2152885-2152988:+	0.04103382	0.253819707	0.207248626
1284	RAB26	ENSG00000167964	16:2153222-2153318:+	0.00036181	0.113141573	0.150616169
1285	CASKIN1	ENSG00000167971	16:2181929-2183645:-	0.02354134	0.081243949	0.097187992
1286	CASKIN1	ENSG00000167971	16:2186824-2186977:-	6.76E-06	0.275029037	0.188037656
1287	CASKIN1	ENSG00000167971	16:2187072-2187165:-	5.76E-07	0.64821622	0.536896597
1288	E4F1	ENSG00000167967	16:2228523-2229569:+	0.00560923	0.080704626	0.102457301
1289	E4F1	ENSG00000167967	16:2229675-2232170:+	2.77E-05	0.170676411	0.229051618
1290	E4F1	ENSG00000167967	16:2233647-2233881:+	0.00245425	0.193145177	0.152915283
1291	ABCA3	ENSG00000167972	16:2283358-2284278:-	0.02941807	0.234658207	0.216608039
1292	ABCA3	ENSG00000167972	16:2304150-2308449:-	0.00047366	0.277039999	0.2325111
1293	TBC1D24	ENSG00000162065	16:2499420-2499834:+	0.00260234	0.339458317	0.300547153
1294	KCTD5	ENSG00000167977	16:2702478-2707297:+	0.03000415	0.126001076	0.105439595
1295	HCFC1R1	ENSG00000103145	16:3023361-3023846:-	0.00011135	0.149886229	0.170564047
1296	THOC6	ENSG00000131652	16:3026413-3026515:+	0.0216727	0.097080306	0.070937314
1297	ZNF205	ENSG00000122386	16:3116547-3118904:+	0.04545113	0.116903795	0.133878669
1298	NAA60	ENSG00000122390	16:3485061-3485466:+	0.00033021	0.292210073	0.348929
1299	CLUAP1	ENSG00000103351	16:3532841-3536121:+	7.30E-05	0.131943278	0.160232244
1300	SLX4	ENSG00000188827	16:3582693-3583096:-	0.03249684	0.299388585	0.247217468
1301	TRAP1	ENSG00000126602	16:3658230-3658792:-	1.38E-12	0.156447287	0.094936027
1302	TRAP1	ENSG00000126602	16:3658865-3661986:-	5.43E-11	0.207960327	0.13412497
1303	TRAP1	ENSG00000126602	16:3662132-3662881:-	7.77E-05	0.181714923	0.139223873
1304	TRAP1	ENSG00000126602	16:3662967-3663423:-	2.88E-06	0.236539063	0.180969465
1305	TRAP1	ENSG00000126602	16:3663562-3664273:-	1.30E-05	0.23374514	0.180782082
1306	CREBBP	ENSG00000005339	16:3736815-3738558:-	0.00015535	0.103895344	0.187559617
1307	GLIS2	ENSG00000126603	16:4335393-4336724:+	0.02193243	0.115134566	0.140055205
1308	MGRN1	ENSG00000102858	16:4673657-4677462:+	0.03691161	0.073459138	0.06765986
1309	MGRN1	ENSG00000102858	16:4680097-4681549:+	0.00748683	0.11671927	0.123592095
1310	MGRN1	ENSG00000102858	16:4686303-4688795:+	0.00901305	0.498778622	0.459967078

S/N	Gene	Ensembl ID	Position of retained intron	p-value	AD_IR_average	Cont_IR_average
1311	ANKS3	ENSG00000168096	16:4714186-4724749:-	0.00870921	0.068993506	0.081940174
1312	ROGDI	ENSG00000067836	16:4801321-4801502:-	0.00051688	0.219318852	0.205702732
1313	GLYR1	ENSG00000140632	16:4812248-4813736:-	0.00074532	0.112989685	0.089712047
1314	GLYR1	ENSG00000140632	16:4813838-4814536:-	0.01599277	0.128543759	0.10716081
1315	C16orf89	ENSG00000153446	16:5044478-5047877:-	0.00040993	0.101975633	0.068203293
1316	C16orf89	ENSG00000153446	16:5047964-5055245:-	4.04E-07	0.095718082	0.055881021
1317	CARHSP1	ENSG00000153048	16:8855326-8858349:-	0.01363467	0.088360511	0.112310257
1318	CARHSP1	ENSG00000153048	16:8858472-8859170:-	0.0386186	0.175043962	0.206117375
1319	USP7	ENSG00000187555	16:8902187-8902380:-	0.03059011	0.154168139	0.119584595
1320	NUBP1	ENSG00000103274	16:10756780-10757872:+	0.01833931	0.203423962	0.175293196
1321	ZC3H7A	ENSG00000122299	16:11761511-11761909:-	0.00173629	0.180843237	0.13705084
1322	ZC3H7A	ENSG00000122299	16:11762043-11762670:-	0.00279242	0.1981677	0.156253383
1323	ZC3H7A	ENSG00000122299	16:11762747-11763477:-	0.03239681	0.14277724	0.124429255
1324	KIAA0430	ENSG00000166783	16:15600701-15601990:-	0.00883006	0.091056614	0.15097894
1325	COQ7	ENSG00000167186	16:19075860-19077305:+	1.34E-06	0.120325796	0.091381738
1326	COQ7	ENSG00000167186	16:19077374-19078080:+	3.09E-06	0.123872885	0.091467265
1327	CCP110	ENSG00000103540	16:19546474-19547954:+	0.03493259	0.224519584	0.197585532
1328	C16orf62	ENSG00000103544	16:19691471-19699501:+	2.18E-07	0.121556907	0.087961775
1329	EEF2K	ENSG00000103319	16:22258695-22260461:+	7.57E-05	0.248021448	0.17545054
1330	GGA2	ENSG00000103365	16:23478501-23478882:-	0.00057269	0.167449135	0.137631732
1331	GTF3C1	ENSG00000077235	16:27463592-27464319:-	0.00140553	0.325066205	0.266211675
1332	GTF3C1	ENSG00000077235	16:27470395-27471747:-	4.76E-05	0.119192154	0.081597955
1333	CLN3	ENSG00000188603	16:28482198-28482326:-	2.52E-05	0.20432622	0.149517518
1334	SGF29	ENSG00000176476	16:28590935-28591589:+	1.28E-05	0.13708179	0.091593405
1335	ATXN2L	ENSG00000168488	16:28824542-28825365:+	0.04087993	0.101165876	0.09036141
1336	ATXN2L	ENSG00000168488	16:28833354-28833438:+	0.00012021	0.107966982	0.069751716
1337	ATXN2L	ENSG00000168488	16:28833508-28834064:+	0.00011308	0.11430298	0.075348649
1338	SH2B1	ENSG00000178188	16:28873269-28873446:+	0.00025689	0.078692828	0.059709965
1339	NFATC2IP	ENSG00000176953	16:28956058-28956150:+	0.03038917	0.467118927	0.418910597
1340	NFATC2IP	ENSG00000176953	16:28958861-28958990:+	0.00111926	0.179519178	0.13144264
1341	SPNS1	ENSG00000169682	16:28981615-28981900:+	2.73E-05	0.303693549	0.242452496
1342	SPNS1	ENSG00000169682	16:28982056-28982355:+	2.39E-05	0.314569695	0.242183639
1343	SPNS1	ENSG00000169682	16:28982545-28982856:+	5.98E-07	0.200075095	0.147100162
1344	SPNS1	ENSG00000169682	16:28982922-28983191:+	1.12E-08	0.099209906	0.058889562
1345	LAT	ENSG00000213658	16:28986569-28986656:+	0.0073177	0.116083038	0.078310298
1346	KIF22	ENSG00000079616	16:29803608-29803997:+	2.97E-06	0.129491644	0.082342969
1347	KIF22	ENSG00000079616	16:29805174-29805262:+	0.00090642	0.177295987	0.125791694
1348	CDIPT	ENSG00000103502	16:29860662-29861105:-	0.04808683	0.107762448	0.094753156
1349	ASPHD1	ENSG00000174939	16:29904965-29905787:+	1.51E-08	0.129572471	0.080276187
1350	KCTD13	ENSG00000174943	16:29911867-29911959:-	0.00029088	0.29554311	0.236281838
1351	TAOK2	ENSG00000149930	16:29989805-29990783:+	0.0405531	0.235785422	0.219234381
1352	HIRIP3	ENSG00000149929	16:29993557-29993639:-	0.0054911	0.218876549	0.180620616
1353	INO80E	ENSG00000169592	16:30001040-30005220:+	0.00426458	0.278503972	0.286193532
1354	PPP4C	ENSG00000149923	16:30076475-30081258:+	4.35E-06	0.169517226	0.125596862
1355	PPP4C	ENSG00000149923	16:30081310-30082483:+	0.00190613	0.132313173	0.106354699
1356	PPP4C	ENSG00000149923	16:30082534-30082745:+	0.02458056	0.123446207	0.105326904
1357	PPP4C	ENSG00000149923	16:30082847-30083393:+	0.00420947	0.266058037	0.224500284
1358	GDPD3	ENSG00000102886	16:30113064-30113339:-	0.02176215	0.290381756	0.345889805
1359	CORO1A	ENSG00000102879	16:30187501-30187724:+	0.00012837	0.17165828	0.127109783
1360	TBC1D10B	ENSG00000169221	16:30365006-30365104:-	0.00134206	0.094096289	0.072136309
1361	PRR14	ENSG00000156858	16:30652842-30652913:+	0.03166736	0.166542678	0.136676297
1362	PRR14	ENSG00000156858	16:30653408-30654229:+	0.04712875	0.116446671	0.103003792
1363	PRR14	ENSG00000156858	16:30654339-30654628:+	0.00142197	0.103452726	0.086838751
1364	FBRS	ENSG00000156860	16:30664920-30665034:+	0.00262391	0.179455094	0.139875919
1365	FBRS	ENSG00000156860	16:30665079-30665305:+	0.00066424	0.238644063	0.191630142
1366	FBRS	ENSG00000156860	16:30665706-30666511:+	0.00080818	0.108807507	0.080296193
1367	FBRS	ENSG00000156860	16:30666541-30666918:+	0.01126889	0.126308513	0.103571002

S/N	Gene	Ensembl ID	Position of retained intron	p-value	AD_IR_average	Cont_IR_average
1368	FBRS	ENSG00000156860	16:30666990-30667319:+	0.00957966	0.135102255	0.107335783
1369	FBRS	ENSG00000156860	16:30667622-30668559:+	0.00577618	0.133118738	0.108552626
1370	FBRS	ENSG00000156860	16:30668643-30668771:+	0.02733243	0.140840468	0.117218923
1371	PHKG2	ENSG00000156873	16:30751603-30753231:+	2.88E-06	0.247469874	0.175059164
1372	PHKG2	ENSG00000156873	16:30753297-30753393:+	0.00137438	0.142833984	0.113801004
1373	FBXL19	ENSG00000099364	16:30928499-30930072:+	7.60E-08	0.103119244	0.064702613
1374	BCKDK	ENSG00000103507	16:31109783-31110076:+	0.02452557	0.097914339	0.088618939
1375	FUS	ENSG00000089280	16:31185179-31186801:+	0.00453797	0.298760005	0.245599404
1376	FUS	ENSG00000089280	16:31186836-31188324:+	0.02302823	0.297998483	0.262016848
1377	FUS	ENSG00000089280	16:31189226-31189664:+	3.15E-08	0.191779959	0.113261944
1378	C16orf58	ENSG00000140688	16:31493524-31493602:-	0.02289004	0.588624732	0.532938922
1379	GNAO1	ENSG00000087258	16:56336860-56340833:+	0.00027781	0.049184902	0.047561969
1380	OGFOD1	ENSG00000087263	16:56467293-56467904:+	0.02029273	0.169015267	0.247930096
1381	BBS2	ENSG00000125124	16:56470614-56484760:-	5.60E-10	0.189930482	0.120657231
1382	COQ9	ENSG00000088682	16:57457015-57458245:+	3.06E-06	0.446595634	0.331582883
1383	COQ9	ENSG00000088682	16:57458350-57459564:+	4.23E-06	0.32689528	0.246829031
1384	COQ9	ENSG00000088682	16:57459720-57460050:+	0.00255272	0.177158089	0.144347179
1385	COQ9	ENSG00000088682	16:57460104-57460588:+	0.00066796	0.146003523	0.116943387
1386	POLR2C	ENSG00000102978	16:57470354-57470974:+	0.04149198	0.095033939	0.08388516
1387	KATNB1	ENSG00000140854	16:57752928-57753076:+	0.00116533	0.22803629	0.180337673
1388	KATNB1	ENSG00000140854	16:57753519-57753944:+	0.00020305	0.135945137	0.093689623
1389	KATNB1	ENSG00000140854	16:57756472-57756813:+	0.00011966	0.178396596	0.130276688
1390	KIFC3	ENSG00000140859	16:57758909-57759124:-	0.00231302	0.282408511	0.23386733
1391	NDRG4	ENSG00000103034	16:58500269-58500987:+	0.03294302	0.116767537	0.127670371
1392	NDRG4	ENSG00000103034	16:58509352-58511421:+	0.00023467	0.288447915	0.226696292
1393	SETD6	ENSG00000103037	16:58516672-58516807:+	0.00206219	0.26360681	0.328078522
1394	SETD6	ENSG00000103037	16:58518231-58518400:+	0.03951463	0.250958506	0.28696574
1395	CNOT1	ENSG00000125107	16:58521036-58521182:-	5.53E-09	0.272251279	0.184264086
1396	CNOT1	ENSG00000125107	16:58521317-58523369:-	1.70E-05	0.299460098	0.233408049
1397	SLC38A7	ENSG00000103042	16:58670167-58671044:-	0.03272217	0.112562237	0.087846644
1398	CKLF	ENSG00000217555	16:66563217-66565885:+	0.00565555	0.259537588	0.211431843
1399	NAE1	ENSG00000159593	16:66803118-66805776:-	3.68E-08	0.147564629	0.096772805
1400	NAE1	ENSG00000159593	16:66809075-66810373:-	0.01780857	0.208658378	0.165588148
1401	NAE1	ENSG00000159593	16:66810413-66810696:-	0.00438913	0.09721396	0.070798109
1402	FAM96B	ENSG00000166595	16:66932300-66932779:-	0.00054627	0.109606679	0.083961335
1403	CES4A	ENSG00000172824	16:67003553-67004083:+	0.01075923	0.155206777	0.114208468
1404	HSF4	ENSG00000102878	16:67164934-67165521:+	0.0002869	0.682480805	0.597618247
1405	HSF4	ENSG00000102878	16:67169101-67169278:+	5.36E-05	0.163007249	0.152840092
1406	HSF4	ENSG00000102878	16:67169348-67169630:+	0.03004507	0.25884178	0.317741078
1407	KIAA0895L	ENSG00000196123	16:67180666-67181557:-	0.00414227	0.093214995	0.084969948
1408	KIAA0895L	ENSG00000196123	16:67181697-67183261:-	0.021133	0.077944212	0.07149837
1409	E2F4	ENSG00000205250	16:67197646-67197866:+	3.92E-05	0.341890549	0.26630557
1410	ELMO3	ENSG00000102890	16:67203583-67203664:+	0.00404241	0.089415033	0.133516627
1411	FHOD1	ENSG00000135723	16:67229718-67229792:-	0.00536953	0.464285244	0.392895065
1412	FHOD1	ENSG00000135723	16:67229990-67230065:-	0.03591415	0.364707007	0.317843875
1413	SLC9A5	ENSG00000135740	16:67265106-67266087:+	6.14E-06	0.112428544	0.085370344
1414	ZDHHC1	ENSG00000159714	16:67399456-67400956:-	0.0019805	0.233597096	0.174109181
1415	AC027682.	ENSG00000283332	16:67546231-67546365:+	0.0004707	0.094154524	0.068492255
1416	ACD	ENSG00000102977	16:67657684-67657761:-	0.02177068	0.130104445	0.103906669
1417	TSNAXIP1	ENSG00000102904	16:67826844-67826962:+	0.00185194	0.178694767	0.130317651
1418	CENPT	ENSG00000102901	16:67828390-67828473:-	0.03197601	0.10651306	0.084260004
1419	NUTF2	ENSG00000102898	16:67868599-67870799:+	4.64E-05	0.117129743	0.086461819
1420	EDC4	ENSG00000038358	16:67880216-67880556:+	5.22E-05	0.113140943	0.159659838
1421	EDC4	ENSG00000038358	16:67881417-67881496:+	0.02225638	0.347076854	0.301282343
1422	EDC4	ENSG00000038358	16:67883731-67883955:+	0.00495386	0.22862943	0.176033925
1423	PSMB10	ENSG00000205220	16:67934948-67935419:-	3.05E-05	0.212169927	0.161198477
1424	PSMB10	ENSG00000205220	16:67935697-67935962:-	0.03265838	0.184703502	0.142745083

S/N	Gene	Ensembl ID	Position of retained intron	p-value	AD_IR_average	Cont_IR_average
1425	COG8	ENSG00000213380	16:69332882-69334520:-	0.00298264	0.099365063	0.076047443
1426	NPIPB14P	ENSG00000226232	16:69978112-69978221:-	0.03462036	0.17269635	0.147913686
1427	FUK	ENSG00000157353	16:70475767-70478271:+	0.00138649	0.279288371	0.336118636
1428	COG4	ENSG00000103051	16:70481487-70481763:-	1.09E-06	0.108667173	0.073498682
1429	MTSS1L	ENSG00000132613	16:70677899-70678251:-	0.0011109	0.08598006	0.05662465
1430	MTSS1L	ENSG00000132613	16:70678409-70679629:-	0.02127753	0.026945114	0.031733538
1431	MTSS1L	ENSG00000132613	16:70680055-70680793:-	0.02293106	0.127291496	0.098789078
1432	VAC14	ENSG00000103043	16:70698811-70731494:-	0.00636498	0.104960652	0.081055545
1433	IST1	ENSG00000182149	16:71922680-71923287:+	0.04667387	0.092784718	0.07374783
1434	IST1	ENSG00000182149	16:71923380-71924768:+	0.01071667	0.347991683	0.303511416
1435	DHX38	ENSG00000140829	16:72104131-72104485:+	0.01856031	0.210282961	0.262946052
1436	GLG1	ENSG00000090863	16:74462630-74463355:-	0.02478936	0.118895361	0.119386339
1437	WDR59	ENSG00000103091	16:74909657-74909821:-	0.00748581	0.577378683	0.536153987
1438	WDR59	ENSG00000103091	16:74956610-74965772:-	0.00056245	0.055049126	0.080908191
1439	ZNRF1	ENSG00000186187	16:75104889-75106481:+	0.04125367	0.19762318	0.172105701
1440	ZNRF1	ENSG00000186187	16:75106571-75107732:+	0.00156109	0.165688488	0.128150881
1441	LDHD	ENSG00000166816	16:75112713-75112833:-	0.00131399	0.226232257	0.150658447
1442	CMC2	ENSG00000103121	16:80997429-81006733:-	0.00122277	0.04975546	0.055766568
1443	MPHOSPH	ENSG00000135698	16:82151514-82164081:-	0.02323435	0.096228265	0.081209583
1444	MBTPS1	ENSG00000140943	16:84059428-84060681:-	0.00136782	0.234605857	0.184703618
1445	MBTPS1	ENSG00000140943	16:84060813-84063304:-	0.00563197	0.314146817	0.257945545
1446	MBTPS1	ENSG00000140943	16:84065767-84066488:-	0.00714009	0.137140479	0.098056003
1447	MBTPS1	ENSG00000140943	16:84066613-84067666:-	0.01954386	0.094555495	0.068133737
1448	COTL1	ENSG00000103187	16:84617583-84617837:-	0.00080028	0.32630442	0.255404377
1449	USP10	ENSG00000103194	16:84772685-84775159:+	0.00255685	0.099682099	0.07451754
1450	USP10	ENSG00000103194	16:84775225-84778894:+	0.00138894	0.097325629	0.073691999
1451	ZCCHC14	ENSG00000140948	16:87414541-87415275:-	0.01400632	0.129387791	0.102102768
1452	KLHDC4	ENSG00000104731	16:87708466-87709264:-	0.01149499	0.128331832	0.108480633
1453	KLHDC4	ENSG00000104731	16:87709667-87711234:-	0.01092557	0.248075376	0.220285625
1454	KLHDC4	ENSG00000104731	16:87726924-87730551:-	0.02880237	0.093300012	0.111052392
1455	MVD	ENSG00000167508	16:88653408-88654691:-	0.04960219	0.123060027	0.09968816
1456	CTU2	ENSG00000174177	16:88712383-88712621:+	0.00054929	0.127960407	0.094588489
1457	CTU2	ENSG00000174177	16:88713447-88713646:+	0.03447235	0.105517299	0.084501142
1458	APRT	ENSG00000198931	16:88811656-88811819:-	0.00587322	0.145357149	0.101372668
1459	TRAPPC2L	ENSG00000167515	16:88857183-88858618:+	2.68E-07	0.15038957	0.093281704
1460	ANKRD11	ENSG00000167522	16:89291183-89305205:-	0.01018449	0.125194372	0.153537351
1461	SPG7	ENSG00000197912	16:89529579-89530682:+	0.00061338	0.072877767	0.103417383
1462	SPG7	ENSG00000197912	16:89530808-89531903:+	0.00242913	0.265811829	0.204338265
1463	SPG7	ENSG00000197912	16:89548113-89550493:+	0.00640516	0.135999629	0.109887592
1464	CDK10	ENSG00000185324	16:89686797-89690552:+	0.0032318	0.11883998	0.136280951
1465	CDK10	ENSG00000185324	16:89693467-89694172:+	0.00567481	0.705379244	0.654544234
1466	CDK10	ENSG00000185324	16:89695070-89695292:+	0.00020365	0.097488982	0.069020131
1467	VPS9D1	ENSG00000075399	16:89707954-89708426:-	1.18E-06	0.357384205	0.254447962
1468	VPS9D1	ENSG00000075399	16:89708956-89709226:-	4.77E-05	0.251786709	0.18275031
1469	VPS9D1	ENSG00000075399	16:89711010-89711326:-	0.00103689	0.273341734	0.199709432
1470	VPS9D1	ENSG00000075399	16:89711969-89712046:-	0.01835386	0.07323551	0.086328207
1471	VPS9D1	ENSG00000075399	16:89712716-89716461:-	0.00936367	0.086801332	0.104100301
1472	VPS9D1	ENSG00000075399	16:89716624-89716729:-	0.00088219	0.078480406	0.107364047
1473	ZNF276	ENSG00000158805	16:89722834-89723136:+	0.02382356	0.143848973	0.167213517
1474	ZNF276	ENSG00000158805	16:89723183-89723259:+	0.04741154	0.194518363	0.215895586
1475	ZNF276	ENSG00000158805	16:89729318-89733301:+	0.00963089	0.179203634	0.151685712
1476	ZNF276	ENSG00000158805	16:89733412-89733481:+	0.00039405	0.41447528	0.326760987
1477	TCF25	ENSG00000141002	16:89895137-89895989:+	0.01540481	0.10258973	0.085177755
1478	TCF25	ENSG00000141002	16:89906284-89907242:+	0.0157526	0.137195276	0.114695686
1479	DEF8	ENSG00000140995	16:89962125-89963362:+	3.17E-09	0.536469232	0.381286805
1480	DEF8	ENSG00000140995	16:89963443-89964169:+	5.43E-09	0.63857211	0.499798286
1481	AC133919.	ENSG00000283182	16:90037399-90037761:+	0.0021058	0.130531172	0.101526884

S/N	Gene	Ensembl ID	Position of retained intron	p-value	AD_IR_average	Cont_IR_average
1482	ABR	ENSG00000159842	17:1007312-1009678:-	0.00706362	0.107772109	0.092131571
1483	ABR	ENSG00000159842	17:1009784-1010728:-	1.56E-06	0.107422501	0.074961018
1484	ABR	ENSG00000159842	17:1011985-1012687:-	0.00032242	0.096158477	0.078543379
1485	PRPF8	ENSG00000174231	17:1659548-1659840:-	0.04956332	0.097964384	0.142078631
1486	DPH1	ENSG00000108963	17:2036091-2036528:+	0.00059041	0.369800939	0.302747613
1487	DPH1	ENSG00000108963	17:2036956-2039754:+	0.0128361	0.586902817	0.535162104
1488	DPH1	ENSG00000108963	17:2039823-2040217:+	0.00186837	0.212277021	0.171587782
1489	SGSM2	ENSG00000141258	17:2364183-2364595:+	0.0068428	0.17185484	0.134103631
1490	SGSM2	ENSG00000141258	17:2364663-2364896:+	0.00014379	0.116211434	0.073092593
1491	SGSM2	ENSG00000141258	17:2372488-2372952:+	0.04673007	0.39677561	0.355258682
1492	SGSM2	ENSG00000141258	17:2373513-2375491:+	8.96E-05	0.368048768	0.297107944
1493	SGSM2	ENSG00000141258	17:2375875-2376136:+	0.00052142	0.130218172	0.0971078
1494	SGSM2	ENSG00000141258	17:2376261-2376732:+	0.00047977	0.132844278	0.096893194
1495	RAP1GAP2	ENSG00000132359	17:3026121-3026349:+	0.01411377	0.205424316	0.176301836
1496	CAMKK1	ENSG00000004660	17:3862283-3865907:-	0.00383858	0.16212577	0.209257665
1497	PELP1	ENSG00000141456	17:4675884-4676035:-	0.00076348	0.131591507	0.101974553
1498	PELP1	ENSG00000141456	17:4676812-4682501:-	0.0138204	0.08785062	0.113028736
1499	ARRB2	ENSG00000141480	17:4710744-4715012:+	0.00354255	0.142486935	0.134525497
1500	ARRB2	ENSG00000141480	17:4716033-4716146:+	0.02277908	0.24779404	0.2227488
1501	ARRB2	ENSG00000141480	17:4716191-4716411:+	0.04464494	0.279923013	0.260565186
1502	ARRB2	ENSG00000141480	17:4716608-4717216:+	5.41E-05	0.318482976	0.238698166
1503	ARRB2	ENSG00000141480	17:4718023-4718260:+	2.01E-05	0.347076671	0.321586065
1504	ARRB2	ENSG00000141480	17:4718345-4718611:+	2.13E-06	0.335967598	0.307229935
1505	ARRB2	ENSG00000141480	17:4720299-4720392:+	0.00684555	0.13234012	0.107240377
1506	ARRB2	ENSG00000141480	17:4720640-4720945:+	0.00226914	0.333836634	0.285581338
1507	PLD2	ENSG00000129219	17:4817055-4817145:+	0.00429503	0.32331522	0.274133682
1508	PLD2	ENSG00000129219	17:4818106-4818296:+	5.72E-06	0.646219183	0.562718403
1509	PLD2	ENSG00000129219	17:4818385-4818493:+	4.46E-05	0.38352372	0.327938097
1510	PLD2	ENSG00000129219	17:4819218-4819428:+	0.00284181	0.094012779	0.114615691
1511	MINK1	ENSG00000141503	17:4894311-4894524:+	0.02796884	0.223143702	0.189876786
1512	MINK1	ENSG00000141503	17:4894633-4895074:+	0.00848405	0.13477716	0.110577794
1513	MINK1	ENSG00000141503	17:4895832-4896002:+	0.02025228	0.158454709	0.134441399
1514	RNF167	ENSG00000108523	17:4942664-4942850:+	0.0007385	0.092604371	0.072682578
1515	SPAG7	ENSG00000091640	17:4959643-4959759:-	0.00060797	0.156849	0.124050575
1516	NUP88	ENSG00000108559	17:5386269-5386707:-	2.57E-05	0.120752772	0.088500642
1517	C17orf49	ENSG00000258315	17:7015156-7015773:+	0.04263456	0.162300326	0.160453164
1518	C17orf49	ENSG00000258315	17:7016685-7016909:+	0.0106175	0.124751456	0.108087532
1519	DLG4	ENSG00000132535	17:7204252-7204945:-	0.04683713	0.082781522	0.063646278
1520	ACADVL	ENSG00000072778	17:7220046-7220121:+	5.17E-05	0.287693476	0.221218474
1521	ACADVL	ENSG00000072778	17:7220529-7220603:+	0.00453279	0.133901393	0.104915561
1522	ACADVL	ENSG00000072778	17:7222081-7222176:+	0.01572415	0.701843512	0.662446169
1523	ACADVL	ENSG00000072778	17:7222302-7222666:+	0.02546891	0.539642561	0.496317013
1524	ACADVL	ENSG00000072778	17:7223875-7223967:+	5.73E-05	0.110503866	0.083982984
1525	ACADVL	ENSG00000072778	17:7224069-7224145:+	1.98E-05	0.112003677	0.080064216
1526	ACADVL	ENSG00000072778	17:7224243-7224320:+	6.72E-05	0.091668733	0.065621747
1527	DVL2	ENSG00000004975	17:7226639-7227089:-	2.87E-07	0.252612888	0.33332444
1528	DVL2	ENSG00000004975	17:7228044-7228968:-	0.0129271	0.141856129	0.11835684
1529	DVL2	ENSG00000004975	17:7229274-7229377:-	0.00025135	0.35821589	0.288682653
1530	DVL2	ENSG00000004975	17:7229447-7229587:-	1.77E-06	0.545981305	0.444963987
1531	DVL2	ENSG00000004975	17:7229678-7229807:-	6.49E-05	0.395173427	0.302192779
1532	EIF5A	ENSG00000132507	17:7309800-7311017:+	0.01486473	0.141593426	0.126515961
1533	GPS2	ENSG00000132522	17:7313291-7313379:-	0.00034543	0.199570885	0.15387004
1534	GPS2	ENSG00000132522	17:7313469-7313567:-	1.66E-07	0.120349118	0.080076829
1535	NEURL4	ENSG00000215041	17:7318172-7318268:-	0.00570775	0.201859467	0.236526156
1536	NEURL4	ENSG00000215041	17:7318356-7318494:-	0.00019827	0.126629355	0.162441756
1537	NEURL4	ENSG00000215041	17:7323123-7323484:-	0.00463294	0.309189732	0.273178652
1538	NLGN2	ENSG00000169992	17:7415148-7415510:+	2.32E-05	0.11104463	0.07507107

S/N	Gene	Ensembl ID	Position of retained intron	p-value	AD_IR_average	Cont_IR_average
1539	TNFSF13	ENSG00000161955	17:7559297-7559623:+	0.00637324	0.1823589	0.143923248
1540	TNFSF12-T	ENSG00000248871	17:7559702-7559845:+	0.00119639	0.251562233	0.19895699
1541	TNFSF12-T	ENSG00000248871	17:7559893-7560048:+	0.02825539	0.096629465	0.079839813
1542	TNFSF12-T	ENSG00000248871	17:7560167-7560349:+	0.00613382	0.111469684	0.092159284
1543	CD68	ENSG00000129226	17:7580585-7580710:+	0.04100295	0.443670689	0.366267131
1544	CD68	ENSG00000129226	17:7580783-7580895:+	0.00616917	0.547750716	0.45805153
1545	CD68	ENSG00000129226	17:7581066-7581377:+	0.00109398	0.464156754	0.359427592
1546	SAT2	ENSG00000141504	17:7627044-7627142:-	0.03540422	0.130979343	0.147611735
1547	WRAP53	ENSG00000141499	17:7702846-7702992:+	0.001783	0.167631121	0.126902104
1548	KDM6B	ENSG00000132510	17:7853380-7853497:+	0.00455738	0.512482512	0.431940571
1549	NAA38	ENSG00000183011	17:7856843-7856984:-	0.00413945	0.033835228	0.028402433
1550	CNTROB	ENSG00000170037	17:7944248-7944475:+	0.03833696	0.444085183	0.401688844
1551	CNTROB	ENSG00000170037	17:7947722-7947915:+	0.00328195	0.113412522	0.089414281
1552	CTC1	ENSG00000178971	17:8229446-8229890:-	0.01869574	0.198999889	0.237406718
1553	CTC1	ENSG00000178971	17:8234655-8234748:-	0.01201855	0.09781258	0.114452273
1554	RANGRF	ENSG00000108961	17:8289588-8289812:+	5.31E-07	0.580482866	0.543203156
1555	ARHGAP44	ENSG00000006740	17:12980233-12984530:+	0.03970681	0.116880823	0.101507051
1556	ELAC2	ENSG00000006744	17:12995978-12996546:-	0.00065562	0.06629605	0.094441177
1557	TTC19	ENSG00000011295	17:16025171-16026539:+	0.00024788	0.184059445	0.137375482
1558	MPRIP	ENSG00000133030	17:17180088-17180606:+	0.0330696	0.098061382	0.091421846
1559	FLCN	ENSG00000154803	17:17215316-17216379:-	0.04076064	0.176361735	0.181414984
1560	FLCN	ENSG00000154803	17:17219209-17221536:-	8.15E-08	0.706140561	0.608018506
1561	FLCN	ENSG00000154803	17:17224143-17226175:-	2.44E-05	0.209609515	0.283079987
1562	FLCN	ENSG00000154803	17:17228161-17231793:-	9.79E-05	0.291685079	0.397590273
1563	RAI1	ENSG00000108557	17:17803849-17809389:+	2.86E-06	0.323256795	0.253396481
1564	RAI1	ENSG00000108557	17:17809439-17809969:+	0.00050959	0.158127213	0.121735197
1565	TOM1L2	ENSG00000175662	17:17848859-17850892:-	6.85E-05	0.139639813	0.112264968
1566	ATPAF2	ENSG00000171953	17:18024704-18026318:-	2.49E-08	0.179577249	0.119469229
1567	DRG2	ENSG00000108591	17:18093973-18098269:+	0.00694951	0.134050515	0.111314809
1568	DRG2	ENSG00000108591	17:18099077-18099632:+	3.81E-07	0.238838301	0.185193618
1569	LLGL1	ENSG00000131899	17:18234175-18234272:+	0.04237533	0.11736554	0.098039857
1570	LLGL1	ENSG00000131899	17:18236939-18237480:+	0.04826501	0.309814988	0.291357714
1571	LLGL1	ENSG00000131899	17:18240873-18241450:+	6.83E-07	0.503734598	0.397713091
1572	LLGL1	ENSG00000131899	17:18241715-18241884:+	1.20E-09	0.379447183	0.256696377
1573	FLII	ENSG00000177731	17:18245419-18245554:-	0.00942131	0.108963771	0.08992804
1574	FLII	ENSG00000177731	17:18245660-18245743:-	0.00010295	0.197810294	0.24004906
1575	FLII	ENSG00000177731	17:18245850-18245933:-	0.00014871	0.229819667	0.280527312
1576	FLII	ENSG00000177731	17:18256597-18256908:-	0.00312815	0.243489884	0.194694081
1577	SHMT1	ENSG00000176974	17:18330671-18333165:-	0.03696102	0.202559183	0.183494521
1578	EPN2	ENSG00000072134	17:19329647-19331852:+	0.0081347	0.131398001	0.112065753
1579	MAPK7	ENSG00000166484	17:19381686-19381780:+	0.04898696	0.189510204	0.214623564
1580	MAP2K3	ENSG00000034152	17:21305128-21312141:+	0.01527193	0.177588895	0.146223871
1581	WSB1	ENSG00000109046	17:27309272-27310060:+	7.74E-06	0.12282451	0.085498584
1582	WSB1	ENSG00000109046	17:27310174-27311508:+	2.56E-05	0.161251791	0.119908652
1583	LGALS9	ENSG00000168961	17:27647432-27648835:+	0.02841291	0.118038868	0.085675125
1584	IFT20	ENSG00000109083	17:28330528-28331858:-	0.00235351	0.138284682	0.106534997
1585	VTN	ENSG00000109072	17:28368673-28368871:-	0.00010551	0.264910912	0.183547508
1586	UNC119	ENSG00000109103	17:28548101-28548591:-	5.62E-07	0.241120591	0.162300539
1587	PIGS	ENSG00000087111	17:28556972-28558475:-	0.00074163	0.148631521	0.116759383
1588	SGK494	ENSG00000167524	17:28610699-28611142:-	0.00182417	0.678466195	0.612107117
1589	SGK494	ENSG00000167524	17:28612084-28612261:-	0.00027306	0.379628976	0.274690104
1590	PHF12	ENSG00000109118	17:28906994-28907589:-	0.03899624	0.183012788	0.163487284
1591	PHF12	ENSG00000109118	17:28907672-28908782:-	0.00329328	0.171457901	0.140455717
1592	MYO18A	ENSG00000196535	17:29092456-29092854:-	0.02559999	0.181379787	0.151530567
1593	MYO18A	ENSG00000196535	17:29094090-29094649:-	6.35E-06	0.15139653	0.09363457
1594	MYO18A	ENSG00000196535	17:29116455-29118044:-	0.01495681	0.131616522	0.177822742
1595	GIT1	ENSG00000108262	17:29576674-29576862:-	0.00402157	0.096661482	0.080574121

S/N	Gene	Ensembl ID	Position of retained intron	p-value	AD_IR_average	Cont_IR_average
1596	ANKRD13B	ENSG00000198720	17:29608110-29608194:+	0.03426741	0.188990329	0.209702896
1597	ANKRD13B	ENSG00000198720	17:29608240-29608850:+	0.0015919	0.196896824	0.23459251
1598	ANKRD13B	ENSG00000198720	17:29609421-29610684:+	0.04133512	0.164630298	0.146060848
1599	ANKRD13B	ENSG00000198720	17:29610766-29611578:+	0.00064338	0.225161146	0.176333047
1600	ANKRD13B	ENSG00000198720	17:29611643-29611875:+	7.31E-05	0.272240805	0.208985996
1601	SUZ12P1	ENSG00000264538	17:30768867-30769000:+	0.00214152	0.924830524	0.908822506
1602	RHOT1	ENSG00000126858	17:32211238-32224615:+	0.00313594	0.03777987	0.032592327
1603	C17orf75	ENSG00000108666	17:32331978-32333416:-	0.00027278	0.115492599	0.084782029
1604	C17orf75	ENSG00000108666	17:32333520-32334468:-	0.00045264	0.107665282	0.083203
1605	PSMD11	ENSG00000108671	17:32474824-32477520:+	0.03170213	0.112466129	0.09246151
1606	PSMD11	ENSG00000108671	17:32477583-32479250:+	0.00017696	0.103318678	0.068365653
1607	PSMD11	ENSG00000108671	17:32479376-32479850:+	0.00048228	0.108751656	0.083530871
1608	PSMD11	ENSG00000108671	17:32479886-32480145:+	0.00404163	0.106240895	0.090155471
1609	DDX52	ENSG00000278053	17:37619839-37620872:-	0.01799265	0.08583479	0.06444063
1610	DDX52	ENSG00000278053	17:37620948-37621126:-	0.00331514	0.135060315	0.104711258
1611	ARHGAP23	ENSG00000275832	17:38498510-38498931:+	0.00209064	0.122946967	0.103021279
1612	ARHGAP23	ENSG00000275832	17:38498954-38500596:+	0.00089892	0.184511096	0.156777529
1613	SRCIN1	ENSG00000277363	17:38547881-38548556:-	0.00992106	0.147258812	0.132101506
1614	SRCIN1	ENSG00000277363	17:38560097-38560332:-	0.04059067	0.080067058	0.099331647
1615	SRCIN1	ENSG00000277363	17:38560425-38561462:-	0.04121077	0.088265193	0.110798753
1616	SRCIN1	ENSG00000277363	17:38562328-38562826:-	0.00147556	0.082549124	0.117080778
1617	SRCIN1	ENSG00000277363	17:38564313-38568210:-	0.00077552	0.073456951	0.100964006
1618	MLLT6	ENSG00000275023	17:38722227-38722677:+	0.02163541	0.193663755	0.163021413
1619	CISD3	ENSG00000277972	17:38730406-38730759:+	0.01249149	0.182788364	0.141770086
1620	PCGF2	ENSG00000277258	17:38735600-38736089:-	0.00596299	0.135543587	0.110744747
1621	STARD3	ENSG00000131748	17:39658026-39658404:+	0.00702063	0.457912	0.413957506
1622	STARD3	ENSG00000131748	17:39661085-39662250:+	0.01094607	0.100278756	0.079992345
1623	STARD3	ENSG00000131748	17:39662344-39662803:+	0.00659941	0.11355027	0.08775646
1624	GSDMB	ENSG00000073605	17:39905985-39906110:-	4.29E-06	0.09136752	0.064491712
1625	GSDMB	ENSG00000073605	17:39909042-39909755:-	8.35E-05	0.289183585	0.250353158
1626	GSDMB	ENSG00000073605	17:39909924-39912325:-	0.00446212	0.111042834	0.090568262
1627	PSMD3	ENSG00000108344	17:39995527-39996182:+	0.00651871	0.12215083	0.105178869
1628	MED24	ENSG00000008838	17:40027034-40027382:-	0.00545337	0.277500354	0.233452003
1629	MED24	ENSG00000008838	17:40027465-40027908:-	0.00089087	0.361039244	0.283991639
1630	MED24	ENSG00000008838	17:40027946-40028825:-	0.00085973	0.362427507	0.278469814
1631	NR1D1	ENSG00000126368	17:40094122-40094934:-	0.01121884	0.102043844	0.088115708
1632	NR1D1	ENSG00000126368	17:40096587-40096690:-	0.0120054	0.106350961	0.087025296
1633	CASC3	ENSG00000108349	17:40166861-40167497:+	0.0202741	0.09518726	0.116620488
1634	RAPGEFL1	ENSG00000108352	17:40184349-40184580:+	5.30E-05	0.189446041	0.137898557
1635	RARA	ENSG00000131759	17:40352507-40354301:+	0.01985239	0.095383199	0.074630601
1636	DNAJC7	ENSG00000168259	17:41976770-41977260:-	0.04345755	0.213016027	0.227442351
1637	KAT2A	ENSG00000108773	17:42114282-42114357:-	0.00689388	0.11685963	0.094655829
1638	KAT2A	ENSG00000108773	17:42114604-42114891:-	0.00557529	0.119912485	0.097826964
1639	KAT2A	ENSG00000108773	17:42115035-42115722:-	0.00017793	0.133351996	0.100080888
1640	GHDC	ENSG00000167925	17:42189921-42190184:-	2.21E-06	0.4634205	0.342463468
1641	GHDC	ENSG00000167925	17:42190270-42190623:-	6.91E-08	0.468089659	0.34421787
1642	GHDC	ENSG00000167925	17:42190757-42190831:-	0.00017231	0.46632961	0.380169338
1643	COASY	ENSG00000068120	17:42564175-42564445:+	0.03024818	0.126195404	0.159463395
1644	COASY	ENSG00000068120	17:42564577-42564708:+	0.00218037	0.084878471	0.11028203
1645	COASY	ENSG00000068120	17:42564898-42564982:+	0.00093353	0.093934311	0.115301425
1646	PSMC3IP	ENSG00000131470	17:42573024-42573106:-	0.01422656	0.35821872	0.403558169
1647	PSMC3IP	ENSG00000131470	17:42573364-42573477:-	0.00501813	0.195716351	0.143251144
1648	TUBG2	ENSG00000037042	17:42659552-42659833:+	2.19E-05	0.331524123	0.253196673
1649	TUBG2	ENSG00000037042	17:42660316-42660638:+	0.00456971	0.094896782	0.079289777
1650	TUBG2	ENSG00000037042	17:42660707-42662972:+	1.39E-05	0.463762159	0.383973468
1651	TUBG2	ENSG00000037042	17:42663052-42663376:+	1.65E-05	0.329913451	0.262238182
1652	TUBG2	ENSG00000037042	17:42665562-42665677:+	0.0002067	0.48751978	0.403465065

S/N	Gene	Ensembl ID	Position of retained intron	p-value	AD_IR_average	Cont_IR_average
1653	PLEKHH3	ENSG00000068137	17:42670705-42670993:-	0.00203407	0.143821905	0.096030979
1654	CNTNAP1	ENSG00000108797	17:42697799-42697902:+	0.01142476	0.124364516	0.104486539
1655	CNTNAP1	ENSG00000108797	17:42697950-42698617:+	0.03027809	0.110455823	0.097765718
1656	EZH1	ENSG00000108799	17:42702961-42703739:-	2.31E-05	0.697420598	0.614659377
1657	EZH1	ENSG00000108799	17:42718067-42718453:-	0.00339328	0.082331588	0.138330349
1658	AARSD1	ENSG00000266967	17:42955224-42955841:-	0.00063808	0.156065783	0.119636053
1659	AARSD1	ENSG00000266967	17:42955972-42956203:-	0.00526907	0.160075539	0.132752906
1660	MPP3	ENSG00000161647	17:43801877-43808955:-	0.00011099	0.100364944	0.074837014
1661	MPP3	ENSG00000161647	17:43810915-43811111:-	1.35E-07	0.117420122	0.067691886
1662	MPP3	ENSG00000161647	17:43811205-43814010:-	0.02809999	0.310209251	0.268905429
1663	HDAC5	ENSG00000108840	17:44079277-44080106:-	3.39E-05	0.118580704	0.091390264
1664	HDAC5	ENSG00000108840	17:44091831-44092171:-	0.00014949	0.145837741	0.108678603
1665	HDAC5	ENSG00000108840	17:44092284-44092380:-	0.03740943	0.145370539	0.119698164
1666	RUNDC3A	ENSG00000108309	17:44315009-44315154:+	0.04707258	0.14124373	0.122069319
1667	RUNDC3A	ENSG00000108309	17:44316522-44316618:+	2.78E-05	0.159527645	0.117787608
1668	SLC25A39	ENSG00000013306	17:44320276-44320354:-	0.02570626	0.096350615	0.078122721
1669	SLC25A39	ENSG00000013306	17:44320731-44321057:-	0.00607409	0.362238073	0.313121299
1670	SLC25A39	ENSG00000013306	17:44322528-44322807:-	0.00037227	0.14600813	0.112711288
1671	SLC25A39	ENSG00000013306	17:44322852-44323283:-	1.89E-06	0.097247948	0.067658164
1672	ADAM11	ENSG00000073670	17:44775311-44775393:+	0.03824917	0.106797126	0.085580595
1673	ADAM11	ENSG00000073670	17:44777863-44777951:+	0.00660291	0.271721768	0.225272929
1674	NMT1	ENSG00000136448	17:45103876-45104858:+	0.03919125	0.20032398	0.187135432
1675	FMNL1	ENSG00000184922	17:45241230-45241381:+	0.02538519	0.628644366	0.585858182
1676	FMNL1	ENSG00000184922	17:45244025-45244175:+	1.33E-07	0.357709154	0.233092221
1677	FMNL1	ENSG00000184922	17:45245973-45246209:+	0.00092461	0.197906073	0.153259008
1678	KANSL1	ENSG00000120071	17:46033192-46033402:-	0.00831246	0.190621582	0.153650631
1679	NPEPPS	ENSG00000141279	17:47619164-47619736:+	0.00742206	0.100138533	0.132597262
1680	TBKBP1	ENSG00000198933	17:47709452-47710497:+	3.82E-05	0.151617274	0.095027052
1681	SCRN2	ENSG00000141295	17:47838450-47838530:-	0.02131723	0.209387862	0.173269727
1682	SCRN2	ENSG00000141295	17:47839006-47839443:-	0.02535162	0.136281513	0.113727675
1683	SCRN2	ENSG00000141295	17:47840372-47840669:-	0.00068514	0.130266518	0.098177483
1684	SNF8	ENSG00000159210	17:48930612-48931642:-	1.81E-11	0.366864378	0.261569086
1685	ITGA3	ENSG00000005884	17:50075526-50075598:+	1.39E-05	0.360227854	0.248047099
1686	ITGA3	ENSG00000005884	17:50077447-50078045:+	0.04235948	0.086334715	0.064396647
1687	ITGA3	ENSG00000005884	17:50087869-50088224:+	0.01980884	0.064266853	0.065738055
1688	ITGA3	ENSG00000005884	17:50088366-50089109:+	0.00113948	0.090368402	0.088053716
1689	PDK2	ENSG00000005882	17:50109400-50109956:+	0.01958461	0.123507967	0.089227008
1690	SAMD14	ENSG00000167100	17:50113048-50113923:-	0.01608933	0.204654976	0.171271204
1691	SAMD14	ENSG00000167100	17:50114079-50114186:-	0.03224752	0.106372717	0.08541207
1692	SAMD14	ENSG00000167100	17:50114306-50115563:-	0.00515561	0.121093609	0.093498748
1693	SAMD14	ENSG00000167100	17:50116090-50117406:-	4.05E-05	0.25028054	0.180349395
1694	SAMD14	ENSG00000167100	17:50117695-50118160:-	0.00015445	0.204217716	0.143808078
1695	RSAD1	ENSG00000136444	17:50482456-50482642:+	0.00046792	0.301718256	0.385654143
1696	RSAD1	ENSG00000136444	17:50482706-50483339:+	2.08E-08	0.196896037	0.299532106
1697	RSAD1	ENSG00000136444	17:50483487-50483705:+	0.00044453	0.326170659	0.395840636
1698	SPATA20	ENSG00000006282	17:50551190-50551510:+	1.67E-07	0.472729634	0.337059312
1699	SPATA20	ENSG00000006282	17:50551679-50551968:+	1.96E-07	0.530069732	0.394156026
1700	UTP18	ENSG00000011260	17:51297003-51297781:+	0.00055702	0.114895466	0.084032656
1701	TOM1L1	ENSG00000141198	17:54949623-54950044:+	2.37E-11	0.463337354	0.294629039
1702	TOM1L1	ENSG00000141198	17:54950126-54960565:+	0.00212489	0.702415268	0.640816506
1703	SCPEP1	ENSG00000121064	17:56995635-56996961:+	0.00729496	0.14993559	0.2001966
1704	MSI2	ENSG00000153944	17:57675126-57676986:+	0.024972	0.089062694	0.064506242
1705	RP11-159D	ENSG00000266086	17:57989269-57989648:-	1.96E-05	0.45887439	0.361421156
1706	SRSF1	ENSG00000136450	17:58005600-58005800:-	0.033917	0.364034902	0.408129273
1707	TSPOAP1	ENSG00000005379	17:58305171-58305386:-	0.00022397	0.265063602	0.203002713
1708	TSPOAP1	ENSG00000005379	17:58305865-58306341:-	0.00530314	0.119967045	0.107918961
1709	TSPOAP1	ENSG00000005379	17:58309380-58309966:-	0.01398654	0.148065435	0.176639839

S/N	Gene	Ensembl ID	Position of retained intron	p-value	AD_IR_average	Cont_IR_average
1710	TSPOAP1	ENSG00000005379	17:58326790-58327587:-	0.04462781	0.083145072	0.108192045
1711	CLTC	ENSG00000141367	17:59687021-59690635:+	0.01230671	0.024304187	0.024607811
1712	CA4	ENSG00000167434	17:60150092-60155313:+	0.00335076	0.206370502	0.170784147
1713	CA4	ENSG00000167434	17:60155367-60156559:+	0.00520616	0.168515472	0.136442643
1714	CA4	ENSG00000167434	17:60156715-60157426:+	4.36E-07	0.530724941	0.379877439
1715	MAP3K3	ENSG00000198909	17:63689735-63690263:+	0.00049124	0.332992515	0.263102078
1716	FTSJ3	ENSG00000108592	17:63822168-63823816:-	0.00077428	0.099621422	0.135097266
1717	SMARCD2	ENSG00000108604	17:63832991-63833068:-	4.28E-05	0.367646806	0.277618208
1718	SMARCD2	ENSG00000108604	17:63833170-63833297:-	0.01225493	0.101697285	0.081077262
1719	SMARCD2	ENSG00000108604	17:63835567-63836921:-	0.03281186	0.205530232	0.174439252
1720	POLG2	ENSG00000256525	17:64485868-64490795:-	0.03741635	0.126176354	0.138214613
1721	DDX5	ENSG00000108654	17:64500326-64500548:-	0.00040994	0.186074076	0.237755336
1722	CEP95	ENSG00000258890	17:64525882-64526070:+	0.03073549	0.134233332	0.10475669
1723	CEP95	ENSG00000258890	17:64527264-64529287:+	2.82E-06	0.24206986	0.162656305
1724	CEP95	ENSG00000258890	17:64529427-64530925:+	4.54E-07	0.32778031	0.214711755
1725	CEP95	ENSG00000258890	17:64531018-64531889:+	2.70E-05	0.522891378	0.417012532
1726	CEP95	ENSG00000258890	17:64537112-64537602:+	0.02501664	0.243289249	0.207750571
1727	PLEKHM1P	ENSG00000214176	17:64822393-64824624:-	0.01873489	0.074497662	0.06136488
1728	WIPI1	ENSG00000070540	17:68426175-68427134:-	0.00744748	0.107503852	0.139009644
1729	WIPI1	ENSG00000070540	17:68428936-68429995:-	0.00017919	0.199095574	0.277398617
1730	WIPI1	ENSG00000070540	17:68435712-68436381:-	0.00911898	0.084268049	0.12092113
1731	ABCA5	ENSG00000154265	17:69248317-69249904:-	5.43E-08	0.16518235	0.104885191
1732	ABCA5	ENSG00000154265	17:69250621-69251746:-	4.88E-07	0.19662435	0.130157182
1733	ABCA5	ENSG00000154265	17:69251866-69253572:-	1.56E-08	0.104349185	0.056961575
1734	COG1	ENSG00000166685	17:73197081-73197225:+	0.00349993	0.130214732	0.099548061
1735	COG1	ENSG00000166685	17:73197396-73199864:+	0.0011549	0.139933049	0.111658447
1736	COG1	ENSG00000166685	17:73203793-73205552:+	5.81E-05	0.1977195	0.146949123
1737	COG1	ENSG00000166685	17:73205680-73206153:+	5.98E-07	0.18678703	0.127054325
1738	RAB37	ENSG00000172794	17:74744929-74745007:+	0.0105887	0.228769921	0.185542866
1739	RAB37	ENSG00000172794	17:74745084-74745305:+	0.01807689	0.413445793	0.363144426
1740	NAT9	ENSG00000109065	17:74771858-74771959:-	0.01568289	0.101697587	0.081077605
1741	NAT9	ENSG00000109065	17:74773039-74773575:-	0.00588653	0.228488232	0.185970848
1742	GRIN2C	ENSG00000161509	17:74846920-74847307:-	5.63E-07	0.378346024	0.26537214
1743	GRIN2C	ENSG00000161509	17:74847537-74847851:-	4.07E-09	0.454494549	0.283693299
1744	GRIN2C	ENSG00000161509	17:74847977-74849779:-	3.69E-08	0.388682244	0.242679542
1745	GRIN2C	ENSG00000161509	17:74850371-74850555:-	0.00015413	0.209421222	0.126818301
1746	FDXR	ENSG00000161513	17:74862947-74863075:-	0.00032725	0.13890499	0.094300291
1747	FDXR	ENSG00000161513	17:74864347-74864479:-	0.00210572	0.23359322	0.207177896
1748	HID1	ENSG00000167861	17:74951633-74951904:-	0.00702142	0.07828895	0.091900553
1749	HID1	ENSG00000167861	17:74953086-74953544:-	0.00096451	0.127247696	0.111893104
1750	HID1	ENSG00000167861	17:74953651-74954137:-	0.00239462	0.123574489	0.109886979
1751	HID1	ENSG00000167861	17:74958478-74958672:-	0.00021084	0.582367707	0.503958857
1752	HID1	ENSG00000167861	17:74958763-74958910:-	0.00044597	0.174337904	0.124082621
1753	MRPL58	ENSG00000167862	17:75012872-75017077:+	1.22E-05	0.309046123	0.246869065
1754	ARMC7	ENSG00000125449	17:75110379-75110462:+	0.00035082	0.099864401	0.066060631
1755	NUP85	ENSG00000125450	17:75208620-75209822:+	0.00631297	0.069519998	0.10520031
1756	NUP85	ENSG00000125450	17:75225464-75225697:+	0.04522796	0.20893545	0.181989677
1757	GGA3	ENSG00000125447	17:75238389-75238651:-	0.04385467	0.15057471	0.182187299
1758	MRPS7	ENSG00000125445	17:75261983-75262496:+	3.67E-05	0.208178761	0.155671104
1759	TMEM94	ENSG00000177728	17:75493102-75493490:+	1.79E-07	0.595803439	0.47949661
1760	CASKIN2	ENSG00000177303	17:75503527-75503658:-	0.0031879	0.172095915	0.125179386
1761	CASKIN2	ENSG00000177303	17:75506894-75506983:-	0.04324893	0.170460068	0.140089948
1762	TSEN54	ENSG00000182173	17:75516910-75517008:+	0.00969879	0.234268752	0.190254663
1763	TSEN54	ENSG00000182173	17:75521510-75521704:+	1.55E-05	0.316035366	0.397697364
1764	LLGL2	ENSG00000073350	17:75574668-75574870:+	0.00640994	0.23712716	0.176652225
1765	MYO15B	ENSG00000266714	17:75619799-75619878:+	0.01628908	0.14199426	0.106611112
1766	MYO15B	ENSG00000266714	17:75621570-75622003:+	0.0007071	0.221458605	0.163936478

S/N	Gene	Ensembl ID	Position of retained intron	p-value	AD_IR_average	Cont_IR_average
1767	MYO15B	ENSG00000266714	17:75622080-75623780:+	0.00936227	0.157068323	0.117211542
1768	MYO15B	ENSG00000266714	17:75624447-75624542:+	0.00169403	0.187691216	0.134120429
1769	MYO15B	ENSG00000266714	17:75625977-75626087:+	0.02339499	0.164077299	0.130554778
1770	RECQL5	ENSG00000108469	17:75627522-75627622:-	0.00026232	0.249055017	0.177560948
1771	SAP30BP	ENSG00000161526	17:75703859-75704755:+	0.00025089	0.093482888	0.071247308
1772	WBP2	ENSG00000132471	17:75847609-75848569:-	2.43E-05	0.060944396	0.055402935
1773	TRIM65	ENSG00000141569	17:75891347-75892010:-	0.00011708	0.312897573	0.353309143
1774	TRIM65	ENSG00000141569	17:75892500-75892754:-	0.01442575	0.369494268	0.330225338
1775	MRPL38	ENSG00000204316	17:75901027-75901200:-	0.01047926	0.099846146	0.083030816
1776	MRPL38	ENSG00000204316	17:75901273-75901711:-	0.01138081	0.353446439	0.318460182
1777	EXOC7	ENSG00000182473	17:76087720-76088059:-	1.48E-06	0.370108549	0.281331779
1778	EXOC7	ENSG00000182473	17:76088122-76088463:-	0.00048035	0.392019805	0.33173626
1779	QRICH2	ENSG00000129646	17:76276767-76277162:-	2.85E-05	0.105359182	0.069040861
1780	QRICH2	ENSG00000129646	17:76277310-76277988:-	0.00198392	0.175519438	0.142222836
1781	QRICH2	ENSG00000129646	17:76280451-76280653:-	0.00739037	0.197279301	0.154364325
1782	UBE2O	ENSG00000175931	17:76395861-76396127:-	1.17E-06	0.11431602	0.195041868
1783	UBE2O	ENSG00000175931	17:76399921-76400146:-	0.00117669	0.100744839	0.147816931
1784	MXRA7	ENSG00000182534	17:76683927-76685071:-	0.00433702	0.026403108	0.02178713
1785	Sep-09	ENSG00000184640	17:77493076-77497314:+	5.78E-05	0.144009604	0.100547044
1786	Sep-09	ENSG00000184640	17:77497366-77498522:+	1.06E-06	0.129512583	0.079681203
1787	TNRC6C	ENSG00000078687	17:78093124-78093619:+	0.02689826	0.086840967	0.11138579
1788	AFMID	ENSG00000183077	17:78191060-78202702:+	6.50E-05	0.075413255	0.103834599
1789	AFMID	ENSG00000183077	17:78191060-78206910:+	2.95E-07	0.070606267	0.094015352
1790	BAIAP2	ENSG00000175866	17:81106907-81108474:+	0.00015421	0.12375623	0.085412904
1791	CEP131	ENSG00000141577	17:81197055-81197711:-	0.00811633	0.114267126	0.081635403
1792	TEPSIN	ENSG00000167302	17:81236801-81236979:-	0.04940781	0.125054406	0.095980006
1793	BAHCC1	ENSG00000266074	17:81455390-81456296:+	0.04694178	0.136488423	0.150788766
1794	CCDC137	ENSG00000185298	17:81671826-81672075:+	0.01163137	0.109431026	0.089943969
1795	HGS	ENSG00000185359	17:81684103-81685604:+	0.00071077	0.266205101	0.217527305
1796	HGS	ENSG00000185359	17:81685689-81686311:+	0.00202716	0.254649174	0.216079869
1797	HGS	ENSG00000185359	17:81693965-81694814:+	0.02257706	0.115991654	0.097747666
1798	HGS	ENSG00000185359	17:81694853-81694923:+	0.0228454	0.146278139	0.126277438
1799	HGS	ENSG00000185359	17:81695999-81696356:+	0.0007051	0.15651468	0.121296435
1800	HGS	ENSG00000185359	17:81696998-81700466:+	0.00021161	0.161117406	0.128123192
1801	HGS	ENSG00000185359	17:81700814-81701044:+	1.78E-06	0.156913601	0.117561383
1802	ARHGDIA	ENSG00000141522	17:81869406-81869541:-	0.00573049	0.130316871	0.101974755
1803	PCYT2	ENSG00000185813	17:81906177-81906463:-	0.00035319	0.294623793	0.243594429
1804	SIRT7	ENSG00000187531	17:81912614-81913773:-	0.00226357	0.246054993	0.273990701
1805	SIRT7	ENSG00000187531	17:81914167-81914293:-	0.00219131	0.093321582	0.131471958
1806	SIRT7	ENSG00000187531	17:81914702-81915439:-	0.04879068	0.250843555	0.290436727
1807	SIRT7	ENSG00000187531	17:81915681-81917614:-	0.00427148	0.421018228	0.367675532
1808	ASPSCR1	ENSG00000169696	17:81977748-81983553:+	0.00432846	0.01630847	0.019843118
1809	ASPSCR1	ENSG00000169696	17:81996846-82009036:+	6.42E-05	0.327817171	0.247587273
1810	CENPX	ENSG00000169689	17:82019219-82019292:-	0.00170097	0.119793338	0.150226684
1811	LRRC45	ENSG00000169683	17:82028683-82029092:+	0.00199916	0.5419845	0.511387013
1812	DCXR	ENSG00000169738	17:82036643-82036720:-	0.00116493	0.134007917	0.104485842
1813	RFNG	ENSG00000169733	17:82049842-82049917:-	0.00232905	0.745349061	0.692949143
1814	RFNG	ENSG00000169733	17:82050006-82050401:-	0.0107592	0.806001232	0.772260026
1815	DUS1L	ENSG00000169718	17:82058408-82058780:-	0.01580533	0.509601671	0.565806195
1816	DUS1L	ENSG00000169718	17:82060093-82060700:-	1.19E-08	0.204083362	0.133784927
1817	FASN	ENSG00000169710	17:82091684-82092454:-	0.0042098	0.07567732	0.124187388
1818	CSNK1D	ENSG00000141551	17:82253244-82255428:-	1.39E-07	0.241150022	0.179308975
1819	CSNK1D	ENSG00000141551	17:82265796-82273305:-	0.00517377	0.105977324	0.084400844
1820	HEXDC	ENSG00000169660	17:82435872-82436666:+	4.37E-05	0.184729776	0.129515468
1821	NARF	ENSG00000141562	17:82478918-82481081:+	0.00140618	0.193146623	0.159276474
1822	FN3KRP	ENSG00000141560	17:82716896-82718905:+	0.00056178	0.264610463	0.219526777
1823	FN3KRP	ENSG00000141560	17:82719057-82720271:+	0.01477859	0.132040322	0.118101825

S/N	Gene	Ensembl ID	Position of retained intron	p-value	AD_IR_average	Cont_IR_average
1824	TBCD	ENSG00000141556	17:82920618-82921500:+	0.00044831	0.119960137	0.151492996
1825	TBCD	ENSG00000141556	17:82929500-82930521:+	6.68E-11	0.594779037	0.482330675
1826	TBCD	ENSG00000141556	17:82932735-82937270:+	9.08E-05	0.111152168	0.08671147
1827	USP14	ENSG00000101557	18:210031-210385:+	0.00140748	0.153019282	0.214270016
1828	THOC1	ENSG00000079134	18:215504-216485:-	0.00224621	0.11056605	0.09056704
1829	THOC1	ENSG00000079134	18:225139-225336:-	0.00044212	0.590564963	0.648072649
1830	ELP2	ENSG00000134759	18:36167222-36170062:+	0.00493304	0.207430826	0.175434645
1831	ELP2	ENSG00000134759	18:36170196-36171046:+	0.00136756	0.091628912	0.070647573
1832	CTIF	ENSG00000134030	18:48758405-48761389:+	0.00090099	0.132280451	0.110708409
1833	MBD1	ENSG00000141644	18:50271540-50272676:-	1.41E-05	0.278909268	0.245391487
1834	MBD1	ENSG00000141644	18:50275728-50275834:-	0.03495876	0.078582046	0.105832677
1835	CXXC1	ENSG00000154832	18:50283564-50283704:-	5.76E-09	0.66467461	0.549521481
1836	CXXC1	ENSG00000154832	18:50285247-50285324:-	1.62E-06	0.206483813	0.272032351
1837	CXXC1	ENSG00000154832	18:50285351-50285748:-	7.48E-07	0.224463141	0.298323013
1838	CXXC1	ENSG00000154832	18:50285928-50286021:-	1.54E-16	0.07507582	0.147021648
1839	CXXC1	ENSG00000154832	18:50286858-50287586:-	5.99E-05	0.177017939	0.227496255
1840	RTTN	ENSG00000176225	18:70005267-70006380:-	0.00081772	0.124053596	0.086756684
1841	MBP	ENSG00000197971	18:76985210-76988494:-	0.001699	0.020340328	0.013394553
1842	CTDP1	ENSG00000060069	18:79729069-79736354:+	0.00010132	0.230284845	0.152426118
1843	HSBP1L1	ENSG00000226742	18:79964786-79966611:+	0.0001773	0.300884752	0.374362803
1844	HSBP1L1	ENSG00000226742	18:79968183-79970439:+	0.00015896	0.183788646	0.149827525
1845	TXNL4A	ENSG00000141759	18:79973856-79988239:-	0.00537143	0.018760831	0.013959303
1846	MIER2	ENSG00000105556	19:326598-327132:-	0.00325693	0.13346635	0.106173019
1847	RNF126	ENSG00000070423	19:650296-651610:-	3.39E-05	0.315368427	0.258141784
1848	PTBP1	ENSG00000011304	19:803636-804035:+	0.00435547	0.086997694	0.114467008
1849	WDR18	ENSG00000065268	19:990364-990851:+	7.59E-07	0.486988976	0.359211442
1850	TMEM259	ENSG00000182087	19:1011663-1011740:-	0.00109202	0.210582417	0.168431808
1851	TMEM259	ENSG00000182087	19:1012188-1012462:-	0.02783646	0.400244951	0.437445844
1852	ABCA7	ENSG00000064687	19:1063863-1064160:+	0.01862035	0.246312804	0.212785127
1853	SBNO2	ENSG00000064932	19:1109777-1110744:-	0.02041225	0.132979504	0.155411986
1854	STK11	ENSG00000118046	19:1220505-1220580:+	0.04708104	0.222369463	0.191797935
1855	STK11	ENSG00000118046	19:1226663-1227592:+	4.45E-07	0.225741243	0.135504435
1856	ATP5D	ENSG00000099624	19:1241991-1242455:+	1.16E-06	0.133163591	0.08175941
1857	GAMT	ENSG00000130005	19:1399587-1399792:-	3.46E-05	0.10356606	0.068293381
1858	DAZAP1	ENSG00000071626	19:1425960-1428841:+	0.02922772	0.357604732	0.323929558
1859	APC2	ENSG00000115266	19:1458060-1460180:+	0.00347085	0.113373902	0.155690494
1860	APC2	ENSG00000115266	19:1460857-1461036:+	0.02842326	0.083036243	0.106842894
1861	PLK5	ENSG00000185988	19:1525418-1525594:+	0.02384746	0.147429834	0.116005678
1862	PLK5	ENSG00000185988	19:1528134-1528301:+	0.00243874	0.12688192	0.074359201
1863	PLK5	ENSG00000185988	19:1528428-1528897:+	0.00366204	0.1528864	0.10416271
1864	PLK5	ENSG00000185988	19:1528974-1529405:+	0.001224	0.386528012	0.291265576
1865	PLK5	ENSG00000185988	19:1529824-1531737:+	0.01036606	0.248361282	0.197008026
1866	MBD3	ENSG00000071655	19:1578158-1578334:-	0.02722699	0.093741543	0.075143419
1867	MBD3	ENSG00000071655	19:1585214-1592521:-	0.00235663	0.32701122	0.266733931
1868	REXO1	ENSG00000079313	19:1823785-1825838:-	0.02529832	0.186319506	0.213168922
1869	SCAMP4	ENSG00000227500	19:1905439-1914978:+	0.00073101	0.080688752	0.068592512
1870	SCAMP4	ENSG00000227500	19:1915026-1917693:+	0.01518448	0.238427248	0.220840582
1871	SCAMP4	ENSG00000227500	19:1918990-1923069:+	0.00112525	0.213708624	0.184019935
1872	CSNK1G2	ENSG00000133275	19:1979093-1979162:+	0.02153433	0.365229732	0.307666758
1873	BTBD2	ENSG00000133243	19:1986649-1986829:-	6.34E-05	0.114995117	0.085272538
1874	BTBD2	ENSG00000133243	19:1986976-1987165:-	0.00383093	0.122580417	0.097823327
1875	BTBD2	ENSG00000133243	19:1987692-1990003:-	0.03627529	0.194796349	0.173543919
1876	IZUMO4	ENSG00000099840	19:2097495-2097928:+	3.41E-15	0.120013416	0.048857661
1877	IZUMO4	ENSG00000099840	19:2097955-2098051:+	0.01227755	0.235260683	0.19193607
1878	IZUMO4	ENSG00000099840	19:2098127-2098286:+	8.84E-09	0.492443402	0.350621494
1879	IZUMO4	ENSG00000099840	19:2098334-2098435:+	2.29E-06	0.153092468	0.091539657
1880	AP3D1	ENSG00000065000	19:2110896-2111284:-	0.00807813	0.153110391	0.132475401

S/N	Gene	Ensembl ID	Position of retained intron	p-value	AD_IR_average	Cont_IR_average
1882	AP3D1	ENSG00000065000	19:2111828-2112859:-	0.01633234	0.211685299	0.178487544
1883	AP3D1	ENSG00000065000	19:2113413-2114124:-	0.0110951	0.113815451	0.093520924
1884	DOT1L	ENSG00000104885	19:2227127-2229784:+	0.00093479	0.55593322	0.505568662
1885	PLEKHJ1	ENSG00000104886	19:2235828-2235922:-	8.81E-08	0.173711848	0.107947977
1886	LSM7	ENSG00000130332	19:2324192-2328386:-	0.0001022	0.094478851	0.07348154
1887	LSM7	ENSG00000130332	19:2328477-2328560:-	0.00057007	0.258637032	0.202288527
1888	SPPL2B	ENSG00000005206	19:2334721-2337442:+	0.00059956	0.114607875	0.074673684
1889	SPPL2B	ENSG00000005206	19:2344424-2344552:+	3.09E-05	0.181392144	0.129264849
1890	LMNB2	ENSG00000176619	19:2430952-2431547:-	0.02477434	0.132915705	0.109097177
1891	SGTA	ENSG00000104969	19:2757782-2759256:-	0.00012573	0.194388487	0.151857948
1892	SGTA	ENSG00000104969	19:2759294-2761459:-	3.38E-06	0.176960196	0.12394924
1893	MFSD12	ENSG00000161091	19:3544939-3546073:-	0.00342561	0.670216427	0.706025475
1894	APBA3	ENSG00000011132	19:3751329-3751433:-	0.00333789	0.239165485	0.181291661
1895	APBA3	ENSG00000011132	19:3751553-3752507:-	0.03184392	0.560212415	0.510602481
1896	PIAS4	ENSG00000105229	19:4033580-4037373:+	4.03E-08	0.452005585	0.338352623
1897	SIRT6	ENSG00000077463	19:4175760-4175841:-	0.04404474	0.280687524	0.242935086
1898	SIRT6	ENSG00000077463	19:4179286-4180781:-	0.00553913	0.10269526	0.086228635
1899	FSD1	ENSG00000105255	19:4310596-4311841:+	9.89E-06	0.60576022	0.524171922
1900	MPND	ENSG00000008382	19:4355021-4355096:+	5.11E-06	0.22121761	0.134533335
1901	MPND	ENSG00000008382	19:4359255-4359915:+	7.77E-07	0.209410657	0.123330095
1902	UBXN6	ENSG00000167671	19:4446197-4446282:-	0.00283343	0.174097011	0.137778396
1903	SEMA6B	ENSG00000167680	19:4552639-4554387:-	7.51E-05	0.190642521	0.137657327
1904	SEMA6B	ENSG00000167680	19:4555095-4555473:-	0.00032248	0.090802196	0.063173612
1905	SEMA6B	ENSG00000167680	19:4555564-4555987:-	0.0004875	0.100984549	0.068720938
1906	SAFB2	ENSG00000130254	19:5592887-5593890:-	4.75E-07	0.093205939	0.058669996
1907	SAFB	ENSG00000160633	19:5648043-5648988:+	0.00054847	0.174463187	0.250509361
1908	SAFB	ENSG00000160633	19:5667450-5667819:+	0.00424885	0.10752256	0.080825549
1909	HSD11B1L	ENSG00000167733	19:5686991-5687281:+	0.00019048	0.17921503	0.135781457
1910	HSD11B1L	ENSG00000167733	19:5687375-5687502:+	6.03E-06	0.248365179	0.180279921
1911	LONP1	ENSG00000196365	19:5708403-5711770:-	0.01859429	0.200490185	0.174144112
1912	DUS3L	ENSG00000141994	19:5785791-5786466:-	0.01266826	0.1442908	0.11055839
1913	DUS3L	ENSG00000141994	19:5786542-5786748:-	0.03465721	0.108030044	0.083416077
1914	CAPS	ENSG00000105519	19:5914489-5914562:+	0.00025657	0.29493707	0.189258157
1915	CAPS	ENSG00000105519	19:5914740-5914939:+	8.00E-05	0.214731417	0.127694981
1916	CAPS	ENSG00000105519	19:5915146-5915220:+	0.0001847	0.209889815	0.130145564
1917	SLC25A23	ENSG00000125648	19:6452479-6453980:-	0.00283922	0.113126823	0.095495821
1918	GPR108	ENSG00000125734	19:6732549-6732986:-	0.0457538	0.298594546	0.265758757
1919	GPR108	ENSG00000125734	19:6733062-6733167:-	0.00243493	0.109775968	0.138599752
1920	GPR108	ENSG00000125734	19:6733913-6734004:-	0.00036066	0.315833976	0.264825849
1921	GPR108	ENSG00000125734	19:6734307-6735621:-	4.26E-05	0.143813591	0.10971559
1922	GPR108	ENSG00000125734	19:6735958-6736591:-	0.00395843	0.092731306	0.075602603
1923	MCOLN1	ENSG00000090674	19:7527960-7528157:+	0.00230948	0.165905813	0.132377442
1924	PNPLA6	ENSG00000032444	19:7541051-7541353:+	0.00228475	0.071514224	0.101843503
1925	PNPLA6	ENSG00000032444	19:7550640-7550993:+	0.00365956	0.113424599	0.143066904
1926	XAB2	ENSG00000076924	19:7619647-7619746:-	0.00062251	0.130808482	0.082542115
1927	XAB2	ENSG00000076924	19:7620446-7620546:-	0.0005583	0.066263112	0.089890321
1928	XAB2	ENSG00000076924	19:7628298-7629476:-	0.04715708	0.143785482	0.129783197
1929	EVI5L	ENSG00000142459	19:7847921-7848920:+	0.0010049	0.181336567	0.131030874
1930	MAP2K7	ENSG00000076984	19:7909896-7910062:+	0.00386777	0.207879391	0.171146018
1931	MAP2K7	ENSG00000076984	19:7910373-7910452:+	0.0384851	0.183976549	0.160250821
1932	TIMM44	ENSG00000104980	19:7928166-7931137:-	0.02239529	0.22165659	0.198139834
1933	TIMM44	ENSG00000104980	19:7932932-7933484:-	7.41E-07	0.134280798	0.084164286
1934	TIMM44	ENSG00000104980	19:7933570-7933863:-	0.02500174	0.371517427	0.332923714
1935	CERS4	ENSG00000090661	19:8255879-8256235:+	3.52E-08	0.284390866	0.210982313
1936	CERS4	ENSG00000090661	19:8257985-8261687:+	0.00145441	0.10695938	0.088402432
1937	ZNF414	ENSG00000133250	19:8513341-8514043:-	0.02293882	0.110795861	0.091512006
1938	EIF3G	ENSG00000130811	19:10116074-10116799:-	0.00548979	0.119378577	0.095982204

S/N	Gene	Ensembl ID	Position of retained intron	p-value	AD_IR_average	Cont_IR_average
1939	EIF3G	ENSG00000130811	19:10117188-10118667:-	0.01208265	0.094390789	0.083184952
1940	DNMT1	ENSG00000130816	19:10133701-10134216:-	0.00729419	0.134367365	0.107975271
1941	DNMT1	ENSG00000130816	19:10139817-10140045:-	7.26E-05	0.069707698	0.106260313
1942	DNMT1	ENSG00000130816	19:10141189-10142027:-	0.03282387	0.153797911	0.19210319
1943	MRPL4	ENSG00000105364	19:10258522-10258608:+	0.00501381	0.303073683	0.259881448
1944	ZGLP1	ENSG00000220201	19:10308550-10309348:-	0.02573761	0.336809496	0.285405923
1945	RAVER1	ENSG00000161847	19:10321259-10321530:-	0.04898063	0.118064777	0.101707912
1946	TYK2	ENSG00000105397	19:10364771-10364850:-	0.03323689	0.111019429	0.089350974
1947	CDC37	ENSG00000105401	19:10395343-10395434:-	1.29E-07	0.201296718	0.291356149
1948	ATG4D	ENSG00000130734	19:10545130-10546838:+	0.03665285	0.081746229	0.095296869
1949	ATG4D	ENSG00000130734	19:10547115-10547188:+	0.01382654	0.339648256	0.290635482
1950	KRI1	ENSG00000129347	19:10557682-10557768:-	0.00221352	0.279148098	0.33525139
1951	KRI1	ENSG00000129347	19:10565034-10565716:-	0.03730742	0.21822974	0.196074499
1952	KRI1	ENSG00000129347	19:10565790-10565905:-	0.00034855	0.088667183	0.057704259
1953	QTRT1	ENSG00000213339	19:10701703-10701949:+	0.00075518	0.202921688	0.161493375
1954	QTRT1	ENSG00000213339	19:10712628-10712757:+	0.00018062	0.143446697	0.092208945
1955	DNM2	ENSG00000079805	19:10795439-10797379:+	9.12E-06	0.095434145	0.130329314
1956	DNM2	ENSG00000079805	19:10805967-10812263:+	0.00019282	0.118406579	0.098859922
1957	DNM2	ENSG00000079805	19:10829268-10830126:+	0.00120109	0.180510428	0.152782458
1958	DNM2	ENSG00000079805	19:10830378-10830977:+	1.21E-06	0.149745485	0.103866474
1959	CARM1	ENSG00000142453	19:10916777-10919594:+	0.01037075	0.304053988	0.268924934
1960	CARM1	ENSG00000142453	19:10919680-10919876:+	0.0069291	0.340749866	0.295478843
1961	TMEM205	ENSG00000105518	19:11345415-11345519:-	0.01536216	0.198644094	0.228419888
1962	CCDC159	ENSG00000183401	19:11352133-11353450:+	0.00723455	0.136423874	0.109014881
1963	PRKCSH	ENSG00000130175	19:11447618-11447692:+	5.52E-05	0.253153488	0.225485491
1964	PRKCSH	ENSG00000130175	19:11447789-11448221:+	5.72E-06	0.280464304	0.209156087
1965	PRKCSH	ENSG00000130175	19:11448291-11448539:+	0.00892417	0.101946118	0.081419371
1966	ELAVL3	ENSG00000196361	19:11466827-11480599:-	0.00162975	0.105776598	0.159194247
1967	ZNF653	ENSG00000161914	19:11487158-11487291:-	0.01668986	0.206358455	0.243057577
1968	MAN2B1	ENSG00000104774	19:12656687-12656948:-	0.00378395	0.164128112	0.131519707
1969	WDR83	ENSG00000123154	19:12670285-12670562:+	0.01370353	0.580980378	0.525944688
1970	WDR83	ENSG00000123154	19:12672914-12673007:+	0.00018903	0.347249355	0.27207274
1971	WDR83	ENSG00000123154	19:12673116-12673201:+	1.98E-05	0.378151415	0.292311588
1972	WDR83	ENSG00000123154	19:12673316-12675522:+	4.41E-09	0.364903683	0.253707688
1973	TNPO2	ENSG00000105576	19:12705599-12705681:-	0.00058928	0.102442887	0.075146539
1974	TNPO2	ENSG00000105576	19:12710773-12711295:-	0.00184326	0.109634721	0.087965188
1975	MAST1	ENSG00000105613	19:12866102-12866652:+	1.48E-05	0.484023529	0.361886987
1976	GCDH	ENSG00000105607	19:12896421-12896909:+	2.43E-09	0.164108663	0.095686569
1977	GCDH	ENSG00000105607	19:12897428-12897702:+	3.78E-05	0.138213141	0.09667261
1978	NFIX	ENSG00000008441	19:13081855-13087988:+	0.00058255	0.046214179	0.037782368
1979	NFIX	ENSG00000008441	19:13088136-13090298:+	0.00089493	0.027350207	0.022595546
1980	TRMT1	ENSG00000104907	19:13109838-13109914:-	0.00477924	0.182482226	0.144149274
1981	TRMT1	ENSG00000104907	19:13110001-13110157:-	0.04266995	0.145854099	0.124228396
1982	STX10	ENSG00000104915	19:13149897-13150138:-	0.03949033	0.426588573	0.392549662
1983	MRI1	ENSG00000037757	19:13768737-13768823:+	0.00109194	0.102288188	0.073538364
1984	DCAF15	ENSG00000132017	19:13956018-13956122:+	6.87E-06	0.160482112	0.105802838
1985	DCAF15	ENSG00000132017	19:13960391-13960464:+	0.00975279	0.457564878	0.383071078
1986	DCAF15	ENSG00000132017	19:13960580-13960939:+	1.39E-06	0.273456849	0.171233516
1987	RFX1	ENSG00000132005	19:13966751-13968564:-	0.03953664	0.161740116	0.13715739
1988	GIPC1	ENSG00000123159	19:14480778-14491655:-	2.77E-05	0.03194697	0.03900743
1989	DNAJB1	ENSG00000132002	19:14516170-14516465:-	5.88E-05	0.073593889	0.046607904
1990	TECR	ENSG00000099797	19:14564858-14564948:+	0.00732343	0.163036382	0.130817283
1991	TECR	ENSG00000099797	19:14564992-14565065:+	0.02397358	0.130909659	0.105592386
1992	ILVBL	ENSG00000105135	19:15115430-15115565:-	1.58E-06	0.339692415	0.240323197
1993	ILVBL	ENSG00000105135	19:15115671-15115849:-	6.16E-06	0.287316146	0.210422173
1994	ILVBL	ENSG00000105135	19:15116071-15116151:-	0.02209171	0.227212163	0.195043186
1995	ILVBL	ENSG00000105135	19:15122804-15122891:-	0.02650301	0.084219327	0.104987008

S/N	Gene	Ensembl ID	Position of retained intron	p-value	AD_IR_average	Cont_IR_average
1996	AKAP8	ENSG00000105127	19:15368322-15370145:-	0.00751397	0.109587396	0.148700334
1997	AKAP8L	ENSG00000011243	19:15399410-15400294:-	7.08E-11	0.138413952	0.215105584
1998	AKAP8L	ENSG00000011243	19:15403555-15403657:-	0.02027587	0.815758744	0.779727234
1999	WIZ	ENSG00000011451	19:15425768-15426981:-	0.03886799	0.159471074	0.130225289
2000	TPM4	ENSG00000167460	19:16093753-16095263:+	0.00300104	0.023853992	0.029993412
2001	RAB8A	ENSG00000167461	19:16129604-16132211:+	0.00904858	0.166697528	0.149497455
2002	EPS15L1	ENSG00000127527	19:16377254-16385128:-	0.00062985	0.100535198	0.081737844
2003	CHERP	ENSG00000085872	19:16520265-16520363:-	0.00040191	0.235000634	0.295912961
2004	CHERP	ENSG00000085872	19:16520507-16520825:-	0.00646965	0.243876683	0.293600987
2005	MYO9B	ENSG00000099331	19:17162466-17162987:+	2.56E-06	0.094669488	0.055336206
2006	OCEL1	ENSG00000099330	19:17228005-17228255:+	0.04568701	0.128634677	0.109376121
2007	AC010646.	ENSG00000269095	19:17236065-17240670:-	0.01132495	0.195495499	0.150244603
2008	BABAM1	ENSG00000105393	19:17275825-17276494:+	0.00994416	0.10691416	0.092082079
2009	BABAM1	ENSG00000105393	19:17276624-17276822:+	0.00011125	0.213905795	0.171220465
2010	BABAM1	ENSG00000105393	19:17276909-17278844:+	0.01533632	0.158475099	0.135961379
2011	ANO8	ENSG00000074855	19:17327569-17327688:-	0.02811017	0.212744648	0.177127642
2012	ANO8	ENSG00000074855	19:17331215-17331294:-	0.00130941	0.186269751	0.135917284
2013	ANO8	ENSG00000074855	19:17331411-17332929:-	0.00057126	0.13500186	0.097909461
2014	ANO8	ENSG00000074855	19:17333026-17333100:-	0.00094323	0.125412855	0.08925619
2015	MVB12A	ENSG00000141971	19:17424067-17424620:+	0.02356685	0.097239428	0.072858908
2016	SLC27A1	ENSG00000130304	19:17487529-17488847:+	0.01571273	0.088444145	0.075675939
2017	SLC27A1	ENSG00000130304	19:17489117-17497254:+	0.00168627	0.15189729	0.11845299
2018	SLC27A1	ENSG00000130304	19:17500632-17500711:+	0.00034506	0.153252157	0.121162549
2019	SLC27A1	ENSG00000130304	19:17500876-17501272:+	9.38E-05	0.340111354	0.284057909
2020	SLC27A1	ENSG00000130304	19:17501419-17504454:+	0.00035945	0.134384034	0.103607855
2021	PGLS	ENSG00000130313	19:17511960-17516172:+	0.00783796	0.132764255	0.105725621
2022	FCHO1	ENSG00000130475	19:17772741-17774238:+	0.00959073	0.162190107	0.136575122
2023	FCHO1	ENSG00000130475	19:17774478-17775055:+	0.00076476	0.320448439	0.252746627
2024	FCHO1	ENSG00000130475	19:17776686-17778136:+	0.0369604	0.295788061	0.269820181
2025	FCHO1	ENSG00000130475	19:17778228-17778608:+	0.02992704	0.251425394	0.222801912
2026	AC007192.	ENSG00000268173	19:18168546-18168725:+	0.00507958	0.103311594	0.085066465
2027	SSBP4	ENSG00000130511	19:18431862-18431999:+	0.00811621	0.1328424	0.104565203
2028	SSBP4	ENSG00000130511	19:18432214-18432558:+	9.46E-05	0.095657522	0.064077488
2029	C19orf60	ENSG00000006015	19:18589683-18590853:+	0.00372893	0.119796262	0.083559827
2030	TMEM59L	ENSG00000105696	19:18618272-18618374:+	0.01468278	0.286888622	0.237065921
2031	CRTC1	ENSG00000105662	19:18765528-18768484:+	0.03749167	0.227264165	0.192806878
2032	CRTC1	ENSG00000105662	19:18775821-18777170:+	0.00028506	0.114226067	0.076737975
2033	SUGP2	ENSG00000064607	19:18993740-18994365:-	0.00293348	0.280103944	0.260429665
2034	ARMC6	ENSG00000105676	19:19055928-19057415:+	0.00078805	0.280367829	0.232405365
2035	SLC25A42	ENSG00000181035	19:19104938-19105560:+	0.0431443	0.097737329	0.08395637
2036	SLC25A42	ENSG00000181035	19:19105727-19106268:+	0.00012526	0.10727054	0.070817638
2037	TMEM161,	ENSG00000064545	19:19121668-19121758:-	0.00680224	0.335541863	0.27555881
2038	TMEM161,	ENSG00000064545	19:19121819-19130155:-	0.00022615	0.161014401	0.123969316
2039	TMEM161,	ENSG00000064545	19:19132508-19132656:-	0.00320029	0.116480289	0.092711786
2040	TMEM161,	ENSG00000064545	19:19132754-19133129:-	2.82E-06	0.240104989	0.173272586
2041	RFXANK	ENSG00000064490	19:19197046-19197185:+	0.00038936	0.148734629	0.104902067
2042	RFXANK	ENSG00000064490	19:19198232-19198656:+	4.37E-07	0.200090613	0.120880112
2043	NR2C2AP	ENSG00000184162	19:19202881-19203022:-	0.00020659	0.273316061	0.218833164
2044	SUGP1	ENSG00000105705	19:19302388-19303347:-	0.00529114	0.142848312	0.127603706
2045	ATP13A1	ENSG00000105726	19:19647528-19647598:-	0.03953165	0.176385106	0.144672075
2046	ATP13A1	ENSG00000105726	19:19652720-19653783:-	0.00434536	0.102779893	0.127246209
2047	ZNF91	ENSG00000167232	19:23342265-23358429:-	0.00054145	0.293446738	0.214032395
2048	PDCD5	ENSG00000105185	19:32582232-32584949:+	0.03222575	0.109062072	0.08544696
2049	PDCD5	ENSG00000105185	19:32586929-32587252:+	0.01428968	0.253449009	0.28842413
2050	LRP3	ENSG00000130881	19:33204852-33205245:+	9.49E-07	0.133377563	0.096552226
2051	LSM14A	ENSG00000257103	19:34221738-34226407:+	1.53E-07	0.086527737	0.049639991
2052	LSM14A	ENSG00000257103	19:34221738-34227364:+	7.88E-07	0.086864378	0.049623029

S/N	Gene	Ensembl ID	Position of retained intron	p-value	AD_IR_average	Cont_IR_average
2053	LSM14A	ENSG00000257103	19:34226464-34227364:+	0.00067375	0.13843157	0.107205816
2054	ZNF302	ENSG00000089335	19:34683238-34683991:+	0.00623309	0.149935972	0.167018929
2055	GRAMD1A	ENSG00000089351	19:35009443-35009887:+	0.00193011	0.121244713	0.096969016
2056	GRAMD1A	ENSG00000089351	19:35011554-35013255:+	0.00074261	0.222801293	0.197446519
2057	GRAMD1A	ENSG00000089351	19:35013368-35013540:+	0.00239539	0.257414634	0.236481081
2058	GRAMD1A	ENSG00000089351	19:35022038-35023235:+	0.03005301	0.16153302	0.125911188
2059	GRAMD1A	ENSG00000089351	19:35023547-35026048:+	3.47E-06	0.116745349	0.085693199
2060	LGI4	ENSG00000153902	19:35125507-35126269:-	0.00746492	0.139328096	0.103382813
2061	LGI4	ENSG00000153902	19:35126775-35126852:-	0.00189959	0.245677238	0.177295953
2062	LGI4	ENSG00000153902	19:35131555-35131788:-	4.89E-06	0.113906112	0.060534762
2063	CTD-2527I	ENSG00000221857	19:35142771-35142916:+	1.35E-06	0.275826717	0.181661565
2064	TMEM147	ENSG00000105677	19:35545816-35545887:+	0.00010428	0.110830233	0.085339726
2065	KMT2B	ENSG00000272333	19:35723502-35723731:+	0.00814928	0.136316572	0.166407832
2066	KMT2B	ENSG00000272333	19:35728171-35728773:+	0.01198681	0.074823199	0.094550738
2067	U2AF1L4	ENSG00000161265	19:35744145-35744322:-	0.00016634	0.295110476	0.225836282
2068	LIN37	ENSG00000267796	19:35754319-35754392:+	0.01253284	0.188970068	0.14998539
2069	ARHGAP33	ENSG00000004777	19:35782869-35784171:+	0.02051248	0.269392524	0.305546805
2070	ARHGAP33	ENSG00000004777	19:35787072-35787167:+	0.03192896	0.23283215	0.19401203
2071	APLP1	ENSG00000105290	19:35878108-35878583:+	0.00066888	0.132494262	0.105447264
2072	ALKBH6	ENSG00000239382	19:36011045-36011403:-	0.01013703	0.292354171	0.254687338
2073	ALKBH6	ENSG00000239382	19:36011464-36013020:-	0.00121046	0.238870793	0.196351191
2074	POLR2I	ENSG00000105258	19:36114412-36114658:-	6.73E-07	0.097217488	0.065238296
2075	POLR2I	ENSG00000105258	19:36114713-36114797:-	0.00010345	0.345860695	0.266337558
2076	DPF1	ENSG00000011332	19:38216403-38217459:-	0.02074747	0.235755578	0.18467113
2077	DPF1	ENSG00000011332	19:38217591-38217797:-	3.82E-05	0.3975185	0.306091634
2078	YIF1B	ENSG00000167645	19:38307521-38307596:-	0.01996253	0.360093598	0.311075273
2079	FAM98C	ENSG00000130244	19:38403218-38403337:+	0.00226705	0.469292634	0.402103571
2080	FAM98C	ENSG00000130244	19:38403694-38404907:+	0.00017489	0.262818341	0.204822979
2081	FAM98C	ENSG00000130244	19:38405113-38405343:+	1.53E-06	0.369945695	0.277374286
2082	FAM98C	ENSG00000130244	19:38405421-38405518:+	8.20E-08	0.35745705	0.245132017
2083	FAM98C	ENSG00000130244	19:38405635-38406909:+	1.22E-05	0.468973805	0.384860312
2084	FAM98C	ENSG00000130244	19:38407077-38408750:+	1.19E-06	0.391516548	0.295118468
2085	ACTN4	ENSG00000130402	19:38706131-38708116:+	0.00034922	0.070965287	0.099768652
2086	ACTN4	ENSG00000130402	19:38727103-38728315:+	8.05E-05	0.052657222	0.042837479
2087	HNRNPL	ENSG00000104824	19:38840559-38843841:-	0.01900929	0.127358049	0.156979075
2088	SIRT2	ENSG00000068903	19:38893867-38899505:-	0.00900795	0.238471863	0.210516171
2089	SAMD4B	ENSG00000179134	19:39383291-39383498:+	0.00865601	0.552178744	0.511602377
2090	PAF1	ENSG00000006712	19:39389015-39389092:-	0.00095531	0.104001667	0.081262878
2091	SUPT5H	ENSG00000196235	19:39457740-39458293:+	0.02066896	0.045140178	0.072924712
2092	SUPT5H	ENSG00000196235	19:39457740-39458817:+	6.85E-06	0.059039298	0.092867149
2093	MAP3K10	ENSG00000130758	19:40204633-40205120:+	6.73E-06	0.082610409	0.059485222
2094	TTC9B	ENSG00000174521	19:40216272-40217186:-	0.00207974	0.316879427	0.26587754
2095	AKT2	ENSG00000105221	19:40236385-40237968:-	2.00E-05	0.27035653	0.222188896
2096	AKT2	ENSG00000105221	19:40238973-40240044:-	9.98E-05	0.344133854	0.276489299
2097	AKT2	ENSG00000105221	19:40240110-40241937:-	0.00601064	0.14570128	0.119841762
2098	SPTBN4	ENSG00000160460	19:40570728-40572018:+	0.02053876	0.360685009	0.403240584
2099	SPTBN4	ENSG00000160460	19:40572380-40575410:+	0.00101632	0.20844206	0.160449168
2100	ITPKC	ENSG00000086544	19:40737087-40737697:+	0.00632448	0.117267035	0.092423335
2101	EGLN2	ENSG00000269858	19:40806674-40807137:+	0.0081273	0.144160257	0.169782808
2102	HNRNPUL1	ENSG00000105323	19:41264798-41268222:+	0.0061859	0.183201252	0.156696371
2103	HNRNPUL1	ENSG00000105323	19:41304261-41305645:+	0.01427177	0.020837401	0.021103132
2104	ARHGEF1	ENSG00000076928	19:41898113-41898441:+	0.00017951	0.454607317	0.540519675
2105	ARHGEF1	ENSG00000076928	19:41905036-41905174:+	0.00077317	0.091643224	0.06790743
2106	ARHGEF1	ENSG00000076928	19:41905261-41905759:+	0.00089796	0.093341906	0.070145824
2107	ERF	ENSG00000105722	19:42249942-42250330:-	0.00475688	0.210717617	0.164359204
2108	CIC	ENSG00000079432	19:42294298-42294603:+	0.04515089	0.041094726	0.032042439
2109	PAFAH1B3	ENSG00000079462	19:42300092-42300170:-	0.00809325	0.118712279	0.089351244

S/N	Gene	Ensembl ID	Position of retained intron	p-value	AD_IR_average	Cont_IR_average
2110	TMEM145	ENSG00000167619	19:42315440-42316480:+	0.03362879	0.102027784	0.115314473
2111	TMEM145	ENSG00000167619	19:42316561-42316661:+	0.00525488	0.283940699	0.23010639
2112	MEGF8	ENSG00000105429	19:42361006-42362089:+	0.02914353	0.125757013	0.088085001
2113	LIPE	ENSG00000079435	19:42402075-42402606:-	0.00569867	0.127668151	0.103698014
2114	LIPE	ENSG00000079435	19:42405561-42406160:-	0.01270859	0.166660038	0.138719114
2115	LIPE	ENSG00000079435	19:42408121-42408231:-	0.03761502	0.098317542	0.146117248
2116	XRCC1	ENSG00000073050	19:43546106-43546594:-	0.02081895	0.108310902	0.085146439
2117	XRCC1	ENSG00000073050	19:43546727-43546883:-	0.00059556	0.231901355	0.179710006
2118	XRCC1	ENSG00000073050	19:43551687-43552016:-	0.04768473	0.182656433	0.160999904
2119	XRCC1	ENSG00000073050	19:43552908-43552981:-	0.01832233	0.196357754	0.163592836
2120	PINLYP	ENSG00000234465	19:43581364-43581562:+	0.01903138	0.106237733	0.082144938
2121	SMG9	ENSG00000105771	19:43733733-43734388:-	1.16E-06	0.116464076	0.080124592
2122	SMG9	ENSG00000105771	19:43734495-43737596:-	4.01E-06	0.457148195	0.377571455
2123	SMG9	ENSG00000105771	19:43737682-43738121:-	4.25E-06	0.202074555	0.153807836
2124	CEACAM19	ENSG00000186567	19:44681312-44682566:+	0.03355326	0.12935728	0.105296342
2125	TOMM40	ENSG00000130204	19:44901310-44903029:+	2.01E-09	0.289247012	0.203453491
2126	CLASRP	ENSG00000104859	19:45052892-45053097:+	8.99E-08	0.168975817	0.106440766
2127	PPP1R37	ENSG00000104866	19:45142458-45143520:+	0.00099232	0.142117504	0.109819147
2128	PPP1R37	ENSG00000104866	19:45143633-45144853:+	0.0113662	0.102113127	0.082551781
2129	PPP1R37	ENSG00000104866	19:45146049-45146389:+	0.00288776	0.126532348	0.088578966
2130	ERCC2	ENSG00000104884	19:45357543-45357629:-	0.04149918	0.089059061	0.073398089
2131	ERCC1	ENSG00000012061	19:45414034-45414351:-	0.03057806	0.043862112	0.036187204
2132	EML2	ENSG00000125746	19:45615889-45616460:-	0.00897615	0.152615904	0.137620792
2133	EML2	ENSG00000125746	19:45616558-45616764:-	0.03195686	0.126999285	0.117210248
2134	EML2	ENSG00000125746	19:45638634-45638844:-	0.03663192	0.196191888	0.184600097
2135	DMPK	ENSG00000104936	19:45771056-45771349:-	0.03998165	0.134930906	0.114910381
2136	DMPK	ENSG00000104936	19:45778641-45779263:-	2.62E-05	0.297809805	0.223013571
2137	SYMPK	ENSG00000125755	19:45823875-45825170:-	1.04E-09	0.242370866	0.15118239
2138	SYMPK	ENSG00000125755	19:45827918-45828969:-	0.0185135	0.102284491	0.082075504
2139	SYMPK	ENSG00000125755	19:45854507-45863057:-	0.04583157	0.122751605	0.144391414
2140	NOVA2	ENSG00000104967	19:45961153-45973266:-	0.00277575	0.089875572	0.118213429
2141	CCDC61	ENSG00000104983	19:46003501-46006558:+	0.0320811	0.144906096	0.119464342
2142	STRN4	ENSG00000090372	19:46720771-46721985:-	0.00504949	0.110300876	0.092748801
2143	STRN4	ENSG00000090372	19:46722072-46722241:-	0.04835612	0.123562785	0.110298539
2144	BBC3	ENSG00000105327	19:47221918-47226563:-	0.00369334	0.12513909	0.091929821
2145	DHX34	ENSG00000134815	19:47380992-47381185:+	1.03E-05	0.486423451	0.397056143
2146	DHX34	ENSG00000134815	19:47381324-47381979:+	0.00397193	0.554942012	0.486206182
2147	MEIS3	ENSG00000105419	19:47406526-47406887:-	0.02274719	0.179194622	0.144754148
2148	KPTN	ENSG00000118162	19:47480407-47480759:-	0.00210743	0.674387293	0.613351818
2149	NAPA	ENSG00000105402	19:47492119-47492960:-	0.03371602	0.108946633	0.096371031
2150	GLTSCR1	ENSG00000063169	19:47695474-47696450:+	0.03630635	0.139920243	0.116995179
2151	LIG1	ENSG00000105486	19:48136125-48137007:-	0.00693054	0.112900506	0.086644092
2152	SPHK2	ENSG00000063176	19:48626362-48627691:+	0.0064872	0.293979171	0.244317091
2153	SPHK2	ENSG00000063176	19:48628069-48628161:+	0.01150385	0.09518589	0.070223262
2154	CA11	ENSG00000063180	19:48638144-48638887:-	0.00815371	0.177806394	0.147902042
2155	CA11	ENSG00000063180	19:48644569-48645402:-	0.00087838	0.186637122	0.146588721
2156	MAMSTR	ENSG00000176909	19:48713770-48713859:-	0.01455706	0.252630204	0.207659488
2157	BCAT2	ENSG00000105552	19:48800100-48800186:-	0.00031628	0.107973924	0.066367167
2158	BCAT2	ENSG00000105552	19:48800297-48806516:-	5.51E-06	0.239535867	0.172995119
2159	BCAT2	ENSG00000105552	19:48806717-48806999:-	0.03026142	0.136022441	0.114298268
2160	BCAT2	ENSG00000105552	19:48807074-48807755:-	0.00066876	0.403372012	0.408738636
2161	PPP1R15A	ENSG00000087074	19:48872651-48873224:+	2.95E-08	0.047069386	0.031083362
2162	PPP1R15A	ENSG00000087074	19:48874898-48875613:+	3.56E-09	0.098395294	0.067415483
2163	BAX	ENSG00000087088	19:48955599-48955686:+	0.01812377	0.29271121	0.275678935
2164	GYS1	ENSG00000104812	19:48970709-48970927:-	0.00030617	0.213316289	0.137103418
2165	GYS1	ENSG00000104812	19:48982375-48982719:-	0.00755574	0.223967018	0.185471735
2166	GYS1	ENSG00000104812	19:48982837-48985460:-	0.02452616	0.257730583	0.23317401

S/N	Gene	Ensembl ID	Position of retained intron	p-value	AD_IR_average	Cont_IR_average
2167	GYS1	ENSG00000104812	19:48985605-48985849:-	0.02568912	0.147221605	0.117145755
2168	LIN7B	ENSG00000104863	19:49114441-49114848:+	0.00776747	0.159314491	0.125749649
2169	LIN7B	ENSG00000104863	19:49114967-49115259:+	0.00029696	0.119709745	0.082735971
2170	PPFIA3	ENSG00000177380	19:49143004-49145942:+	0.01877882	0.123394023	0.100622231
2171	PPFIA3	ENSG00000177380	19:49149718-49150079:+	0.01339147	0.105780799	0.088826656
2172	PIH1D1	ENSG00000104872	19:49447099-49447337:-	2.94E-06	0.273871207	0.200497622
2173	PIH1D1	ENSG00000104872	19:49447467-49447826:-	1.97E-06	0.212519666	0.153036335
2174	PIH1D1	ENSG00000104872	19:49447908-49448000:-	0.0361826	0.278306817	0.252664432
2175	RRAS	ENSG00000126458	19:49635660-49635733:-	0.00731251	0.156293105	0.108235813
2176	RRAS	ENSG00000126458	19:49635852-49636618:-	0.00312267	0.104095847	0.07181004
2177	IRF3	ENSG00000126456	19:49659833-49660712:-	1.60E-05	0.120282523	0.08886261
2178	PRMT1	ENSG00000126457	19:49686726-49688161:+	0.01725878	0.227260161	0.184266021
2179	CPT1C	ENSG00000169169	19:49710484-49710722:+	1.74E-05	0.424916561	0.340306312
2180	CPT1C	ENSG00000169169	19:49710857-49711808:+	4.56E-11	0.578316024	0.446555844
2181	AP2A1	ENSG00000196961	19:49798952-49799326:+	0.00997105	0.198982172	0.176615775
2182	MED25	ENSG00000104973	19:49832415-49834985:+	0.00271105	0.139712649	0.113377574
2183	PTOV1	ENSG00000104960	19:49855077-49856974:+	0.00068963	0.531420939	0.456043312
2184	PNKP	ENSG00000039650	19:49861365-49861448:-	0.02249332	0.438925976	0.361167662
2185	PNKP	ENSG00000039650	19:49864236-49864323:-	0.00124284	0.614349085	0.535317195
2186	AKT1S1	ENSG00000204673	19:49870060-49871546:-	0.00849891	0.146558796	0.123997265
2187	AKT1S1	ENSG00000204673	19:49871889-49872916:-	0.00238538	0.118649099	0.093146292
2188	TBC1D17	ENSG00000104946	19:49878572-49880278:+	0.00185894	0.269675317	0.229880727
2189	VRK3	ENSG00000105053	19:49976784-49979082:-	0.00100204	0.211698496	0.160685582
2190	VRK3	ENSG00000105053	19:49988492-49989638:-	0.00015207	0.119181332	0.087717604
2191	MYH14	ENSG00000105357	19:50281842-50286481:+	0.00237934	0.146165943	0.114989147
2192	MYH14	ENSG00000105357	19:50309177-50309639:+	0.00186846	0.099478541	0.071100088
2193	KCNC3	ENSG00000131398	19:50320349-50320592:-	0.00121544	0.142110695	0.10804271
2194	CTD-2545N	ENSG00000142539	19:50415826-50416395:+	0.00628016	0.410370683	0.346062638
2195	ASPDH	ENSG00000204653	19:50511773-50512135:-	0.00011563	0.519615744	0.418095909
2196	C19orf81	ENSG00000235034	19:50658608-50658946:+	0.00420813	0.361986512	0.298927506
2197	SPACA6	ENSG00000182310	19:51694555-51701657:+	9.84E-06	0.389262284	0.302294651
2198	SPACA6	ENSG00000182310	19:51701726-51702628:+	7.32E-05	0.380111951	0.308956355
2199	SPACA6	ENSG00000182310	19:51702652-51703020:+	1.33E-06	0.448573293	0.369925532
2200	SPACA6	ENSG00000182310	19:51703098-51703227:+	0.01095173	0.276338622	0.235494306
2201	SPACA6	ENSG00000182310	19:51703337-51704029:+	2.77E-05	0.209585273	0.176518127
2202	SPACA6	ENSG00000182310	19:51704186-51704269:+	0.0014222	0.261530528	0.206369116
2203	SPACA6	ENSG00000182310	19:51704480-51705089:+	1.22E-05	0.287428362	0.214066142
2204	AC074141.	ENSG00000283617	19:51877377-51878388:-	0.02325105	0.091983174	0.074062891
2205	AC074141.	ENSG00000283617	19:51878515-51880322:-	0.00271183	0.153663373	0.121236108
2206	CACNG7	ENSG00000105605	19:53914586-53915364:+	0.0018799	0.160697661	0.133236906
2207	TFPT	ENSG00000105619	19:54108244-54108325:-	0.00529168	0.173293576	0.140988444
2208	TFPT	ENSG00000105619	19:54110121-54114441:-	0.04443632	0.15335883	0.133572905
2209	TFPT	ENSG00000105619	19:54114700-54115246:-	0.01439291	0.246353321	0.211297687
2210	PRPF31	ENSG00000105618	19:54128377-54129056:+	0.00378452	0.113980309	0.092723327
2211	CNOT3	ENSG00000088038	19:54143749-54144005:+	0.00237077	0.120177618	0.093848666
2212	CNOT3	ENSG00000088038	19:54145817-54145909:+	0.00492951	0.114544006	0.085711858
2213	PPP1R12C	ENSG00000125503	19:55095940-55096050:-	7.50E-08	0.118935577	0.07283323
2214	PPP1R12C	ENSG00000125503	19:55096178-55096261:-	0.02705596	0.077146713	0.078023621
2215	PPP6R1	ENSG00000105063	19:55240300-55240944:-	0.00019945	0.140225634	0.098122081
2216	PPP6R1	ENSG00000105063	19:55241391-55241476:-	4.24E-06	0.300192251	0.182984758
2217	KMT5C	ENSG00000133247	19:55342851-55343679:+	0.00979765	0.300073226	0.336006701
2218	KMT5C	ENSG00000133247	19:55343843-55343977:+	0.0213555	0.108012898	0.085110397
2219	ISOC2	ENSG00000063241	19:55455330-55455635:-	0.00052732	0.113271645	0.091292832
2220	NAT14	ENSG00000090971	19:55485780-55486407:+	9.00E-05	0.370286793	0.297380152
2221	ZSCAN18	ENSG00000121413	19:58085376-58086173:-	5.56E-05	0.146581804	0.2066198
2222	ZSCAN18	ENSG00000121413	19:58086266-58086905:-	0.00167668	0.23017604	0.201618904
2223	ZSCAN18	ENSG00000121413	19:58087008-58087315:-	5.25E-05	0.158792705	0.122064823

S/N	Gene	Ensembl ID	Position of retained intron	p-value	AD_IR_average	Cont_IR_average
2224	A1BG	ENSG00000121410	19:58347029-58347352:-	0.0185622	0.124352012	0.098988388
2225	ZBTB45	ENSG00000119574	19:58514310-58516394:-	1.34E-05	0.230677196	0.15940114
2226	TRIM28	ENSG00000130726	19:58545537-58545763:+	0.00079881	0.102260563	0.076349351
2227	TRIM28	ENSG00000130726	19:58545896-58547375:+	0.01306454	0.134603439	0.120100782
2228	TRAPPC12	ENSG00000171853	2:3443891-3457620:+	0.01497159	0.107448869	0.083147903
2229	TRAPPC12	ENSG00000171853	2:3477795-3478845:+	0.00801479	0.277581123	0.241967701
2230	TRAPPC12	ENSG00000171853	2:3478933-3479218:+	8.15E-10	0.16283819	0.105696822
2231	ITGB1BP1	ENSG00000119185	2:9414256-9418625:-	0.00911892	0.095751091	0.077229955
2232	CPSF3	ENSG00000119203	2:9467776-9471342:+	0.01103651	0.109239021	0.088155351
2233	DDX1	ENSG00000079785	2:15627145-15628444:+	0.00239117	0.128252744	0.091351515
2234	DDX1	ENSG00000079785	2:15628517-15628637:+	0.02017142	0.175661049	0.148578705
2235	DDX1	ENSG00000079785	2:15628710-15628796:+	0.03098834	0.15642329	0.136497768
2236	FKBP1B	ENSG00000119782	2:24049886-24053901:+	9.24E-05	0.155093267	0.108020692
2237	ADCY3	ENSG00000138031	2:24820114-24820723:-	0.01889713	0.346950439	0.312947974
2238	ADCY3	ENSG00000138031	2:24827608-24827901:-	0.03607719	0.167271699	0.142289801
2239	DNMT3A	ENSG00000119772	2:25239215-25240301:-	0.01060154	0.210844362	0.178751783
2240	DTNB	ENSG00000138101	2:25379323-25383835:-	6.52E-05	0.048572636	0.034286828
2241	DPYSL5	ENSG00000157851	2:26934734-26940030:+	0.00188532	0.168095885	0.147153445
2242	AGBL5	ENSG00000084693	2:27056792-27057302:+	0.01826153	0.319484683	0.272027147
2243	AGBL5	ENSG00000084693	2:27057438-27058399:+	0.00690418	0.300407873	0.250958314
2244	AGBL5	ENSG00000084693	2:27058602-27059189:+	6.86E-06	0.188128524	0.131056066
2245	AGBL5	ENSG00000084693	2:27067646-27068631:+	0.00025957	0.581538171	0.502321532
2246	KHK	ENSG00000138030	2:27099284-27099419:+	0.00686027	0.398563951	0.461961273
2247	CGREF1	ENSG00000138028	2:27102591-27104286:-	0.02080455	0.15515434	0.121557738
2248	PREB	ENSG00000138073	2:27133337-27133531:-	0.01420941	0.31993022	0.378293831
2249	SLC5A6	ENSG00000138074	2:27201453-27201665:-	0.00138082	0.213716978	0.172985129
2250	MPV17	ENSG00000115204	2:27311951-27312213:-	2.45E-05	0.336435179	0.266881338
2251	MPV17	ENSG00000115204	2:27312246-27312493:-	0.00623811	0.394873707	0.362726935
2252	GTF3C2	ENSG00000115207	2:27328189-27328467:-	0.01503408	0.164839709	0.199990795
2253	IFT172	ENSG00000138002	2:27444521-27445013:-	0.00116588	0.113557039	0.084218691
2254	IFT172	ENSG00000138002	2:27445843-27445928:-	0.01526745	0.102188811	0.085798486
2255	IFT172	ENSG00000138002	2:27447634-27447811:-	0.00038856	0.086805083	0.066848153
2256	IFT172	ENSG00000138002	2:27449031-27449293:-	0.03938347	0.11271012	0.09712651
2257	SLC4A1AP	ENSG00000163798	2:27688020-27688699:+	0.00232151	0.115818915	0.097004694
2258	TTC27	ENSG00000018699	2:32812615-32817456:+	7.10E-08	0.14167999	0.077299408
2259	CEBPZ	ENSG00000115816	2:37202865-37202949:-	0.00655168	0.366908537	0.410200442
2260	CEBPZ	ENSG00000115816	2:37212392-37213863:-	0.00026344	0.135479273	0.096473794
2261	CEBPZ	ENSG00000115816	2:37214952-37216139:-	5.97E-05	0.096683943	0.144733344
2262	SRSF7	ENSG00000115875	2:38745187-38746693:-	1.19E-06	0.027044815	0.023122795
2263	MAP4K3	ENSG00000011566	2:39258587-39260605:-	5.88E-07	0.090030817	0.046704757
2264	DYNC2LI1	ENSG00000138036	2:43805246-43809704:+	1.12E-08	0.175204963	0.096287616
2265	TTC7A	ENSG00000068724	2:46994514-46995135:+	0.01353317	0.093557423	0.070914099
2266	TTC7A	ENSG00000068724	2:47024359-47029223:+	0.00596771	0.092266565	0.067066672
2267	MSH2	ENSG00000095002	2:47480871-47482778:+	0.00274782	0.212312502	0.173236122
2268	MSH6	ENSG00000116062	2:47801155-47803419:+	0.00649065	0.206664854	0.26645626
2269	MSH6	ENSG00000116062	2:47806651-47806778:+	0.03681097	0.114564045	0.094209079
2270	RPS27A	ENSG00000143947	2:55232708-55232807:+	0.00035236	0.113509999	0.076071112
2271	AHSA2	ENSG00000173209	2:61184800-61185501:+	0.00119345	0.13611986	0.103626821
2272	AHSA2	ENSG00000173209	2:61186497-61186616:+	0.0029077	0.20725223	0.174690301
2273	USP34	ENSG00000115464	2:61189069-61190270:-	0.04144787	0.290642037	0.316602351
2274	USP34	ENSG00000115464	2:61190414-61190517:-	8.87E-07	0.337978012	0.420826208
2275	USP34	ENSG00000115464	2:61190658-61192900:-	0.00033159	0.09832427	0.065606284
2276	USP34	ENSG00000115464	2:61192980-61203139:-	0.00152428	0.095892321	0.093589431
2277	ETAA1	ENSG00000143971	2:67405335-67409910:+	0.01609603	0.138268645	0.099859066
2278	TIA1	ENSG00000116001	2:70216292-70216403:-	0.00036073	0.131285979	0.094737585
2279	TIA1	ENSG00000116001	2:70216994-70224553:-	0.02185877	0.123191548	0.107864684
2280	AC007040.	ENSG00000258881	2:70989816-70991829:-	0.00210231	0.194819741	0.164346735

S/N	Gene	Ensembl ID	Position of retained intron	p-value	AD_IR_average	Cont_IR_average
2281	NAGK	ENSG00000124357	2:71068712-71070501:+	6.96E-06	0.272542451	0.236161694
2282	NAGK	ENSG00000124357	2:71070586-71070740:+	1.12E-05	0.257429695	0.209082735
2283	ZNF638	ENSG00000075292	2:71427414-71431326:+	0.01126245	0.04663142	0.040481502
2284	SFXN5	ENSG00000144040	2:72961248-72968447:-	0.01118229	0.090646298	0.066957919
2285	SMYD5	ENSG00000135632	2:73223106-73223425:+	0.00992852	0.097390861	0.078549436
2286	SMYD5	ENSG00000135632	2:73223532-73223946:+	1.44E-05	0.143950241	0.096685027
2287	SMYD5	ENSG00000135632	2:73224003-73224865:+	0.00338529	0.13109496	0.104083497
2288	SMYD5	ENSG00000135632	2:73225701-73225795:+	0.02213703	0.133763627	0.108881742
2289	PRADC1	ENSG00000135617	2:73228574-73228794:-	0.04733453	0.118340434	0.099435096
2290	PRADC1	ENSG00000135617	2:73228962-73229460:-	0.00017686	0.12082899	0.090856318
2291	PRADC1	ENSG00000135617	2:73229570-73230112:-	0.00015379	0.106702323	0.076983953
2292	TPRKB	ENSG00000144034	2:73730029-73730559:-	1.33E-05	0.179532477	0.130446956
2293	TPRKB	ENSG00000144034	2:73730736-73732162:-	4.62E-06	0.167551356	0.121380722
2294	TPRKB	ENSG00000144034	2:73732285-73732583:-	0.0327036	0.057912663	0.053352468
2295	DGUOK	ENSG00000114956	2:73950732-73958145:+	0.00054248	0.045215148	0.033870327
2296	DCTN1	ENSG00000204843	2:74365241-74365514:-	0.00015491	0.160145294	0.129131555
2297	WDR54	ENSG00000005448	2:74425237-74425416:+	0.00208236	0.106425239	0.080914624
2298	WDR54	ENSG00000005448	2:74425491-74425569:+	0.0038799	0.105107759	0.078909081
2299	RTKN	ENSG00000114993	2:74428737-74428847:-	0.04859506	0.132849446	0.114082534
2300	TTC31	ENSG00000115282	2:74490473-74490655:+	0.02661684	0.115821543	0.145157326
2301	PCGF1	ENSG00000115289	2:74507147-74507575:-	2.12E-08	0.421662878	0.297439221
2302	AUP1	ENSG00000115307	2:74528321-74528416:-	2.35E-06	0.174993766	0.143242414
2303	AUP1	ENSG00000115307	2:74528935-74529131:-	0.00025899	0.659754878	0.595326286
2304	AUP1	ENSG00000115307	2:74529282-74529361:-	0.00287667	0.127052648	0.095645736
2305	HTRA2	ENSG00000115317	2:74531702-74531855:+	0.00020666	0.1826244	0.147464566
2306	HTRA2	ENSG00000115317	2:74531925-74532618:+	0.00036085	0.084428111	0.066114829
2307	GCFC2	ENSG00000005436	2:75664783-75665928:-	4.02E-08	0.337988256	0.242283374
2308	GCFC2	ENSG00000005436	2:75666053-75670137:-	1.55E-05	0.327296654	0.247713003
2309	GCFC2	ENSG00000005436	2:75670284-75671949:-	0.00032039	0.369738037	0.310303714
2310	GCFC2	ENSG00000005436	2:75672016-75673443:-	0.00070472	0.342724122	0.283364419
2311	ELMOD3	ENSG00000115459	2:85377474-85389750:+	0.0023849	0.186693954	0.142389088
2312	ELMOD3	ENSG00000115459	2:85389827-85390137:+	0.02196392	0.163845989	0.147824903
2313	ELMOD3	ENSG00000115459	2:85390265-85390759:+	0.00465732	0.380035024	0.328813364
2314	GGCX	ENSG00000115486	2:85550126-85550554:-	4.54E-05	0.130791123	0.082774428
2315	GGCX	ENSG00000115486	2:85550750-85550924:-	0.00048159	0.253997118	0.190415787
2316	GGCX	ENSG00000115486	2:85551072-85551479:-	0.00013342	0.132467457	0.092503997
2317	TMEM150,	ENSG00000168890	2:85599317-85599524:-	0.04476751	0.172536245	0.148221075
2318	C2orf68	ENSG00000168887	2:85609067-85609434:-	0.02857298	0.545938585	0.589134364
2319	PTCD3	ENSG00000132300	2:86130737-86131077:+	0.00084522	0.208789283	0.14453086
2320	PTCD3	ENSG00000132300	2:86131106-86132317:+	0.0098566	0.114437637	0.090977921
2321	PTCD3	ENSG00000132300	2:86132424-86133177:+	0.01268294	0.221644284	0.172398747
2322	PTCD3	ENSG00000132300	2:86136562-86136981:+	3.26E-05	0.134142779	0.188762391
2323	KDM3A	ENSG00000115548	2:86482102-86482457:+	8.72E-05	0.235044712	0.334147297
2324	KDM3A	ENSG00000115548	2:86482694-86483986:+	0.0205927	0.212890143	0.265281574
2325	KDM3A	ENSG00000115548	2:86489437-86489519:+	0.01753034	0.125353618	0.100863401
2326	MRPS5	ENSG00000144029	2:95101723-95104639:-	0.00093395	0.420231159	0.333969091
2327	FAHD2A	ENSG00000115042	2:95407157-95410526:+	0.030408	0.270735878	0.222595343
2328	FAHD2A	ENSG00000115042	2:95412542-95412676:+	0.01059516	0.647133122	0.600404338
2329	ANKRD23	ENSG00000163126	2:96840095-96840231:-	0.02463696	0.247700332	0.208846122
2330	SEMA4C	ENSG00000168758	2:96861455-96861578:-	0.04920092	0.084687991	0.099202578
2331	SEMA4C	ENSG00000168758	2:96861894-96863681:-	0.01178852	0.148911726	0.178699243
2332	SEMA4C	ENSG00000168758	2:96867923-96869875:-	0.01114583	0.215023782	0.251970109
2333	ACTR1B	ENSG00000115073	2:97656960-97657151:-	1.31E-05	0.26851263	0.190362117
2334	ACTR1B	ENSG00000115073	2:97658643-97658878:-	0.00670175	0.421881354	0.357342325
2335	ACTR1B	ENSG00000115073	2:97659477-97660570:-	0.03939584	0.195165094	0.177497534
2336	ACTR1B	ENSG00000115073	2:97660646-97661881:-	0.00281611	0.221237473	0.176301721
2337	ACTR1B	ENSG00000115073	2:97661946-97663842:-	9.12E-05	0.417140856	0.322050078

S/N	Gene	Ensembl ID	Position of retained intron	p-value	AD_IR_average	Cont_IR_average
2338	TMEM131	ENSG00000075568	2:97772424-97775842:-	0.0480963	0.104132549	0.087044029
2339	MITD1	ENSG00000158411	2:99170652-99171342:-	0.00011993	0.202588362	0.166223552
2340	MITD1	ENSG00000158411	2:99171429-99171509:-	6.48E-05	0.34259298	0.277134312
2341	REV1	ENSG00000135945	2:99402346-99402643:-	0.00439964	0.29250222	0.23594883
2342	REV1	ENSG00000135945	2:99406106-99406324:-	0.00035814	0.100006843	0.148410308
2343	CNOT11	ENSG00000158435	2:101266879-101269039:+	0.01301359	0.147355227	0.12376894
2344	RNF149	ENSG00000163162	2:101277281-101281858:-	0.00964888	0.105944756	0.086763981
2345	RNF149	ENSG00000163162	2:101282057-101286080:-	0.03627081	0.166802099	0.143345068
2346	MAP4K4	ENSG00000071054	2:101864051-101864929:+	0.04655925	0.0869793	0.086185011
2347	MAP4K4	ENSG00000071054	2:101869797-101870294:+	0.00080962	0.159725554	0.117404449
2348	UXS1	ENSG00000115652	2:106096821-106098715:-	0.03258847	0.087946516	0.07731673
2349	UXS1	ENSG00000115652	2:106098773-106101057:-	0.01934066	0.107104288	0.092397249
2350	BIN1	ENSG00000136717	2:127050912-127051153:-	0.01283163	0.152502595	0.120112
2351	BIN1	ENSG00000136717	2:127051243-127059010:-	0.00015828	0.242461976	0.215224948
2352	BIN1	ENSG00000136717	2:127064018-127068162:-	0.04974669	0.083618116	0.063195123
2353	BIN1	ENSG00000136717	2:127064018-127068923:-	3.65E-05	0.077574386	0.058523827
2354	WDR33	ENSG00000136709	2:127709856-127713582:-	0.00257919	0.086019754	0.106529714
2355	SMPD4	ENSG00000136699	2:130153935-130154276:-	0.00587878	0.099705605	0.125149992
2356	MZT2B	ENSG00000152082	2:130182452-130190468:+	9.29E-05	0.032967274	0.025793404
2357	MZT2B	ENSG00000152082	2:130183966-130190468:+	8.06E-05	0.030891906	0.024270385
2358	PTPN18	ENSG00000072135	2:130359492-130359607:+	0.01201679	0.240367313	0.2030558
2359	ARHGEF4	ENSG00000136002	2:131040440-131041229:+	0.00618753	0.213019101	0.176423938
2360	ARHGEF4	ENSG00000136002	2:131045446-131046037:+	0.01277052	0.319258963	0.272528662
2361	MZT2A	ENSG00000173272	2:131492024-131492206:-	0.00035282	0.200117005	0.143423292
2362	UBXN4	ENSG00000144224	2:135742011-135748266:+	0.0275804	0.115887579	0.097691834
2363	ORC4	ENSG00000115947	2:147938213-147938297:-	0.009432	0.132664289	0.098815069
2364	FMNL2	ENSG00000157827	2:152575244-152578887:+	0.04139455	0.081827912	0.11077033
2365	WDSUB1	ENSG00000196151	2:159236190-159248371:-	0.0028825	0.297584727	0.244501826
2366	BAZ2B	ENSG00000123636	2:159320418-159324810:-	0.00846719	0.274221554	0.201727012
2367	FASTKD1	ENSG00000138399	2:169537340-169538012:-	0.02903925	0.08175625	0.084877008
2368	OSBPL6	ENSG00000079156	2:178394435-178395450:+	0.00077351	0.1091876	0.074926545
2369	PRKRA	ENSG00000180228	2:178432254-178436144:-	0.001936	0.099721918	0.078377313
2370	PRKRA	ENSG00000180228	2:178436319-178441609:-	0.00225454	0.157766344	0.122485645
2371	SSFA2	ENSG00000138434	2:181892660-181896029:+	0.00403589	0.124765323	0.092185169
2372	WDR75	ENSG00000115368	2:189468569-189469343:+	4.61E-09	0.096475441	0.056887441
2373	WDR75	ENSG00000115368	2:189469439-189470075:+	0.04646943	0.160942455	0.139322901
2374	OSGEPL1	ENSG00000128694	2:189750656-189752652:-	0.04091493	0.169129773	0.142031995
2375	HIBCH	ENSG00000198130	2:190205232-190208879:-	0.0144548	0.172439632	0.140158366
2376	HIBCH	ENSG00000198130	2:190208913-190212955:-	0.00918396	0.094223391	0.073516088
2377	HIBCH	ENSG00000198130	2:190249726-190252161:-	0.00010828	0.083736067	0.04905475
2378	SF3B1	ENSG00000115524	2:197398581-197398968:-	0.00184984	0.008608908	0.007277096
2379	HSPE1	ENSG00000115541	2:197503128-197503208:+	0.01272708	0.139808323	0.115375901
2380	CLK1	ENSG00000013441	2:200859746-200860124:-	2.08E-06	0.633881122	0.717176714
2381	CLK1	ENSG00000013441	2:200860215-200861237:-	5.73E-06	0.581813515	0.65626
2382	STRADB	ENSG00000082146	2:201478191-201478356:+	0.0088034	0.088590818	0.111239692
2383	TMEM237	ENSG00000155755	2:201626147-201627320:-	0.00219798	0.094490399	0.072953436
2384	ALS2	ENSG00000003393	2:201718210-201723042:-	0.00606301	0.091736339	0.134365129
2385	NOP58	ENSG00000055044	2:202300137-202300233:+	0.00030972	0.283923488	0.29294313
2386	CTDSP1	ENSG00000144579	2:218403417-218404296:+	0.01544724	0.122604091	0.146609634
2387	BCS1L	ENSG00000074582	2:218661307-218661405:+	0.00161292	0.06769763	0.093321691
2388	BCS1L	ENSG00000074582	2:218662260-218662509:+	0.00720888	0.15831585	0.190627551
2389	RNF25	ENSG00000163481	2:218668146-218668238:-	0.00032668	0.236220915	0.189497449
2390	TTLL4	ENSG00000135912	2:218748934-218749252:+	0.00949366	0.180117527	0.138058173
2391	CNPPD1	ENSG00000115649	2:219174207-219174777:-	3.89E-05	0.078504356	0.056906785
2392	ZFAND2B	ENSG00000158552	2:219208348-219208425:+	0.00091496	0.17311226	0.128803523
2393	ABCB6	ENSG00000115657	2:219210475-219210710:-	0.00681418	0.210556268	0.175445826
2394	ABCB6	ENSG00000115657	2:219212491-219213007:-	9.66E-05	0.274717305	0.215758296

S/N	Gene	Ensembl ID	Position of retained intron	p-value	AD_IR_average	Cont_IR_average
2395	ABCB6	ENSG00000115657	2:219214186-219214388:-	7.51E-06	0.699972829	0.620184649
2396	ANKZF1	ENSG00000163516	2:219232683-219233078:+	0.0073073	0.080629591	0.096299112
2397	ANKZF1	ENSG00000163516	2:219233191-219233285:+	0.00130586	0.185150188	0.239563468
2398	ANKZF1	ENSG00000163516	2:219233433-219233714:+	0.00033908	0.141179516	0.186969734
2399	DNPEP	ENSG00000123992	2:219382139-219383130:-	0.00268843	0.134307077	0.102635496
2400	DNPEP	ENSG00000123992	2:219384443-219385423:-	0.00042101	0.197475922	0.154859657
2401	DNPEP	ENSG00000123992	2:219385706-219385967:-	0.00269659	0.118720259	0.088578512
2402	SPEG	ENSG00000072195	2:219464608-219466056:+	0.00994059	0.098781755	0.081629962
2403	SPEG	ENSG00000072195	2:219481456-219481637:+	0.0086405	0.114556301	0.08168875
2404	GMPPA	ENSG00000144591	2:219504213-219505227:+	0.03949659	0.456109744	0.428514688
2405	CHPF	ENSG00000123989	2:219542189-219543224:-	0.00021471	0.092322901	0.063602919
2406	TMEM198	ENSG00000188760	2:219544893-219547505:+	0.00593599	0.20883084	0.164183591
2407	TMEM198	ENSG00000188760	2:219548081-219549151:+	2.49E-05	0.198847223	0.139062934
2408	TMEM198	ENSG00000188760	2:219549354-219549716:+	1.84E-05	0.18396687	0.119110017
2409	OBSL1	ENSG00000124006	2:219551798-219552111:-	0.00025023	0.163583955	0.111207475
2410	OBSL1	ENSG00000124006	2:219552216-219552535:-	3.42E-05	0.396982256	0.303545675
2411	OBSL1	ENSG00000124006	2:219557618-219557822:-	0.02080838	0.218105773	0.246047974
2412	STK11IP	ENSG00000144589	2:219609540-219611603:+	0.01853292	0.079980461	0.100821777
2413	FARSB	ENSG00000116120	2:222624479-222624713:-	0.00234883	0.078279748	0.135077821
2414	USP40	ENSG00000085982	2:233477503-233481202:-	0.02352324	0.146687262	0.115215288
2415	LRRFIP1	ENSG00000124831	2:237756187-237760063:+	0.00798249	0.039092823	0.040387424
2416	SCLY	ENSG00000132330	2:238094522-238096800:+	0.00727181	0.327784841	0.272653156
2417	ILKAP	ENSG00000132323	2:238185287-238188130:-	0.0205711	0.107004609	0.089334892
2418	HES6	ENSG00000144485	2:238239251-238239486:-	0.0223202	0.225089232	0.180284305
2419	HES6	ENSG00000144485	2:238239747-238239824:-	0.00296237	0.117311512	0.088490816
2420	NDUFA10	ENSG00000130414	2:240011696-240014738:-	0.0009956	0.402496256	0.302432714
2421	GPC1	ENSG00000063660	2:240465648-240466057:+	9.36E-06	0.141807342	0.08177157
2422	RNPEPL1	ENSG00000142327	2:240569114-240572422:+	3.16E-05	0.24991858	0.178858539
2423	RNPEPL1	ENSG00000142327	2:240573261-240573774:+	0.0225162	0.168110716	0.135263703
2424	RNPEPL1	ENSG00000142327	2:240574348-240574514:+	0.02082999	0.369541463	0.330313974
2425	RNPEPL1	ENSG00000142327	2:240575610-240576534:+	0.02612297	0.165220098	0.187623166
2426	CAPN10	ENSG00000142330	2:240591011-240591932:+	6.65E-07	0.175275707	0.117755797
2427	CAPN10	ENSG00000142330	2:240595304-240596318:+	0.0122935	0.346700732	0.371576338
2428	CAPN10	ENSG00000142330	2:240596521-240596680:+	1.60E-05	0.198081605	0.241475247
2429	KIF1A	ENSG00000130294	2:240726940-240728388:-	0.0219998	0.013858248	0.017288984
2430	MTERF4	ENSG00000122085	2:241075682-241089187:-	0.00100818	0.452778829	0.386539584
2431	SNED1	ENSG00000162804	2:241082364-241087391:+	0.01580261	0.2973325	0.259657727
2432	PPP1R7	ENSG00000115685	2:241166441-241167044:+	0.00096076	0.038954691	0.028961701
2433	Sep-02	ENSG00000168385	2:241315982-241324215:+	0.03840487	0.096720327	0.076274571
2434	FARP2	ENSG00000006607	2:241463468-241463898:+	0.00221525	0.219540741	0.169020977
2435	FARP2	ENSG00000006607	2:241483533-241484241:+	2.41E-07	0.216857778	0.15276817
2436	FARP2	ENSG00000006607	2:241484331-241489961:+	0.00785963	0.445201402	0.439956234
2437	FARP2	ENSG00000006607	2:241490044-241491060:+	0.00115365	0.220430829	0.176159918
2438	FARP2	ENSG00000006607	2:241491179-241491515:+	0.00458204	0.214545876	0.174122851
2439	FARP2	ENSG00000006607	2:241491679-241492928:+	0.00035648	0.24616243	0.192889751
2440	FARP2	ENSG00000006607	2:241493036-241493292:+	6.57E-05	0.263787934	0.215075548
2441	STK25	ENSG00000115694	2:241498349-241498638:-	0.00594064	0.145172365	0.123140974
2442	BOK	ENSG00000176720	2:241570288-241572296:+	0.00884544	0.248483318	0.197374539
2443	ATG4B	ENSG00000168397	2:241654647-241655270:+	0.00085827	0.229075221	0.174981003
2444	ATG4B	ENSG00000168397	2:241666838-241668142:+	0.00049667	0.073348218	0.097985092
2445	ATG4B	ENSG00000168397	2:241671405-241672190:+	0.00206033	0.572716183	0.510432208
2446	DTYMK	ENSG00000168393	2:241678649-241680228:-	0.02754339	0.137509406	0.152408873
2447	DTYMK	ENSG00000168393	2:241680319-241685768:-	0.04420805	0.2470077	0.20533819
2448	RBCK1	ENSG00000125826	20:421031-422126:+	0.00274342	0.675037268	0.632626325
2449	RBCK1	ENSG00000125826	20:428589-428950:+	0.02794731	0.096607046	0.110743094
2450	SIRPA	ENSG00000198053	20:1922645-1924763:+	0.04078748	0.108219666	0.082017895
2451	SIRPA	ENSG00000198053	20:1934754-1937319:+	0.00095254	0.109836055	0.082079488

S/N	Gene	Ensembl ID	Position of retained intron	p-value	AD_IR_average	Cont_IR_average
2452	IDH3B	ENSG00000101365	20:2659793-2660029:-	0.01315061	0.135153927	0.111338708
2453	PCED1A	ENSG00000132635	20:2835709-2836038:-	0.04762859	0.127102288	0.115625387
2454	PCED1A	ENSG00000132635	20:2836314-2838231:-	0.00181724	0.304780037	0.264318481
2455	PCED1A	ENSG00000132635	20:2839271-2839788:-	0.00297562	0.390090585	0.334317234
2456	PCED1A	ENSG00000132635	20:2839933-2840210:-	0.00056963	0.287459927	0.227514039
2457	PTPRA	ENSG00000132670	20:2864287-2864364:+	0.00778561	0.074103927	0.094678826
2458	LZTS3	ENSG00000088899	20:3167181-3167737:-	0.01493957	0.338805037	0.328148273
2459	CDC25B	ENSG00000101224	20:3801388-3801721:+	0.02472666	0.291296987	0.267110766
2460	CDC25B	ENSG00000101224	20:3803537-3804568:+	0.02501062	0.133236448	0.11267243
2461	NDUFAF5	ENSG00000101247	20:13814515-13816462:+	0.01841159	0.056407127	0.047570461
2462	RRBP1	ENSG00000125844	20:17619728-17620298:-	0.01054615	0.130289188	0.10941784
2463	CRNKL1	ENSG00000101343	20:20036362-20037322:-	0.00725987	0.097107249	0.07783033
2464	KIZ	ENSG00000088970	20:21232830-21244244:+	5.15E-15	0.189652427	0.106704057
2465	KIZ	ENSG00000088970	20:21244288-21246478:+	5.27E-06	0.41060878	0.320885601
2466	ACSS1	ENSG00000154930	20:25012664-25012811:-	0.00167922	0.302514991	0.245875695
2467	PYGB	ENSG00000100994	20:25274723-25276645:+	0.01758549	0.12569805	0.111331391
2468	PYGB	ENSG00000100994	20:25292613-25294157:+	0.00030467	0.121524491	0.091506055
2469	ABHD12	ENSG00000100997	20:25300884-25302218:-	4.79E-07	0.111518572	0.076736556
2470	ABHD12	ENSG00000100997	20:25303628-25306832:-	1.02E-10	0.101403216	0.058485212
2471	NINL	ENSG00000101004	20:25455786-25458382:-	0.049469	0.31910578	0.269718297
2472	NINL	ENSG00000101004	20:25480267-25481967:-	0.00792345	0.122727613	0.094296083
2473	HM13	ENSG00000101294	20:31566295-31568077:+	0.00348984	0.08276811	0.071584269
2474	ID1	ENSG00000125968	20:31605813-31606052:+	0.01037586	0.071901513	0.0950085
2475	TM9SF4	ENSG00000101337	20:32150875-32155102:+	0.00714631	0.232601139	0.215058205
2476	TM9SF4	ENSG00000101337	20:32158514-32159991:+	0.00217694	0.101450177	0.081902035
2477	CDK5RAP1	ENSG00000101391	20:33359123-33360350:-	1.64E-12	0.342121044	0.215985716
2478	CDK5RAP1	ENSG00000101391	20:33372697-33374114:-	0.01687845	0.382658195	0.340364169
2479	SNTA1	ENSG00000101400	20:33410331-33412295:-	0.02715263	0.123326921	0.099210887
2480	RALY	ENSG00000125970	20:34073635-34073818:+	0.00017675	0.095812083	0.067782969
2481	RALY	ENSG00000125970	20:34073635-34075873:+	5.41E-05	0.093657844	0.072397968
2482	RALY	ENSG00000125970	20:34073866-34075873:+	0.00197045	0.095221832	0.07421853
2483	PIGU	ENSG00000101464	20:34560979-34575103:-	8.11E-08	0.116122918	0.069810142
2484	GGT7	ENSG00000131067	20:34861562-34862813:-	0.00192673	0.091306217	0.068459009
2485	GGT7	ENSG00000131067	20:34862965-34863312:-	0.02479737	0.121337068	0.104785855
2486	ACSS2	ENSG00000131069	20:34920709-34921005:+	0.00441522	0.462570634	0.422248532
2487	ACSS2	ENSG00000131069	20:34921139-34921329:+	0.00506694	0.481757244	0.429040143
2488	ACSS2	ENSG00000131069	20:34921462-34921543:+	0.00623564	0.31828172	0.264984597
2489	ACSS2	ENSG00000131069	20:34921600-34921785:+	0.00184445	0.238343949	0.193241806
2490	ACSS2	ENSG00000131069	20:34921866-34923322:+	0.00925743	0.16792865	0.148971391
2491	ACSS2	ENSG00000131069	20:34925766-34926104:+	0.00022047	0.136751706	0.106764958
2492	ACSS2	ENSG00000131069	20:34926281-34926876:+	2.29E-06	0.124573651	0.091467542
2493	ACSS2	ENSG00000131069	20:34926951-34927086:+	7.16E-06	0.11904513	0.089339429
2494	GSS	ENSG00000100983	20:34928951-34929400:-	0.00124537	0.269348596	0.200325032
2495	GSS	ENSG00000100983	20:34929590-34931335:-	0.00198801	0.130622872	0.090472906
2496	MYH7B	ENSG00000078814	20:35001158-35001244:+	0.02143918	0.185834097	0.143015454
2497	MYH7B	ENSG00000078814	20:35001526-35001947:+	0.01552788	0.290493589	0.222856491
2498	TRPC4AP	ENSG00000100991	20:35003283-35003409:-	0.00880595	0.11816578	0.091280561
2499	UQCC1	ENSG00000101019	20:35304069-35306665:-	0.00712941	0.2741135	0.233407538
2500	CEP250	ENSG00000126001	20:35474052-35475501:+	0.00592074	0.059114337	0.077765921
2501	CPNE1	ENSG00000214078	20:35627413-35630438:-	6.99E-06	0.395331927	0.311128351
2502	CPNE1	ENSG00000214078	20:35632025-35632162:-	2.36E-05	0.217916061	0.191619049
2503	NFS1	ENSG00000244005	20:35674617-35675044:-	9.42E-05	0.093246638	0.130584518
2504	SCAND1	ENSG00000171222	20:35954339-35954447:-	0.0123896	0.610279439	0.656753766
2505	RPN2	ENSG00000118705	20:37228744-37229972:+	0.00145478	0.097250877	0.070683383
2506	TTI1	ENSG00000101407	20:37983639-37996374:-	0.00021865	0.171766188	0.126864508
2507	PLCG1	ENSG00000124181	20:41159758-41159869:+	6.75E-05	0.070275662	0.105951039
2508	PLCG1	ENSG00000124181	20:41165826-41166193:+	0.00443939	0.075228928	0.102419525

S/N	Gene	Ensembl ID	Position of retained intron	p-value	AD_IR_average	Cont_IR_average
2509	PLCG1	ENSG00000124181	20:41166859-41167851:+	0.02129307	0.508204732	0.459645974
2510	L3MBTL1	ENSG00000185513	20:43536294-43536408:+	0.01497874	0.145660792	0.108699091
2511	L3MBTL1	ENSG00000185513	20:43540308-43540752:+	0.01552255	0.136438783	0.111079713
2512	GDAP1L1	ENSG00000124194	20:44258607-44263229:+	6.30E-06	0.104146351	0.068288652
2513	TOMM34	ENSG00000025772	20:44943213-44943452:-	0.01106631	0.108989909	0.090578101
2514	PIGT	ENSG00000124155	20:45420693-45421382:+	0.02007292	0.093400048	0.072397794
2515	PIGT	ENSG00000124155	20:45421583-45424215:+	0.00010282	0.115308037	0.076664261
2516	DNTTIP1	ENSG00000101457	20:45802057-45803332:+	0.01405118	0.09816405	0.076927558
2517	ACOT8	ENSG00000101473	20:45843721-45844262:-	1.02E-05	0.405761596	0.30551394
2518	ACOT8	ENSG00000101473	20:45844420-45848449:-	4.34E-05	0.353346573	0.277592886
2519	ACOT8	ENSG00000101473	20:45848675-45855158:-	0.00097088	0.181065593	0.154521966
2520	CTSA	ENSG00000064601	20:45891762-45891915:+	1.09E-06	0.163286899	0.108144582
2521	CTSA	ENSG00000064601	20:45892027-45892272:+	4.49E-05	0.116665188	0.082850149
2522	CTSA	ENSG00000064601	20:45892323-45892397:+	1.14E-06	0.143208873	0.095704005
2523	CTSA	ENSG00000064601	20:45895133-45896964:+	0.04802525	0.324381712	0.295661532
2524	CTSA	ENSG00000064601	20:45897040-45897716:+	0.04177649	0.470004232	0.437187909
2525	PCIF1	ENSG00000100982	20:45939339-45940474:+	0.02144801	0.278999423	0.253878816
2526	ZNF335	ENSG00000198026	20:45949080-45949169:-	7.84E-07	0.136093765	0.094825625
2527	ZNF335	ENSG00000198026	20:45949251-45949332:-	2.07E-07	0.143197938	0.097051971
2528	ZNF335	ENSG00000198026	20:45949398-45949484:-	5.90E-08	0.160353802	0.106104309
2529	ZNF335	ENSG00000198026	20:45949568-45949799:-	6.51E-05	0.223729354	0.176546852
2530	ZNF335	ENSG00000198026	20:45949877-45949965:-	8.07E-08	0.139657313	0.093384473
2531	ZNF335	ENSG00000198026	20:45950069-45950218:-	4.97E-07	0.150318756	0.099350919
2532	ZNF335	ENSG00000198026	20:45950373-45950452:-	0.0002041	0.166536896	0.124156608
2533	ZNF335	ENSG00000198026	20:45950595-45952146:-	0.00015852	0.250082012	0.202391764
2534	ZNF335	ENSG00000198026	20:45952521-45952597:-	1.60E-05	0.178955395	0.130342816
2535	ZNF335	ENSG00000198026	20:45952709-45953688:-	4.53E-10	0.160992695	0.099588669
2536	CD40	ENSG00000101017	20:46122358-46122609:+	0.01167249	0.142674815	0.116365778
2537	SLC35C2	ENSG00000080189	20:46350524-46350760:-	0.01923467	0.125828328	0.157361558
2538	TMEM189	ENSG00000240849	20:50125179-50127974:-	0.00017753	0.275268715	0.221796627
2539	DPM1	ENSG00000000419	20:50945762-50945846:-	0.00994729	0.169962346	0.139533835
2540	RAE1	ENSG00000101146	20:57368812-57373474:+	0.04968276	0.110195544	0.093526765
2541	RAE1	ENSG00000101146	20:57373581-57373662:+	0.00391898	0.104519004	0.081595048
2542	RBM38	ENSG00000132819	20:57393333-57407542:+	0.00111349	0.168407483	0.138789777
2543	NPEPL1	ENSG00000215440	20:58693050-58693736:+	0.00019737	0.316020427	0.245620227
2544	NPEPL1	ENSG00000215440	20:58712579-58713419:+	0.0193755	0.18744539	0.156294048
2545	GNAS	ENSG00000087460	20:58898985-58899948:+	1.04E-08	0.019456404	0.012073827
2546	GNAS	ENSG00000087460	20:58898985-58903527:+	0.00072037	0.017470034	0.013260762
2547	ADRM1	ENSG00000130706	20:62306320-62306647:+	0.00045884	0.298115066	0.244010906
2548	ADRM1	ENSG00000130706	20:62306734-62307370:+	0.00676841	0.390236902	0.33727926
2549	ADRM1	ENSG00000130706	20:62307452-62307595:+	0.00185765	0.284577929	0.226325581
2550	ADRM1	ENSG00000130706	20:62308178-62308367:+	1.24E-06	0.246423889	0.179240903
2551	LAMA5	ENSG00000130702	20:62311094-62311161:-	0.018936	0.098920572	0.117247958
2552	LAMA5	ENSG00000130702	20:62312780-62312887:-	0.01015192	0.353832378	0.288747169
2553	LAMA5	ENSG00000130702	20:62313010-62313087:-	0.00015404	0.366115415	0.278697443
2554	LAMA5	ENSG00000130702	20:62314440-62314554:-	0.01349754	0.086777433	0.105167431
2555	LAMA5	ENSG00000130702	20:62314725-62314798:-	0.01247637	0.106152999	0.131465061
2556	LAMA5	ENSG00000130702	20:62315207-62315947:-	0.00171602	0.073133224	0.101684939
2557	LAMA5	ENSG00000130702	20:62317023-62317344:-	0.01067316	0.074581287	0.104733644
2558	LAMA5	ENSG00000130702	20:62317499-62317661:-	0.00169617	0.064067827	0.097820719
2559	LAMA5	ENSG00000130702	20:62319013-62319683:-	0.00250576	0.095937763	0.13584259
2560	SLCO4A1	ENSG00000101187	20:62668184-62668476:+	0.02253293	0.172928622	0.21999577
2561	TCFL5	ENSG00000101190	20:62842097-62854015:-	0.04282902	0.077299177	0.066401499
2562	GID8	ENSG00000101193	20:62943183-62943494:+	0.02192947	0.084718254	0.114321879
2563	ARFGAP1	ENSG00000101199	20:63276651-63277204:+	0.00082801	0.112866083	0.08507587
2564	ARFGAP1	ENSG00000101199	20:63277305-63278116:+	0.00034703	0.097625549	0.068508531
2565	KCNQ2	ENSG00000075043	20:63445364-63446746:-	0.00862241	0.073139172	0.098130464

S/N	Gene	Ensembl ID	Position of retained intron	p-value	AD_IR_average	Cont_IR_average
2566	RTEL1	ENSG00000258366	20:63678346-63679848:+	0.00551114	0.085150957	0.113619118
2567	RTEL1	ENSG00000258366	20:63688605-63689054:+	0.01172505	0.216324594	0.170878678
2568	RTEL1	ENSG00000258366	20:63690210-63690293:+	0.03198824	0.283089749	0.224821717
2569	ARFRP1	ENSG00000101246	20:63700530-63700601:-	0.00474913	0.312753651	0.247446221
2570	ZGPAT	ENSG00000197114	20:63708118-63708552:+	7.50E-05	0.103256761	0.076987314
2571	TPD52L2	ENSG00000101150	20:63873816-63882718:+	0.01518965	0.15992934	0.193020453
2572	UCKL1	ENSG00000198276	20:63945975-63946160:-	0.00970676	0.42603	0.48676074
2573	TCEA2	ENSG00000171703	20:64067020-64068046:+	4.22E-05	0.154714678	0.112667357
2574	TCEA2	ENSG00000171703	20:64069491-64069764:+	4.07E-06	0.148794489	0.094886047
2575	TCEA2	ENSG00000171703	20:64070414-64070488:+	9.50E-06	0.110955765	0.072665084
2576	MRPL39	ENSG00000154719	21:25585754-25588834:-	0.01404215	0.197750317	0.162891249
2577	SCAF4	ENSG00000156304	21:31692449-31693293:-	0.0037712	0.09202341	0.06584026
2578	TCP10L	ENSG00000242220	21:32576923-32578693:-	0.0257511	0.112795845	0.092203313
2579	PSMG1	ENSG00000183527	21:39175664-39177434:-	0.00300722	0.21241272	0.180432477
2580	HMGN1	ENSG00000205581	21:39345274-39345829:-	0.00032852	0.155700571	0.152630351
2581	PDXK	ENSG00000160209	21:43743807-43746078:+	0.00097039	0.308597805	0.252161271
2582	RRP1	ENSG00000160214	21:43792729-43793318:+	0.0005546	0.19866165	0.15634314
2583	RRP1	ENSG00000160214	21:43802387-43803511:+	0.01659388	0.278725707	0.248566208
2584	PFKL	ENSG00000141959	21:44313682-44313912:+	0.00105786	0.161463217	0.118972657
2585	PFKL	ENSG00000141959	21:44320147-44321728:+	0.04373983	0.160712634	0.139224242
2586	PFKL	ENSG00000141959	21:44324917-44325152:+	0.00095434	0.113767167	0.084552883
2587	PFKL	ENSG00000141959	21:44325264-44325960:+	0.00343686	0.128295044	0.101599951
2588	C21orf2	ENSG00000160226	21:44337667-44339117:-	0.01192128	0.14444128	0.112026278
2589	PCBP3	ENSG00000183570	21:45917629-45929916:+	0.01662513	0.091689582	0.072724442
2590	PCBP3	ENSG00000183570	21:45929995-45930785:+	0.01669959	0.091004529	0.07544779
2591	PCBP3	ENSG00000183570	21:45930845-45935252:+	0.01744216	0.113009609	0.093754122
2592	LSS	ENSG00000160285	21:46191959-46194490:-	0.00046767	0.581099451	0.493746805
2593	LSS	ENSG00000160285	21:46222738-46227551:-	0.00017446	0.082473939	0.124904497
2594	MCM3AP	ENSG00000160294	21:46270563-46272560:-	2.55E-05	0.087119026	0.156001775
2595	PCNT	ENSG00000160299	21:46430657-46431528:+	0.00121023	0.337393244	0.291891806
2596	PCNT	ENSG00000160299	21:46443948-46444693:+	0.01090974	0.46111189	0.496596156
2597	CECR5	ENSG00000069998	22:17143131-17145023:-	0.00638609	0.210603255	0.17530016
2598	DGCR6	ENSG00000183628	22:18910272-18910887:+	0.02066084	0.313478866	0.297342468
2599	PRODH	ENSG00000100033	22:18913362-18913437:-	0.02660944	0.331759549	0.284732396
2600	PRODH	ENSG00000100033	22:18925722-18930989:-	0.02631941	0.225404733	0.171984423
2601	SLC25A1	ENSG00000100075	22:19177204-19177726:-	0.00088495	0.31831328	0.259211039
2602	SLC25A1	ENSG00000100075	22:19178041-19178132:-	0.03945955	0.101458904	0.080552496
2603	UFD1L	ENSG00000070010	22:19450744-19454748:-	0.00270527	0.339622415	0.286971182
2604	ARVCF	ENSG00000099889	22:19975757-19977414:-	3.10E-06	0.041772388	0.046180248
2605	ARVCF	ENSG00000099889	22:19980242-19981210:-	0.00072815	0.253164	0.332119935
2606	DGCR8	ENSG00000128191	22:20090258-20091434:+	5.66E-05	0.489870402	0.405898874
2607	TRMT2A	ENSG00000099899	22:20113007-20113117:-	0.00036821	0.232207198	0.181630304
2608	TRMT2A	ENSG00000099899	22:20113234-20113431:-	0.00056519	0.313926598	0.245434583
2609	TRMT2A	ENSG00000099899	22:20113808-20114573:-	0.00114775	0.241458393	0.188828245
2610	TRMT2A	ENSG00000099899	22:20115079-20115265:-	0.00288439	0.314787305	0.382080701
2611	ZDHHC8	ENSG00000099904	22:20139892-20140114:+	0.00113593	0.605491512	0.53849326
2612	DGCR6L	ENSG00000128185	22:20315476-20316118:-	0.00536279	0.157618718	0.149456818
2613	DGCR6L	ENSG00000128185	22:20315476-20319638:-	9.66E-07	0.1409415	0.119467065
2614	DGCR6L	ENSG00000128185	22:20316219-20319638:-	7.85E-05	0.142377127	0.120287952
2615	MED15	ENSG00000099917	22:20585015-20585100:+	0.00013659	0.223908717	0.172422296
2616	AIFM3	ENSG00000183773	22:20973867-20974062:+	0.00314144	0.114112588	0.148705812
2617	AIFM3	ENSG00000183773	22:20978005-20979270:+	0.00697858	0.132649652	0.118239187
2618	AIFM3	ENSG00000183773	22:20979702-20980019:+	8.24E-06	0.166350511	0.124720771
2619	AIFM3	ENSG00000183773	22:20980124-20980991:+	0.01093997	0.176674	0.139921131
2620	LZTR1	ENSG00000099949	22:20995026-20995745:+	0.0024382	0.166790641	0.210889736
2621	SLC2A11	ENSG00000133460	22:23882646-23882758:+	0.01531407	0.179342666	0.21428887
2622	CABIN1	ENSG00000099991	22:24091843-24095930:+	0.02773645	0.225979855	0.190788569

S/N	Gene	Ensembl ID	Position of retained intron	p-value	AD_IR_average	Cont_IR_average
2623	SRRD	ENSG00000100104	22:26490198-26491024:+	0.00279931	0.095950478	0.162555438
2624	TFIP11	ENSG00000100109	22:26492368-26494138:-	0.04144303	0.337023268	0.363490532
2625	TFIP11	ENSG00000100109	22:26494939-26496072:-	0.00156535	0.120383479	0.150724683
2626	TFIP11	ENSG00000100109	22:26496889-26498868:-	0.00066877	0.133634713	0.182754832
2627	TPST2	ENSG00000128294	22:26526267-26528213:-	0.01083114	0.096539889	0.115816045
2628	HSCB	ENSG00000100209	22:28746008-28751240:+	0.0130824	0.067947444	0.055170401
2629	RHBDD3	ENSG00000100263	22:29260864-29263834:-	1.70E-07	0.19419877	0.138298405
2630	EWSR1	ENSG00000182944	22:29273864-29278029:+	0.00084193	0.168218074	0.117930512
2631	EWSR1	ENSG00000182944	22:29288786-29291561:+	0.00030989	0.316735538	0.244545297
2632	EWSR1	ENSG00000182944	22:29291599-29292136:+	0.00340291	0.282984052	0.245087571
2633	AP1B1	ENSG00000100280	22:29329720-29330377:-	0.00198341	0.052974838	0.039921935
2634	AP1B1	ENSG00000100280	22:29331916-29334264:-	0.01348119	0.106523822	0.089659027
2635	NF2	ENSG00000186575	22:29683064-29694751:+	0.0126415	0.08363403	0.066558587
2636	CABP7	ENSG00000100314	22:29728742-29729054:+	0.00011199	0.17677246	0.132773778
2637	RNF215	ENSG00000099999	22:30379622-30379710:-	0.00036294	0.453371598	0.36864913
2638	RNF215	ENSG00000099999	22:30385989-30386069:-	0.04977951	0.350425024	0.315833805
2639	SMTN	ENSG00000183963	22:31095609-31096732:+	0.0183748	0.41830739	0.450664922
2640	SMTN	ENSG00000183963	22:31099179-31099744:+	3.04E-06	0.211221187	0.128399845
2641	EIF4ENIF1	ENSG00000184708	22:31440121-31440703:-	0.00596562	0.226874117	0.190176696
2642	EIF4ENIF1	ENSG00000184708	22:31442118-31442961:-	0.03399316	0.196332939	0.166101866
2643	SFI1	ENSG00000198089	22:31611303-31611765:+	0.00199279	0.161557382	0.12098796
2644	SFI1	ENSG00000198089	22:31613216-31613353:+	0.00089958	0.249921893	0.186293716
2645	SFI1	ENSG00000198089	22:31613530-31613601:+	0.00040978	0.209747806	0.140849678
2646	SFI1	ENSG00000198089	22:31613855-31614788:+	6.16E-06	0.546814634	0.43277826
2647	SFI1	ENSG00000198089	22:31614860-31615047:+	5.15E-05	0.446711415	0.344007623
2648	RBFOX2	ENSG00000100320	22:35746561-35756104:-	0.00462346	0.100235	0.127809162
2649	MYH9	ENSG00000100345	22:36305102-36305929:-	1.83E-08	0.162713427	0.095665362
2650	MPST	ENSG00000128309	22:37024810-37029215:+	0.02078589	0.108972074	0.081780708
2651	KCTD17	ENSG00000100379	22:37061629-37062524:+	0.00496409	0.143046873	0.120515974
2652	GGA1	ENSG00000100083	22:37616997-37618447:+	0.04289513	0.351026159	0.329321532
2653	GGA1	ENSG00000100083	22:37618546-37620237:+	0.0042644	0.113155452	0.094089808
2654	GGA1	ENSG00000100083	22:37620361-37620812:+	1.61E-07	0.171388446	0.113791696
2655	NOL12	ENSG00000273899	22:37691424-37693331:+	4.32E-06	0.627068598	0.533537455
2656	PICK1	ENSG00000100151	22:38073823-38074306:+	9.53E-06	0.762181683	0.711360351
2657	PLA2G6	ENSG00000184381	22:38113654-38115526:-	0.00023261	0.171737756	0.123284364
2658	PLA2G6	ENSG00000184381	22:38115681-38116074:-	7.61E-05	0.203990271	0.152908151
2659	PLA2G6	ENSG00000184381	22:38120909-38123094:-	0.00014029	0.176971237	0.136463553
2660	CSNK1E	ENSG00000213923	22:38293319-38294108:-	0.00246924	0.26406928	0.224845266
2661	CBX7	ENSG00000100307	22:39134048-39134400:-	0.00319502	0.100133828	0.078460132
2662	SYNGR1	ENSG00000100321	22:39374553-39376051:+	0.01499071	0.378544449	0.309531818
2663	SGSM3	ENSG00000100359	22:40406662-40407016:+	5.58E-05	0.280682963	0.211133265
2664	SGSM3	ENSG00000100359	22:40407071-40407200:+	0.02932242	0.278126166	0.23734346
2665	SGSM3	ENSG00000100359	22:40407568-40407788:+	0.00013096	0.463591927	0.387767091
2666	SGSM3	ENSG00000100359	22:40407843-40408070:+	0.00019391	0.438717512	0.36612639
2667	SGSM3	ENSG00000100359	22:40408842-40408932:+	0.00893588	0.258822378	0.217030887
2668	L3MBTL2	ENSG00000100395	22:41224251-41224724:+	0.03689814	0.083896085	0.112097052
2669	L3MBTL2	ENSG00000100395	22:41224801-41224966:+	0.00167869	0.076614778	0.114477409
2670	L3MBTL2	ENSG00000100395	22:41225071-41225793:+	0.00541942	0.079101411	0.11161091
2671	L3MBTL2	ENSG00000100395	22:41229656-41230138:+	0.00114233	0.122337907	0.164848457
2672	ACO2	ENSG00000100412	22:41517631-41518480:+	1.20E-06	0.113713509	0.090933186
2673	SREBF2	ENSG00000198911	22:41900498-41902969:+	0.02240281	0.17401272	0.149638639
2674	RRP7A	ENSG00000189306	22:42516136-42518004:-	0.024577	0.112996593	0.096496909
2675	TTLL12	ENSG00000100304	22:43183149-43186892:-	0.04578972	0.155502827	0.13315471
2676	KIAA0930	ENSG00000100364	22:45212107-45212264:-	9.78E-05	0.648855451	0.577444247
2677	FAM118A	ENSG00000100376	22:45336411-45340385:+	0.03367312	0.12766367	0.113341427
2678	TTC38	ENSG00000075234	22:46289561-46289825:+	0.02742961	0.10483747	0.073788521
2679	TRMU	ENSG00000100416	22:46355588-46355989:+	0.02641851	0.46449511	0.439527584

S/N	Gene	Ensembl ID	Position of retained intron	p-value	AD_IR_average	Cont_IR_average
2680	TRMU	ENSG00000100416	22:46356072-46356841:+	0.02042835	0.453190598	0.421596325
2681	BRD1	ENSG00000100425	22:49777813-49787389:-	3.73E-07	0.149219185	0.101140653
2682	TRABD	ENSG00000170638	22:50195040-50197240:+	0.01107604	0.167132841	0.191633195
2683	TRABD	ENSG00000170638	22:50197351-50197448:+	0.00905231	0.212944362	0.247838384
2684	TUBGCP6	ENSG00000128159	22:50219456-50219643:-	8.41E-06	0.328019378	0.230576865
2685	TUBGCP6	ENSG00000128159	22:50219791-50219956:-	0.0004794	0.122607362	0.081779989
2686	HDAC10	ENSG00000100429	22:50246966-50247691:-	0.00145495	0.227357907	0.211781539
2687	HDAC10	ENSG00000100429	22:50248292-50248365:-	0.00219976	0.356138444	0.300862622
2688	HDAC10	ENSG00000100429	22:50248472-50248661:-	0.00049245	0.321960646	0.262564169
2689	HDAC10	ENSG00000100429	22:50248751-50248830:-	5.82E-05	0.35902128	0.282625
2690	HDAC10	ENSG00000100429	22:50250160-50250770:-	0.01100121	0.330802512	0.271500078
2691	MAPK12	ENSG00000188130	22:50253480-50255196:-	0.00099971	0.168252251	0.204365156
2692	MAPK12	ENSG00000188130	22:50255694-50255809:-	3.82E-06	0.143252068	0.094589218
2693	MAPK12	ENSG00000188130	22:50255881-50256084:-	5.38E-05	0.316148659	0.249493218
2694	MAPK12	ENSG00000188130	22:50256646-50256934:-	0.00434682	0.172797116	0.163096508
2695	MAPK11	ENSG00000185386	22:50266305-50266539:-	9.69E-06	0.286027207	0.214412052
2696	MAPK11	ENSG00000185386	22:50266611-50266933:-	5.56E-07	0.34840239	0.251595455
2697	MAPK11	ENSG00000185386	22:50267482-50267568:-	0.00052883	0.191821012	0.159222843
2698	PLXNB2	ENSG00000196576	22:50283990-50284131:-	0.00759184	0.169376677	0.145007575
2699	PLXNB2	ENSG00000196576	22:50284213-50284572:-	0.00043452	0.517735146	0.454189221
2700	PLXNB2	ENSG00000196576	22:50286098-50286172:-	0.00409992	0.077026676	0.102249783
2701	PLXNB2	ENSG00000196576	22:50290597-50294718:-	0.00564108	0.033996961	0.050954504
2702	DENND6B	ENSG00000205593	22:50313878-50314206:-	0.0007682	0.554424976	0.469400299
2703	DENND6B	ENSG00000205593	22:50314272-50314399:-	6.17E-07	0.248804439	0.150091674
2704	PPP6R2	ENSG00000100239	22:50439857-50439960:+	0.00062132	0.111497468	0.080075532
2705	PPP6R2	ENSG00000100239	22:50441026-50443865:+	0.03051873	0.465384537	0.414481117
2706	SBF1	ENSG00000100241	22:50460712-50461158:-	1.40E-05	0.169757406	0.102464024
2707	SBF1	ENSG00000100241	22:50466482-50466604:-	0.00921446	0.067134537	0.092880727
2708	SBF1	ENSG00000100241	22:50468461-50474785:-	0.00651183	0.071192827	0.094191287
2709	LMF2	ENSG00000100258	22:50505153-50505228:-	0.00667647	0.394050427	0.334565753
2710	LMF2	ENSG00000100258	22:50505334-50505402:-	0.00110678	0.38458311	0.326326247
2711	LMF2	ENSG00000100258	22:50505815-50506034:-	0.00117755	0.0993609	0.07346271
2712	LMF2	ENSG00000100258	22:50506213-50506284:-	0.01098867	0.168050272	0.1268973
2713	NCAPH2	ENSG00000025770	22:50521036-50521542:+	0.00305627	0.198685413	0.160698303
2714	CPT1B	ENSG00000205560	22:50569081-50569335:-	0.00505068	0.452019402	0.404850597
2715	CHKB	ENSG00000100288	22:50580271-50580357:-	0.00046483	0.382644659	0.435893455
2716	CHKB	ENSG00000100288	22:50581553-50581748:-	0.00092558	0.583808244	0.514392818
2717	CHKB	ENSG00000100288	22:50581862-50582248:-	0.00149303	0.533757634	0.593308325
2718	MAPK8IP2	ENSG00000008735	22:50606765-50606920:+	0.04295977	0.111681795	0.089481357
2719	ARSA	ENSG00000100299	22:50627406-50627555:-	0.00123083	0.107756448	0.147204236
2720	SHANK3	ENSG00000251322	22:50715753-50720183:+	0.01618366	0.067243828	0.083904824
2721	THUMPD3	ENSG00000134077	3:9380618-9383198:+	8.39E-05	0.518053963	0.457852481
2722	THUMPD3	ENSG00000134077	3:9383309-9384211:+	3.01E-10	0.436996134	0.31296599
2723	THUMPD3	ENSG00000134077	3:9384335-9384523:+	4.21E-09	0.428896378	0.319810377
2724	SETD5	ENSG00000168137	3:9434485-9434823:+	1.55E-05	0.071657512	0.119051927
2725	SETD5	ENSG00000168137	3:9435906-9436849:+	0.00042394	0.110431005	0.167329848
2726	SETD5	ENSG00000168137	3:9441741-9442127:+	0.02735422	0.117989116	0.1643967
2727	SETD5	ENSG00000168137	3:9443417-9445047:+	0.03606781	0.086383455	0.140432423
2728	MTMR14	ENSG00000163719	3:9684670-9684887:+	5.22E-06	0.135072866	0.094518355
2729	MTMR14	ENSG00000163719	3:9685247-9687820:+	1.38E-06	0.213065066	0.152800913
2730	MTMR14	ENSG00000163719	3:9687891-9688695:+	1.78E-05	0.197712065	0.145794061
2731	MTMR14	ENSG00000163719	3:9689082-9689963:+	1.74E-05	0.05638118	0.042930762
2732	MTMR14	ENSG00000163719	3:9689082-9701789:+	0.00757658	0.093519811	0.065354079
2733	MTMR14	ENSG00000163719	3:9690143-9697710:+	4.97E-07	0.150596932	0.101950644
2734	MTMR14	ENSG00000163719	3:9697866-9701789:+	2.34E-09	0.142348776	0.099987878
2735	OGG1	ENSG00000114026	3:9756621-9756766:+	0.00079633	0.173087977	0.124751464
2736	TADA3	ENSG00000171148	3:9789963-9791259:-	3.19E-08	0.09696247	0.167302205

S/N	Gene	Ensembl ID	Position of retained intron	p-value	AD_IR_average	Cont_IR_average
2737	ARPC4-TTL	ENSG00000250151	3:9820741-9825799:+	0.02665309	0.13259878	0.108771119
2738	ARPC4-TTL	ENSG00000250151	3:9825948-9826996:+	0.00516447	0.105346891	0.083084397
2739	ARPC4-TTL	ENSG00000250151	3:9829395-9834680:+	0.00822507	0.235465032	0.194010791
2740	RPUSD3	ENSG00000156990	3:9839171-9840183:-	0.03435159	0.333805232	0.28994283
2741	CRELD1	ENSG00000163703	3:9937672-9938014:+	0.03002653	0.103677555	0.091684218
2742	CRELD1	ENSG00000163703	3:9943515-9944364:+	0.00096582	0.405860756	0.348839104
2743	VGLL4	ENSG00000144560	3:11559455-11564796:-	0.02432949	0.277451259	0.239990117
2744	TAMM41	ENSG00000144559	3:11790581-11807832:-	1.61E-06	0.193589491	0.14100981
2745	RAF1	ENSG00000132155	3:12584657-12584846:-	0.00936363	0.099202696	0.083892043
2746	NUP210	ENSG00000132182	3:13319979-13321584:-	0.00488977	0.079183145	0.101420525
2747	TMEM43	ENSG00000170876	3:14132935-14133738:+	0.04923225	0.101926646	0.148297787
2748	XPC	ENSG00000154767	3:14148731-14148813:-	0.01525377	0.16263113	0.133594447
2749	XPC	ENSG00000154767	3:14148948-14152334:-	0.00244201	0.17569097	0.131901635
2750	MRPS25	ENSG00000131368	3:15052633-15053379:-	0.00468753	0.077142111	0.069340347
2751	COLQ	ENSG00000206561	3:15451713-15453828:-	0.00052634	0.091747582	0.054903094
2752	COLQ	ENSG00000206561	3:15456019-15456459:-	0.0295773	0.192829138	0.147038213
2753	HACL1	ENSG00000131373	3:15560897-15563357:-	4.04E-09	0.2280397	0.170546151
2754	MLH1	ENSG00000076242	3:37040294-37042267:+	2.13E-06	0.128692222	0.078572592
2755	PLCD1	ENSG00000187091	3:38008163-38008234:-	4.80E-05	0.184519822	0.123739832
2756	CTNNB1	ENSG00000168036	3:41239354-41239659:+	0.02332132	0.729207854	0.74541287
2757	LZTFL1	ENSG00000163818	3:45826332-45827355:-	2.73E-06	0.223678178	0.149916149
2758	PTH1R	ENSG00000160801	3:46901085-46901413:+	0.00217483	0.260904955	0.20430931
2759	PTH1R	ENSG00000160801	3:46901480-46901765:+	0.01756016	0.210975099	0.171982752
2760	PTH1R	ENSG00000160801	3:46901860-46902525:+	0.0157613	0.159850359	0.126362377
2761	NBEAL2	ENSG00000160796	3:47005847-47005945:+	0.0453773	0.1028539	0.126628444
2762	NBEAL2	ENSG00000160796	3:47006262-47006332:+	0.00226919	0.140553337	0.099447869
2763	NBEAL2	ENSG00000160796	3:47006449-47007065:+	0.04503168	0.492679366	0.439885117
2764	PTPN23	ENSG00000076201	3:47405081-47405748:+	0.04844963	0.12391366	0.14827499
2765	SCAP	ENSG00000114650	3:47414993-47415097:-	6.78E-05	0.213888824	0.168732348
2766	SCAP	ENSG00000114650	3:47415180-47417121:-	0.00164775	0.247734	0.215278581
2767	SCAP	ENSG00000114650	3:47418249-47418320:-	0.0035727	0.177348562	0.122961456
2768	MAP4	ENSG00000047849	3:47853039-47853162:-	0.0003189	0.077809935	0.055489509
2769	PLXNB1	ENSG00000164050	3:48412009-48412237:-	0.00298445	0.174079511	0.138965995
2770	PLXNB1	ENSG00000164050	3:48415759-48416030:-	0.01147705	0.111236517	0.080144381
2771	PLXNB1	ENSG00000164050	3:48422947-48423504:-	0.02927557	0.081979631	0.106624838
2772	ATRIP	ENSG00000164053	3:48465083-48465486:+	0.02759417	0.267327116	0.241382939
2773	COL7A1	ENSG00000114270	3:48572421-48572502:-	0.00259642	0.128913614	0.080849914
2774	COL7A1	ENSG00000114270	3:48572538-48572670:-	0.00033396	0.169338101	0.109516751
2775	COL7A1	ENSG00000114270	3:48573557-48573689:-	0.00449315	0.095634701	0.063025235
2776	COL7A1	ENSG00000114270	3:48574550-48574676:-	0.01361944	0.110945515	0.076410773
2777	COL7A1	ENSG00000114270	3:48574721-48574796:-	0.00689718	0.127737345	0.083987252
2778	CELSR3	ENSG00000008300	3:48645868-48646089:-	0.00487615	0.382662585	0.306662878
2779	NCKIPSD	ENSG00000213672	3:48679457-48679574:-	0.00820817	0.134255389	0.116212673
2780	DALRD3	ENSG00000178149	3:49016086-49016157:-	0.0109137	0.615600317	0.573767714
2781	IMPDH2	ENSG00000178035	3:49024404-49024494:-	8.47E-08	0.248972309	0.183219756
2782	IMPDH2	ENSG00000178035	3:49024578-49024658:-	3.39E-09	0.177871568	0.124978377
2783	IMPDH2	ENSG00000178035	3:49024802-49024895:-	6.31E-08	0.15931371	0.110914229
2784	IMPDH2	ENSG00000178035	3:49025040-49025125:-	2.43E-07	0.136410876	0.090373062
2785	IMPDH2	ENSG00000178035	3:49025269-49026323:-	0.00029665	0.19176563	0.147108188
2786	IMPDH2	ENSG00000178035	3:49026419-49026518:-	1.09E-05	0.178435744	0.123877884
2787	IMPDH2	ENSG00000178035	3:49026609-49026686:-	4.47E-05	0.201407487	0.142450512
2788	IMPDH2	ENSG00000178035	3:49026886-49026959:-	0.00016389	0.295295045	0.228654595
2789	IMPDH2	ENSG00000178035	3:49027047-49027709:-	0.00266032	0.228217766	0.185941213
2790	IMPDH2	ENSG00000178035	3:49028532-49028757:-	0.01099625	0.403955927	0.368131575
2791	IMPDH2	ENSG00000178035	3:49028806-49029252:-	0.01493656	0.365318366	0.33941324
2792	QARS	ENSG00000172053	3:49098117-49098191:-	0.01366166	0.125840311	0.102908679
2793	QARS	ENSG00000172053	3:49099253-49099343:-	1.79E-09	0.202935709	0.104537135

S/N	Gene	Ensembl ID	Position of retained intron	p-value	AD_IR_average	Cont_IR_average
2794	QARS	ENSG00000172053	3:49099431-49099509:-	2.09E-10	0.179176576	0.088072309
2795	QARS	ENSG00000172053	3:49099647-49099760:-	4.78E-06	0.263111123	0.179427216
2796	LAMB2	ENSG00000172037	3:49123373-49123446:-	0.00323979	0.119297401	0.086705036
2797	C3orf62	ENSG00000188315	3:49271445-49274048:-	0.01150786	0.109864183	0.0907412
2798	C3orf62	ENSG00000188315	3:49274140-49276426:-	0.03271179	0.107292924	0.092409034
2799	APEH	ENSG00000164062	3:49683146-49683236:+	0.012746	0.095579994	0.079317918
2800	MST1	ENSG00000173531	3:49684453-49684549:-	0.00014385	0.303232904	0.227235439
2801	MST1	ENSG00000173531	3:49684656-49684737:-	6.12E-05	0.301511373	0.214666264
2802	MST1	ENSG00000173531	3:49685732-49685859:-	0.00136045	0.432688966	0.333582205
2803	RNF123	ENSG00000164068	3:49697939-49698051:+	0.0305034	0.101940311	0.123398201
2804	RNF123	ENSG00000164068	3:49713587-49713737:+	0.00016489	0.202075284	0.142068214
2805	RNF123	ENSG00000164068	3:49713825-49713909:+	0.00022317	0.243154305	0.178146617
2806	MON1A	ENSG00000164077	3:49909154-49909252:-	0.00111585	0.10507184	0.13996791
2807	RBM5	ENSG00000003756	3:50109688-50110378:+	0.00059493	0.092753062	0.079014627
2808	RBM5	ENSG00000003756	3:50110463-50110678:+	0.00237659	0.135086807	0.115768249
2809	RBM5	ENSG00000003756	3:50110770-50113382:+	8.20E-05	0.218214246	0.164421717
2810	RBM5	ENSG00000003756	3:50117171-50117249:+	0.00010915	0.199566789	0.146351568
2811	IFRD2	ENSG00000214706	3:50290294-50290387:-	0.03809235	0.186693112	0.212530134
2812	NPRL2	ENSG00000114388	3:50347901-50348123:-	0.04090975	0.44958128	0.41625387
2813	NPRL2	ENSG00000114388	3:50348410-50348526:-	0.01421702	0.330266878	0.292861405
2814	NPRL2	ENSG00000114388	3:50348563-50348684:-	0.03031125	0.209342829	0.185521173
2815	NPRL2	ENSG00000114388	3:50349010-50349385:-	0.00021893	0.303063171	0.250104065
2816	NPRL2	ENSG00000114388	3:50349494-50349664:-	7.07E-05	0.479427585	0.408958273
2817	NPRL2	ENSG00000114388	3:50349833-50349930:-	5.32E-05	0.297888756	0.23776749
2818	CACNA2D2	ENSG00000007402	3:50366338-50366577:-	0.04056258	0.243324446	0.234353653
2819	PCBP4	ENSG00000090097	3:51961304-51962809:-	0.02930475	0.117664639	0.098082588
2820	PCBP4	ENSG00000090097	3:51962088-51962809:-	0.04153838	0.152640144	0.128613734
2821	PCBP4	ENSG00000090097	3:51962892-51967325:-	5.81E-05	0.676355463	0.589570688
2822	ABHD14A-	ENSG00000114786	3:51985946-51986254:+	0.01150765	0.215624116	0.172932486
2823	ABHD14A-	ENSG00000114786	3:51986504-51986604:+	0.00092445	0.384781768	0.312663597
2824	ABHD14A-	ENSG00000114786	3:51987453-51987555:+	6.34E-08	0.292275702	0.18765167
2825	ABHD14A-	ENSG00000114786	3:51987624-51988523:+	0.0007119	0.345938957	0.273197883
2826	ABHD14A-	ENSG00000114786	3:51988603-51988765:+	0.00086663	0.18989889	0.142848403
2827	TWF2	ENSG00000247596	3:52229201-52229660:-	6.93E-05	0.159511473	0.113819161
2828	PPM1M	ENSG00000164088	3:52249063-52249173:+	0.04281646	0.167785246	0.134602575
2829	NT5DC2	ENSG00000168268	3:52527375-52527616:-	0.01709261	0.136664728	0.106295014
2830	NT5DC2	ENSG00000168268	3:52527718-52527828:-	0.02263901	0.121035884	0.095098942
2831	NT5DC2	ENSG00000168268	3:52527931-52528012:-	0.04841021	0.096283395	0.077676825
2832	NT5DC2	ENSG00000168268	3:52529334-52533505:-	0.04852635	0.171397777	0.156336647
2833	CCDC66	ENSG00000180376	3:56619527-56619776:+	0.00645369	0.106161204	0.086836295
2834	PXK	ENSG00000168297	3:58410159-58412900:+	0.00849739	0.099010301	0.079784864
2835	PSMD6	ENSG00000163636	3:64022523-64023274:-	9.34E-06	0.194523404	0.1432579
2836	CPOX	ENSG00000080819	3:98588854-98590631:-	0.0002195	0.133737766	0.189337742
2837	TOMM70	ENSG00000154174	3:100365717-100368043:-	0.0475016	0.08462309	0.110908378
2838	CD47	ENSG00000196776	3:108047292-108051938:-	8.65E-06	0.100869205	0.078824023
2839	CD47	ENSG00000196776	3:108050602-108051938:-	0.00030327	0.238375137	0.16013666
2840	IFT57	ENSG00000114446	3:108162655-108163662:-	0.02228599	0.193919376	0.16314336
2841	TMEM39A	ENSG00000176142	3:119432214-119434761:-	0.00049867	0.33700232	0.251188168
2842	CCDC58	ENSG00000160124	3:122363027-122368175:-	0.02146189	0.063535706	0.065638501
2843	KLF15	ENSG00000163884	3:126343895-126351840:-	0.02486413	0.208373101	0.163857353
2844	TPRA1	ENSG00000163870	3:127573788-127575184:-	9.76E-06	0.231465463	0.168959913
2845	TPRA1	ENSG00000163870	3:127576050-127576616:-	0.00016622	0.379291232	0.316976883
2846	TPRA1	ENSG00000163870	3:127576696-127576820:-	0.00104028	0.244277146	0.200265799
2847	PODXL2	ENSG00000114631	3:127669202-127671433:+	0.02100757	0.136035252	0.1145055
2848	ABTB1	ENSG00000114626	3:127677563-127677675:+	0.01060058	0.22362526	0.264211286
2849	ACAD9	ENSG00000177646	3:128909421-128910020:+	0.00329584	0.246196293	0.191566408
2850	IFT122	ENSG00000163913	3:129519732-129520175:+	0.03414294	0.132304805	0.103540449

S/N	Gene	Ensembl ID	Position of retained intron	p-value	AD_IR_average	Cont_IR_average
2851	TF	ENSG00000091513	3:133777238-133778585:+	0.04491647	0.133989498	0.120323912
2852	CEP70	ENSG00000114107	3:138495076-138498030:-	2.17E-06	0.508817488	0.415913364
2853	CEP70	ENSG00000114107	3:138498110-138500109:-	7.41E-08	0.304974039	0.216709994
2854	MRPS22	ENSG00000175110	3:139351060-139352646:+	5.96E-05	0.169117824	0.129948844
2855	MRPS22	ENSG00000175110	3:139352792-139355681:+	8.13E-05	0.524254244	0.435432247
2856	MRPS22	ENSG00000175110	3:139355790-139356918:+	1.92E-06	0.555618866	0.408499588
2857	ZIC1	ENSG00000152977	3:147412681-147413353:+	0.0009021	0.107790302	0.082674368
2858	PFN2	ENSG00000070087	3:149966264-149968357:-	0.01181517	0.035741214	0.034439956
2859	CCNL1	ENSG00000163660	3:157148589-157149286:-	0.03707203	0.104839417	0.085231152
2860	ACTL6A	ENSG00000136518	3:179580701-179580893:+	0.00082122	0.150357496	0.10379149
2861	ACTL6A	ENSG00000136518	3:179581008-179581139:+	1.01E-06	0.185597723	0.11105079
2862	ACTL6A	ENSG00000136518	3:179581220-179583352:+	0.00020236	0.183663838	0.138312365
2863	ACTL6A	ENSG00000136518	3:179583448-179586545:+	0.01419695	0.257313743	0.219573087
2864	ACTL6A	ENSG00000136518	3:179586632-179587929:+	1.69E-06	0.216012116	0.153203621
2865	YEATS2	ENSG00000163872	3:183804188-183806865:+	0.0147277	0.153009707	0.210209044
2866	YEATS2	ENSG00000163872	3:183807092-183808029:+	0.00116768	0.216384126	0.304219336
2867	ABCC5	ENSG00000114770	3:183942916-183945849:-	0.00040914	0.107466741	0.072893378
2868	ABCC5	ENSG00000114770	3:183987917-183988571:-	1.54E-05	0.08246914	0.143311839
2869	EIF2B5	ENSG00000145191	3:184140157-184140417:+	0.0375534	0.147688271	0.193145009
2870	DVL3	ENSG00000161202	3:184164931-184165112:+	0.04675285	0.316732963	0.356138987
2871	DVL3	ENSG00000161202	3:184165206-184165421:+	0.01864148	0.219503017	0.192446086
2872	DVL3	ENSG00000161202	3:184166522-184166605:+	0.00721247	0.134263428	0.110391652
2873	ABCF3	ENSG00000161204	3:184186280-184186506:+	0.0109019	0.11563477	0.097288244
2874	ABCF3	ENSG00000161204	3:184192689-184192804:+	1.32E-05	0.073861893	0.102948626
2875	ALG3	ENSG00000214160	3:184242676-184242812:-	0.00023485	0.1017612	0.069770653
2876	ALG3	ENSG00000214160	3:184242957-184243553:-	0.01265428	0.123970106	0.098162531
2877	ALG3	ENSG00000214160	3:184243996-184244600:-	0.03589333	0.089499254	0.071645108
2878	ECE2	ENSG00000145194	3:184290667-184290792:+	0.03831162	0.178223157	0.168178588
2879	CLCN2	ENSG00000114859	3:184347021-184352012:-	0.00225676	0.275287134	0.227335127
2880	CLCN2	ENSG00000114859	3:184352117-184352292:-	0.00952532	0.193784872	0.154731409
2881	CLCN2	ENSG00000114859	3:184352331-184352442:-	0.04562226	0.15526016	0.126300613
2882	CLCN2	ENSG00000114859	3:184354314-184354547:-	0.03130488	0.091510991	0.067733715
2883	CHRD	ENSG00000090539	3:184382530-184382633:+	0.01183731	0.105672243	0.144735473
2884	CHRD	ENSG00000090539	3:184386755-184386844:+	0.02120792	0.112424582	0.099718656
2885	TNK2	ENSG00000061938	3:195868709-195870113:-	0.00210768	0.187607761	0.208089401
2886	TCTEX1D2	ENSG00000213123	3:196291374-196296005:-	0.00069587	0.119789722	0.089129
2887	TCTEX1D2	ENSG00000213123	3:196296069-196306942:-	0.04028505	0.137816534	0.117927057
2888	BDH1	ENSG00000161267	3:197522781-197532411:-	0.0077271	0.07412813	0.099485574
2889	BDH1	ENSG00000161267	3:197533561-197546360:-	0.01179219	0.182506316	0.223273423
2890	PIGG	ENSG00000174227	4:516185-521055:+	0.00052148	0.071551228	0.116717338
2891	PIGG	ENSG00000174227	4:527230-530435:+	0.00415701	0.183601837	0.215394221
2892	PIGG	ENSG00000174227	4:530745-533817:+	0.03487811	0.291445201	0.313377338
2893	PCGF3	ENSG00000185619	4:731110-733671:+	0.00010387	0.064135557	0.105343235
2894	PCGF3	ENSG00000185619	4:744688-761278:+	5.13E-06	0.080266212	0.133677192
2895	PCGF3	ENSG00000185619	4:761416-764983:+	0.00059721	0.1202491	0.175415071
2896	PCGF3	ENSG00000185619	4:765064-766031:+	0.00041641	0.079407781	0.124198388
2897	GAK	ENSG00000178950	4:868685-870710:-	3.46E-05	0.137337074	0.094517311
2898	TMEM175	ENSG00000127419	4:951258-951645:+	0.00094065	0.044405372	0.032186016
2899	TMEM175	ENSG00000127419	4:951717-952366:+	0.01265878	0.11871189	0.093236061
2900	TMEM175	ENSG00000127419	4:952450-953189:+	0.00360582	0.101607085	0.075877418
2901	DGKQ	ENSG00000145214	4:962082-962434:-	0.00296862	0.23662272	0.178921034
2902	IDUA	ENSG00000127415	4:1003625-1004011:+	9.78E-05	0.169743532	0.105067006
2903	SLBP	ENSG00000163950	4:1694840-1696201:-	0.00499028	0.096698912	0.079463113
2904	TMEM129	ENSG00000168936	4:1717428-1717515:-	0.00755999	0.353655341	0.293522078
2905	TMEM129	ENSG00000168936	4:1717675-1718151:-	0.00799532	0.476880976	0.41893126
2906	MXD4	ENSG00000123933	4:2255294-2257981:-	0.04383175	0.297807783	0.294921819
2907	ZFYVE28	ENSG00000159733	4:2270856-2271310:-	0.00225625	0.511644073	0.44835213

S/N	Gene	Ensembl ID	Position of retained intron	p-value	AD_IR_average	Cont_IR_average
2908	SH3BP2	ENSG00000087266	4:2825196-2827229:+	0.0067381	0.122236765	0.088963112
2909	ADD1	ENSG00000087274	4:2905015-2907742:+	0.02741207	0.111606011	0.089187917
2910	MFSD10	ENSG00000109736	4:2930953-2931060:-	0.01429578	0.408228768	0.340516234
2911	MFSD10	ENSG00000109736	4:2931150-2931226:-	0.02904606	0.21380819	0.170377842
2912	MFSD10	ENSG00000109736	4:2931465-2931554:-	0.00098254	0.29529722	0.241231091
2913	MFSD10	ENSG00000109736	4:2931663-2931824:-	0.02706089	0.421551061	0.383718
2914	MFSD10	ENSG00000109736	4:2931936-2932044:-	6.39E-07	0.246164122	0.173539978
2915	NOP14	ENSG00000087269	4:2945229-2946411:-	0.00146185	0.09142239	0.130135217
2916	NOP14	ENSG00000087269	4:2953645-2954423:-	5.46E-06	0.070189101	0.114149704
2917	GRK4	ENSG00000125388	4:3028001-3029200:+	0.00138054	0.202319695	0.160287532
2918	RGS12	ENSG00000159788	4:3417541-3420641:+	0.01003407	0.453867939	0.399577364
2919	RGS12	ENSG00000159788	4:3420718-3422375:+	0.03293104	0.230827626	0.196456557
2920	LYAR	ENSG00000145220	4:4268615-4273582:-	0.04517141	0.115020446	0.091003203
2921	STX18	ENSG00000168818	4:4420129-4420863:-	0.00019024	0.1526591	0.112152583
2922	CRMP1	ENSG00000072832	4:5836906-5839521:-	4.44E-05	0.112488007	0.079804116
2923	WFS1	ENSG00000109501	4:6295189-6300656:+	0.00031813	0.298194468	0.23105249
2924	BOD1L1	ENSG00000038219	4:13581054-13581131:-	0.00057959	0.123903378	0.075026691
2925	CC2D2A	ENSG00000048342	4:15596207-15597406:+	0.01640613	0.166205295	0.127447707
2926	CC2D2A	ENSG00000048342	4:15599706-15601236:+	2.39E-05	0.244062568	0.169785745
2927	ANAPC4	ENSG00000053900	4:25394905-25396663:+	0.0009846	0.252884385	0.1982073
2928	ANAPC4	ENSG00000053900	4:25396899-25402970:+	0.01131939	0.097325115	0.075456578
2929	WDR19	ENSG00000157796	4:39273061-39274807:+	0.00264187	0.580634573	0.562649805
2930	WDR19	ENSG00000157796	4:39274958-39277019:+	3.66E-06	0.166380952	0.134041178
2931	GUF1	ENSG00000151806	4:44695734-44697407:+	0.00148997	0.269499329	0.203084656
2932	NFXL1	ENSG00000170448	4:47851148-47851855:-	0.00839696	0.095392615	0.067384908
2933	OCIAD1	ENSG00000109180	4:48851975-48857212:+	4.30E-05	0.086374477	0.056686157
2934	OCIAD1	ENSG00000109180	4:48851975-48860724:+	0.00183903	0.06458046	0.041387888
2935	OCIAD1	ENSG00000109180	4:48857365-48860724:+	8.29E-07	0.090005438	0.063292473
2936	FIP1L1	ENSG00000145216	4:53458790-53459301:+	0.00109417	0.09060476	0.108280368
2937	TMEM165	ENSG00000134851	4:55417247-55417802:+	0.00147684	0.083065188	0.106146144
2938	TMEM165	ENSG00000134851	4:55417985-55424537:+	0.03482877	0.070926214	0.072442221
2939	EXOC1	ENSG00000090989	4:55891422-55892634:+	0.00251262	0.116032745	0.078732562
2940	AASDH	ENSG00000157426	4:56343684-56345126:-	0.00030312	0.298775548	0.240064144
2941	YTHDC1	ENSG00000083896	4:68318862-68320122:-	0.02007206	0.219694946	0.189554665
2942	RUFY3	ENSG00000018189	4:70800205-70802955:+	3.32E-05	0.342486952	0.267168305
2943	RUFY3	ENSG00000018189	4:70802983-70804347:+	2.88E-05	0.225701038	0.176611743
2944	RUFY3	ENSG00000018189	4:70804416-70806515:+	3.73E-07	0.199824045	0.143874001
2945	RCHY1	ENSG00000163743	4:75490701-75491610:-	0.00827428	0.120454701	0.091845055
2946	RCHY1	ENSG00000163743	4:75491637-75491723:-	0.00019156	0.1046265	0.069575446
2947	ART3	ENSG00000156219	4:76100320-76100794:+	0.00679612	0.250484	0.180439582
2948	MANBA	ENSG00000109323	4:102632281-102634787:-	0.00918577	0.1413702	0.112836044
2949	HADH	ENSG00000138796	4:108027760-108033175:+	0.01039136	0.116023676	0.085526019
2950	OSTC	ENSG00000198856	4:108650794-108655563:+	9.78E-10	0.128342338	0.06883941
2951	LARP7	ENSG00000174720	4:112646955-112647033:+	0.00079886	0.069216754	0.114177587
2952	LARP7	ENSG00000174720	4:112654159-112657246:+	5.88E-07	0.108915128	0.072506906
2953	RP11-33B1	ENSG00000245958	4:119550443-119551887:+	2.19E-05	0.684759683	0.592932506
2954	EXOSC9	ENSG00000123737	4:121816447-121816771:+	0.00739169	0.351752046	0.285466299
2955	CLGN	ENSG00000153132	4:140389304-140390627:-	0.00019903	0.123850019	0.071284583
2956	PLRG1	ENSG00000171566	4:154536744-154537285:-	0.0029085	0.113495882	0.089750782
2957	ETFDH	ENSG00000171503	4:158690425-158695496:+	0.00163085	0.106744341	0.079475662
2958	ETFDH	ENSG00000171503	4:158697699-158698986:+	0.00183844	0.179612843	0.124649331
2959	ETFDH	ENSG00000171503	4:158699130-158703422:+	0.005459	0.103802584	0.078348331
2960	PPID	ENSG00000171497	4:158710670-158710761:-	5.37E-07	0.10558364	0.168855217
2961	LRP2BP	ENSG00000109771	4:185367245-185370639:-	9.73E-17	0.365773537	0.212754536
2962	LRP2BP	ENSG00000109771	4:185370814-185372855:-	1.05E-09	0.446755195	0.333917195
2963	LRP2BP	ENSG00000109771	4:185373079-185374134:-	1.96E-18	0.195215156	0.096859196
2964	LRP2BP	ENSG00000109771	4:185374240-185374318:-	2.45E-11	0.29717369	0.194911356

S/N	Gene	Ensembl ID	Position of retained intron	p-value	AD_IR_average	Cont_IR_average
2965	LRP2BP	ENSG00000109771	4:185374461-185375612:-	0.00379337	0.293285455	0.224968238
2966	LRP2BP	ENSG00000109771	4:185375726-185376908:-	2.81E-13	0.28991444	0.173954064
2967	ANKRD37	ENSG00000186352	4:185396950-185397149:+	0.04605942	0.148959024	0.127678864
2968	UFSP2	ENSG00000109775	4:185400478-185403493:-	0.0137283	0.098793879	0.079744361
2969	PDCD6	ENSG00000249915	5:306754-311292:+	0.0498454	0.043030071	0.036701598
2970	NSUN2	ENSG00000037474	5:6600232-6602460:-	7.21E-05	0.180824783	0.135906245
2971	NSUN2	ENSG00000037474	5:6604685-6605272:-	0.0004335	0.389360622	0.320576201
2972	NSUN2	ENSG00000037474	5:6606912-6607199:-	0.04321595	0.401598049	0.358023058
2973	TRIO	ENSG00000038382	5:14462925-14465544:+	0.00159495	0.090117994	0.122538832
2974	ANKH	ENSG00000154122	5:14711310-14712873:-	0.00855169	0.189630143	0.160996703
2975	ANKH	ENSG00000154122	5:14713667-14716705:-	0.00166937	0.092619782	0.136389478
2976	CDH10	ENSG00000040731	5:24488153-24491575:-	0.01371876	0.20526359	0.151783406
2977	CDH10	ENSG00000040731	5:24491827-24492816:-	3.23E-07	0.097217626	0.048502641
2978	CDH10	ENSG00000040731	5:24492925-24498397:-	0.01037698	0.102677595	0.075630581
2979	CDH10	ENSG00000040731	5:24505248-24509565:-	0.02205281	0.088552468	0.087715539
2980	WDR70	ENSG00000082068	5:37721215-37722854:+	1.07E-06	0.127611785	0.078063319
2981	WDR70	ENSG00000082068	5:37722934-37724933:+	9.94E-06	0.130743437	0.086764844
2982	DHX29	ENSG00000067248	5:55269637-55270411:-	0.01651344	0.140134077	0.109299329
2983	DHX29	ENSG00000067248	5:55270487-55270577:-	0.01645948	0.096603732	0.07022828
2984	SKIV2L2	ENSG00000039123	5:55422982-55424719:+	4.36E-12	0.151606278	0.098508838
2985	GPBP1	ENSG00000062194	5:57236032-57246299:+	0.00305261	0.061869127	0.07474477
2986	DIMT1	ENSG00000086189	5:62389052-62390875:-	0.00744086	0.71910522	0.669374779
2987	DIMT1	ENSG00000086189	5:62392234-62392925:-	0.00301191	0.089504298	0.058473864
2988	PPWD1	ENSG00000113593	5:65579613-65583037:+	0.0120654	0.235423427	0.196672926
2989	PPWD1	ENSG00000113593	5:65583219-65585013:+	0.00320054	0.135346168	0.107238687
2990	PPWD1	ENSG00000113593	5:65585095-65585998:+	0.00025198	0.150198782	0.113644091
2991	PPWD1	ENSG00000113593	5:65586181-65587252:+	1.72E-05	0.304585183	0.232459281
2992	CCNB1	ENSG00000134057	5:69175537-69177238:+	0.00672829	0.163292461	0.121041729
2993	CCNB1	ENSG00000134057	5:69177349-69177523:+	0.04138031	0.26836591	0.229914386
2994	ANKRA2	ENSG00000164331	5:73552852-73553405:-	2.72E-05	0.409875524	0.293518688
2995	ANKRA2	ENSG00000164331	5:73553486-73554321:-	1.81E-07	0.300612759	0.17926784
2996	GFM2	ENSG00000164347	5:74721783-74722378:-	0.003639	0.148401412	0.120366906
2997	GFM2	ENSG00000164347	5:74722561-74725639:-	0.00131943	0.182236366	0.134269647
2998	POC5	ENSG00000152359	5:75674578-75677773:-	0.00067215	0.165790288	0.124201717
2999	ZCCHC9	ENSG00000131732	5:81308711-81308945:+	3.79E-05	0.091107354	0.126985075
3000	SSBP2	ENSG00000145687	5:81442723-81446867:-	1.69E-08	0.15903894	0.079877096
3001	SSBP2	ENSG00000145687	5:81446922-81448789:-	1.42E-07	0.113641167	0.054652514
3002	CCNH	ENSG00000134480	5:87394484-87395043:-	2.41E-05	0.102127789	0.076711834
3003	CETN3	ENSG00000153140	5:90394107-90399357:-	0.01545152	0.092006896	0.066770458
3004	CHD1	ENSG00000153922	5:98860068-98863407:-	0.00554521	0.151145761	0.108398808
3005	YTHDC2	ENSG00000047188	5:113593390-113593481:+	0.00034664	0.640112976	0.52882774
3006	FEM1C	ENSG00000145780	5:115543683-115544522:-	0.02338443	0.105473982	0.148707555
3007	ZNF608	ENSG00000168916	5:124639214-124641251:-	0.00056356	0.178927171	0.128330626
3008	GRAMD3	ENSG00000155324	5:126488892-126492914:+	0.02833082	0.118848099	0.134829983
3009	LMNB1	ENSG00000113368	5:126826107-126832693:+	0.003653	0.120865344	0.090355877
3010	P4HA2	ENSG00000072682	5:132198380-132198878:-	4.71E-06	0.387793829	0.260008977
3011	RAD50	ENSG00000113522	5:132609209-132609282:+	0.0047812	0.121516082	0.094207918
3012	Sep-08	ENSG00000164402	5:132758556-132760801:-	0.00222876	0.14455831	0.133658487
3013	SHROOM1	ENSG00000164403	5:132823522-132823622:-	0.00111659	0.084322168	0.109728597
3014	LEAP2	ENSG00000164406	5:132874089-132874409:+	0.03271313	0.098606541	0.077976326
3015	H2AFY	ENSG00000113648	5:135346057-135350822:-	0.00300275	0.056953993	0.052528684
3016	CTNNA1	ENSG00000044115	5:138930935-138932577:+	0.00745731	0.133444734	0.111326865
3017	NRG2	ENSG00000158458	5:139848697-139851603:-	0.00116568	0.200379326	0.16737413
3018	ANKHD1-E	ENSG00000254996	5:140549076-140549233:+	2.28E-06	0.160270854	0.118650306
3019	APBB3	ENSG00000113108	5:140558821-140560312:-	2.14E-05	0.331618476	0.266472858
3020	APBB3	ENSG00000113108	5:140560504-140560638:-	0.00027827	0.21722772	0.170864377
3021	APBB3	ENSG00000113108	5:140560754-140561017:-	0.00073438	0.25885755	0.210943088

S/N	Gene	Ensembl ID	Position of retained intron	p-value	AD_IR_average	Cont_IR_average
3022	APBB3	ENSG00000113108	5:140561701-140561843:-	0.00201393	0.330329563	0.405321844
3023	AC011380.	ENSG00000283155	5:140562723-140563593:-	0.01953191	0.652731695	0.608559052
3024	APBB3	ENSG00000113108	5:140563915-140564196:-	2.29E-07	0.699806476	0.574389299
3025	TMCO6	ENSG00000113119	5:140643041-140643563:+	0.00192132	0.241431343	0.190802318
3026	TMCO6	ENSG00000113119	5:140643675-140643779:+	0.0043332	0.228414717	0.182859034
3027	TMCO6	ENSG00000113119	5:140643966-140644099:+	0.01028209	0.298583723	0.250934443
3028	TMCO6	ENSG00000113119	5:140644194-140644572:+	0.0001062	0.340503561	0.265162094
3029	TMCO6	ENSG00000113119	5:140644740-140644984:+	0.00149985	0.3600995	0.30083441
3030	WDR55	ENSG00000120314	5:140668502-140668611:+	0.01498065	0.071860499	0.099288988
3031	HDAC3	ENSG00000171720	5:141628187-141628558:-	0.00403785	0.088172544	0.07444169
3032	HDAC3	ENSG00000171720	5:141629306-141629683:-	0.0079869	0.26795858	0.245106831
3033	HDAC3	ENSG00000171720	5:141629739-141629859:-	0.00829586	0.432984915	0.383178532
3034	HDAC3	ENSG00000171720	5:141630125-141634810:-	0.03472183	0.2755795	0.231347052
3035	KIAA0141	ENSG00000081791	5:141930074-141930177:+	0.00161948	0.10893671	0.073263081
3036	LARS	ENSG00000133706	5:146140261-146142871:-	0.00127171	0.094287438	0.143748319
3037	LARS	ENSG00000133706	5:146143084-146143411:-	0.00935946	0.08075145	0.118216212
3038	ABLIM3	ENSG00000173210	5:149249844-149250446:+	0.01515842	0.143086187	0.115038998
3039	ABLIM3	ENSG00000173210	5:149250505-149251358:+	0.01390473	0.128886417	0.101885319
3040	ABLIM3	ENSG00000173210	5:149251419-149252200:+	0.00102512	0.17518595	0.128775526
3041	ABLIM3	ENSG00000173210	5:149252208-149252756:+	0.03110301	0.290052439	0.247337101
3042	CSNK1A1	ENSG00000113712	5:149496860-149505446:-	0.029988	0.11244437	0.089949168
3043	TCOF1	ENSG00000070814	5:150375554-150375720:+	0.00025552	0.0758956	0.126968718
3044	TCOF1	ENSG00000070814	5:150375909-150376081:+	0.0271725	0.072271693	0.095730187
3045	TCOF1	ENSG00000070814	5:150390023-150391543:+	0.02237471	0.066235939	0.056356534
3046	ANXA6	ENSG00000197043	5:151105303-151108454:-	3.39E-14	0.132993911	0.068387969
3047	ANXA6	ENSG00000197043	5:151109846-151117126:-	6.39E-06	0.014072039	0.006928239
3048	ANXA6	ENSG00000197043	5:151124367-151126401:-	0.00021783	0.113345823	0.087333834
3049	ANXA6	ENSG00000197043	5:151126480-151128180:-	2.52E-05	0.120578329	0.092866447
3050	STK10	ENSG00000072786	5:172061268-172064719:-	0.00015068	0.299651976	0.22640724
3051	DUSP1	ENSG00000120129	5:172769794-172770160:-	0.02342261	0.170511624	0.154555413
3052	DUSP1	ENSG00000120129	5:172770306-172770585:-	0.03013741	0.219728376	0.203047943
3053	SFXN1	ENSG00000164466	5:175521968-175522374:+	0.04839275	0.100435402	0.074157461
3054	RNF44	ENSG00000146083	5:176529090-176529287:-	0.00043283	0.181298502	0.1391405
3055	UIMC1	ENSG00000087206	5:176958154-176968554:-	9.82E-05	0.046269842	0.062621536
3056	ZNF346	ENSG00000113761	5:177050936-177062057:+	0.0302853	0.098163465	0.1343663
3057	RAB24	ENSG00000169228	5:177301987-177302127:-	0.0097173	0.203089122	0.166615448
3058	RAB24	ENSG00000169228	5:177302178-177302396:-	0.0001669	0.675504768	0.604137247
3059	GRK6	ENSG00000198055	5:177430967-177431994:+	0.0219119	0.07746655	0.111507204
3060	DDX41	ENSG00000183258	5:177513082-177513352:-	0.00111594	0.102288907	0.082755061
3061	DDX41	ENSG00000183258	5:177515258-177515684:-	0.0223938	0.34270472	0.407404948
3062	DDX41	ENSG00000183258	5:177515821-177515928:-	0.0215119	0.175183944	0.214733478
3063	DDX41	ENSG00000183258	5:177516835-177516918:-	0.0003615	0.218844001	0.167503171
3064	FAM193B	ENSG00000146067	5:177532200-177532442:-	0.00071989	0.147034234	0.159970284
3065	B4GALT7	ENSG00000027847	5:177607527-177608538:+	0.00045957	0.159450088	0.116907626
3066	B4GALT7	ENSG00000027847	5:177608622-177608909:+	0.00977386	0.142582028	0.115492187
3067	B4GALT7	ENSG00000027847	5:177609014-177609539:+	0.03574123	0.121188763	0.094465411
3068	HNRNPAB	ENSG00000197451	5:178210272-178210552:+	0.00010756	0.090466648	0.117241919
3069	PHYKPL	ENSG00000175309	5:178211970-178212972:-	0.00023559	0.07589824	0.051000699
3070	PHYKPL	ENSG00000175309	5:178213103-178214795:-	0.0025542	0.068297883	0.049641434
3071	RUFY1	ENSG00000176783	5:179605924-179607581:+	1.69E-05	0.224969488	0.176220186
3072	HNRNPH1	ENSG00000169045	5:179615595-179616125:-	0.0013187	0.09139501	0.070207008
3073	HNRNPH1	ENSG00000169045	5:179616218-179617020:-	0.00469517	0.159905678	0.154766616
3074	HNRNPH1	ENSG00000169045	5:179617110-179617513:-	0.00013136	0.228668374	0.147332168
3075	MGAT4B	ENSG00000161013	5:179798434-179798512:-	0.02048207	0.165759857	0.140906494
3076	MGAT4B	ENSG00000161013	5:179800597-179800906:-	0.00141659	0.101662509	0.074643148
3077	MGAT4B	ENSG00000161013	5:179800953-179801333:-	0.00193748	0.1763883	0.133167988
3078	MRNIP	ENSG00000161010	5:179837885-179840871:-	0.00099893	0.614881744	0.558249675

S/N	Gene	Ensembl ID	Position of retained intron	p-value	AD_IR_average	Cont_IR_average
3079	TBC1D9B	ENSG00000197226	5:179870495-179871461:-	0.01253459	0.116207283	0.094793662
3080	RNF130	ENSG00000113269	5:179955669-179963470:-	5.05E-08	0.112517519	0.071668286
3081	TRIM41	ENSG00000146063	5:181230839-181232658:+	0.04297727	0.117637248	0.10525244
3082	TRIM41	ENSG00000146063	5:181232889-181233412:+	0.0014316	0.217907641	0.178202412
3083	TRIM41	ENSG00000146063	5:181233435-181233635:+	0.00179134	0.304813866	0.258812682
3084	NQO2	ENSG00000124588	6:3016985-3019478:+	0.03475562	0.186550132	0.210968782
3085	PSMG4	ENSG00000180822	6:3259196-3263683:+	6.13E-06	0.239669902	0.162760639
3086	PSMG4	ENSG00000180822	6:3263759-3267590:+	1.22E-05	0.298720526	0.214797839
3087	PRPF4B	ENSG00000112739	6:4060686-4061014:+	0.0037717	0.797423049	0.744566961
3088	PRPF4B	ENSG00000112739	6:4061147-4061654:+	0.00375889	0.712375134	0.664989273
3089	PRPF4B	ENSG00000112739	6:4062299-4062556:+	1.05E-08	0.418676085	0.273246582
3090	PRPF4B	ENSG00000112739	6:4063119-4064800:+	9.55E-13	0.808177195	0.663802091
3091	RIOK1	ENSG00000124784	6:7411451-7412888:+	2.81E-06	0.109459623	0.063351581
3092	RIOK1	ENSG00000124784	6:7412942-7414237:+	0.00328644	0.253792493	0.205439113
3093	RANBP9	ENSG00000010017	6:13632521-13634430:-	0.02286143	0.221586713	0.201595091
3094	BTN2A2	ENSG00000124508	6:26390707-26390802:+	0.03340167	0.2232864	0.183038775
3095	BTN2A1	ENSG00000112763	6:26459828-26463243:+	0.00792574	0.076713352	0.111166151
3096	BTN2A1	ENSG00000112763	6:26465973-26466061:+	0.04763268	0.15756356	0.127872895
3097	PGBD1	ENSG00000137338	6:28296945-28297894:+	0.00103384	0.382355378	0.326802597
3098	TRIM27	ENSG00000204713	6:28904665-28907235:-	3.49E-05	0.222007029	0.163513517
3099	TRIM27	ENSG00000204713	6:28907262-28908807:-	2.87E-05	0.196940757	0.126734639
3100	GABBR1	ENSG00000204681	6:29624024-29627485:-	0.01601894	0.108799687	0.094627517
3101	RPP21	ENSG00000241370	6:30345228-30345297:+	0.00308891	0.259196415	0.221562883
3102	PRR3	ENSG00000204576	6:30562124-30562388:+	0.00115066	0.330573168	0.254821581
3103	ATAT1	ENSG00000137343	6:30640422-30640534:+	0.00187099	0.085037284	0.075001754
3104	ATAT1	ENSG00000137343	6:30640603-30642175:+	0.006224	0.18051912	0.175951638
3105	C6orf136	ENSG00000204564	6:30649959-30650993:+	0.03454486	0.256034171	0.227810032
3106	FLOT1	ENSG00000137312	6:30740798-30741189:-	0.00366064	0.156186211	0.147848014
3107	FLOT1	ENSG00000137312	6:30741333-30741613:-	0.0108209	0.216418107	0.204711369
3108	GTF2H4	ENSG00000213780	6:30911767-30912013:+	0.00046656	0.214321989	0.285689717
3109	VARS2	ENSG00000137411	6:30917806-30918826:+	4.66E-06	0.412792354	0.357852403
3110	VARS2	ENSG00000137411	6:30918915-30919757:+	7.28E-07	0.418945671	0.358570117
3111	VARS2	ENSG00000137411	6:30923231-30923352:+	4.64E-10	0.1109709	0.066439194
3112	VARS2	ENSG00000137411	6:30923505-30924353:+	7.02E-10	0.149822513	0.093816186
3113	CCHCR1	ENSG00000204536	6:31142716-31142962:-	0.00201317	0.460916829	0.387658766
3114	CCHCR1	ENSG00000204536	6:31143134-31143261:-	0.04042301	0.296705659	0.253499506
3115	CCHCR1	ENSG00000204536	6:31143413-31144686:-	0.00568524	0.118998028	0.094288579
3116	CCHCR1	ENSG00000204536	6:31144788-31144884:-	0.01716059	0.141397068	0.17335147
3117	CCHCR1	ENSG00000204536	6:31145302-31145447:-	0.00321726	0.161350776	0.122094027
3118	CCHCR1	ENSG00000204536	6:31145493-31145695:-	0.01788042	0.159951452	0.128435152
3119	HLA-B	ENSG00000234745	6:31355592-31356166:-	0.00861703	0.207166605	0.173017627
3120	DDX39B	ENSG00000198563	6:31531405-31532779:-	0.01379408	0.108076967	0.095137043
3121	DDX39B	ENSG00000198563	6:31532911-31535366:-	0.00064132	0.572819207	0.520040338
3122	PRRC2A	ENSG00000204469	6:31625871-31626019:+	0.01641891	0.149933848	0.128944032
3123	PRRC2A	ENSG00000204469	6:31628239-31629143:+	0.00452967	0.137245251	0.172137739
3124	GPANK1	ENSG00000204438	6:31664577-31664840:-	0.00877719	0.073937363	0.097872887
3125	CSNK2B	ENSG00000204435	6:31668654-31669096:+	9.89E-12	0.151180941	0.094906599
3126	CSNK2B	ENSG00000204435	6:31669172-31669318:+	0.00114235	0.256588991	0.215882519
3127	ABHD16A	ENSG00000204427	6:31688786-31689014:-	7.58E-05	0.171539739	0.127329425
3128	ABHD16A	ENSG00000204427	6:31689119-31689580:-	2.47E-08	0.144850034	0.08768169
3129	ABHD16A	ENSG00000204427	6:31689704-31690077:-	0.00017386	0.095604084	0.072883092
3130	VARS	ENSG00000204394	6:31782845-31783095:-	0.02619161	0.093528447	0.075021175
3131	EHMT2	ENSG00000204371	6:31888747-31888968:-	0.01611821	0.087119256	0.084884992
3132	EHMT2	ENSG00000204371	6:31889070-31889227:-	0.00364409	0.103215637	0.09341516
3133	NELFE	ENSG00000204356	6:31953831-31954079:-	0.00035841	0.463722732	0.389760065
3134	SKIV2L	ENSG00000204351	6:31961094-31961195:+	0.00053713	0.066839209	0.096638186
3135	SKIV2L	ENSG00000204351	6:31968082-31968327:+	0.00042303	0.560236463	0.484489844

S/N	Gene	Ensembl ID	Position of retained intron	p-value	AD_IR_average	Cont_IR_average
3136	DXO	ENSG00000204348	6:31970024-31970108:-	6.76E-05	0.355997195	0.289050974
3137	DXO	ENSG00000204348	6:31970203-31970342:-	0.01716014	0.510477073	0.457658506
3138	DXO	ENSG00000204348	6:31970478-31970605:-	0.02870707	0.492691378	0.447404818
3139	STK19	ENSG00000204344	6:31979553-31980450:+	0.00028497	0.31201772	0.260950455
3140	TNXB	ENSG00000168477	6:32050321-32052669:-	8.04E-05	0.143433232	0.210135768
3141	ATF6B	ENSG00000213676	6:32115968-32116479:-	6.99E-05	0.634105537	0.53530713
3142	ATF6B	ENSG00000213676	6:32117404-32117586:-	1.14E-06	0.108878794	0.062580911
3143	ATF6B	ENSG00000213676	6:32117694-32117858:-	4.47E-05	0.155944029	0.104833087
3144	ATF6B	ENSG00000213676	6:32118866-32118955:-	6.32E-05	0.260053779	0.189088584
3145	PPT2-EGFL	ENSG00000258388	6:32157924-32162567:+	1.25E-05	0.145437857	0.105228834
3146	PPT2	ENSG00000221988	6:32162622-32162806:+	0.00483569	0.446873805	0.40684139
3147	EGFL8	ENSG00000241404	6:32166620-32166700:+	9.02E-05	0.238196622	0.174133234
3148	EGFL8	ENSG00000241404	6:32167005-32167086:+	0.00208319	0.303234293	0.251211299
3149	AGER	ENSG00000204305	6:32181632-32182246:-	0.00140354	0.122387874	0.099359332
3150	AGER	ENSG00000204305	6:32182388-32182567:-	0.00384544	0.13625651	0.105632024
3151	AGER	ENSG00000204305	6:32182698-32182840:-	0.01097752	0.240105145	0.19620546
3152	PBX2	ENSG00000204304	6:32186474-32186603:-	0.01054819	0.082615957	0.096204986
3153	TAP2	ENSG00000204267	6:32830440-32830617:-	0.03340963	0.075193448	0.096870327
3154	COL11A2	ENSG00000204248	6:33163818-33164266:-	3.91E-10	0.211220276	0.09447041
3155	COL11A2	ENSG00000204248	6:33165984-33166170:-	0.00034992	0.090692164	0.063515865
3156	COL11A2	ENSG00000204248	6:33167123-33167263:-	5.10E-10	0.076841776	0.038400781
3157	RXRB	ENSG00000204231	6:33195454-33195569:-	0.01217894	0.062748634	0.084837209
3158	RXRB	ENSG00000204231	6:33195702-33195906:-	0.00546557	0.174851838	0.224691466
3159	RXRB	ENSG00000204231	6:33196036-33196433:-	0.00171457	0.191827277	0.247786026
3160	VPS52	ENSG00000223501	6:33264497-33264781:-	0.04200192	0.443480854	0.422263156
3161	WDR46	ENSG00000227057	6:33280522-33280673:-	1.91E-07	0.316181874	0.423965844
3162	WDR46	ENSG00000227057	6:33280987-33286794:-	0.01409967	0.11550871	0.138844573
3163	WDR46	ENSG00000227057	6:33286894-33287090:-	0.00780257	0.086414236	0.101573934
3164	WDR46	ENSG00000227057	6:33287505-33287613:-	0.01076898	0.087236341	0.109812077
3165	WDR46	ENSG00000227057	6:33287718-33287964:-	0.0139278	0.0861516	0.112435545
3166	WDR46	ENSG00000227057	6:33288235-33288357:-	0.0331064	0.116953901	0.150313134
3167	WDR46	ENSG00000227057	6:33288694-33288803:-	2.72E-05	0.142353645	0.219523386
3168	RGL2	ENSG00000237441	6:33293524-33293603:-	1.09E-07	0.492023476	0.439421104
3169	RGL2	ENSG00000237441	6:33293699-33293794:-	7.57E-10	0.477603927	0.384509571
3170	RGL2	ENSG00000237441	6:33293916-33294033:-	6.71E-05	0.337669439	0.269011896
3171	RGL2	ENSG00000237441	6:33294066-33294687:-	0.01238461	0.103777006	0.081640075
3172	RGL2	ENSG00000237441	6:33295759-33296027:-	1.67E-07	0.459904439	0.564741325
3173	PHF1	ENSG00000112511	6:33413557-33413735:+	5.65E-06	0.315512768	0.248036519
3174	PHF1	ENSG00000112511	6:33413831-33414040:+	6.44E-05	0.164165221	0.124197873
3175	PHF1	ENSG00000112511	6:33414829-33414954:+	0.01970833	0.065897906	0.053567491
3176	SYNGAP1	ENSG00000197283	6:33444617-33446574:+	0.0190649	0.084014621	0.068731892
3177	BAK1	ENSG00000030110	6:33573907-33574033:-	0.02546618	0.178287713	0.215376408
3178	ZNF76	ENSG00000065029	6:35287845-35290265:+	0.03687072	0.204926496	0.23785326
3179	ZNF76	ENSG00000065029	6:35293915-35294455:+	5.71E-11	0.199272662	0.1248075
3180	PPARD	ENSG00000112033	6:35411217-35420126:+	0.00501796	0.160569055	0.130822049
3181	PPARD	ENSG00000112033	6:35421958-35423945:+	0.01374169	0.121740828	0.107819696
3182	FANCE	ENSG00000112039	6:35462914-35466243:+	0.0049419	0.092369466	0.064066806
3183	ADCY10P1	ENSG00000161912	6:41140069-41140597:+	0.00037389	0.15242451	0.115482951
3184	TFEB	ENSG00000112561	6:41687169-41687752:-	0.00012232	0.224836223	0.203337318
3185	UBR2	ENSG00000024048	6:42688386-42689568:+	0.00432136	0.098975151	0.07846622
3186	PEX6	ENSG00000124587	6:42964471-42964789:-	2.61E-07	0.10642274	0.072945658
3187	PEX6	ENSG00000124587	6:42964929-42965074:-	0.00090733	0.199388113	0.160436836
3188	PEX6	ENSG00000124587	6:42965152-42965251:-	9.40E-05	0.153195404	0.113063206
3189	PEX6	ENSG00000124587	6:42965368-42965680:-	3.52E-07	0.112161689	0.075740382
3190	PEX6	ENSG00000124587	6:42966105-42966241:-	0.01490885	0.509501293	0.464749234
3191	PEX6	ENSG00000124587	6:42966657-42966781:-	0.01339855	0.171772111	0.141757855
3192	PEX6	ENSG00000124587	6:42966858-42967367:-	0.00013335	0.197229607	0.147682103

S/N	Gene	Ensembl ID	Position of retained intron	p-value	AD_IR_average	Cont_IR_average
3193	CUL7	ENSG00000044090	6:43038692-43038841:-	0.0003757	0.337090183	0.276196455
3194	MRPL2	ENSG00000112651	6:43054486-43055544:-	0.03404318	0.107742726	0.09142993
3195	MRPL2	ENSG00000112651	6:43055618-43055896:-	0.02131888	0.132721393	0.108221852
3196	MRPL2	ENSG00000112651	6:43056007-43056080:-	0.03397773	0.117708846	0.098426965
3197	KLC4	ENSG00000137171	6:43066525-43066995:+	0.00432787	0.171311533	0.141201926
3198	KLC4	ENSG00000137171	6:43073338-43073901:+	0.00058428	0.120075576	0.08996783
3199	CUL9	ENSG00000112659	6:43221321-43221684:+	0.00099669	0.271488666	0.219395158
3200	CUL9	ENSG00000112659	6:43221778-43222315:+	0.0110963	0.144674033	0.116635997
3201	TTBK1	ENSG00000146216	6:43255114-43255551:+	0.01708137	0.119989966	0.149618601
3202	TTBK1	ENSG00000146216	6:43259269-43259530:+	0.02060567	0.12123331	0.1457308
3203	ABCC10	ENSG00000124574	6:43445314-43445598:+	0.00651851	0.30802613	0.276708674
3204	TJAP1	ENSG00000137221	6:43501687-43502282:+	0.01050741	0.141705841	0.185766408
3205	YIPF3	ENSG00000137207	6:43513200-43513358:-	0.01412369	0.130926312	0.116168304
3206	POLR1C	ENSG00000171453	6:43520427-43520624:+	0.00844104	0.126244614	0.098665252
3207	XPO5	ENSG00000124571	6:43527731-43528158:-	0.02463131	0.309283222	0.269786351
3208	TMEM63B	ENSG00000137216	6:44148945-44149858:+	1.46E-06	0.483542	0.36183561
3209	TMEM63B	ENSG00000137216	6:44149965-44150223:+	3.11E-05	0.220439248	0.124820904
3210	TMEM63B	ENSG00000137216	6:44150629-44151845:+	0.00091794	0.239921317	0.187683048
3211	TMEM63B	ENSG00000137216	6:44152008-44152592:+	0.00156335	0.197566063	0.156566716
3212	TMEM63B	ENSG00000137216	6:44152698-44153675:+	0.0456976	0.357010866	0.320287883
3213	TMEM63B	ENSG00000137216	6:44153843-44154072:+	7.21E-05	0.163917365	0.122434373
3214	TMEM63B	ENSG00000137216	6:44154188-44154364:+	0.00013252	0.163274287	0.118940305
3215	GSTA4	ENSG00000170899	6:52985583-52987356:-	0.0011005	0.254091184	0.220704786
3216	FBXO9	ENSG00000112146	6:53092824-53093465:+	0.03665746	0.420833341	0.384555974
3217	DST	ENSG00000151914	6:56463764-56464684:-	0.00387753	0.074117035	0.071732543
3218	DST	ENSG00000151914	6:56464756-56466077:-	0.00053097	0.049165335	0.040889584
3219	HMGN3	ENSG00000118418	6:79202389-79203579:-	0.00187563	0.12790548	0.096625071
3220	DOPEY1	ENSG00000083097	6:83151659-83151882:+	0.0090615	0.145535138	0.126353851
3221	RIPPLY2	ENSG00000203877	6:83853773-83854096:+	0.0006649	0.105444404	0.075421463
3222	RIPPLY2	ENSG00000203877	6:83854161-83857241:+	0.00018123	0.188980398	0.142951192
3223	SNX14	ENSG00000135317	6:85508059-85513799:-	0.00036465	0.092492761	0.07087964
3224	RP3-382I1(	ENSG00000213204	6:87509175-87511398:+	0.00204081	0.256875756	0.200453636
3225	RARS2	ENSG00000146282	6:87514499-87514956:-	0.00047134	0.130596894	0.089687587
3226	RARS2	ENSG00000146282	6:87516880-87518168:-	0.00440087	0.136879045	0.105185492
3227	ANKRD6	ENSG00000135299	6:89624057-89624538:+	0.01975791	0.148305638	0.199430317
3228	MICAL1	ENSG00000135596	6:109444995-109445196:-	0.00058475	0.098774163	0.078453655
3229	MICAL1	ENSG00000135596	6:109445290-109445415:-	0.0114476	0.106560767	0.088821648
3230	MICAL1	ENSG00000135596	6:109445862-109446135:-	0.04857728	0.119989205	0.109715329
3231	MICAL1	ENSG00000135596	6:109446772-109447072:-	0.01708242	0.130054673	0.111314165
3232	MICAL1	ENSG00000135596	6:109447229-109447356:-	0.00229593	0.267288256	0.237830361
3233	MICAL1	ENSG00000135596	6:109447722-109447874:-	0.00935176	0.490701159	0.463984286
3234	MICAL1	ENSG00000135596	6:109447963-109448202:-	0.00572133	0.425427463	0.382327961
3235	MICAL1	ENSG00000135596	6:109448393-109448731:-	0.00187656	0.210405054	0.173394543
3236	MICAL1	ENSG00000135596	6:109449783-109449969:-	0.03228989	0.534484439	0.481549688
3237	FAM184A	ENSG00000111879	6:118964771-118966834:-	0.00452606	0.091418779	0.065563549
3238	FAM184A	ENSG00000111879	6:118966952-118974427:-	0.00019831	0.054510499	0.029945571
3239	FAM184A	ENSG00000111879	6:118966952-118975023:-	2.71E-05	0.051684127	0.029369684
3240	SGK1	ENSG00000118515	6:134170915-134171022:-	0.02736717	0.125634571	0.096336985
3241	MAP7	ENSG00000135525	6:136344238-136345855:-	3.19E-07	0.399783244	0.263424539
3242	CCDC28A	ENSG00000024862	6:138779985-138785226:+	0.00854002	0.101654728	0.11790233
3243	CCDC28A	ENSG00000024862	6:138788388-138792748:+	2.34E-06	0.105344322	0.072522104
3244	PEX3	ENSG00000034693	6:143472328-143474785:+	0.04345065	0.107676903	0.096363461
3245	PEX3	ENSG00000034693	6:143474856-143479075:+	0.00290889	0.169680029	0.125414032
3246	PEX3	ENSG00000034693	6:143479198-143485151:+	2.43E-05	0.178700459	0.106917005
3247	PEX3	ENSG00000034693	6:143485248-143489142:+	3.56E-06	0.278388995	0.169681439
3248	KATNA1	ENSG00000186625	6:149595234-149597062:-	0.00734917	0.233164832	0.196545716
3249	KATNA1	ENSG00000186625	6:149597189-149597506:-	3.35E-05	0.153209057	0.10940679

S/N	Gene	Ensembl ID	Position of retained intron	p-value	AD_IR_average	Cont_IR_average
3250	DYNLT1	ENSG00000146425	6:158636897-158637127:-	0.03617483	0.09495451	0.106187594
3251	MAP3K4	ENSG00000085511	6:161115302-161116849:+	0.01274501	0.107166554	0.091107643
3252	SFT2D1	ENSG00000198818	6:166322886-166324536:-	0.0123498	0.118847363	0.093301226
3253	SFT2D1	ENSG00000198818	6:166324595-166326131:-	0.00856871	0.093733622	0.068440269
3254	FAM120B	ENSG00000112584	6:170388493-170391012:+	0.00303602	0.098921744	0.076633186
3255	FAM120B	ENSG00000112584	6:170391121-170395486:+	8.33E-06	0.27657533	0.205211822
3256	DNAAF5	ENSG00000164818	7:770618-774047:+	0.00050149	0.205474485	0.266522725
3257	SUN1	ENSG00000164828	7:842130-843205:+	0.03041512	0.076653328	0.117672966
3258	COX19	ENSG00000240230	7:969456-973180:-	3.43E-05	0.287336634	0.229137752
3259	MICALL2	ENSG00000164877	7:1435147-1436741:-	0.00073149	0.49535689	0.407882961
3260	MICALL2	ENSG00000164877	7:1436856-1437534:-	0.00499427	0.430369795	0.354201519
3261	MICALL2	ENSG00000164877	7:1437608-1437889:-	0.00323514	0.144282689	0.094431928
3262	MICALL2	ENSG00000164877	7:1437980-1438096:-	0.00032011	0.188831102	0.122682794
3263	MICALL2	ENSG00000164877	7:1440085-1440590:-	0.00059908	0.361585623	0.273256268
3264	INTS1	ENSG00000164880	7:1483853-1484002:-	0.00205852	0.516579	0.432922065
3265	BRAT1	ENSG00000106009	7:2539888-2540978:-	0.01071065	0.690031585	0.647060338
3266	IQCE	ENSG00000106012	7:2607227-2610043:+	0.00768327	0.098114294	0.077632957
3267	TTYH3	ENSG00000136295	7:2647253-2647417:+	6.09E-05	0.233790234	0.162223677
3268	TTYH3	ENSG00000136295	7:2649988-2652186:+	0.00806958	0.14681345	0.115376875
3269	TTYH3	ENSG00000136295	7:2658459-2658939:+	0.00051597	0.106647991	0.077682781
3270	AP5Z1	ENSG00000242802	7:4789929-4790458:+	0.0332605	0.525327634	0.558974584
3271	RADIL	ENSG00000157927	7:4822554-4832140:-	0.04236298	0.127283602	0.105515345
3272	FSCN1	ENSG00000075618	7:5604030-5605271:+	0.00851672	0.11692072	0.100697749
3273	USP42	ENSG00000106346	7:6157055-6159449:+	0.00409762	0.191190237	0.159535675
3274	USP42	ENSG00000106346	7:6159493-6160554:+	0.00444808	0.15933538	0.131302278
3275	C7orf26	ENSG00000146576	7:6602140-6607959:+	0.00584179	0.369348768	0.417777558
3276	ZNF316	ENSG00000205903	7:6638009-6639041:+	0.00013242	0.113714812	0.161685403
3277	ZNF316	ENSG00000205903	7:6641962-6642381:+	2.76E-05	0.200715839	0.278214423
3278	MIOS	ENSG00000164654	7:7588563-7589404:+	0.01964823	0.172915717	0.135437624
3279	MIOS	ENSG00000164654	7:7589563-7594979:+	0.01462139	0.120864127	0.095839064
3280	ICA1	ENSG00000003147	7:8114044-8127872:-	0.02118698	0.100378915	0.079179399
3281	MALSU1	ENSG00000156928	7:23301017-23307867:+	0.00250195	0.117462967	0.084481642
3282	TRA2A	ENSG00000164548	7:23506266-23507419:-	0.02815951	0.137405346	0.114020955
3283	HNRNPA2E	ENSG00000122566	7:26192338-26192494:-	0.03000174	0.173746288	0.19748991
3284	GARS	ENSG00000106105	7:30628669-30631447:+	3.23E-09	0.123478005	0.070056349
3285	GARS	ENSG00000106105	7:30631541-30632246:+	0.02062729	0.19940124	0.171911645
3286	NT5C3A	ENSG00000122643	7:33014831-33015669:-	0.00286059	0.144105471	0.108248857
3287	STARD3NL	ENSG00000010270	7:38215105-38217024:+	0.00304192	0.068809525	0.055589987
3288	YAE1D1	ENSG00000241127	7:39570627-39572276:+	0.00016096	0.262269899	0.201693244
3289	MRPS24	ENSG00000062582	7:43869376-43869456:-	0.00464587	0.343375366	0.294777494
3290	URGCP-MF	ENSG00000270617	7:43881697-43881906:-	0.04131717	0.11825216	0.110034578
3291	UBE2D4	ENSG00000078967	7:43942857-43942953:+	0.03574278	0.176328224	0.148840692
3292	DBNL	ENSG00000136279	7:44059658-44060047:+	0.02905411	0.115447574	0.090704277
3293	DBNL	ENSG00000136279	7:44060153-44060776:+	0.01519164	0.116176456	0.088356177
3294	PGAM2	ENSG00000164708	7:44062930-44064831:-	0.00726831	0.229033141	0.1937832
3295	POLD2	ENSG00000106628	7:44114945-44115294:-	0.00163702	0.199618813	0.159129039
3296	POLD2	ENSG00000106628	7:44115893-44116114:-	3.46E-05	0.134953772	0.100498741
3297	POLD2	ENSG00000106628	7:44116272-44116429:-	1.97E-05	0.188762317	0.137635855
3298	POLD2	ENSG00000106628	7:44117247-44117618:-	0.0163099	0.159261388	0.139237513
3299	CAMK2B	ENSG00000058404	7:44219522-44220059:-	0.00987415	0.070867566	0.084072309
3300	ZMIZ2	ENSG00000122515	7:44759460-44760424:+	0.00657879	0.357581951	0.404825078
3301	NACAD	ENSG00000136274	7:45081263-45081600:-	0.00033733	0.153399611	0.120238247
3302	NACAD	ENSG00000136274	7:45081672-45081754:-	0.03539128	0.20225033	0.173473634
3303	GBAS	ENSG00000146729	7:55978395-55981472:+	0.02678321	0.1340999	0.096778148
3304	CCT6A	ENSG00000146731	7:56061849-56062682:+	0.0003458	0.078569882	0.111436621
3305	SUMF2	ENSG00000129103	7:56076889-56078101:+	0.00033121	0.122196154	0.089352306
3306	SUMF2	ENSG00000129103	7:56078186-56078363:+	0.0091879	0.222613374	0.190078092

S/N	Gene	Ensembl ID	Position of retained intron	p-value	AD_IR_average	Cont_IR_average
3307	GUSB	ENSG00000169919	7:65976202-65979398:-	0.00640797	0.114703949	0.097341661
3308	ASL	ENSG00000126522	7:66086662-66086743:+	0.01799229	0.236269527	0.194370113
3309	AC068533.	ENSG00000249319	7:66087791-66088806:+	0.03795875	0.146795417	0.133408162
3310	SBDSP1	ENSG00000225648	7:72830813-72831601:+	0.01390191	0.137194804	0.128226461
3311	MLXIPL	ENSG00000009950	7:73593983-73594273:-	0.01089723	0.174949223	0.140943287
3312	MLXIPL	ENSG00000009950	7:73594403-73595636:-	7.89E-06	0.138811148	0.090771281
3313	MLXIPL	ENSG00000009950	7:73595760-73595841:-	0.00020647	0.129569026	0.085993904
3314	WBSCR22	ENSG00000071462	7:73683804-73686635:+	0.00261292	0.075763067	0.068036269
3315	WBSCR22	ENSG00000071462	7:73692646-73693328:+	1.50E-07	0.11124644	0.07514534
3316	WBSCR22	ENSG00000071462	7:73694050-73697604:+	8.21E-05	0.268596634	0.221305873
3317	ELN	ENSG00000049540	7:74065997-74066731:+	2.95E-05	0.060865995	0.044324671
3318	GTF2IRD1	ENSG00000006704	7:74601180-74602364:+	3.84E-09	0.483542671	0.356058558
3319	POR	ENSG00000127948	7:75985207-75985578:+	0.00561266	0.165867179	0.133415416
3320	TMEM120,	ENSG00000189077	7:75987428-75987537:-	0.00912253	0.174875617	0.138639412
3321	TMEM120,	ENSG00000189077	7:75989224-75992143:-	6.78E-05	0.169706617	0.132153416
3322	AKAP9	ENSG00000127914	7:92105763-92107292:+	6.16E-07	0.085226687	0.054502262
3323	AKAP9	ENSG00000127914	7:92108633-92110121:+	6.94E-12	0.343438207	0.217779826
3324	PEX1	ENSG00000127980	7:92487541-92489292:-	0.03643886	0.127591707	0.100157556
3325	PEX1	ENSG00000127980	7:92489423-92489713:-	0.03592727	0.179555937	0.146703001
3326	PEX1	ENSG00000127980	7:92489911-92491271:-	0.042597	0.180577987	0.135885665
3327	RBM48	ENSG00000127993	7:92534970-92536850:+	0.00791345	0.432267573	0.389909896
3328	SGCE	ENSG00000127990	7:94585515-94588688:-	0.02421993	0.085735084	0.060102261
3329	PDAP1	ENSG00000106244	7:99396740-99397861:-	3.48E-07	0.178029216	0.131271509
3330	ATP5J2-PT	ENSG00000248919	7:99420149-99423774:-	0.04432482	0.082035052	0.094864466
3331	CPSF4	ENSG00000160917	7:99450371-99450701:+	0.01915673	0.101426985	0.08227995
3332	ZSCAN21	ENSG00000166529	7:100057405-100057697:+	0.00043733	0.135069885	0.190089134
3333	COPS6	ENSG00000168090	7:100089746-100090398:+	0.04818432	0.148206145	0.139717234
3334	MCM7	ENSG00000166508	7:100099403-100099588:-	0.00744359	0.182609066	0.232152284
3335	TAF6	ENSG00000106290	7:100110073-100110199:-	1.92E-06	0.26212275	0.193840865
3336	CNPY4	ENSG00000166997	7:100122577-100122783:+	0.0012056	0.22533727	0.172978918
3337	C7orf43	ENSG00000146826	7:100155825-100156385:-	0.02085578	0.276837256	0.257397961
3338	C7orf43	ENSG00000146826	7:100156992-100157093:-	0.01711224	0.093719756	0.076710982
3339	GATS	ENSG00000239521	7:100222720-100223569:-	1.26E-07	0.415618387	0.312689918
3340	STAG3L5P	ENSG00000242294	7:100345968-100349716:+	0.00259984	0.11753622	0.126412577
3341	STAG3L5P-	ENSG00000272752	7:100349887-100351251:+	0.00835351	0.162976185	0.117884216
3342	STAG3L5P-	ENSG00000272752	7:100351410-100352162:+	0.00017777	0.805837841	0.848693169
3343	PILRB	ENSG00000121716	7:100352914-100352996:+	0.00047854	0.323101671	0.398158208
3344	PILRB	ENSG00000121716	7:100353123-100353209:+	7.29E-05	0.227105183	0.171945235
3345	PILRB	ENSG00000121716	7:100353270-100353372:+	0.0166028	0.109077001	0.084102762
3346	PILRB	ENSG00000121716	7:100354012-100355142:+	0.00010819	0.086424809	0.060959927
3347	ZCWPW1	ENSG00000078487	7:100401336-100403693:-	0.03436321	0.085840437	0.071969048
3348	ZCWPW1	ENSG00000078487	7:100403785-100404177:-	0.00053446	0.228600948	0.181641961
3349	ZCWPW1	ENSG00000078487	7:100404244-100405012:-	0.00031054	0.24737922	0.204892704
3350	PPP1R35	ENSG00000160813	7:100435767-100435847:-	0.00449785	0.558596207	0.488616013
3351	TSC22D4	ENSG00000166925	7:100467600-100467694:-	0.00198606	0.094489752	0.074352855
3352	TSC22D4	ENSG00000166925	7:100467600-100474273:-	0.00482472	0.028591719	0.017148482
3353	PCOLCE	ENSG00000106333	7:100605812-100606415:+	0.00016887	0.676868098	0.593413442
3354	MOSPD3	ENSG00000106330	7:100612996-100613193:+	5.21E-05	0.12793575	0.0901774
3355	MOSPD3	ENSG00000106330	7:100613706-100614866:+	1.58E-06	0.273644727	0.192620575
3356	ACTL6B	ENSG00000077080	7:100643326-100646248:-	0.00663862	0.180790666	0.144207635
3357	ACTL6B	ENSG00000077080	7:100647533-100648555:-	0.00012195	0.348087491	0.271289696
3358	SLC12A9	ENSG00000146828	7:100861262-100861391:+	2.71E-05	0.422281671	0.358848597
3359	SRRT	ENSG00000087087	7:100881413-100881658:+	0.01760887	0.082137054	0.105325121
3360	SRRT	ENSG00000087087	7:100888143-100888256:+	0.00857781	0.111563141	0.12996739
3361	PLOD3	ENSG00000106397	7:101210161-101210330:-	0.00875298	0.194426168	0.146703319
3362	PLOD3	ENSG00000106397	7:101210444-101210531:-	0.04635544	0.101376886	0.074783824
3363	FIS1	ENSG00000214253	7:101240906-101244897:-	1.58E-05	0.009713756	0.008886335

S/N	Gene	Ensembl ID	Position of retained intron	p-value	AD_IR_average	Cont_IR_average
3364	SH2B2	ENSG00000160999	7:102320502-102321298:+	1.48E-05	0.312475212	0.211957949
3365	PRKRIP1	ENSG00000128563	7:102376322-102395976:+	2.99E-05	0.064719937	0.076597599
3366	PRKRIP1	ENSG00000128563	7:102376322-102399547:+	0.03610913	0.063295539	0.074060018
3367	PRKRIP1	ENSG00000128563	7:102396537-102397619:+	0.01278169	0.339808585	0.299141883
3368	PRKRIP1	ENSG00000128563	7:102397698-102399547:+	0.00809185	0.0725673	0.060676565
3369	ALKBH4	ENSG00000160993	7:102457981-102459603:-	4.99E-09	0.167545761	0.107823266
3370	LRWD1	ENSG00000161036	7:102468377-102468553:+	0.00268453	0.591123293	0.527389455
3371	PMPCB	ENSG00000105819	7:103311728-103311807:+	0.00665229	0.09332751	0.07879827
3372	PMPCB	ENSG00000105819	7:103312131-103312206:+	8.33E-06	0.529152049	0.437175299
3373	DNAJC2	ENSG00000105821	7:103312643-103312946:-	0.01181936	0.432509598	0.390077364
3374	DNAJC2	ENSG00000105821	7:103313101-103315763:-	0.01420925	0.333264357	0.288912936
3375	DNAJC2	ENSG00000105821	7:103315871-103315987:-	0.04819379	0.373311768	0.400140818
3376	HBP1	ENSG00000105856	7:107186472-107186568:+	0.00350867	0.291123902	0.393295
3377	LAMB1	ENSG00000091136	7:107924389-107926182:-	1.07E-05	0.129377943	0.085613292
3378	LAMB1	ENSG00000091136	7:107929619-107931355:-	0.00366796	0.105195722	0.073991348
3379	LRRC4	ENSG00000128594	7:128030740-128030912:-	2.83E-05	0.110091582	0.1602852
3380	RBM28	ENSG00000106344	7:128315020-128317658:-	3.77E-06	0.144504754	0.104356647
3381	RBM28	ENSG00000106344	7:128317733-128317956:-	0.00028168	0.152402011	0.120137495
3382	IMPDH1	ENSG00000106348	7:128394599-128394888:-	0.00030438	0.207982728	0.152026104
3383	IMPDH1	ENSG00000106348	7:128395274-128396599:-	0.00023979	0.237471205	0.174280635
3384	IMPDH1	ENSG00000106348	7:128400891-128401014:-	0.02681787	0.132191239	0.105106519
3385	CCDC136	ENSG00000128596	7:128805901-128817757:+	5.44E-05	0.125249263	0.090470652
3386	CCDC136	ENSG00000128596	7:128810366-128811799:+	0.00256682	0.410910273	0.464993961
3387	CCDC136	ENSG00000128596	7:128812312-128812707:+	0.00125605	0.202742592	0.255335765
3388	CCDC136	ENSG00000128596	7:128817864-128821798:+	0.00539152	0.167873724	0.134092095
3389	COPG2	ENSG00000158623	7:130506806-130507273:-	9.23E-05	0.116420384	0.067323322
3390	COPG2	ENSG00000158623	7:130507372-130507684:-	0.00014499	0.113581441	0.071427159
3391	WDR91	ENSG00000105875	7:135186315-135186971:-	3.17E-05	0.162291763	0.112405204
3392	WDR91	ENSG00000105875	7:135187169-135188432:-	0.00565554	0.321336451	0.273110675
3393	WDR91	ENSG00000105875	7:135188545-135189343:-	0.00928558	0.205693737	0.168648864
3394	WDR91	ENSG00000105875	7:135195084-135196143:-	0.00378048	0.109015536	0.078975671
3395	MKRN1	ENSG00000133606	7:140455233-140455789:-	0.01634658	0.09427583	0.131620606
3396	ADCK2	ENSG00000133597	7:140690813-140694662:+	0.01683685	0.147021183	0.117544808
3397	GSTK1	ENSG00000197448	7:143263585-143264085:+	0.00109443	0.189261863	0.149781074
3398	GSTK1	ENSG00000197448	7:143264167-143264547:+	0.00015666	0.211063163	0.159402613
3399	GSTK1	ENSG00000197448	7:143264676-143264991:+	0.00236911	0.264639793	0.222585675
3400	GSTK1	ENSG00000197448	7:143267733-143268090:+	0.02007727	0.170515944	0.14179446
3401	GSTK1	ENSG00000197448	7:143268184-143268787:+	0.03849202	0.116965444	0.098193865
3402	CUL1	ENSG00000055130	7:148799388-148800501:+	0.02040181	0.093780537	0.079188055
3403	ZNF767P	ENSG00000133624	7:149549374-149550867:-	0.01258799	0.265634707	0.214075355
3404	ZNF775	ENSG00000196456	7:150408488-150410159:+	0.00543752	0.179976585	0.220779269
3405	KCNH2	ENSG00000055118	7:150950420-150950920:-	0.00035745	0.116284881	0.074274178
3406	KCNH2	ENSG00000055118	7:150951120-150951447:-	0.0011659	0.265213439	0.210015014
3407	ABCB8	ENSG00000197150	7:151034829-151035580:+	0.00241968	0.097932972	0.071843691
3408	ABCB8	ENSG00000197150	7:151035742-151035881:+	0.02148409	0.116080599	0.0938092
3409	ABCB8	ENSG00000197150	7:151040634-151040827:+	0.04053302	0.080549287	0.06998156
3410	ABCB8	ENSG00000197150	7:151040922-151041098:+	0.0066169	0.100973965	0.081973929
3411	ABCB8	ENSG00000197150	7:151041232-151041960:+	0.04798984	0.119772348	0.104562979
3412	ABCB8	ENSG00000197150	7:151042108-151043970:+	0.00051816	0.136334501	0.099861877
3413	ASIC3	ENSG00000213199	7:151051095-151051171:+	0.00868036	0.094830208	0.072476155
3414	ASIC3	ENSG00000213199	7:151051319-151051809:+	0.00961612	0.09558635	0.073634821
3415	ASIC3	ENSG00000213199	7:151051901-151051982:+	0.01473012	0.110074555	0.09029291
3416	SLC4A2	ENSG00000164889	7:151076186-151076286:+	0.00954775	0.159856939	0.131231896
3417	FASTK	ENSG00000164896	7:151077236-151077309:-	0.01796969	0.313637366	0.266790649
3418	FASTK	ENSG00000164896	7:151077401-151077620:-	3.84E-05	0.611182744	0.532239195
3419	FASTK	ENSG00000164896	7:151077780-151077878:-	0.00078274	0.423239122	0.364126597
3420	FASTK	ENSG00000164896	7:151079021-151079499:-	3.18E-07	0.471500728	0.365242532

S/N	Gene	Ensembl ID	Position of retained intron	p-value	AD_IR_average	Cont_IR_average
3421	AGAP3	ENSG00000133612	7:151134568-151141897:+	0.00629287	0.070394271	0.078175613
3422	NUB1	ENSG00000013374	7:151341846-151345347:+	6.59E-06	0.060504348	0.130787408
3423	NUB1	ENSG00000013374	7:151375943-151376633:+	0.01836301	0.133272934	0.109994747
3424	DPP6	ENSG00000130226	7:154889530-154892333:+	0.02073114	0.16912296	0.147761674
3425	PAXIP1	ENSG00000157212	7:154957294-154959889:-	0.01814913	0.100162077	0.073854668
3426	PAXIP1	ENSG00000157212	7:154959933-154960892:-	0.00078456	0.088265346	0.06153696
3427	PAXIP1	ENSG00000157212	7:154963766-154967815:-	0.02069505	0.136538817	0.116145434
3428	WDR60	ENSG00000126870	7:158926463-158926991:+	0.00116915	0.08480859	0.063496125
3429	WDR60	ENSG00000126870	7:158927043-158930454:+	0.00389379	0.094435309	0.07648173
3430	MCPH1	ENSG00000147316	8:6621691-6642993:+	0.00266779	0.100214538	0.072061231
3431	VPS37A	ENSG00000155975	8:17284616-17286346:+	0.00264949	0.093677599	0.067426723
3432	VPS37A	ENSG00000155975	8:17286427-17294986:+	0.00119321	0.163922778	0.123344755
3433	INTS10	ENSG00000104613	8:19844238-19845703:+	0.00046874	0.132550459	0.10113069
3434	INTS10	ENSG00000104613	8:19845797-19851648:+	0.03712139	0.14327501	0.129887448
3435	INTS10	ENSG00000104613	8:19849239-19851648:+	0.0019587	0.142839745	0.126211603
3436	XPO7	ENSG00000130227	8:22004030-22004994:+	0.00585397	0.155059735	0.124815458
3437	NPM2	ENSG00000158806	8:22034544-22036492:+	5.53E-05	0.214719582	0.177481695
3438	DMTN	ENSG00000158856	8:22067682-22069015:+	0.00211442	0.085651485	0.081451332
3439	DMTN	ENSG00000158856	8:22069060-22069418:+	0.00305047	0.093833571	0.09327283
3440	DMTN	ENSG00000158856	8:22080625-22080804:+	0.00092333	0.10746877	0.097432008
3441	DMTN	ENSG00000158856	8:22080870-22081112:+	0.00039862	0.097895806	0.089604125
3442	FAM160B2	ENSG00000158863	8:22094518-22096336:+	1.38E-07	0.291241646	0.204732668
3443	FAM160B2	ENSG00000158863	8:22097620-22097716:+	0.00078296	0.117478572	0.151281355
3444	FAM160B2	ENSG00000158863	8:22100972-22101439:+	7.27E-07	0.370861927	0.460253052
3445	FAM160B2	ENSG00000158863	8:22101530-22101707:+	3.00E-08	0.320356354	0.416407182
3446	FAM160B2	ENSG00000158863	8:22101851-22102174:+	1.04E-06	0.337123793	0.426821026
3447	FAM160B2	ENSG00000158863	8:22102315-22102527:+	0.00554404	0.108418982	0.126182314
3448	FAM160B2	ENSG00000158863	8:22102628-22102792:+	0.00432318	0.221491712	0.251682026
3449	BMP1	ENSG00000168487	8:22207516-22209444:+	0.02879492	0.188100346	0.21135401
3450	PPP3CC	ENSG00000120910	8:22533018-22539468:+	0.02713583	0.120891874	0.109575799
3451	SORBS3	ENSG00000120896	8:22561931-22563986:+	0.00016369	0.182271461	0.137702403
3452	SORBS3	ENSG00000120896	8:22566484-22566660:+	0.04051773	0.151986201	0.144851231
3453	SORBS3	ENSG00000120896	8:22566713-22566821:+	0.0047445	0.309590329	0.268223429
3454	AC037459.	ENSG00000248235	8:22589741-22591550:+	0.00010195	0.13851818	0.137586892
3455	PDLIM2	ENSG00000120913	8:22589741-22593732:+	0.00560008	0.132214974	0.131601595
3456	AC037459.	ENSG00000248235	8:22591668-22593732:+	0.00030503	0.131451804	0.133073171
3457	CCAR2	ENSG00000158941	8:22613136-22614091:+	0.00981422	0.172016646	0.224678026
3458	BIN3	ENSG00000147439	8:22624363-22629963:-	2.80E-05	0.240965635	0.178320944
3459	CHMP7	ENSG00000147457	8:23249381-23255246:+	0.00104068	0.065037389	0.099448394
3460	CHMP7	ENSG00000147457	8:23258449-23258731:+	0.00766845	0.117449741	0.107537042
3461	R3HCC1	ENSG00000104679	8:23294864-23295966:+	7.34E-05	0.135854146	0.1052231
3462	STMN4	ENSG00000015592	8:27236905-27239225:-	0.03187608	0.009776266	0.009599241
3463	PTK2B	ENSG00000120899	8:27431016-27431397:+	0.00758328	0.489146683	0.415485325
3464	PTK2B	ENSG00000120899	8:27451509-27453113:+	0.00274699	0.157634654	0.111309816
3465	EPHX2	ENSG00000120915	8:27518072-27520882:+	0.00017803	0.099279674	0.0625024
3466	EPHX2	ENSG00000120915	8:27543829-27544185:+	5.24E-14	0.237032854	0.1018437
3467	EPHX2	ENSG00000120915	8:27544244-27544443:+	1.25E-07	0.386943488	0.272295903
3468	LETM2	ENSG00000165046	8:38407463-38408211:+	0.01525125	0.76428072	0.716920395
3469	FGFR1	ENSG00000077782	8:38418373-38419532:-	0.04071395	0.094087223	0.110175548
3470	GPAT4	ENSG00000158669	8:41609955-41610735:+	0.0125522	0.446514439	0.414742273
3471	GPAT4	ENSG00000158669	8:41615048-41618683:+	6.31E-06	0.194566378	0.140175044
3472	IKBKB	ENSG00000104365	8:42319646-42320734:+	0.02098477	0.33477139	0.332440494
3473	IKBKB	ENSG00000104365	8:42322153-42322346:+	0.00694734	0.111783905	0.137534417
3474	IKBKB	ENSG00000104365	8:42326097-42329123:+	0.00024743	0.213636477	0.169250988
3475	IKBKB	ENSG00000104365	8:42329214-42330913:+	0.0271673	0.309205927	0.28742139
3476	POLB	ENSG00000070501	8:42338685-42339011:+	0.0002419	0.03942724	0.056470187
3477	POLB	ENSG00000070501	8:42339069-42344952:+	0.00202401	0.03863222	0.058286597

S/N	Gene	Ensembl ID	Position of retained intron	p-value	AD_IR_average	Cont_IR_average
3478	POLB	ENSG00000070501	8:42350065-42352518:+	0.0275675	0.119760955	0.109767205
3479	CSPP1	ENSG00000104218	8:67154136-67158446:+	0.0053257	0.18321066	0.141778356
3480	CSPP1	ENSG00000104218	8:67193602-67195381:+	0.00362766	0.197437037	0.162447588
3481	TRAM1	ENSG00000067167	8:70575005-70583163:-	7.43E-05	0.094595748	0.055850744
3482	TERF1	ENSG00000147601	8:73027052-73032041:+	2.73E-07	0.057571422	0.07402893
3483	ZFAND1	ENSG00000104231	8:81714039-81714803:-	0.00466366	0.082317689	0.118976599
3484	ZFAND1	ENSG00000104231	8:81714895-81714986:-	9.96E-05	0.093273914	0.055492968
3485	C8orf59	ENSG00000176731	8:85214631-85214920:-	0.02927622	0.153508234	0.135091319
3486	RMDN1	ENSG00000176623	8:86477324-86478922:-	0.00338117	0.301067762	0.234790106
3487	RMDN1	ENSG00000176623	8:86480332-86484871:-	0.0472105	0.099228157	0.084958838
3488	FAM92A	ENSG00000188343	8:93708016-93709770:+	0.00027079	0.101184491	0.069365318
3489	FAM92A	ENSG00000188343	8:93718788-93726393:+	6.19E-09	0.168295054	0.108560662
3490	FAM92A	ENSG00000188343	8:93726513-93728204:+	2.80E-06	0.161576961	0.111493364
3491	INTS8	ENSG00000164941	8:94867196-94867275:+	1.78E-06	0.103801595	0.068048847
3492	INTS8	ENSG00000164941	8:94876147-94876220:+	0.00069551	0.109821587	0.088864652
3493	MTERF3	ENSG00000156469	8:96257114-96258356:-	0.04930819	0.105156051	0.116207797
3494	TBC1D31	ENSG00000156787	8:123150128-123151805:+	5.27E-05	0.125781112	0.088862114
3495	C8orf76	ENSG00000189376	8:123239144-123241229:-	0.02304686	0.111515391	0.096630821
3496	PTK2	ENSG00000169398	8:140668424-140669726:-	0.00496312	0.083975691	0.103661757
3497	PTK2	ENSG00000169398	8:140744767-140746759:-	0.01020517	0.111097123	0.164831227
3498	ADGRB1	ENSG00000181790	8:142522710-142524237:+	0.00039724	0.108426798	0.063908051
3499	ADGRB1	ENSG00000181790	8:142543708-142544219:+	0.008395	0.125820595	0.089207021
3500	ZC3H3	ENSG00000014164	8:143438087-143440040:-	6.69E-06	0.277093073	0.204455266
3501	ZC3H3	ENSG00000014164	8:143468540-143468616:-	0.00794175	0.124915755	0.096650673
3502	NAPRT	ENSG00000147813	8:143575345-143575422:-	0.0093171	0.202368778	0.163295761
3503	TSTA3	ENSG00000104522	8:143613603-143613750:-	4.70E-08	0.196618756	0.125984701
3504	TSTA3	ENSG00000104522	8:143614228-143614319:-	0.00149152	0.164979812	0.130686539
3505	TSTA3	ENSG00000104522	8:143614697-143614786:-	0.00078239	0.207633735	0.161697688
3506	TSTA3	ENSG00000104522	8:143614915-143616105:-	0.0127407	0.181843371	0.154673897
3507	SCRIB	ENSG00000180900	8:143791440-143791665:-	0.00975549	0.236641529	0.18466454
3508	SCRIB	ENSG00000180900	8:143791740-143791875:-	0.01581804	0.223647455	0.175637477
3509	SCRIB	ENSG00000180900	8:143791913-143791990:-	0.00010904	0.108374503	0.064890547
3510	SCRIB	ENSG00000180900	8:143793083-143793899:-	0.00090919	0.103099515	0.130934168
3511	PUF60	ENSG00000179950	8:143818534-143821596:-	9.56E-05	0.058272607	0.072782423
3512	NRBP2	ENSG00000185189	8:143836030-143836126:-	0.02841739	0.177976412	0.137634062
3513	NRBP2	ENSG00000185189	8:143839195-143839313:-	0.00034523	0.160273957	0.114140212
3514	NRBP2	ENSG00000185189	8:143839825-143839928:-	0.00036502	0.187260657	0.133489577
3515	PARP10	ENSG00000178685	8:143984431-143984543:-	0.02426872	0.077390716	0.099439768
3516	GPAA1	ENSG00000197858	8:144083303-144083388:+	0.00313761	0.107251033	0.083334118
3517	GPAA1	ENSG00000197858	8:144083500-144083713:+	0.02277449	0.495955037	0.455512169
3518	GPAA1	ENSG00000197858	8:144084875-144085042:+	0.04596745	0.257116049	0.223785075
3519	SHARPIN	ENSG00000179526	8:144098788-144098865:-	0.02689863	0.233924354	0.199654027
3520	SHARPIN	ENSG00000179526	8:144098994-144099080:-	7.80E-05	0.173250167	0.120151518
3521	SHARPIN	ENSG00000179526	8:144103225-144103552:-	0.0001426	0.130184883	0.093003913
3522	MROH1	ENSG00000179832	8:144192401-144199121:+	0.00177613	0.07541486	0.119234597
3523	MROH1	ENSG00000179832	8:144247436-144247566:+	0.00178263	0.094538385	0.052892298
3524	MROH1	ENSG00000179832	8:144254978-144255508:+	0.01901021	0.108158912	0.081273315
3525	MROH1	ENSG00000179832	8:144255705-144258776:+	0.00217082	0.171172576	0.127309582
3526	MROH1	ENSG00000179832	8:144259354-144259910:+	0.0037154	0.199990474	0.145768488
3527	MROH1	ENSG00000179832	8:144260057-144260185:+	9.28E-06	0.176758451	0.112814891
3528	MROH1	ENSG00000179832	8:144260374-144260676:+	2.09E-06	0.480519549	0.372447844
3529	MROH1	ENSG00000179832	8:144260832-144260906:+	0.00120769	0.520605976	0.427055403
3530	MROH1	ENSG00000179832	8:144261041-144261113:+	0.0006	0.174150479	0.12026423
3531	MROH1	ENSG00000179832	8:144261216-144261283:+	1.35E-06	0.225544532	0.125432201
3532	MROH1	ENSG00000179832	8:144261349-144261654:+	5.13E-05	0.306160593	0.21786003
3533	BOP1	ENSG00000261236	8:144262317-144262395:-	0.02415147	0.298894904	0.249989496
3534	HSF1	ENSG00000185122	8:144309014-144309454:+	0.0330164	0.145997122	0.122730352

S/N	Gene	Ensembl ID	Position of retained intron	p-value	AD_IR_average	Cont_IR_average
3535	DGAT1	ENSG00000185000	8:144317109-144317186:-	0.00040836	0.441896976	0.371906247
3536	DGAT1	ENSG00000185000	8:144317445-144317543:-	0.00804831	0.174122355	0.145082006
3537	DGAT1	ENSG00000185000	8:144318362-144318460:-	0.00038173	0.118113702	0.153857472
3538	DGAT1	ENSG00000185000	8:144318566-144318698:-	0.00133835	0.133028174	0.165454388
3539	DGAT1	ENSG00000185000	8:144319068-144321320:-	0.00660706	0.132509084	0.159070583
3540	DGAT1	ENSG00000185000	8:144321408-144326436:-	1.53E-08	0.176557884	0.269571269
3541	CPSF1	ENSG00000071894	8:144395551-144396347:-	1.42E-06	0.631581012	0.722483805
3542	CPSF1	ENSG00000071894	8:144398132-144398301:-	0.03687507	0.128439097	0.097972549
3543	CPSF1	ENSG00000071894	8:144409172-144409288:-	0.03448551	0.187019877	0.15601896
3544	VPS28	ENSG00000160948	8:144423922-144424040:-	0.00486565	0.228337756	0.186254249
3545	VPS28	ENSG00000160948	8:144424132-144424214:-	1.04E-05	0.154225037	0.104271866
3546	VPS28	ENSG00000160948	8:144424268-144424717:-	0.01742822	0.197666122	0.165735331
3547	CYHR1	ENSG00000187954	8:144464612-144464808:-	0.00180172	0.082929644	0.105620368
3548	KIFC2	ENSG00000167702	8:144467779-144467858:+	0.00060248	0.107333263	0.07708201
3549	KIFC2	ENSG00000167702	8:144467987-144468328:+	3.88E-07	0.127890989	0.078316371
3550	KIFC2	ENSG00000167702	8:144468650-144468724:+	3.05E-05	0.127762191	0.08973721
3551	PPP1R16A	ENSG00000160972	8:144490212-144496460:+	0.00448037	0.088289621	0.108964712
3552	PPP1R16A	ENSG00000160972	8:144500391-144500486:+	0.00717735	0.158445962	0.185597673
3553	MFSD3	ENSG00000167700	8:144510722-144510793:+	0.00262898	0.386495537	0.321553351
3554	RECQL4	ENSG00000160957	8:144511555-144511680:-	0.01538713	0.118315115	0.164781175
3555	RECQL4	ENSG00000160957	8:144512067-144512143:-	0.00221076	0.21442311	0.281886423
3556	RECQL4	ENSG00000160957	8:144513480-144513570:-	8.14E-11	0.274571427	0.413470156
3557	RECQL4	ENSG00000160957	8:144513712-144513927:-	0.00677473	0.0695289	0.086868562
3558	LRRC14	ENSG00000160959	8:144520054-144520237:+	0.00057629	0.518340415	0.427901903
3559	LRRC14	ENSG00000160959	8:144520822-144520910:+	0.00036143	0.467454085	0.37108027
3560	LRRC24	ENSG00000254402	8:144524719-144524815:-	0.01874476	0.543634317	0.588395377
3561	ARHGAP39	ENSG00000147799	8:144548489-144555559:-	0.03524882	0.075931335	0.089609473
3562	RPL8	ENSG00000161016	8:144792140-144792244:-	0.01153031	0.015797405	0.012072811
3563	KANK1	ENSG00000107104	9:742405-744490:+	0.00357965	0.113031265	0.088572475
3564	UBAP2	ENSG00000137073	9:33923033-33923185:-	0.00026837	0.279480963	0.213743414
3565	UBAP2	ENSG00000137073	9:33923293-33923378:-	0.00345755	0.129237085	0.10496199
3566	C9orf24	ENSG00000164972	9:34379181-34379645:-	0.01240226	0.231886238	0.176860083
3567	SIGMAR1	ENSG00000147955	9:34637089-34637219:-	0.03092973	0.33539261	0.289262151
3568	GALT	ENSG00000213930	9:34649081-34649409:+	0.02987577	0.379255159	0.339449169
3569	GALT	ENSG00000213930	9:34649564-34650368:+	1.56E-05	0.204339735	0.149566562
3570	IL11RA	ENSG00000137070	9:34655665-34656738:+	0.00368895	0.406269549	0.324529377
3571	IL11RA	ENSG00000137070	9:34656908-34657034:+	0.00285471	0.281389122	0.213943525
3572	RP11-195F	ENSG00000187186	9:34665190-34665313:-	0.00393986	0.224280756	0.178105658
3573	FANCG	ENSG00000221829	9:35075082-35075278:-	0.00494683	0.236737902	0.204757539
3574	PIGO	ENSG00000165282	9:35089221-35089379:-	0.02386294	0.095703817	0.12138439
3575	PIGO	ENSG00000165282	9:35090280-35090465:-	0.04007546	0.060387021	0.074406559
3576	STOML2	ENSG00000165283	9:35100726-35100931:-	0.00887296	0.11050253	0.092086106
3577	RUSC2	ENSG00000198853	9:35560851-35560959:+	0.03126349	0.141632983	0.159590014
3578	TESK1	ENSG00000107140	9:35606285-35606836:+	5.10E-09	0.116922509	0.068624021
3579	TESK1	ENSG00000107140	9:35607409-35607581:+	0.02214643	0.250214756	0.239294835
3580	TESK1	ENSG00000107140	9:35607672-35607927:+	0.0462568	0.105669237	0.094011926
3581	TLN1	ENSG00000137076	9:35704174-35704331:-	0.00867575	0.246596927	0.295463961
3582	GBA2	ENSG00000070610	9:35738152-35738231:-	0.01664192	0.245311037	0.280181442
3583	GBA2	ENSG00000070610	9:35738374-35738525:-	0.00464364	0.30557739	0.354746909
3584	GBA2	ENSG00000070610	9:35738632-35738751:-	0.00028289	0.281384171	0.348004701
3585	NPR2	ENSG00000159899	9:35802607-35802731:+	0.01088539	0.074404754	0.06545021
3586	TMEM8B	ENSG00000137103	9:35834650-35835010:+	0.00517062	0.132171923	0.185156182
3587	TMEM8B	ENSG00000137103	9:35846611-35846816:+	0.00014483	0.10578206	0.066058113
3588	CLTA	ENSG00000122705	9:36204179-36211602:+	0.00369401	0.035830696	0.026565794
3589	CLTA	ENSG00000122705	9:36209320-36211602:+	0.00011925	0.034609863	0.028961005
3590	TRMT10B	ENSG00000165275	9:37770739-37776281:+	0.01083326	0.718496024	0.676560805
3591	TRMT10B	ENSG00000165275	9:37776405-37777600:+	0.00077092	0.724852354	0.661953662

S/N	Gene	Ensembl ID	Position of retained intron	p-value	AD_IR_average	Cont_IR_average
3592	EXOSC3	ENSG00000107371	9:37780880-37781985:-	0.00062634	0.063516167	0.0780741
3593	NMRK1	ENSG00000106733	9:75077580-75083086:-	0.04171145	0.085361857	0.068201808
3594	CTSL	ENSG00000135047	9:87730498-87731007:+	0.00513564	0.12915466	0.151088949
3595	GADD45G	ENSG00000130222	9:89605174-89605431:+	0.00795264	0.090848261	0.087423856
3596	GADD45G	ENSG00000130222	9:89605533-89605666:+	0.02267251	0.070285471	0.066939283
3597	AUH	ENSG00000148090	9:91214425-91216058:-	0.00738883	0.159101329	0.131531175
3598	AUH	ENSG00000148090	9:91216106-91217276:-	0.00024854	0.141311071	0.096507708
3599	SPTLC1	ENSG00000090054	9:92032558-92034809:-	4.03E-06	0.169147424	0.11168324
3600	NOL8	ENSG00000198000	9:92310675-92311145:-	0.0177939	0.205288956	0.194927896
3601	IPPK	ENSG00000127080	9:92616057-92619485:-	0.04100787	0.093764832	0.111579238
3602	CARD19	ENSG00000165233	9:93107816-93110567:+	0.00246189	0.169010372	0.151907455
3603	CARD19	ENSG00000165233	9:93111938-93112217:+	0.03258223	0.565264756	0.56127961
3604	WNK2	ENSG00000165238	9:93300149-93306776:+	0.03687987	0.363501372	0.32063913
3605	WNK2	ENSG00000165238	9:93306821-93308327:+	0.00836195	0.178767209	0.152565151
3606	NCBP1	ENSG00000136937	9:97668974-97669592:+	0.01550307	0.241763268	0.287826455
3607	XPA	ENSG00000136936	9:97687261-97689533:-	0.00794652	0.162233424	0.114653089
3608	ANKS6	ENSG00000165138	9:98782573-98783952:-	0.00382365	0.080584949	0.060309004
3609	ANKS6	ENSG00000165138	9:98784876-98790103:-	0.02759048	0.450649515	0.384546431
3610	TEX10	ENSG00000136891	9:100302304-100303631:-	1.62E-14	0.284790705	0.183483014
3611	TEX10	ENSG00000136891	9:100308681-100310298:-	4.44E-15	0.214141083	0.121608678
3612	TEX10	ENSG00000136891	9:100310379-100320264:-	0.00011593	0.231656506	0.184215006
3613	CTNNAL1	ENSG00000119326	9:108944018-108948185:-	0.00779309	0.095630588	0.075119689
3614	KIAA0368	ENSG00000136813	9:111383332-111384521:-	0.00188988	0.163255587	0.133978043
3615	KIAA0368	ENSG00000136813	9:111384569-111385336:-	0.00048767	0.12035099	0.079838221
3616	ALAD	ENSG00000148218	9:113390676-113390797:-	0.00495826	0.54497311	0.49650404
3617	ALAD	ENSG00000148218	9:113391623-113392118:-	0.0070871	0.134953378	0.114573138
3618	CDK5RAP2	ENSG00000136861	9:120389787-120394511:-	1.05E-14	0.208061084	0.126188465
3619	CDK5RAP2	ENSG00000136861	9:120403071-120404035:-	0.00606492	0.21997363	0.250759613
3620	CDK5RAP2	ENSG00000136861	9:120407248-120408346:-	0.00086616	0.088298061	0.068937569
3621	CDK5RAP2	ENSG00000136861	9:120439972-120443619:-	0.01189649	0.289911802	0.238197478
3622	FBXW2	ENSG00000119402	9:120776226-120778350:-	0.00577619	0.13090971	0.171806219
3623	PHF19	ENSG00000119403	9:120862005-120862587:-	0.00709182	0.066730513	0.05126865
3624	CNTRL	ENSG00000119397	9:121175224-121177162:+	0.001151	0.158249351	0.121711914
3625	DAB2IP	ENSG00000136848	9:121776391-121781463:+	0.00286689	0.102108412	0.078648691
3626	GARNL3	ENSG00000136895	9:127353944-127354293:+	0.03116981	0.092793955	0.083039056
3627	GARNL3	ENSG00000136895	9:127383545-127385026:+	0.00059952	0.222693616	0.177063753
3628	GARNL3	ENSG00000136895	9:127387331-127388903:+	2.08E-07	0.111642946	0.074236545
3629	SLC2A8	ENSG00000136856	9:127404067-127404817:+	0.02394269	0.147396309	0.122704381
3630	SLC2A8	ENSG00000136856	9:127404991-127405419:+	0.00017208	0.26105109	0.20109399
3631	SLC2A8	ENSG00000136856	9:127405565-127407111:+	0.00017429	0.104393802	0.081988642
3632	LRSAM1	ENSG00000148356	9:127451669-127451928:+	0.0092784	0.417986744	0.368616974
3633	LRSAM1	ENSG00000148356	9:127452084-127454495:+	0.017445	0.152031721	0.132712888
3634	LRSAM1	ENSG00000148356	9:127492897-127495319:+	2.36E-08	0.284074355	0.207745403
3635	LRSAM1	ENSG00000148356	9:127495418-127495963:+	3.16E-10	0.29802372	0.208827284
3636	LRSAM1	ENSG00000148356	9:127496095-127497252:+	3.42E-06	0.135484016	0.095248255
3637	LRSAM1	ENSG00000148356	9:127497334-127501009:+	3.46E-05	0.18421963	0.136694397
3638	FAM129B	ENSG00000136830	9:127509131-127510145:-	0.01124312	0.101277513	0.081807496
3639	SH2D3C	ENSG00000095370	9:127738921-127739681:-	0.04744687	0.128944663	0.110009873
3640	SH2D3C	ENSG00000095370	9:127739888-127740257:-	0.01140288	0.100989935	0.078854343
3641	SH2D3C	ENSG00000095370	9:127740369-127741787:-	0.00109481	0.154482459	0.115947817
3642	FPGS	ENSG00000136877	9:127808311-127808557:+	0.00918586	0.072053998	0.052477767
3643	FPGS	ENSG00000136877	9:127808889-127809683:+	0.01808012	0.140147977	0.103021631
3644	ENG	ENSG00000106991	9:127820037-127824303:-	0.00141636	0.093787916	0.154573627
3645	ST6GALNA	ENSG00000136840	9:127908581-127909950:-	0.00620849	0.236821451	0.210218145
3646	ST6GALNA	ENSG00000136840	9:127910058-127912267:-	1.11E-05	0.160602672	0.133446119
3647	DPM2	ENSG00000136908	9:127936655-127937433:-	3.75E-07	0.482543683	0.38670874
3648	DPM2	ENSG00000136908	9:127937523-127937817:-	4.32E-07	0.456372707	0.349251545

S/N	Gene	Ensembl ID	Position of retained intron	p-value	AD_IR_average	Cont_IR_average
3649	FAM102A	ENSG00000167106	9:127948225-127948367:-	0.02160436	0.153360628	0.139508406
3650	FAM102A	ENSG00000167106	9:127953604-127953804:-	0.04962149	0.104015501	0.084161823
3651	CIZ1	ENSG00000148337	9:128178490-128178708:-	1.25E-05	0.076838524	0.109824216
3652	DNM1	ENSG00000106976	9:128247935-128248582:+	0.01128854	0.093115091	0.077659916
3653	GOLGA2	ENSG00000167110	9:128262704-128263033:-	0.01712739	0.195973541	0.169894565
3654	GOLGA2	ENSG00000167110	9:128265881-128265969:-	0.00798138	0.099314772	0.144997271
3655	URM1	ENSG00000167118	9:128389309-128389665:+	0.0047128	0.217834011	0.180830682
3656	CERCAM	ENSG00000167123	9:128422978-128423145:+	0.00051049	0.21145101	0.150966165
3657	CERCAM	ENSG00000167123	9:128423263-128424137:+	8.04E-05	0.136485716	0.097964288
3658	PKN3	ENSG00000160447	9:128713642-128714045:+	0.03269095	0.091512517	0.072151748
3659	ZER1	ENSG00000160445	9:128735431-128739930:-	0.00011024	0.222546512	0.185468208
3660	ENDOG	ENSG00000167136	9:128820848-128822327:+	4.14E-06	0.450016439	0.366098065
3661	SPOUT1	ENSG00000198917	9:128826152-128826383:-	0.00024378	0.558825159	0.489040584
3662	KYAT1	ENSG00000171097	9:128833643-128833739:-	1.08E-07	0.16001219	0.225315588
3663	KYAT1	ENSG00000171097	9:128835402-128835480:-	0.00010373	0.108174459	0.077366905
3664	NUP188	ENSG00000095319	9:128993403-128993524:+	0.0014821	0.159523932	0.226642976
3665	NUP188	ENSG00000095319	9:128994442-128994855:+	0.00046857	0.180940932	0.248484379
3666	NUP188	ENSG00000095319	9:128999805-129001528:+	0.01797267	0.189001552	0.161592165
3667	NUP188	ENSG00000095319	9:129001976-129002816:+	0.04028288	0.165071599	0.141494157
3668	NUP188	ENSG00000095319	9:129005530-129005644:+	0.01831978	0.330785951	0.289827439
3669	NUP188	ENSG00000095319	9:129005776-129006049:+	0.0017243	0.345247829	0.299200234
3670	NUP188	ENSG00000095319	9:129006123-129006238:+	0.00221753	0.29372889	0.247824927
3671	NUP188	ENSG00000095319	9:129006368-129006501:+	0.00491017	0.281658857	0.239982888
3672	SH3GLB2	ENSG00000148341	9:129008791-129009105:-	0.00011138	0.21842601	0.157228417
3673	SH3GLB2	ENSG00000148341	9:129009346-129009770:-	5.43E-05	0.066241368	0.040989771
3674	MIGA2	ENSG00000148343	9:129069129-129069848:+	0.04058813	0.303486646	0.265876805
3675	DOLPP1	ENSG00000167130	9:129085107-129085206:+	0.0207057	0.124994427	0.101986422
3676	PTPA	ENSG00000119383	9:129140151-129142444:+	0.00038043	0.015276563	0.010806343
3677	USP20	ENSG00000136878	9:129874756-129874828:+	0.03075371	0.18072567	0.154614492
3678	FNBP1	ENSG00000187239	9:129890546-129895837:-	0.00646629	0.128141366	0.158889065
3679	FNBP1	ENSG00000187239	9:129915980-129923843:-	0.00294531	0.206325544	0.243991483
3680	FUBP3	ENSG00000107164	9:130613027-130614287:+	0.00617702	0.098796321	0.069579074
3681	FUBP3	ENSG00000107164	9:130614345-130616354:+	0.03546129	0.132348141	0.103701968
3682	FUBP3	ENSG00000107164	9:130632278-130634666:+	0.00582586	0.182486644	0.151911382
3683	EXOSC2	ENSG00000130713	9:130698251-130699328:+	0.04223933	0.109788644	0.099758671
3684	EXOSC2	ENSG00000130713	9:130700935-130702133:+	0.00204125	0.546682768	0.495157922
3685	EXOSC2	ENSG00000130713	9:130702310-130703052:+	0.00898473	0.101916344	0.124804735
3686	POMT1	ENSG00000130714	9:131504340-131506113:+	6.00E-05	0.20209854	0.27339507
3687	POMT1	ENSG00000130714	9:131509022-131509742:+	0.03456507	0.130911051	0.159067
3688	UCK1	ENSG00000130717	9:131525977-131528943:-	0.00014958	0.100015072	0.074282582
3689	MED27	ENSG00000160563	9:131863140-131884057:-	8.31E-05	0.099926762	0.070260044
3690	NTNG2	ENSG00000196358	9:132239271-132240909:+	2.68E-05	0.109900634	0.069159242
3691	TSC1	ENSG00000165699	9:132904454-132905580:-	0.04359566	0.144328004	0.124527465
3692	RALGDS	ENSG00000160271	9:133101762-133101937:-	0.0138403	0.184991857	0.215202732
3693	MED22	ENSG00000148297	9:133345252-133346539:-	0.01869121	0.166868284	0.142125568
3694	SURF1	ENSG00000148290	9:133354957-133356268:-	0.02020325	0.085442044	0.070105012
3695	LINC00094	ENSG00000235106	9:134026090-134026207:+	0.00061185	0.12936006	0.176458374
3696	WDR5	ENSG00000196363	9:134154541-134155339:+	0.03408932	0.137141637	0.117925419
3697	WDR5	ENSG00000196363	9:134156593-134157892:+	0.00130851	0.303915488	0.260559247
3698	QSOX2	ENSG00000165661	9:136219164-136221795:-	0.00513278	0.089344455	0.131053947
3699	GPSM1	ENSG00000160360	9:136348767-136349586:+	0.03867627	0.112396221	0.128308091
3700	SNAPC4	ENSG00000165684	9:136392594-136392672:-	0.01326416	0.191781104	0.147152399
3701	SDCCAG3	ENSG00000165689	9:136404693-136405090:-	0.00889982	0.471932963	0.43175239
3702	PMPCA	ENSG00000165688	9:136416391-136416950:+	0.01052519	0.305473927	0.268070974
3703	PMPCA	ENSG00000165688	9:136421976-136423094:+	0.01438316	0.090623533	0.105461186
3704	INPP5E	ENSG00000148384	9:136429807-136430276:-	0.03573392	0.150303743	0.128298973
3705	INPP5E	ENSG00000148384	9:136430413-136431001:-	0.04892375	0.263653634	0.230900042

S/N	Gene	Ensembl ID	Position of retained intron	p-value	AD_IR_average	Cont_IR_average
3706	INPP5E	ENSG00000148384	9:136432586-136432955:-	0.02687827	0.311608399	0.26710579
3707	INPP5E	ENSG00000148384	9:136433075-136433154:-	0.00568824	0.277774348	0.224015693
3708	SEC16A	ENSG00000148396	9:136445719-136446854:-	0.02709495	0.103808278	0.087946171
3709	SEC16A	ENSG00000148396	9:136447680-136447852:-	0.00555915	0.079349238	0.10844637
3710	SEC16A	ENSG00000148396	9:136447909-136448083:-	0.00560834	0.078318388	0.10429986
3711	SEC16A	ENSG00000148396	9:136455793-136456052:-	0.01190562	0.136751394	0.178718697
3712	PHPT1	ENSG00000054148	9:136850560-136850754:+	0.00655322	0.149891968	0.134409748
3713	MAMDC4	ENSG00000177943	9:136857586-136857658:+	0.02217494	0.498554317	0.410533935
3714	MAMDC4	ENSG00000177943	9:136859317-136859885:+	0.00219525	0.356887437	0.272791036
3715	MAMDC4	ENSG00000177943	9:136860064-136860561:+	0.0242077	0.166823771	0.122445984
3716	FBXW5	ENSG00000159069	9:136942466-136942546:-	0.04612492	0.131825412	0.146239755
3717	PTGDS	ENSG00000107317	9:136979299-136979945:+	0.02367459	0.077411029	0.08440249
3718	C9orf142	ENSG00000148362	9:136992562-136992639:+	0.03152944	0.344283472	0.295554457
3719	ABCA2	ENSG00000107331	9:137016471-137016573:-	0.00190269	0.060813467	0.0899153
3720	ABCA2	ENSG00000107331	9:137019306-137020335:-	0.02907226	0.101129628	0.135249075
3721	ABCA2	ENSG00000107331	9:137023052-137024142:-	0.03487939	0.106003276	0.128505767
3722	NPDC1	ENSG00000107281	9:137040436-137040513:-	3.23E-05	0.098196824	0.068920197
3723	NPDC1	ENSG00000107281	9:137040598-137040670:-	0.00058301	0.133493223	0.099904643
3724	NPDC1	ENSG00000107281	9:137040737-137040813:-	0.01510949	0.096996163	0.077529281
3725	NPDC1	ENSG00000107281	9:137040984-137041061:-	0.00225532	0.116955516	0.090823291
3726	MAN1B1	ENSG00000177239	9:137106315-137106688:+	0.00212087	0.191095495	0.144827784
3727	DPP7	ENSG00000176978	9:137112805-137112952:-	5.49E-11	0.338775671	0.193232439
3728	DPP7	ENSG00000176978	9:137113287-137113360:-	0.00322894	0.30213589	0.242547106
3729	DPP7	ENSG00000176978	9:137114576-137114646:-	0.03619499	0.494715671	0.451788922
3730	ANAPC2	ENSG00000176248	9:137175472-137175707:-	0.0458847	0.140505748	0.154172174
3731	ANAPC2	ENSG00000176248	9:137180384-137180451:-	5.95E-05	0.183955507	0.116095677
3732	ANAPC2	ENSG00000176248	9:137183791-137184912:-	0.01822132	0.16604535	0.130506069
3733	ANAPC2	ENSG00000176248	9:137186356-137187480:-	0.00285276	0.295842207	0.352419857
3734	SSNA1	ENSG00000176101	9:137188778-137189065:+	3.07E-06	0.598372122	0.487775377
3735	TPRN	ENSG00000176058	9:137192691-137198986:-	0.02121215	0.222981896	0.185947935
3736	NDOR1	ENSG00000188566	9:137214691-137214797:+	0.02118723	0.070052027	0.083500806
3737	NDOR1	ENSG00000188566	9:137215018-137215094:+	0.04813247	0.455177439	0.403812104
3738	PNPLA7	ENSG00000130653	9:137462331-137462684:-	0.03094701	0.2728208	0.189781023
3739	PNPLA7	ENSG00000130653	9:137462833-137463414:-	1.94E-05	0.294890898	0.165002497
3740	ZMYND19	ENSG00000165724	9:137587107-137587716:-	0.00894307	0.321724756	0.278602969
3741	ARRDC1	ENSG00000197070	9:137613510-137613614:+	4.73E-08	0.579854232	0.468808597
3742	ARRDC1	ENSG00000197070	9:137614214-137614298:+	8.26E-08	0.47656822	0.361768697
3743	ARRDC1	ENSG00000197070	9:137614475-137614558:+	2.18E-06	0.486630927	0.386796188
3744	EHMT1	ENSG00000181090	9:137814508-137815946:+	0.00514254	0.420072902	0.368017935
3745	GTPBP6	ENSG00000178605	X:307512-307731:-	0.03259025	0.147394805	0.126587435
3746	GTPBP6	ENSG00000178605	X:311627-312765:-	0.00734364	0.528774878	0.483288338
3747	GTPBP6	ENSG00000178605	X:312924-314149:-	0.04013662	0.114439091	0.096668765
3748	GTPBP6	ENSG00000178605	X:315299-316913:-	0.00282784	0.149674977	0.118006716
3749	PPP2R3B	ENSG00000167393	X:338710-338777:-	0.01504284	0.212934571	0.170029631
3750	ASMTL	ENSG00000169093	X:1412854-1417972:-	0.00399034	0.183263557	0.135837561
3751	ASMTL	ENSG00000169093	X:1418116-1418981:-	0.04810316	0.178252408	0.143528583
3752	ASMTL	ENSG00000169093	X:1419114-1421657:-	0.00285535	0.141351465	0.102345664
3753	OFD1	ENSG00000046651	X:13767284-13768053:+	2.62E-06	0.101020756	0.072190267
3754	CTPS2	ENSG00000047230	X:16589775-16590751:-	0.03567332	0.120218541	0.095110202
3755	PHKA2	ENSG00000044446	X:18907097-18907899:-	0.03156635	0.115641985	0.091180038
3756	MED14	ENSG00000180182	X:40651879-40654363:-	0.00028591	0.193159889	0.129087056
3757	RGN	ENSG00000130988	X:47089991-47091677:+	0.00325952	0.12693136	0.172058806
3758	NDUFB11	ENSG00000147123	X:47142440-47142583:-	0.00088762	0.040610744	0.030980473
3759	RBM10	ENSG00000182872	X:47180037-47180211:+	0.04437859	0.130812068	0.104298923
3760	UBA1	ENSG00000130985	X:47194024-47198802:+	0.02016378	0.115057289	0.152153383
3761	CDK16	ENSG00000102225	X:47226027-47226278:+	5.52E-09	0.416201398	0.294774948
3762	ARAF	ENSG00000078061	X:47568894-47568986:+	0.00032571	0.137186363	0.094670246

S/N	Gene	Ensembl ID	Position of retained intron	p-value	AD_IR_average	Cont_IR_average
3763	FTSJ1	ENSG00000068438	X:48478518-48478616:+	0.02734566	0.20138891	0.185306838
3764	FTSJ1	ENSG00000068438	X:48481528-48481631:+	0.00033416	0.33072228	0.269659473
3765	FTSJ1	ENSG00000068438	X:48481715-48482402:+	0.0141897	0.172388774	0.204092899
3766	FTSJ1	ENSG00000068438	X:48483027-48485735:+	0.0257482	0.148221146	0.125989844
3767	PORCN	ENSG00000102312	X:48512507-48512588:+	0.02855446	0.10573653	0.085068468
3768	PORCN	ENSG00000102312	X:48514365-48514524:+	1.72E-06	0.185607095	0.145400881
3769	RBM3	ENSG00000102317	X:48576083-48576313:+	2.36E-08	0.244689006	0.263099851
3770	RBM3	ENSG00000102317	X:48577109-48577464:+	1.88E-08	0.39009151	0.214435587
3771	WDR13	ENSG00000101940	X:48597614-48597957:+	0.00368609	0.664162329	0.614530714
3772	WDR13	ENSG00000101940	X:48600626-48601783:+	2.31E-09	0.14888661	0.099630129
3773	HDAC6	ENSG00000094631	X:48815642-48815883:+	0.00031058	0.370669	0.310687901
3774	HDAC6	ENSG00000094631	X:48816269-48816464:+	0.00038469	0.095867545	0.069930935
3775	HDAC6	ENSG00000094631	X:48816633-48817325:+	0.01090474	0.085478345	0.1121768
3776	HDAC6	ENSG00000094631	X:48817459-48818040:+	0.03996325	0.106032865	0.132629925
3777	PQBP1	ENSG00000102103	X:48902517-48902731:+	0.03225525	0.135319106	0.154906327
3778	TFE3	ENSG00000068323	X:49034251-49038009:-	0.0015269	0.10785286	0.092292908
3779	WDR45	ENSG00000196998	X:49076524-49076644:-	7.16E-08	0.129485283	0.085349832
3780	WDR45	ENSG00000196998	X:49077747-49077836:-	0.01570678	0.246317551	0.212975578
3781	PLP2	ENSG00000102007	X:49172096-49173128:+	0.00356055	0.17309907	0.133711334
3782	PLP2	ENSG00000102007	X:49173281-49173387:+	0.03928486	0.120141062	0.096149978
3783	CCDC22	ENSG00000101997	X:49237263-49242015:+	6.11E-06	0.054921056	0.087385143
3784	CCDC22	ENSG00000101997	X:49242148-49242885:+	0.00967344	0.191212217	0.233078348
3785	CCDC22	ENSG00000101997	X:49246925-49247495:+	0.00106648	0.212997151	0.165424097
3786	CCDC22	ENSG00000101997	X:49248924-49249166:+	0.00017005	0.106756487	0.07270897
3787	CCDC22	ENSG00000101997	X:49249262-49249508:+	0.00037621	0.112235578	0.075109846
3788	TSPYL2	ENSG00000184205	X:53083305-53084544:+	0.02817203	0.055250473	0.045779458
3789	TSPYL2	ENSG00000184205	X:53084622-53084754:+	0.00717065	0.246897415	0.199768161
3790	KDM5C	ENSG00000126012	X:53193332-53193436:-	0.00026545	0.065996124	0.085858247
3791	IQSEC2	ENSG00000124313	X:53248236-53248720:-	5.45E-05	0.104797863	0.068597956
3792	FGD1	ENSG00000102302	X:54448967-54449142:-	0.00157807	0.29748978	0.238255116
3793	FGD1	ENSG00000102302	X:54449760-54450270:-	0.00302122	0.35468378	0.298349462
3794	MAGED2	ENSG00000102316	X:54815690-54815880:+	1.45E-05	0.300957551	0.238875403
3795	TRO	ENSG00000067445	X:54924733-54924988:+	3.54E-06	0.110370082	0.063849324
3796	TRO	ENSG00000067445	X:54925068-54925591:+	1.63E-06	0.129903402	0.071989843
3797	TRO	ENSG00000067445	X:54925683-54926409:+	3.61E-07	0.147648618	0.082319534
3798	TRO	ENSG00000067445	X:54927105-54927666:+	5.28E-05	0.09593726	0.068322593
3799	TRO	ENSG00000067445	X:54928710-54930740:+	2.66E-08	0.69740178	0.555221714
3800	TRO	ENSG00000067445	X:54931031-54931203:+	0.00513117	0.165502632	0.190365797
3801	RRAGB	ENSG00000083750	X:55757331-55758245:+	4.63E-07	0.20000034	0.128329638
3802	ZMYM3	ENSG00000147130	X:71242424-71242969:-	0.00019184	0.190432037	0.147207932
3803	TAF1	ENSG00000147133	X:71460803-71463823:+	0.0008538	0.083425894	0.11613781
3804	CSTF2	ENSG00000101811	X:100837449-100838238:+	0.00871709	0.097345323	0.132724632
3805	BEX4	ENSG00000102409	X:103215238-103216148:+	4.62E-05	0.044481222	0.05014503
3806	TCEAL9	ENSG00000185222	X:103356606-103357614:+	0.0357247	0.049117232	0.054790499
3807	MORF4L2	ENSG00000123562	X:103685260-103687988:-	6.21E-08	0.041215934	0.034189455
3808	TSC22D3	ENSG00000157514	X:107715950-107716314:-	3.51E-06	0.098854299	0.107162562
3809	THOC2	ENSG00000125676	X:123610957-123611439:-	0.00082165	0.041252913	0.031825796
3810	SMARCA1	ENSG00000102038	X:129447233-129448332:-	2.60E-09	0.257959817	0.163688381
3811	FAM127B	ENSG00000203950	X:135051368-135051916:-	0.0139356	0.673104427	0.632895584
3812	PNCK	ENSG00000130822	X:153671190-153671284:-	0.00513704	0.339739366	0.263222758
3813	SLC6A8	ENSG00000130821	X:153688836-153690374:+	0.04513086	0.121661523	0.101538064
3814	PLXNB3	ENSG00000198753	X:153773016-153773229:+	0.00659203	0.182031899	0.131701233
3815	PLXNB3	ENSG00000198753	X:153773406-153773517:+	0.02345485	0.381148993	0.317012687
3816	PLXNB3	ENSG00000198753	X:153774571-153774705:+	0.04743076	0.116044683	0.129028492
3817	PLXNB3	ENSG00000198753	X:153775660-153775886:+	0.03432874	0.166961971	0.131052618
3818	PLXNB3	ENSG00000198753	X:153776980-153777207:+	0.0055017	0.295470909	0.227827757
3819	PLXNB3	ENSG00000198753	X:153777688-153777947:+	9.81E-05	0.261863732	0.202993821

S/N	Gene	Ensembl ID	Position of retained intron	p-value	AD_IR_average	Cont_IR_average
3820	PLXNB3	ENSG00000198753	X:153778471-153778599:+	8.84E-06	0.277296317	0.191960186
3821	SRPK3	ENSG00000184343	X:153784213-153784293:+	0.00017812	0.456293265	0.355235974
3822	SRPK3	ENSG00000184343	X:153785003-153785080:+	0.03249124	0.092066855	0.122834608
3823	PDZD4	ENSG00000067840	X:153806840-153807278:-	0.01633329	0.462306541	0.40524884
3824	NAA10	ENSG00000102030	X:153932115-153932315:-	0.03237201	0.405056744	0.357052701
3825	IRAK1	ENSG00000184216	X:154016097-154016436:-	0.00102974	0.11155806	0.082915909
3826	FLNA	ENSG00000196924	X:154351041-154351580:-	0.00614319	0.595458524	0.539563416
3827	EMD	ENSG00000102119	X:154380019-154380233:+	0.03617229	0.365734646	0.315765558
3828	TAZ	ENSG00000102125	X:154412214-154413206:+	1.86E-07	0.572852317	0.436713948
3829	TAZ	ENSG00000102125	X:154413567-154419542:+	2.01E-11	0.12639105	0.199773639
3830	TAZ	ENSG00000102125	X:154419623-154419704:+	0.00021357	0.680224768	0.719615403
3831	TAZ	ENSG00000102125	X:154419746-154420031:+	0.00037637	0.495881524	0.549126494
3832	FAM3A	ENSG00000071889	X:154507329-154507405:-	0.03091297	0.159639351	0.136124656
3833	FAM3A	ENSG00000071889	X:154515812-154515909:-	3.48E-05	0.598528988	0.683462221
