# Supplementary material for: Increased intron retention is a post‐transcriptional signature associated with progressive aging and Alzheimer’s disease
Source: Aging Cell. 2019 Mar 13;18(3):e12928. doi: 10.1111/acel.12928 (PMC6516162; doi:10.1111/acel.12928)
Supplement: Supplementary file 9 [file ACEL-18-e12928-s009.pdf]

Table S8: DAVID functional annotation chart of differential IR genes between Control & AD Frontal Cortex (UOK: SRS373308)

GOTERM_BP_DIRECT	Count	%	*P-Value*	Fold Enrichment	Benjamini value
GO:0006406~mRNA export from nucleus	16	2.053915	1.84E-05	3.789449929	0.047715216
GO:0000398~mRNA splicing, via spliceosome	25	3.209243	2.33E-05	2.667124106	0.030418952
GO:0016569~covalent chromatin modification	16	2.053915	7.93E-05	3.353495513	0.067742598
GO:0030866~cortical actin cytoskeleton organization	7	0.898588	3.86E-04	6.907851434	0.225838906
GO:0007020~microtubule nucleation	6	0.770218	7.12E-04	7.894687353	0.314726949
GO:0043248~proteasome assembly	5	0.641849	0.001183	9.868359191	0.407506086
GO:0006281~DNA repair	21	2.695764	0.002322	2.116448099	0.585742021
GO:0046907~intracellular transport	6	0.770218	0.002863	5.921015515	0.613727042
GO:0000226~microtubule cytoskeleton organization	10	1.283697	0.002877	3.335783389	0.572437246
GO:0006338~chromatin remodeling	11	1.412067	0.003277	3.029356775	0.581516298
GO:0031124~mRNA 3'-end processing	8	1.026958	0.004737	3.789449929	0.681953495
GO:2001184~positive regulation of interleukin-12 secretion	3	0.385109	0.005177	23.68406206	0.682688197
GO:0006418~tRNA aminoacylation for protein translation	7	0.898588	0.006208	4.14471086	0.719562987
GO:0034063~stress granule assembly	4	0.513479	0.007171	9.473624824	0.744430168
GO:0006396~RNA processing	11	1.412067	0.007714	2.685821471	0.745938991
GO:0006887~exocytosis	10	1.283697	0.008139	2.853501453	0.742184208
GO:0032206~positive regulation of telomere maintenance	4	0.513479	0.009554	8.612386203	0.776581093
GO:0032508~DNA duplex unwinding	7	0.898588	0.009909	3.767918964	0.769701865
GO:0006886~intracellular protein transport	19	2.439024	0.01129	1.906767708	0.795261457
GO:0006370~7-methylguanosine mRNA capping	6	0.770218	0.011706	4.306193102	0.790406351

Table S8: Pathway analysis of differential IR genes between Control & AD Frontal Cortex (UOK: SRS373308)

ID	Pathway Name	#Gene	*P* -value	FDR
P00015	Circadian clock system	4	7.24E-04	8.18E-02
P02756	N-acetylglucosamine metabolism	2	2.80E-02	8.00E-01
hsa04710	Circadian rhythm - Homo sapiens (human)	7	3.89E-04	1.18E-01
hsa03015	mRNA surveillance pathway - Homo sapiens (human)	11	2.68E-03	2.16E-01
hsa03040	Spliceosome - Homo sapiens (human)	14	2.82E-03	2.16E-01
hsa00970	Aminoacyl-tRNA biosynthesis - Homo sapiens (human)	7	3.41E-03	2.16E-01
hsa04530	Tight junction - Homo sapiens (human)	14	4.22E-03	2.16E-01
hsa00563	Glycosylphosphatidylinositol (GPI)-anchor biosynthesis - Homo sapiens (human)	5	4.76E-03	2.16E-01
hsa04961	Endocrine and other factor-regulated calcium reabsorption - Homo sapiens (human)	7	4.99E-03	2.16E-01
hsa04120	Ubiquitin mediated proteolysis - Homo sapiens (human)	13	9.38E-03	3.55E-01
hsa05169	Epstein-Barr virus infection - Homo sapiens (human)	17	1.15E-02	3.87E-01
hsa04912	GnRH signaling pathway - Homo sapiens (human)	9	2.37E-02	6.37E-01

Table S8: Overlap of differential IR genes with curated AD genes (DisGeNET)					
ABCA2	CLUH	HARS	NR4A2	RENBP	WASF1
ABCB6	COG1	HGS	PCBP4	RPS6KB1	XRCC1
ACADVL	COL11A2	HLA-B	PCNT	RRP1	ZMYM3
ACO2	CRHR1	HM13	PDE7A	SGSM3	
AKAP9	CRTC1	HMGA1	PER2	SHANK3	
ALS2	CSNK1D	HMGCS1	PHF1	SLC25A27	
APC2	CYP27A1	HNRNPA2B1	PIK3R1	SLC25A38	
APLP1	DDX39B	HTRA2	PKP4	SLC30A3	
ARRB2	DGAT1	IDUA	PLA2G6	SPG7	
ATAT1	DLG1	IKBKB	PLD2	SPPL2B	
BECN1	DYSF	KIDINS220	PPARD	SREBF2	
BIN1	EIF2AK2	L1CAM	PPP2R2B	SRSF3	
BRD2	EIF2S3	LAMP2	PRKRA	STIM2	
BRD8	ELAC2	MAPK14	PRPF31	STK11	
BRSK1	ETS2	MAPT	PSMB8	SYNJ1	
CACNA1A	FLOT1	MCOLN1	PTGDS	TFEB	
CALB1	GABBR1	MPHOSPH6	PTH1R	TOMM40	
CD47	GCA	NAE1	RAF1	UBR5	
CDC25B	GGA3	NCAPH2	RANBP9	VAMP2	
CLOCK	GOLGA2	NELFE	RELN	VCAN	

Table S8: Differential IR between Control & AD Frontal Cortex (UOK: SRS373308)

S/N	Gene Affected	ENSEMBL_ID	Position of retained intron	IR ratio AD	IR ratio Control	*p*- value
1	AARSD1	ENSG00000266967	17:42955224-42955841:-	0.12500004	0.019148967	0.000143
2	AATK	ENSG00000181409	17:81119580-81119935:-	0.10114511	0.023076977	0.013693
3	ABCA2	ENSG00000107331	9:137023052-137024142:-	0.3750001	0.104477761	0.032493
4	ABCA5	ENSG00000154265	17:69248317-69249904:-	0.25000005	0.078886376	0.004513
5	ABCB6	ENSG00000115657	2:219212491-219213007:-	0.21465973	0.087804941	0.029082
6	ABCB7	ENSG00000131269	X:75073776-75073867:-	0.19230779	0.023255926	0.012837
7	ABCE1	ENSG00000164163	4:145120153-145121173:+	0.15183251	0.048110019	0.013762
8	ABCE1	ENSG00000164163	4:145121233-145121332:+	0.25806458	0.050156806	0.000865
9	ABCE1	ENSG00000164163	4:145121391-145123020:+	0.1126761	0.043918961	0.033881
10	ABCF3	ENSG00000161204	3:184186875-184187396:+	0.17045462	0.050387671	0.022563
11	ABCF3	ENSG00000161204	3:184187443-184187663:+	0.30890058	0.103174681	0.007937
12	ABCF3	ENSG00000161204	3:184187761-184187860:+	0.15254243	0.055555622	0.041692
13	ABCF3	ENSG00000161204	3:184189931-184190998:+	0.14285719	0.056603823	0.028056
14	ABHD16A	ENSG00000204427	6:31688786-31689014:-	0.12595423	0.037356356	0.003285
15	ABTB1	ENSG00000114626	3:127677843-127679984:+	0.36290328	0.125000091	0.008648
16	ABTB1	ENSG00000114626	3:127676581-127676966:+	0.24542129	0.062189105	0.000509
17	AC007192.4	ENSG00000268173	19:18160969-18161053:+	0.10344848	0.00934616	0.043881
18	AC011380.9	ENSG00000283155	5:140562499-140562662:-	0.26190487	0.069620381	0.033298
19	ACADVL	ENSG00000072778	17:7220529-7220603:+	0.14245017	0.071269521	0.024632
20	ACD	ENSG00000102977	16:67657684-67657761:-	0.16296303	0.037735919	0.011712
21	ACD	ENSG00000102977	16:67659613-67659701:-	0.1000001	0.013958498	0.044579
22	ACO2	ENSG00000100412	22:41526453-41527287:+	0.11407162	0.046181193	0.001037
23	ACOT8	ENSG00000101473	20:45843721-45844262:-	0.22279798	0.051118267	0.001285
24	ACOT8	ENSG00000101473	20:45844420-45848449:-	0.15492964	0.043333394	0.012641
25	ACSS2	ENSG00000131069	20:34921462-34921543:+	0.27840919	0.064516244	0.015742
26	ACSS2	ENSG00000131069	20:34921600-34921785:+	0.21031752	0.070422617	0.027193
27	ACTN4	ENSG00000130402	19:38727103-38728315:+	0.19565226	0.052044702	0.022933
28	ACTN4	ENSG00000130402	19:38709476-38711275:+	0.10443042	0.035143818	0.026623
29	ACTN4	ENSG00000130402	19:38711361-38714468:+	0.20394742	0.061497384	0.004968
30	ACTR1B	ENSG00000115073	2:97661946-97663842:-	0.43636368	0.166666754	0.009004
31	ACTR1B	ENSG00000115073	2:97656960-97657151:-	0.41095894	0.107544216	0.000304
32	ACTR1B	ENSG00000115073	2:97658643-97658878:-	0.3573668	0.151785779	0.005882
33	ACTR8	ENSG00000113812	3:53878467-53879938:-	0.1052633	0.011496321	0.03259
34	ADAM11	ENSG00000073670	17:44778242-44779221:+	0.1818183	0.018292769	0.006234
35	ADAMTSL2	ENSG00000197859	9:133561295-133566935:+	0.1363638	0.009514201	0.010488
36	ADCY5	ENSG00000173175	3:123325462-123327617:-	0.14666674	0.031250082	0.01952
37	ADD1	ENSG00000087274	4:2926112-2928170:+	0.10130721	0.034820482	0.001802
38	ADD1	ENSG00000087274	4:2926675-2928170:+	0.18057927	0.066536266	0.018245
39	ADD1	ENSG00000087274	4:2905015-2907742:+	0.102289	0.054852338	0.01103
40	ADD1	ENSG00000087274	4:2909431-2914883:+	0.26890765	0.072847784	0.017449
41	ADGRB1	ENSG00000181790	8:142522710-142524237:+	0.12612626	0.009852292	0.009304
42	ADGRB1	ENSG00000181790	8:142542647-142543402:+	0.1010639	0.020979076	0.014055
43	ADGRB1	ENSG00000181790	8:142543438-142543600:+	0.15899586	0.070621524	0.047537
44	ADGRE5	ENSG00000123146	19:14408009-14408091:+	0.26229522	0.033333495	0.021811
45	ADGRL1	ENSG00000072071	19:14160297-14160592:-	0.1274511	0.007677191	0.006285
46	ADGRL1	ENSG00000072071	19:14161626-14162605:-	0.10588244	0.009682927	0.012285
47	ADHFE1	ENSG00000147576	8:66456895-66457069:+	0.40372678	0.105691163	0.006216
48	ADRM1	ENSG00000130706	20:62306734-62307370:+	0.16228075	0.050980448	0.012546

S/N	Gene Affected	ENSEMBL_ID	Position of retained intron	IR ratio AD	IR ratio Control	*p*- value
49	ADRM1	ENSG00000130706	20:62306320-62306647:+	0.116041	0.039877344	0.017094
50	AFMID	ENSG00000183077	17:78191060-78206910:+	0.3174604	0.105263281	0.046434
51	AFTPH	ENSG00000119844	2:64581273-64585421:+	0.17142863	0.053333395	0.02016
52	AGAP3	ENSG00000133612	7:151118632-151122724:+	0.21818194	0.044776264	0.045686
53	AGBL5	ENSG00000084693	2:27058602-27059189:+	0.03000009	0.142857291	0.042738
54	AGFG1	ENSG00000173744	2:227550106-227551958:+	0.27450989	0.034313808	0.00179
55	AIFM1	ENSG00000156709	X:130139871-130140532:-	0.12931041	0.036809885	0.036608
56	AKAP9	ENSG00000127914	7:92105763-92107292:+	0.11475414	0.044444493	0.040613
57	ALCAM	ENSG00000170017	3:105550259-105552143:+	0.16626509	0.082352988	0.048815
58	ALCAM	ENSG00000170017	3:105550259-105552467:+	0.22558928	0.088607662	0.022375
59	ALCAM	ENSG00000170017	3:105552182-105552467:+	0.12045457	0.04186051	0.014889
60	ALKBH6	ENSG00000239382	19:36011045-36011403:-	0.36231889	0.147783345	0.027019
61	ALS2	ENSG00000003393	2:201704218-201704453:-	0.03763446	0.111111193	0.049354
62	AMZ2P1	ENSG00000214174	17:64972927-64973241:-	0.21495331	0.070080911	0.002819
63	ANAPC2	ENSG00000176248	9:137183791-137184912:-	0.21428581	0.06611581	0.04773
64	ANK2	ENSG00000145362	4:113372625-113373089:+	0.22302168	0.05762723	0.025464
65	ANKH	ENSG00000154122	5:14711310-14712873:-	0.23676883	0.058011085	3.99E-05
66	ANKRA2	ENSG00000164331	5:73552852-73553405:-	0.4132232	0.182266111	0.033916
67	ANKRA2	ENSG00000164331	5:73553486-73554321:-	0.27956994	0.103773659	0.015578
68	ANKRA2	ENSG00000164331	5:73555585-73557574:-	0.40458023	0.132867254	0.020836
69	ANKRD19P	ENSG00000187984	9:92826578-92836776:+	0.64705887	0.120482107	0.006179
70	ANKRD28	ENSG00000206560	3:15677056-15677479:-	0.1726191	0.066225235	0.045115
71	AP1S2	ENSG00000182287	X:15827372-15828191:-	0.39790579	0.043782884	1.79E-07
72	AP1S2	ENSG00000182287	X:15828200-15845378:-	0.10483878	0.015473928	0.002539
73	AP3D1	ENSG00000065000	19:2110896-2111284:-	0.12408762	0.03801173	0.003686
74	AP3D1	ENSG00000065000	19:2111332-2111678:-	0.16104872	0.047619086	0.001975
75	AP3D1	ENSG00000065000	19:2111828-2112859:-	0.22093029	0.042016863	0.000738
76	AP5Z1	ENSG00000242802	7:4781754-4783315:+	0.29268307	0.060000182	0.049241
77	APC2	ENSG00000115266	19:1458060-1460180:+	0.23021588	0.061224555	0.004939
78	APLP1	ENSG00000105290	19:35877825-35878081:+	0.16251361	0.052451614	0.021665
79	APLP1	ENSG00000105290	19:35878108-35878583:+	0.10360804	0.035550817	0.015547
80	APOL2	ENSG00000128335	22:36233455-36239440:-	0.15723275	0.038461579	0.001634
81	APPBP2	ENSG00000062725	17:60454492-60456295:-	0.16580317	0.052631651	0.027902
82	APPBP2	ENSG00000062725	17:60461915-60461993:-	0.10588241	0.03141367	0.034183
83	ARFGAP1	ENSG00000101199	20:63276651-63277204:+	0.12408767	0.02985082	0.02981
84	ARFGAP1	ENSG00000101199	20:63282851-63283850:+	0.74157303	0.327272906	0.048533
85	ARFRP1	ENSG00000101246	20:63700530-63700601:-	0.31333341	0.091428679	0.01659
86	ARHGEF4	ENSG00000136002	2:131035288-131038852:+	0.19718322	0.043478372	0.032536
87	ARHGEF4	ENSG00000136002	2:131040440-131041229:+	0.13747237	0.035961359	0.029349
88	ARHGEF4	ENSG00000136002	2:131045446-131046037:+	0.29208931	0.085185287	0.008119
89	ARL2BP	ENSG00000102931	16:57246141-57248536:+	0.12647063	0.045045087	0.012855
90	ARMC6	ENSG00000105676	19:19055928-19057415:+	0.26415102	0.066666759	0.011442
91	ARMC9	ENSG00000135931	2:231215001-231216637:+	0.15151543	0.009514224	0.023288
92	ARMCX2	ENSG00000184867	X:101658142-101658460:-	0.12048199	0.026315852	0.014755
93	ARRB2	ENSG00000141480	17:4720640-4720945:+	0.22597406	0.090909133	0.004312
94	ARRB2	ENSG00000141480	17:4716608-4717216:+	0.35947718	0.04093574	4.67E-05
95	ARRB2	ENSG00000141480	17:4717276-4717684:+	0.22540987	0.05442181	0.000213
96	ARRB2	ENSG00000141480	17:4718023-4718260:+	0.20962202	0.052287612	3.09E-05
97	ARRDC1	ENSG00000197070	9:137614214-137614298:+	0.27272736	0.086419873	0.0439

S/N	Gene Affected	ENSEMBL_ID	Position of retained intron	IR ratio AD	IR ratio Control	*p*- value
98	ASGR1	ENSG00000141505	17:7174067-7174137:-	0.23076943	0.027397428	0.035903
99	ASMTL	ENSG00000169093	X:1403489-1412731:-	0.1081082	0.014925439	0.013051
100	ASPSCR1	ENSG00000169696	17:82016869-82016940:+	0.23333341	0.07746488	0.031045
101	ATAT1	ENSG00000137343	6:30640422-30642175:+	0.40909101	0.121621777	0.037157
102	ATAT1	ENSG00000137343	6:30640603-30642175:+	0.35000009	0.095744808	0.022087
103	ATAT1	ENSG00000137343	6:30643011-30643528:+	0.56962029	0.216049472	0.005181
104	ATF6B	ENSG00000213676	6:32115968-32116479:-	0.39498435	0.164948505	0.002085
105	ATF6B	ENSG00000213676	6:32118866-32118955:-	0.38277517	0.088235361	0.000327
106	ATF6B	ENSG00000213676	6:32119957-32120770:-	0.33750005	0.139664887	0.024832
107	ATG4B	ENSG00000168397	2:241671405-241672190:+	0.57800513	0.226415139	5.12E-05
108	ATG4B	ENSG00000168397	2:241653610-241654545:+	0.13870973	0.030100385	0.003677
109	ATG4B	ENSG00000168397	2:241654647-241655270:+	0.1651652	0.037415004	0.000351
110	ATL2	ENSG00000119787	2:38296590-38296685:-	0.59509207	0.140187029	0.000958
111	ATL2	ENSG00000119787	2:38296700-38298143:-	0.59322036	0.247311933	0.013769
112	ATP13A1	ENSG00000105726	19:19647528-19647598:-	0.19205305	0.035087809	0.009518
113	ATP13A1	ENSG00000105726	19:19649940-19651688:-	0.16666673	0.056910651	0.049219
114	ATP13A4	ENSG00000127249	3:193433917-193435647:-	0.13559336	0.015805951	0.033605
115	ATP1B3	ENSG00000069849	3:141922063-141925530:+	0.10916585	0.037470765	0.00974
116	ATP8A1	ENSG00000124406	4:42588329-42590810:-	0.56910572	0.136842202	0.000603
117	ATP8A1	ENSG00000124406	4:42590884-42600477:-	0.25000009	0.066265166	0.025881
118	ATRX	ENSG00000085224	X:77520916-77521402:-	0.15062766	0.036184255	0.002111
119	AUH	ENSG00000148090	9:91214425-91216058:-	0.15677971	0.064343229	0.04985
120	AUP1	ENSG00000115307	2:74528321-74528416:-	0.10512132	0.042183658	0.018731
121	AUP1	ENSG00000115307	2:74528935-74529131:-	0.63870971	0.302816984	0.00917
122	AUP1	ENSG00000115307	2:74529282-74529361:-	0.10136989	0.034188077	0.019267
123	BBS2	ENSG00000125124	16:56499907-56500853:-	0.17586915	0.078341055	0.014385
124	BBS2	ENSG00000125124	16:56502456-56502672:-	0.30803575	0.116216264	0.001491
125	BBS7	ENSG00000138686	4:121828269-121828401:-	0.11445789	0.023411416	0.004614
126	BCAT2	ENSG00000105552	19:48800297-48806516:-	0.25600007	0.084210624	0.031294
127	BECN1	ENSG00000126581	17:42818877-42819547:-	0.13930352	0.044510427	0.007906
128	BHLHE40	ENSG00000134107	3:4980408-4981391:+	0.10027104	0.036832448	0.015723
129	BHLHE40	ENSG00000134107	3:4981515-4982835:+	0.10352945	0.038610077	0.018845
130	BIN1	ENSG00000136717	2:127051243-127052254:-	0.65200003	0.223776293	0.000105
131	BIN1	ENSG00000136717	2:127052362-127053421:-	0.77980134	0.274261662	1.10E-05
132	BIN1	ENSG00000136717	2:127053445-127053904:-	0.76237626	0.250000052	2.32E-06
133	BIN1	ENSG00000136717	2:127054012-127057472:-	0.7986871	0.317120684	5.61E-05
134	BIN1	ENSG00000136717	2:127057601-127059010:-	0.27240146	0.081374357	4.13E-05
135	BIN1	ENSG00000136717	2:127064018-127068162:-	0.27272732	0.037459327	4.12E-06
136	BIN1	ENSG00000136717	2:127068255-127068923:-	0.16878986	0.01048954	2.57E-06
137	BIN3	ENSG00000147439	8:22624363-22629963:-	0.24285724	0.068965632	0.040745
138	BIRC6	ENSG00000115760	2:32478818-32479461:+	0.00862224	0.136363846	0.007569
139	BOK	ENSG00000176720	2:241570288-241572296:+	0.21604947	0.070967832	0.033122
140	BRD2	ENSG00000204256	6:32976169-32976249:+	0.19746841	0.058997115	0.00833
141	BRD8	ENSG00000112983	5:138157267-138159554:-	0.15463919	0.053333358	0.000404
142	BRD8	ENSG00000112983	5:138170409-138170831:-	0.13551407	0.034482813	0.009039
143	BRSK1	ENSG00000160469	19:55303826-55304049:+	0.11446746	0.014705914	9.92E-05
144	BRSK1	ENSG00000160469	19:55304110-55304550:+	0.25775197	0.04284107	3.33E-07
145	C17orf49	ENSG00000258315	17:7015156-7015773:+	0.19512201	0.064102633	0.022289
146	C17orf49	ENSG00000258315	17:7016685-7016909:+	0.10566042	0.011661842	0.000334

S/N	Gene Affected	ENSEMBL_ID	Position of retained intron	IR ratio AD	IR ratio Control	*p*- value
147	C18orf8	ENSG00000141452	18:23529712-23530027:+	0.31770838	0.076271246	0.000435
148	C18orf8	ENSG00000141452	18:23530132-23530228:+	0.13468018	0.04363641	0.011662
149	C18orf8	ENSG00000141452	18:23530297-23530386:+	0.1482759	0.047445301	0.007801
150	C19orf60	ENSG00000006015	19:18589683-18590853:+	0.11111116	0.029585841	0.007083
151	C8orf59	ENSG00000176731	8:85214631-85214920:-	0.14092449	0.07986505	0.048208
152	C8orf76	ENSG00000189376	8:123239144-123241229:-	0.32432447	0.063829978	0.04383
153	C9orf142	ENSG00000148362	9:136992562-136992639:+	0.05806457	0.23333343	0.003349
154	CA11	ENSG00000063180	19:48638144-48638887:-	0.1066754	0.036665006	8.07E-06
155	CABYR	ENSG00000154040	18:24139118-24143090:+	0.11111132	0.010411777	0.0371
156	CABYR	ENSG00000154040	18:24143259-24143359:+	0.12500021	0.010820084	0.031526
157	CACNA1A	ENSG00000141837	19:13332925-13334377:-	0.26470606	0.042253697	0.042638
158	CACNA2D1	ENSG00000153956	7:82060527-82064303:-	0.10810831	0.006648489	0.014819
159	CALB1	ENSG00000104327	8:90060286-90060628:-	0.12500015	0.020833424	0.040966
160	CALB1	ENSG00000104327	8:90063153-90063280:-	0.10000012	0.0022073	0.00023
161	CALB1	ENSG00000104327	8:90063320-90063405:-	0.11111124	0.009345859	0.01041
162	CALB1	ENSG00000104327	8:90063461-90065897:-	0.1714287	0.032710345	0.016108
163	CAMK1	ENSG00000134072	3:9757846-9759487:-	0.11333336	0.044405003	0.010529
164	CAMK1	ENSG00000134072	3:9761757-9762913:-	0.11522638	0.03787883	0.015066
165	CAMK1	ENSG00000134072	3:9757621-9757728:-	0.18965523	0.073275921	0.031248
166	CAMTA2	ENSG00000108509	17:4969337-4969499:-	0.12844043	0.008695683	4.14E-05
167	CAMTA2	ENSG00000108509	17:4969520-4969629:-	0.1428572	0.013725526	8.98E-05
168	CAMTA2	ENSG00000108509	17:4983043-4985879:-	0.15555561	0.037037085	0.003839
169	CARM1	ENSG00000142453	19:10916777-10919594:+	0.17757017	0.050691324	0.026685
170	CARM1	ENSG00000142453	19:10919680-10919876:+	0.28776985	0.103286485	0.030816
171	CASD1	ENSG00000127995	7:94551478-94552349:+	0.13718416	0.048223393	0.012018
172	CASD1	ENSG00000127995	7:94552427-94554482:+	0.13114758	0.051546434	0.02005
173	CASKIN2	ENSG00000177303	17:75503527-75503658:-	0.18965528	0.02631592	0.023081
174	CBX7	ENSG00000100307	22:39138702-39141370:-	0.18257266	0.053731394	0.004465
175	CBX7	ENSG00000100307	22:39134048-39134400:-	0.17316023	0.017793639	0.000109
176	CCAR2	ENSG00000158941	8:22619355-22619637:+	0.1285141	0.031168871	0.002634
177	CCDC130	ENSG00000104957	19:13759272-13762298:+	0.39062505	0.186046598	0.047257
178	CCDC136	ENSG00000128596	7:128805901-128817757:+	0.14516136	0.019851161	0.00079
179	CCDC136	ENSG00000128596	7:128817864-128821798:+	0.27935227	0.083606626	0.002471
180	CCDC74A	ENSG00000163040	2:131532937-131533012:+	0.11111125	0.008360968	0.012741
181	CCHCR1	ENSG00000204536	6:31142716-31142962:-	0.47058827	0.222222301	0.02286
182	CCHCR1	ENSG00000204536	6:31144788-31144884:-	0.18571442	0.01853111	0.019902
183	CCT6A	ENSG00000146731	7:56061849-56062682:+	0.10298106	0.038872724	0.008251
184	CCT8	ENSG00000156261	21:29062243-29062327:-	0.10964469	0.02600299	8.77E-06
185	CCT8	ENSG00000156261	21:29062415-29062489:-	0.16645492	0.037600739	2.08E-06
186	CD37	ENSG00000104894	19:49337033-49337146:+	0.10752702	0.012262746	0.037233
187	CD47	ENSG00000196776	3:108047292-108049618:-	0.26145558	0.082777131	0.012011
188	CD47	ENSG00000196776	3:108049651-108050577:-	0.23773591	0.088815898	0.040916
189	CD47	ENSG00000196776	3:108050602-108051938:-	0.28571434	0.105678345	0.027838
190	CDC25B	ENSG00000101224	20:3801388-3801721:+	0.38559327	0.169230848	0.023017
191	CDC27	ENSG00000004897	17:47157124-47157229:-	0.13483156	0.009265564	0.005868
192	CDC37	ENSG00000105401	19:10395343-10395434:-	0.20125789	0.0898438	0.009855
193	CDK16	ENSG00000102225	X:47226027-47226278:+	0.18678819	0.053701054	0.000452
194	CDK20	ENSG00000156345	9:87970897-87971146:-	0.13333348	0.005410044	0.002438
195	CDK3	ENSG00000250506	17:76001951-76002021:+	0.11594218	0.011740929	0.0299

S/N	Gene Affected	ENSEMBL_ID	Position of retained intron	IR ratio AD	IR ratio Control	*p*- value
196	CEBPZOS	ENSG00000218739	2:37201726-37201862:+	0.75463919	0.402535805	0.044372
197	CENPT	ENSG00000102901	16:67828390-67828473:-	0.11834324	0.02127664	0.002084
198	CENPT	ENSG00000102901	16:67828843-67829422:-	0.10119051	0.039711237	0.041872
199	CEP41	ENSG00000106477	7:130400821-130401880:-	0.11627927	0.010820077	0.032321
200	CERS4	ENSG00000090661	19:8255879-8256235:+	0.27635333	0.115577967	0.030221
201	CERS4	ENSG00000090661	19:8257985-8261687:+	0.13942312	0.028985553	0.001801
202	CES2	ENSG00000172831	16:66941848-66942104:+	0.1125001	0.025157311	0.044048
203	CHD3	ENSG00000170004	17:7909338-7910427:+	0.30303037	0.107296239	0.025586
204	CHERP	ENSG00000085872	19:16519352-16519620:-	0.32624119	0.087591322	0.004129
205	CIAPIN1	ENSG00000005194	16:57430339-57431150:-	0.11643841	0.029316007	0.009972
206	CIRBP	ENSG00000099622	19:1271467-1271550:+	0.15384633	0.006416565	0.003532
207	CLASP2	ENSG00000163539	3:33573354-33574472:-	0.46913584	0.107883957	0.005801
208	CLASP2	ENSG00000163539	3:33574496-33576168:-	0.32307699	0.069958962	0.008914
209	CLASRP	ENSG00000104859	19:45052892-45053097:+	0.28846161	0.086419836	0.009165
210	CLASRP	ENSG00000104859	19:45057898-45059267:+	0.10869571	0.023668703	0.019033
211	CLCN2	ENSG00000114859	3:184352331-184352442:-	0.19587642	0.024995438	0.035473
212	CLIP4	ENSG00000115295	2:29115665-29121373:+	0.12500024	0.012262754	0.043672
213	CLOCK	ENSG00000134852	4:55450232-55453053:-	0.1538464	0.012262751	0.025948
214	CLTA	ENSG00000122705	9:36209320-36210624:+	0.10385763	0.013847713	0.000724
215	CLTA	ENSG00000122705	9:36209320-36211602:+	0.12996395	0.01985115	0.000149
216	CLTC	ENSG00000141367	17:59687021-59690635:+	0.13104841	0.056748498	0.011424
217	CLUH	ENSG00000132361	17:2690777-2691608:-	0.16766472	0.04926114	0.010068
218	CLUH	ENSG00000132361	17:2695526-2696158:-	0.24096395	0.070000113	0.039711
219	CMC2	ENSG00000103121	16:80997429-80998284:-	0.05244762	0.218935123	0.014867
220	CNOT11	ENSG00000158435	2:101266879-101269039:+	0.10655742	0.040000051	0.046326
221	CNOT11	ENSG00000158435	2:101269136-101269215:+	0.45977015	0.200819748	0.009732
222	CNOT3	ENSG00000088038	19:54146657-54148147:+	0.18292696	0.016535457	0.015651
223	CNTNAP1	ENSG00000108797	17:42685420-42685956:+	0.19607858	0.013333426	0.00596
224	CNTNAP1	ENSG00000108797	17:42691991-42692498:+	0.16000007	0.040000072	0.014277
225	CNTNAP1	ENSG00000108797	17:42697367-42697553:+	0.12949649	0.035598791	0.03802
226	CNTRL	ENSG00000119397	9:121161971-121162053:+	0.0095142	0.100000263	0.037866
227	COG1	ENSG00000166685	17:73205680-73206153:+	0.10924376	0.03455729	0.029451
228	COG1	ENSG00000166685	17:73196751-73196899:+	0.1190478	0.012213706	0.039825
229	COG1	ENSG00000166685	17:73197081-73197225:+	0.1040001	0.016736473	0.021898
230	COL11A2	ENSG00000204248	6:33163818-33164266:-	0.25531948	0.022992653	0.037836
231	COLGALT1	ENSG00000130309	19:17579609-17580698:+	0.72774873	0.345679115	0.027363
232	COPS7B	ENSG00000144524	2:231786538-231788554:+	0.13157915	0.013459113	0.038409
233	COPS7B	ENSG00000144524	2:231788732-231791732:+	0.20645167	0.079754674	0.038557
234	CPNE1	ENSG00000214078	20:35627413-35630438:-	0.37686571	0.191489436	0.034757
235	CPNE1	ENSG00000214078	20:35632025-35632162:-	0.10256415	0.01865676	0.003731
236	CPNE2	ENSG00000140848	16:57146321-57147550:+	0.12396697	0.048583039	0.022246
237	CPNE7	ENSG00000178773	16:89595603-89596483:+	0.21428593	0.027777939	0.045811
238	CPSF1	ENSG00000071894	8:144394038-144394112:-	0.29611656	0.104046324	0.01477
239	CPSF1	ENSG00000071894	8:144395182-144395264:-	0.1300449	0.01834868	0.002854
240	CPSF1	ENSG00000071894	8:144394160-144394233:-	0.24778766	0.073891689	0.004693
241	CPSF3	ENSG00000119203	2:9467776-9471342:+	0.10714291	0.034632086	0.033652
242	CPT1C	ENSG00000169169	19:49697465-49700683:+	0.23076937	0.030000121	0.014383
243	CPT1C	ENSG00000169169	19:49705114-49705213:+	0.54545461	0.177914278	0.024495
244	CPT1C	ENSG00000169169	19:49710857-49711808:+	0.43715853	0.154166791	0.018677

S/N	Gene Affected	ENSEMBL_ID	Position of retained intron	IR ratio AD	IR ratio Control	*p*- value
245	CRELD1	ENSG00000163703	3:9942896-9943076:+	0.12292363	0.032258101	0.002487
246	CRELD1	ENSG00000163703	3:9943515-9944364:+	0.38381204	0.132107088	0.000941
247	CRHR1	ENSG00000120088	17:45833849-45834006:+	0.25000031	0.018531174	0.043515
248	CRTC1	ENSG00000105662	19:18768793-18771441:+	0.16190484	0.046783705	0.034006
249	CRTC1	ENSG00000105662	19:18771546-18774899:+	0.20879129	0.025510272	0.0014
250	CRYBG3	ENSG00000080200	3:97942443-97943225:+	0.16438362	0.480874512	0.009274
251	CSNK1D	ENSG00000141551	17:82246039-82248874:-	0.23577243	0.076923167	0.028193
252	CSNK1E	ENSG00000213923	22:38293319-38294108:-	0.17848413	0.057086646	0.000475
253	CSNK2B	ENSG00000204435	6:31669172-31669318:+	0.12701423	0.058823546	0.000907
254	CSPG5	ENSG00000114646	3:47562761-47569070:-	0.14074078	0.059459503	0.027151
255	CTBP1	ENSG00000159692	4:1241519-1248915:-	0.55371905	0.24683556	0.03317
256	CTNNA1	ENSG00000044115	5:138930935-138932577:+	0.1137539	0.042465776	0.001142
257	CTU2	ENSG00000174177	16:88714926-88715047:+	0.15476201	0.023809644	0.033928
258	CUL7	ENSG00000044090	6:43038692-43038841:-	0.34920646	0.092105412	0.033711
259	CUL9	ENSG00000112659	6:43221321-43221684:+	0.24615394	0.077519492	0.041579
260	CWC22	ENSG00000163510	2:179970856-179970940:-	0.1123597	0.008115062	0.013744
261	CYP27A1	ENSG00000135929	2:218814187-218814379:+	0.10126586	0.034482807	0.027551
262	D2HGDH	ENSG00000180902	2:241756014-241767709:+	0.11224499	0.010537304	0.014288
263	DAZAP1	ENSG00000071626	19:1422396-1425877:+	0.33181823	0.130044919	0.013579
264	DAZAP1	ENSG00000071626	19:1425960-1428841:+	0.53358212	0.226337511	0.002223
265	DCTN1	ENSG00000204843	2:74374340-74376741:-	0.12962976	0.020362086	0.026582
266	DDX1	ENSG00000079785	2:15628517-15628637:+	0.15199039	0.039580005	0.000468
267	DDX39B	ENSG00000198563	6:31532911-31535366:-	0.51197265	0.295560784	0.001812
268	DDX3X	ENSG00000215301	X:41341616-41342494:+	0.13991772	0.065810622	0.009548
269	DDX52	ENSG00000278053	17:37620948-37621126:-	0.16853942	0.037037152	0.041171
270	DEF8	ENSG00000140995	16:89962125-89963362:+	0.52150548	0.157232874	0.023446
271	DENND6B	ENSG00000205593	22:50318889-50318964:-	0.11538478	0.009951913	0.025861
272	DGAT1	ENSG00000185000	8:144317445-144317543:-	0.16517862	0.07083339	0.044095
273	DGCR6	ENSG00000183628	22:18910272-18910887:+	0.30769236	0.138888977	0.044046
274	DGKQ	ENSG00000145214	4:960721-961048:-	0.32653075	0.074074258	0.048406
275	DGKQ	ENSG00000145214	4:962920-963138:-	0.10810834	0.00823619	0.030443
276	DGKQ	ENSG00000145214	4:966527-966747:-	0.14285738	0.011036489	0.027911
277	DGKQ	ENSG00000145214	4:967649-967727:-	0.34782645	0.014914198	0.015541
278	DGUOK	ENSG00000114956	2:73950732-73958145:+	0.19354846	0.058823608	0.027911
279	DGUOK	ENSG00000114956	2:73957240-73958145:+	0.11744971	0.04699744	0.043826
280	DHX30	ENSG00000132153	3:47847956-47848179:+	0.13654624	0.042424296	0.015694
281	DHX38	ENSG00000140829	16:72097009-72097676:+	0.18656722	0.058823614	0.03354
282	DHX8	ENSG00000067596	17:43492292-43492680:+	0.10204101	0.010820078	0.044827
283	DLG1	ENSG00000075711	3:197066754-197068501:-	0.37500005	0.063545233	0.000391
284	DLG1	ENSG00000075711	3:197068537-197069218:-	0.40196085	0.094527479	0.005424
285	DLG1	ENSG00000075711	3:197069260-197075836:-	0.15743445	0.050666722	0.012049
286	DLGAP4	ENSG00000080845	20:36497066-36499241:+	0.28378387	0.059259354	0.006928
287	DMTF1	ENSG00000135164	7:87174669-87179544:+	0.10671941	0.040000051	0.045024
288	DMTF1	ENSG00000135164	7:87179702-87181308:+	0.20338988	0.085227342	0.042676
289	DNAJB11	ENSG00000090520	3:186577800-186581370:+	0.18974363	0.083743899	0.040359
290	DNAJC2	ENSG00000105821	7:103319678-103319755:-	0.20329681	0.042780843	0.013841
291	DNAJC2	ENSG00000105821	7:103322632-103322702:-	0.41361268	0.126213776	0.041717
292	DOCK3	ENSG00000088538	3:51275206-51277607:+	0.48051953	0.064102665	0.000351
293	DOCK3	ENSG00000088538	3:51277754-51280105:+	0.55932207	0.05384626	0.000146

S/N	Gene Affected	ENSEMBL_ID	Position of retained intron	IR ratio AD	IR ratio Control	*p*- value
294	DOCK3	ENSG00000088538	3:51333027-51333157:+	0.13793117	0.025862175	0.047971
295	DOCK4	ENSG00000128512	7:111732287-111736916:-	0.57894739	0.104762079	0.007257
296	DPH1	ENSG00000108963	17:2036956-2039754:+	0.42748096	0.182353037	0.026992
297	DPH1	ENSG00000108963	17:2036091-2036528:+	0.26168232	0.025477781	0.000471
298	DPH7	ENSG00000148399	9:137576167-137576308:-	0.00935297	0.153846688	0.037864
299	DPM1	ENSG00000000419	20:50945762-50945846:-	0.14189193	0.043269271	0.00428
300	DPM2	ENSG00000136908	9:127936655-127937433:-	0.46315792	0.232758694	0.02338
301	DPP7	ENSG00000176978	9:137112805-137112952:-	0.17658355	0.05016728	0.004297
302	DPP7	ENSG00000176978	9:137113287-137113360:-	0.26148413	0.107476686	0.006419
303	DPY19L4	ENSG00000156162	8:94788052-94789745:+	0.20833339	0.066666773	0.04643
304	DRG2	ENSG00000108591	17:18099077-18099632:+	0.26744194	0.057377144	0.008294
305	DROSHA	ENSG00000113360	5:31466281-31467938:-	0.19000007	0.060606141	0.034495
306	DST	ENSG00000151914	6:56460254-56463564:-	0.24257431	0.053164615	0.000718
307	DTNB	ENSG00000138101	2:25383889-25388201:-	0.20588251	0.030612364	0.025832
308	DUS1L	ENSG00000169718	17:82060093-82060700:-	0.15254241	0.040178618	0.003716
309	DUSP1	ENSG00000120129	5:172769132-172769574:-	0.1461149	0.068493191	0.027392
310	DUSP1	ENSG00000120129	5:172770306-172770585:-	0.20055714	0.063953543	0.005474
311	DUSP11	ENSG00000144048	2:73762859-73766417:-	0.21052637	0.075949432	0.02269
312	DYNC1I2	ENSG00000077380	2:171692894-171707232:+	0.02614387	0.142857346	0.035778
313	DYSF	ENSG00000135636	2:71674296-71679056:+	0.1008265	0.033033087	0.034881
314	E2F4	ENSG00000205250	16:67194980-67195781:+	0.2131148	0.087248391	0.037313
315	E2F4	ENSG00000205250	16:67197646-67197866:+	0.30985921	0.093896781	0.002756
316	EDC4	ENSG00000038358	16:67877845-67878165:+	0.2580646	0.04504514	0.005421
317	EDC4	ENSG00000038358	16:67880216-67880556:+	0.21848748	0.063829894	0.038204
318	EDC4	ENSG00000038358	16:67881417-67881496:+	0.29054061	0.06962034	0.005296
319	EDC4	ENSG00000038358	16:67881533-67881667:+	0.24870471	0.052287645	0.001091
320	EDC4	ENSG00000038358	16:67883731-67883955:+	0.29186606	0.097826139	0.001818
321	EFR3B	ENSG00000084710	2:25128332-25129974:+	0.10638313	0.006897798	0.008757
322	EGFL7	ENSG00000172889	9:136669721-136669913:+	0.23232331	0.051903195	0.005458
323	EGFL7	ENSG00000172889	9:136671014-136671925:+	0.31524016	0.106576063	0.01444
324	EGLN2	ENSG00000269858	19:40801415-40806554:+	0.17410718	0.074204993	0.022747
325	EHMT2	ENSG00000204371	6:31884559-31884644:-	0.14070357	0.026738024	0.004091
326	EIF2AK2	ENSG00000055332	2:37107395-37107473:-	0.10000011	0.005463605	0.003751
327	EIF2S3	ENSG00000130741	X:24073263-24076721:+	0.234139	0.093922685	0.000698
328	EIF3G	ENSG00000130811	19:10116074-10116799:-	0.16206265	0.042283337	0.000751
329	EIF4E2	ENSG00000135930	2:232574341-232580903:+	0.20967755	0.046153967	0.036911
330	EIF5A	ENSG00000132507	17:7308113-7309614:+	0.25000027	0.014149332	0.012869
331	EIF5A	ENSG00000132507	17:7309800-7311017:+	0.11495179	0.016412678	1.05E-07
332	ELAC2	ENSG00000006744	17:12994884-12994962:-	0.12149537	0.039215732	0.016313
333	ELP2	ENSG00000134759	18:36164667-36167100:+	0.18473899	0.080971702	0.016982
334	ELP2	ENSG00000134759	18:36170196-36171046:+	0.14912285	0.030651384	0.001099
335	EML2	ENSG00000125746	19:45619191-45621206:-	0.1193549	0.038461598	0.031763
336	ENDOG	ENSG00000167136	9:128820848-128822327:+	0.34365327	0.171296342	0.007878
337	ENTPD4	ENSG00000197217	8:23434478-23435391:-	0.12142863	0.030927892	0.017295
338	EPB41L2	ENSG00000079819	6:130880489-130880600:-	0.1298703	0.011560789	0.021431
339	EPB41L3	ENSG00000082397	18:5394793-5395066:-	0.1253688	0.027983596	0.00588
340	EPHX2	ENSG00000120915	8:27543829-27544185:+	0.32352948	0.056179877	0.003168
341	EPHX2	ENSG00000120915	8:27544244-27544443:+	0.50961549	0.149253892	0.02577
342	EPN2	ENSG00000072134	17:19332068-19333955:+	0.27044028	0.114832584	0.007343

S/N	Gene Affected	ENSEMBL_ID	Position of retained intron	IR ratio AD	IR ratio Control	*p*- value
343	ERCC3	ENSG00000163161	2:127289501-127289688:-	0.21559637	0.063025261	0.002467
344	ERF	ENSG00000105722	19:42249942-42250330:-	0.32558146	0.109091002	0.016608
345	ERGIC3	ENSG00000125991	20:35554386-35555043:+	0.10599085	0.023297541	0.009145
346	ETS2	ENSG00000157557	21:38806120-38810034:+	0.18253978	0.040697764	0.018305
347	EVI2A	ENSG00000126860	17:31319023-31320372:-	0.34883744	0.036788167	0.030292
348	EWSR1	ENSG00000182944	22:29291599-29292136:+	0.42974362	0.191907588	0.005989
349	EXOC3	ENSG00000180104	5:459459-461959:+	0.11111115	0.050377873	0.048199
350	EXOC6B	ENSG00000144036	2:72179461-72184074:-	0.20000011	0.048780581	0.033376
351	EXOC7	ENSG00000182473	17:76088122-76088463:-	0.17085433	0.058823595	0.024802
352	EXOC7	ENSG00000182473	17:76091235-76094413:-	0.26771661	0.086363744	0.030915
353	EXOSC9	ENSG00000123737	4:121813380-121813865:+	0.10775868	0.020408212	0.005649
354	EZH1	ENSG00000108799	17:42702961-42703739:-	0.66124663	0.303977338	0.001691
355	FAM102A	ENSG00000167106	9:127944898-127945366:-	0.10422539	0.04150948	0.043617
356	FAM120B	ENSG00000112584	6:170391121-170395486:+	0.39247316	0.134199203	0.002528
357	FAM133B	ENSG00000234545	7:92577717-92578149:-	0.26291084	0.085106444	0.004763
358	FAM13B	ENSG00000031003	5:137945997-137946227:-	0.30645172	0.087805009	0.027809
359	FAM149A	ENSG00000109794	4:186167262-186171913:+	0.14285727	0.02272736	0.025027
360	FAM184A	ENSG00000111879	6:118966952-118975023:-	0.12903245	0.01709412	0.046203
361	FAM3A	ENSG00000071889	X:154507861-154508288:-	0.17647065	0.062992202	0.04375
362	FAM91A1	ENSG00000176853	8:123810351-123812518:+	0.35398236	0.113207639	0.011661
363	FAM92A	ENSG00000188343	8:93708016-93709770:+	0.10884363	0.020080393	0.022922
364	FAM98C	ENSG00000130244	19:38405421-38405518:+	0.1626017	0.053892293	0.045124
365	FAM98C	ENSG00000130244	19:38405635-38406909:+	0.25000005	0.113537183	0.042891
366	FAM98C	ENSG00000130244	19:38407077-38408750:+	0.27567571	0.133333396	0.037891
367	FARP2	ENSG00000006607	2:241484331-241489961:+	0.55319156	0.169491701	0.035389
368	FASTK	ENSG00000164896	7:151079021-151079499:-	0.39784952	0.158536678	0.016408
369	FBXO11	ENSG00000138081	2:47834711-47834787:-	0.13300502	0.008264521	0.002151
370	FBXO11	ENSG00000138081	2:47808247-47808328:-	0.10050253	0.031161495	0.000616
371	FBXO9	ENSG00000112146	6:53092824-53093465:+	0.36533962	0.130307547	0.003596
372	FCHSD1	ENSG00000197948	5:141644690-141644858:-	0.12857155	0.435897674	0.043432
373	FHOD1	ENSG00000135723	16:67229718-67229792:-	0.35897453	0.06451632	0.030138
374	FIP1L1	ENSG00000145216	4:53440608-53442652:+	0.27044031	0.097222303	0.019452
375	FLCN	ENSG00000154803	17:17215316-17216379:-	0.43783789	0.139072931	0.003389
376	FLCN	ENSG00000154803	17:17216503-17217068:-	0.3221477	0.094488267	0.005283
377	FLII	ENSG00000177731	17:18253458-18253543:-	0.19879523	0.080645234	0.045488
378	FLOT1	ENSG00000137312	6:30741333-30741613:-	0.12804881	0.056562771	0.024619
379	FMNL1	ENSG00000184922	17:45244025-45244175:+	0.17142865	0.014285792	0.003136
380	FMNL1	ENSG00000184922	17:45246604-45246866:+	0.33333341	0.051282143	0.00156
381	FMNL2	ENSG00000157827	2:152643544-152647795:+	0.70370374	0.258620822	0.013031
382	FMNL2	ENSG00000157827	2:152619718-152622536:+	0.357143	0.038461712	0.015408
383	FMNL2	ENSG00000157827	2:152622557-152625437:+	0.30303046	0.027027177	0.012458
384	FMNL2	ENSG00000157827	2:152640914-152643418:+	0.31730778	0.081761127	0.017155
385	FSD1	ENSG00000105255	19:4318951-4322985:+	0.14843755	0.052631632	0.028434
386	FSD1	ENSG00000105255	19:4310596-4311841:+	0.42857147	0.173010474	0.014015
387	FTSJ1	ENSG00000068438	X:48478168-48478448:+	0.2253522	0.062500115	0.04225
388	G6PD	ENSG00000160211	X:154534496-154535167:-	0.10144935	0.021739198	0.029357
389	GAB1	ENSG00000109458	4:143433716-143437998:+	0.21028045	0.06185577	0.032472
390	GAB1	ENSG00000109458	4:143438600-143439801:+	0.22614848	0.0471699	0.007244
391	GABBR1	ENSG00000204681	6:29627646-29629086:-	0.41333339	0.083333487	0.009666

S/N	Gene Affected	ENSEMBL_ID	Position of retained intron	IR ratio AD	IR ratio Control	*p*- value
392	GABBR1	ENSG00000204681	6:29629107-29630457:-	0.82995952	0.351449373	0.003008
393	GABBR1	ENSG00000204681	6:29630643-29631395:-	0.12571434	0.037500062	0.027073
394	GALT	ENSG00000213930	9:34649564-34650368:+	0.12751686	0.035175958	0.043061
395	GARS	ENSG00000106105	7:30631541-30632246:+	0.14983716	0.038602969	7.94E-05
396	GATB	ENSG00000059691	4:151701518-151703850:-	0.11347523	0.017910484	0.001123
397	GBA2	ENSG00000070610	9:35740362-35740525:-	0.36018961	0.128630762	0.001805
398	GBA2	ENSG00000070610	9:35741890-35744296:-	0.25116283	0.118421114	0.03574
399	GCA	ENSG00000115271	2:162344275-162347577:+	0.24812037	0.090277871	0.040061
400	GCAT	ENSG00000100116	22:37815280-37815417:+	0.13084119	0.030674914	0.020906
401	GCC2	ENSG00000135968	2:108486648-108487698:+	0.1153847	0.010869629	0.006406
402	GFPT2	ENSG00000131459	5:180330834-180331494:-	0.29411774	0.055555684	0.014298
403	GGA3	ENSG00000125447	17:75238389-75238651:-	0.21481492	0.045751737	0.017635
404	GGA3	ENSG00000125447	17:75241696-75242335:-	0.26923084	0.081081175	0.018408
405	GGT7	ENSG00000131067	20:34860042-34860253:-	0.15037601	0.038277577	0.015824
406	GHDC	ENSG00000167925	17:42190270-42190623:-	0.40397357	0.127118745	0.010209
407	GIT1	ENSG00000108262	17:29578779-29578961:-	0.12456755	0.027160554	0.010262
408	GIT1	ENSG00000108262	17:29578988-29581337:-	0.15087725	0.022988547	0.0003
409	GK	ENSG00000198814	X:30727552-30728731:+	0.44444453	0.142857307	0.047034
410	GLE1	ENSG00000119392	9:128527291-128527455:+	0.33333343	0.081632782	0.020479
411	GNAS	ENSG00000087460	20:58898985-58899948:+	0.175532	0.034883799	0.008699
412	GNB4	ENSG00000114450	3:179413613-179413714:-	0.01082007	0.106383202	0.034057
413	GNL3	ENSG00000163938	3:52693329-52693407:+	0.22006476	0.096103949	0.017711
414	GNPDA2	ENSG00000163281	4:44717295-44718308:-	0.17307701	0.037974767	0.017587
415	GOLGA2	ENSG00000167110	9:128262704-128263033:-	0.23275869	0.075949453	0.02679
416	GOLGA2	ENSG00000167110	9:128263092-128265584:-	0.11627916	0.009860596	0.010012
417	GOPC	ENSG00000047932	6:117569736-117570859:-	0.21476517	0.070351846	0.030663
418	GOPC	ENSG00000047932	6:117575352-117577447:-	0.34920642	0.078571522	0.004629
419	GOPC	ENSG00000047932	6:117577471-117578899:-	0.23913054	0.065217492	0.0346
420	GPAA1	ENSG00000197858	8:144083500-144083713:+	0.15217396	0.046875054	0.013616
421	GPAA1	ENSG00000197858	8:144085138-144085288:+	0.16279075	0.043715903	0.006114
422	GPAT4	ENSG00000158669	8:41609955-41610735:+	0.28112455	0.129186682	0.04697
423	GPBP1	ENSG00000062194	5:57237172-57246299:+	0.40495873	0.124352436	0.00895
424	GPR108	ENSG00000125734	19:6732549-6732986:-	0.39285721	0.088888979	0.001815
425	GPR108	ENSG00000125734	19:6733913-6734004:-	0.18264846	0.065217469	0.038813
426	GPR108	ENSG00000125734	19:6734307-6735621:-	0.16564423	0.02105273	0.010187
427	GPRASP1	ENSG00000198932	X:102652898-102653220:+	0.18518531	0.041025758	0.039295
428	GPS2	ENSG00000132522	17:7313291-7313379:-	0.133452	0.055696249	0.028733
429	GPS2	ENSG00000132522	17:7313721-7313905:-	0.36120403	0.198564629	0.006163
430	GPS2	ENSG00000132522	17:7314597-7314958:-	0.20863312	0.10067118	0.014745
431	GRAMD1A	ENSG00000089351	19:35011554-35013255:+	0.15483876	0.052356083	0.028579
432	GRAMD1A	ENSG00000089351	19:35022038-35022899:+	0.31250006	0.042801627	0.000265
433	GRAMD1A	ENSG00000089351	19:35022911-35023235:+	0.48466261	0.087272803	5.47E-05
434	GRINA	ENSG00000178719	8:143992078-143992244:+	0.10074236	0.014266333	0.000113
435	GRIPAP1	ENSG00000068400	X:48976111-48976240:-	0.12874255	0.036253811	0.001931
436	GRIPAP1	ENSG00000068400	X:48988198-48989610:-	0.18624646	0.051823459	0.001001
437	GRSF1	ENSG00000132463	4:70825431-70826123:-	0.11414398	0.03467567	0.017661
438	GTF3C3	ENSG00000119041	2:196775251-196776009:-	0.10000007	0.023622126	0.047669
439	GTPBP3	ENSG00000130299	19:17341322-17341477:+	0.15217411	0.010411773	0.01364
440	GTPBP6	ENSG00000178605	X:311627-312765:-	0.40217395	0.15909098	0.006942

S/N	Gene Affected	ENSEMBL_ID	Position of retained intron	IR ratio AD	IR ratio Control	*p*- value
441	GTPBP6	ENSG00000178605	X:315299-316913:-	0.10606064	0.040909138	0.037489
442	GZF1	ENSG00000125812	20:23368929-23369583:+	0.1235956	0.014330726	0.025499
443	HARS	ENSG00000170445	5:140683219-140690854:-	0.14516134	0.046647281	0.014476
444	HBS1L	ENSG00000112339	6:134979268-134982457:-	0.11612908	0.032432478	0.009014
445	HCFC1	ENSG00000172534	X:153955542-153956190:-	0.40000009	0.125000151	0.041518
446	HDAC10	ENSG00000100429	22:50246376-50246678:-	0.20491814	0.048000107	0.027145
447	HDAC3	ENSG00000171720	5:141629306-141629683:-	0.20155047	0.064935165	0.048609
448	HDHD2	ENSG00000167220	18:47136449-47150377:-	0.19480529	0.049505055	0.036602
449	HERC3	ENSG00000138641	4:88668081-88669859:+	0.24074084	0.027933041	0.001465
450	HERC5	ENSG00000138646	4:88500985-88504231:+	0.10465129	0.011740924	0.031445
451	HEY1	ENSG00000164683	8:79767092-79767218:-	0.10638312	0.012121302	0.03007
452	HGS	ENSG00000185359	17:81684103-81685604:+	0.30827076	0.09714298	0.031604
453	HGS	ENSG00000185359	17:81685689-81686311:+	0.13425931	0.051851904	0.03586
454	HGS	ENSG00000185359	17:81690234-81690673:+	0.10294123	0.019455302	0.006837
455	HGS	ENSG00000185359	17:81690742-81691446:+	0.13541671	0.011070152	0.00022
456	HGS	ENSG00000185359	17:81693965-81694814:+	0.10300434	0.025531966	0.014607
457	HGS	ENSG00000185359	17:81694853-81694923:+	0.18623486	0.044982745	0.001085
458	HGS	ENSG00000185359	17:81695999-81696356:+	0.19354842	0.051181146	0.001123
459	HGS	ENSG00000185359	17:81696747-81696823:+	0.10970468	0.027397297	0.003309
460	HID1	ENSG00000167861	17:74953086-74953544:-	0.19892482	0.036842184	0.005696
461	HID1	ENSG00000167861	17:74955956-74958140:-	0.16447375	0.046511698	0.016748
462	HIST1H2AC	ENSG00000180573	6:26124633-26138053:+	0.35714295	0.116666807	0.045131
463	HLA-B	ENSG00000234745	6:31355592-31356166:-	0.21300456	0.054115082	0.008087
464	HLA-DMB	ENSG00000242574	6:32934987-32935341:-	0.34170859	0.138364862	0.021495
465	HM13	ENSG00000101294	20:31566295-31568077:+	0.263889	0.056410383	0.020794
466	HMGA1	ENSG00000137309	6:34236963-34237203:+	0.11290337	0.016393546	0.048761
467	HMGCS1	ENSG00000112972	5:43297166-43298008:-	0.11467896	0.0342613	0.026682
468	HNRNPA2B1	ENSG00000122566	7:26197866-26200571:-	0.1789773	0.086274542	0.009923
469	HNRNPA2B1	ENSG00000122566	7:26192338-26192494:-	0.30264134	0.165661739	0.007683
470	HNRNPA3	ENSG00000170144	2:177219314-177219407:+	0.10786916	0.041167684	0.000548
471	HNRNPH1	ENSG00000169045	5:179615595-179616125:-	0.11467578	0.07007128	0.043164
472	HNRNPH1	ENSG00000169045	5:179617110-179617513:-	0.26169849	0.108108171	0.012327
473	HNRNPH1	ENSG00000169045	5:179618323-179619268:-	0.42206407	0.208333363	0.000102
474	HNRNPH1	ENSG00000169045	5:179621035-179621241:-	0.21254615	0.111538503	0.021367
475	HNRNPH1	ENSG00000169045	5:179621397-179623036:-	0.2664942	0.129213519	0.004076
476	HTRA2	ENSG00000115317	2:74531105-74531338:+	0.20647777	0.088803137	0.017442
477	IDH3B	ENSG00000101365	20:2660365-2660456:-	0.14012744	0.023136282	0.00013
478	IDH3B	ENSG00000101365	20:2660590-2660696:-	0.12127239	0.03140706	0.000195
479	IDUA	ENSG00000127415	4:1001563-1001678:+	0.28571443	0.040816512	0.030422
480	IFRD2	ENSG00000214706	3:50290294-50290387:-	0.04504513	0.183333489	0.035385
481	IFRD2	ENSG00000214706	3:50289628-50289711:-	0.15942043	0.014149313	0.015465
482	IFT172	ENSG00000138002	2:27458868-27459377:-	0.38461546	0.146067544	0.049998
483	IKBKB	ENSG00000104365	8:42319421-42319584:+	0.25203265	0.040000176	0.033993
484	ILVBL	ENSG00000105135	19:15115671-15115849:-	0.16309018	0.046296356	0.010655
485	ILVBL	ENSG00000105135	19:15115921-15116000:-	0.22317601	0.084577177	0.017722
486	IMPDH2	ENSG00000178035	3:49026886-49026959:-	0.12933758	0.055702968	0.047167
487	IMPDH2	ENSG00000178035	3:49028532-49028757:-	0.23722632	0.07665511	0.004455
488	IMPDH2	ENSG00000178035	3:49028806-49029252:-	0.26486492	0.060606126	0.001508
489	INPP4A	ENSG00000040933	2:98559495-98563464:+	0.20000011	0.039215779	0.019113

S/N	Gene Affected	ENSEMBL_ID	Position of retained intron	IR ratio AD	IR ratio Control	*p*- value
490	INPP5E	ENSG00000148384	9:136433075-136433154:-	0.2698414	0.036363804	0.023471
491	INTS1	ENSG00000164880	7:1481488-1482545:-	0.31958777	0.053333486	0.016684
492	INTS1	ENSG00000164880	7:1482707-1483741:-	0.38666674	0.090909192	0.003969
493	INTS1	ENSG00000164880	7:1483853-1484002:-	0.39316247	0.103092894	0.008501
494	INTS8	ENSG00000164941	8:94876147-94876220:+	0.17745808	0.06130274	0.014478
495	IRF3	ENSG00000126456	19:49663514-49664442:-	0.33333343	0.067567696	0.01189
496	ISOC2	ENSG00000063241	19:55455330-55455635:-	0.19626177	0.047058907	0.015432
497	ITGA3	ENSG00000005884	17:50075526-50075598:+	0.22857174	0.018394134	0.032917
498	ITGAE	ENSG00000083457	17:3720402-3723687:-	0.15706812	0.045977075	0.016868
499	ITGB4	ENSG00000132470	17:75755850-75756428:+	0.23788553	0.076923165	0.021227
500	ITPA	ENSG00000125877	20:3218632-3221840:+	0.20805377	0.069307025	0.040668
501	IZUMO4	ENSG00000099840	19:2097495-2097928:+	0.14673918	0.013513583	0.002695
502	JOSD2	ENSG00000161677	19:50507699-50510285:-	0.13978498	0.054945101	0.023652
503	KANSL3	ENSG00000114982	2:96602862-96604249:-	0.1000001	0.013435058	0.041157
504	KCNAB1	ENSG00000169282	3:156523947-156531408:+	0.17266194	0.058419321	0.037576
505	KCTD17	ENSG00000100379	22:37059438-37062524:+	0.17500006	0.047846954	0.010944
506	KCTD17	ENSG00000100379	22:37061629-37062524:+	0.29797986	0.082608776	0.004922
507	KDM3A	ENSG00000115548	2:86478029-86478169:+	0.12796218	0.028571522	0.041232
508	KDM5C	ENSG00000126012	X:53198637-53198763:-	0.37305704	0.159624496	0.021611
509	KDM6A	ENSG00000147050	X:45082640-45082714:+	0.25263167	0.011557041	0.000921
510	KHDRBS3	ENSG00000131773	8:135648121-135648539:+	0.08496738	0.228915778	0.038447
511	KIAA0368	ENSG00000136813	9:111383332-111384521:-	0.13962268	0.030898916	0.001016
512	KIAA1191	ENSG00000122203	5:176355749-176361601:-	0.28571442	0.052083466	0.025433
513	KIAA1586	ENSG00000168116	6:57047396-57050773:+	0.1379313	0.01174094	0.03653
514	KIDINS220	ENSG00000134313	2:8736999-8750111:-	0.4880953	0.088050446	0.002686
515	KIF19	ENSG00000196169	17:74346524-74347776:+	0.13333355	0.013459123	0.041128
516	KIZ	ENSG00000088970	20:21244288-21246478:+	0.4038462	0.182795781	0.024595
517	KLC4	ENSG00000137171	6:43066525-43066995:+	0.16935491	0.050505147	0.048697
518	KLC4	ENSG00000137171	6:43073338-43073901:+	0.15547707	0.058479593	0.032013
519	KLHL18	ENSG00000114648	3:47340676-47342718:+	0.35714297	0.057692474	0.017418
520	KLHL18	ENSG00000114648	3:47342830-47343554:+	0.32500018	0.048780716	0.045904
521	KLHL22	ENSG00000099910	22:20446676-20457807:-	0.16000008	0.031055974	0.011053
522	KPTN	ENSG00000118162	19:47480407-47480759:-	0.57831332	0.203125148	0.029549
523	KRBOX4	ENSG00000147121	X:46463308-46471141:+	0.61538469	0.163265503	0.028411
524	KYAT1	ENSG00000171097	9:128835868-128835996:-	0.14705894	0.026315892	0.034253
525	L1CAM	ENSG00000198910	X:153868952-153869519:-	0.14285731	0.020100614	0.035009
526	L3MBTL2	ENSG00000100395	22:41209933-41213892:+	0.1097562	0.021897896	0.044793
527	L3MBTL2	ENSG00000100395	22:41227869-41229539:+	0.16666674	0.040983691	0.025073
528	L3MBTL2	ENSG00000100395	22:41229656-41230138:+	0.20731714	0.07602348	0.048083
529	LAIR1	ENSG00000167613	19:54356267-54356355:-	0.16923117	0.011261756	0.042579
530	LAMB2	ENSG00000172037	3:49123052-49123131:-	0.20588241	0.034482838	0.003452
531	LAMB2	ENSG00000172037	3:49129324-49129603:-	0.41489376	0.076923255	0.01849
532	LAMP2	ENSG00000005893	X:120428626-120441729:-	0.5024631	0.152343845	0.002109
533	LARS	ENSG00000133706	5:146144349-146144471:-	0.1859297	0.060913764	0.009463
534	LARS	ENSG00000133706	5:146144537-146144623:-	0.24813899	0.090047438	0.001722
535	LCA5	ENSG00000135338	6:79487866-79489083:-	0.07216506	0.291666936	0.039611
536	LDHD	ENSG00000166816	16:75112713-75112833:-	0.17391313	0.051724235	0.049507
537	LGALS9	ENSG00000168961	17:27647432-27648835:+	0.14204552	0.036036122	0.031834
538	LGI3	ENSG00000168481	8:22148977-22151488:-	0.14051528	0.038461589	0.006211

S/N	Gene Affected	ENSEMBL_ID	Position of retained intron	IR ratio AD	IR ratio Control	*p*- value
539	LGI3	ENSG00000168481	8:22154213-22154559:-	0.16502469	0.065645583	0.040383
540	LIN7B	ENSG00000104863	19:49118018-49118351:+	0.37356327	0.084112235	0.001059
541	LLGL1	ENSG00000131899	17:18240873-18241450:+	0.40000005	0.192488342	0.02997
542	LMBR1	ENSG00000105983	7:156728007-156728643:-	0.13281257	0.032967101	0.022638
543	LONP1	ENSG00000196365	19:5708403-5711770:-	0.11518329	0.042016855	0.034757
544	LRP11	ENSG00000120256	6:149820703-149826263:-	0.17989423	0.036474212	0.000909
545	LRP11	ENSG00000120256	6:149837463-149842982:-	0.12307698	0.036923122	0.01605
546	LRP2BP	ENSG00000109771	4:185377018-185378080:-	0.55072469	0.213333472	0.038331
547	LRSAM1	ENSG00000148356	9:127452084-127454495:+	0.28125015	0.005933598	0.000307
548	LRSAM1	ENSG00000148356	9:127497334-127501009:+	0.20161296	0.039215741	0.001248
549	LRWD1	ENSG00000161036	7:102469882-102472217:+	0.16923088	0.040404146	0.046142
550	LSM14B	ENSG00000149657	20:62126439-62127584:+	0.25925934	0.04255327	0.00228
551	LSM14B	ENSG00000149657	20:62127701-62129784:+	0.32967041	0.048780584	0.001661
552	LSM7	ENSG00000130332	19:2324192-2328386:-	0.36521746	0.119266157	0.016766
553	LTV1	ENSG00000135521	6:143858007-143860425:+	0.29850752	0.090909169	0.006977
554	LTV1	ENSG00000135521	6:143860553-143862103:+	0.32558148	0.118644188	0.045871
555	LZTFL1	ENSG00000163818	3:45827459-45828438:-	0.19259268	0.045833424	0.018343
556	MAEA	ENSG00000090316	4:1322503-1327626:+	0.11711719	0.015957507	0.00707
557	MAEA	ENSG00000090316	4:1338617-1339073:+	0.12068972	0.03272733	0.019769
558	MAGED2	ENSG00000102316	X:54815690-54815880:+	0.24803152	0.153134678	0.049416
559	MAN1B1	ENSG00000177239	9:137106315-137106688:+	0.11504433	0.026881792	0.032681
560	MAN2B2	ENSG00000013288	4:6617492-6619926:+	0.51190482	0.193181949	0.032548
561	MAN2B2	ENSG00000013288	4:6620044-6621187:+	0.32203396	0.06000008	0.001202
562	MAP2K7	ENSG00000076984	19:7910373-7910452:+	0.10638304	0.015354342	0.035377
563	MAP3K7	ENSG00000135341	6:90550549-90552048:-	0.56338032	0.166666758	0.001614
564	MAP4	ENSG00000047849	3:47852938-47855247:-	0.33333343	0.1052633	0.046421
565	MAP7	ENSG00000135525	6:136377868-136388392:-	0.38461549	0.118644227	0.049487
566	MAPK11	ENSG00000185386	22:50266611-50266933:-	0.18539331	0.072674484	0.033372
567	MAPK14	ENSG00000112062	6:36100267-36102570:+	0.33928581	0.091836877	0.033958
568	MAPRE3	ENSG00000084764	2:27024252-27025582:+	0.24230774	0.108359209	0.043656
569	MAPT	ENSG00000186868	17:45971945-45974384:+	0.15000039	0.008622288	0.040906
570	MAST1	ENSG00000105613	19:12866102-12866652:+	0.52777786	0.071749082	0.006887
571	MAST3	ENSG00000099308	19:18121922-18122672:+	0.19298258	0.030769332	0.017036
572	MAST3	ENSG00000099308	19:18122751-18123216:+	0.14516137	0.03311266	0.021757
573	MAST3	ENSG00000099308	19:18128458-18128865:+	0.37278116	0.095420004	0.019927
574	MATR3	ENSG00000015479	5:139314736-139315696:+	0.23265744	0.090981685	0.001066
575	MBTPS1	ENSG00000140943	16:84065767-84066488:-	0.1028807	0.030188725	0.016225
576	MCM3AP	ENSG00000160294	21:46241017-46242801:-	0.12536446	0.060606098	0.044602
577	MCOLN1	ENSG00000090674	19:7527960-7528157:+	0.11290332	0.013605523	0.017584
578	MCOLN1	ENSG00000090674	19:7528257-7528596:+	0.14782617	0.004637176	0.000225
579	MCOLN1	ENSG00000090674	19:7528703-7528820:+	0.20833344	0.023809644	0.012923
580	MED10	ENSG00000133398	5:6372601-6374323:-	0.12851409	0.058536623	0.035555
581	MED24	ENSG00000008838	17:40023395-40026155:-	0.17197459	0.045454612	0.01193
582	MED24	ENSG00000008838	17:40027034-40027382:-	0.17721524	0.030434831	0.000679
583	MED24	ENSG00000008838	17:40027465-40027908:-	0.2064517	0.061320853	0.033011
584	MED24	ENSG00000008838	17:40027946-40028825:-	0.22516563	0.050000075	0.00463
585	MED25	ENSG00000104973	19:49832415-49834985:+	0.10328643	0.024691402	0.008082
586	MED25	ENSG00000104973	19:49828547-49828969:+	0.13661207	0.041237171	0.019651
587	MFSD10	ENSG00000109736	4:2931150-2931226:-	0.16666672	0.035971292	0.007866

S/N	Gene Affected	ENSEMBL_ID	Position of retained intron	IR ratio AD	IR ratio Control	*p*- value
588	MFSD12	ENSG00000161091	19:3544732-3544808:-	0.33720939	0.098214409	0.025515
589	MINK1	ENSG00000141503	17:4893067-4893285:+	0.50961543	0.076555126	0.000284
590	MINK1	ENSG00000141503	17:4893309-4893433:+	0.53030308	0.108910994	0.000608
591	MINK1	ENSG00000141503	17:4893597-4893987:+	0.27380956	0.109090969	0.011181
592	MINK1	ENSG00000141503	17:4894311-4894524:+	0.10851069	0.030769273	0.008267
593	MINK1	ENSG00000141503	17:4895832-4896002:+	0.20030584	0.040662678	1.73E-06
594	MLLT6	ENSG00000275023	17:38715828-38716366:+	0.3826367	0.152439099	0.010581
595	MLLT6	ENSG00000275023	17:38717953-38719516:+	0.10948911	0.0333334	0.043247
596	MLLT6	ENSG00000275023	17:38707540-38707762:+	0.11382119	0.015625078	0.01279
597	MORF4L2	ENSG00000123562	X:103685260-103686630:-	0.12571433	0.018579987	1.68E-05
598	MORF4L2	ENSG00000123562	X:103677051-103685170:-	0.12461065	0.034869304	0.017982
599	MPHOSPH6	ENSG00000135698	16:82149403-82151423:-	0.2021661	0.086956565	0.011979
600	MPP1	ENSG00000130830	X:154781802-154783426:-	0.11182113	0.047297343	0.04453
601	MPP1	ENSG00000130830	X:154783507-154784027:-	0.12264156	0.026966329	0.001696
602	MPP1	ENSG00000130830	X:154784108-154785050:-	0.10344833	0.021844699	0.003923
603	MPRIP	ENSG00000133030	17:17147387-17150143:+	0.17788465	0.055016224	0.002334
604	MPV17	ENSG00000115204	2:27311951-27312213:-	0.1920375	0.093181863	0.026343
605	MPV17	ENSG00000115204	2:27312246-27312493:-	0.24945773	0.127572074	0.035841
606	MRAS	ENSG00000158186	3:138348325-138372865:+	0.17857165	0.009514208	0.008209
607	MRFAP1L1	ENSG00000178988	4:6708643-6709230:-	0.35361845	0.175213749	0.022041
608	MROH1	ENSG00000179832	8:144260374-144260676:+	0.50602414	0.152318014	0.012184
609	MROH1	ENSG00000179832	8:144261041-144261113:+	0.12903237	0.017045533	0.014307
610	MROH1	ENSG00000179832	8:144192401-144199121:+	0.13888907	0.016535461	0.047479
611	MRPL2	ENSG00000112651	6:43055618-43055896:-	0.10000006	0.028368848	0.029648
612	MRPL38	ENSG00000204316	17:75901273-75901711:-	0.2975207	0.125506139	0.016316
613	MRPL39	ENSG00000154719	21:25585754-25588834:-	0.24657539	0.096774258	0.01621
614	MRPL4	ENSG00000105364	19:10258522-10258608:+	0.27941185	0.085858712	0.028804
615	MRPL58	ENSG00000167862	17:75012872-75017077:+	0.23809533	0.060000105	0.026427
616	MRPS26	ENSG00000125901	20:3046737-3047734:+	0.12500003	0.046683082	0.010551
617	MRPS5	ENSG00000144029	2:95101723-95104639:-	0.34219271	0.145383133	9.20E-05
618	MSH6	ENSG00000116062	2:47805707-47806203:+	0.03989364	0.137440818	0.002578
619	MSL3	ENSG00000005302	X:11760937-11761499:+	0.12500007	0.032653125	0.02338
620	MYH7B	ENSG00000078814	20:34997640-34998294:+	0.12676071	0.011261705	0.018615
621	MYH9	ENSG00000100345	22:36305102-36305929:-	0.11111118	0.026455092	0.023939
622	MYL5	ENSG00000215375	4:680018-680508:+	0.12345693	0.016284924	0.048862
623	MYO15B	ENSG00000266714	17:75620357-75620466:+	0.00532082	0.102272876	0.003332
624	MYO9B	ENSG00000099331	19:17162466-17162987:+	0.20000012	0.03333349	0.040907
625	MYO9B	ENSG00000099331	19:17181040-17183828:+	0.02162168	0.129032405	0.015752
626	MYOM2	ENSG00000036448	8:2123365-2123554:+	0.24786336	0.040000106	0.008118
627	MYPOP	ENSG00000176182	19:45901825-45902569:-	0.21212139	0.032258228	0.04951
628	MZT2B	ENSG00000152082	2:130183966-130190468:+	0.19753093	0.074074147	0.049938
629	NAA10	ENSG00000102030	X:153930847-153932070:-	0.30241938	0.147410399	0.004687
630	NAA10	ENSG00000102030	X:153932115-153932315:-	0.13194448	0.050125356	0.017699
631	NAA20	ENSG00000173418	20:20026919-20032507:+	0.11720701	0.046017735	0.014225
632	NAA38	ENSG00000183011	17:7856843-7856984:-	0.17142865	0.032467605	0.008051
633	NABP1	ENSG00000173559	2:191683804-191684229:+	0.15384644	0.014521685	0.046664
634	NACAD	ENSG00000136274	7:45081672-45081754:-	0.1488971	0.045634962	0.003287
635	NAE1	ENSG00000159593	16:66823600-66824854:-	0.12015509	0.043189422	0.033959
636	NAE1	ENSG00000159593	16:66824885-66826522:-	0.1115703	0.040000055	0.04366

S/N	Gene Affected	ENSEMBL_ID	Position of retained intron	IR ratio AD	IR ratio Control	*p*- value
637	NARF	ENSG00000141562	17:82478918-82481081:+	0.20253169	0.035573167	0.000225
638	NAT14	ENSG00000090971	19:55485780-55486407:+	0.21428578	0.076087039	0.036593
639	NBEAL2	ENSG00000160796	3:47003309-47003815:+	0.32352975	0.032460186	0.045159
640	NCAPH2	ENSG00000025770	22:50521036-50521542:+	0.31914898	0.123287742	0.011718
641	NCKIPSD	ENSG00000213672	3:48678969-48679054:-	0.16425125	0.050100248	0.005391
642	NDUFA10	ENSG00000130414	2:240011696-240014738:-	0.44269343	0.187317134	0.001114
643	NDUFB2	ENSG00000090266	7:140697391-140702865:+	0.3611112	0.123456927	0.049876
644	NELFE	ENSG00000204356	6:31953831-31954079:-	0.42967247	0.258227902	0.035346
645	NEURL4	ENSG00000215041	17:7323123-7323484:-	0.42718455	0.100000126	0.007665
646	NICN1	ENSG00000145029	3:49424874-49424948:-	0.17525779	0.030075239	0.000902
647	NICN1	ENSG00000145029	3:49425438-49425882:-	0.17500007	0.057692378	0.030484
648	NICN1	ENSG00000145029	3:49425996-49426251:-	0.21186449	0.041176552	0.006762
649	NIPSNAP3B	ENSG00000165028	9:104772908-104772996:+	0.23529425	0.023622152	0.006563
650	NMI	ENSG00000123609	2:151275670-151275757:-	0.18965541	0.009046308	0.00793
651	NMT1	ENSG00000136448	17:45103876-45104858:+	0.13871639	0.046992518	0.004234
652	NONO	ENSG00000147140	X:71298816-71299941:+	0.10410096	0.033670055	0.000577
653	NONO	ENSG00000147140	X:71297938-71298468:+	0.23292472	0.107476675	0.005989
654	NOP56	ENSG00000101361	20:2655746-2655933:+	0.11041014	0.019002411	0.000829
655	NPAS2	ENSG00000170485	2:100988276-100990255:+	0.20652182	0.061403603	0.035881
656	NPR2	ENSG00000159899	9:35805670-35805829:+	0.12328779	0.017060387	0.046376
657	NPR2	ENSG00000159899	9:35800157-35800388:+	0.15384629	0.017619743	0.029682
658	NPRL2	ENSG00000114388	3:50348410-50348526:-	0.18274119	0.058558634	0.026035
659	NPRL2	ENSG00000114388	3:50349010-50349385:-	0.27464793	0.082353005	0.004127
660	NPTX2	ENSG00000106236	7:98619859-98624921:+	0.12658236	0.023529477	0.015552
661	NR1D1	ENSG00000126368	17:40094122-40094934:-	0.10762335	0.042857176	0.014837
662	NR1D1	ENSG00000126368	17:40096087-40096442:-	0.12500005	0.036529735	0.019605
663	NR4A2	ENSG00000153234	2:156326000-156326149:-	0.13114769	0.014330738	0.034551
664	NRBP1	ENSG00000115216	2:27435227-27435722:+	0.5789474	0.153846287	0.005153
665	NRBP1	ENSG00000115216	2:27435227-27436752:+	0.17587942	0.069478943	0.003835
666	NRBP1	ENSG00000115216	2:27435746-27436752:+	0.75630254	0.192307821	0.001297
667	NSUN2	ENSG00000037474	5:6600232-6602460:-	0.19306935	0.05990789	0.007611
668	NUP50	ENSG00000093000	22:45168246-45171599:+	0.20930246	0.030303172	0.030517
669	NUP50	ENSG00000093000	22:45171683-45175893:+	0.16666674	0.033898389	0.016614
670	NUP85	ENSG00000125450	17:75235201-75235577:+	0.13705588	0.045454604	0.026463
671	OCIAD1	ENSG00000109180	4:48851975-48860724:+	0.31372552	0.115839293	0.001514
672	OGFOD1	ENSG00000087263	16:56462634-56466151:+	0.11428581	0.017543949	0.032512
673	OGFOD1	ENSG00000087263	16:56466967-56467164:+	0.38562097	0.175532017	0.047826
674	OGFOD1	ENSG00000087263	16:56467293-56467904:+	0.17391313	0.037634483	0.012438
675	OGFOD1	ENSG00000087263	16:56468018-56470002:+	0.18421058	0.052401797	0.006214
676	OGFOD1	ENSG00000087263	16:56470082-56470486:+	0.19047626	0.030769293	0.001821
677	OGFOD1	ENSG00000087263	16:56470791-56474827:+	0.14358979	0.04587161	0.013576
678	OGFOD1	ENSG00000087263	16:56474950-56475506:+	0.12000004	0.032258109	0.007356
679	OGFOD1	ENSG00000087263	16:56453408-56458547:+	0.10810821	0.017699207	0.040947
680	ORC3	ENSG00000135336	6:87656982-87657920:+	0.201878	0.077777859	0.042723
681	OSBPL7	ENSG00000006025	17:47816482-47816562:-	0.2222225	0.018394126	0.029631
682	OSTC	ENSG00000198856	4:108650794-108655563:+	0.16564425	0.020134303	0.003977
683	PACSIN2	ENSG00000100266	22:42876333-42879047:-	0.27659586	0.058823654	0.023135
684	PACSIN2	ENSG00000100266	22:42879169-42880616:-	0.56097563	0.095652329	0.004207
685	PACSIN2	ENSG00000100266	22:42880622-42882183:-	0.43243252	0.053571597	0.008386

S/N	Gene Affected	ENSEMBL_ID	Position of retained intron	IR ratio AD	IR ratio Control	*p*- value
686	PAK1IP1	ENSG00000111845	6:10703457-10704506:+	0.13461546	0.034090979	0.026833
687	PAK1IP1	ENSG00000111845	6:10707514-10708952:+	0.12751684	0.039325908	0.036175
688	PARP8	ENSG00000151883	5:50826803-50827943:+	0.12048202	0.020000075	0.020342
689	PARP8	ENSG00000151883	5:50828056-50828311:+	0.12962971	0.034965117	0.046838
690	PCBP4	ENSG00000090097	3:51958349-51958789:-	0.13005276	0.048543734	0.017108
691	PCED1A	ENSG00000132635	20:2836314-2838231:-	0.35173826	0.164634185	0.002007
692	PCGF1	ENSG00000115289	2:74505190-74505338:-	0.2396695	0.075268925	0.040835
693	PCIF1	ENSG00000100982	20:45939339-45940474:+	0.21678327	0.074324398	0.023234
694	PCM1	ENSG00000078674	8:17953186-17955469:+	0.15306128	0.044586056	0.021645
695	PCNT	ENSG00000160299	21:46430657-46431528:+	0.28169023	0.075000116	0.025889
696	PCYT2	ENSG00000185813	17:81906177-81906463:-	0.15107918	0.047138097	0.009326
697	PDAP1	ENSG00000106244	7:99396740-99397861:-	0.15893274	0.038543921	7.92E-06
698	PDE7A	ENSG00000205268	8:65724351-65724776:-	0.10937508	0.013435056	0.026396
699	PDHA1	ENSG00000131828	X:19354583-19355348:+	0.27528094	0.100358482	0.003789
700	PDHA1	ENSG00000131828	X:19353173-19354490:+	0.20802924	0.087939753	0.019052
701	PDXK	ENSG00000160209	21:43743807-43746078:+	0.16886546	0.048543717	0.000151
702	PDZD4	ENSG00000067840	X:153806840-153807278:-	0.18402784	0.049896118	0.007932
703	PER2	ENSG00000132326	2:238249212-238250550:-	0.25806485	0.018394141	0.028721
704	PHF1	ENSG00000112511	6:33413557-33413735:+	0.28571432	0.088560936	0.000876
705	PHF1	ENSG00000112511	6:33413831-33414040:+	0.19653182	0.073482471	0.005116
706	PHF20	ENSG00000025293	20:35939108-35940863:+	0.25423748	0.025316616	0.021223
707	PHIP	ENSG00000146247	6:78969917-78970048:-	0.16981159	0.00875913	0.013348
708	PIAS4	ENSG00000105229	19:4033580-4037373:+	0.51428582	0.117647236	0.018198
709	PIGO	ENSG00000165282	9:35093580-35093900:-	0.11111124	0.011036469	0.023053
710	PIGS	ENSG00000087111	17:28556972-28558475:-	0.15000007	0.034013679	0.018264
711	PIGT	ENSG00000124155	20:45420693-45421382:+	0.14239487	0.03641461	0.002943
712	PIGT	ENSG00000124155	20:45421583-45424215:+	0.11708864	0.033950656	0.005549
713	PIH1D1	ENSG00000104872	19:49447099-49447337:-	0.29285719	0.063380335	0.000155
714	PIH1D1	ENSG00000104872	19:49447908-49448000:-	0.19930075	0.050131979	0.00172
715	PIK3R1	ENSG00000145675	5:68290834-68292258:+	0.25454555	0.032374202	0.005027
716	PISD	ENSG00000241878	22:31619836-31620552:-	0.19339628	0.049833939	0.002459
717	PISD	ENSG00000241878	22:31620713-31620995:-	0.12413804	0.024793473	0.030297
718	PITPNB	ENSG00000180957	22:27853663-27854853:-	0.18681324	0.042553247	0.002548
719	PITPNB	ENSG00000180957	22:27853663-27858386:-	0.18656724	0.046948431	0.012862
720	PITPNB	ENSG00000180957	22:27854939-27858386:-	0.14743595	0.043103504	0.014179
721	PKDCC	ENSG00000162878	2:42053361-42054035:+	0.17741948	0.036144705	0.047823
722	PKP4	ENSG00000144283	2:158676867-158678580:+	0.04312019	0.213750149	0.000187
723	PLA2G15	ENSG00000103066	16:68255037-68255281:+	0.11320775	0.008900394	0.025093
724	PLA2G6	ENSG00000184381	22:38113654-38115526:-	0.10606067	0.015789536	0.0095
725	PLA2G6	ENSG00000184381	22:38120909-38123094:-	0.19047628	0.056910674	0.049719
726	PLA2G6	ENSG00000184381	22:38123258-38126370:-	0.22222232	0.059829168	0.03251
727	PLA2G6	ENSG00000184381	22:38126449-38129453:-	0.5555556	0.105263307	0.005035
728	PLCB4	ENSG00000101333	20:9473365-9478920:+	0.26315806	0.044117818	0.04084
729	PLD2	ENSG00000129219	17:4809205-4809297:+	0.14864879	0.014524395	0.022998
730	PLD2	ENSG00000129219	17:4809363-4809492:+	0.18032802	0.017060395	0.021143
731	PLD2	ENSG00000129219	17:4817055-4817145:+	0.19718322	0.031915016	0.024931
732	PLD2	ENSG00000129219	17:4818385-4818493:+	0.25210097	0.030075293	0.005007
733	PLOD3	ENSG00000106397	7:101210161-101210330:-	0.2142859	0.039604109	0.039177
734	PLRG1	ENSG00000171566	4:154536744-154537285:-	0.12068968	0.056179809	0.032259

S/N	Gene Affected	ENSEMBL_ID	Position of retained intron	IR ratio AD	IR ratio Control	*p*- value
735	PLXNB1	ENSG00000164050	3:48410558-48410867:-	0.10580208	0.036363686	0.026313
736	PLXNB1	ENSG00000164050	3:48412009-48412237:-	0.13404509	0.060344856	0.007987
737	PLXNB1	ENSG00000164050	3:48415347-48415582:-	0.18750004	0.075581458	0.031875
738	PLXNB3	ENSG00000198753	X:153774344-153774419:+	0.11355321	0.011313741	0.018324
739	PLXNB3	ENSG00000198753	X:153777688-153777947:+	0.23513878	0.085889647	0.021695
740	PLXND1	ENSG00000004399	3:129567805-129569842:-	0.36363646	0.112676203	0.042642
741	PMPCB	ENSG00000105819	7:103312131-103312206:+	0.51972557	0.208151422	1.61E-05
742	PNKP	ENSG00000039650	19:49862463-49862537:-	0.13333346	0.010967405	0.014073
743	PNPLA6	ENSG00000032444	19:7542067-7542560:+	0.44102572	0.137614793	0.012762
744	PNPLA6	ENSG00000032444	19:7557284-7558849:+	0.12142859	0.037444959	0.000636
745	POLI	ENSG00000101751	18:54271485-54273925:+	0.12987026	0.017913408	0.048376
746	POLR2B	ENSG00000047315	4:57023580-57023661:+	0.15883672	0.064377725	0.012485
747	POLR2H	ENSG00000163882	3:184363565-184364965:+	0.25339372	0.083682079	0.009974
748	POLR2I	ENSG00000105258	19:36114412-36114658:-	0.10777629	0.040332182	0.009894
749	PORCN	ENSG00000102312	X:48514141-48514239:+	0.31111127	0.007457085	0.000342
750	PORCN	ENSG00000102312	X:48514365-48514524:+	0.10256421	0.010435002	0.021616
751	PPARD	ENSG00000112033	6:35421958-35423945:+	0.1610739	0.04624285	0.026784
752	PPP1R12C	ENSG00000125503	19:55094798-55095290:-	0.15492961	0.064516169	0.013699
753	PPP1R21	ENSG00000162869	2:48505596-48507268:+	0.22277234	0.073275939	0.018254
754	PPP1R37	ENSG00000104866	19:45146049-45146389:+	0.10256417	0.014285765	0.004714
755	PPP1R3F	ENSG00000049769	X:49282063-49285833:+	0.17910453	0.038567543	0.001307
756	PPP2R2B	ENSG00000156475	5:147081136-147081256:-	0.15789482	0.027027121	0.016538
757	PPP5C	ENSG00000011485	19:46376574-46383410:+	0.12918665	0.04558409	0.017949
758	PPP6R1	ENSG00000105063	19:55241391-55241476:-	0.18954258	0.016304421	0.001706
759	PPP6R2	ENSG00000100239	22:50441026-50443865:+	0.38095242	0.142857204	0.003365
760	PPP6R2	ENSG00000100239	22:50444117-50444198:+	0.16450221	0.038167988	0.002227
761	PPP6R2	ENSG00000100239	22:50436452-50436987:+	0.17213121	0.053030377	0.028367
762	PPP6R2	ENSG00000100239	22:50436452-50437505:+	0.2395834	0.042682993	0.001504
763	PPP6R2	ENSG00000100239	22:50437068-50437505:+	0.13500005	0.043715905	0.02505
764	PQBP1	ENSG00000102103	X:48902517-48902731:+	0.13390316	0.057569332	0.018197
765	PRAF2	ENSG00000243279	X:49072650-49073808:-	0.10833341	0.030172479	0.042798
766	PRKCD	ENSG00000163932	3:53181732-53183120:+	0.11320769	0.009108524	0.01536
767	PRKCSH	ENSG00000130175	19:11447789-11448221:+	0.28745647	0.103053474	0.000299
768	PRKDC	ENSG00000253729	8:47778660-47778727:-	0.16525433	0.046025198	0.037341
769	PRKRA	ENSG00000180228	2:178444500-178447504:-	0.10232563	0.036809861	0.034025
770	PRMT1	ENSG00000126457	19:49686726-49688161:+	0.18683654	0.038397352	8.02E-07
771	PRMT2	ENSG00000160310	21:46649739-46658744:+	0.13967614	0.054919944	0.008636
772	PRPF31	ENSG00000105618	19:54129185-54129271:+	0.18627455	0.07657663	0.025885
773	PRRT1	ENSG00000204314	6:32149722-32150367:-	0.10126594	0.01117326	0.021538
774	PRUNE2	ENSG00000106772	9:76614600-76619339:-	0.51401874	0.091811544	0.000992
775	PRUNE2	ENSG00000106772	9:76619387-76624451:-	0.45205484	0.112211335	0.002867
776	PRUNE2	ENSG00000106772	9:76624490-76629191:-	0.2285715	0.049505025	0.004455
777	PRUNE2	ENSG00000106772	9:76637549-76638185:-	0.12605049	0.021951271	0.003137
778	PRUNE2	ENSG00000106772	9:76638288-76641971:-	0.16374276	0.040000118	0.048066
779	PRUNE2	ENSG00000106772	9:76638288-76644738:-	0.16279081	0.004299232	0.000432
780	PSMB10	ENSG00000205220	16:67934948-67935419:-	0.14176248	0.046296333	0.00433
781	PSMB8	ENSG00000204264	6:32841735-32842133:-	0.11176478	0.02797211	0.046752
782	PSMC3IP	ENSG00000131470	17:42573364-42573477:-	0.00627073	0.116666942	0.009521
783	PSMD11	ENSG00000108671	17:32477583-32479250:+	0.11881191	0.049079794	0.024957

S/N	Gene Affected	ENSEMBL_ID	Position of retained intron	IR ratio AD	IR ratio Control	*p*- value
784	PSMD5	ENSG00000095261	9:120821464-120824493:-	0.18181824	0.067114166	0.042012
785	PSMD6	ENSG00000163636	3:64022523-64023274:-	0.23888895	0.057471335	0.00369
786	PSMG4	ENSG00000180822	6:3263759-3267590:+	0.30188687	0.048780573	0.002312
787	PTCD3	ENSG00000132300	2:86136562-86136981:+	0.08125002	0.167647112	0.022425
788	PTGDS	ENSG00000107317	9:136979299-136979945:+	0.25297969	0.126837716	0.022515
789	PTH1R	ENSG00000160801	3:46901480-46901765:+	0.35576934	0.094736986	0.027222
790	PTH1R	ENSG00000160801	3:46901860-46902525:+	0.28846167	0.057142989	0.017499
791	PTK2	ENSG00000169398	8:140669735-140674297:-	0.29646024	0.125926023	0.039166
792	PTOV1	ENSG00000104960	19:49855077-49856974:+	0.31258114	0.114441526	0.021437
793	PTPRK	ENSG00000152894	6:127998904-128005083:-	0.05586598	0.220779358	0.011417
794	PTPRK	ENSG00000152894	6:128003232-128005083:-	0.10606073	0.020958155	0.036652
795	PTPRN	ENSG00000054356	2:219300977-219301587:-	0.11049732	0.019230831	0.011342
796	QARS	ENSG00000172053	3:49099253-49099343:-	0.16587687	0.028846231	0.008001
797	QARS	ENSG00000172053	3:49099431-49099509:-	0.10429454	0.027397318	0.026179
798	QARS	ENSG00000172053	3:49099647-49099760:-	0.19580427	0.053921645	0.01531
799	RAB24	ENSG00000169228	5:177302178-177302396:-	0.5978261	0.287128776	0.002443
800	RAB3GAP1	ENSG00000115839	2:135167713-135168544:+	0.20338992	0.06472503	0.049861
801	RAB40B	ENSG00000141542	17:82658134-82658490:-	0.14285719	0.034168605	0.001392
802	RAD50	ENSG00000113522	5:132609209-132609282:+	0.13953496	0.038596563	0.024687
803	RAD51D	ENSG00000185379	17:35103324-35103453:-	0.107143	0.013605128	0.047701
804	RAD54B	ENSG00000197275	8:94378380-94378567:-	0.16216243	0.015328446	0.043618
805	RAE1	ENSG00000101146	20:57373581-57373662:+	0.12380957	0.014492794	0.000467
806	RAF1	ENSG00000132155	3:12584657-12584846:-	0.12181823	0.027472569	0.002373
807	RALGPS1	ENSG00000136828	9:127214842-127218739:+	0.60714292	0.238095402	0.048026
808	RANBP10	ENSG00000141084	16:67729484-67729679:-	0.13157915	0.014914148	0.047255
809	RANBP9	ENSG00000010017	6:13632521-13634430:-	0.13725493	0.042553225	0.001624
810	RBBP7	ENSG00000102054	X:16852628-16852748:-	0.12980772	0.06368334	0.035452
811	RBBP7	ENSG00000102054	X:16852875-16853681:-	0.14634149	0.059183702	0.003956
812	RBFOX2	ENSG00000100320	22:35744249-35745922:-	0.10294128	0.020618625	0.036812
813	RBM10	ENSG00000182872	X:47180037-47180211:+	0.29577476	0.061538607	0.024909
814	RBM10	ENSG00000182872	X:47180506-47181214:+	0.23308276	0.051094959	0.002928
815	RBM3	ENSG00000102317	X:48577109-48577464:+	0.38976383	0.060773555	5.99E-05
816	RBM33	ENSG00000184863	7:155774647-155774992:+	0.1413045	0.017335567	0.043841
817	RBM5	ENSG00000003756	3:50110770-50113382:+	0.2509579	0.121134088	0.040748
818	RBM5	ENSG00000003756	3:50106864-50107481:+	0.19101128	0.058139587	0.004309
819	RCHY1	ENSG00000163743	4:75490701-75491610:-	0.1726619	0.059190072	0.004238
820	RCHY1	ENSG00000163743	4:75491637-75491723:-	0.14028782	0.054380715	0.027042
821	RELL2	ENSG00000164620	5:141639649-141639919:+	0.39500005	0.109422578	0.001808
822	RELL2	ENSG00000164620	5:141640295-141640411:+	0.3650794	0.129629703	0.004511
823	RELL2	ENSG00000164620	5:141640445-141640670:+	0.46892659	0.096774262	4.99E-05
824	RELN	ENSG00000189056	7:103472908-103478388:-	0.4047621	0.044776339	0.022434
825	RELN	ENSG00000189056	7:103478394-103482872:-	0.20833371	0.00919711	0.023395
826	RENBP	ENSG00000102032	X:153941653-153941949:-	0.17567582	0.013779558	0.013503
827	RGS12	ENSG00000159788	4:3417541-3420641:+	0.4536083	0.18487407	0.039938
828	RHPN1	ENSG00000158106	8:143379078-143379314:+	0.26605514	0.052238908	0.008269
829	RHPN1	ENSG00000158106	8:143380783-143381267:+	0.37931042	0.125541258	0.025578
830	RHPN1	ENSG00000158106	8:143381344-143381571:+	0.44134084	0.148305201	0.01227
831	RIOK3	ENSG00000101782	18:23477086-23477178:+	0.33714291	0.159793901	0.049887
832	RIPPLY2	ENSG00000203877	6:83854161-83857241:+	0.19230776	0.057613247	0.022292

S/N	Gene Affected	ENSEMBL_ID	Position of retained intron	IR ratio AD	IR ratio Control	*p*- value
833	RNF123	ENSG00000164068	3:49713587-49713737:+	0.12328787	0.009511576	0.026929
834	RNF123	ENSG00000164068	3:49713825-49713909:+	0.12500013	0.012795297	0.023972
835	RNF123	ENSG00000164068	3:49704756-49704983:+	0.21153865	0.015805966	0.015222
836	RNF126	ENSG00000070423	19:650296-651610:-	0.27350435	0.085526413	0.02273
837	RNF213	ENSG00000173821	17:80354166-80354440:+	0.02857156	0.210526537	0.029211
838	RNF216P1	ENSG00000196204	7:4995582-4996609:+	0.1935486	0.012262749	0.010744
839	RNF25	ENSG00000163481	2:218668146-218668238:-	0.17241388	0.050633001	0.040791
840	RNMT	ENSG00000101654	18:13754147-13759941:+	0.2000001	0.059259362	0.049469
841	RP9P	ENSG00000205763	7:32917542-32918310:-	0.32758628	0.100840441	0.018872
842	RPGR	ENSG00000156313	X:38276772-38290958:-	0.29411776	0.069444586	0.031398
843	RPL34	ENSG00000109475	4:108625253-108630020:+	0.07377057	0.227848249	0.042167
844	RPN2	ENSG00000118705	20:37236709-37238401:+	0.17142867	0.050000101	0.046109
845	RPS6KB1	ENSG00000108443	17:59931722-59934169:+	0.20535723	0.038835054	0.015342
846	RPS6KB1	ENSG00000108443	17:59934260-59934433:+	0.20833341	0.045454647	0.018556
847	RPS6KB1	ENSG00000108443	17:59934524-59935192:+	0.36486497	0.109091069	0.048942
848	RPUSD3	ENSG00000156990	3:9839171-9840183:-	0.17777787	0.046666756	0.031612
849	RPUSD3	ENSG00000156990	3:9840307-9840531:-	0.17567576	0.043478346	0.026585
850	RPUSD3	ENSG00000156990	3:9840616-9840697:-	0.18823539	0.042944876	0.021618
851	RRAGB	ENSG00000083750	X:55757331-55758245:+	0.1854305	0.056074809	0.001721
852	RRP1	ENSG00000160214	21:43802387-43803511:+	0.24615391	0.089172057	0.029809
853	RSBN1L	ENSG00000187257	7:77778446-77778529:+	0.20270277	0.060000095	0.032335
854	RTEL1	ENSG00000258366	20:63693283-63694371:+	0.16279081	0.028169151	0.046866
855	RTEL1	ENSG00000258366	20:63694488-63694740:+	0.3372094	0.059701625	0.009224
856	RTEL1	ENSG00000258366	20:63695221-63695327:+	0.18965527	0.042857277	0.048726
857	RTEL1	ENSG00000258366	20:63688605-63689054:+	0.55813968	0.125000255	0.044465
858	RTEL1	ENSG00000258366	20:63691837-63692804:+	0.20779232	0.032258212	0.031436
859	RTKN	ENSG00000114993	2:74427273-74427423:-	0.19910017	0.084210587	0.027427
860	RTKN	ENSG00000114993	2:74428737-74428847:-	0.13151936	0.025974096	0.013344
861	RUBCN	ENSG00000145016	3:197695981-197696953:-	0.11214962	0.020408267	0.04927
862	RUBCN	ENSG00000145016	3:197697049-197699186:-	0.42553202	0.065934221	0.008614
863	RUBCN	ENSG00000145016	3:197699231-197700612:-	0.3061226	0.027523071	0.007245
864	SAFB	ENSG00000160633	19:5648043-5648988:+	0.23192023	0.122715452	0.035019
865	SAFB	ENSG00000160633	19:5664439-5667045:+	0.11046515	0.050473223	0.041937
866	SAFB	ENSG00000160633	19:5667450-5667819:+	0.14805199	0.04336739	0.003667
867	SAFB2	ENSG00000130254	19:5590408-5591747:-	0.16027879	0.046729029	0.009938
868	SAFB2	ENSG00000130254	19:5592887-5593890:-	0.12640453	0.033333377	0.004184
869	SAMD14	ENSG00000167100	17:50116090-50117406:-	0.17307704	0.042553286	0.042658
870	SAT2	ENSG00000141504	17:7626793-7626942:-	0.3146552	0.155594452	0.007306
871	SATB2	ENSG00000119042	2:199456096-199457338:-	0.18181852	0.014521698	0.042901
872	SBF1	ENSG00000100241	22:50456673-50459254:-	0.11200009	0.028688598	0.042317
873	SBF1	ENSG00000100241	22:50459666-50459951:-	0.11764711	0.043165521	0.038331
874	SBF1	ENSG00000100241	22:50460712-50461158:-	0.12903231	0.016877687	0.001435
875	SCRIB	ENSG00000180900	8:143791440-143791665:-	0.3161765	0.138888948	0.01381
876	SCRIB	ENSG00000180900	8:143791740-143791875:-	0.25443792	0.071428648	0.007493
877	SCRN2	ENSG00000141295	17:47838450-47838530:-	0.19540239	0.040000095	0.01732
878	SDAD1	ENSG00000198301	4:75973391-75974075:-	0.12871296	0.022222286	0.010879
879	SEMA3B	ENSG00000012171	3:50274934-50275011:+	0.17910453	0.070512884	0.03035
880	SEMA6B	ENSG00000167680	19:4552639-4554387:-	0.12745103	0.041095934	0.012665
881	SEPT7P2	ENSG00000214765	7:45728313-45728831:-	0.11764723	0.013795587	0.045026

S/N	Gene Affected	ENSEMBL_ID	Position of retained intron	IR ratio AD	IR ratio Control	*p*- value
882	SF3A2	ENSG00000104897	19:2245555-2246752:+	0.15458941	0.069565265	0.042109
883	SF3A2	ENSG00000104897	19:2246802-2246881:+	0.16521743	0.062992173	0.01538
884	SFT2D1	ENSG00000198818	6:166331407-166342418:-	0.11111134	0.008759118	0.029164
885	SGCE	ENSG00000127990	7:94585515-94587777:-	0.21031752	0.082914657	0.040827
886	SGSM2	ENSG00000141258	17:2362905-2362988:+	0.19718318	0.056497263	0.026181
887	SGSM3	ENSG00000100359	22:40406662-40407016:+	0.20754722	0.076923139	0.017993
888	SGSM3	ENSG00000100359	22:40408120-40408276:+	0.13600006	0.031802176	0.007462
889	SGSM3	ENSG00000100359	22:40409018-40409249:+	0.43598619	0.240131657	0.047806
890	SGSM3	ENSG00000100359	22:40400813-40402138:+	0.27868864	0.046875146	0.019952
891	SGSM3	ENSG00000100359	22:40401675-40402138:+	0.13173658	0.012244944	0.000596
892	SGSM3	ENSG00000100359	22:40409525-40409681:+	0.22006475	0.09383382	0.006096
893	SGTA	ENSG00000104969	19:2757782-2759256:-	0.15776701	0.077889482	0.023462
894	SGTA	ENSG00000104969	19:2759294-2761459:-	0.13597737	0.061764744	0.029196
895	SH3GLB2	ENSG00000148341	9:129010209-129010669:-	0.24458209	0.074902796	0.003991
896	SH3GLB2	ENSG00000148341	9:129010693-129014410:-	0.32624119	0.119632021	0.033307
897	SHANK3	ENSG00000251322	22:50706152-50711614:+	0.11764712	0.037558747	0.041152
898	SHKBP1	ENSG00000160410	19:40578212-40578461:+	0.25000007	0.060606185	0.025288
899	SHKBP1	ENSG00000160410	19:40590422-40590729:+	0.40000009	0.11111126	0.027472
900	SHMT1	ENSG00000176974	17:18330671-18333165:-	0.29090919	0.059701625	0.019395
901	SHMT1	ENSG00000176974	17:18333288-18335558:-	0.18867937	0.017060385	0.013798
902	SIN3B	ENSG00000127511	19:16871398-16876054:+	0.19767448	0.074561476	0.034585
903	SKIV2L	ENSG00000204351	6:31969420-31969514:+	0.4497817	0.220930308	0.025474
904	SLC12A9	ENSG00000146828	7:100861262-100861391:+	0.42281888	0.148148277	0.032859
905	SLC20A1	ENSG00000144136	2:112652798-112657121:+	0.22222231	0.070270373	0.041637
906	SLC22A23	ENSG00000137266	6:3298218-3323833:-	0.19540238	0.053191588	0.034355
907	SLC25A27	ENSG00000153291	6:46653298-46655842:+	0.72789118	0.290909213	0.010841
908	SLC25A27	ENSG00000153291	6:46671228-46676382:+	0.46391758	0.168421206	0.03626
909	SLC25A27	ENSG00000153291	6:46656034-46658961:+	0.48704666	0.094339675	2.38E-06
910	SLC25A32	ENSG00000164933	8:103403324-103404775:-	0.22093032	0.068702386	0.038326
911	SLC25A38	ENSG00000144659	3:39392021-39394409:+	0.04232812	0.312883759	0.002655
912	SLC25A42	ENSG00000181035	19:19104938-19105560:+	0.10909106	0.009197061	0.017487
913	SLC25A46	ENSG00000164209	5:110739402-110742046:+	0.33962282	0.045454737	0.022482
914	SLC26A11	ENSG00000181045	17:80221794-80222654:+	0.12121234	0.009681124	0.026063
915	SLC26A11	ENSG00000181045	17:80223337-80225836:+	0.27848109	0.078947468	0.020564
916	SLC26A11	ENSG00000181045	17:80248674-80249153:+	0.24657544	0.075471814	0.04774
917	SLC26A11	ENSG00000181045	17:80249287-80251328:+	0.31034488	0.090277857	0.006572
918	SLC27A1	ENSG00000130304	19:17501419-17504454:+	0.20858901	0.072463839	0.023194
919	SLC27A4	ENSG00000167114	9:128355796-128360333:+	0.13017756	0.043478313	0.025505
920	SLC30A3	ENSG00000115194	2:27258058-27258160:-	0.19230795	0.014084654	0.03568
921	SLC4A2	ENSG00000164889	7:151071605-151071688:+	0.1120001	0.015576877	0.042764
922	SLC6A1	ENSG00000157103	3:10992929-11015671:+	0.16666675	0.02898559	0.011407
923	SLC6A1	ENSG00000157103	3:11029352-11031176:+	0.11212126	0.042357331	0.046772
924	SLC6A8	ENSG00000130821	X:153688836-153690374:+	0.16062182	0.049792591	0.013773
925	SLC7A6	ENSG00000103064	16:68296513-68296626:+	0.21739151	0.012541451	0.00813
926	SLMAP	ENSG00000163681	3:57923023-57927295:+	0.27272744	0.050000176	0.048737
927	SMARCA1	ENSG00000102038	X:129447233-129448332:-	0.24018479	0.116232518	0.022389
928	SMARCD2	ENSG00000108604	17:63832991-63833068:-	0.32828287	0.155555634	0.045779
929	SMARCD3	ENSG00000082014	7:151240247-151240424:-	0.14136129	0.066384231	0.045745
930	SMARCE1	ENSG00000073584	17:40631693-40632194:-	0.12865501	0.056140396	0.039222

S/N	Gene Affected	ENSEMBL_ID	Position of retained intron	IR ratio AD	IR ratio Control	*p*- value
931	SMG9	ENSG00000105771	19:43734495-43737596:-	0.50862072	0.151898792	9.20E-05
932	SMG9	ENSG00000105771	19:43737682-43738121:-	0.16455701	0.048888942	0.008166
933	SMURF1	ENSG00000198742	7:99049709-99051356:-	0.30000014	0.027559067	0.014778
934	SMURF1	ENSG00000198742	7:99052446-99054789:-	0.16279084	0.019541893	0.034872
935	SMURF1	ENSG00000198742	7:99047882-99049562:-	0.26666676	0.05952393	0.020979
936	SMURF1	ENSG00000198742	7:99057270-99057417:-	0.12765972	0.01535435	0.036861
937	SMURF1	ENSG00000198742	7:99060707-99061798:-	0.12658246	0.012833112	0.034131
938	SON	ENSG00000159140	21:33569087-33573307:+	0.17211057	0.091787469	0.015893
939	SOX10	ENSG00000100146	22:37978135-37983356:-	0.11940303	0.042328101	0.03828
940	SPAG7	ENSG00000091640	17:4959643-4959759:-	0.13993178	0.061224535	0.033053
941	SPATA20	ENSG00000006282	17:50551679-50551968:+	0.45355195	0.164383641	0.00416
942	SPG7	ENSG00000197912	16:89530808-89531903:+	0.11827962	0.040816383	0.040416
943	SPG7	ENSG00000197912	16:89548113-89550493:+	0.13398696	0.053846195	0.020158
944	SPHK2	ENSG00000063176	19:48626362-48627691:+	0.27173921	0.058823636	0.011475
945	SPPL2B	ENSG00000005206	19:2344424-2344552:+	0.11409404	0.007902983	0.005408
946	SPTBN4	ENSG00000160460	19:40554646-40556083:+	0.51798569	0.154929806	0.041529
947	SPTBN4	ENSG00000160460	19:40566359-40567662:+	0.25225239	0.056680329	0.044158
948	SRCIN1	ENSG00000277363	17:38543969-38548556:-	0.58928581	0.081081273	0.006424
949	SREBF2	ENSG00000198911	22:41903155-41904862:+	0.12349401	0.024714856	0.000121
950	SREBF2	ENSG00000198911	22:41904974-41905439:+	0.11894277	0.022099482	0.000711
951	SREBF2	ENSG00000198911	22:41900498-41902969:+	0.10840712	0.014377023	4.87E-05
952	SRRT	ENSG00000087087	7:100882241-100884069:+	0.41052635	0.211678909	0.047368
953	SRSF3	ENSG00000112081	6:36598983-36601151:+	0.24757283	0.11282847	0.001341
954	SSB	ENSG00000138385	2:169806892-169806970:+	0.10682113	0.039196003	0.001488
955	SSBP2	ENSG00000145687	5:81442723-81446867:-	0.19871801	0.036649279	0.002286
956	SSBP2	ENSG00000145687	5:81446922-81448789:-	0.12751684	0.028571488	0.012294
957	STAT5B	ENSG00000173757	17:42201864-42202339:-	0.18674704	0.045161353	0.005367
958	STAT5B	ENSG00000173757	17:42217464-42218150:-	0.1823205	0.065476259	0.030008
959	STIM2	ENSG00000109689	4:27007700-27008427:+	0.11842115	0.006416555	0.002701
960	STK11	ENSG00000118046	19:1226663-1227592:+	0.10526321	0.027173973	0.026048
961	STK19	ENSG00000204344	6:31979553-31980450:+	0.52000004	0.227272835	0.03006
962	STRN4	ENSG00000090372	19:46720771-46721985:-	0.11960137	0.044673583	0.022609
963	STRN4	ENSG00000090372	19:46722072-46722241:-	0.10593224	0.033834628	0.015449
964	SUMF2	ENSG00000129103	7:56078186-56078363:+	0.18333343	0.039370168	0.015104
965	SUN2	ENSG00000100242	22:38740432-38741006:-	0.23154124	0.136439301	0.025583
966	SUPT5H	ENSG00000196235	19:39457740-39458817:+	0.17708343	0.018181886	0.002249
967	SYMPK	ENSG00000125755	19:45823875-45825170:-	0.20512825	0.048507511	0.001009
968	SYNGR1	ENSG00000100321	22:39374553-39376051:+	0.17283957	0.06329122	0.044715
969	SYNGR1	ENSG00000100321	22:39376197-39381695:+	0.15000007	0.030476247	0.004443
970	SYNJ1	ENSG00000159082	21:32673531-32676331:-	0.10416684	0.010989098	0.035013
971	SYNRG	ENSG00000275066	17:37538420-37539191:-	0.1929826	0.034482896	0.038028
972	SYTL4	ENSG00000102362	X:100686781-100687066:-	0.19000025	0.009681134	0.008442
973	TAB3	ENSG00000157625	X:30831575-30834050:-	0.19354859	0.008900388	0.004603
974	TACC1	ENSG00000147526	8:38838546-38840223:+	0.13145545	0.048048103	0.029436
975	TACC1	ENSG00000147526	8:38827288-38831124:+	0.30769238	0.075000097	0.008232
976	TAF6	ENSG00000106290	7:100110073-100110199:-	0.16956526	0.060000043	0.007548
977	TANGO2	ENSG00000183597	22:20056013-20061529:+	0.22058833	0.061947002	0.034821
978	TAPBP	ENSG00000231925	6:33304638-33304988:-	0.16071434	0.03535359	0.003753
979	TBC1D13	ENSG00000107021	9:128792574-128797054:+	0.30841128	0.093525273	0.014178

S/N	Gene Affected	ENSEMBL_ID	Position of retained intron	IR ratio AD	IR ratio Control	*p*- value
980	TBC1D17	ENSG00000104946	19:49878572-49880278:+	0.21052634	0.072115421	0.000829
981	TBC1D25	ENSG00000068354	X:48559024-48559157:+	0.18918937	0.007357656	0.002363
982	TBCD	ENSG00000141556	17:82929500-82930521:+	0.40410964	0.149122909	0.015698
983	TBKBP1	ENSG00000198933	17:47709452-47710497:+	0.21794892	0.027272877	0.029079
984	TBPL1	ENSG00000028839	6:133984671-133986960:+	0.1164384	0.046875051	0.045583
985	TCERG1	ENSG00000113649	5:146482727-146483539:+	0.32298142	0.101382575	0.008696
986	TCF25	ENSG00000141002	16:89893858-89895037:+	0.10569109	0.025316487	0.00124
987	TECPR1	ENSG00000205356	7:98217811-98217935:-	0.11864423	0.008667798	0.016191
988	TECPR1	ENSG00000205356	7:98229166-98230960:-	0.14754112	0.02083345	0.035959
989	TECPR1	ENSG00000205356	7:98231118-98231223:-	0.16666685	0.006648486	0.002822
990	TESK1	ENSG00000107140	9:35606285-35606836:+	0.1233767	0.033783865	0.04682
991	TESK1	ENSG00000107140	9:35607409-35607581:+	0.26470592	0.060085886	0.000207
992	TFEB	ENSG00000112561	6:41687169-41687752:-	0.21120697	0.06422028	0.032403
993	TFPT	ENSG00000105619	19:54114700-54115246:-	0.11180129	0.032258114	0.019253
994	THUMPD3	ENSG00000134077	3:9377888-9380502:+	0.18867932	0.043103534	0.01604
995	THUMPD3	ENSG00000134077	3:9380618-9383198:+	0.46231162	0.139394045	0.005999
996	THUMPD3	ENSG00000134077	3:9383309-9384211:+	0.24175832	0.07534256	0.025741
997	THUMPD3	ENSG00000134077	3:9384335-9384523:+	0.18468475	0.068965595	0.042533
998	TIAM2	ENSG00000146426	6:155249969-155250912:+	0.11224499	0.029520369	0.049677
999	TIMM21	ENSG00000075336	18:74155207-74155305:+	0.20647777	0.074349492	0.007003
1000	TIMM44	ENSG00000104980	19:7933570-7933863:-	0.22727278	0.085106454	0.027701
1001	TLE4	ENSG00000106829	9:79706899-79708117:+	0.16733073	0.052631648	0.021349
1002	TM9SF4	ENSG00000101337	20:32150875-32155102:+	0.14179112	0.033898378	0.020926
1003	TM9SF4	ENSG00000101337	20:32155186-32157793:+	0.22302164	0.074324401	0.022453
1004	TM9SF4	ENSG00000101337	20:32158514-32159991:+	0.1283423	0.034042606	0.01188
1005	TMEM120A	ENSG00000189077	7:75987428-75987537:-	0.19127521	0.059360785	0.006007
1006	TMEM120A	ENSG00000189077	7:75987602-75987717:-	0.2753037	0.109289696	0.025734
1007	TMEM120A	ENSG00000189077	7:75989224-75992143:-	0.11000006	0.026455088	0.021167
1008	TMEM165	ENSG00000134851	4:55396396-55411613:+	0.19125689	0.070175523	0.048522
1009	TMEM181	ENSG00000146433	6:158631389-158631809:+	0.23728823	0.048611207	0.009771
1010	TMEM184B	ENSG00000198792	22:38221710-38222641:-	0.44692744	0.079602079	0.000349
1011	TMEM184B	ENSG00000198792	22:38222662-38224784:-	0.42948726	0.150000153	0.037842
1012	TMEM214	ENSG00000119777	2:27035728-27035969:+	0.29411773	0.104166781	0.049469
1013	TMEM63B	ENSG00000137216	6:44148945-44149858:+	0.26829275	0.063492149	0.00487
1014	TMEM63B	ENSG00000137216	6:44150629-44151845:+	0.16176478	0.039130501	0.011562
1015	TMEM94	ENSG00000177728	17:75491852-75492473:+	0.75268826	0.22580665	0.025529
1016	TMEM94	ENSG00000177728	17:75493102-75493490:+	0.52777783	0.184210619	0.004955
1017	TNK2	ENSG00000061938	3:195879175-195882050:-	0.10502286	0.027196681	0.001017
1018	TNPO3	ENSG00000064419	7:128955385-128957223:-	0.25694449	0.102040887	0.025419
1019	TOM1L2	ENSG00000175662	17:17848859-17861475:-	0.37681166	0.065292196	0.00119
1020	TOMM40	ENSG00000130204	19:44901310-44903029:+	0.17410718	0.041237152	0.000522
1021	TOPBP1	ENSG00000163781	3:133617326-133618212:-	0.36290331	0.126126251	0.036692
1022	TPD52L1	ENSG00000111907	6:125248383-125252021:+	0.39726036	0.067415879	0.007618
1023	TPD52L1	ENSG00000111907	6:125252036-125253716:+	0.57954549	0.103448401	0.000944
1024	TPGS1	ENSG00000141933	19:507844-518888:+	0.15094355	0.011682673	0.015441
1025	TPRKB	ENSG00000144034	2:73730736-73732162:-	0.19277115	0.067532539	0.021723
1026	TPRN	ENSG00000176058	9:137192691-137204103:-	0.66666674	0.237288328	0.048717
1027	TPRN	ENSG00000176058	9:137192174-137192258:-	0.2004718	0.058558657	0.03283
1028	TRABD	ENSG00000170638	22:50193654-50194339:+	0.06666674	0.225352263	0.034084

S/N	Gene Affected	ENSEMBL_ID	Position of retained intron	IR ratio AD	IR ratio Control	*p*- value
1029	TRABD	ENSG00000170638	22:50195040-50197240:+	0.06040275	0.233333478	0.013804
1030	TRABD	ENSG00000170638	22:50197351-50197448:+	0.07692315	0.317757191	0.006729
1031	TRIM27	ENSG00000204713	6:28909088-28911695:-	0.27500005	0.110091816	0.022916
1032	TRIM28	ENSG00000130726	19:58548776-58548861:+	0.13090132	0.033653878	0.000686
1033	TRMT1	ENSG00000104907	19:13109838-13109914:-	0.17692315	0.040000087	0.01816
1034	TRRAP	ENSG00000196367	7:98933402-98935578:+	0.21875032	0.021223994	0.049276
1035	TSC22D3	ENSG00000157514	X:107715950-107716314:-	0.49600008	0.140187044	0.010018
1036	TSGA10	ENSG00000135951	2:99105436-99105526:-	0.12000019	0.007883207	0.015059
1037	TSPAN17	ENSG00000048140	5:176652913-176654894:+	0.25274737	0.068181953	0.044628
1038	TSPOAP1	ENSG00000005379	17:58305171-58305386:-	0.18471347	0.048611212	0.031232
1039	TSPOAP1	ENSG00000005379	17:58306968-58307610:-	0.10476196	0.015151557	0.002024
1040	TSPOAP1	ENSG00000005379	17:58320581-58322307:-	0.36904769	0.123287805	0.036052
1041	TSPYL2	ENSG00000184205	X:53084622-53084754:+	0.11751158	0.021602204	0.001615
1042	TTC19	ENSG00000011295	17:16025171-16026539:+	0.10377364	0.026004769	0.007569
1043	TTYH3	ENSG00000136295	7:2647253-2647417:+	0.12962976	0.010967401	0.014775
1044	TUBG2	ENSG00000037042	17:42660707-42662972:+	0.47524756	0.136783821	0.000681
1045	TUBG2	ENSG00000037042	17:42663052-42663376:+	0.15478618	0.027544937	8.32E-06
1046	TUBG2	ENSG00000037042	17:42665562-42665677:+	0.23728816	0.086118295	0.001187
1047	TUBG2	ENSG00000037042	17:42659552-42659833:+	0.10833339	0.02179841	0.00475
1048	U2AF2	ENSG00000063244	19:55655153-55659209:+	0.14859443	0.037735905	0.007451
1049	U2AF2	ENSG00000063244	19:55660221-55660515:+	0.11405839	0.032934171	0.005539
1050	UBA1	ENSG00000130985	X:47194024-47198802:+	0.26640931	0.071428633	0.001389
1051	UBA3	ENSG00000144744	3:69056693-69056778:-	0.34879408	0.148148184	0.000445
1052	UBA5	ENSG00000081307	3:132660698-132665822:+	0.43283588	0.131386981	0.015023
1053	UBA5	ENSG00000081307	3:132665868-132665983:+	0.34108536	0.106741702	0.028108
1054	UBAP2	ENSG00000137073	9:33923033-33923185:-	0.29268302	0.081081212	0.035564
1055	UBAP2	ENSG00000137073	9:33923293-33923378:-	0.16000011	0.026666795	0.040356
1056	UBAP2	ENSG00000137073	9:33923478-33923794:-	0.10344842	0.008900383	0.019004
1057	UBE2O	ENSG00000175931	17:76391613-76391755:-	0.15000012	0.029239865	0.032432
1058	UBE2O	ENSG00000175931	17:76391813-76391909:-	0.22330108	0.023952182	0.002764
1059	UBE2Z	ENSG00000159202	17:48908820-48910807:+	0.31707324	0.094890602	0.01313
1060	UBE2Z	ENSG00000159202	17:48910880-48912833:+	0.18627456	0.077868911	0.034178
1061	UBR5	ENSG00000104517	8:102257717-102258984:-	0.14542193	0.078378413	0.046586
1062	UFD1L	ENSG00000070010	22:19450744-19454748:-	0.315493	0.151515227	0.038256
1063	UFSP2	ENSG00000109775	4:185400478-185403493:-	0.11602213	0.047318648	0.018105
1064	UFSP2	ENSG00000109775	4:185418507-185418586:-	0.10734476	0.020304662	0.047432
1065	UNC13A	ENSG00000130477	19:17627597-17627862:-	0.12500015	0.013043561	0.017123
1066	UNC13B	ENSG00000198722	9:35386293-35389845:+	0.11428583	0.006342803	0.003942
1067	UPF1	ENSG00000005007	19:18852296-18852986:+	0.10204088	0.019607913	0.023798
1068	UPF3B	ENSG00000125351	X:119841258-119841734:-	0.11464973	0.025906791	0.011981
1069	UQCC1	ENSG00000101019	20:35304069-35306665:-	0.1790124	0.054263627	0.012428
1070	URM1	ENSG00000167118	9:128389309-128389665:+	0.11217953	0.037940424	0.01857
1071	USP16	ENSG00000156256	21:29042528-29043422:+	0.14788736	0.063559374	0.041679
1072	USP19	ENSG00000172046	3:49117570-49117656:-	0.16853948	0.025316602	0.047487
1073	USP20	ENSG00000136878	9:129874756-129874828:+	0.14000014	0.014285804	0.017093
1074	USP34	ENSG00000115464	2:61188709-61188909:-	0.27655103	0.110056969	0.001578
1075	USP34	ENSG00000115464	2:61189069-61190270:-	0.36645965	0.176470628	0.001955
1076	USP34	ENSG00000115464	2:61190414-61190517:-	0.48522406	0.19087143	0.000991
1077	UTP14A	ENSG00000156697	X:129906236-129907366:+	0.42857158	0.017244471	0.001172

S/N	Gene Affected	ENSEMBL_ID	Position of retained intron	IR ratio AD	IR ratio Control	*p*- value
1078	UTP14A	ENSG00000156697	X:129908130-129908669:+	0.45070433	0.081632821	0.011428
1079	UTP4	ENSG00000141076	16:69137885-69139824:+	0.14285725	0.034482851	0.049213
1080	VAMP2	ENSG00000220205	17:8160871-8161472:-	0.10043672	0.013854548	2.59E-06
1081	VARS	ENSG00000204394	6:31782443-31782529:-	0.10769243	0.012949457	0.036228
1082	VARS2	ENSG00000137411	6:30921305-30921588:+	0.11931827	0.010748053	0.012887
1083	VCAN	ENSG00000038427	5:83580162-83580306:+	0.00742117	0.113095322	2.97E-05
1084	VDAC3	ENSG00000078668	8:42404924-42405370:+	0.13399697	0.063774458	0.042959
1085	VEZF1	ENSG00000136451	17:57974900-57979151:-	0.18577081	0.05494515	0.034171
1086	VILL	ENSG00000136059	3:38006252-38006448:+	0.43589758	0.096154072	0.048794
1087	VPS28	ENSG00000160948	8:144423922-144424040:-	0.1498423	0.050215236	0.000553
1088	VPS28	ENSG00000160948	8:144424268-144424717:-	0.11464091	0.033029634	0.000125
1089	VPS9D1	ENSG00000075399	16:89711010-89711326:-	0.23846163	0.069230876	0.03166
1090	VPS9D1	ENSG00000075399	16:89707954-89708426:-	0.41772157	0.137254983	0.004263
1091	VWA5B2	ENSG00000145198	3:184241107-184241186:+	0.3678162	0.099290956	0.040258
1092	WASF1	ENSG00000112290	6:110102216-110103377:-	0.14659692	0.046647307	0.025548
1093	WBP1	ENSG00000239779	2:74459552-74459642:+	0.78527613	0.351648499	0.043941
1094	WBP2	ENSG00000132471	17:75847609-75848569:-	0.61818183	0.119230887	0.000414
1095	WBSCR22	ENSG00000071462	7:73694050-73697604:+	0.21612906	0.071246858	0.000944
1096	WDR13	ENSG00000101940	X:48598037-48598716:+	0.24381629	0.119617284	0.041481
1097	WDR13	ENSG00000101940	X:48600626-48601783:+	0.13076926	0.049701828	0.010506
1098	WDR18	ENSG00000065268	19:990364-990851:+	0.52439029	0.148437604	0.003767
1099	WDR36	ENSG00000134987	5:111105360-111106056:+	0.15151538	0.014521674	0.035012
1100	WDR54	ENSG00000005448	2:74423982-74424874:+	0.28282834	0.114832631	0.049393
1101	WDR54	ENSG00000005448	2:74424975-74425074:+	0.28378386	0.101083156	0.045785
1102	WDR54	ENSG00000005448	2:74425237-74425416:+	0.20863319	0.044217788	0.0154
1103	WDR54	ENSG00000005448	2:74425491-74425569:+	0.22222228	0.054621912	0.004427
1104	WDR59	ENSG00000103091	16:74909657-74909821:-	0.79459462	0.428571522	0.033658
1105	WDR59	ENSG00000103091	16:74909917-74912197:-	0.73631845	0.330188773	0.01191
1106	WDR6	ENSG00000178252	3:49014116-49014209:+	0.66331662	0.366515924	0.035219
1107	WDR83	ENSG00000123154	19:12670285-12670562:+	0.26530621	0.080808197	0.038713
1108	WDR91	ENSG00000105875	7:135188545-135189343:-	0.26315801	0.043956167	0.013234
1109	WDSUB1	ENSG00000196151	2:159236190-159248371:-	0.27225136	0.111940381	0.037452
1110	WIZ	ENSG00000011451	19:15425768-15426981:-	0.13513522	0.019230868	0.021865
1111	WRNIP1	ENSG00000124535	6:2784403-2785006:+	0.14652959	0.075630286	0.037113
1112	XAB2	ENSG00000076924	19:7620446-7620546:-	0.13084117	0.045833384	0.023828
1113	XAB2	ENSG00000076924	19:7627440-7627727:-	0.10891097	0.004798468	0.001027
1114	XPO7	ENSG00000130227	8:22004030-22004994:+	0.13454549	0.05519485	0.028962
1115	XRCC1	ENSG00000073050	19:43552908-43552981:-	0.1976745	0.059322123	0.035908
1116	YAE1D1	ENSG00000241127	7:39570627-39572276:+	0.23770497	0.092307771	0.038347
1117	YEATS2	ENSG00000163872	3:183803335-183803986:+	0.25657902	0.096774294	0.048698
1118	YEATS2	ENSG00000163872	3:183804188-183806865:+	0.12592599	0.037500072	0.044363
1119	YIF1B	ENSG00000167645	19:38307521-38307596:-	0.25000004	0.072463821	0.00135
1120	YJEFN3	ENSG00000250067	19:19528991-19532631:+	0.36538469	0.088670075	0.010037
1121	YWHAE	ENSG00000108953	17:1345499-1347961:-	0.4000001	0.090909252	0.023932
1122	ZER1	ENSG00000160445	9:128735431-128739930:-	0.14005607	0.039316288	0.005822
1123	ZFAND1	ENSG00000104231	8:81714039-81714803:-	0.1136364	0.048710639	0.033773
1124	ZFAND1	ENSG00000104231	8:81714895-81714986:-	0.11052637	0.017793639	0.002559
1125	ZMIZ2	ENSG00000122515	7:44759460-44760424:+	0.24117653	0.103603684	0.049495
1126	ZMIZ2	ENSG00000122515	7:44761593-44761694:+	0.26294827	0.09138391	0.024364

S/N	Gene Affected	ENSEMBL_ID	Position of retained intron	IR ratio AD	IR ratio Control	*p*- value
1127	ZMYM3	ENSG00000147130	X:71242424-71242969:-	0.1733334	0.044444523	0.018357
1128	ZNF207	ENSG00000010244	17:32360967-32361467:+	0.22981376	0.066326636	0.029407
1129	ZNF346	ENSG00000113761	5:177041870-177044388:+	0.12280716	0.014524391	0.037834
1130	ZNF444	ENSG00000167685	19:56158602-56159623:+	0.1307693	0.019230864	0.021054
1131	ZNF526	ENSG00000167625	19:42224305-42224386:+	0.22222253	0.016721942	0.029328
1132	ZNF607	ENSG00000198182	19:37699895-37707913:-	0.17857167	0.015766388	0.029943
1133	ZNF775	ENSG00000196456	7:150379392-150388421:+	0.16326548	0.01902838	0.037359
1134	ZNRF1	ENSG00000186187	16:75104889-75106481:+	0.12820518	0.041095939	0.019195
1135	ZSCAN18	ENSG00000121413	19:58087008-58087315:-	0.12923082	0.021818217	0.000292
1136	ZSCAN18	ENSG00000121413	19:58086266-58086905:-	0.26760568	0.067889963	0.000397
