# Supplementary material for: Increased intron retention is a post‐transcriptional signature associated with progressive aging and Alzheimer’s disease
Source: Aging Cell. 2019 Mar 13;18(3):e12928. doi: 10.1111/acel.12928 (PMC6516162; doi:10.1111/acel.12928)
Supplement: Supplementary file 10 [file ACEL-18-e12928-s010.pdf]

Table S9: DAVID functional annotation chart of differential IR genes between Control & AD frontal cortex (Emory: SRS373257)

GOTERM_BP_DIRECT	Count	%	*P* -Value	Fold Enrichment	Benjamini value
GO:0006541~glutamine metabolic process	3	3.7974684	0.0033668	34.03783784	0.811000718
GO:0045333~cellular respiration	3	3.7974684	0.0037108	32.41698842	0.600790221
GO:0015991~ATP hydrolysis coupled proton transport	3	3.7974684	0.0084985	21.27364865	0.754731139
GO:0009070~serine family amino acid biosynthetic process	2	2.5316456	0.008676	226.9189189	0.659097264
GO:0006406~mRNA export from nucleus	4	5.0632911	0.0094302	9.076756757	0.607853808
GO:0006405~RNA export from nucleus	3	3.7974684	0.0238645	12.37739558	0.863121939
GO:0006537~glutamate biosynthetic process	2	2.5316456	0.0258058	75.63963964	0.841984583
GO:0006544~glycine metabolic process	2	2.5316456	0.0384613	50.42642643	0.911243768
GO:0007030~Golgi organization	3	3.7974684	0.0411825	9.199415632	0.900572554
GO:0051301~cell division	5	6.3291139	0.0659604	3.241698842	0.965640617
GO:0006281~DNA repair	4	5.0632911	0.0826131	3.862449684	0.979190132
GO:0015031~protein transport	5	6.3291139	0.0930191	2.872391379	0.982033742

Table S9: Differential IR between Control & AD frontal cortex (Emory: SRS373257)

S/N	Gene ID	Ensembl ID	Chr Position	p-value	IR ratio AD	IR ratio Control
1	COG5	ENSG00000164597	7:107548180-107548277:-	0.045150552	0.185185451	0.021640105
2	USO1	ENSG00000138768	4:75804272-75805139:+	0.035373017	0.166666915	0.018394084
3	DIMT1	ENSG00000086189	5:62394047-62394483:-	0.04254845	0.333333474	0.065217579
4	POLR3C	ENSG00000186141	1:145837596-145838055:+	0.019148383	0.178571647	0.016721895
5	SMNDC1	ENSG00000119953	10:110295381-110297566:-	0.040455855	0.119047781	0.016721885
6	QRSL1	ENSG00000130348	6:106654922-106655614:+	0.031938954	0.324324445	0.062500172
7	IKZF5	ENSG00000095574	10:122994723-122995993:-	0.012939394	0.185185398	0.015328404
8	CNOT8	ENSG00000155508	5:154870822-154871729:+	0.047646266	0.185185404	0.024756493
9	ARHGEF12	ENSG00000196914	11:120477305-120477446:+	0.045123617	0.156862889	0.024756479
10	THOC1	ENSG00000079134	18:223505-224083:-	0.025191574	0.184210672	0.024261344
11	ADD1	ENSG00000087274	4:2894093-2894581:+	0.034580622	0.179487347	0.024261354
12	TSGA10	ENSG00000135951	2:99105436-99105526:-	0.019409713	0.353846282	0.05769248
13	COG4	ENSG00000103051	16:70481487-70481763:-	0.037524471	0.125000219	0.014149301
14	GTF2H3	ENSG00000111358	12:123652561-123652706:+	0.032563163	0.116279242	0.014149295
15	MMS19	ENSG00000155229	10:97460232-97460694:-	0.029641081	0.361111216	0.084745911
16	USF1	ENSG00000158773	1:161040670-161040813:-	0.031151403	0.148148411	0.013625265
17	GORASP2	ENSG00000115806	2:170950290-170951327:+	0.04051167	0.11538485	0.013625246
18	ZNF585A	ENSG00000196967	19:37165696-37169838:-	0.049263602	0.111111352	0.01362525
19	IPO5	ENSG00000065150	13:98015641-98015725:+	0.033113797	0.153846317	0.020560471
20	TIA1	ENSG00000116001	2:70212845-70214348:-	0.045395139	0.227272845	0.049180469
21	TPRKB	ENSG00000144034	2:73732285-73732583:-	0.008268492	0.210526451	0.019886352
22	DYNC1LI1	ENSG00000144635	3:32530328-32530460:-	0.041760191	0.156250206	0.019565624
23	RRM1	ENSG00000167325	11:4121765-4122140:+	0.044909335	0.147059019	0.01956562
24	POLR3C	ENSG00000186141	1:145833582-145836493:+	0.035778396	0.114285933	0.012061706
25	GTPBP8	ENSG00000163607	3:112995265-112996891:+	0.028210179	0.125000253	0.011867157
26	MSH2	ENSG00000095002	2:47475270-47476366:+	0.031630704	0.125000257	0.011867161
27	MTHFD1	ENSG00000100714	14:64431639-64431786:+	0.017491145	0.160000258	0.011678801
28	PHGDH	ENSG00000092621	1:119726905-119727003:+	0.027576971	0.116279267	0.011678795
29	NELFCD	ENSG00000101158	20:58992020-58992997:+	0.027634745	0.150943545	0.01895419
30	GART	ENSG00000159131	21:33534753-33535224:-	0.023819345	0.125000245	0.011496306
31	HMGCR	ENSG00000113161	5:75359310-75359397:+	0.04113849	0.275862244	0.04411782
32	ANKRD37	ENSG00000186352	4:185396950-185397149:+	0.030780113	0.020560474	0.166666934
33	CDC40	ENSG00000168438	6:110219479-110219735:+	0.041651598	0.250000163	0.043478412
34	ATG10	ENSG00000152348	5:82253429-82254067:+	0.04147013	0.187500158	0.028169149
35	NXF1	ENSG00000162231	11:62800486-62801093:-	0.039106816	0.125000163	0.017085467
36	OGFOD1	ENSG00000087263	16:56462634-56466151:+	0.035446526	0.181818311	0.027777908
37	GLS2	ENSG00000135423	12:56472751-56473227:-	0.042926065	0.250000176	0.040540704
38	ATG13	ENSG00000175224	11:46657622-46659391:+	0.048274714	0.111111262	0.016848167
39	PDLIM5	ENSG00000163110	4:94585737-94586407:+	0.017879351	0.105263331	0.010078952
40	SKAP2	ENSG00000005020	7:26669656-26670090:-	0.036449147	0.1282053	0.016392817
41	IFT172	ENSG00000138002	2:27445105-27445295:-	0.04536338	0.10909105	0.016174244
42	EIF5A	ENSG00000132507	17:7311481-7311577:+	0.008120218	0.127272882	0.009681104
43	LGMN	ENSG00000100600	14:92712871-92713822:-	0.008893186	0.166666913	0.009555385
44	TRNAU1AP	ENSG00000180098	1:28571900-28577499:+	0.011359809	0.111111267	0.009555371

S/N	Gene ID	Ensembl ID	Chr Position	p-value	IR ratio AD	IR ratio Control
45	SLC4A1AP	ENSG00000163798	2:27675692-27677294:+	0.049499883	0.106383135	0.01555216
46	COQ10B	ENSG00000115520	2:197470171-197473756:+	0.031504241	0.137931241	0.015163361
47	OGFOD1	ENSG00000087263	16:56474950-56475506:+	0.008046599	0.49230774	0.150537736
48	NMD3	ENSG00000169251	3:161247330-161249453:+	0.018865735	0.10714308	0.009083504
49	RAD1	ENSG00000113456	5:34909356-34911553:-	0.033341399	0.106383113	0.01479351
50	PTK2	ENSG00000169398	8:140668424-140669726:-	0.035461563	0.232142989	0.046511763
51	ATP6V0A1	ENSG00000033627	17:42495716-42498923:+	0.026772649	0.230769381	0.034883855
52	MLF2	ENSG00000089693	12:6751676-6751924:-	0.022822578	0.135135309	0.014271397
53	MFSD14A	ENSG00000156875	1:100077277-100078450:+	0.006849772	0.135135322	0.008656049
54	NDUFS1	ENSG00000023228	2:206147834-206149019:-	0.04137445	0.108108284	0.014105449
55	SBNO1	ENSG00000139697	12:123328895-123330418:-	0.003838856	0.125000147	0.008360948
56	TRAPPC13	ENSG00000113597	5:65664384-65664503:+	0.044381269	0.100000167	0.013629983
57	G3BP2	ENSG00000138757	4:75655870-75656923:-	0.039650638	0.25000014	0.052631709
58	OGFOD1	ENSG00000087263	16:56467293-56467904:+	0.037384797	0.100000157	0.01333042
59	FASTKD2	ENSG00000118246	2:206788155-206788818:+	0.036666887	0.214285819	0.052631688
60	RNF41	ENSG00000181852	12:56208298-56210296:-	0.032259502	0.102564263	0.01276914
61	METTL3	ENSG00000165819	14:21499802-21500494:-	0.039672013	0.196969795	0.050505154
62	PRUNE2	ENSG00000106772	9:76629290-76636470:-	0.018038287	0.121212287	0.012636126
63	PPME1	ENSG00000214517	11:74204445-74222311:+	0.034823255	0.125000247	0.0121307
64	ENPP5	ENSG00000112796	6:46165563-46167433:-	0.01484043	0.133333517	0.011892831
65	RP3-382I1(	ENSG00000213204	6:87500667-87501157:+	0.006635185	0.100000137	0.007284792
66	ATP6V0A1	ENSG00000033627	17:42499042-42500706:+	0.012956221	0.103448497	0.007074658
67	TM9SF2	ENSG00000125304	13:99540793-99541558:+	0.033907067	0.214285826	0.050847566
68	PTCD3	ENSG00000132300	2:86125515-86125794:+	0.042874667	0.27272735	0.094488286
69	KLHDC2	ENSG00000165516	14:49779675-49779747:+	0.038938168	0.376344152	0.140845187
70	ATP6V1A	ENSG00000114573	3:113788875-113789731:+	0.035207233	0.218750142	0.04411776
71	PPID	ENSG00000171497	4:158715684-158717011:-	0.012939061	0.20454557	0.034246664
72	ATP1A1	ENSG00000163399	1:116390411-116390781:+	0.010532869	0.105263323	0.008196416
73	ERLEC1	ENSG00000068912	2:53814935-53817897:+	0.019723476	0.181818276	0.042944859
74	GLUD1	ENSG00000148672	10:87060825-87060914:-	0.048838836	0.188034251	0.071428644
75	ACTR6	ENSG00000075089	12:100218586-100220007:+	0.03324121	0.101695021	0.018750068
76	ACTR6	ENSG00000075089	12:100210208-100210294:+	0.014370027	0.108433838	0.011560765
77	NAPB	ENSG00000125814	20:23379495-23379866:-	0.024340469	0.162162324	0.022346464
78	PREPL	ENSG00000138078	2:44339363-44342416:-	0.022380328	0.100000134	0.011111181
79	IFI44L	ENSG00000137959	1:78635489-78637031:+	0.014606249	0.172414074	0.006628842
80	CLASP2	ENSG00000163539	3:33498717-33501651:-	0.03187246	0.132075535	0.04123717
81	FBXO3	ENSG00000110429	11:33754500-33755770:-	0.043878536	0.135802558	0.038647415
82	TRIM2	ENSG00000109654	4:153324148-153328529:+	0.041436874	0.156862807	0.057777836
83	NSL1	ENSG00000117697	1:212738686-212739533:-	0.003088803	0.143939453	0.026087008
84	ZNF207	ENSG00000010244	17:32368014-32369294:+	0.035341742	0.153333388	0.056603832
85	NDUFS2	ENSG00000158864	1:161213921-161214155:+	0.019058652	0.380880173	0.145077841
