# Supplementary material for: Increased intron retention is a post‐transcriptional signature associated with progressive aging and Alzheimer’s disease
Source: Aging Cell. 2019 Mar 13;18(3):e12928. doi: 10.1111/acel.12928 (PMC6516162; doi:10.1111/acel.12928)
Supplement: Supplementary file 11 [file ACEL-18-e12928-s011.pdf]

Table S10: Differential IR genes whose quantile normalized peptide expression is significantly different between Control & AD frontal cortex

Gene	Accession	AD1	AD2	AD3	AD4	AD5	AD6	AD7	AD8	AD9	AD10	Average
MAPT	P10636	1.738671	1.84477	1.151977	1.342767	1.076752	1.608114	1.779346	1.19947	1.150064	2.176316	1.50682473
AP3D1	O14617-5	0.825239	0.975037	0.957936	1.023596	0.830064	0.827893	0.872182	0.852882	0.808655	0.864334	0.88378183
ADAM11	O75078	0.910923	0.801974	0.879972	0.993109	0.842598	0.970275	0.932228	0.939643	0.910569	1.086574	0.92678672
NPTX2	P47972	0.24523	0.234407	0.649577	0.616106	0.223178	0.751482	0.24748	0.414792	0.820307	0.866915	0.50694742
ABHD16A	O95870	0.827465	0.85258	0.88604	0.926356	0.880032	0.879227	0.858791	0.890894	0.868901	0.778537	0.86488234
RAB3GAP1	Q15042-3	0.825378	0.937161	0.96407	0.981767	0.920502	0.853819	0.882818	0.951773	0.948865	0.805275	0.907143
MAPT	P10636-4	2.160359	2.272352	1.090306	1.526824	1.078492	2.957526	2.126318	1.12891	1.040302	3.33051	1.87118991
ACTN4	K7EJH8	0.686108	0.682997	0.818833	0.850523	1.073386	0.79464	0.903214	1.006444	0.952301	0.983744	0.87521899
PDHA1	P08559-4	0.767548	0.803183	1.025595	1.061145	0.788662	0.835401	0.966521	0.826353	0.840523	1.009419	0.89243499
BRSK1	Q8TDC3-2	0.789722	0.972451	1.001872	1.077078	0.888144	0.883996	0.904077	0.87916	0.952596	0.901019	0.92501164
DOCK3	Q8IZD9	0.778817	0.942315	0.879854	1.003908	0.911814	0.845317	0.926519	0.982149	0.977641	0.8284	0.9076736
PLXNB1	O43157	1.328527	1.334818	0.823432	0.930212	1.291216	1.261905	1.021888	1.212717	1.234168	1.066353	1.15052351
TIMM44	O43615	0.762004	0.778817	1.014972	1.196345	0.899952	0.839929	0.938646	0.944456	0.893709	0.966179	0.92350095
NRBP1	F8W6G1	0.875817	0.879521	0.87605	1.003136	0.807255	0.789675	1.012172	0.909307	0.9098	0.890755	0.89534873
CSNK1E	P49674	1.026787	0.944418	1.03178	1.150287	1.068305	1.106712	1.092003	1.051855	1.040216	0.899315	1.04116779
GALT	P07902	1.247315	1.152061	1.355518	1.274357	1.068224	1.078615	1.201786	1.085539	1.381638	0.915472	1.1760525
ACTN4	H7C144	0.377773	0.795578	0.852174	0.66768	0.965664	0.760151	0.827976	0.65829	0.749693	0.935493	0.75904721
NCKIPSD	Q9NZQ3	0.843233	0.99182	0.977716	1.008804	0.916485	0.935913	0.904795	0.837582	0.914886	0.75162	0.90828527
NDUFA10	E7ESZ7	0.611996	0.733741	0.967979	1.09041	0.713715	0.873482	0.887599	0.749363	0.815149	0.99488	0.84383148
SLC25A27	O95847	0.523803	0.616417	0.932411	1.248211	0.665766	0.547713	1.0276	0.875598	0.706599	0.664828	0.7808946
ATL2	Q8NHH9	0.854321	0.916095	1.093864	0.849055	0.864829	0.72422	0.8051	0.882158	1.066844	0.774257	0.88307437
AUH	Q13825	0.731463	0.880963	1.003296	0.97468	0.887803	0.867755	1.01474	0.78156	0.833407	1.01316	0.89888269
ACTN4	O43707	0.83547	0.759354	1.070997	0.996882	0.965199	0.89022	0.963112	0.838497	1.07534	0.981422	0.93764917
SCRIB	Q14160-3	1.230819	1.204473	1.024068	0.915586	1.074465	0.930327	0.905956	1.170828	1.16091	0.997001	1.06144321
ATL2	Q8NHH9-4	0.854321	0.916095	1.142821	0.860668	0.864829	0.72422	0.8051	0.882158	1.053594	0.777103	0.88809091
DLG1	Q12959-2	1.256927	0.915127	0.930164	0.809488	1.320875	1.650803	1.075746	1.333664	1.247139	1.093187	1.163312
CACNA2D1	P54289-2	0.74529	0.976588	1.130045	1.032191	0.910014	0.832157	0.726329	0.869351	0.848634	0.98757	0.90581696
PPP5C	P53041	0.798244	0.893223	0.942154	1.02578	0.865854	0.887963	1.043021	0.916808	0.917985	0.910045	0.92010762
CHERP	J3QK89	1.10775	1.078236	1.100695	0.979875	1.121651	1.187231	1.261363	1.011968	1.080165	1.185023	1.1113956
SLC30A3	Q99726	0.751772	0.79169	1.040372	1.258814	0.681976	0.663982	0.84866	0.281228	0.808775	0.91835	0.80456198
UNC13A	F8W059	0.718915	1.040375	1.097147	1.140823	0.804099	0.808965	0.947623	0.789206	0.785698	0.773695	0.8906546

Gene	Accession	AD1	AD2	AD3	AD4	AD5	AD6	AD7	AD8	AD9	AD10	Average
SYNGR1	O43759	0.674858	0.946965	1.117185	1.043498	0.624915	0.584412	1.011932	0.275235	0.631515	0.681177	0.75916928
TPD52L1	J3KNE7	1.347693	1.258327	1.164416	0.902899	1.115505	1.002464	1.156564	1.253353	0.985619	1.007419	1.11942601
HMGA1	P17096	0.99426	0.826503	0.902497	0.876814	1.191589	1.10653	0.994302	1.225302	1.192814	1.270803	1.05814138
MAGED2	Q9UNF1	1.052644	1.17814	1.224932	0.874349	0.989526	1.164152	1.063366	1.059428	1.152808	1.016176	1.07755209
HMGA1	P17096-3	0.99426	0.826503	0.892406	0.843476	1.191589	1.10653	0.994302	1.225302	1.192814	1.270803	1.05379851
PMPCB	O75439	0.872137	0.972198	0.824452	1.12262	0.816278	0.942078	1.01143	0.948036	0.842848	0.986367	0.93384444
ALCAM	Q13740	0.954856	0.98295	1.104678	0.892062	1.121741	1.189303	1.0762	1.168538	1.228696	1.156779	1.08758048
PMPCB	G3V0E4	0.872137	0.972198	0.834362	1.103129	0.832228	0.937098	1.016405	0.955898	0.842848	0.986367	0.93526705
RNMT	O43148-2	0.857428	0.685791	0.830122	0.985768	0.865127	0.892245	0.931054	0.772646	0.847102	1.027599	0.86948822
ALCAM	Q13740-2	0.954374	0.981945	1.107368	0.889212	1.121431	1.182945	1.0762	1.168538	1.229861	1.152668	1.08645428
HNRNPA2B1	P22626	1.102078	0.985615	0.9651	0.898966	1.120409	1.049114	0.951911	1.052486	1.080309	1.246428	1.04524171
ITGA3	P26006-1	1.532365	1.313401	0.946099	1.033127	1.20756	1.307857	1.073273	0.982528	0.806987	0.70335	1.09065464
ACO2	A2A274	0.90096	0.9087	1.020844	1.01871	0.850519	0.931098	0.965018	0.84897	0.902589	1.064336	0.94117427
ACO2	Q99798	0.902126	0.908599	1.020844	1.01871	0.850519	0.931098	0.965018	0.84897	0.902589	1.064336	0.94128072
MYH7B	A0A087X0T3	1.284338	1.640422	0.202002	0.924563	1.071707	1.112201	1.34014	0.79425	1.283592	2.193455	1.18466708
GK	P32189	0.763767	0.815718	0.990322	1.191677	0.876787	0.859488	1.100946	0.811597	0.86176	0.902157	0.91742178
EPB41L3	Q9Y2J2-2	0.778919	0.795825	0.931541	0.899091	0.857635	0.910014	0.866977	0.943922	0.964922	1.009381	0.8958229
CLTC	A0A087WVQ	0.880074	0.968764	1.035546	0.984166	0.863484	0.893352	1.041627	0.876122	0.88575	0.858703	0.92875871
EFR3B	Q9Y2G0	0.927626	0.998889	1.13861	0.969614	0.919343	0.83124	0.886781	0.813601	0.8948	0.977233	0.9357738
C9orf142	Q9BUH6	1.222219	1.042874	1.038025	0.974363	1.062938	0.788145	0.997775	1.234054	1.037343	1.000784	1.03985208
GRIPAP1	Q4V328	0.811438	0.98776	0.985045	1.077894	0.882486	0.900905	0.965311	0.926628	0.859639	0.875381	0.92724879
SHMT1	P34896	1.275349	1.399174	0.927293	0.68515	1.273222	1.596278	0.803919	1.085165	1.159734	1.091182	1.1296465
TTC19	Q6DKK2	0.819872	1.013798	0.912854	0.961086	0.907431	1.019172	0.273835	0.222629	0.743981	0.996557	0.78712158
SYNJ1	C9JFZ1	0.705809	0.938056	1.010317	0.989543	0.870549	0.877475	1.01703	0.938587	0.900855	0.88313	0.91313511
CTNNA1	P35221	1.09993	1.033259	1.1081	0.88301	1.25108	1.092777	1.008895	1.349979	1.156418	0.927274	1.09107214
WBP2	Q969T9	0.83609	0.985668	0.850733	0.999542	0.967425	0.953301	0.9029	0.835379	0.905505	1.028623	0.92651666
FSD1	Q9BTV5	0.777392	0.76539	0.986557	1.106591	0.949622	0.891601	0.830027	0.936831	1.126426	1.078903	0.94493408
ANK2	I6L894	0.950612	1.032654	0.995095	0.949266	1.407183	1.320693	0.899243	0.855006	1.560126	1.157362	1.11272388
MRPL39	Q9NYK5	0.669747	0.96108	0.496905	1.198839	0.836315	0.969711	0.953632	0.842455	0.97417	1.233011	0.91358641
SBF1	O95248-4	0.859849	1.019919	0.947127	0.965156	0.911327	0.833602	1.019641	0.9687	0.932749	0.807649	0.92657187
ANKRD28	O15084-1	0.76512	0.831099	1.143824	1.015216	0.965559	0.853444	0.891227	1.015005	0.976911	0.888472	0.93458761
YIF1B	Q5BJH7	0.809235	0.521927	0.202002	0.196187	1.021871	0.911417	0.854895	1.008178	1.017039	0.235499	0.67782512

Gene	Accession	AD1	AD2	AD3	AD4	AD5	AD6	AD7	AD8	AD9	AD10	Average
MAPT	P10636-8	1.755314	1.848022	1.287149	1.663253	0.223178	0.21117	1.799803	1.204369	1.150675	2.251523	1.33944559
TECPR1	Q7Z6L1-4	0.885716	0.98873	0.828853	1.043224	0.900954	0.851416	0.835003	0.77755	0.847427	1.361596	0.93204707
GGA3	Q9NZ52	0.598971	0.589251	0.880227	0.839039	0.966964	0.984558	0.927096	0.840348	0.780612	0.947002	0.83540677
MAST3	O60307	0.962377	1.038137	0.912907	1.006396	0.90527	0.992896	1.012805	1.000813	0.98316	0.747263	0.9562025
HMGCS1	Q01581	0.876318	1.197222	0.640003	0.611586	0.796703	1.106803	0.819351	0.619332	0.84099	0.869034	0.83773416
GOPC	Q9HD26	1.006917	0.923076	1.000035	0.994068	0.897228	0.910191	1.109119	0.870453	0.986337	0.968207	0.96656326
MAPRE3	Q9UPY8	0.901602	1.004847	0.979704	1.089857	0.891705	0.870998	0.933384	0.971996	0.888841	1.006725	0.95396596
GAB1	Q13480-2	1.171013	0.939969	0.833302	0.767473	1.499983	1.528353	0.959247	1.601143	1.455646	1.050914	1.18070436
MORF4L2	Q15014	1.113458	0.787274	0.959235	1.155741	1.049802	0.899535	1.460343	1.38129	1.105936	1.277069	1.11896815
ITGB4	P16144	1.522631	1.530543	1.147583	0.812298	1.243962	1.091215	0.699689	0.926423	1.141422	0.96741	1.1083175
HTRA2	O43464	0.963371	0.880698	0.787544	0.887632	0.899391	0.996917	0.809558	0.896937	0.894988	1.071055	0.90880916
LAMP2	P13473-3	1.300956	0.826919	0.774311	0.757129	1.54166	1.511504	0.868807	1.312572	2.176316	2.268116	1.33382902
PPP1R3F	Q6ZSY5	0.846359	0.818758	0.914986	0.869048	0.989751	1.202736	1.016771	0.908915	0.845231	0.919759	0.9332315
AIFM1	O95831	0.854002	0.571056	0.89485	1.013307	0.873602	0.916485	1.106445	0.810053	0.902103	1.118251	0.9060154
GABBR1	Q9UBS5	0.722222	1.060005	1.048474	1.187516	0.847332	0.825841	0.870119	0.772125	0.815637	0.828552	0.89778236
DLGAP4	Q9Y2H0-1	0.794613	0.92811	1.017197	1.204045	0.926025	0.899281	0.906658	0.905226	0.745347	0.854266	0.91807683
WASF1	Q92558	0.807356	0.954374	1.13834	0.981286	0.88342	0.845413	0.88483	0.914666	0.820786	0.937281	0.91677508
RBBP7	Q16576-2	1.028165	0.971053	1.181395	0.86234	1.077345	1.0144	0.901744	0.909028	1.010484	1.041796	0.99977508
STK11	Q15831	0.741425	0.704161	1.130535	1.06986	1.06253	1.028578	0.705994	1.048511	0.902403	0.942011	0.93360081
MPV17	P39210	0.942998	0.851958	1.047638	0.992995	0.865302	1.032734	0.715372	0.706664	0.92215	0.873972	0.89517834
FLII	Q13045	0.895112	0.903039	1.022627	1.002562	0.926449	0.955061	0.919983	0.918548	1.078459	0.763122	0.93849629
MAP7	Q14244	1.237982	1.083944	1.061145	1.528188	1.196638	1.465834	0.898226	1.379295	1.435333	1.199837	1.24864223
ZNF207	X6R4W8	1.078047	0.871182	1.068095	1.125722	1.080047	1.170403	1.077476	0.968795	1.021992	1.007058	1.04688177
ARFGAP1	Q8N6T3-2	0.81099	0.947491	0.954765	1.117391	0.885138	0.876286	0.859989	0.784154	0.883292	0.85911	0.89786052
MAP4	P27816	1.383963	1.092533	0.929029	0.829747	1.276776	1.381583	0.816762	1.407707	1.320433	1.082804	1.15213374
PCM1	Q15154	0.883834	1.030204	1.012362	1.095361	0.955106	0.890278	0.911718	0.973592	1.042919	0.811596	0.96069688
PCIF1	Q9H4Z3	1.12819	1.164002	0.869852	0.937038	1.3413	1.095353	0.817766	0.830027	1.204036	0.925605	1.03131692
TIMM21	Q9BVV7	0.858474	0.698697	0.993771	0.981158	0.866833	0.921473	0.743947	1.016944	1.02815	0.935188	0.90446351
VCAN	P13611	1.437055	0.988455	0.668126	0.592933	1.958964	1.330957	1.270271	1.82933	1.711452	1.272321	1.30598645
NONO	Q15233	1.001139	1.009891	1.106123	0.94016	1.064537	1.027452	0.962468	0.969746	1.06039	1.246692	1.03885975
SRRT	Q9BXP5	1.03989	1.026697	0.960677	0.883546	1.143964	1.030757	0.925363	1.031943	1.211083	1.070156	1.03240749
RBBP7	Q16576	1.028165	0.971053	1.181395	0.86234	1.08442	1.012554	0.877911	0.900478	1.006725	1.033412	0.99584531

Gene	Accession	AD1	AD2	AD3	AD4	AD5	AD6	AD7	AD8	AD9	AD10	Average
CARM1	Q86X55	0.93735	1.050002	0.926846	0.891804	1.007033	0.975082	0.96712	0.92003	0.986984	0.8549	0.95171517
MRPL2	Q5T653	1.045916	0.861179	0.766175	1.19076	0.979245	0.844144	0.946386	0.828453	0.761575	0.739145	0.89629789
SH3GLB2	Q9NR46-2	0.772758	1.0143	1.036412	0.970211	0.721151	0.7246	1.075327	0.939424	0.79254	0.892401	0.89391223
UFD1L	Q92890-1	0.98639	0.879734	0.933827	1.051471	1.190878	1.104471	1.113511	1.108462	1.096571	1.128714	1.05940293
DPM1	H0Y368	0.973698	1.150115	0.998089	0.912283	0.919963	1.052136	0.779914	0.858945	1.01977	0.918094	0.95830067
PPP6R2	O75170-5	0.895687	1.112569	0.867602	1.060051	0.905829	0.994873	0.942308	0.880237	0.988167	0.834137	0.94814592
SLC27A4	Q6P1M0	0.770807	0.89789	0.863338	0.996579	0.924493	0.953834	1.124042	0.795344	0.945373	0.795811	0.90675099
PTGDS	P41222	1.529483	0.757259	0.739354	0.802088	1.250824	1.124966	1.396378	1.364809	1.306907	1.590083	1.186215
RAD50	Q92878-2	1.140837	0.974142	1.049025	0.897491	1.083464	1.019461	0.992427	1.079515	1.102836	0.958221	1.02974174
MAP4	P27816-7	1.483076	1.884414	1.41347	0.861678	1.455724	1.860006	0.838418	1.353877	1.287272	0.959301	1.33972367
UPF1	Q92900	0.999547	1.035325	0.97748	1.01969	1.038075	0.926	1.002022	1.012881	0.981004	0.998222	0.99902452
PRAF2	O60831	0.865977	0.919995	1.074473	0.92012	1.028935	0.878514	0.96737	1.107394	1.044867	0.682716	0.94903608
LONP1	P36776	0.935971	0.879633	0.977831	1.077391	0.885491	0.964081	0.985524	0.877201	0.931268	0.920048	0.94344398
CACNA1A	A0A087WW6	0.812978	0.941526	1.146666	1.200457	0.761821	0.808209	0.844282	0.765814	0.800784	0.880751	0.89632871
MPRIP	H0Y2S9	0.946139	1.104833	0.959381	1.03865	0.880614	0.865092	0.967321	0.858139	0.901134	0.962055	0.94833589
DLG1	Q12959-9	0.925051	0.913679	1.013952	1.038881	0.888087	0.866075	0.888886	0.967159	0.948809	1.08863	0.95392105
ACTR1B	P42025	0.970035	0.909368	1.055806	0.905843	1.026594	0.829283	0.576551	0.990291	1.113217	0.973688	0.93506759
ZER1	Q7Z7L7	1.030904	0.973922	0.954088	0.899913	0.983545	1.063603	1.219821	0.955939	1.078593	0.939634	1.00999618
SPTBN4	Q9H254	0.912899	0.907054	0.889002	0.992252	1.053692	1.120854	0.979025	1.062246	1.088922	0.910108	0.99160538
SRRT	Q9BXP5-3	1.033974	1.034846	0.960677	0.883546	1.087105	0.974904	0.925363	1.031943	1.211083	1.070156	1.02135969
SPTBN4	C9JY79	0.911169	0.905733	0.889002	0.992252	1.053692	1.120854	0.979131	1.062919	1.088031	0.909424	0.99122061
PPP6R2	O75170	0.895687	1.112569	0.867602	1.060051	0.908958	1.02405	0.942308	0.880237	0.988167	0.834137	0.95137647
TNPO3	Q9Y5L0	0.974647	1.066145	1.000321	1.023011	0.976094	0.987087	1.028507	1.029555	0.959004	0.704618	0.9748988
MAPK14	Q16539	1.29099	1.233824	0.956248	1.260744	0.927804	0.49632	1.212899	0.965677	1.047101	0.940645	1.03322534
SAFB2	Q14151	1.070391	1.012644	0.970933	1.028821	1.198896	0.973472	0.979777	1.081573	1.065159	1.015181	1.03968468
APOL2	J3KQL8	0.90026	0.95822	1.086351	0.98643	0.969126	0.855943	0.903547	0.271713	0.91261	1.02538	0.88695808
LARS	Q9P2J5	0.824283	1.025804	0.921476	1.096704	0.866463	0.974682	1.04404	0.904907	0.89645	0.869273	0.9424083
EML2	O95834-3	1.156329	1.047977	0.893058	0.873733	1.103324	1.350133	1.24503	0.851278	1.012071	0.988146	1.0521079
UBA5	Q9GZZ9	0.953807	1.118779	1.051629	0.89117	0.957436	0.939092	1.095478	1.257289	0.955108	1.030699	1.02504865
GIT1	Q9Y2X7-3	0.86948	0.971571	1.038777	1.041372	1.00407	0.949671	0.933705	0.963354	0.923024	0.88828	0.95833053
COG1	Q8WTW3	1.065949	1.172647	0.793539	0.954705	0.950928	1.059402	0.806267	0.927144	0.889317	1.027775	0.9647673
PSMB8	P28062	1.527363	1.071056	1.102276	0.845427	1.234471	1.292183	0.987602	1.126586	1.162114	2.15216	1.25012346

Gene	Accession	AD1	AD2	AD3	AD4	AD5	AD6	AD7	AD8	AD9	AD10	Average
SAT2	Q96F10	1.225038	0.693593	0.7278	1.361863	1.136699	0.870833	1.51147	0.94885	1.026367	0.869543	1.03720564

Gene	Accession	CTL1	CTL2	CTL3	CTL4	CTL5	CTL6	CTL7	CTL8	CTL9	CTL10	Average
MAPT	P10636	0.937528	0.282927	0.645077	0.708893	0.699925	0.814606	0.676059	0.263107	0.819472	0.925164	0.67727572
AP3D1	O14617-5	1.189014	1.004018	1.054105	1.119465	1.057616	1.085463	1.09973	1.057041	1.090124	0.969371	1.07259463
ADAM11	O75078	1.15366	1.077091	1.123697	1.359266	1.077345	1.163594	1.25849	1.079712	1.106456	1.14935	1.15486628
NPTX2	P47972	1.369001	1.805746	0.935829	2.040809	1.54042	1.350714	2.605226	1.206008	0.873216	1.238329	1.4965299
ABHD16A	O95870	1.116258	1.034193	1.197682	1.143202	1.072314	1.217655	1.003735	1.139784	0.9316	0.892297	1.07487203
RAB3GAP1	Q15042-3	1.025155	1.052377	1.158029	1.077471	1.024099	0.961996	1.117576	1.105187	1.041583	1.005881	1.05693523
MAPT	P10636-4	0.706052	0.21956	0.312088	0.724989	0.46936	0.784621	0.2464	0.135821	0.637903	1.147908	0.53847036
ACTN4	K7EJH8	1.238312	1.143286	0.971221	1.375432	1.027038	1.128619	1.239265	1.159507	1.015076	1.16666	1.14644142
PDHA1	P08559-4	1.286138	1.045726	1.15416	1.23234	1.138016	0.98446	1.017529	1.198962	1.120259	0.997001	1.11745917
BRSK1	Q8TDC3-2	1.093343	1.025577	1.137757	1.055766	1.092548	1.107708	1.084787	1.053395	0.956554	1.107938	1.07153718
DOCK3	Q8IZD9	1.112231	1.025466	1.088483	1.009798	0.958081	1.07004	1.344926	1.119436	1.34162	1.126426	1.11965077
PLXNB1	O43157	0.807629	0.961591	0.900015	0.891409	0.914982	0.761821	0.807194	0.889265	0.940104	0.965491	0.88395013
TIMM44	O43615	1.21551	1.061464	1.250695	1.18794	1.07583	1.058434	1.138865	1.121606	1.037884	1.114318	1.12625465
NRBP1	F8W6G1	1.152626	1.136677	0.905673	0.962248	1.020786	1.139974	1.078632	1.017945	1.038662	1.005846	1.04590709
CSNK1E	P49674	0.821261	1.016489	0.953968	0.964238	0.841096	0.879227	0.963579	0.723331	0.886245	0.874499	0.89239322
GALT	P07902	0.790338	0.794669	0.785397	0.955762	0.772486	1.061735	0.870453	1.021128	0.82023	1.248974	0.91211725
ACTN4	H7C144	1.498032	1.048684	1.030404	0.840594	1.149852	1.433845	1.203123	0.97388	0.851159	0.958951	1.09885249
NCKIPSD	Q9NZQ3	1.055991	1.15296	1.258218	1.128221	1.076838	1.137374	1.028959	0.981969	1.214563	0.843055	1.08781478
NDUFA10	E7ESZ7	1.201482	0.967396	1.408917	1.27586	1.243014	0.97274	0.940213	1.299316	0.95315	0.958904	1.12209916
SLC25A27	O95847	1.134209	1.18912	1.322772	1.252046	1.00909	1.089275	0.955853	1.116482	1.043008	0.860415	1.09722698
ATL2	Q8NHH9	1.130976	1.233581	1.003009	1.13209	0.934407	1.129823	0.937497	1.119841	1.3639	0.9826	1.09677231
AUH	Q13825	1.045806	1.10315	1.270404	1.223447	1.120712	0.977238	1.019728	1.036471	1.136786	0.876664	1.0810406
ACTN4	O43707	1.093643	0.93916	1.094756	1.152518	1.050748	1.119443	1.1208	1.106201	1.02187	1.145466	1.08446055
SCRIB	Q14160-3	0.839877	0.865705	0.856487	0.880793	0.906865	0.953655	0.921495	0.906265	0.853626	1.029175	0.9013943
ATL2	Q8NHH9-4	1.130976	1.233581	1.023411	1.127688	0.934407	1.129823	0.937497	1.119841	1.30655	0.95271	1.0896483
DLG1	Q12959-2	0.702317	0.930497	0.54855	0.61684	0.966179	0.787955	0.967046	0.890871	0.982449	1.0256	0.84183031
CACNA2D1	P54289-2	1.27392	0.953334	1.021474	0.942633	1.096163	1.094179	1.15036	1.069147	1.183393	1.04153	1.08261327
PPP5C	P53041	1.101818	1.024654	0.926015	1.092772	0.954805	1.020725	1.05104	1.111412	1.02166	0.991939	1.02968405
CHERP	J3QK89	1.030417	1.073356	0.713885	0.839636	0.881541	1.176267	0.84327	0.639388	1.022325	0.9316	0.91516853

Gene	Accession	CTL1	CTL2	CTL3	CTL4	CTL5	CTL6	CTL7	CTL8	CTL9	CTL10	Average
SLC30A3	Q99726	1.411356	0.913877	0.851558	1.317621	1.07177	1.385718	1.092455	1.003948	1.228233	1.08089	1.1357425
UNC13A	F8W059	1.244273	0.898271	1.199665	1.161709	1.024637	1.009356	1.030219	1.172065	1.157667	0.994026	1.08918889
SYNGR1	O43759	1.319431	0.804308	1.320146	1.224932	1.167912	1.035578	0.816876	1.23856	1.196967	0.85045	1.09751607
TPD52L1	J3KNE7	0.653442	0.940371	1.047516	0.978612	0.800339	0.92899	0.987402	0.879214	1.051103	0.983664	0.92506509
HMGA1	P17096	0.84306	0.989842	0.521776	0.779594	0.732557	0.944323	0.92569	0.253325	0.78517	0.98503	0.77603671
MAGED2	Q9UNF1	0.956149	1.035928	0.833066	0.933034	1.035912	1.050616	0.792524	0.665613	0.876825	0.938127	0.91177949
HMGA1	P17096-3	0.84306	0.989842	0.487972	0.758711	0.732557	0.932399	0.92569	0.253325	0.78517	0.98503	0.76937562
PMPCB	O75439	1.094737	0.946204	1.169416	1.220759	1.014	1.156244	1.023984	1.160509	0.898955	1.120737	1.08055444
ALCAM	Q13740	0.825378	1.055344	0.889465	0.906136	0.881804	0.82012	1.019461	1.145379	0.86835	0.911524	0.93229617
PMPCB	G3V0E4	1.094737	0.946204	1.150063	1.196868	0.990155	1.183724	1.025211	1.155254	0.898955	1.120737	1.07619081
RNMT	O43148-2	1.383565	1.050749	0.950342	0.940615	1.082687	1.21645	0.870119	0.975564	1.025295	0.99539	1.04907768
ALCAM	Q13740-2	0.823093	1.05062	0.888532	0.903952	0.879934	0.819497	1.019461	1.145379	0.869348	0.923146	0.93229619
HNRNPA2B1	P22626	0.962612	1.015066	0.923806	0.878006	0.81432	0.907266	0.852454	0.884111	0.976186	0.999285	0.92131121
ITGA3	P26006-1	0.265218	0.625671	0.632632	0.889337	0.59527	0.744771	0.808274	0.968745	0.85371	1.148774	0.75324027
ACO2	A2A274	1.158039	1.011638	1.081797	1.252741	1.109753	1.03026	1.020519	1.133988	0.963604	0.915857	1.06781967
ACO2	Q99798	1.158997	1.011841	1.081797	1.252741	1.109753	1.03026	1.020519	1.133988	0.963604	0.915857	1.0679357
MYH7B	A0A087X0T3	0.805382	0.26581	0.475307	0.19583	0.826957	0.837833	0.520002	0.739283	0.80592	0.839974	0.63122973
GK	P32189	1.269705	1.064854	1.290385	1.158029	1.019812	1.078081	1.186888	1.169049	1.09391	0.782935	1.11136477
EPB41L3	Q9Y2J2-2	1.306752	1.067091	1.048784	1.140823	0.808042	1.043019	1.288739	0.910533	1.004111	0.993243	1.06111371
CLTC	A0A087WVQ	1.155585	0.932477	1.093372	1.059951	1.01957	1.033852	1.027416	1.023774	1.101206	0.906734	1.03539362
EFR3B	Q9Y2G0	1.135182	0.88405	1.098924	1.101858	1.14736	1.017894	0.95867	1.030344	1.209415	1.069135	1.06528322
C9orf142	Q9BUH6	0.881237	0.922638	0.824225	0.935193	0.705598	0.844144	0.955699	1.13771	0.817111	0.732502	0.87560585
GRIPAP1	Q4V328	1.038763	0.92834	1.053237	1.013481	1.012554	1.116834	1.048968	0.987772	0.996693	1.005054	1.02016955
SHMT1	P34896	0.892456	0.991479	0.669616	0.731491	0.466971	1.171918	0.774386	0.713673	0.852221	0.987889	0.82521
TTC19	Q6DKK2	1.265848	1.111839	1.27586	1.100367	1.248314	1.151552	0.97388	0.856153	0.738212	1.292682	1.10147072
SYNJ1	C9JFZ1	1.068808	0.919514	0.951442	1.063968	1.043956	1.041551	1.022535	1.008137	0.994142	1.015181	1.01292345
CTNNA1	P35221	0.713012	0.90634	0.838384	0.907065	1.123717	0.938803	0.975673	0.961333	0.809368	1.064171	0.92378648
WBP2	Q969T9	1.088165	1.086007	0.992717	1.161002	1.089341	1.185037	1.301549	0.883391	1.003765	0.863131	1.06541065
FSD1	Q9BTV5	1.038018	0.981303	1.10803	1.194291	1.027878	0.968417	1.067453	1.194496	1.124949	1.147802	1.08526378
ANK2	I6L894	1.047036	0.855445	0.905491	0.949838	0.730354	0.813448	0.943795	0.916919	0.759856	0.939569	0.88617502
MRPL39	Q9NYK5	1.054793	0.917321	1.244507	1.660206	1.084126	1.223339	1.134735	1.025066	1.072843	1.328796	1.17457317
SBF1	O95248-4	1.035128	0.99362	1.044464	1.043276	0.961228	1.001315	0.944373	1.023541	1.079678	0.966997	1.00936201

Gene	Accession	CTL1	CTL2	CTL3	CTL4	CTL5	CTL6	CTL7	CTL8	CTL9	CTL10	Average
ANKRD28	O15084-1	1.069024	0.975288	1.112816	1.092544	1.075491	0.945504	1.220151	1.013268	0.935493	1.170423	1.06100017
YIF1B	Q5BJH7	1.03661	1.153094	2.353134	2.762054	0.901918	0.773379	0.860336	0.817086	0.932774	1.680723	1.32711068
MAPT	P10636-8	0.942137	0.279839	0.841558	1.565889	0.212265	0.207708	0.689345	0.263393	0.817007	0.932658	0.6751798
TECPR1	Q7Z6L1-4	1.121432	1.253836	1.071796	0.989419	1.105846	1.25108	1.088894	1.053241	0.976324	1.050645	1.09625122
GGA3	Q9NZ52	0.924177	0.988958	0.93763	1.034003	0.849322	1.152332	0.85081	0.984179	1.31184	0.981004	1.00142558
MAST3	O60307	1.02722	0.963522	1.129141	0.978274	1.047294	1.179439	1.368618	1.049683	0.946535	1.177982	1.0867709
HMGCS1	Q01581	1.167005	1.074165	0.934854	0.984166	1.533064	1.84348	1.071865	0.846098	0.818693	1.133262	1.14066518
GOPC	Q9HD26	1.073681	1.036765	1.269553	1.175275	0.876286	0.975617	1.036139	1.03462	1.150064	1.179175	1.08071747
MAPRE3	Q9UPY8	1.116348	0.996201	0.995294	1.021998	0.992127	1.062743	1.124587	0.951306	1.091053	1.011083	1.03627402
GAB1	Q13480-2	0.853424	1.248649	0.826487	0.750778	0.721913	0.834932	0.999283	0.233357	0.799463	1.170784	0.84390702
MORF4L2	Q15014	1.115874	0.874314	0.811466	0.664362	0.998974	0.72648	0.924783	0.954043	1.049136	1.010792	0.91302239
ITGB4	P16144	0.802372	0.826319	0.930814	0.95317	0.998717	0.793111	0.744123	0.825658	0.783852	1.074064	0.8732199
HTRA2	O43464	0.998072	0.983454	0.910565	0.957936	1.186812	1.071826	0.859691	1.126144	1.152172	0.978119	1.02247896
LAMP2	P13473-3	0.276226	1.186829	0.896126	0.816581	0.748565	0.666594	0.971318	0.861816	0.872574	1.242412	0.853904
PPP1R3F	Q6ZSY5	0.985041	1.220262	1.101778	1.001425	1.131839	1.309457	0.973221	1.094513	1.051006	0.854266	1.07228092
AIFM1	O95831	1.015029	0.991772	1.102042	1.123218	1.039121	1.042209	0.958747	1.241296	1.077928	0.92238	1.05137405
GABBR1	Q9UBS5	1.219743	0.864407	1.063918	1.052318	1.016549	1.119897	0.902416	1.084367	1.175475	0.969315	1.04684048
DLGAP4	Q9Y2H0-1	1.182711	0.892155	1.080688	1.111654	1.166493	1.073348	1.100566	0.869479	1.035727	0.981958	1.04947801
WASF1	Q92558	1.189253	0.924397	0.999262	1.073988	1.049753	1.085148	0.926108	0.929687	1.131721	0.953451	1.02627692
RBBP7	Q16576-2	0.800285	0.879366	0.973769	1.021508	0.884895	0.979872	0.739283	0.869801	0.944617	0.866718	0.89601155
STK11	Q15831	1.06578	1.020842	1.196345	1.215127	1.041238	1.077028	1.036733	1.016164	1.000944	1.068128	1.07383291
MPV17	P39210	1.105241	1.024033	1.598791	1.000119	1.004374	1.08984	0.954551	1.249577	0.859417	0.930777	1.08167195
FLII	Q13045	0.942908	1.027433	1.029655	1.077654	1.062444	0.982483	0.965985	1.055573	1.056317	1.001264	1.02017163
MAP7	Q14244	0.884525	1.172714	1.026553	1.458592	0.924336	1.204705	0.819293	0.656596	0.870143	1.139826	1.01572828
ZNF207	X6R4W8	0.976153	0.887926	0.894144	0.981109	1.085268	1.071979	0.944007	0.830218	0.898685	0.955864	0.95253515
ARFGAP1	Q8N6T3-2	1.229528	0.916876	1.027304	1.03638	1.041473	0.968036	1.001463	0.967467	0.948115	0.891861	1.00285039
MAP4	P27816	0.868991	1.263237	0.643334	0.833552	0.731891	0.943303	1.021039	0.222629	0.848634	1.273642	0.86502524
PCM1	Q15154	1.177606	1.131513	1.040623	1.094407	1.136699	1.130508	0.938149	0.9206	1.006819	1.017012	1.05939362
PCIF1	Q9H4Z3	0.589251	0.984811	0.668633	0.530163	1.090699	0.606088	1.045569	0.983039	0.931642	0.80665	0.82365446
TIMM21	Q9BVV7	1.084047	1.034119	1.110429	1.346106	1.21661	0.876208	0.877987	0.99388	0.93699	0.993979	1.04703537
VCAN	P13611	0.28143	1.577665	0.707355	0.7278	0.772073	0.644862	1.360672	0.573336	0.696612	1.218049	0.85598513
NONO	Q15233	0.980933	0.949861	1.102117	0.842965	0.873406	1.017629	0.850607	0.809088	0.977278	0.979949	0.93838338

Gene	Accession	CTL1	CTL2	CTL3	CTL4	CTL5	CTL6	CTL7	CTL8	CTL9	CTL10	Average
SRRT	Q9BXP5	0.968278	0.984522	0.85525	0.840594	0.843216	0.888237	0.856639	1.072673	0.980982	1.026186	0.93165767
RBBP7	Q16576	0.800285	0.879366	0.973769	1.021508	0.88261	0.963917	0.732911	0.854604	0.954126	0.8803	0.89433956
CARM1	Q86X55	1.053953	0.937895	0.963613	1.035081	1.072094	1.08799	1.07192	0.953037	0.946374	1.171035	1.02929924
MRPL2	Q5T653	1.29099	1.089969	1.367969	0.984811	1.485465	1.791591	0.822488	1.026214	0.780984	0.895278	1.15357583
SH3GLB2	Q9NR46-2	1.36779	0.891975	0.883392	1.161595	0.837697	1.200621	1.144959	0.935222	1.151753	0.967716	1.05427183
UFD1L	Q92890-1	1.007687	1.058666	0.806054	0.905075	0.936483	1.070563	0.890119	0.778491	0.933144	1.10803	0.94943116
DPM1	H0Y368	1.244742	1.206534	1.009903	1.015315	0.927117	1.048491	1.042696	0.957659	1.292375	1.031031	1.07758624
PPP6R2	O75170-5	1.074903	1.126207	0.966513	0.967591	1.033023	1.091215	1.054092	0.96253	1.025946	0.992396	1.02944163
SLC27A4	Q6P1M0	1.095441	0.965265	1.231368	0.947772	1.124061	1.058132	1.014465	0.985055	0.947536	0.82881	1.01979051
PTGDS	P41222	0.386804	1.352947	0.732636	0.663079	0.930959	0.826336	1.357569	0.816876	0.708824	1.027449	0.88034786
RAD50	Q92878-2	0.746366	1.019471	0.875465	0.938129	1.059004	0.902855	1.047947	1.003904	0.726455	0.959675	0.92792708
MAP4	P27816-7	0.999275	1.590821	0.340267	1.159898	0.467664	1.162504	1.098107	0.202188	0.788464	1.456514	0.92657014
UPF1	Q92900	0.950662	0.872975	1.005705	0.96062	0.951776	0.936093	0.953493	0.950577	1.017261	0.883039	0.94822026
PRAF2	O60831	0.995887	0.900462	1.076104	1.006494	1.226081	1.228236	1.092521	0.998371	1.19405	0.981708	1.06999134
LONP1	P36776	1.09225	0.931888	1.103928	1.038595	1.021219	0.920626	0.996507	1.166207	0.96725	0.964056	1.0202524
CACNA1A	A0A087WW6	1.270355	0.894042	0.976883	1.130586	0.87533	1.160375	0.951306	1.199608	1.209793	0.847517	1.05157965
MPRIP	H0Y2S9	1.247315	0.815951	1.028488	1.015607	1.061475	1.09388	1.072926	0.959041	1.072692	1.092754	1.04601292
DLG1	Q12959-9	1.1434	0.934466	1.09041	1.113388	0.996118	1.017356	1.101367	0.903016	0.965364	1.102603	1.03674866
ACTR1B	P42025	1.145044	1.026131	1.005191	0.909147	1.122056	1.110964	1.123952	0.935032	1.077444	1.106532	1.05614943
ZER1	Q7Z7L7	0.869876	0.772758	0.908455	0.958538	1.053236	0.909371	0.888928	0.855756	0.913288	1.051915	0.9182122
SPTBN4	Q9H254	0.937052	1.153359	1.173116	1.089798	1.011413	1.038847	1.227664	1.06545	1.001139	1.10954	1.08073798
SRRT	Q9BXP5-3	0.969793	0.97575	0.85525	0.840594	0.830822	0.894185	0.856639	1.072673	0.980982	1.026186	0.9302872
SPTBN4	C9JY79	0.935015	1.152857	1.170552	1.082778	1.011413	1.038847	1.226596	1.066073	1.006603	1.107233	1.07979667
PPP6R2	O75170	1.074903	1.126207	0.966513	0.967591	1.040552	1.099998	1.054092	0.96253	1.025946	0.992396	1.03107285
TNPO3	Q9Y5L0	1.054129	1.202104	1.143733	1.178979	0.950474	1.037898	1.096111	1.024238	1.098395	0.925303	1.07113639
MAPK14	Q16539	0.789222	0.94673	1.016703	0.691617	0.930757	0.207708	0.980951	0.879316	0.856442	0.782012	0.80814592
SAFB2	Q14151	1.041175	1.041218	0.786742	0.862758	0.699628	1.212008	0.726106	0.279648	0.905879	1.023199	0.85783612
APOL2	J3KQL8	1.04328	1.427275	1.116243	1.213854	1.132685	0.945841	0.693686	1.100239	1.44826	0.894749	1.10161122
LARS	Q9P2J5	0.954544	0.956674	1.16718	1.177228	1.102809	1.061241	0.917858	0.988257	1.010484	0.980682	1.03169569
EML2	O95834-3	0.832318	0.838026	1.013127	1.221058	0.949119	0.892862	0.71152	0.875751	0.86638	0.911768	0.91119296
UBA5	Q9GZZ9	0.990865	1.004284	0.913899	0.921015	0.879332	0.924895	1.08205	0.907062	0.893512	0.827799	0.9344712
GIT1	Q9Y2X7-3	1.154602	0.927418	1.030053	1.002737	1.006042	0.977043	1.03352	1.025954	1.140953	0.961145	1.02594677

Gene	Accession	CTL1	CTL2	CTL3	CTL4	CTL5	CTL6	CTL7	CTL8	CTL9	CTL10	Average
COG1	Q8WTW3	1.029911	1.030753	1.108491	1.131928	1.208712	1.238495	1.050998	0.922327	1.121355	0.897187	1.07401578
PSMB8	P28062	0.779872	0.96713	0.889282	1.015793	1.446444	0.9013	1.075499	0.943314	0.848914	1.004398	0.9871946
SAT2	Q96F10	0.757877	0.706527	0.797562	1.052568	0.786396	0.682335	0.708931	0.980438	1.039899	0.894191	0.84067242
