# Supplementary material for: Increased intron retention is a post‐transcriptional signature associated with progressive aging and Alzheimer’s disease
Source: Aging Cell. 2019 Mar 13;18(3):e12928. doi: 10.1111/acel.12928 (PMC6516162; doi:10.1111/acel.12928)
Supplement: Supplementary file 12 [file ACEL-18-e12928-s012.pdf]

Table S11: Primers information and summary of sequencing reads

Primer set 1 for visualization on DNA gel

Gene	Genomic Coordinates for IR	Forward Primer (5' - 3')	Reverse Primer (5' - 3')	IR Size	No IR Size
Unc-89	24,003,890 – 24,003,956	GCCCCTCAAGTACGAAGTCA	ATAAAGGTGAACAAGGGCGA	367	300
l(2)efl	23,685,382 – 23,685,475	CATTCTGGATGTGCAGCAGT	GTCACATCGTCCCTCTCCTC	279	186
Bt	767,690 – 767,761	GGCGAAGACGCTGATGAATA	TACACTGGGTTAGGGACGGT	282	210

Primer set 2 for quantification by real-time PCR

Gene	Genomic Coordinates for IR	Forward Primer (5' - 3')	Reverse Primer (5' - 3')
l(2)efl	23,685,382 – 23,685,475	CCAAGTGAGTCCGAATTCCA	CATCACCTAGTGGAAGGATCA
Bt	767,690 – 767,761	ACATATTGTGCGTTTTTGGTATAA	TGGAGCAGCTAAGAATTTTTCAA
Unc-89	24,003,890 – 24,003,956	TCGATGGGGTTTGTTTCTAA	AGTGGTCCTAAACGATTGCAG
lbm	7,049,965 – 7,050,033	AAAAGGTTAACACACTTGAGATTCG	TCTGAAAACATGATCCAAACG
HDAC4	13,270,320 – 13,270,390	CTCAACGTAAGATATAGCTCGATC	GAAGCACTGTCAAAGGATTAGG
Zasp66	8,630,376 – 8,630,516	CGAAGAAGCAAGTTGCCAAT	TCAATTCGAACAAAATAGAAACGA
Actin	internal control	GGCGCAGAGCAAGCGTGGTA	GGGTGCCACACGCAGCTCAT

RNA-sequencing reads

Sample	Raw Reads	Aligned Reads
D10 r1	58559930	54438024
D10 r2	39475158	36284218
D10 r3	44246082	41966666
D20 r1	51123462	47439424
D20 r2	64452922	58841122
D20r3	44216638	41528126
D30 r1	45566308	41733466
D30 r2	43029326	39630056
D30 r3	44118280	41422530
D50 r1	48787024	44293044
D50 r2	52274082	45973894
D50 r3	44340038	41248160

MNase-seq aligned reads count (pooling of 2 replicates; after quality control and duplicates removal)

Day 10	34,616,136
Day 50	34,322,840
